# Supplementary material for: Synthetic studies on the tetrasubstituted D-ring of cystobactamids lead to potent terephthalic acid antibiotics
Source: Commun Chem. 2024 Nov 5;7:252. doi: 10.1038/s42004-024-01337-6 (PMC11538350; doi:10.1038/s42004-024-01337-6)
Supplement: Supplementary file 2 — Supplementary Material [file 42004_2024_1337_MOESM2_ESM.pdf]

Supplementary Information

Synthetic studies on the tetrasubstituted D-ring of cystobactamids lead to potent terephthalic acid antibiotics

Moritz Stappert, Daniel Kohnhäuser, Tim Seedorf, Janetta Coetzee, Katharina Rox, Hazel L. S. Fuchs, Katarina Cirnski, Christian Leitner, Jennifer Herrmann, Andreas Kirschning, Rolf Müller, Mark Brönstrup

Table of Contents

Table of Contents 1
1 Chemistry and physicochemistry 3
1.1 Materials and Methods 3
Solvents and eluents 3
Reactant/reagent/product handling and general reaction setup 3
Reaction control 3
Column chromatography 4
HPLC/LCMS 4
NMR spectroscopy 5
HRMS 6
Determination of optical rotation 7
Determination of log D 7
1.2 Synthetic procedures 8
AB-fragments 8
AB-central AA-fragments 9
AB-central AA-C-fragments 10
Ring D analogues 12
Ring E analogues 45
DE-fragments 47

CD-fragments 73
CDE-fragments 74
AA-CDE-fragments and cystobactamid precursors 96
Final cystobactamids 110
2 Biology 135
2.1 Materials and Methods 135
MIC testing 135
Determination of in vitro ADME properties 135
HPLC-MS/MS analysis of cystobactamids 135
Human and mouse plasma 137
Animals 137
PK study 137
Bioanalysis of pharmacokinetic and pharmacodynamic samples 137
AlbD enzyme assay 139
3 Supplementary references 142
4 NMR Spectra 143

1 Chemistry and physicochemistry

1.1 Materials and Methods

Solvents and eluents

Solvents for synthesis and purification were used as delivered, if not stated else. Dry solvents were used:

1) bought, involving the following dry solvents (manufacturer): acetonitrile, acetone, dimethylformamide, dimethylsulfoxide, 1,4-dioxane, isopropanol, methanol, methylene chloride, pyridine, tetrahydrofurane, toluene (Acros Organics)

2) taken from a MBraun solvent purification system (MeCN, DMF, Et_2O), 3) freshly destilled over sodium (benzophenone as indicator) (THF), or over KOH (TEA), or at 60°C (petrol ether).

Organic solvents for HPLC were used as HPLC grade and degassed before use. Water was either used as HPLC grade or purified with Milli-Q water filter device (Elga Veolia Purelab flex) before use.

Reactant/reagent/product handling and general reaction setup

Non-volatile solid reactants and reagents were dried under high vacuum before use or used as supplied, where possible. In addition to the synthesized building blocks, some reactant/ reagents were also supplied as noticed. Reactions were conducted in glass ware (dried, where necessary) using magnetic stirring. The use of inert gas is indicated where it was employed. Light sensitive reactions were carried out under light exclusion. For reactions under microwave irratiation a CEM Discover S-Class was used with a power maximum of 300 W. Products were isolated from their solutions at a rotary evaporator (Heidolph Hei-Vap Advant) using a membrane pump (Vacuubrand PC3001 VARIO select). For storage, products were transferred to small flasks as solution, followed by renewed removal of the solvent. Further drying was conducted under high vacuum with an oil rotary vane pump (Vacuubrand RZ 2.5).

Reaction control

The reaction mixtures were monitored by using thin-layer chromatography (TLC) and/or LCMS. For sample extraction, a truncated metal canula was used and the sample was diluted before analysis. TLC was conducted with silica-coated aluminum plates and fluorescence-indicator (TLC Silica gel 60 F_{254}, Merck (Darmstadt)). After

development, the TLC was radiated with UV light (λ=254 nm or λ=365 nm) for evaluation. For a closer assignment of the spots to expected products the TLC-plates were dyed using Ninhydrin-solution in EtOH (1.0 g Ninhydrin in 100 mL EtOH) for amines, a solution of $FeCl_3$ in EtOH (50 mL) and water (50 mL) or hydrochloric acid (0.1 M, 50 mL) for phenols, a bromo cresol green solution (40 mg in aqueous NaOH (0.1 M, 100 mL)) for carboxylic acids, or a $KMnO_4$-solution (1.0 g in a solution of K_2CO_3 (2.0 g) in H_2O (100 mL)) for oxidizable compounds. Other stains involved vanillin or anisaldehyde as detection reagents. Subsequently, the plates were treated with a heat gun where necessary.

Crude products and purified products were also analyzed on an Agilent LCMS device (Agilent Technologies 1260 Infinity II) with MeCN/H_2O as eluent system using LCMS method 6 (see attachment). Mass spectra were generated in positive mode with ESI as ionization method with a Quadrupole mass spectrometer (Agilent Technologies 6130 ES superior). Solvents for LCMS were used with Formic Acid (0.1%) added to both MeCN and H_2O before measurement.

Column chromatography

Reaction products were purified and isolated using manual or automated flash-column chromatography (FCC). For this purpose, silica 60 gel (particle size: 40-63 µm, otherwise noted) was used as stationary phase. The dimensions of the silica column was adapted to the reaction scale and the estimated separation efficiency. The ratio of silica to the estimated amount of reaction material is given, where necessary. The composition of eluents was adjusted to the separation using analysis of product mixtures by TLC. Fractions were analyzed by TLC, spot detection and staining was analogous to TLC reaction control. Automated flash column chromatography was conducted with the flash purification system Sepacore® by Büchi® or Biotage® SP using prepacked cartridges (puriFlash® by Interchim or Chromobond® by Macherey-Nagel).

HPLC/LCMS

Preparative HPLC was implemented:

1) on a Thermo Scientific Ultimate 3000 HPLC-system using either a basic or an acidic eluent system. It was applied to purify final cystobactamids. Further details for sample preparation is mentioned in section 5.2.

2) or on a semi-preparative WATERS Alliance 2695 HPLC-system with a 996 diode array detector (λ = 200-350 nm) and a MACHEREY-NAGEL Nucleodur C18 ISIS column (5 µm, 250 mm, diameter = 8 mm). Mass detection was conducted with a WATERS Quattro micro API mass spectrometer in negative ionization mode

Analytical LCMS was conducted on an Agilent LCMS device (Agilent Technologies 1260 Infinity II) as mentioned in section 5.1.3, using method 6 (see attachment).

The eluent programs used are listed in the attachment.

NMR spectroscopy

NMR spectra were measured in deuterated solvents either with a 500 MHz NMR-spectrometer (Bruker Advance-III HD 500 MHz, Frequencies: ^{1}H NMR: 500 MHz, ^{13}C NMR: 126 MHz), a 700 MHz NMR-spectrometer (Bruker Advance-III HD 700 MHz, Frequencies: ^{1}H NMR: 700 MHz, ^{13}C NMR: 176 MHz), a Bruker Ascend 600 MHz with Avance Neo console, Ultrashield 500 MHz with Avance-III HD console, Ascend 400 MHz with Avance- III console, Ascend 400 MHz with Avance-III HD console or Ultrashield 400 MHz with Avance-I console. All spectra were measured at room temperature and ^{13}C NMR-spectras were measured ^{1}H-decoupled. Chemical shifts (δ) of the compounds are given in ppm, the coupling constants J are given in Hz. The spectra were always referenced to the respective residual solvent peak given in Table S1.

Table S1. Reference peaks for NMR data depending on solvent.

NMR-solvent	^{1}H [ppm]	^{13}C [ppm]
$CDCl_3$	7.26	77.16
DMSO-*d6*	2.50	39.52
acetone-*d6*	2.05	29.84
THF-*d8*	3.58	65.57
MeOH-*d4*	4.87	49.00

Multiplicities are given according to Table S2:

Table S2. Abbreviations of the multiplicities for NMR data.

**multiplicity**	**abbreviation**
singlet	s
doublet	d
doublet of doublet	dd
doublet of triplet	dt
triplet	t
quartet	q
heptet	hept
multiplet	m
broad	br

Yields were determined by weighting and also corrected by NMR when solvent impurities would substantially falsify yield. The correction was done using following equation:

$$Y_{corrected} = Y_{measured} \frac{M_P J_P N_I}{M_P J_P N_I + M_I J_I N_P}$$

M_P : product molar mass, J_P : integral of observed product peak, N_P : number of protons assigned to product peak, M_I : molar mass of main impurity, J_I : integral of observed impurity peak, N_I : number of protons assigned to impurity peak

For spectra of key substances in the synthesis, assignment of signals to the corresponding atom was verified by 2D NMR validation.

In cases of common solvent impurities in the NMR sample, the observed number of signals in NMR may not correspond with the expected number. Signals assignable to known impurities (solvent, reactant/ reagent etc.)[1] are not listed.

HRMS

HRMS spectra were recorded on a Bruker maXis HD spectrometer, a Micromass LCT with lock-spray unit and injection via loop modus in a Waters (Alliance 2695) HPLC device, or a Micromass Q-TOF in combination with a Waters Aquity UPLC device. Mass signals are given in their *m/z* values with the relative intensities in brackets together with the calculated mass.

Determination of optical rotation

The specific optical rotation [α] was measured with a polarimeter type 341 from Perkin-Elmer at λ = 589.3 nm (sodium D line) in a 10 cm quartz cuvette. It is given in 10^{-1} cm^2 g^{-1}. The concentration c is given in 10 mg mL^{-1}.

Determination of log D

Log D values were determined for cystobactamids **4**, **10**, **11**, **42** and **45b**. Methods for determination of log D values were adapted from[2, 3]. PBS buffer (pH=7.4, pH meter checked) and octanol were mutually saturated by shaking in separating funnel. Phases separated at least 24 hours. Every sample was measured in glass vials (0.5 mL, 2 mL, 4 mL, 8 mL). The cystobactamids were diluted as DMSO solution with PBS buffer to 0.10 mM (standard solution). From this, phase mixtures of PBS/octanol were made in at least two of these ratios: 1:1, 5:1, 10:1, 50:1. The phase mixtures were shaken strongly for at least 10 min per hand, then left to separate for at least 8 h. The lower phase was extracted with an Eppendorf pipet tip after careful removal of octanol residues. The aequeos fraction was measured by UV-absorption in HPLC device (Shimadzu Prominence). All samples were measured in such a way that at most half of the maximum UV intensity was reached. Log D values were determined by using following formula:

$$\log D = \log\left(\left(\frac{A_{\mathrm{st}}}{A_w}\cdot\frac{v_{\mathrm{inj}(w)}}{v_{\mathrm{inj(st)}}}r - 1\right)\frac{V_w}{V_o}\right)$$

log D: pH dependent log P, Ast: Peak area for standard solution, Aw: Peak area for sample solution, $v_{inj(w)}$: injection volume for aequos phase, $v_{inj(st)}$: inj. vol. for standard solution, r: dilution factor of standard solution, V_w/V_o: volume fraction of aequos phase and organic phase.

For every measurement, triple determination was conducted with varying injection volumina and arithmetic mean was calculated.

1.2 Synthetic procedures

The following syntheses are sorted by the corresponding fragments, using the nomenclature used for cystobactamids.

AB-fragments

Synthesis of 4-(4-cyanobenzamido)benzoic acid ***(86)***[4]

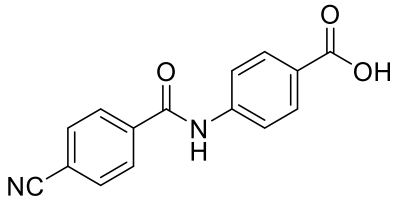

4-amino benzoic acid (2.00 g, 14.6 mmol, 1.00 Eq) was solved in DMA (44 mL) and DMAP (71 mg, 0.58 mmol, 0.040 Eq) was added. 4-cyano benzoyl chloride (2.41 g, 14.6 mmol, 1.00 Eq) was added and the reaction mixture stirred 18 h at RT. The mixture was added to a heavily stirred brine solution (150 mL) which was then filtrated (Note: Precipitate forms a very fine powder, small pore size required). The colorless precipitate was dried u.r.p. overnight to give a fine colorless powder. Because the precipitate contained greater amounts of NaCl, the solid was resuspended, filtered and washed with water several times and dried u.r.p. overnight to give a fatty colorless solid, 2.66 g, 69%.

^{1}H NMR (500 MHz, DMSO): δ = 12.79 (s, 1H), 10.74 (s, 1H), 8.11 (d, *J* = 8.4, 2H), 8.04 (d, *J* = 8.2, 2H), 7.96 (d, *J* = 8.9, 2H), 7.91 (d, *J* = 8.9, 2H).

^{13}C NMR (126 MHz, DMSO): δ = 166.9, 164.6, 142.9, 138.6, 132.5, 130.3, 128.7, 125.9, 119.7, 118.3, 114.1.

AB-central AA-fragments

Synthesis of (S)-2-(4-(4-cyanobenzamido)benzamido)pent-4-ynoic acid ***(88)***

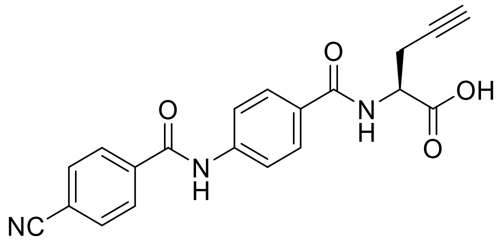

a) Under Argon atmosphere: (*S*)-Propargyl glycine (340 mg, 3.01 mmol, 1.00 Eq) was suspended in MeOH (10 mL) and TMS-Cl (1.9 mL, 15 mmol, 5.0 Eq) was added. The clear solution was stirred overnight before being concentrated u.r.p. and coevaporated several times with MeCN. The intransparent viscous liquid was directly coupled in the next step.

b) Modified from[5, 6]: 4-(4-cyanobenzamido)benzoic acid **86** (762 mg, 2.86 mmol, 1.00 Eq) and HATU (1.09 mg, 1.00 Eq) were solved in dry DMF (20 mL) and DiPEA (1.0 mL, 2.0 Eq) was added. The reaction was stirred for 2 h while reaction was monitored by LCMS. Then a solution of the material from part a) in dry DMF (7 mL) and DiPEA (1.0 mL, 2.0 Eq) were added and the reaction stirred 1.5 h. The solution was neutralized with HCl (5.7 mL, 2 M) and concentrated to a volume of ~5 mL. (Note: smaller amounts of acid may increase yield). DCM (~5 mL) and water were added to the concentrate until great amounts of precipitate were formed. The precipitate was filtered through pore size 4 and washed several times with water before it was dried u.r.p. to give ~0.94 g crude product.

The filtrate solution was extracted with DCM (3x) and EA (3x), organic phases were combined and dried over Na_2SO_4. The solution was concentrated u.r.p. before water was added to the residual solution (containing DMF) to result in precipitation. The precipitate was filtered off and washed with water. This precipitate and the precipitate from before were combined.

The collected precipitates were suspended in a THF/water mixture (20/20 mL) and LiOH*H_2O (0.96 g, 8.0 Eq) was added. The reaction stirred for 1.5 h before solving the reaction solution between H_2O and EA (80 mL each). (Note: Here the product stays in the alkaline aqueous phase and reactant residuals stay in the organic phase). The aqueous phase was then acidified with HCl (2 M, 15 mL) and extracted with EA

(1x80 mL, 2x50 mL). The organic phases were combined afterwards, dried over Na_2SO_4 and solvents were removed u.r.p. and by coevaporation (3-4x) with chloroform and heptane. (Note: acetone or MeOH should be avoided here). The product was obtained as a colorless fatty solid (849 mg, 82% o2s).

^{1}H NMR (500 MHz, DMSO): *δ* = 12.88 (br s, 1H), 10.70 (s, 1H), 8.70 (d, *J* = 7.9, 1H), 8.13 (d, *J* = 8.7, 2H), 8.04 (d, *J* = 8.5, 2H), 7.93 – 7.87 (m, 4H), 4.54 (ddd, *J* = 8.9, 7.8, 5.4, 1H), 2.87 (t, *J* = 2.6, 1H), 2.80 – 2.67 (m, 2H).

^{13}C NMR (126 MHz, DMSO): *δ* = 172.0, 165.7, 164.5, 141.6, 138.7, 132.5, 129.0, 128.6, 128.2, 119.6, 118.3, 114.0, 80.9, 72.8, 51.7, 20.8.

HRMS (ESI): calculated for $[M+H]^+$: 362.1135, found: 362.1135.

AB-central AA-C-fragments

*Synthesis of tert-Butyl (S)-4-(2-((((9H-fluoren-9-yl)methoxy)carbonyl)amino)pent-4-ynamido)benzoate acid (**S1**)*

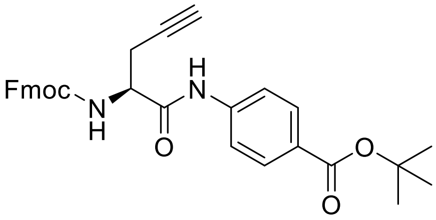

tert-butyl 4-aminobenzoate (200 mg, 1.0 mmol, 1.0 Eq) and (S)-2-((((9*H*-fluoren-9-yl)methoxy)carbonyl)amino)pent-4-ynoic acid (500 mg, 1.5 mmol, 1.4 Eq) were added to a dry flask and were dried under high vacuum. Dry pyridine (0.25 mL, 3.0 eq) and dry ethyl acetate (10 mL) were added under nitrogen atmosphere. The reaction mixture was cooled down to 0°C. T3P solution (1.4 mL, 50 wt % in ethyl acetate, 2.3 Eq) was added very slowly while keeping the temperature below 0°C. The reaction was stirred at 0°C for 4 h and controlled over LCMS. After completion, the reaction was quenched with HCl (10 ml, 1 M) and brine (30 mL) and extracted with ethyl acetate (3x10 ml). The combined organic phases were washed with brine and the solvent was removed under reduced pressure. The crude product was purified by flash chromatography (PE/ EtOAc). A colorless glass was obtained, yield: 519.3 mg, 98 %.

^{1}H NMR (500 MHz, $CDCl_3$): *δ* = 8.30 (br s, 1H), 7.95 (d, 2H, *J* = 8.8 Hz), 7.77 (d, 2H, *J* = 7.6 Hz), 7.58 (d, 2H, *J* = 7.5 Hz), 7.55 (d, 2H, *J* = 8.8 Hz), 7.40 (t, 2H, *J* = 7.5 Hz),

7.30 (t, 2H, *J* = 7.3 Hz), 5.57 (br s, 1H), 4.54 – 4.43 (m, 3H), 4.24 (t, 1H, *J* = 6.7 Hz), 2.95 – 2.81 (m, 1H), 2.74 – 2.65 (m, 1H), 2.15 (t, 1H, *J* = 2.6 Hz), 1.59 (s, 9H).

^{13}C NMR (126 MHz, $CDCl_3$): δ = 168.1, 165.3, 143.7, 143.6, 141.5, 140.9, 130.8, 128.3, 128.0, 127.3, 125.1, 120.3, 119.2, 81.2, 72.5, 67.6, 54.2, 47.3, 28.4, 22.1.

HRMS (ESI): calculated for [M+H$^+$]: 511.2233, found: 511.2228.

*Synthesis of tert-butyl (S)-4-(2-(4-(4-cyanobenzamido)benzamido)pent-4-ynamido)benzoate (**S2**)*

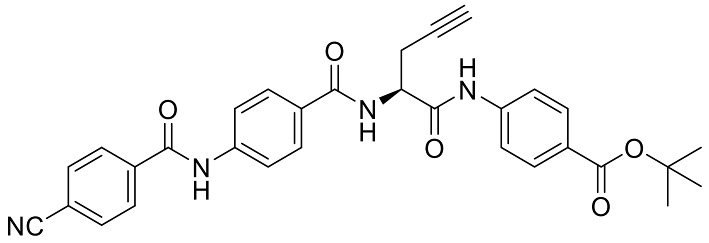

tert-butyl (S)-4-(2-((((9H-fluoren-9-yl)methoxy)carbonyl)amino)pent-4-ynamido)benzoate **S1** (510.0 mg 1.0 mmol, 1.0 eq) was dissolved in MeCN (1 mL) and diethylamine (1.5 mL, 15 Eq) at 0°C and was stirred for 1 h. The solvent was evaporated under reduced pressure. MeCN (1 mL) was added to the residue and the solvent was removed again. This was repeated twice. The flask was dried at the vaccum pump overnight. 4-(4-cyanobenzamido)benzoic acid (300.0 mg, 1.1 mmol, 1.1 eq) and HATU (430.0 mg, 1.1 mmol, 1.1 eq) were added and the flask was dried under vacuum pump. dry DMF (3.0 ml) and dry DIPEA (0.35 mL, 2.0 eq) were added under nitrogen atmosphere and the reaction was stirred at 0°C. The reaction was controlled by LCMS. After completion, HCl (3 mL, 1M) and brine (15 mL) were added. The inorganic layer was extracted with ethyl acetate (4x6 mL). The organic phases were combined and washed with brine. The crude product was purified by chromatography. A white solid (516 mg crude) was obtained and directly used in next step.

*Synthesis of (S)-4-(2-(4-(4-cyanobenzamido)benzamido)pent-4-ynamido)benzoic acid (**S3**)*

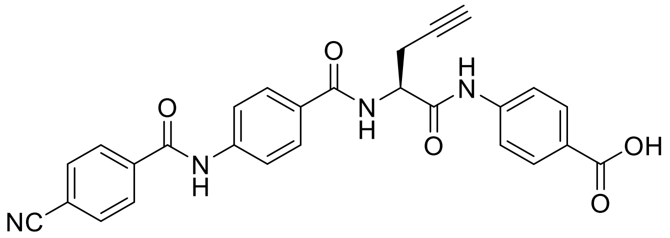

Crude tert-butyl (S)-4-(2-(4-(4-cyanobenzamido)benzamido)pent-4-ynamido)benzoate **S2** (516 mg, 0.96 mmol, 1.0 Eq) was added to a dry flask and further dried at the vaccum pump. Dry DCM (6.4 mL) was added under nitrogen atmosphere and the solution was cooled down to 0°C. Trifluoroacetic acid (3.9 mL, 53 Eq) was added under nitrogen atmosphere. The solution was stirred for 3 h at 0°C and controlled over LCMS. After the reaction was completed, the solvent was removed under reduced pressure. The residue was dissolved in DCM and the solvent was removed again. This was repeated twice. The crude product was purified by reversed-phase chromatography. The product was obtained as a white solid, yield: 334.0 mg, 70 % o2s.

^{1}H NMR (700 MHz, DMSO): *δ* = 12.72 (br s, 1H), 10.70 (s, 1H), 10.55 (s, 1H), 8.75 (d, 1H, *J* = 7.5 Hz), 8.13 (d, 2H, *J* = 8.5 Hz), 8.04 (d, 2H, *J* = 8.5 Hz), 7.95 (d, 2H, *J* = 8.8 Hz), 7.92 – 7.87 (m, 4H), 7.75 (d, 2H, *J* = 8.8 Hz), 4.78 (dd, 1H, *J* = 7.7 Hz, 14.7 Hz), 2.92 (t, 1H, *J* = 2.6 Hz), 2.82 – 2.71 (m, 2H).

^{13}C NMR (176 MHz, DMSO): *δ* = 169.7, 166.9, 165.9, 164.5, 142.8, 141.7, 138.7, 132.5, 130.4, 128.9, 128.6, 128.4, 125.4, 119.5, 118.7, 118.3, 114.1, 80.6, 73.2, 53.5, 21.4.

Ring D analogues

*Synthesis of 2,5-dihydroxy-4-nitrobenzoic acid **(12)***

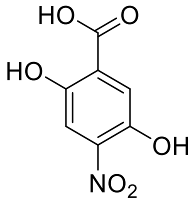

Modified from[7]: 3-Hydroxy-4-nitrobenzoic acid (5.00 g, 27.4 mmol, 1.00 Eq) was dissolved in aqueous NaOH (2 M, 100 mL) and a solution of $K_2S_2O_8$ (7.76 g, 1.05 Eq) in H_2O (150 mL) was added. The reaction mixture was stirred at room temperature for 5 d. The reaction mixture was then cooled in an ice bath and strongly acidified by adding conc. H_2SO_4 (~15 mL). The resulting precipitate was removed by filtration and the product was extracted with small amounts of water. The aqueous solution was then refluxed for 1 h. The resulting precipitate was washed with small amounts of water and transferred with acetone in a flask, solvents were removed u.r.p. and the solid was dried in vacuum. A red-brown solid was obtained, yield: 0.961 g, 18%. Analytical data was in agreement with literature.

Synthesis of 6-hydroxy-2,2-dimethyl-7-nitro-4H-benzo[d][1,3]dioxin-4-one ***(S4)***

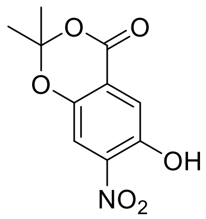

Modified from[8]: 2,5-dihydroxy-4-nitrobenzoic acid **12** (400 mg, 2.01 mmol, 1.00 Eq) was solved in TFA (2.6 mL) in a 2-neck-flask and TFAA (1.96 mL, 7.00 Eq) and acetone (1.0 mL, 7.0 Eq) were added. The mixture was refluxed for 6 d. The reaction mixture was concentrated carefully u.r.p., solved in EtOAc (20 mL) and washed with water (3x10 mL) before washing with saturated $NaHCO_3$-solution (1x15 mL). EtOAc (15 mL) was added and the organic phase was washed again with brine (1x15 mL) before drying over Na_2SO_4 and concentrating u.r.p. The black mush was withdrawn on Celite and purified by FCC (50x reactant mass, PE/EA, 80/20, silica particle size: 63-200 µm). After solvent removal u.r.p. a dark brown solid was obtained, yield: 328 mg, 68%. The analytical data was in agreement with literature.

Synthesis of 6-isopropoxy-2,2-dimethyl-7-nitro-4H-benzo[d][1,3]dioxin-4-one ***(13)***

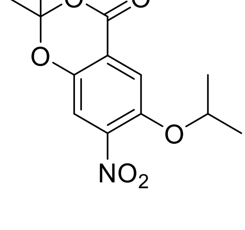

Modified from[9]: In a nitrogen flushed flask PPh_3 (362 mg, 1.10 Eq) was solved in dried THF (2 mL) at 0°C and DIAD (0.27 mL, 1.1 Eq) was added. To the resulting yellowish suspension, **S4** (300 mg, 1.25 mmol, 1.00 Eq) was added as a solution in dry THF (1.5 mL), its container was washed with 0.5 mL, that was also added. *i*PrOH (145 µL, 1.50 Eq) dried over molecular sieve (4 Å) was added and the reaction was then stirred 2 h at RT. To the mixture PE was added (30 mL) and the precipitate was filtered through celite. The solution was freed from solvent over silica u.r.p. and purified by FCC (PE/EA, 85/15, silica particle size: 63-200 µm) to give 319 mg, 90% yield of a yellowbrown liquid that slowly formed crystals.

^{1}H NMR (500 MHz, $CDCl_3$): δ = 7.64 (s, 1H), 7.29 (s, 1H), 4.70 – 4.61 (m, 1H), 1.38 (d, J = 6.1, 6H).

^{13}C NMR (126 MHz, $CDCl_3$): δ = 159.8, 148.9, 146.2, 145.8, 116.9, 116.4, 114.0, 107.6, 73.9, 25.9, 21.9.

HRMS (ESI): calculated for $[M+H]^+$: 282.0973, found: 282.0978.

Synthesis of 2-(allyloxy)-5-isopropoxy-4-nitrobenzoic acid ***(14)***

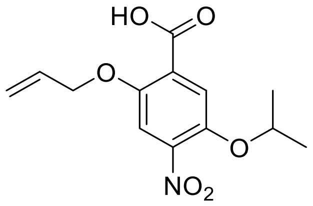

Modified from[7]:

a) **13** (300 mg, 1.07 mmol, 1.00 Eq) was solved in MeOH (25 mL), sodium methoxide (173 mg, 3.00 Eq) was added and the reaction mixture stirred for 15 min at RT. HCl (1 M, 3 mL) was added and the solution was concentrated u.r.p.. The residue was solved in MTBE (25 mL) and washed with H_2O (2x15 mL). The organic layer was dried with Na_2SO_4 and solvents were removed u.r.p. to afford a dark-yellow solid. Yield: 262 mg, 96%.

b) The crude product (250 mg, 980 µmol, 1.00 Eq) was solved in DMF (8 mL) and allyl bromide (169 µL, 2.00 Eq) and K_2CO_3 (203 mg, 1.50 Eq) were added. (Note: Reactant and product have very similar Rf-values with PE/EA) Reaction was finished after 3.5 h (reaction shows kinetic behaviour of 0. order) and the suspension was diluted between

MTBE (100 mL) and water (100 mL). The aqueous phase was extracted with MTBE (1x50 mL) before adding NaCl until saturation. The aqueous phase was further extracted with MTBE (2x50 mL). Solvent was removed u.r.p. from combined organic phases to obtain product: 299 mg, >98%, yellow solid. The product was directly used in next step.

c) The crude product was solved in THF/water (2.5 mL/2.5 mL) and LiOH monohydrate (178 mg, 4.20 Eq) was added. The reaction was stirred for 3 h, before HCl (1 M, 10 mL) and 20 mL water were added and the aequos phase was extracted with MTBE (3x20 mL). Solvents were removed from organic phase u.r.p. to give a yellow-brownish solid (276 mg, 96% o3s).

^{1}H NMR (500 MHz, $CDCl_3$): δ = 7.87 (s, 1H), 7.44 (s, 1H), 6.13 – 6.01 (m, 1H), 5.55 – 5.43 (m, 2H), 4.78 (dt, J = 5.7, 1.3, 2H), 4.73 – 4.64 (m, 1H), 1.37 (d, J = 6.1, 6H).

^{13}C NMR (126 MHz, $CDCl_3$): δ = 164.3, 150.1, 145.7, 143.8, 130.5, 122.5, 121.4, 121.3, 110.7, 73.9, 72.0, 21.9.

HRMS (ESI): calculated for $[M+H]^+$: 282.0973, found: 282.0978.

Synthesis of 2-fluoro-3-hydroxy-4-nitrobenzaldehyde ***(16a)***

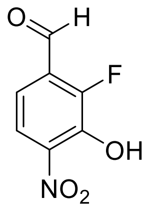

2-fluoro-3-hydroxy-benzaldehyde **15a** (150 mg, 1.07 mmol, 1.00 Eq) was solved in MeCN (11 mL) and HNO_3 (fum., 0.45 mL, 10 Eq) was added dropwise at 0°C. The reaction was stirred for 30 min at 0°C while being screened continuously by LCMS and then quenched by adding sat. $NaHCO_3$ sol. (20 mL). The pH was adjusted to ~1-3 (color change!) with HCl (1 M). The aqueous phase was extracted with EA (2x20 mL), the combined organic phases were washed with brine (1x10 mL), dried over Na_2SO_4 and the solvent was removed u.r.p.. The correct regioisomer was isolated by FCC (solid-loading, 85x theoretical product mass, PE/EA/AcOH, 85/10/5) to give yellow crystals (yield: 76 mg, 38%).

¹H NMR (500 MHz, $CDCl_3$): *δ* = 10.51 (s, 1H), 10.45 (d, *J* = 0.8, 1H), 8.01 (ddd, *J* = 9.0, 2.1, 0.9, 1H), 7.45 (dd, *J* = 9.1, 6.0, 1H).

¹³C NMR (126 MHz, $CDCl_3$): *δ* = 185.6 (d, *J* = 7.0, COH), 156.3, 154.2, 145.6, 129.5, 128.8, 120.1 (d, *J* = 4.0, C_{Ar}-H), 116.7 (C_{Ar}-H).

¹⁹F NMR (471 MHz, $CDCl_3$): *δ* = -139.4.

HRMS (ESI): calculated for $[M+H]^+$: 186.0197, found: 186.0199.

Synthesis of 2-chloro-3-hydroxy-4-nitrobenzaldehyde ***(16b)***

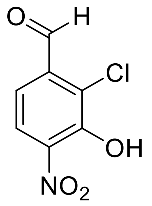

2-chloro-3-hydroxy-benzaldehyde **15b** (400 mg, 2.55 mmol, 1.00 Eq) was solved in MeCN (25 mL) and HNO_3 (fum., 1.1 mL, 10 Eq) was added dropwise at 0°C. The reaction was stirred for 30 min at 0°C while being screened continuously by LCMS and then quenched by adding sat. $NaHCO_3$ sol. (40 mL) and diluted between water/EA (75 mL each phase). The pH of the aqueous phase was adjusted to ~1 (color-change from orange to yellow!) with HCl(aq., 2 M). The aqueous phase was extracted with EA (2x30 mL), the combined organic phases were washed with brine (1x25 mL), dried over Na_2SO_4 and the solvent was removed u.r.p.. The correct regioisomer was isolated by FCC (solid-loading, 75x theoretical product mass, PE/EA/AcOH, 85/10/5->50/50/0) to give yellow crystals (yield: 225 mg, 44%).

¹H NMR (500 MHz, Acetone): *δ* = 10.52 (d, *J* = 0.9, 1H), 8.23 (dd, *J* = 8.9, 0.9, 1H), 7.54 (d, *J* = 8.9, 1H).

¹³C NMR (126 MHz, Acetone): *δ* = 189.5 (COH), 152.0, 138.7, 138.6, 128.7, 124.5 (C_{Ar}-H), 119.2.

HRMS (ESI): calculated for $[M+H]^+$: 201.9902, found: 201.9901.

Synthesis of 2-bromo-3-hydroxy-4-nitrobenzaldehyde ***(16c)***

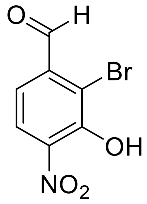

2-bromo-3-hydroxy-benzaldehyde **15c** (100 mg, 0.497 mmol, 1.00 Eq) was solved in MeCN (5.0 mL) and HNO_3 (fum., 0.21 mL, 10 Eq) was added dropwise at 0°C. The reaction was stirred for 30 min at 0°C while being screened continuously by LCMS and then quenched by adding sat. $NaHCO_3$ sol. (10 mL) and diluted between water/EA (50 mL each). The organic phase was collected and the pH of the aqueous phase was adjusted to ~4-5 (color-change from orange to yellow!) with HCl (aq.). The aqueous phase was then extracted with EA (2x30 mL), the combined organic phases were dried over Na_2SO_4 and solvent was removed u.r.p.. The correct regioisomer was isolated by FCC (solid-loading, 100x theoretical product mass, PE/EA/AcOH 90/5/5->85/10/5). Yield: 54 mg, 44%.

^{1}H NMR (500 MHz, $CDCl_3$): δ = 11.28 (s, 1H), 10.46 (d, J = 0.9, 1H), 8.21 (dd, J = 8.9, 0.9, 1H), 7.54 (d, J = 8.9, 1H).

^{13}C NMR (126 MHz, $CDCl_3$): δ = 190.6 (COH), 152.9, 139.4, 136.6, 124.2 (C_{Ar}-H), 119.6 (C_{Ar}-H), 119.0.

HRMS (ESI): calculated for $[M+H]^+$: 245.9397/ 247.9376, found: 245.9395/ 247.9375.

Synthesis of 2-fluoro-3-isopropoxy-4-nitrobenzoic acid ***(17a)***

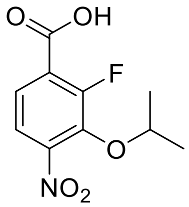

a) Modified from[10]: 2-Fluoro-3-hydroxy-4-nitro-benzaldehyde **16a** (72 mg, 0.39 mmol, 1.0 Eq) was suspended in a *t*BuOH/THF-mixture (1.6 mL each) and 2-Me-2-butene (0.41 mL, 10 Eq) was added. $NaClO_2$ (80%, 88 mg, 2.0 Eq) solved in aqueous NaH_2PO_4-sol. (1 M, 0.74 mL) at 0°C was added dropwise in 3 portions after 0 min,

15 min and 1 h. 2-Me-2-butene (0.21 mL, 5 Eq) was added after 3 h and $NaClO_2$ (80%, 3x22 mg, 3x0.5 Eq) was added again in aqueous NaH_2PO_4-sol. (1 M, 3x0.18 mL) after 3 h, 3.5 h and 4 h. After 5 h and screening by LCMS the reaction mixture was concentrated u.r.p. to ~half volume, diluted between EA/HCl (0.5 M) (30 mL each), the pH was controlled to be ~1 and the aqueous phase was extracted with EA (2x20 mL). The organic phases were collected, dried over Na_2SO_4 and solvents were removed u.r.p. to get a yellow solid directly used in next reaction.

b) PPh_3 (224 mg, 2.2 Eq) was solved in dry THF (1.5 mL) and DIAD (0.17 mL, 2.2 Eq) was added at 0°C. After precipitation *i*PrOH (75µL, 2.5 Eq) was added and then the product from a) (78 mg, 1.0 Eq) was added as solution in dry THF (2.5 mL) at 0°C. The reaction was stirred at RT overnight and then LiOH*H_2O (163 mg, 10 Eq) and H_2O (4.0 mL) were added to the reaction solution. The mixture was then stirred vigourously at RT for 3 h while being screened with TLC. After 2.5 h the mixture was diluted between EA and NaOH (0.5 M) (30 mL each). The organic phase was extracted with NaOH (0.5 M, 2x10 mL) and organic phase was analysed for product residuals by TLC. The combined aqueous phases were washed with DCM (2x10 mL) and then acidified with HCl (2 M, 20 mL). The product was now extracted with EA (3x20 mL), the organic phases were dried over Na_2SO_4 and a beige solid was obtained (52 mg, 44% o2s) after solvent removal u.r.p..

^{1}H NMR (500 MHz, Acetone): δ = 7.80 (dd, J = 8.7, 6.4, 1H), 7.73 (dd, J = 8.7, 1.7, 1H), 4.69 (heptd, J = 6.2, 1.3, 1H), 1.34 (dd, J = 6.1, 0.9, 6H).

^{13}C NMR (126 MHz, Acetone): δ = 163.9, 157.6, 155.5, 148.8, 140.8, 130.5, 126.5 (C_{Ar}-H), 124.8 (d, J = 10.1), 119.8 (d, J = 4.9, C_{Ar}-H), 79.9, 22.6.

^{19}F NMR (471 MHz, Acetone): δ = -122.7.

HRMS (ESI): calculated for $[M+H]^+$: 244.0616, found: 244.0616.

Synthesis of 2-chloro-3-isopropoxy-4-nitrobenzoic acid ***(17b)***

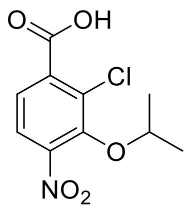

a) Modified from[10]: 2-Chloro-3-hydroxy-4-nitro-benzaldehyde **16b** (220 mg, 1.10 mmol, 1.0 Eq) was suspended in a *t*BuOH/THF mixture (4.5 mL each) and 2-Me-2-butene (1.2 mL, 10 Eq) was added. $NaClO_2$ (80%, 0.25 g, 2.0 Eq) solved in aqueous NaH_2PO_4-sol. (1 M, 2.0 mL) at 0°C was added dropwise in 3 portions after 0 min, 15 min and 1 h. 2-Me-2-butene (0.58 mL, 5 Eq) was added after 3 h and $NaClO_2$ (80%, 3x62 mg, 3x0.5 Eq) was added again in aqueous NaH_2PO_4-sol. (1 M, 3x0.50 mL) after 3 h, 3.5 h and 4 h. After 4 h 45 min and screening by LCMS the reaction mixture was concentrated u.r.p. to ~half volume, diluted between EA/HCl (0.5 M) (50 mL each), the pH was controlled to be ~1 and the aqueous phase was extracted with EA (2x30 mL). The organic phases were collected, dried over Na_2SO_4 and solvents were removed u.r.p. to get a yellow solid directly used in next reaction.

b) PPh_3 (0.63 g, 2.2 Eq) was solved in dry THF (3.5 mL) and DIAD (0.47 mL, 2.2 Eq) was added at 0°C. After precipitation *i*PrOH (0.21 mL, 2.5 Eq) was added and then the product from a) (238 mg, 1 Eq) was added dropwise as solution in dry THF (6.5 mL) at 0°C. The reaction was stirred at RT overnight and then LiOH*H_2O (0.46 g, 10 Eq) and water (10 mL) were added to the reaction solution. The mixture was heated to 60°C for 3.5 h under vigourous stirring. After 2.5 h the mixture was diluted between EA and NaOH (0.5 M) (50 mL each). The organic phase was extracted with NaOH (0.5 M, 2x20 mL) and organic phase was analysed for product residuals by TLC. The combined aqueous phases were washed with DCM (2x15 mL) and then acidified with HCl (2 M, 45 mL). The product was now extracted with EA (4x30 mL), the organic phases were dried over Na_2SO_4 and a colorless-orange solid was obtained (138 mg, 49% o2s) after solvent removal u.r.p..

^{1}H NMR (500 MHz, Acetone): δ = 7.88 (d, *J* = 8.5, 1H), 7.71 (d, *J* = 8.4, 1H), 4.62 (hept, *J* = 6.1, 1H), 1.31 (d, *J* = 6.1, 6H).

^{13}C NMR (126 MHz, Acetone): δ = 166.0, 148.4, 138.2, 133.1-129.5 (m), 125.9 (C_{Ar}-H), 123.7 (C_{Ar}-H), 79.9, 22.4.

HRMS (ESI): calculated for $[M+H]^+$: 260.0321, found: 260.0320.

Synthesis of 2-bromo-3-isopropoxy-4-nitrobenzoic acid ***(17c)***

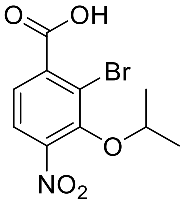

a) Modified from[10]: 2-Bromo-3-hydroxy-4-nitro-benzaldehyde **16c** (48.4 mg, 1.0 Eq) was solved in a THF/*t*BuOH-mixture (0.8 mL each) and 2-Me-2-butene (0.21 mL, 10 Eq) was added at 0°C to avoid evaporation. $NaClO_2$ (80%, 27 mg, 1.2 Eq) solved in aqueous NaH_2PO_4-solution (1 M, 0.20 mL) at 0°C was added and the reaction stirred for 2 h 45 min at RT. The reaction mixture was diluted between EA/HCl (0.1 M) (30 mL each), the pH was controlled to be ~1-2 and the aqueous phase was extracted with EA (2x10 mL). The organic phases were collected, dried over Na_2SO_4 and solvents were removed u.r.p. to get a yellow solid directly used in next reaction.

b) PPh_3 (119 mg, 2.3 Eq) was solved in dry THF (0.5 mL) and DIAD (89 µL, 2.3 Eq) was added at 0°C. After precipitation *i*PrOH (40µL, 2.6 Eq) was added and then the product from a) (51.5 mg, 1.0 Eq) was added as solution in dry THF (1.5 mL) at 0°C. The reaction was stirred at RT and *i*PrOH (8 µL, 0.5 Eq) was added again after 1.5 h. After 2 h LiOH*H_2O (87 mg, 10 Eq) and water (2.0 mL) were added to the reaction solution. The mixture was stirred vigourously for 30 min at RT, then at 60°C while being screened by TLC. After 4 h the reaction mixture was diluted between EA (30 mL) and brine/NaOH (1 M) (25 mL/ 5 mL). The organic phase was extracted with H_2O (1x15 mL), the combined aqueous phases were washed with EA (20 mL) and then acidified with HCl (1 M). The product was now extracted with EA (2x10 mL, 1x15 mL), the organic phases were dried over Na_2SO_4 and an off-colorless-solid was obtained (42.1 mg, 67% o2s) after solvent removal u.r.p. and by coevaporation with *n*-heptane.

^{1}H NMR (500 MHz, Acetone): δ = 7.92 (d, J = 8.4, 1H), 7.64 (d, J = 8.4, 1H), 4.59 (hept, J = 6.3, 1H), 1.31 (d, J = 6.1, 6H).

^{13}C NMR (126 MHz, Acetone): δ = 166.9, 149.2, 147.9, 141.2, 125.6, 124.6, 118.9, 80.0, 22.3.

HRMS (ESI): calculated for $[M+H]^+$: 303.9815/ 305.9795, found: 303.9818/ 305.9798.

Synthesis of methyl 2-amino-3-hydroxybenzoate ***(S5)***

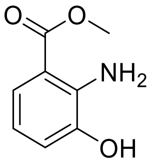

2-Hydroxy-3-amino benzoic acid **18** (1.12 g, 7.28 mmol, 1.00 Eq) was suspended in MeOH (50 mL) and H_2SO_4 (98%, 2.5 mL, 6.5 Eq) was added dropwise forming a clear dark brown solution. The mixture was refluxed overnight and after 24 h solvent volume was reduced to ~half volume by removing the refluxing device. Then NaOH (2 M) was added dropwise until neutralization occured. The solution was diluted between DCM (100 mL) and H_2O (100 mL) and the aqueous phase was extracted with DCM (3x50 mL). Combined organic phases were dried over Na_2SO_4 and solvents were removed u.r.p. to give 909 mg, 75% yield of a dark brown solid.

^{1}H NMR (500 MHz, $CDCl_3$): δ = 7.48 (dd, J = 8.2, 1.4, 1H), 6.82 (dd, J = 7.6, 1.4, 1H), 6.50 (t, J = 7.9, 1H), 5.4 (br s, 3H), 3.87 (s, 3H).

^{13}C NMR (126 MHz, $CDCl_3$): δ = 168.9, 143.2, 140.7, 123.5, 118.1, 115.2, 111.6, 51.8.

HRMS (ESI): calculated for $[M+H]^+$: 168.0656, found: 168.0657.

Synthesis of methyl 2-acetamido-3-hydroxybenzoate ***(S6)***

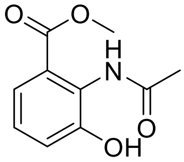

Crude methyl 2-amino-3-hydroxybenzoate **S5** (0.91 g, 5.4 mmol, 1.0 Eq) and acetic anhydride (0.54 mL, 1.05 Eq) were stirred in dry pyridine (15 mL) at RT for 26 h. The reaction was screened by LCMS (Note: first a mixture of *O*- or *N*-acetylated product is formed which slowly converts to the *N*-acetylated product). The reaction solution was diluted between DCM (50 mL) and HCl (2 M, 100 mL). The aqueous phase was extracted with DCM (3x30 mL) and organic phases were washed with H_2O (1x30 mL). Solvents were removed u.r.p. and a brown solid was obtained (1.12 g, 99%, NMR: 90%) that was used without further purification.

^{1}**H NMR** (500 MHz, $CDCl_3$): δ = 11.21 (s, 1H), 9.88 (s, 1H), 7.61 (dd, J = 7.8, 1.5, 1H), 7.24 (dd, J = 8.1, 1.5, 1H), 7.14 (t, J = 8.0, 1H), 3.93 (s, 3H), 2.34 (s, 4H).

HRMS (ESI): calculated for $[M+H]^+$: 210.0761, found: 210.0759.

Synthesis of methyl 2-acetamido-3-hydroxy-4-nitrobenzoate ***(19)***

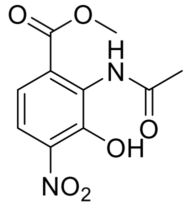

Methyl 2-acetamido-3-hydroxybenzoate **S6** (300 mg, 1.43 mmol, 1.00 Eq) was solved in dry acetone (14.3 mL) under Argon atmosphere. After stirring at 0°C for 10 min, HNO_3 (fuming, 0.48 mL, 8.0 Eq) was added dropwise. The reaction progress was then monitored carefully by LCMS. After 75 min HNO_3 (0.18 mL, 3.0 Eq) was added again and the reaction was stirred further for 45 min while being screened by LCMS.

The reaction was quenched by pouring the solution into sat. $NaHCO_3$ sol. (50 mL). The aqueous phase was extracted with DCM (3x40 mL or 1x50 mL) (organic phase 1). Then the pH of the aequos solution was adjusted to pH~5, changing its color from red to yellow and the solution was further extracted with DCM (3x40 mL) (organic phase 2). Organic phase 2 was dried over Na_2SO_4 and solvent was removed u.r.p.. To improve the yield organic phase 1 was reextracted with water and sat. $NaHCO_3$ sol. (40 mL each), followed by neutralization and extraction with DCM (2x30 mL). Dried over Na_2SO_4, this solution was combined with organic phase 2.

The reaction was repeated with same amounts of material but the second portion of HNO_3 (3.0 Eq) was already added after 15 min and the reaction was already quenched after 1 h. The workup was the same as shown above. The crude products from both reactions were combined and purified by FCC (solid loading, 80x reactant mass, PE/EA/AcOH, 65/30/5). After solvent removal u.r.p. and by coevaporation with heptane, the desired regioisomer was obtained as a yellow solid: 209 mg, 29%, NMR: 27%.

A mixed fraction was also collected, containing both regioisomers (238 mg).

^{1}H NMR (500 MHz, Acetone): δ = 10.97 (s, 1H), 10.03 (s, 1H), 7.85 (d, J = 8.7, 1H), 7.51 (d, J = 8.9, 1H), 3.90 (s, 3H), 2.26 (s, 3H).

^{13}C NMR (126 MHz, Acetone): δ = 171.6, 167.1, 147.1, 140.7, 130.1, 130.0, 121.5, 121.1, 53.1, 23.6.

HRMS (ESI): calculated for $[M+H]^+$: 255.0612, found: 255.0611.

Synthesis of methyl 2-acetamido-3-isopropoxy-4-nitrobenzoate ***(20)***

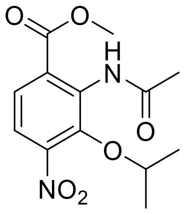

PPh_3 (56 mg, 2.0 Eq) was solved in dry THF (0.2 mL) under Ar-atmosphere and DIAD (42 µL, 2.0 Eq) was added at 0°C. After precipitation occured, *i*PrOH (16.4 µL, 2.0 Eq) was added and then a solution of methyl 2-acetamido-3-hydroxy-4-nitrobenzoate **19** (27 mg, 0.11 mmol, 1.0 Eq) in dry THF (0.6-0.8 mL) was added dropwise to the reaction. The reaction was stirred for 1.5 h and monitored by TLC, before solving the solution between brine and EA (15 mL each). The aqueous phase was extracted with EA (1x20 mL or 2x15 mL) and the combined organic phases were dried over Na_2SO_4. Solvents were removed u.r.p. to get a brown oil. This crude material was either used directly in next reaction or was purified by FCC (solid loading, 300x reactant mass, PE/acetone, 75/25) to give a brownish solid, yield 17.7 mg, 56%.

^{1}H NMR (700 MHz, $CDCl_3$): δ = 8.15 (s, 1H), 7.63 (d, J = 8.6, 1H), 7.59 (d, J = 8.6, 1H), 4.23 (hept, J = 6.2, 1H), 3.91 (s, 3H), 2.20 (s, 3H), 1.26 (d, J = 6.2, 6H).

^{13}C NMR (176 MHz, $CDCl_3$): δ = 168.6, 166.2, 146.1, 144.8, 132.7, 130.9, 124.9, 121.1, 79.6, 53.0, 23.8, 22.5.

HRMS (ESI): calculated for $[M+H]^+$: 297.1081, found: 297.1081.

Synthesis of 2-formamido-3-hydroxy-4-nitrobenzoic acid ***(22)***

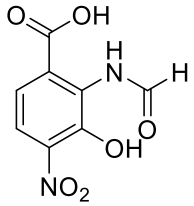

a) 2-amino-3-hydroxy benzoic acid **18** (1.00 g, 6.53 mmol, 1.00 Eq) was solved in HCOOH (pure, 20 mL) and Ac_2O (0.65 mL, 1.05 Eq) was added dropwise. After 1 h the precipitate was isolated, using pore size filter 4. Water (3x10 mL) was used for washing and the precipitate was collected mechanically and by solvation in EA (fraction A).

The washing solution was diluted between water (100 mL) and EA (50 mL) and the aqueous phase was extracted with EA (2x50 mL). The organic phases were combined, washed with brine (25 mL) and dried over Na_2SO_4 (fraction B). After LCMS analysis both fractions were combined and solvents were removed u.r.p. and by coevaporation with heptane to give a grey-reddish powder (1.13 g). The material was used without further purifications in the next step.

b) A part of the intermediate (0.80 g, 4.4 mmol, 1.0 Eq) was suspended in dry DCM (45 mL) under Argon atmosphere and cooled down to -25°C. Then HNO_3 (fum., 1.85 mL, 10.0 Eq) was added over 10 min resulting in a dark brown lumpy suspension. After 3 h the reaction mixture was quantitatively poured into water (50 mL, then 150 mL added portionwise) forming two phases. After extraction, the organic phase was collected and the aqueous phase (together with a precipitate between the two phases) was extracted with EA (4x40 mL). The combined organic phases were washed with brine, dried over Na_2SO_4 before adding toluene (100 mL) as a scavenger for nitronium. The orange brown-solution was concentrated u.r.p. to the toluene fraction (down to ~80 mbar, 40°C).

The residual suspension (~50 mL) was stored over 4 d in fridge forming a dark brown precipitate. The precipitate was isolated using pore size filter 4. Toluene (2x10 mL) was used for washing and the product was collected mechanically and by solvation in EA. The solvent was removed u.r.p. to give a yellow-brown fine solid (327 mg,

31% o2s). Note: A purity of 96% of this regioisomer was confirmed by LCMS while the filtrate contained almost exclusively a different regioisomer.

¹H NMR (500 MHz, DMSO): *δ* = 13.36 (br s, 1H), 10.85 (br s, 1H), 10.07 (s, 1H), 8.25 (s, 1H), 7.84 (d, *J* = 8.7, 1H), 7.41 – 7.23 (m, 1H).

¹³C NMR (126 MHz, DMSO): *δ* = 166.6, 160.7, 146.6, 138.9, 133.9, 126.1, 121.5, 119.6.

HRMS (ESI): calculated for $[M+H]^+$: 227.0299, found: 227.0299.

Synthesis of 2-amino-3-isopropoxy-4-nitrobenzoic acid ***(24)***

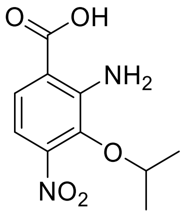

<u>Method 1:</u>

a) PPh_3 (230 mg, 1.1 Eq) was solved in dry THF (3 mL) under Argon atmosphere and DIAD (0.17 mL, 1.1 Eq) was added at 0°C. The reagent solution was stirred for 15 min at 0°C before adding methyl 2-acetamido-3-hydroxy-4-nitrobenzoate **19** (203 mg, 0.799 mmol, 1.00 Eq) as a solution in dry THF (5 mL) dropwise. After this, *i*PrOH (92 µL, 1.5 Eq) was added and the reaction was stirred for 3 h while being monitored with TLC.

b) The reaction mixture was concentrated u.r.p. to ~half volume, then KOH (4 M, 15 mL) was added and the mixture was stirred at 80°C before raising the temperature to 95°C after 1 h. After 2 h the heat source was switched off and the reaction mixture was stirred overnight. The next day the temperature was increased again to 95°C to ensure full conversion. After 2.5 h and reaction control by LCMS the reaction solution was extracted with EA (1x30 mL). The organic phase was extracted with NaOH (1 M, 2x20 mL) and the combined aqueous phases were washed with DCM (2x15 mL). After the pH was adjusted to ~7 using conc. HCl. the neutralized aqueous phase was extracted with EA (3x20 mL). Combining the organic phases was followed by drying over Na_2SO_4. Solvent removal u.r.p. and coevaporation with heptane (5x) resulted in a dark orange-brown solid (139 mg, 72% o2s).

Method 2:

a) PPh_3 (0.66 g, 2.1 Eq) was solved in dry THF (5.0 mL) under Argon atmosphere and DIAD (0.49 mL, 2.1 Eq) was added dropwise at 0°C forming a precipitate after few minutes. The mixture was stirred for 10 min and then *i*PrOH (0.23 mL, 2.5 Eq) was added. After 10 min 2-formamido-3-hydroxy-4-nitrobenzoic acid **22** (270 mg, 1.19 mmol, 1.00 Eq) was added dropwise as suspension in dry THF (7.0 mL) over 10 min. Then the mixture was stirred at RT for 1 h before it was diluted with EA. After adsorbtion on silica the material was purified by FCC (solid loading, 130x theoretical product mass, cyclohexane/EA 85/15->80/20->70/30) to give an off colorless solid that was dried under HV overnight and directly used in next step:

b) The crude material was suspended in a THF/water mixture (6 mL each) and $LiOH*H_2O$ (0.50 g, 10 Eq) was added. The reaction was then stirred at 60°C while being screened with LCMS. After 4 h water (6 mL) was added again and 2 h later the mixture was concentrated u.r.p. and the residue was diluted between DCM and H_2O (70 mL each). The aqueous phase was washed with DCM (1x30 mL), brine (5 mL) was added and washing was continued with EA (3x30 mL). The aqueous phase was then acidified to pH~5 by adding HCl (1 M) before it was extracted with EA (4x30 mL). The combined organic phases were dried over Na_2SO_4 before solvents were removed u.r.p. and by multiple coevaporation with heptane to give yellow-orange crystals (181 mg, 63% o2s). (Note: Reextraction of the former organic washing solution could not raise the yield significantly)

^{1}H NMR (500 MHz, $CDCl_3$): δ = 7.74 (d, J = 8.9, 1H), 6.96 (d, J = 9.0, 1H), 4.28 (hept, J = 6.1, 1H), 1.34 (d, J = 6.1, 6H).

^{13}C NMR (126 MHz, $CDCl_3$): δ = 171.0, 148.2, 146.7, 138.4, 127.0, 112.0, 110.2, 78.9, 22.7.

HRMS (ESI): calculated for $[M+H]^+$: 241.0819, found: 241.0820.

Synthesis of 2-isopropoxy-3-nitro-6-vinylpyridine ***(S7)***

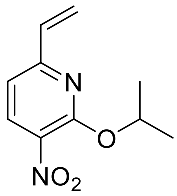

Potassium vinyltrifluoroborate (618 mg, 2.00 Eq), K_2CO_3 (415 mg, 1.30 Eq) and 6-chloro-2-isopropoxy-3-nitropyridine **26** (synthesized as described in[11], 500 mg, 2.31 mmol, 1.00 Eq) were subsequently added to a Schlenk flask. Dioxane (11 mL) and H_2O (3.0 mL) were added under Argon atmosphere and after a solution was formed, the mixture was freezed in liquid nitrogen. The flask was evacuated before refilling with Ar and warming up to RT. This procedure was repeated once again before $Pd(PPh_3)_4$ (67 mg, 0.025 Eq) was added. After one more freeze-degas cycle the mixture was stirred under Ar at 100°C for 17 h. The reaction mixture was diluted between H_2O/EA (50 mL each) and the aqueous phase was extracted with EA (2x30 mL). The combined organic phases were dried over Na_2SO_4 and solvents were removed u.r.p.. The crude product was purified by FCC (solid loading, 50x reactant mass, cyclohexane/EA, 95/5) to give a yellow oil (450 mg, 94%).

^{1}H NMR (500 MHz, $CDCl_3$): δ = 8.21 (d, J = 8.1, 1H), 6.89 (d, J = 8.1, 1H), 6.72 (dd, J = 17.1, 10.5, 1H), 6.37 (dd, J = 17.2, 1.4, 1H), 5.62 (dd, J = 10.5, 1.5, 1H), 5.60 – 5.52 (m, 1H), 1.44 (d, J = 6.3, 6H).

^{13}C NMR (126 MHz, $CDCl_3$): δ = 157.6, 155.7, 136.1, 135.1, 122.6, 114.0, 70.8, 22.0.

HRMS (ESI): calculated for $[M+H]^+$: 209.0921, found: 209.0921.

Synthesis of 6-isopropoxy-5-nitropicolinic acid ***(27)***

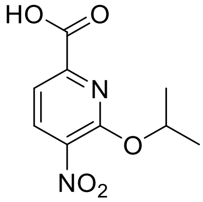

Modified from[11]: 2-isopropoxy-3-nitro-6-vinylpyridine **S7** (442 mg, 2.12 mmol, 1.00 Eq) was solved in acetone (7 mL) and a solution of $KMnO_4$ (1.34 g, 4.00 Eq) in

acetone/water (18 mL each) was added within 1 min. The reaction was stirred at RT for 20 h. The mixture was basified by mixing with NaOH (1 M, 2.1 mL) and the dark brown suspension was filtered through a pad of celite. The precipitate was washed with water and acetone and the pH of the yellow filtrate was adjusted to pH~4 by adding $NaHSO_4$-sol. (1 M). The solution was concentrated u.r.p. and the remaining aqueous solution was extracted with EA (4x20 mL). The combined organic phase was washed with brine (1x10 mL) before drying over Na_2SO_4 and removing solvents u.r.p.. An orange crystalline solid (413 mg, 86%) was obtained.

^{1}H NMR (500 MHz, DMSO): *δ* = 13.73 (s, 1H), 8.50 (d, *J* = 8.0, 1H), 7.75 (d, *J* = 8.0, 1H), 5.51 (hept, *J* = 6.2, 1H), 1.35 (d, *J* = 6.1, 6H).

^{13}C NMR (126 MHz, DMSO): *δ* = 164.4, 154.2, 149.2, 136.3, 136.1, 117.8, 71.0, 21.6.

HRMS (ESI): calculated for $[M+H]^+$: 227.0663, found: 227.0660.

Synthesis of methyl 3-hydroxy-2-methylbenzoate ***(S8)***

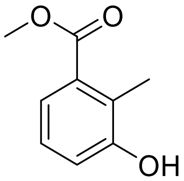

3-Hydroxy-2-methylbenzoic acid **28** (2.00 g, 13.1 mmol, 1.0 Eq) was dissolved in MeOH (6 mL) and $SOCl_2$ (1.63 mL, 22.3 mmol, 1.70 Eq) was added at 0°C. The solution was stirred at 80°C for 2 h. All volatiles were removed under reduced pressure to furnish the title compound in quantitative yield as colorless amorphous solid, which was used in the next step without further purification. The analytical data are consistent with those reported in the literature.[12]

R*f* (PE/EtOAc = 7:1) = 0.22

^{1}H NMR (400 MHz, $CDCl_3$): *δ* = 7.41-7.39 (dd, *J* = 1.0, 7.8 Hz, 1H, H_{Ar}), 7.11-7.07 (t, *J* = 7.9 Hz, 1H, H_{Ar}), 6.96-6.93 (dd, *J* = 0.8, 8.0 Hz, 1H, H_{Ar}), 4.39 (bs, 1H, OH), 3.89 (s, 3H, CO_2CH_3), 2.45 (s, 3H, CH_3).

*Synthesis of methyl 3-hydroxy-2-methylbenzoate (**S9**)*

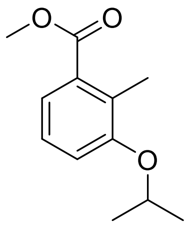

Methyl 3-hydroxy-2-methylbenzoate **S8** (2.18 g, 13.1 mmol, 1.0 Eq) was dissolved in DMF (10 mL). NaH (631 mg, 60% in mineral oil, 15.8 mmol, 1.20 Eq) was added in portions. The mixture was stirred at RT for 1 h. 2-Bromopropane (1.48 mL, 15.8 mmol, 1.20 Eq) was added and the mixture was stirred at RT for 20 h. The reaction was stopped with a 1 M HCl solution. The aq. phase was extracted with Et_2O (3x). The combined organic phases were washed with brine, dried over $MgSO_4$, filtered und concentrated. The crude product was purified by column chromatography (PE/EtOAc, 7:1) to furnish the product (1.66 g, 92 %) as colorless oil.

$\mathbf{R}_{\boldsymbol{f}}$ (PE/EtOAc = 7:1) = 0.66

^{1}H NMR (400 MHz, $CDCl_3$): δ = 7.39-7.37 (d, J = 7.9 Hz, 1H, H_{Ar}), 7.18-7.14 (t, J = 7.8 Hz, 1H, H_{Ar}), 7.00-6.99 (d, J = 8.1 Hz, 1H, H_{Ar}), 4.53-4.47 (sept, J = 6.0 Hz, 1H, $C\underline{H}(CH_3)_2$), 3.88 (s, 3H, CO_2CH_3), 2.41 (s, 3H, CH_3), 1.35-1.33 (d, J = 5.9 Hz, 6H, $CH(C\underline{H}_3)_2$)

^{13}C NMR (101 MHz, $CDCl_3$): δ = 168.8 (CO), 156.6 (C_{Ar}), 132.0 (C_{Ar}), 129.9 (C_{Ar}), 126.0 (C_{Ar}), 122.2 (C_{Ar}), 117.1 (C_{Ar}), 71.2 ($\underline{C}H(CH_3)_2$), 52.1 ($CO_2\underline{C}H_3$), 22.3 ($CH(\underline{C}H_3)_2$), 13.2 (CH_3).

*Synthesis of methyl 2-(bromomethyl)-3-isopropoxybenzoate (**29**)*

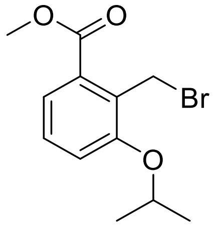

Methyl 3-hydroxy-2-methylbenzoate **S9** (1.63 g, 7.81 mmol, 1.0 Eq) was dissolved in CCl_4 (40 mL). NBS (2.08 g, 11.7 mmol, 1.50 Eq) and AIBN (0.27 g, 1.56 mmol, 0.2 Eq) were added. The mixture was stirred at 100°C for 16 h. The solvent was removed and the residue was diluted with EtOAc and H_2O. The aq. phase was extracted with EtOAc (3x). The combined organic phases were washed with brine, dried over $MgSO_4$, filtered and concentrated under reduced pressure. The crude product was purified by column

chromatography (PE/EtOAc = 10:1) to furnish the title compound (2.16 g, 7.54 mmol, 97%) as yellow amorphous solid.

R*f* (PE/EtOAc = 10:1) = 0.44;

^{1}H NMR (400 MHz, $CDCl_3$): *δ* = 7.49-7.46 (dd, *J* = 1.1, 7.8 Hz, 1H, H_{Ar}), 7.31-7.27 (t, *J* = 8.1 Hz, 1H, H_{Ar}), 7.07-7.05 (d, *J* = 8.0 Hz, 1H, H_{Ar}), 5.04 (s, 2H, CH_2Br), 4.68-4.59 (sept, *J* = 6.1 Hz, 1H, C<u>H</u>(CH_3)$_2$), 3.93 (s, 3H, CO_2CH_3), 1.41-1.39 (d, *J* = 6.0 Hz, 6H, CH(C<u>H</u>$_3$)$_2$)

^{13}C NMR (101 MHz, $CDCl_3$): *δ* = 167.5 (CO), 156.5 (C_{Ar}), 131.1 (C_{Ar}), 129.2 (C_{Ar}), 128.6 (C_{Ar}), 122.7 (C_{Ar}), 117.1 (C_{Ar}), 71.3 (<u>C</u>H(CH_3)$_2$), 52.5 (CO_2<u>C</u>H$_3$), 25.0 (CH_2Br), 22.2 (CH(<u>C</u>H$_3$)$_2$)

*Synthesis of methyl 2-formyl-3-isopropoxybenzoate (**S10**)*

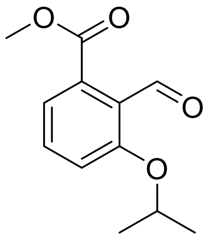

Methyl 2-(bromomethyl)-3-isopropoxybenzoate **29** (1.20 g, 4.19 mmol, 1.0 Eq) was dissolved in MeCN (36 mL) and NMO (1.96 g, 16.8 mmol, 4.00 Eq) was added. The solution was stirred at RT for 3 h. The solvent was removed under reduced pressure. The crude product was purified by column chromatography (PE/EtOAc = 10:1) to furnish the title compound (873 mg, 3.93 mmol, 94%) as yellowish amorphous solid (The synthesis was performed in collaboration with Norman Birke).

R*f* (PE/EtOAc = 10:1) = 0.26;

^{1}H NMR (400 MHz, $CDCl_3$): *δ* = 10.47 (s, 1H, CHO), 7.52-7.48 (t, *J* = 7.9 Hz, 1H, H_{Ar}), 7.10-7.07 (m, 2H, H_{Ar}), 4.70-4.64 (sept, *J* = 6.0 Hz, 1H, C<u>H</u>(CH_3)$_2$), 3.90 (s, 3H, CO_2CH_3), 1.39-1.38 (d, *J* = 6.1 Hz, 6H, CH(C<u>H</u>$_3$)$_2$)

^{13}C NMR (101 MHz, $CDCl_3$): *δ* = 190.4 (CHO), 169.4 (<u>C</u>O$_2$CH$_3$), 159.6 (C_{Ar}), 134.3 (C_{Ar}), 134.1 (C_{Ar}), 125.1 (C_{Ar}), 120.1 (C_{Ar}), 116.3 (C_{Ar}), 71.9 (<u>C</u>H(CH_3)$_2$), 52.9 (CO_2<u>C</u>H$_3$), 22.1 (CH(<u>C</u>H$_3$)$_2$)

*Synthesis of methyl 2-(difluoromethyl)-3-isopropoxybenzoate (**30**)*

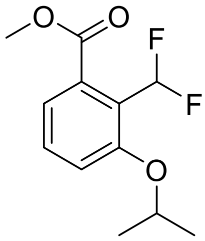

Methyl 2-formyl-3-isopropoxybenzoate **S10** (299 mg, 1.35 mmol, 1.0 Eq) was dissolved in DCM (3.3 mL) and DAST (533 µL, 4.04 mmol, 4.00 Eq) was added at 0°C. The mixture was stirred at RT for 20 h. A sat. $NaHCO_3$ solution was added at 0°C and the aq. phase was extracted with DCM (3x). The combined organic phases were washed with brine, dried over $MgSO_4$, filtered and concentrated under reduced pressure. The crude product was purified by column chromatography (PE/EtOAc = 15:1) to furnish the product (204 mg, 0.83 mmol, 92% brsm) as yellow oil.

R_f (PE/EtOAc = 10:1) = 0.34;

^{1}H NMR (400 MHz, $CDCl_3$): δ = 7.44-7.40 (t, J = 8.0 Hz, 1H, H_{Ar}), 7.31-7.03 (t, J = 54.5 Hz, 1H, CHF_2), 7.22-7.20 (d, J = 7.7 Hz, 1H, H_{Ar}), 7.09-7.07 (d, J = 8.4 Hz, 1H, H_{Ar}) 4.66-4.60 (sept, J = 6.0 Hz, 1H, C<u>H</u>(CH_3)$_2$), 3.91 (s, 3H, CO_2CH_3), 1.37-1.36 (d, J = 5.9 Hz, 6H, CH(C<u>H</u>$_3$)$_2$)

^{13}C NMR (101 MHz, $CDCl_3$): δ = 168.5 (CO), 156.8 (C_{Ar}), 133.0 (t, J = 3.5 Hz, C_{Ar}), 131.7 (t, J = 1.3 Hz, C_{Ar}), 122.0-121.6 (t, J = 21.9 Hz, C_{Ar}), 121.2 (t, J = 1.1 Hz, C_{Ar}), 116.7 (C_{Ar}), 113.8-109.1 (t, J = 237.3 Hz, CHF_2), 71.9 (<u>C</u>H(CH_3)$_2$), 52.8 (CO_2<u>C</u>H_3), 22.1 (CH(<u>C</u>H_3)$_2$)

^{19}F NMR (376 MHz, $CDCl_3$): δ = -114.67- -114.81 (d, J = 54.6 Hz, 2F, CHF_2)

HRMS (ESI): calculated for $[M+Na]^+$: 267.0809; found: 267.0811.

*Synthesis of 2-(difluoromethyl)-3-isopropoxybenzaldehyde (**31**)*

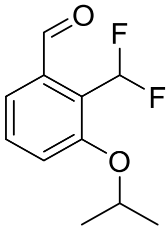

a) LAH (58.8 mg, 1.55 mmol, 2.00 Eq) was suspended in THF (1.1 mL) at 0°C. Methyl 2-(difluoromethyl)-3-isopropoxybenzoate **30** (189 mg, 0.78 mmol) in THF (1.5 mL) was added at 0°C and the mixture was stirred at RT for 4 h. H_2O was added at 0°C and the

suspension was filtered through a short Celite® plug. The plug was washed with EtOAc and the aq. phase was extracted with EtOAc. The combined organic phases were washed with HCl (1M), sat. $NaHCO_3$ solution, brine, dried over $MgSO_4$, filtered and concentrated under reduced pressure to give the title compound (141 mg, 84%) as colorless oil, which was used in the next step without further purification.

R*f* (PE/EtOAc = 5:1) = 0.46.

b) The material (141 mg, 0.65 mmol, 1.0 Eq) was dissolved in DCM (3.8 mL). Celite® (210 mg) and PCC (211 mg, 0.98 mmol, 1.50 Eq) were added subsequently. The mixture was stirred at RT for 1 h before it was poured onto a small silica plug. Elution with PE/EtOAc (7:3) furnished the title compound (135 mg, 96%) as a colorless oil.

R*f* (PE/EtOAc = 5:1) = 0.69

^{1}H NMR (400 MHz, $CDCl_3$): δ = 10.55-10.54 (t, J = 2.0 Hz, 1H, CHO), 7.64-7.62 (d, J = 7.7 Hz, 1H, H_{Ar}), 7.53-7.49 (t, J = 8.3 Hz, 1H, H_{Ar}), 7.50-7.23 (t, J = 54.5 Hz, 1H, CHF_2), 7.19-7.17 (d, J = 8.0 Hz, 1H, H_{Ar}), 4.67-4.58 (sept, J = 6.1 Hz, 1H, $C\underline{H}(CH_3)_2$), 1.39-1.38 (d, J = 6.0 Hz, 6H, $CH(C\underline{H}_3)_2$)

^{13}C NMR (101 MHz, $CDCl_3$): δ = 191.1 (t, J = 4.6 Hz, CHO), 156.3 (C_{Ar}), 136.6 (C_{Ar}), 132.2 (t, J = 1.4 Hz, C_{Ar}), 124.4 (C_{Ar}), 120.6 (t, J = 1.8 Hz, C_{Ar}) 119.1 (C_{Ar}), 114.3-109.6 (t, J = 235.3 Hz, CHF_2), 72.3 ($\underline{C}H(CH_3)_2$), 22.1 ($CH(\underline{C}H_3)_2$)

*Synthesis of 2-(difluoromethyl)-3-isopropoxy-4-nitrobenzaldehyde (**S11**)*

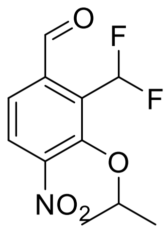

2-(Difluoromethyl)-3-isopropoxybenzaldehyde **31** (126 mg, 0.59 mmol, 1.0 Eq) in DCM (0.5 mL) was added dropwise to fuming HNO_3 (272 µL, 6.47 mmol, 11.0 Eq) at -40°C. The solution was stirred at -40°C for 2 h. The mixture was diluted with H_2O and the aq. phase was extracted with DCM (3x). The combined organic phases were washed with brine, dried over $MgSO_4$, filtered and concentrated under reduced pressure. The crude product was purified by column chromatography (PE/EtOAc = 10:1) to furnish the product (35.5 mg, 0.14 mmol, 23%) as brown oil.

R*f* (PE/EtOAc = 10:1) = 0.47;

^{1}H NMR (400 MHz, $CDCl_3$): *δ* = 10.57-10.56 (t, *J* = 1.9 Hz, 1H, CHO), 8.01-7.98 (d, *J* = 8.4 Hz, 1H, H_{Ar}), 7.93-7.91 (d, *J* = 8.5 Hz, 1H, H_{Ar}), 7.43-7.16 (t, *J* = 53.8 Hz, 1H, CHF_2), 4.31-4.22 (sept, *J* = 6.3 Hz, 1H, C<u>H</u>$(CH_3)_2$), 1.36-1.35 (d, *J* = 6.2 Hz, 6H, CH(C<u>H</u>$_3)_2$)

^{13}C NMR (101 MHz, $CDCl_3$): *δ* = 188.7 (t, *J* = 5.1 Hz, CHO), 149.8 (t, *J* = 7.3 Hz, C_{Ar}), 147.0 (C_{Ar}), 138.8 (C_{Ar}), 132.0-131.5 (t, *J* = 24.9 Hz, C_{Ar}), 128.0 (C_{Ar}), 124.0 (C_{Ar}), 113.6-108.9 (t, *J* = 237.8 Hz, CHF_2), 81.5 (<u>C</u>H$(CH_3)_2$), 22.3 (CH(<u>C</u>H$_3)_2$)

*Synthesis of 2-(difluoromethyl)-3-isopropoxy-4-nitrobenzoic acid (**32**)*

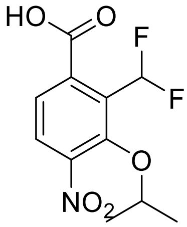

2-(Difluoromethyl)-3-isopropoxy-4-nitrobenzaldehyde **S11** (31.8 mg, 0.12 mmol, 1.0 Eq) and 2-methyl-2-butene (130 µL, 1.22 mmol, 10.0 Eq) were dissolved in *t*BuOH (0.5 mL). $NaClO_2$ (12.2 mg, 0.13 mmol, 1.10 Eq) in a NaH_2PO_4 solution (0.1 mL, 1M) was added dropwise at RT. The solution was stirred for 5 h, then HCl (1M) was added. The aq. layer was extracted with Et_2O (3x) and the combined organic layers were washed with brine, dried over $MgSO_4$ and filtered. The filtrate was concentrated under reduced pressure to give the crude product (30.4 mg, 0.11 mmol, 90%) as a yellow amorphous solid.

^{1}H NMR (400 MHz, $CDCl_3$): *δ* = 9.42 (bs, 1H, CO_2H), 7.94-7.92 (d, *J* = 8.4 Hz, 1H, H_{Ar}), 7.68-7.66 (d, *J* = 8.4 Hz, 1H, H_{Ar}), 7.35-7.08 (t, *J* = 53.8 Hz, 1H, CHF_2), 4.42-4.33 (sept, *J* = 6.3 Hz, 1H, C<u>H</u>$(CH_3)_2$), 1.35-1.33 (d, *J* = 6.1 Hz, 6H, CH(C<u>H</u>$_3)_2$)

^{13}C NMR (101 MHz, $CDCl_3$): *δ* = 170.6 (CO), 150.4 (C_{Ar}), 146.5 (C_{Ar}), 135.3 (C_{Ar}), 130.1-129.8 (t, *J* = 23.4 Hz, C_{Ar}), 127.4 (C_{Ar}), 125.2 (C_{Ar}), 112.6-107.8 (t, *J* = 240.3 Hz, CHF_2)), 81.2 (<u>C</u>H$(CH_3)_2$), 22.1 (CH(<u>C</u>H$_3)_2$)

HRMS (ESI): m/z calculated for $C_{11}H_{10}F_2NO_5$ $[M-H]^-$: 274.0527; found: 274.0529.

*Synthesis of tert-butyl 2-(allyloxy)-4-amino-3-methoxybenzoate (**34**)*

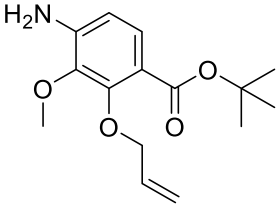

The compound was prepared from 2-(allyloxy)-3-methoxy-4-nitrobenzoic acid as modified from literature[13, 14] in a yield of 67%.

^{1}H NMR (400 MHz, DMSO): *δ* = 7.22 (d, J=8.6, 1H), 6.43 (d, J=8.6, 1H), 6.08 (ddt, J=17.3, 10.6, 5.3, 1H), 5.62 (s, 2H), 5.36 (dq, J=17.3, 1.8, 1H), 5.20 (dq, J=10.5, 1.5, 1H), 4.44 (dt, J=5.3, 1.6, 2H), 3.67 (s, 3H), 1.48 (s, 9H).

*Synthesis of methyl (3-(methoxymethoxy)phenyl)carbamate **(36)***[15, 16]

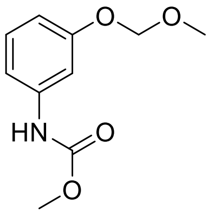

In an Ar flushed flask 3-aminophenol **35** (10.0 g, 91.6 mmol, 1.00 Eq) was solved in dry DMF (50 mL) and NaH (60% in mineral oil, 3.66 g, 1.00 Eq) was added in portions. After gas evolution calmed down, the mixture was stirred at 50°C for 30 min. The brownish suspension was stirred for 30 min before adding MOMCl (technical, 7.0 mL, 1.0 Eq) dropwise. The reaction was stirred further at 50°C. After 2 h, NaH (60% in mineral oil, 1.32 g, 0.60 Eq) was added again in portions before adding MOMCl (technical, 2.8 mL, 0.40 Eq) after 15 min dropwise.

The reaction mixture was diluted with water (200 mL). The aqueous reaction solution was extracted with EA (1x200 mL, 2x150 mL). The organic phase was dried over Na_2SO_4 and the solution was concentrated until ~100 mL.

Dry pyridine (15 mL, 2.0 Eq) was added to the product solution and then Methyl chloroformiate (7.8 mL, 1.1 Eq) was added dropwise over 15 min. The reaction was stirred for 15 min at RT before solving between EA (100 mL) and H_2O (100 mL) and extracting the aqueous phase with EA (1x50 mL). The organic phase was washed with HCl (1 M, 200 mL). The combined aqueous phases were extracted again with EA (2x50 mL) after pH check. The combined organic phases were washed with brine

(1x30 mL) before removing solvent u.r.p. over silica. The product was purified by filtration over silica (PE/acetone, 100/0->90/10->85/15) to obtain a clear oil (10.8 g, 56% o2s).

^{1}H NMR (500 MHz, $CDCl_3$): δ = 7.20 (t, J = 8.2, 1H, H-Ar), 7.17 (s, 1H), 6.99 (d, J = 7.8, 1H, H-Ar), 6.74 (ddd, J = 8.2, 2.2, 0.9, 1H, H-Ar), 6.64 (s, 1H), 5.17 (s, 2H, O-CH_2-O), 3.77 (s, 3H, CH_3), 3.47 (s, 3H, CH_3).

^{13}C NMR (126 MHz, $CDCl_3$): δ = 158.0 (NH-COO), 154.0 (C_{Ar}), 139.2 (C_{Ar}), 123.0 (C_{Ar}), 112.3 (C_{Ar}), 111.4 (C_{Ar}), 106.9 (C_{Ar}), 94.6 (O-CH_2-O), 56.2 (CH_3), 52.5 (CH_3).

HRMS (ESI): calculated for $[M+H]^+$: 212.0918, found: 212.0918.

Synthesis of methyl (3-(methoxymethoxy)-2-(2-methylallyl)phenyl)carbamate ***(S12)***

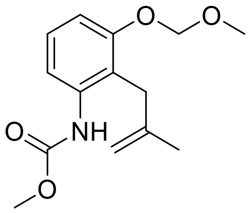

Preparation of cuprate solution: LiCl (4.16 g, 98.1 mmol) was heated extensively u.r.p. while gassing and degassing with Ar. CuCN (4.40 g, 49.1 mmol) and dry THF (48 mL) were added under inert conditions and the green mixture was stirred until salts were solved.

Modified from[16, 17]: Under strict Ar-atmosphere: **36** (4.00 g, 18.9 mmol, 1.00 Eq) and a stirring bar were added to a Schlenk flask and dried u.r.p. before gassing with Ar. Then dry THF (100 mL) was added and the mixture was cooled to -78°C by cryostate. *t*BuLi (1.6 M in pentane, 24.9 mL, 2.10 Eq) was added dropwise over 20 min, resulting in a yellow solution. The reaction was stirred for 15 min before raising temperature to -20°C over 70 min. A suspension formed which turned to a solution again above -30°C. The mixture was stirred for additional 1 h at -20°C, then cooled down again to -78°C. The mentioned solution of CuCN*2 LiCl in THF (21 mL, 1.1 Eq) was added dropwise over 20 min. After 40 min further stirring, 3-bromo-2-methylpropene (0.48 mL, 1.0 Eq) was added dropwise and the mixture was slowly warmed to -20°C over 80 min.

The reaction was quenched by adding ammonia (13% in H_2O, 130 mL) and stirring the solution vigourously for 20 min. The blueish suspension was concentrated u.r.p. and

was then extracted with EA (3x70 mL). The combined organic phases were washed with brine (1x20 mL) and dried over Na_2SO_4 before removing solvent u.r.p.. The purification by FCC (solid loading, 30x reactant mass, PE/EA, 93/7->90/10->85/15) led to a slightly yellowish transparent oil after solvent removal (2.43 g, 48%).

R_f(PE/EA, 70/30)=0.55

^{1}H NMR (700 MHz, $CDCl_3$): δ = 7.52 (s, 1H), 7.19 (t, J = 8.3, 1H, H-Ar), 6.88 (d, J = 8.3, 1H, H-Ar), 6.75 (s, 1H), 5.17 (s, 2H, O-CH_2-O), 4.85 (s, 1H, CH_2=C), 4.65 (s, 1H, CH_2=C), 3.75 (s, 3H, CH_3), 3.45 (s, 3H, CH_3), 3.42 (s, 2H, CH_2-Ar), 1.76 (s, 3H, C-CH_3).

^{13}C NMR (126 MHz, $CDCl_3$): δ = 155.4 (C_{Ar}), 154.5 (NH-COO), 144.0 ($C_2\underline{C}$=C), 137.9 (C_{Ar}), 127.7 (HC_{Ar}-$\underline{C}_{Ar}$-$C_{Ar}H$), 118.3* (C_{Ar}), 115.3* (C_{Ar}), 111.4 (CH_2=C), 110.1 (C_{Ar}), 94.8 (O-CH_2-O), 56.2 (CH_2-O-$\underline{C}H_3$), 52.4 (COO-$\underline{C}H_3$), 32.9 (CH_2-Ar), 22.4 (C-$\underline{C}H_3$).

*assignment proved by HSQC

HRMS (ESI): calculated for $[M+H]^+$: 266.1387, found: 266.1387.

Synthesis of methyl (3-(methoxymethoxy)-2-isobutylphenyl)carbamate ***(S13)***

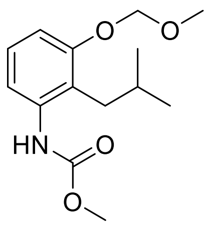

Compound **S12** (2.42 g, 9.12 mmol, 1.00 Eq) was solved in MeOH (90 mL) and Pd/C (0.49 g, 0.05 Eq) was added. The flask was filled with H_2 by multiple degassing and gassing with a balloon and the reaction was stirred vigourously. After 3.5 h the black suspension was filtrated over celite and a pore size 4 filter. The solvent was removed u.r.p. and by coevaporation with heptane to give a transparent oil (2.37 g, 97%).

^{1}H NMR (700 MHz, $CDCl_3$): δ = 7.46 (s, 1H), 7.14 (t, J = 8.2, 1H, H-Ar), 6.88 (d, J = 8.3, 1H), 6.43 (s, 1H), 5.17 (s, 2H, O-CH_2-O), 3.77 (s, 3H, O-CH_3), 3.47 (s, 3H, O-CH_3), 2.51 (d, J = 7.4, 2H, CH_2-Ar), 1.91 – 1.80 (m, 1H, $C\underline{H}(CH_3)_2$), 0.94 (d, J = 6.7, 6H, $CH(C\underline{H}_3)_2$).

^{13}C NMR (126 MHz, $CDCl_3$): δ = 155.9 (C_{Ar}-O), 154.6 (NH-COO), 136.7 (C_{Ar}), 127.1 (C_{Ar}), 115.6 (C_{Ar}), 110.0 (C_{Ar}), 94.7 (O-CH_2-O), 56.2 (CH_2-O-$\underline{C}H_3$), 52.5 (COO-$\underline{C}H_3$), 33.5 (CH_2-Ar), 28.9 ($\underline{C}(CH_3)_2$), 22.8 ($C(\underline{C}H_3)_2$).

HRMS (ESI): calculated for $[M+H]^+$: 268.1544, found: 268.1543.

Synthesis of methyl (3-hydroxy-2-isobutylphenyl)carbamate ***(37)***

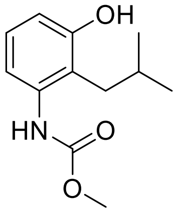

Compound **S13** (0.70 g, 2.6 mmol, 1.0 Eq) was solved in MeOH (13 mL) and TMSCl (1.7 mL, 5.0 Eq) was added dropwise. The reaction was stirred at RT for 2 h before removing the reaction solvent u.r.p. and coevaporate with heptane (3x) to give a brownish gum becoming slowly a fluffy solid (0.57 g, 97%).

^{1}H NMR (500 MHz, $CDCl_3$): δ = 7.37 (s, 1H), 7.05 (t, J = 8.1, 1H, H-Ar), 6.55 (d, J = 8.0, 1H, H-Ar), 6.43 (s, 1H), 5.01 (br s, 1H, OH), 3.77 (s, 3H, CH_3), 2.47 (d, J = 7.5, 2H, CH_2-Ar), 1.93 – 1.82 (m, 1H, $C\underline{H}(CH_3)_2$), 0.95 (d, J = 6.6, 6H, $CH(C\underline{H}_3)_2$).

^{13}C NMR (126 MHz, $CDCl_3$): δ = 154.7 (NH-COO), 154.2 (C_{Ar}-O), 136.8 (C_{Ar}), 127.1 (C_{Ar}), 114.70 (C_{Ar}), 111.60 (C_{Ar}), 52.6 (COO-$\underline{C}H_3$), 33.4 (CH_2-Ar), 28.8 ($\underline{C}(CH_3)_2$), 22.7 ($C(\underline{C}H_3)_2$).

HRMS (ESI): calculated for $[M+H]^+$: 224.1281, found: 224.1282.

Synthesis of 3-amino-2-isobutylphenol ***(S14)***

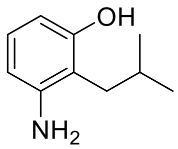

Compound **37** (224 mg, 1.00 mmol, 1.00 Eq) was solved in KOH (4 M, 10 mL) and refluxed for 3 h. The reaction solution was poured into NaH_2PO_4-solution (1 M, 50 mL) and the aqueous phase was extracted with EA (2x30 mL). Solvents were removed u.r.p. to give a brown crystalline solid (158 mg, 95%).

^{1}H NMR (700 MHz, DMSO-d_6): δ = 8.64 (s, 1H, OH), 6.63 (t, J = 7.9, 1H, H-Ar), 6.08 (dd, J = 7.9, 1.2, 1H, H-Ar), 6.03 (dd, J = 7.9, 1.2, 1H, H-Ar), 4.56 (s, 2H, NH_2), 2.29 (d, J = 7.4, 2H, CH_2-Ar), 1.88 – 1.82 (m, 1H, $C\underline{H}(CH_3)_2$), 0.85 (d, J = 6.6, 6H, $CH(C\underline{H}_3)_2$).

^{13}C NMR (176 MHz, DMSO-d_6) δ = 155.9 (C_{Ar}-O), 147.5 (C_{Ar}-N), 126.1 (C_{Ar}), 111.45 (C_{Ar}), 106.3 (C_{Ar}), 103.5 (C_{Ar}), 32.3 (CH_2-Ar), 27.1 ($\underline{C}(CH_3)_2$), 22.6 ($C(\underline{C}H_3)_2$).

HRMS (ESI): calculated for $[M+H]^+$: 166.1227, found: 166.1229.

Synthesis of tert-butyl (3-hydroxy-2-isobutylphenyl)carbamate ***(S15)***

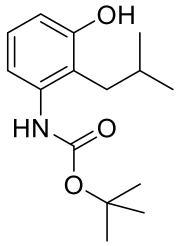

The previous product **S14** (154 mg, 0.932 mmol, 1.00 Eq) was solved in dry EtOH (3.5 mL) and Boc_2O (0.32 mL, 1.5 Eq) was added dropwise over 5 min before the mixture was refluxed. The reaction solution was concentrated u.r.p. after 2 h 15 min until crystals were formed. Then the rest of Boc_2O was removed by coevaporation with EA/heptane to give a brownish solid (252 mg, >98%, NMR corrected yield: 95%).

^{1}H NMR (700 MHz, DMSO-d_6): δ = 9.17 (s, 1H, NH-COO), 8.27 (s, 1H, OH), 6.90 (t, J = 8.0, 1H, H-Ar), 6.68 (dd, J = 8.0, 1.1, 1H, H-Ar), 6.60 (dd, J = 8.0, 1.2, 1H, H-Ar), 2.43 (d, J = 7.2, 2H, CH_2-Ar), 1.82 – 1.74 (m, 1H, $C\underline{H}(CH_3)_2$), 1.42 (s, 9H, $C(CH_3)_3$), 0.81 (d, J = 6.7, 6H, $CH(C\underline{H}_3)_2$).

^{13}C NMR (176 MHz, DMSO-d_6) δ = 155.8 (C_{Ar}-O), 154.0 (NH-COO), 137.5 (C_{Ar}-N), 125.7 (C_{Ar}), 123.3 (C_{Ar}), 117.4 (C_{Ar}), 111.8 (C_{Ar}), 78.2 ($\underline{C}(CH_3)_3$), 32.9 (CH_2-Ar), 28.2 ($C(\underline{C}H_3)_2$), 26.9 ($(CH_3)_2\underline{C}H$), 22.5 ($(\underline{C}H_3)_2CH$).

HRMS (ESI): calculated for $[M+H]^+$: 266.1751, found: 266.1751.

Synthesis of tert-butyl (4-formyl-3-hydroxy-2-isobutylphenyl)carbamate ***(38)***

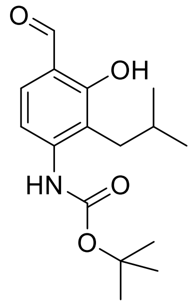

A flask equipped with **S15** (179 mg, 0.675 mmol, 1.00 Eq) and heat gun dried $MgCl_2$ (129 mg, 2.00 Eq) was flushed with Ar and the content was suspended in dry MeCN (3.4 mL). Then dry TEA (0.37 mL, 4.0 Eq), DMPU (0.16 mL, 2.0 Eq) and paraformaldehyde (162 mg, 8.0 Eq) were added subsequentially. The mixture was stirred at 80°C getting yellow after few minutes. After 2 h the mixture was diluted between HCl (0.1 M, 30 mL) and EA (30 mL) and the aqueous phase was extracted with EA (2x20 mL). The combined organic phases were washed with brine (1x10 mL) and dried over Na_2SO_4 before the product was isolated by FCC (solid loading, 85x reactant mass, PE/EA, 95/5) to obtain colorless crystals (86 mg, 43%).

^{1}H NMR (700 MHz, $CDCl_3$): δ = 11.57* (s, 1H, OH), 9.72* (s, 1H, CHO), 7.78* (d, J = 8.7, 1H, $\underline{H}$-C_{Ar}-C_{Ar}-N), 7.37* (d, J = 8.7, 1H, $\underline{H}$-C_{Ar}-C_{Ar}-CHO), 6.68 (s, 1H, NH-COO), 2.49 (d, J = 7.5, 2H, CH_2-Ar), 1.93 – 1.87* (m, 1H, $C\underline{H}(CH_3)_2$), 1.53* (s, 9H, $C(CH_3)_2$), 0.96* (d, J = 6.8, 6H, $(C\underline{H}_3)_2CH$).

^{13}C NMR (176 MHz, $CDCl_3$): δ = 195.3* (COH), 161.0* (C_{Ar}-O), 152.2* (NH-COO), 144.4* (C_{Ar}-N), 132.7* ($\underline{C}_{Ar}$-C_{Ar}-COH), 116.4 (C_{Ar}), 116.2 (C_{Ar}), 110.9* ($\underline{C}_{Ar}$-C_{Ar}-N), 81.7 ($\underline{C}(CH_3)_3$), 32.0* (CH_2-Ar), 28.3* ($C(\underline{C}H_3)_3$), 28.2* ($(CH_3)_2\underline{C}H$), 22.7 ($(\underline{C}H_3)_2CH$).

*assignment proved by HSQC/HMBC

HRMS (ESI): calculated for $[M+H]^+$: 294.1700, found: 294.1701.

*Synthesis of 2-(allyloxy)-4-((tert-butoxycarbonyl)amino)-3-isobutylbenzoic acid **(39)***

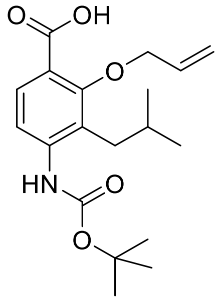

Modified from[10]:

a) Cmpd. **38** (85 mg, 0.29 mmol, 1.0 Eq) and K_2CO_3 (0.16 g, 4.0 Eq) were secured under Ar and dry DMF (1.5 mL) was added. Then allyl bromide (30 µL, 1.2 Eq) was added and the reaction was stirred at RT for 2 h. The mixture was diluted between EA/H_2O (25 mL each) and the aqueous phase was extracted with EA (2x20 mL). The combined organic phases were dried over Na_2SO_4 before removing solvent u.r.p. to give a yellow oil (yield: 96 mg, 99%) directly used in the next step.

b) A part of the material from a) (86 mg, 0.26 mmol, 1.0 Eq) was solved in MeCN (1.3 mL) and aqueous NaH_2PO_4-sol. (0.5 M, 0.52 mL) and H_2O_2 (30%, 79 µL) were added. $NaClO_2$ (80%, as 1 M solution in NaH_2PO_4-buffer (1 M), 0.36 mL, 1.4 Eq) was added and the reaction was stirred at RT. After 45 min $NaClO_2$-solution (0.16 mL, 0.60 Eq) was added again and after 1 h Na_2SO_3-sol. (1 M, 5 mL) and NaH_2PO_4-sol. (1 M, 15 mL) were added. The mixture was extracted with EA (3x20 mL) and after drying the combined organic phases over Na_2SO_4 solvents were removed u.r.p.. A colorless crystalline solid (yield: 76 mg, 84% o2s) was obtained.

^{1}H NMR (700 MHz, $CDCl_3$): δ = 7.97 (d, J = 8.8, 1H, C_{Ar}-H), 7.91 (d, J = 8.9, 1H, C_{Ar}-H), 6.54 (s, 1H, N-H), 6.14 – 6.04 (m, 1H), 5.48 (dq, J = 17.2, 1.6, 1H), 5.37 (dq, J = 10.4, 1.2, 1H), 4.42 (d, J = 5.6, 2H), 2.48 (d, J = 7.4, 2H), 1.92 – 1.83 (m, 1H, -C<u>H</u>(CH_3)$_2$), 1.53 (s, 9H), 0.95 (d, J = 6.6, 6H, -CH(C<u>H</u>$_3$)$_2$).

^{13}C NMR (176 MHz, $CDCl_3$): δ = 166.5, 157.4, 152.5, 142.8, 132.1, 131.6, 123.9, 119.6, 117.2, 117.1, 81.7, 77.1, 33.7, 28.9, 28.4, 22.8.

HRMS (ESI): calculated for $[M+H]^+$: 350.1962, found: 350.1962.

*Synthesis of dimethyl 2,3-dihydroxyterephthalate (**S16**)*

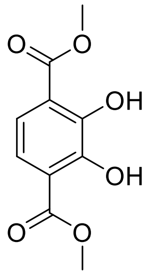

2,3-dihydroxyterephthalic acid **56** (1.0 g, 5.05 mmol, 1 Eq) was added to a dry vial and further dried at high vacuum. Dry methanol (20 mL) was added under argon atmosphere and the solution was cooled down to 0°C. Chlorotrimethylsilane (2 mL, 3.1 Eq) was slowly added under argon atmosphere at 0°C. The reaction was stirred overnight and controlled over LCMS. After completion, the solvent was removed under reduced pressure. The crude product was purified by chromatography. White needles were obtained, yield: 887 mg (78 %).

^{1}H NMR (500 MHz, $CDCl_3$): δ = 10.92 (br s, 2H), 7.33 (br s, 2H), 3.98 (s, 6H).

^{13}C NMR (126 MHz, $CDCl_3$): δ = 170.3, 151.7, 118.5, 116.0, 52.9.

*Synthesis of 2-hydroxy-3-isopropoxy-4-(methoxycarbonyl)benzoic acid (**57**)*

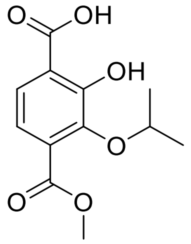

a) Dimethyl 2,3-dihydroxyterephthalate **S16** (1.00 g, 4.42 mmol, 1.0 Eq) and triphenylphosphine (1.27 g, 4.84 mmol, 1.1 Eq) were added to a dry flask and further dried under high vacuum. Dry THF (10 mL) and dry *i*PrOH (0.68 mL, 8.89 mmol, 2.0 Eq) were added under argon atmosphere. The mixture was stirred until all reactants/ reagents dissolved. Half of the DIAD (in total: 0.95 mL, 4.82 mmol, 1.1 Eq) was added dropwise under argon atmosphere before the solution was cooled down to 0 °C and the remaining DIAD was added dropwise under argon atmosphere. The reaction was slowly warmed up to RT and stirred overnight. After completion, the reaction was concentrated under reduced pressure. The crude product was purified by chromatography.

b) Nicotinic acid (2.28 g, 18.5 mmol, 5 Eq) and potassium carbonate (510 mg, 3.7 mmol, 1.0 Eq) were added to a flask. Dry DMF (4 mL) was added and the mixture was stirred at 110 °C for 30 min. The crude dimethyl 2-hydroxy-3-isopropoxyterephthalate (3.7 mmol, 1 Eq) was dissolved in dry DMF (7 mL) and added to the mixture. The reaction was stirred for 48 h at 110°C and controlled over LCMS. After completion, HCl (1 M, 30 mL) and brine (100 mL) were added and extracted with EtOAc (3x40 mL). The crude product was purified by chromatography. A white to off-white solid was obtained, yield: 509.6 mg (45 % over 2 steps).

^{1}H NMR (500 MHz, DMSO): *δ* = 11.73 (br s, 1H), 7.56 (d, 1H, J = 8.3 Hz), 7.06 (d, 1H, J = 8.3 Hz), 4.53 (hept., 1H, J = 6.2 Hz), 3.82 (s, 3H), 1.19 (d, 6H, J = 6.2 Hz)

^{13}C NMR (126 MHz, DMSO): *δ* = 171.9, 166.3, 155.6, 144.4, 131.7, 124.2, 118.4, 116.1, 75.2, 52.3, 22.2

*Synthesis of 2-bromo-6-isopropoxyphenol (**61**)*

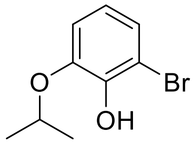

Dry *tert*-butylamine (2.8 mL, 26.6 mmol, 2.0 Eq) and dry toluene (27 mL) were added to a dry flask under nitrogen atmosphere. The mixture was cooled down to -30°C. Bromine (0.68 ml, 13.2 mmol, 1.0 Eq) was slowly added under nitrogen atmosphere and the reaction mixture was stirred for 30 min at -30°C. Afterwards the reaction was cooled down to -78°C. 2-isopropoxyphenol **60** (1.95 ml, 13.2 mmol, 1.0 Eq) dissolved in dry DCM (2 mL) was slowly added. The reaction was allowed to warm up to RT over 5 h. After completion, water (40 mL) and diethyl ether (10 ml) were added. HCl (5 mL, 1M) was added and the organic phase was separated. The aqueous phase was extracted with diethyl ether (3x10 mL). The organic phase was washed with HCl (15 mL, 1M) and saturated Na_2SO_3 solution. The solvent was removed under reduced pressure. The product was isolated by vacuum distillation (bp: 113°C at 14.7 mbar). The product was a clear colorless oil, yield: 1.90 mg, 62 %.

^{1}H NMR (500 MHz, $CDCl_3$): *δ* = 7.08 (dd, 1H, *J* = 1.4 Hz, 8.2 Hz), 6.82 – 6.80 (m, 1H), 6.71 (t, 1H, *J* = 8.2 Hz), 6.01 (s, 1H), 4.58 (hept., 1H, *J* = 6.1 Hz), 1.37 (d, 6H, *J* = 6.1 Hz)

^{13}C NMR (126 MHz, $CDCl_3$): *δ* = 145.5, 144.4, 125.0, 120.6, 112.6, 108.4, 72.5, 22.3

HRMS (ESI): calculated for $[M-H]^+$: 228.9864/230.9844, found: 228.9870/230.9850.

Synthesis of 6-bromo-2-isopropoxy-3-nitrophenyl acetate ***(62)***[18]

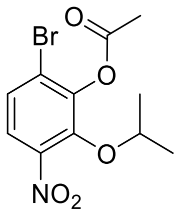

a) 2-bromo-6-isopropoxyphenol **61** (2.20 g, 9.5 mmol, 1.00 Eq) was added to a nitrogen-flushed flask and dry pyridine (4.5 mL, 6.0 Eq) and Ac_2O (1.8 mL, 2.0 Eq) were added at 0°C. The reaction was stirred for 2 h at RT. HCl(aq) (1 M, 80 mL) and brine (120 mL) were added, the aequeous phase was extracted with Et_2O (3x50 mL) then the organic phase was washed with brine (2x8 mL). Solvent was removed under reduced pressure to give a colorless oil, 2.55 g (97%). The product was directly used in the next step.

b) The intermediate (747 mg, 2.73 mmol, 1.00 Eq) was solved in DCM (4.5 mL), the solution was added dropwise to fuming HNO_3 (1.5 mL, 13 Eq) at -40°C (cooled with MeCN/dry ice mixture). After full reactant conversion the reaction mixture was quenched with 25 mL water and the aqueous phase was extracted with DCM (3x20 mL). The product was adsorbed on celite and purified by FCC (PE/EA, 90/10, silica particle size: 63-200 µm). Product was obtained as yellow crystals, yield: 693 mg, 80%.

^{1}H NMR (500 MHz, $CDCl_3$): *δ* = 7.63 (d, 1H, *J* = 9.0, Ar-H), 7.43 (d, 1H, *J* = 8.9, Ar-H), 4.44 (hept, 1H, *J* = 6.1, C-H), 2.39 (s, 3H, CO-CH_3), 1.31 (d, 6H, *J* = 6.3, CH(C$\underline{H}_3$)$_2$).

^{13}C NMR (126 MHz, $CDCl_3$): 166.9, 145.7, 144.5, 144.4, 127.1, 123.4, 123.1, 79.7, 22.6, 20.6.

Synthesis of 2-isopropoxy-3-nitro-6-((trimethylsilyl)ethynyl)phenyl acetate ***(63)***

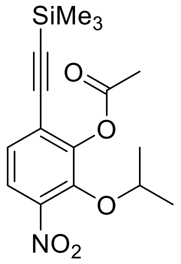

Modified from[19]: 6-bromo-2-isopropoxy-3-nitrophenyl acetate **62** (516 mg, 1.62 mmol, 1.00 Eq), CuI (31 mg, 0.10 Eq) and $PdCl_2(PPh)_3$ (57 mg, 0.05 Eq) were added to a flask which subsequently was flushed with nitrogen (3x). Dry DMF (2 mL) and DiPEA (1.4 mL, 5.0 Eq) were added and Ethynyl-TMS (0.48 g, 3.0 Eq) was introduced as a solution in dry DMF (2 mL). The mixture turned its color to brownish and was stirred overnight at 35°C. The reaction mixture was diluted with DCM (30 mL) and organic phase was washed with HCl (1 M, 2x30 mL). The aqueous phase was reextracted with DCM (2x 25 mL). The solvent was removed u.r.p. from the combined organic phases. The crude product was absorbed on Celite and purified by FCC (PE/EA, 90/10, silica particle size: 63-200 µm) (note: purification is necessary for the next reaction step) to give 445 mg, 82% of a brown solid.

^{1}H NMR (500 MHz, $CDCl_3$): δ = 7.65 (d, J = 8.5, 1H), 7.27 (d, J = 8.5, 1H), 4.42 (hept, J = 6.2, 1H), 2.36 (s, 3H), 1.30 (d, J = 6.1, 6H), 0.25 (s, 9H).

^{13}C NMR (126 MHz, $CDCl_3$): δ = 167.2, 147.3, 144.9, 144.8, 126.8, 124.1, 122.3, 104.7, 97.8, 79.3, 22.6, 20.7, -0.2.

HRMS (ESI): calculated for $[M+H]^+$: 336.1262, found: 336.1259.

Ring E analogues

*Synthesis of tert-butyl 4-(methylamino)benzoate (**S17**)*

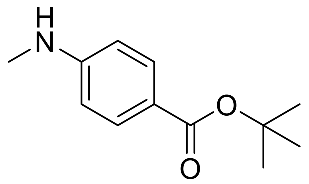

4-Methylaminobenzoic acid **82** (0.50 g, 3.31 mmol, 1.0 Eq) was dissolved in *t*BuOH (17 mL). EDC*HCl (2.22 g, 11.6 mmol, 3.50 Eq) and DMAP (2.02 g, 16.5 mmol, 5.00 Eq) were added and the solution was stirred at RT for 20 h. The solvent was removed under reduced pressure. The residue was purified by column chromatography (dry load, PE/EtOAc = 6:1) to furnish the title compound (0.34 g, 1.65 mmol, 50%) as colorless oil.

The analytical data are consistent with those reported in the literature.[20]

R*f* (PE/EtOAc = 6:1) = 0.40;

^{1}H NMR (400 MHz, $CDCl_3$): *δ* = 7.84-7.82 (d, *J* = 8.8 Hz, 2H, H_{Ar}), 6.57-6.55 (d, *J* = 8.8 Hz, 2H, H_{Ar}), 4.50 (bs, 1H, NH), 2.88 (s, 3H, CH_3), 1.57 (s, 9H, $C(CH_3)_3$)

*Synthesis of methyl 4-amino-3-formylbenzoate (**S18**)*

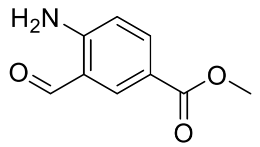

Methyl 3-formyl-4-nitrobenzoate **76** (300 mg, 1.4 mmol, 1.0 Eq) was dissolved in THF (6.2 mL) and EtOH (5.0 ml) and cooled down to 0°C. Zinc dust (1.38 g, 15 Eq) was added. Acetic acid (1.15 mL, 20.1 mmol, 14.0 Eq) was added to the stirring mixture over 30 min and the mixture was allowed to reach room temperature. After completion, the zinc dust was filtered off. Saturated $NaHCO_3$ solution (45 mL) was added and the aqueous phase was extracted with EtOAc (3x25 mL). The combined organic phases were removed under reduced pressure and the crude product was purified by chromatography (PE/ EtOAc). The product was a yellow solid, yield: 156.1 mg, 61 %.

^{1}H NMR (700 MHz, MeOH): *δ* = 9.85 (s, 1H), 8.21 (d, 1H, *J* = 2.1 Hz), 7.86 (dd, 1H, *J* = 2.1 Hz, 8.8 Hz), 6.76 (d, 1H, *J* = 8.8 Hz), 3.86 (s, 3H)

^{13}C NMR (176 MHz, MeOH): *δ* = 195.1, 168.0, 155.6, 140.0, 136.5, 118.6, 118.0, 116.8, 52.2

HRMS (ESI) calculated for [M+H$^+$]: 180.0661, found: 180.0655.

DE-fragments

Synthesis of tert-butyl 4-(2-(allyloxy)-4-amino-5-isopropoxybenzamido)benzoate ***(S19)***

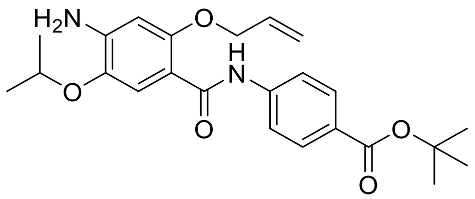

a) Under Argon atmosphere: 2-(allyloxy)-5-isopropoxy-4-nitrobenzoic acid **14** (36.9 mg, 131 µmol, 1.00 Eq) was solved in dry THF (1.2 mL) and $(COCl)_2$ (12 µL, 1.1 Eq) was added. DiPEA (45 µL, 2.0 Eq) was added at 0°C and the solution was stirred at 0°C for 30 min leading to gas generation. Then a solution of *tert*-butyl 4-aminobenzoate (27.9 mg, 1.10 Eq) in dry THF (1.2 mL) and DiPEA (33 µL, 1.5 Eq) were added dropwise at 0°C. The solution was stirred at RT until reaction control showed the end of reaction. The reaction solution was diluted between MTBE (20 mL) and brine (20 mL) and the aqueous phase was extracted with MTBE (20 mL). Combined organic phases were washed with NaOH (0.1 M, 20 mL) and the aqueous phase was extracted with MTBE (20 mL). Combined organic phase were washed with brine (20 mL). Because the aqueous phase still contained UV-absorbing substances it was acidified and reextracted again with EA. Organic phases were combined and solvents removed u.r.p.. The crude product was directly used in next step.

b) Modified from[6, 13]: Zn dust (86 mg, 10 Eq) was added in two portions to a solution of the crude product in THF/EtOH (1 mL each) and AcOH (75 µL, 10 Eq). After 15 min vigourously stirring AcOH (75 µL, 10 Eq) was added and the reaction was stirred further while being monitored by LCMS. After 1 h the reaction solution was diluted between MTBE (20 mL) and sat. $NaHCO_3$ sol. (20 mL) and the aqueous phase was extracted with MTBE (2x20 mL). Combined organic phases were washed with water (20 mL), and dried over Na_2SO_4 before removing solvents u.r.p. to get the crude product which was purified by FCC (solid loading, 100x reactants mass, PE/EA, 90/10->85/15->80/20). The product was obtained as yellow solid (36.2 mg, 65% o2s).

^{1}H NMR (500 MHz, $CDCl_3$): δ = 10.21 (s, 1H, H-N), 7.95 (d, J = 8.9, 2H, H-$C_{Ar,E}$), 7.70 (s, 1H, H-$C_{Ar,D}$), 7.68 (d, J = 9.0, 2H, H-$C_{Ar,E}$), 6.34 (s, 1H, H-$C_{Ar,D}$), 6.17 (ddt, J = 17.2, 10.4, 5.8, 1H), 5.52 (dq, J = 17.2, 1.4, 1H), 5.45 (dq, J = 10.4, 1.1, 1H), 4.65 – 4.56 (m, 3H), 4.43 (s, 2H, H_2N), 1.59 (s, 9H), 1.34 (d, J = 6.0, 6H).

^{13}C NMR (126 MHz, $CDCl_3$): δ = 165.7, 164.0, 152.4, 142.9, 142.6, 139.8, 132.1, 130.7, 126.8, 120.3, 118.9, 116.4, 110.8, 99.7, 80.8, 71.6, 71.1, 28.4, 22.3.

HRMS (ESI): calculated for $[M+H]^+$: 427.2228, found: 427.2231.

Synthesis of tert-butyl 4-(4-amino-2-fluoro-3-isopropoxybenzamido)benzoate ***(S20)***

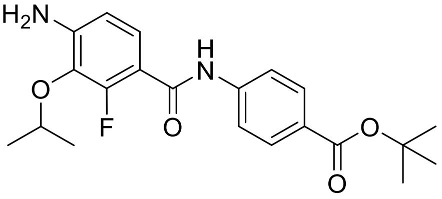

a) 2-fluoro-3-isopropoxy-4-nitro-benzoic acid **17a** (51 mg, 0.21 mmol, 1.0 Eq) and DiPEA (0.18 mL, 5.0 Eq) were solved in dry DCM (1.7 mL) under Argon atmosphere and $POCl_3$ (23 µL, 1.2 Eq) was added at 0°C. The reaction was stirred 5 min at 0°C, then at RT. After 1.5 h, *tert*-butyl 4-aminobenzoate (51 mg, 1.3 Eq, solution in dry DCM (1.7 mL)) was added dropwise. After 2 h DiPEA (0.18 mL, 5.0 Eq) was added again and the reaction was stirred overnight at RT. After LCMS reaction control the reaction mixture was diluted between EA/HCl (0.5 M) (25 mL each) and the aqueous phase was extracted with EA (2x15 mL). The organic phase was washed with brine (10 mL), NaOH (0.5 M, 2x15 mL) and brine (2x10 mL). (The alkaline aqueous solution was later acidified with HCl (2 M), then extracted with EA (2x15 mL) to recover reactant). The combined organic phases containing product were dried over Na_2SO_4 and solvents were removed u.r.p. to give 105 mg crude.

b) Modified from[6, 13]: The crude material was solved in dry THF (1.5 mL) under Ar and EtOH (1.5 mL) was added. AcOH (0.12 mL, 10 Eq) and Zn powder (69 mg, 5.0 Eq) were added and the reaction was stirred vigourously. After 30 min, 2 h and 3.5 h AcOH (3x0.12 mL, 3x10 Eq) and Zn powder (3x69 mg, 3x5.0 Eq) were added again. After 4.5 h reaction and screening with LCMS the mixture was diluted between EA/sat. $NaHCO_3$ sol. (20 mL each) and the aqueous phase was extracted with EA (2x15 mL). The combined organic phases were washed with brine (10 mL), dried over Na_2SO_4 and solvents were removed u.r.p.. The crude product was purified by FCC (solid loading, 80x theoretical product mass, PE/acetone, 85/15->80/20) to give a pale-yellow gum, yield: 52.8 mg, 65% o2s.

^{1}H NMR (500 MHz, Acetone): δ = 9.22 (d, J = 7.6, 1H, H-NCO), 7.94 (d, J = 9.0, 2H), 7.89 (d, J = 9.0, 2H), 7.42 (t, J = 8.4, 1H), 6.65 (dd, J = 8.7, 1.4, 1H), 5.39 – 5.29 (m, 2H, H_2N), 4.49 – 4.38 (m, 1H), 1.58 (s, 9H), 1.33 (dd, J = 6.2, 0.8, 6H).

^{13}C NMR (126 MHz, Acetone): δ = 165.7, 163.3, 155.8 (d, J = 246), 148.6 (d, J = 6.3), 144.2, 131.8 (d, J = 15.3), 131.0 (C_{Ar}-H), 127.6, 126.8 (d, J = 3.8), 119.8 (C_{Ar}-H), 112.1 (d, J = 11.3), 110.7, 80.9, 76.6, 28.4, 22.7.

^{19}F NMR (471 MHz, Acetone): δ = -131.0.

HRMS (ESI): calculated for $[M+H]^+$: 389.1871, found: 389.1871.

Synthesis of tert-butyl 4-(4-amino-2-chloro-3-isopropoxybenzamido)benzoate ***(S21)***

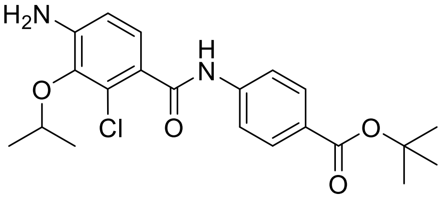

a) Note: The first step was splitted to two equal-sized batches: 2-chloro-3-isopropoxy-4-nitrobenzoic acid **17b** (98 mg in total, 0.38 mmol, 1.0 Eq) and DiPEA (0.32 mL, 5.0 Eq) were solved in dry DCM (3.0 mL) under Argon atmosphere and $POCl_3$ (41 µL, 1.2 Eq) was added at 0°C. The reaction was stirred 5 min at 0°C, then at RT. After ~1 h DiPEA (0.32 µL, 5.0 Eq) and *tert*-butyl 4-aminobenzoate (91 mg, 1.25 Eq, solution in dry DCM (3.0 mL)) were added. The reaction was continued overnight either at 5°C or RT. Both batches were now combined.

After LCMS reaction control the reaction mixture was diluted between EA and HCl (0.5 M) (40 mL each) and the aqueous phase was extracted with EA (2x20 mL). The organic phase was washed with brine (10 mL), sat. $NaHCO_3$ sol. (20 mL), NaOH (0.5 M, 2x20 mL) and brine (1x20 mL). (The alkaline aqueous solution was later acidified with HCl (2 M), then extracted with DCM (2x20 mL) to recover reactant). The combined organic phases were dried over Na_2SO_4 and solvents were removed u.r.p.. The crude product was directly used in the next step:

b) Modified from[6, 13]: The crude material was solved in dry THF (2.5 mL) under Ar and EtOH (2.5 mL) was added. AcOH (0.22 mL, 10 Eq) and Zn powder (123 mg, 5.00 Eq) were added and the reaction was stirred vigourously. After 30 min and 2 h AcOH

(2x0.22 mL, 2x10 Eq) and Zn powder (2x123 mg, 2x5.00 Eq) were added again. After 3 h reaction and screening with LCMS the mixture was diluted between DCM/sat. $NaHCO_3$ sol. (30 mL each) and the aqueous phase was extracted with DCM (2x20 mL). The combined organic phases were dried over Na_2SO_4 and solvents were removed u.r.p.. The crude product was purified by FCC (solid loading, 100x theoretical product mass, PE/acetone, 85/15->80/20) to give an off-colorless foam, yield: 82.2 mg, 54% o2s.

^{1}H NMR (500 MHz, Acetone): δ = 9.52 (s, 1H, H-NCO), 7.95 (d, J = 8.9, 2H), 7.89 (d, J = 9.0, 2H), 7.16 (d, J = 8.2, 1H), 6.76 (d, J = 8.4, 1H), 5.16 – 5.05 (m, 2H), 4.53 (hept, J = 6.1, 1H), 1.58 (s, 9H), 1.34 (d, J = 6.1, 6H).

^{13}C NMR (126 MHz, Acetone): δ = 166.2, 165.7, 146.7, 144.4, 141.2, 131.1, 127.6, 126.4, 126.1, 125.9, 119.4, 113.5, 80.9, 76.2, 28.4, 22.7.

HRMS (ESI): calculated for $[M+H]^+$: 405.1576, found: 405.1575.

Synthesis of tert-butyl 4-(4-amino-2-bromo-3-isopropoxybenzamido)benzoate ***(S22)***

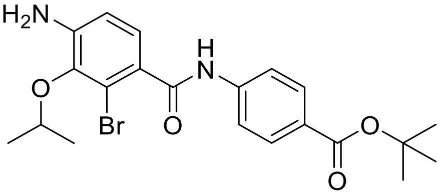

a) 2-bromo-3-isopropoxy-4-nitrobenzoic acid **17c** (43.8 mg, 0.144 mmol, 1.00 Eq) and DiPEA (0.12 mL, 5.0 Eq) were solved in dry DCM (1.2 mL) and $POCl_3$ (16 µL, 1.2 Eq) was added at 0°C. The reaction was stirred 5 min at 0°C, then at RT. After 1.5 h *tert*-butyl 4-aminobenzoate (35 mg, 1.25 Eq, solution in dry DCM (1.2 mL)) was added dropwise. After 2 h, DiPEA (0.12 mL, 5.0 Eq) was added again and the reaction was stirred overnight at RT. After LCMS reaction control the reaction mixture was diluted between EA/HCl (0.5 M) (25 mL each) and the aqueous phase was extracted with EA (2x15 mL). The organic phase was washed with brine (10 mL), NaOH (0.5 M, 2x15 mL) and brine (2x10 mL) (=organic phase 1). The washing solution was acidified with HCl (2 M), extracted with EA (2x15 mL) and this organic phase was again washed with NaOH (0.2 M, 20 mL) (=organic phase 2). (The alkaline aqueous solution was later acidified with HCl, then extracted with EA (2x15 mL) to recover reactant). Organic

phases 1 and 2 were combined, dried over Na_2SO_4 and solvents were removed u.r.p. to give 87 mg crude material.

b) Modified from[6, 13]: The crude material was solved in dry THF (1.0 mL) under Ar and EtOH (1.0 mL) was added. AcOH (82 µL, 10 Eq) and Zn powder (47 mg, 5.0 Eq) were added and the reaction was stirred vigourously. After 30 min, 2 h and 3.5 h AcOH (3x82 µL, 3x10 Eq) and Zn powder (3x47 mg, 3x5.0 Eq) were added again. After 4 h reaction and screening with LCMS, the mixture was diluted between EA/sat. $NaHCO_3$ sol. (20 mL each) and the aqueous phase was extracted with EA (2x15 mL). The combined organic phases were washed with brine (10 mL), dried over Na_2SO_4 and solvents were removed u.r.p.. The crude product was purified by FCC (solid loading, 100x theoretical product mass, PE/acetone, 85/15->80/20) to give a pale-yellow gum, yield: 46.5 mg, 72% o2s, NMR: 66%.

^{1}H NMR (500 MHz, Acetone): δ = 9.51 (s, 1H, H-NCO), 7.95 (d, J = 8.9, 2H), 7.89 (d, J = 9.0, 2H), 7.10 (d, J = 8.2, 1H), 6.79 (d, J = 8.2, 1H), 5.07 (s, 2H, H_2N), 4.57 (hept, J = 6.1, 1H), 1.58 (s, 9H), 1.35 (d, J = 6.1, 6H).

^{13}C NMR (126 MHz, Acetone): δ = 167.2, 165.7, 146.4, 144.4, 142.3, 131.1, 128.7, 127.6, 125.8, 119.4, 116.5, 114.2, 80.9, 76.3, 28.4, 22.6.

HRMS (ESI): calculated for $[M+H]^+$: 449.1071/ 451.1050, found: 449.1069/ 451.1051.

Synthesis of tert-butyl 4-(8-isopropoxy-2-methyl-7-nitro-4-oxoquinazolin-3(4H)-yl)benzoate ***(70)***

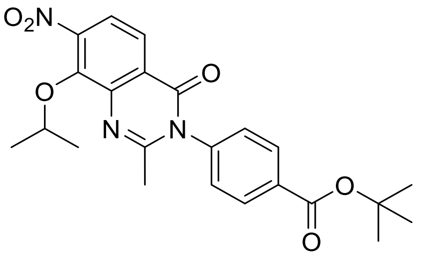

a) Methyl 2-acetamido-3-isopropoxy-4-nitrobenzoate **20** (27 mg, 0.11 mmol, 1.0 Eq, non-purified product) was solved in THF/water (0.5 mL each) and $LiOH*H_2O$ (22 mg, 5.0 Eq) was added. After stirring for 45 min the reaction solution was diluted between EA and H_2O (10 mL each). The organic phase was extracted again with NaOH (0.1 M, 10 mL) and the combined aqueous phases were washed with EA (2x10 mL) (organic phase 1) before acidification with HCl (conc., ~3 mL) and extraction with EA (3-4x

15 mL) (organic phase 2). To ensure full product recovery organic phase 1 was extracted again with NaOH (2x20 mL). The aqueous phase was acidified with HCl (conc.) and extracted with EA (2x20 mL). This fraction and organic phase 2 were combined, dried over Na_2SO_4 and solvents were removed u.r.p. and by coevaporation with heptane to give a colorless-yellowish solid (22.2 mg) used without further purification.

b) The vacuum-dried solid with intermediate **69** (22.2 mg crude, 79 µmol, 1.0 Eq) was suspended in dry DCM (0.8 mL) under Ar and $(COCl)_2$ was added (8.4 µL, 1.25 Eq). Then a catalytic amount of DMF (0.4 µL, 0.07 Eq) was added and the reaction stirred 1 h while being screened by LCMS. Additional $(COCl)_2$ (5.0 µL, 0.75 Eq) and DMF (0.4 µL, 0.07 Eq) were added and the reaction was stirred 30 min before removing solvents and reagent u.r.p. and high vacuum. The residue was solved in dry DCM (0.8 mL) and *tert*-butyl 4-aminobenzoate (21 mg, 1.4 Eq) and pyridine (19 µL, 3.0 Eq) were added. The reaction was stirred for 1 h while being screened with TLC. The reaction mixture was then diluted between DCM and water (15 mL each) and the aqueous phase was extracted with DCM (1x15 mL). The aqueous phase was acidified with HCl (1 M) and extracted again with EA (2x20 mL). The combined organic phases were dried over Na_2SO_4 and solvent was removed u.r.p.. The crude product was purified by FCC (solid loading, 100x reactants mass, PE/EA, 85/15) to give a light green oil. A mixed fraction was washed again with $NaHSO_4$ (1 M, 2x10 mL) and HCl (0.1 M, 6x) and combined with the other product fractions to give 14.5 mg (42% o2s, 31% o3s) material.

^{1}H NMR (500 MHz, $CDCl_3$): δ = 8.20 (d, J = 8.5, 2H), 8.01 (d, J = 8.7, 1H), 7.63 (d, J = 8.5, 1H), 7.34 (d, J = 8.7, 2H), 5.30 (hept, J = 6.2, 1H), 2.28 (s, 3H), 1.63 (s, 9H), 1.40 (d, J = 6.1, 6H).

^{13}C NMR (126 MHz, CDCl3): δ = 164.6, 161.2, 154.1, 148.4, 146.6, 143.1, 140.7, 133.6, 131.5, 128.0, 124.3, 121.5, 121.1, 82.1, 79.4, 28.3, 24.8, 22.6.

HRMS (ESI): calculated for $[M+H]^+$: 440.1816, found: 440.1818.

Synthesis of tert-butyl 4-(7-amino-8-isopropoxy-2-methyl-4-oxoquinazolin-3(4H)-yl)benzoate ***(S23)***

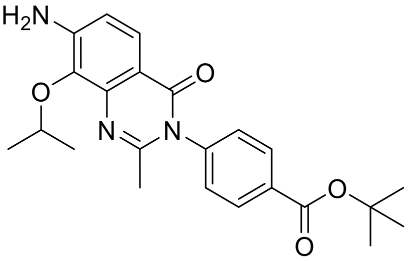

Modified from[6, 13]: *tert*-butyl 4-(8-isopropoxy-2-methyl-7-nitro-4-oxoquinazolin-3(4H)-yl)benzoate **70** (12.9 mg, 29.4 µmol, 1.00 Eq) was solved in EtOH (0.33 mL) and dry THF (0.13 mL). Then AcOH (44 µL, 27 Eq) and Zn dust (15 mg, 8.0 Eq) were added and the reaction was stirred at RT and monitored with LCMS. Another portion Zn dust was added (15 mg, 8.0 Eq) after 1.5 h and after 2 h the suspension was diluted between sat. $NaHCO_3$ sol. (15 mL) and DCM (15 mL). The aqueous suspension was extracted with DCM (2x10 mL) and the organic phase was dried over Na_2SO_4 and solvent removed u.r.p. to get 10.9 mg, 91% of a light-yellow solid used without further purification.

^{1}H NMR (500 MHz, CDCl3): δ = 8.16 (d, J = 8.7, 2H), 7.82 (d, J = 8.5, 1H), 7.31 (d, J = 8.7, 2H), 6.84 (d, J = 8.5, 1H), 4.93 (hept, J = 6.2, 1H), 2.27 (s, 3H), 1.62 (s, 9H), 1.40 (d, J = 6.3, 6H).

^{13}C NMR (126 MHz, $CDCl_3$): δ = 164.8, 161.7, 146.7, 141.5, 136.5, 133.0, 131.5, 131.2, 128.4, 128.2, 123.3, 115.3, 112.1, 81.8, 76.6, 28.3, 24.5, 22.8.

HRMS (ESI): calculated for $[M+H]^+$: 410.2074, found: 410.2075.

Synthesis of tert-butyl 4-(7-amino-8-isopropoxy-4-oxoquinazolin-3(4H)-yl)benzoate ***(74)***

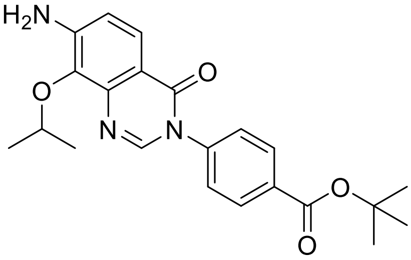

a) 2-amino-3-isopropoxy-4-nitrobenzoic acid **73** (60 mg, 0.25 mmol, 1.0 Eq) was solved in HCOOH (98%, 2.5 mL) and Ac_2O (24.8 µL, 1.05 Eq) was added. The reaction mixture was stirred for 1 h 15 min while being monitored by LCMS. The reaction was quenched by solving the reaction mixture between DCM (20 mL) and H_2O (15 mL). The aqueous phase was extracted with DCM (3x20 mL) and the organic phases were combined and dried over Na_2SO_4 before removing solvent u.r.p. to give a brown-yellow solid (50.8 mg, 76%) directly used in next reaction.

b) Note: The following reaction step was first conducted with DiPEA as base leading to very small turnover and the reactants and products were therefore recovered by aequeos workup.

The recovered material containing the crude formyl-intermediate (45.5 mg, 0.17 mmol, 1.0 Eq) and *tert*-butyl 4-aminobenzoate (49 mg, 1.5 Eq) was solved in dry DCM (1.7 mL) together with DMF (2.0 µL, 15 mol%). Then $POCl_3$ (31 µL, 2.0 Eq) was added and the reaction was stirred for 1 h before adding *tert*-butyl 4-aminobenzoate again (49 mg, 1.5 Eq) and dry pyridine (55 µL, 4.0 Eq). After 2 h stirring at RT the reaction mixture was diluted between DCM/HCl (0.5 M) (20 mL each) and the aqueous phase was extracted with DCM (2x15 mL). Combined organic phases were washed with brine (10 mL), the washing solution was reextracted with DCM (1x10 mL) and all organic phases were combined and dried over Na_2SO_4. The solvent was removed u.r.p. to give a brown crude product directly used in the next step.

c) Modified from[6, 13]: The crude product was suspended in THF/EtOH mixture (1.2 mL each) and AcOH (0.29 mL, 30 Eq) was added. Then Zn dust (166 mg, 15 Eq) was added and the reaction was stirred vigourously while being monitored by LCMS. After 3 h the mixture was diluted between EA/sat. $NaHCO_3$ sol. (20 mL each). The aqueous phase was extracted with EA (2x15 mL) and the combined organic phases were washed with brine (1x5 mL). The organic phase was dried over Na_2SO_4, filtered through pore size 4 and solvents were removed u.r.p.. The residue was purified by FCC (solid loading, 100x theoretical product mass, PE/EA, 70/30) to give 30.2 mg (34% o3s) of an off colorless gum.

^{1}H NMR (500 MHz, Acetone): δ = 8.14 (s, 1H, N_2C-H), 8.12 (d, J = 8.7, 2H), 7.77 (d, J = 8.5, 1H), 7.66 (d, J = 8.9, 1H), 6.99 (d, J = 8.5, 1H), 5.42 (s, 2H, H_2N), 5.04 (hept, J = 6.1, 1H), 1.62 (s, 9H), 1.33 (d, J = 6.3, 6H).

^{13}C NMR (126 MHz, Acetone): δ = 165.3, 160.5, 148.7, 145.6, 142.9, 142.9, 137.4, 132.7, 130.8, 128.2, 123.3, 115.9, 113.4, 81.8, 76.4, 28.3, 22.8.

HRMS (ESI): calculated for $[M+H]^+$: 396.1918, found: 396.1918.

Synthesis of tert-butyl 4-(2,4-diamino-3-isopropoxybenzamido)benzoate ***(S24)***

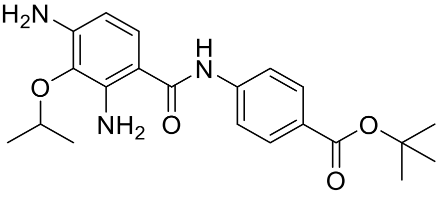

a) 2-amino-3-isopropoxy-4-nitrobenzoic acid **24** (80.5 mg, 0.335 mmol, 1.00 Eq) and *tert*-butyl 4-aminobenzoate (130 mg, 2.00 Eq) were suspended in dry DCM (2.7 mL) under Argon atmosphere and dry pyridine (68 µL, 2.5 Eq) was added. After a clear solution was formed T3P (50%-sol. in EA, 0.30 mL, 1.5 Eq) was added dropwise. The reaction was carefully screened by LCMS and after 75 min the mixture was diluted between HCl (0.1 M) and EA (30 mL each) and the aqueous phase was extracted with EA (2x20 mL). The combined organic phases were dried over Na_2SO_4 before removing solvents u.r.p.. The material was directly used in the next step.

b) Modified from[6, 13]: The material was solved in a THF/EtOH-mixture (2.5 mL each) and Zn dust (219 mg, 10.0 Eq) was added. Then AcOH (2x0.19 mL, 2x10 Eq) was added dropwise directly and after 30 min while the reaction was screened by LCMS. After 1 h 20 min AcOH (0.19 mL, 10.0 Eq) was added again. After 1 h 45 min the mixture was diluted between sat. $NaHCO_3$ sol./EA (30 mL each) and the aqueous phase was extracted with EA (2x20 mL). The combined organic phases were washed with brine (5 mL) and dried over Na_2SO_4 before removing solvents u.r.p.. The material was then purified by FCC (solid loading, 100x reactants mass (21 g-scale column), cyclohexane/EA, 85/15->80/20->70/30) to give an almost colorless solid after solvent removal u.r.p. and coevaporation with Cy (3x). Yield: 48.4 mg, 37% o2s.

^{1}H NMR (500 MHz, THF): δ = 9.04 (s, 1H, H-NCO), 7.88 (d, J = 8.9, 2H), 7.77 (d, J = 9.0, 2H), 7.20 (d, J = 8.7, 1H, H-$C_{Ar,D}$), 6.11 (s, 2H, H_2N), 5.98 (d, J = 8.7, 1H, H-$C_{Ar,D}$), 4.69 (s, 2H, H_2N), 4.34 (hept, J = 6.3, 1H), 1.57 (d, J = 1.1, 9H), 1.30 (d, J = 6.1, 6H).

^{13}C NMR (126 MHz, THF): δ = 168.8, 165.7, 146.8, 145.9, 145.2, 131.0, 130.9, 126.8, 125.0, 119.5, 106.8, 104.1, 80.4, 73.2, 28.5, 23.0.

HRMS (ESI): calculated for $[M+H]^+$: 386.2075, found: 386.2075.

Synthesis of tert-butyl 4-(5-amino-6-isopropoxypicolinamido)benzoate ***(S25)***

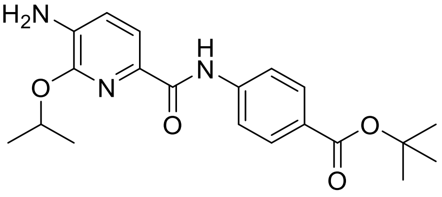

a) 6-isopropoxy-5-nitropicolinic acid **27** (207 mg, 0.915 mmol, 1.00 Eq) and *tert*-butyl 4-aminobenzoate (230 mg, 1.30 Eq) were solved in dry EA (9 mL) under Argon atmosphere. Then dry pyridine (0.30 mL, 4.0 Eq) was added, T3P (50% sol. in EA, 1.09 mL, 2.00 Eq) was added dropwise and the yellow solution was stirred at RT. After ~5 min a colorless precipitate was formed. The reaction was screened with LCMS and after 1 h the reaction mixture was diluted between EA/HCl (0.5 M) (40 mL each). The aqueous phase was extracted with EA (3x20 mL) and the combined organic phases were dried over Na_2SO_4. Solvents were removed u.r.p. to give a light-yellow amorphous solid that was taken directly for next reaction.

b) Modified from[6, 13]: The crude product was secured under Ar and solved in THF/EtOH (4.5 mL each). Then AcOH (1.05 mL, 20.0 Eq) and Zn powder (598 mg, 10.0 Eq) were added and the mixture was stirred vigourously at RT while being monitored with LCMS. After 1.5 h the mixture was suspended between EA/sat. $NaHCO_3$ sol. (50 mL each) and the aqueous phase was extracted with EA (2x25 mL). The combined organic phases were washed with brine (1x10 mL), dried over Na_2SO_4 and solvents were removed u.r.p.. The crude product was purified by FCC (solid loading, 80x theoretical product mass, PE/EA, 80/20) and solvents were removed u.r.p. and by coevaporation with MTBE and cyclohexane to give 371 mg, >98%, NMR: 81% (cyclohexane residue), of a light brown highly viscous oil.

^{1}H NMR (700 MHz, CD_3CN): δ = 9.73 (s, 1H, H-NCO), 7.95 (d, *J* = 8.8, 2H), 7.83 (d, *J* = 8.8, 2H), 7.63 (d, *J* = 7.9, 1H), 7.00 (d, *J* = 7.9, 1H), 5.54 (hept, *J* = 6.2, 1H), 4.77 (s, 2H, H_2N), 1.57 (s, 9H), 1.42 (d, *J* = 6.1, 6H).

^{13}C NMR (176 MHz, CD_3CN): δ = 166.1, 164.0, 150.6, 143.6, 137.6, 134.5, 131.2, 127.7, 119.7, 119.2, 118.6, 81.4, 69.8, 28.4, 22.3.

HRMS (ESI): calculated for $[M+H]^+$: 372.1918, found: 372.1920.

*Synthesis of tert-butyl 4-(4-amino-2-(difluoromethyl)-3-isopropoxybenzamido)benzoate (**S26**)*

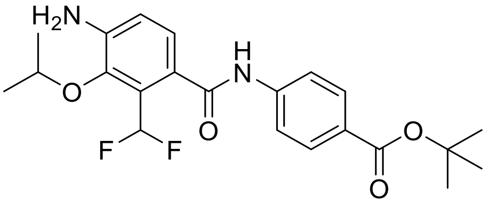

a) 2-(Difluoromethyl)-3-isopropoxy-4-nitrobenzoic acid **32** (293 mg, 1.07 mmol, 1.0 Eq) and *tert*-butyl 4-aminobenzoate (196 mg, 1.01 mmol, 0.95 Eq) were dissolved in DCM (5 mL). The solution was cooled to 0°C and DIPEA (316 µL, 1.81 mmol, 1.70 Eq) and $POCl_3$ (99 µL, 1.07 mmol, 1.00 Eq) were added. The solution was stirred for 2 h at 0°C. A sat. NH_4Cl solution was added and the aq. phase was extracted with DCM (3x). The combined organic phases were dried over $MgSO_4$, filtered and concentrated under reduced pressure. The crude product was purified by column chromatography (PE/EtOAc = 3:1) to furnish the intermediate (395 mg) as orange amorphous solid, which contained small impurities. The product was used in the next step without further purification.

R*f* (PE/EtOAc = 5:1) = 0.37;

HRMS (ESI): calculated for $[M+Na]^+$: 473.1500; found: 473.1479.

b) The crude material (395 mg, 0.88 mmol, 1.0 Eq) was dissolved in THF (1.5 mL) and EtOH (1.3 mL). Zinc dust (0.86 g, 13 mmol, 15 Eq) was added. The mixture was cooled to 0°C and AcOH (0.80 mL, 13 mmol, 15 Eq) was slowly added dropwise. The mixture was warmed to RT and stirred for 6 h, before Et_2O and a sat. $NaHCO_3$ solution were added. The mixture was filtered and the phases were separated. The aq. phase was extracted with Et_2O (3x). The combined organic phases were washed with brine, dried over $MgSO_4$, filtered and concentrated under reduced pressure. The crude product was purified by column chromatography (dry load, PE/EtOAc = 2:1) to furnish the title compound (154 mg, 0.37 mmol, 35% over two steps) as yellowish amorphous solid.

R*f* (PE/EtOAc = 2:1) = 0.22;

^{1}H NMR (500 MHz, DMSO-d_6): δ = 10.40 (s, 1H, NH), 7.86-7.84 (d, J = 8.8 Hz, 2H, H_{Ar}), 7.80-7.78 (d, J = 8.8 Hz, 2H, H_{Ar}), 7.22-6.95 (t, J = 54.2 Hz, 1H, CHF_2), 7.13-7.09 (d, J = 8.6 Hz, 1H, H_{Ar}), 6.88-6.86 (d, J = 8.5 Hz, 1H, H_{Ar}), 5.44 (s, 2H, NH_2), 4.37-4.35 (sept, J = 6.1 Hz, 1H, $C\underline{H}(CH_3)_2$), 1.54 (s, 9H, $C(C\underline{H}_3)_3$), 1.27-1.26 (d, J = 6.1 Hz, 6H, $CH(C\underline{H}_3)_2$)

^{13}C NMR (126 MHz, DMSO-d$_6$): *δ* = 166.9 (CO), 164.6 (CO), 144.6 (C_{Ar}), 143.7 (C_{Ar}), 142.1-142.0 (t, *J* = 4.9 Hz, C_{Ar}), 129.9 (C_{Ar}), 125.6 (C_{Ar}), 125.2-124.9 (t, *J* = 21.8 Hz, C_{Ar}), 124.8 (C_{Ar}), 123.9 (C_{Ar}), 118.6 (C_{Ar}), 115.7 (C_{Ar}), 113.9-110.8 (t, *J* = 236.1 Hz, CHF_2), 80.2 ($C(\underline{C}H_3)_3$), 75.1 ($\underline{C}H(CH_3)_2$), 27.9 ($C(\underline{C}H_3)_3$), 21.8 ($CH(\underline{C}H_3)_2$)
HRMS (ESI) calculated for $C_{22}H_{26}F_2N_2O_4Na$ $[M+Na]^+$: 443.1758; found: 443.1756.

Synthesis of allyl 4-(2-(allyloxy)-4-amino-3-isobutylbenzamido)benzoate ***(S27)***

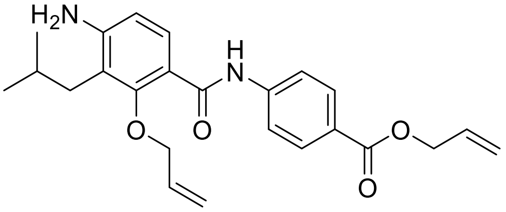

a) 2-(allyloxy)-4-((*tert*-butoxycarbonyl)amino)-3-isobutylbenzoic acid **39** (37.6 mg, 0.108 mmol, 1.00 Eq) and 4-amino allylbenzoate (synthesized as reported in[10], 31 mg, 1.6 Eq) were solved in dry EA (1.1 mL) and pyridine (43 µL, 5.0 Eq) was added. After stirring for 5 min, T3P (50% in EA, 0.13 mL, 2.0 Eq) was added dropwise. The mixture was stirred at RT and screened by LCMS. After 1 h the mixture was diluted between EA/HCl (0.1 M) (20 mL each), the aqueous phase was extracted with EA (2x20 mL), combined organic phases were dried over Na_2SO_4 and solvents were removed u.r.p..

b) The crude material was solved in HCl (sol. in dioxane, 4 M, 1.0 mL) and the reaction was stirred at RT. After 1.5 h and screening by TLC the reaction mixture was diluted between EA/sat. $NaHCO_3$ sol. (20 mL each) and the aqueous phase was extracted with EA (2x20 mL) before drying the combined organic phases over Na_2SO_4 and removing solvents u.r.p.. The crude product was purified by FCC (solid loading, 100x theoretical product mass, PE/EA, 85/15->70/30) to give a light red gum, 34.6 mg, 79% o2s.

^{1}H NMR (700 MHz, $CDCl_3$): *δ* = 10.00 (s, 1H), 8.03 (d, *J* = 8.8, 2H), 7.89 (d, *J* = 8.5, 1H), 7.74 (d, *J* = 8.8, 2H), 6.63 (d, *J* = 8.5, 1H), 6.12 – 6.00 (m, 2H), 5.51 (dd, *J* = 17.2, 1.6, 1H), 5.41 (dd, *J* = 17.2, 0.9, 1H), 5.36 (dd, *J* = 10.5, 1.4, 1H), 5.28 (dd, *J* = 10.4, 1.3, 1H), 4.81 (d, *J* = 5.7, 2H), 4.4 (br s, 1H, H_2N), 4.35 (d, *J* = 5.1, 2H), 2.43 (d, *J* = 7.3, 2H), 1.98 (hept, *J* = 6.8, 1H), 0.99 (d, *J* = 6.7, 6H).

^{13}C NMR (176 MHz, $CDCl_3$): *δ* = 166.0, 164.1, 156.6, 149.4, 143.2, 132.6, 132.5, 131.0, 130.9, 125.0, 119.2, 119.1, 118.4, 118.2, 116.7, 112.7, 75.8, 65.5, 33.8, 28.2, 22.9.

HRMS (ESI): calculated for $[M+H]^+$: 409.2122, found: 409.2123.

Synthesis of methyl 4-((4-(tert-butoxycarbonyl)phenyl)carbamoyl)-3-hydroxy-2-isopropoxybenzoate ***(S28)***

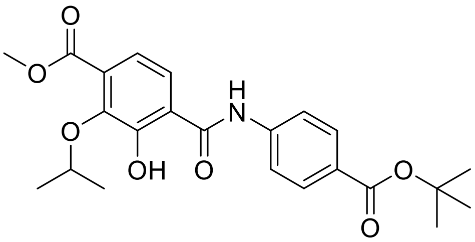

2-hydroxy-3-isopropoxy-4-(methoxycarbonyl)benzoic acid **57** (131 mg, 515 µmol, 1.00 Eq) and *tert*-butyl 4-aminobenzoate (149 mg, 1.50 Eq) were suspended in dry toluene (1.3 mL) under Argon atmosphere and $P(OPh)_3$ (0.18 mL, 1.3 Eq) was added. The reaction was refluxed for 24 h and the product was directly purified by FCC (solid loading, 75x theoretical product mass, PE/EA, 90/10->85/15->80/20). (Note: TLC was not sufficient to identify product fractions, instead LCMS was used). The product was isolated with a yield of 168 mg, 76% containing an unquantified residual fraction of phenolic compounds.

^{1}H NMR (500 MHz, $CDCl_3$): δ = 9.27 (s, 1H), 9.22 (s, 1H), 8.00 (d, J = 9.0, 2H), 7.72 (d, J = 9.0, 2H), 7.71 (d, J = 8.5, 2H), 7.40 (d, J = 8.5, 1H), 4.54 (hept, J = 6.1, 1H), 3.94 (s, 3H), 1.60 (s, 9H), 1.36 (d, J = 6.1, 6H).

^{13}C NMR (126 MHz, $CDCl_3$): δ = 166.0, 165.4, 164.6, 151.7, 145.8, 141.3, 130.8, 130.2, 128.2, 123.3, 121.4, 120.3, 119.8, 81.2, 78.7, 52.6, 28.4, 22.5.

HRMS (ESI): calculated for $[M+H]^+$: 430.1861, found: 430.1862.

Synthesis of 3-(allyloxy)-4-((4-(tert-butoxycarbonyl)phenyl)carbamoyl)-2-isopropoxybenzoic acid ***(58)***

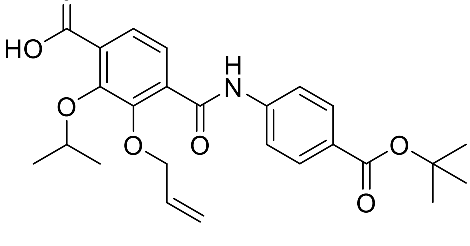

a) Methyl 4-((4-(*tert*-butoxycarbonyl)phenyl)carbamoyl)-3-hydroxy-2-isopropoxybenzoate purified material **S28** (162 mg, 0.377 mmol, 1.00 Eq) and K_2CO_3

(104 mg, 2.00 Eq) were suspended in dry DMF (1.9 mL) under Argon atmosphere. The suspension was stirred for 10 min before adding AllBr (36 µL, 1.1 Eq) and stirring further at RT while being screened with LCMS. After 5 h reaction the mixture was diluted between EA/HCl (0.1 M) (30 mL each) and the aqueous phase was extracted with EA (2x20 mL). Organic phases were combined, dried over Na_2SO_4 and solvents were removed u.r.p. to give a yellowish oil directly used in saponification.

b) The crude material and LiOH*H_2O (63 mg, 4.0 Eq) were solved in a THF/H_2O mixture (1.3 mL each) forming a 2-phase system. The reaction was stirred at RT while being screened with LCMS. After 3 h the mixture was diluted between EA/HCl (0.1 M) (20 mL each) and the aqueous phase was extracted with EA (2x20 mL). Organic phases were combined and dried over Na_2SO_4 before removing solvents u.r.p.. The crude product was purified by FCC (solid loading, 85x reactant mass, PE/EA/AcOH, 80/15/5). Pure product fractions were identified with NMR and the product was isolated as a colorless fluffy solid, 115 mg, 67% o2s, 51% o3s.

^{1}H NMR (500 MHz, Acetone): δ = 11.50 (br s, 1H), 10.23 (s, 1H), 7.97 (d, J = 8.7, 2H), 7.87 (d, J = 9.0, 2H), 7.75 (d, J = 8.2, 1H), 7.69 (d, J = 8.2, 1H), 6.17 (ddt, J = 17.2, 10.4, 5.8, 1H), 5.51 (dq, J = 17.2, 1.6, 1H), 5.31 (dq, J = 10.4, 1.2, 1H), 4.76 (dt, J = 5.8, 1.4, 2H), 4.75 – 4.68 (m, 1H), 1.59 (s, 9H), 1.33 (d, J = 6.1, 6H).

^{13}C NMR (126 MHz, Acetone): δ = 166.4, 165.6, 163.8, 151.5, 151.4, 143.5, 134.1, 133.4, 131.2, 128.2, 126.8, 125.5, 119.9, 119.8, 119.3, 81.1, 78.4, 75.8, 28.3, 22.6.

HRMS (ESI): calculated for $[M+H]^+$: 456.2017, found: 456.2018.

*Synthesis of tert-butyl 4-((2-(allyloxy)-3-isopropoxy-4-nitrobenzyl)amino)benzoate (**S29**)*

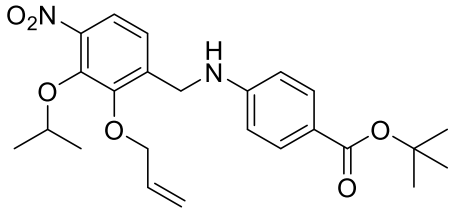

2-(allyloxy)-3-isopropoxy-4-nitrobenzaldehyde[10] **52** (100 mg, 0.38 mmol, 1.0 Eq) and *tert*-butyl 4-aminobenzoate (73 mg, 0.38 mmol, 1.0 Eq) were added to a dry flask and further dried under high vacuum while stirring. Dry DCM (2.0 mL) was added under argon atmosphere and stirred overnight. The solution was cooled down to 0°C and 0.2 ml acetic acid (0.2 mL, 9 Eq) was added under argon atmosphere. Sodium borohydride (43 mg, 3 Eq) was added portion wise under argon atmosphere. The reaction was controlled by TLC. After completion, the reaction was quenched with saturated $NaHCO_3$ (8 mL) and brine (12 mL). The aqueous layer was extracted with DCM (3x8 mL). The combined organic layers were concentrated under reduced pressure. The product was purified by chromatography. A yellow semi-solid was obtained, yield: 141.3 mg, 85 %.

^{1}H NMR (500 MHz, $CDCl_3$): δ = 7.81 (d, 2H, *J* = 8.7 Hz), 7.47 (d, 1H, *J* = 8.5 Hz), 7.11 (d, 1H, *J* = 8.2 Hz), 6.55 (d, 2H, *J* = 8.2 Hz), 6.06 (ddt, 1H, *J* = 5.9 Hz, 10.4 Hz, 22.3 Hz), 5.40 (dd, 1H, *J* = 1.4 Hz, 17.1 Hz), 5.30 (dd, 1H, *J* = 1.2 Hz, 10.4 Hz), 4.64 (dd, 2H, *J* = 1.1 Hz, 5.9 Hz), 4.62 – 4.56 (m, 1H), 4.46 (s, 2H), 1.55 (s, 9H), 1.30 (d, 6H, *J* = 6.2 Hz)

^{13}C NMR (126 MHz, $CDCl_3$): δ = 166.0, 151.3, 145.4, 144.8, 138.4, 133.2, 131.5, 130.8, 122.5, 119.9, 119.1, 112.0, 80.2, 77.9, 74.1, 43.1, 28.5, 22.5

*Synthesis of tert-butyl 4-((2-(allyloxy)-4-amino-3-isopropoxybenzyl)(tert-butoxycarbonyl)amino)benzoate (**53**)*

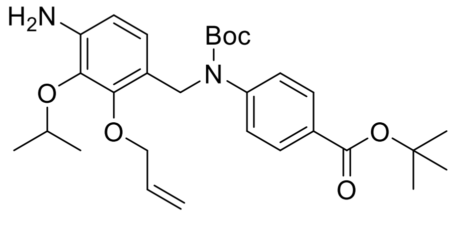

tert-butyl 4-((2-(allyloxy)-3-isopropoxy-4-nitrobenzyl)amino)benzoate **S29** (137 mg, 0.31 mmol, 1.0 Eq) and DMAP (4 mg, 0.1 Eq) were added to a dry flask and further dried under high vacuum. Dry THF (0.6 mL), Boc_2O (79 µL, 1.1 Eq) and dry triethylamine (52 µL, 0.37 mmol, 1.2 Eq) were added. The reaction was stirred at RT overnight. After completion, the solvent was partially removed under reduced pressure and further dried under high vacuum. The crude residue was dissolved in THF (1.3 mL) and EtOH (1.1 mL) and cooled down to 0°C. Zinc dust (280 mg, 14 Eq) was added. Acetic acid (0.24 mL, 14 Eq) was added to the stirring mixture over 1 h and the mixture was allowed to cool down to RT. After completion, the zinc dust was filtered off. Saturated $NaHCO_3$ (10 mL) was added to the solution and it was extracted with EtOAc (3x5 mL). The combined organic phases were absorbed on silica and the product was purified by chromatography. A yellow foam was obtained, yield: (133.2 mg, 84 % o2s).

^{1}H NMR (500 MHz, THF): *δ* = 7.79 (d, 2H, *J* = 8.8 Hz), 7.22 (d, 2H, *J* = 8.7 Hz), 6.68 (d, 1H, *J* = 8.3 Hz), 6.34 (d, 1H, *J* = 8.3 Hz), 6.05 – 5.96 (m, 1H), 5.28 (dq, 1H, *J* = 1.7 Hz, 17.2 Hz), 5.11 (ddd, 1H, *J* = 1.3 Hz, 3.2 Hz, 10.5 Hz), 4.83 (s, 2H), 4.41 (quart., 1H, *J* = 6.2 Hz), 4.34 (dt, 2H, *J* = 1.5 Hz, 5.5 Hz), 4.30 (br s, 2H), 1.54 (s, 9H), 1.40 (s, 9H), 1.19 (d, 6H, *J* = 6.2 Hz)

^{13}C NMR (176 MHz, THF): *δ* = 165.2, 154.4, 150.7, 147.6, 143.4, 137.8, 135.7, 129.9, 129.1, 126.3, 123.9, 120.3, 116.3, 111.0, 80.6, 80.4, 74.4, 73.5, 48.1, 28.3, 28.1, 22.6

Synthesis of 4-(4-(2-hydroxy-3-isopropoxy-4-nitrophenyl)-1H-1,2,3-triazol-1-yl)benzoic acid ***(S30)***

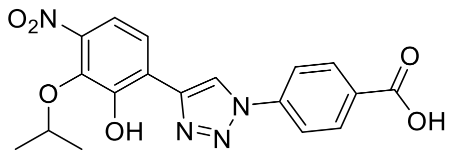

a) 2-isopropoxy-3-nitro-6-((trimethylsilyl)ethynyl)phenyl acetate **63** (100 mg, 0.298 mmol, 1.00 Eq) was solved in MeOH (7 mL) and K_2CO_3 (82 mg, 2.0 Eq) was added. The mixture stirred 15 min, before it was diluted between DCM (30 mL), water (30 mL) and HCl (1 M, 2 mL). The aqueous phase was extracted with DCM (2x20 mL). Organic phases were combined and solvents were removed u.r.p. to use this material in the next step.

b) The crude product was solved in *t*BuOH (3 mL) and 4-Azidobenzoic acid (73 mg, 0.45 mmol, 1.5 Eq) was added. Then a solution of $CuSO_4$ pentahydrate (7.4 mg, 0.10 Eq), Na-ascorbate (30 mg, 0.50 Eq) and THPTA (8 mg, 0.06 Eq) in H_2O (2 mL) was added. The reaction produced a suspension after few minutes and was stirred overnight. The reaction mixture was concentrated u.r.p. to remove *t*BuOH and was then diluted between EA (30 mL) and water (30 mL). The aqueous phase was extracted with EA (2x30 mL). The combined organic phases were washed with brine (1x30 mL) and dried over Na_2SO_4. To purify the product, the organic phase was concentrated slowly u.r.p. at 40°C. Once precipitation occured, the solution was put in fridge for 90 min and was filtrated over pore size 4. After washing with small portions of cold EA, the precipitate was washed out with acetone and the solution was concentrated u.r.p.. The filtrate solution was concentrated again u.r.p. at 60°C until precipitation and was stored at RT over weekend. The filtration was repeated and the solid was washed with MeCN and extracted with acetone to give a yield of 74 mg, 65% o2s product as a pale-yellow solid. NMR showed 6 mol% reactant residue that was tolerated.

^{1}H NMR (500 MHz, THF): δ = 11.70 (br s, 1H, COOH), 10.24 (s, 1H, OH), 9.06 (s, 1H, $H_{triazol}$), 8.24 (d, J = 8.7, 2H), 8.08 (d, J = 8.7, 2H), 7.88 (d, J = 8.7, 1H), 7.44 (d, J = 8.7, 1H), 4.61 (hept, J = 6.1, 1H), 1.34 (d, J = 6.2, 6H).

^{13}C NMR (126 MHz, THF): δ = 172.0, 166.8, 150.7, 145.6, 145.2, 141.3, 141.0, 132.4, 122.1, 122.0, 121.6, 120.9, 116.5, 78.8, 22.7, 20.5.

HRMS (ESI): calculated for $[M+H]^+$: 385.1143, found: 385.1140.

Synthesis of allyl 4-(4-(2-(allyloxy)-4-amino-3-isopropoxyphenyl)-1H-1,2,3-triazol-1-yl)benzoate ***(64)***

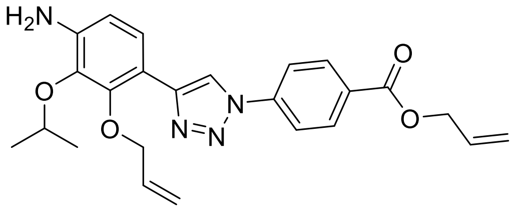

a) 4-(4-(2-hydroxy-3-isopropoxy-4-nitrophenyl)-1*H*-1,2,3-triazol-1-yl)benzoic acid **S30** (70 mg, 0.18 mmol, 1.0 Eq) and K_2CO_3 (101 mg, 4.00 Eq) were suspended in DMF (1.8 mL) and allyl bromide (63 µL, 4.0 Eq) was added. The reaction was stirred

overnight, then the reaction mixture was diluted between MTBE (25 mL) and water (25 mL) and aqueous phase extracted with MTBE (1x25 mL). Solvent was removed u.r.p. and DMF was removed by coevaporation with heptane (3x). Crude product yield: 80 mg, 95%.

b) Modified from[6, 13]: The crude product (80 mg, 1.0 Eq) was suspended in EtOH (2.5 mL) and AcOH (0.35 µL, 35 Eq) in a glass vial flushed with Ar and the solution cooled in ice bath. Zn powder (112 mg, 10.0 Eq) was added in two portions over 15 min and the mixture was stirred at RT while vigourously stirred. The solution was diluted after 1 h between MTBE (25 mL) and sat. $NaHCO_3$ sol. (25 mL). The aqueous phase was extracted again with MTBE (1x25 mL) and the combined organic phases were washed with brine (25 mL) and dried over Na_2SO_4.

The reaction and workup was repeated with following amounts: protected Triazole (110 mg, 237 µmol, 1.00 Eq), EtOH (3.5 mL), AcOH (0.45 mL, 33 Eq), Zn powder (154 mg, 10.0 Eq). Crude products were combined and purified by FCC (solid loading, 75x silica mass, PE/EA, 80/20->70/30). Solvent was removed u.r.p. overnight to give a yield of 143 mg, 76% o2s.

^{1}H NMR (500 MHz, $CDCl_3$): δ = 8.51 (s, 1H), 8.23 (d, J = 8.9, 2H), 7.92 (d, J = 8.4, 1H), 7.87 (d, J = 9.0, 2H), 6.75 (d, J = 8.5, 1H), 6.17 – 6.01 (m, 2H), 5.51 – 5.40 (m, 2H), 5.36 – 5.28 (m, 2H), 4.86 (dt, J = 5.8, 1.4, 2H), 4.64 (hept, J = 6.1, 1H), 4.56 (dt, J = 5.5, 1.5, 2H), 1.36 (d, J = 6.3, 6H).

^{13}C NMR (126 MHz, $CDCl_3$): δ = 165.3, 149.2, 144.8, 140.9, 140.6, 138.7, 134.5, 132.1, 131.6, 129.9, 123.0, 119.6, 118.9, 118.8, 117.4, 115.7, 112.7, 75.5, 72.7, 66.1, 22.9.

HRMS (ESI): calculated for $[M+H]^+$: 435.2027, found: 435.2025.

*Synthesis of tert-butyl 2'-hydroxy-3'-isopropoxy-4'-nitro-[1,1'-biphenyl]-4-carboxylate (**66**)*

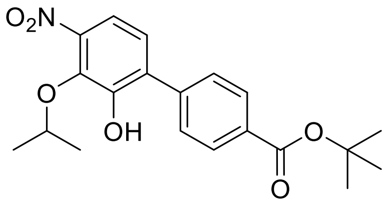

6-bromo-2-isopropoxy-3-nitrophenyl acetate **62** (200 mg, 0.63 mmol, 1.0 Eq), *tert*-butyl 4-(4,4,5,5-tetramethyl-1,3,2-dioxaborolan-2-yl) benzoate (192.0 mg, 0.63 mmol, 1.0 Eq), tetrakis(triphenylphosphine)-palladium(0) (36.0 mg, 0.03 mmol, 0.05 Eq) and potassium carbonate (261 mg, 3.0 Eq) were added to a flask under nitrogen atmosphere. 1,4-dioxane (6.9 mL) and water (2.3 mL) were added and the reaction mixture was degassed with nitrogen. The reaction flask was sealed and stirred at 100°C for 2 h. After completion, HCl (5 mL, 1M) and brine (15 mL) were added to the mixture. The aqueous layer was extracted with EtOAc (3x8 mL). The solvent was removed under reduced pressure. The crude residue was dissolved in THF (1 mL) and water (1 mL) and LiOH*H_2O (75.0 mg, 5 Eq) were added. The reaction was controlled by LCMS. After completion, the reaction was quenched with HCl (5 mL, 1M) and brine (15 mL) and extracted with EtOAc (3x8 mL). The organic solvent was removed under reduced pressure and the crude product was purified by chromatography (PE/ EtOAc). A yellow solid was obtained, yield: 70.4 mg, 30 %.

^{1}H NMR (500 MHz, $CDCl_3$): δ = 8.08 (d, 2H, *J* = 8.4 Hz), 7.66 (d, 2H, *J* = 8.4 Hz), 7.58 (d, 1H, *J* = 8.7 Hz), 7.20 (d, 1H, *J* = 8.7 Hz), 6.41 – 6-39 (m, 1H), 4.39 (hept., 1H, *J* = 6.1 Hz), 1.61 (s, 9H), 1.40 (d, 6H, *J* = 6.1 Hz)

^{13}C NMR (126 MHz, $CDCl_3$): δ = 165.5, 148.7, 141.8, 140.0, 139.3, 132.4, 132.0, 129.7, 129.1, 124.8, 116.9, 81.4, 80.1, 28.4, 22.7

HRMS (ESI): calculated for $[M-H]^+$: 372.1447, found: 372.1453.

*Synthesis of tert-butyl 4'-amino-3'-isopropoxy-2'-(methoxymethoxy)-[1,1'-biphenyl]-4-carboxylate (**67**)*

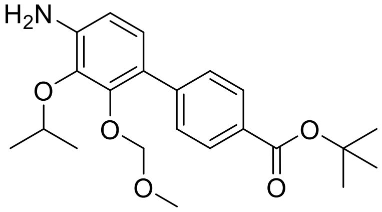

tert-butyl 2'-hydroxy-3'-isopropoxy-4'-nitro-[1,1'-biphenyl]-4-carboxylate **66** (70.0 mg, 0.19 mmol, 1.0 Eq) and DMAP (8.0 mg, 0.35 Eq) were added to a dry flask and further dried under high vacuum. Dry DCM (4.2 mL), Dry THF (1 mL) and DIPEA (66.0 µL, 2 Eq) were added under nitrogen atmosphere and the mixture was cooled down to 0°C. MOM-Br (17.0 µL, 90 % technical grade, 0.19 mmol, 1.0 Eq) was slowly added to the

stirring mixture under nitrogen atmosphere. The reaction warmed up to RT and stirred for 3 h. After completion, HCl (1 mL, 1M) and water (4 mL) were added. The aqueous phase was extracted three times with Et_2O (2 mL). The combined organic phases were concentrated under reduced pressure and dried under high vacuum. The residue was dissolved in THF (0.8 mL) and EtOH (0.65 mL) and cooled down to 0°C. Zinc dust (176 mg, 15.0 Eq) was added. Acetic acid (0.16 mL, 16 Eq) was added to the stirring mixture over 30 min and the mixture was allowed to reach RT. After completion, the zinc dust was filtered off. Saturated $NaHCO_3$ solution (10 mL) was added and the aqueous phase was extracted with EtOAc (3x6 mL). The combined organic phases were concentrated under reduced pressure and dried under high vacuum. An orange to brown oil was obtained, yield: 74.6 mg, >98%.

^{1}H NMR (500 MHz, $CDCl_3$): δ = 7.98 (d, 2H, *J* = 8.5 Hz), 7.56 (d, 2H, *J* = 8.5 Hz), 6.90 (d, 1H, *J* = 8.3 Hz), 6.58 (d, 1H, *J* = 8.3 Hz), 4.88 (s, 2H), 4.61 (hept., 1H, *J* = 6.2 Hz), 3.91 (br s, 2H), 3.01 (s, 3H), 1.61 (s, 9H), 1.35 (d, 6H, *J* = 6.2 Hz)

^{13}C NMR (126 MHz, $CDCl_3$): δ = 166.1, 148.1, 143.6, 142.3, 138.1, 129.8, 129.4, 129.3, 126.0, 125.5, 111.6, 98.8, 81.0, 75.2, 57.3, 28.4, 22.9

HRMS (ESI): calculated for $[M+H]^+$: 388.2124, found: 388.2118.

*Synthesis of tert-butyl 4-(2-(allyloxy)-3-isopropoxy-N-methyl-4-nitrobenzamido)benzoate (**S31**)*

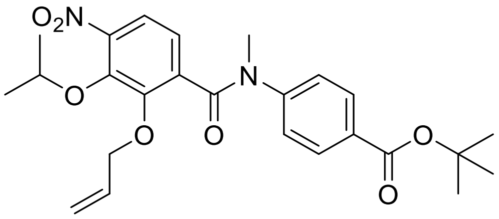

Standard ring D benzoic acid **85** (484 mg, 1.72 mmol, 1.0 Eq) and *tert*-Butyl 4-(methylamino)benzoate (339 mg, 1.64 mmol, 0.95 Eq) were dissolved in DCM (5 mL). The solution was cooled to 0°C and DIPEA (510 µL, 2.93 mmol, 1.70 Eq) and $POCl_3$ (160 µL, 1.72 mmol, 1.00 Eq) were added. The solution was stirred for 2 h at 0°C. A sat. NH_4Cl solution was added and the aq. phase was extracted with DCM. The combined organic phases were dried over $MgSO_4$, filtered and concentrated under

reduced pressure. The crude product was purified by column chromatography (dry load, PE/EtOAc = 4:1) to furnish the title compound (639 mg, 79%) as yellow gum.

R*f* (PE/EtOAc = 4:1) = 0.16

^{1}H NMR (400 MHz, $CDCl_3$): *δ* = 7.79-7.77 (d, *J* = 8.2 Hz, 2H, H_{Ar}), 7.40-7.38 (d, *J* = 8.1 Hz, 1H, H_{Ar}), 7.09-7.07 (d, *J* = 8.1 Hz, 2H, H_{Ar}), 7.03-7.01 (d, *J* = 8.3 Hz, 1H, H_{Ar}), 6.07-5.97 (m, 1H, C<u>H</u>CH_2), 5.40-5.35 (dq, *J* = 1.5, 17.2 Hz, 1H, CHC<u>H</u>$_2$), 5.30-5.27 (dq, *J* = 1.4, 10.4 Hz, 1H, CHC<u>H</u>$_2$), 4.53-4.52 (d, *J* = 4.6 Hz, 2H, OCH_2), 4.29-4.23 (p, *J* = 6.1 Hz, 1H, C<u>H</u>$(CH_3)_2$), 3.49 (s, 3H, NCH_3), 1.54 (s, 9H, $C(CH_3)_3$), 1.01 (bs, 6H, CH(C<u>H</u>$_3)_2$)

^{13}C NMR (101 MHz, $CDCl_3$): *δ* = 166.8 (CO), 164.7 (CO), 149.6 (C_{Ar}), 146.7 (C_{Ar}), 146.4 (C_{Ar}), 144.8 (C_{Ar}), 137.0 (C_{Ar}), 133.2 (<u>C</u>HCH_2), 130.9 (C_{Ar}), 130.1 (C_{Ar}), 126.8 (C_{Ar}), 122.3 (C_{Ar}), 120.0 (C_{Ar}), 118.5 (CH<u>C</u>H$_2$), 81.6 (<u>C</u>$(CH_3)_3$), 77.4 (<u>C</u>H$(CH_3)_2$), 75.2 (OCH_2), 37.1 (NCH_3), 28.3 (C(<u>C</u>H$_3)_3$), 22.0 (CH(<u>C</u>H$_3)_2$)

HRMS (ESI): calculated for $[M+Na]^+$: 493.1951; found: 493.1956.

*Synthesis of tert-butyl 4-(2-(allyloxy)-4-amino-3-isopropoxy-N-methylbenzamido)benzoate (**83**)*

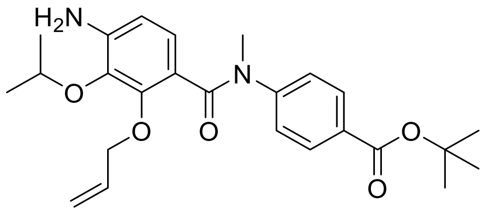

tert-Butyl 4-(2-(allyloxy)-3-isopropoxy-N-methyl-4-nitrobenzamido)benzoate **S31** (587 mg, 1.25 mmol, 1.0 Eq) was dissolved in THF (2.1 mL) and EtOH (1.8 mL). Zinc dust (1.22 g, 18.7 mmol, 15.0 Eq) was added. The mixture was cooled to 0°C and AcOH (1.00 mL, 18.7 mmol, 15.0 Eq) was added dropwise over 1 h. The mixture was warmed to RT and stirred for 1 h. After completion of the reaction Et_2O was added and the reaction was terminated with a sat. $NaHCO_3$ solution. The mixture was filtered and the phases were separated. The aq. phase was extracted with Et_2O (2x). The combined organic phases were washed with brine, dried over $MgSO_4$, filtered and concentrated under reduced pressure to furnish the title compound (533 mg, 97%) as colorless amorphous solid.

R*f* (PE/EtOAc = 2:1) = 0.21

^{1}H NMR (400 MHz, $CDCl_3$): *δ* = 7.77-7.75 (d, *J* = 8.6 Hz, 2H, H_{Ar}), 7.06-7.04 (d, *J* = 8.5 Hz, 2H, H_{Ar}), 6.88-6.86 (d, *J* = 8.2 Hz, 1H, H_{Ar}), 6.51-6.49 (d, *J* = 7.8 Hz, 1H,

H_{Ar}), 6.04-5.94 (m, 1H, CHCH$_2$), 5.34-5.29 (dq, *J* = 1.6, 17.2 Hz, 1H, CHCH$_2$), 5.23-5.19 (dq, *J* = 1.5, 10.4 Hz, 1H, CHCH$_2$), 4.41-4.39 (d, *J* = 5.6 Hz, 1H, OCH$_2$), 4.13-4.07 (p, *J* = 6.0 Hz, 1H, CH(CH$_3$)$_2$), 3.45 (s, 3H, NCH$_3$), 1.54 (s, 9H, C(CH$_3$)$_3$), 1.03-1.01 (d, *J* = 6.0 Hz, 1H, CH(CH$_3$)$_2$)

^{13}C NMR (101 MHz, CDCl$_3$): δ = 169.1 (CO), 165.2 (CO), 148.6 (C_{Ar}), 148.1 (C_{Ar}), 137.9 (C_{Ar}), 134.2 (CHCH$_2$), 131.4 (C_{Ar}), 129.6 (C_{Ar}), 129.4 (C_{Ar}), 126.3 (C_{Ar}), 123.9 (C_{Ar}), 117.3 (CHCH$_2$), 111.9 (C_{Ar}), 81.2 (C(CH$_3$)$_3$), 74.7 (CH(CH$_3$)$_2$), 74.5 (OCH$_2$), 37.3 (NCH$_3$), 28.3 (C(CH$_3$)$_3$), 22.3 (CH(CH$_3$)$_2$)

HRMS (ESI): m/z calculated for $C_{25}H_{32}N_2O_5Na$ [M+Na]$^+$: 463.2209; found: 463.2206.

*Synthesis of methyl 2-(2-(allyloxy)-3-isopropoxy-4-nitrophenyl)quinazoline-6-carboxylate (**77**)*

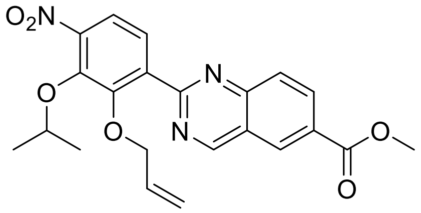

Methyl 4-amino-3-formylbenzoate **76** (50 mg, 0.28 mmol, 1.0 Eq) and 2-(allyloxy)-3-isopropoxy-4-nitrobenzaldehyde **52** (93 mg, 0.35 mmol, 1.3 Eq) were added to a flask. Water (5.0 mL) and ammonium acetate (220 mg, 10 eq) were added and the mixture was heated to 75°C. After completion, the solid was filtered off and dissolved in EtOAc. The crude product was purified by flash chromatography (PE/ EtOAc). An orange solid was obtained, yield: 49.3 mg, 42 %.

^{1}H NMR (500 MHz, Aceton): δ = 9.87 (d, 1H, *J* = 0.8 Hz), 8.89 (dd, 1H, *J* = 0.6 Hz, 1.9 Hz), 8.55 (dd, 1H, *J* = 1.9 Hz, 8.8 Hz), 8.17 (dt, 1H, *J* = 0.7 Hz, 8.8 Hz), 7.85 (d, 1H, *J* = 8.6 Hz), 7.73 (d, 1H, *J* = 8.6 Hz), 6.08 (ddt, 1H, *J* = 5.6 Hz, 10.5 Hz, 17.2 Hz), 5.34 (dq, 1H, *J* = 1.7 Hz, 17.2 Hz), 5.16 (dq, 1H, *J* = 1.3 Hz, 10.5 Hz), 4.83 – 4.76 (m, 3H), 4.01 (s, 3H), 1.30 (d, 6H, *J* = 6.2 Hz)

^{13}C NMR (126 MHz, Aceton): δ = 166.2, 163.1, 162.7, 153.4, 153.0, 139.3, 134.9, 134.5, 131.3, 130.6, 129.8, 127.1, 123.7, 119.8, 117.7, 78.0, 75.5, 53.0, 22.6

HRMS (ESI): calculated from [M+H]$^+$: 424.1509, found: 424.1503.

*Synthesis of methyl 2-(2-(allyloxy)-4-amino-3-isopropoxyphenyl)quinazoline-6-carboxylate (**78**)*

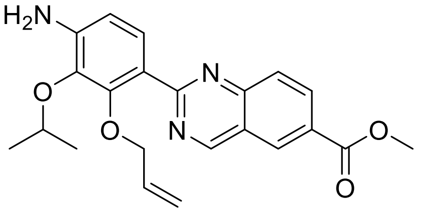

Methyl 2-(2-(allyloxy)-3-isopropoxy-4-nitrophenyl)quinazoline-6-carboxylate **77** (212 mg, 0.5 mmol, 1 Eq) was dissolved in EtOH (3.0 mL) and cooled down to 0°C. The reaction was sealed with a septum. Sodium dithionite (436 mg, 5 Eq) was dissolved in water (1.2 mL) and added dropwise while maintaining 0°C. The reaction was allowed to reach RT. After 3 h, another portion sodium dithionite (436 mg, 5 Eq) was added. The reaction was stirred overnight. After completion, the solvent was removed under reduced pressure. The crude product was purified by RP flash chromatography. A yellow to orange solid was obtained, yield: 28.0 mg, 14 %.

^{1}H NMR (500 MHz, MeOH): δ = 8.18 (d, 1H, *J* = 1.8 Hz), 8.08 (dd, 1H, *J* = 1.9 Hz, 8.4 Hz), 7.38 (d, 1H, *J* = 8.7 Hz), 7.30 (d, 1H, *J* = 8.4 Hz), 6.68 (d, 1H, *J* = 8.7 Hz), 6.03 (ddt, 1H, *J* = 6.1 Hz, 10.4 Hz, 16.8 Hz), 5.64 (s, 1H), 5.27 (dq, 1H, *J* = 1.4 Hz, 17.2 Hz), 5.16 – 5.13 (m, 1H), 4.74 – 4.67 (m, 3H), 4.53 – 4.48 (m, 1H), 3.92 (s, 3H), 1.43 (d, 3H, *J* = 6.2 Hz), 1.26 (d, 3H, *J* = 6.1 Hz)

^{13}C NMR (126 MHz, MeOH): δ = 188.4, 176.4, 171.9, 171.0, 153.8, 153.4, 149.9, 147.3, 146.8, 143.8, 142.4, 132.2, 131.6, 130.4, 122.6, 118.8, 81.4, 80.7, 72.3, 53.5, 18.6, 17.5

HRMS (ESI): calculated for $[M+H]^+$: 394.1767, found: 394.1760.

*Synthesis of methyl 2-(2-(allyloxy)-3-isopropoxy-4-nitrophenyl)-benzo[d]imidazole-5-carboxylate (isomeric mixture) (**79**)*

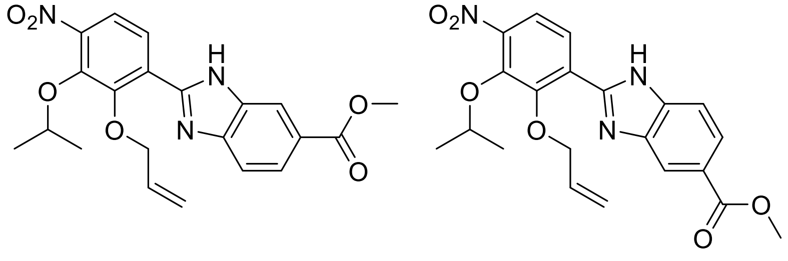

2-(allyloxy)-3-isopropoxy-4-nitrobenzaldehyde **52** (150 mg, 0.57 mmol, 1.0 Eq) and methyl 3,4-diaminobenzoate **81** (94.0 mg, 0.57 mmol, 1.0 Eq) were dissolved in MeOH (1 mL) and THF (0.6 mL) and stirred under open atmosphere. After completion, the solvent was removed under reduced pressure. The crude product purified by chromatography. An orange solid was obtained, yield: 154.3 mg (66 %)

^{1}H NMR (500 MHz, $CDCl_3$): δ = 10.94 (br s, 1H), 8.41 (d, 1H, J = 8.8 Hz), 8.05 (dd, 1H, J = 1.6 Hz, 8.6 Hz), 7.71 (d, 1H, J = 8.8 Hz), 6.16 – 6.07 (m, 1H), 5.52 (dq, 1H, J = 1.4 Hz, 17.1 Hz), 5.43 (dd, 1H, J = 1.0 Hz, 10.4 Hz), 4.81 – 4.78 (m, 2H), 4.67 (hept., 1H, J = 6.2 Hz), 3.97 (s, 3H), 1.37 (d, 6H, J = 6.2 Hz).

^{13}C NMR (176 MHz, $CDCl_3$): δ = 167.4, 151.2, 146.8, 145.4, 132.3, 124.4, 120.9, 120.8, 78.9, 75.1, 52.4, 22.5.

*Synthesis of 1-allyl 5-methyl 2-(2-(allyloxy)-3-isopropoxy-4-nitrophenyl)-benzo[d]imidazole-1,5-dicarboxylate and 1-allyl 6-methyl 2-(2-(allyloxy)-3-isopropoxy-4-nitrophenyl)-1H-benzo[d]imidazole-1,6-dicarboxylate (**S32**)*

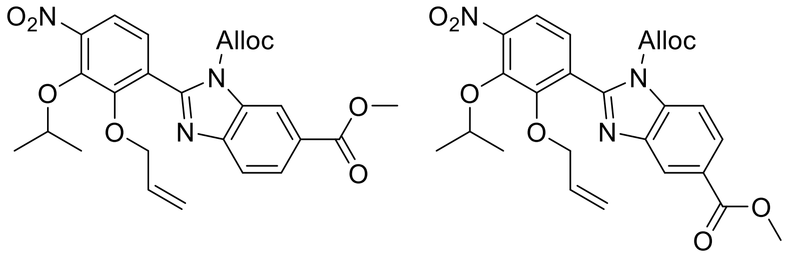

Methyl 2-(2-(allyloxy)-3-isopropoxy-4-nitrophenyl)-benzo[d]imidazole-5-carboxylate (isomeric mixture) **79** (150 mg, 0.36 mmol, 1.0 Eq) was added to a dry flask and further dried under high vacuum. Dry DCM (1.1 mL) and dry DIPEA (0.13 mL, 0.75 mmol, 2.05 Eq) were added. The solution was cooled down to 0 °C. Allyl chloroformate (43 µL, 0.4 mmol, 1.1 Eq) was slowly added under argon atmosphere. The reaction was warmed up to room temperature and stirred overnight. After completion, the reaction was quenched with HCl (1 M, 1 mL) and brine (7 mL). The product was extracted with EtOAc (3x4 mL). The combined organic layers were concentrated under reduced pressure. The crude product was purified by chromatography. A yellow oil was obtained, 130.5 mg (72 %).

^{1}H NMR (500 MHz, $CDCl_3$): δ = 8.71 (dd, 0.5 H, J = 0.6 Hz, 1.6 Hz), 8.52 (dd, 0.5 H, J = 0.6 Hz, 1.6 Hz), 8.17 (dd, 0.5 H, J = 1.6 Hz, 8.7 Hz), 8.14 (dd, 0.5 H, J = 1.6 Hz, 8.4

Hz), 8.04 (dd, 0.5 H, J = 0.6 Hz, 8.7 Hz), 7.86 (dd, 0.5 H, J = 0.6 Hz, 8.5 Hz), 7.64 (dd, 1H, J = 0.5 Hz, 8.5 Hz), 7.49 (dd, 1H, J = 3.2 Hz, 8.5 Hz), 5.95 – 5.84 (m, 1H), 5.63 – 5.54 (m, 1H), 5.33 – 5.30 (m, 1H), 5.29 – 5.24 (m, 1H), 5.05 – 4.99 (m, 1H), 4.99 – 4.95 (m, 1H), 4.89 – 4.80 (m, 2H), 4.68 – 4.62 (m, 1H), 4.44 – 4.32 (m, 2H), 3.99 – 3.98 (m, 3H), 1.36 – 1.31 (m, 6H)

*Synthesis of 1-allyl 5-methyl 2-(2-(allyloxy)-4-amino-3-isopropoxyphenyl)-1H-benzo[d]imidazole-1,5-dicarboxylate and 1-allyl 6-methyl 2-(2-(allyloxy)-4-amino-3-isopropoxyphenyl)-1H-benzo[d]imidazole-1,6-dicarboxylate (**80**)*

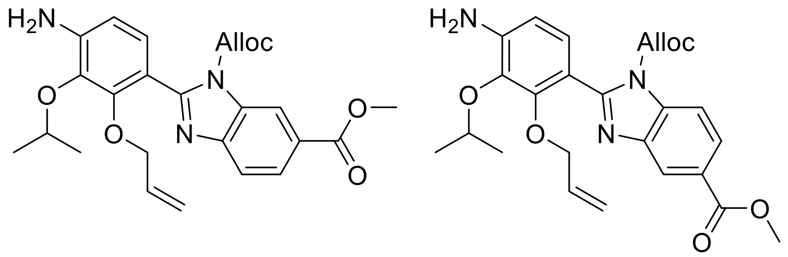

A mixture **S32** containing 1-allyl 5-methyl 2-(2-(allyloxy)-3-isopropoxy-4-nitrophenyl)-1H-benzo[d]imidazole-1,5-dicarboxylate and 1-allyl 6-methyl 2-(2-(allyloxy)-3-isopropoxy-4-nitrophenyl)-1H-benzo[d]imidazole-1,6-dicarboxylate (125 mg, 0.25 mmol, 1 Eq) was dissolved in THF (1.1 mL) and EtOH (0.9 mL) and cooled to 0°C. Zinc dust (240 mg, 3.7 mmol, 15 Eq) was added. AcOH (0.21 mL, 3.7 mmol, 15 Eq) was added to the stirring mixture over 30 min and the mixture was warmed up to RT. After completion, the zinc dust was filtered off. Saturated $NaHCO_3$ solution (10 mL) was added to the solution and it was extracted with EtOAc (3x5 mL). The crude product was of sufficient purity for further reactions. A yellow to orange foam was obtained, yield: 120.8 mg (quant.).

^{1}H NMR (500 MHz, $CDCl_3$): δ = 8.65 (dd, 0.5 H, J = 0.6 Hz, 1.6 Hz), 8.48 (br s, 0.5 H), 8.11 – 8.08 (m, 1H), 7.98 (dd, 0.5 H, J = 0.6 Hz, 8.6 Hz), 7.83 (d, 0.5 H, J = 8.3 Hz), 7.41 – 7.34 (m, 1H), 6.63 (dd, 1H, J = 1.3 Hz, 8.3 Hz), 5.87 (dddt, 1H, J = 5.5 Hz, 10.8 Hz, 14.1 Hz, 17.2 Hz), 5.64 (ddtd, 1H, J = 3.5 Hz, 6.0 Hz, 10.3 Hz, 17.1 Hz), 5.25 – 5.15 (m, 2H), 5.03 (ddq, 1H, J = 1.5 Hz, 6.7 Hz, 17.2 Hz), 4.97 – 4.92 (m, 1H), 4.82 (dd, 2H), 4.57 – 4.51 (m, 1H), 4.27 (br s, 2H), 3.96 (d, 3H, J = 1.9 Hz), 2.04 (s, 3H), 1.33 (d, 6H, J = 6.1 Hz)

CD-fragments

Synthesis of 2-(allyloxy)-3-methoxy-4-(4-nitrobenzamido)benzoic acid ***(S33)***

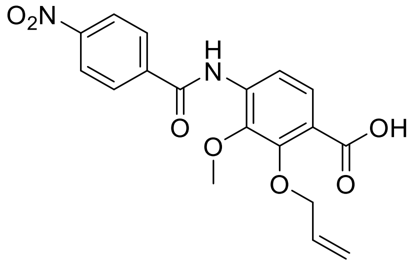

tert-butyl 2-(allyloxy)-4-amino-3-methoxybenzoate **34** (400 mg, 1.43 mmol, 1.00 Eq) and 4-nitrobenzoyl chloride (279 mg, 1.05 Eq) were solved in dry DCM (7.0 mL) under Argon atmosphere and dry pyridine (0.23 mL, 2.0 Eq) was added. After 1 h, solvents were removed from the reaction mixture u.r.p. and by coevaporation with EA/heptane to give a yellowish crude product. The crude product was secured under Ar, solved in HCl/dioxane (4 M, 10 mL) and the reaction was stirred for 40 min. The reaction mixture was quenched by diluting in water (70 mL) and extracting with EA (1x50 mL, 2x30 mL). The combined organic phases were washed with brine (10 mL) and water (10 mL), dried over Na_2SO_4 and solvents were removed u.r.p.. The residue was solved again in HCl/dioxane (4 M, 6.0 mL) and the reaction was stirred for 1 h. Solvents were removed u.r.p. to obtain a pale-yellow amorphous solid (553 mg, NMR: >98%) containing residual amounts of 4-nitrobenzoic acid (~8 mol%).

^{1}H NMR (700 MHz, DMSO): δ = 12.86 (s, 1H), 10.11 (s, 1H, H-NCO), 8.37 (d, J = 8.9, 2H), 8.17 (d, J = 8.9, 2H), 7.74 (d, J = 8.6, 1H), 7.50 (d, J = 8.5, 1H), 6.09 (ddt, J = 17.3, 10.5, 5.6, 1H), 5.39 (dq, J = 17.2, 1.7, 1H), 5.23 (dq, J = 10.4, 1.3, 1H), 4.53 (dt, J = 5.6, 1.5, 2H), 3.85 (s, 3H).

^{13}C NMR (176 MHz, DMSO): δ = 166.5, 164.3, 151.4, 149.3, 145.8, 140.0, 135.1, 134.2, 129.4, 125.3, 123.8, 123.6, 118.8, 117.5, 74.5, 60.9.

HRMS (ESI): calculated for [M+H]+: 373.1031, found: 373.1033.

CDE-fragments

General procedure **A** (coupling of 4-nitrobenzoyl chloride with the DE-fragments):

Modified from[6]: The DE-fragment and 4-nitrobenzoyl chloride were solved in dry DCM under Argon atmosphere. Then dry pyridine was added and the solution was stirred at RT for the given amount of time while being screened via TLC or LCMS. The mixture was diluted between HCl (0.1-0.5 M, 15-30 mL) and DCM (1x15-30 mL) and the aqueous phase was extracted with DCM (1-2x15-30 mL). The combined organic phases were washed with H_2O (1x5-10 mL) when required and dried with Na_2SO_4. The solvent was removed u.r.p. to give the crude intermediate that was directly used in the subsequent nitro reduction. If a workup was omitted, only the solvent was removed u.r.p..

Synthesis of tert-butyl 4-(2-(allyloxy)-4-(4-aminobenzamido)-5-isopropoxybenzamido) benzoate ***(S34)***

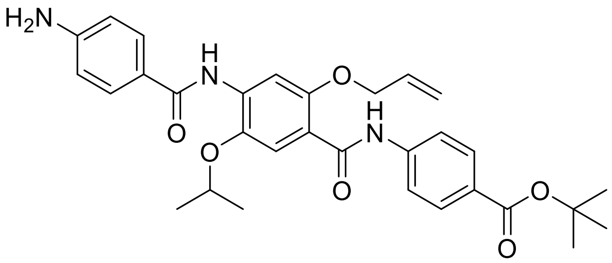

a) The nitro-CDE-fragment was obtained according to general procedure A, involving the reaction of the DE-fragment **S19** (23 mg, 54 µmol, 1.0 Eq), 4-nitrobenzoyl chloride (10.6 mg, 1.05 Eq) and dry pyridine (5.3 µL, 65 µmol, 1.2 Eq) in dry DCM (1.0 mL) for 2 h.

Deviating from the general aqueous workup, this workup was performed: The mixture was diluted between HCl (0.1 M, 15 mL) and DCM (1x15 mL), the aqueous phase was extracted with DCM (1x15 mL) and washed with sat. $NaHCO_3$ solution (1x20 mL). Combined organic phases were dried with Na_2SO_4 and the solvent was removed u.r.p. to give a yellow solid that was directly used in the next step.

b) Modified from[6, 13]: The crude product was solved in THF/EtOH (1.0 mL+0.5 mL) and AcOH (47 µL, 15 Eq) and Zn powder (53 mg, 15 Eq) were added in two portions directly and after 10 min. The reaction was stirred 2 h before the suspension was diluted between MTBE (15 mL) and sat. $NaHCO_3$ sol. (15 mL). The aqueous phase

was extracted with MTBE (1x15 mL) and combined organic phases were washed with brine (1x20 mL), dried over Na_2SO_4 and solvents were removed u.r.p.. The pale-yellow colored solid was purified by FCC (solid loading, 100x reactants mass, PE/EA, 75/25) and solvents were removed u.r.p. to give a yellowish oil, yield: 25 mg, 84% o2s, purity: ~90 wt% (impurity: low boiling residues from petrol ether). The product was dried further at high vacuum overnight.

^{1}H NMR (500 MHz, $CDCl_3$) δ = 10.36 (s, 1H, H-NCO), 8.76 (s, 1H, H-NCO), 8.51 (s, 1H, H-$C_{Ar,D}$), 7.97 (d, J = 8.9, 2H), 7.84 (s, 1H, H-$C_{Ar,D}$), 7.72 (d, J = 8.9, 2H), 7.70 (d, J = 9.0, 2H), 6.81 (d, J = 8.5, 2H), 6.21 (ddt, J = 17.2, 10.5, 6.0, 1H), 5.57 (dq, J = 17.2, 1.4, 1H), 5.48 (dq, J = 10.2, 1.0, 1H), 4.79 (d, J = 6.0, 2H), 4.75 – 4.68 (m, 1H), 1.59 (s, 9H), 1.41 (d, J = 6.1, 6H).

^{13}C NMR (126 MHz, $CDCl_3$) δ 165.6, 165.2, 163.3, 151.5, 149.3, 142.6, 140.8, 133.9, 131.9, 130.8, 129.0, 127.2, 124.7, 120.7, 119.1, 115.5, 115.4, 115.2, 104.7, 80.9, 72.4, 71.2, 28.4, 22.4.

HRMS (ESI): calculated for $[M+H]^+$: 546.2599, found: 546.2598.

Synthesis of tert-butyl 4-(4-(4-aminobenzamido)-2-fluoro-3-isopropoxybenzamido) benzoate ***(S35)***

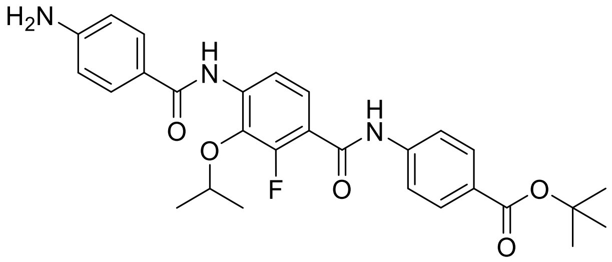

a) The nitro-CDE-fragment was obtained according to general procedure A, involving the reaction of the DE-fragment **S20** (47.6 mg, 0.123 mmol, 1.00 Eq), 4-nitrobenzoyl chloride (24.2 mg, 1.07 Eq) and dry pyridine (20 µL, 2.0 Eq) in dry DCM (2.5 mL) for 3 h. An aqueous workup was omitted.

b) Modified from[6, 13]: The crude product was suspended in a THF/EtOH-mixture (1.4 mL each) under Argon atmosphere and Zn powder (80 mg, 10 Eq) and AcOH (0.14 mL, 20 Eq) were added. The reaction was stirred vigourously and Zn (2x40 mg, 2x5.0 Eq) and AcOH (2x70 µL, 2x10 Eq) were added again after 1 h and 2.5 h. After

3.5 h and LCMS control the mixture was diluted between EA/sat. $NaHCO_3$ sol. (30 mL each) and the aqueous phase was extracted with EA (2x15 mL). The organic phase was washed with brine (10 mL) and dried over Na_2SO_4. The solvents were removed u.r.p. and the material was purified by FCC (solid loading, 75x theoretical product mass, PE/EA, 60/40). The product was obtained as a green-colorless gum (60.5 mg, 97% o2s, NMR: 88% o2s). For the next reaction the product was coevaporated with DCM/heptane to give an off-colorless solid.

^{1}H NMR (500 MHz, Acetone): δ = 9.61 (d, J = 4.3, 1H, H-NCO), 8.77 (s, 1H, H-NCO), 8.39 (dd, J = 8.8, 1.4, 1H), 7.98 (d, J = 8.9, 2H), 7.91 (d, J = 9.0, 2H), 7.75 (d, J = 8.9, 2H), 7.56 (dd, J = 8.7, 7.6, 1H), 6.79 (d, J = 8.7, 2H), 5.44 (s, 2H, H_2N), 4.65 – 4.56 (m, 1H), 1.59 (s, 9H), 1.41 (dd, J = 6.3, 0.8, 6H).

^{13}C NMR (126 MHz, Acetone): δ = 165.6, 165.3, 163.0, 153.8 (d, J = 248), 153.6, 143.8, 138.4 (d, J = 5.0), 135.4 (d, J = 14.1), 131.1 (C_{Ar}-H), 129.8 (C_{Ar}-H), 128.1, 125.5 (d, J = 2.9), 122.2, 119.9 (C_{Ar}-H), 119.7 (d, J = 12.0), 115.8 (d, J = 4.0), 114.3, 81.0, 78.4 (d, J = 5.2), 28.3, 22.9.

^{19}F NMR (471 MHz, Acetone): δ = -130.7.

HRMS (ESI): calculated for $[M+H]^+$: 508.2242, found: 508.2242.

Synthesis of tert-butyl 4-(4-(4-aminobenzamido)-2-chloro-3-isopropoxybenzamido) benzoate ***(93)***

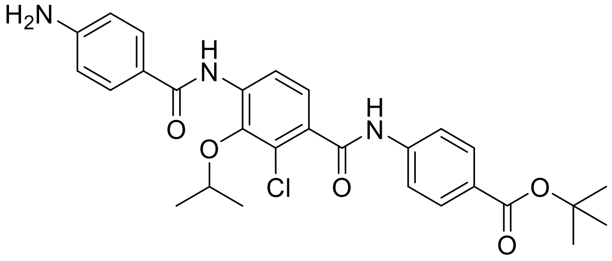

a) The nitro-CDE-fragment was obtained according to general procedure A, involving the reaction of the DE-fragment **S21** (76.8 mg, 0.190 mmol, 1.00 Eq), 4-nitrobenzoyl chloride (37.0 mg, 1.05 Eq) and dry pyridine (30.6 µL, 2.0 Eq) in dry DCM (3.0 mL) for 1-2 h. An aqueous workup was omitted and residual pyridine was coevaporated with acetone and toluene.

b) Modified from[6, 13]: The crude product was suspended in a THF/EtOH-mixture (2.0 mL each) and Zn powder (124 mg, 10.0 Eq) and AcOH (0.22 mL, 20 Eq) were added. The reaction was stirred vigourously and Zn (2x62 mg, 2x5.0 Eq) and AcOH (2x0.11 mL, 2x10 Eq) were added again after 1 h and 2.5 h. After 3.5 h and LCMS control the mixture was diluted between EA/sat. $NaHCO_3$ sol. (40 mL each) and the aqueous phase was extracted with EA (2x20 mL). The combined organic phases were washed with brine (10 mL) and dried over Na_2SO_4. Solvents were removed u.r.p. and the material was purified by FCC (solid loading, 75x theoretical product mass, PE/EA, 60/40->50/50). The product was obtained as green-colorless gum (99.2 mg, 100% o2s, NMR-corrected yield: 90.5 mg, 91%). For the next reaction the product was coevaporated with DCM/heptane to give an off-colorless solid.

^{1}H NMR (500 MHz, Acetone): δ = 9.84 (s, 1H, H-NCO), 8.75 (s, 1H, H-NCO), 8.42 (d, J = 8.4, 1H), 7.98 (d, J = 8.9, 2H), 7.92 (d, J = 8.9, 2H), 7.79 (d, J = 8.9, 2H), 7.39 (d, J = 8.4, 1H), 6.79 (d, J = 8.7, 2H), 5.43 (s, 2H, H_2N), 4.60 (hept, J = 6.2, 1H), 1.59 (s, 9H), 1.37 (d, J = 6.1, 6H).

^{13}C NMR (126 MHz, Acetone): δ = 165.8, 165.6, 165.3, 153.5, 144.6, 143.9, 137.1, 133.1, 131.1, 129.9, 128.0, 125.4, 124.8, 122.2, 119.7, 119.6, 114.3, 81.0, 78.0, 28.3, 22.7.

HRMS (ESI): calculated for $[M+H]^+$: 524.1947, found: 524.1947.

Synthesis of tert-butyl 4-(4-(4-aminobenzamido)-2-bromo-3-isopropoxybenzamido) benzoate ***(S36)***

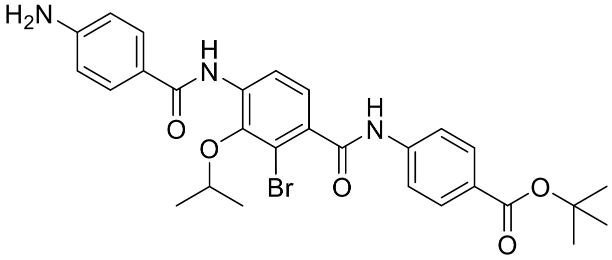

a) The nitro-CDE-fragment was obtained according to general procedure A, involving the reaction of the DE-fragment **S22** (43 mg, 95 µmol, 1.0 Eq), 4-nitrobenzoyl chloride (19.3 mg, 1.09 Eq) and dry pyridine (15.4 µL, 2.00 Eq) in dry DCM (2.0 mL) for 3.0 h. An aqueous workup was omitted.

b) Modified from[6, 13]: The crude product was suspended in a THF/EtOH-mixture (1.0 mL each) under Argon atmosphere and Zn powder (62 mg, 10 Eq) and AcOH (0.11 mL, 20 Eq) were added. The reaction was stirred vigourously and Zn (2x31 mg, 2x5.0 Eq) and AcOH (2x55 µL, 2x10 Eq) were added again after 1 h and 2.5 h. After 3.5 h and LCMS control the mixture was diluted between EA/sat. $NaHCO_3$ sol. (30 mL each) and the aqueous phase was extracted with EA (2x15 mL). The combined organic phases were washed with brine (10 mL) and dried over Na_2SO_4. The material was purified by FCC (solid loading, 75x theoretical product mass, PE/EA, 60/40) to give a green-colorless gum (46.3 mg, 85% o2s, purity: 82 mol% (NMR)) that was found by LCMS to be contaminated with a debrominated byproduct (NMR-signals not listed here).

^{1}H NMR (700 MHz, CD_3CN): δ = 8.87 (s, 1H, H-NCO), 8.59 (s, 1H, H-NCO), 8.40 (d, J = 8.4, 1H), 7.99 – 7.94 (m, 2H), 7.75 (d, J = 8.6, 2H), 7.72 (d, J = 8.6, 2H), 7.31 (d, J = 8.4, 1H), 6.73 (d, J = 8.6, 2H), 4.76 (s, 2H), 4.68 – 4.61 (m, 1H), 1.57 (d, J = 1.6, 9H), 1.36 (d, J = 6.2, 6H).

^{13}C NMR (176 MHz, CD_3CN): δ = 167.0, 166.0, 165.7, 153.2, 146.0, 143.5, 137.0, 131.3, 130.0, 128.5, 125.0, 122.7, 120.8, 120.4, 119.8, 115.3, 114.5, 81.6, 78.5, 28.4, 22.6.

HRMS (ESI): calculated for $[M+H]^+$: 568.1469/ 570.1421, found: 554.1442/ 570.1426.

Synthesis of tert-butyl 4-(7-(4-aminobenzamido)-8-isopropoxy-2-methyl-4-oxoquinazolin-3(4H)-yl)benzoate ***(71)***

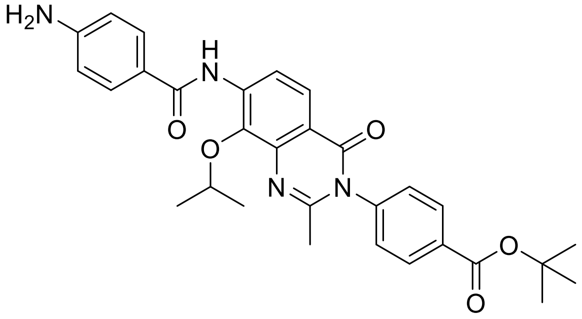

a) The nitro-CDE-fragment was obtained according to general procedure A, involving the reaction of the DE-fragment **S23** (10 mg, 23 µmol, 1.0 Eq), 4-nitrobenzoyl chloride (4.5 mg, 1.05 Eq) and dry pyridine (2.9 µL, 1.6 Eq) in dry DCM (0.5 mL) for 2.0 h.

b) Modified from[6, 13]: The crude material was solved in a EtOH/THF mixture (0.3 mL each) and AcOH (33 µL, 25 Eq) and Zn dust (11 mg, 7.5 Eq) were added. The reaction was screened by TLC and LCMS and after 1 h Zn dust (11 mg, 7.5 Eq) was added again and the reaction was stirred overnight. The next day THF (0.5 mL) and Zn dust (11 mg, 7.5 Eq) were added again and the reaction was stirred for 1 h before adding AcOH (33 µL, 25 Eq). To ensure full turnover Zn dust (11 mg, 7.5 Eq) and AcOH (13 µL, 10 Eq) were added again 1.5 h after the last addition and the reaction was stirred for additional 15 min. The reaction solution was diluted between DCM/sat. $NaHCO_3$ sol. (15 mL each) and the aqueous phase was extracted with DCM (3x10 mL). The combined organic phases were dried over Na_2SO_4 and solvents were removed u.r.p. . The crude material was purified by FCC (solid loading, 100x theoretical product mass, PE/EA, 60/40) to get a pale-yellow gum, yield: 6.2 mg, 51% o2s, purity: ~90% (NMR).

^{1}H NMR (700 MHz, CD_3CN): δ = 8.95 (s, 1H, H-NCO), 8.62 (d, J = 8.8, 1H, H-$C_{Ar,D}$), 8.14 (d, J = 8.1, 2H, H-C_{Ar}), 7.89 (d, J = 8.8, 1H, H-$C_{Ar,D}$), 7.73 (d, J = 8.6, 2H, H-C_{Ar}), 7.46 (d, J = 8.1, 2H, H-C_{Ar}), 6.75 (d, J = 8.7, 2H, H-C_{Ar}), 5.24 – 5.18 (m, 1H, H-C_{iPr}O), 4.76 (s, 2H, H_2N), 2.16 (s, 3H, H_3C), 1.61 (s, 9H, $(H_3C)_3$), 1.41 (d, J = 6.1, 6H, $(H_3C)_2C$).

^{13}C NMR (HSQC, HMBC, 176 MHz, CD_3CN): δ = 165.7 (C=O), 165.6 (C=O), 162.5 ($C_{Ar,D}$-$\underline{C}$=O), 154.2 ($\underline{C}$(=N)(N)CH_3), 153.1 (C_{Ar}-NH_2), 143.1 (C_{Ar}), 141.7 ($C_{Ar,D}$-N), 141.0 ($C_{Ar,D}$-OiPr), 138.9 ($C_{Ar,D}$-N), 133.7 (C_{Ar}), 131.5 (C_{Ar}-H), 129.8 (C_{Ar}-H), 129.6 (C_{Ar}-H), 123.1 (C_{Ar}), 122.3 ($C_{Ar,D}$-H), 118.5 ($C_{Ar,D}$-H), 117.9 ($C_{Ar,D}$-CO), 114.5 (C_{Ar}-H), 82.4 ($\underline{C}(CH_3)_3$), 78.2 ($\underline{C}(CH_3)_2$), 28.2 ($C(\underline{C}H_3)_3$), 24.8 (CH_3), 22.8 ($(\underline{C}H_3)_2C$).

HRMS (ESI): calculated for $[M+H]^+$: 529.2445, found: 529.2442.

Synthesis of tert-butyl 4-(7-(4-aminobenzamido)-8-isopropoxy-4-oxoquinazolin-3(4H)-yl) benzoate ***(75)***

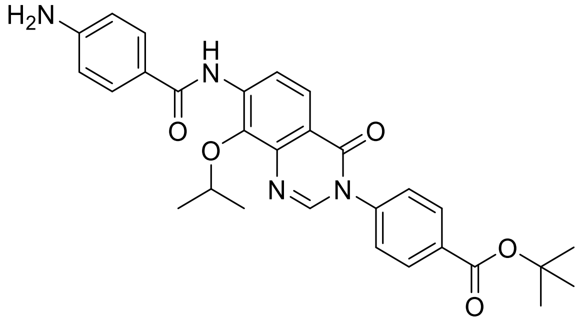

a) The nitro-CDE-fragment was obtained according to general procedure A, involving the reaction of the DE-fragment **74** (28.3 mg, 71.6 µmol, 1.00 Eq), 4-nitrobenzoyl chloride (17.8 mg, 1.35 Eq) and dry pyridine (12 µL, 2.0 Eq) in dry DCM (1.5 mL) overnight.

b) Modified from[6, 13]: The crude material was suspended in dry THF/EtOH (1.0 mL each) and AcOH (82 µL, 20 Eq) was added. Then Zn dust (47 mg, 10 Eq) was added and the reaction was stirred vigourously while being screened by LCMS. AcOH (82 µL, 20 Eq) and Zn dust (47 mg, 10 Eq) were added again after 75 min. After 2 h reaction the mixture was diluted between DCM/sat. $NaHCO_3$ sol. (20 mL each) and the organic phase was extracted with DCM (2x15 mL). The combined organic phases were dried over Na_2SO_4 and the solvent was removed u.r.p.. The material was purified by FCC (solid loading, 100x theoretical product mass, PE/Acetone, 70/30) to give a colorless solid after removing solvents u.r.p. and by coevaporation with heptane. Yield: 29.1 mg, 79%.

^{1}H NMR (700 MHz, CD_3CN): δ = 8.96 (s, 1H, H-NCO), 8.70 (d, J = 8.8, 1H, H-$C_{Ar,D}$), 8.13 (d, J = 8.4, 2H, H-C_{Ar}), 8.12 (s, 1H, H-C(=N)(N)), 8.00 (d, J = 8.7, 1H, H-$C_{Ar,D}$), 7.74 (d, J = 8.6, 2H, H-C_{Ar}), 7.58 (d, J = 8.4, 2H, H-C_{Ar}), 6.75 (d, J = 8.7, 2H, H-C_{Ar}), 5.19 (hept, J = 6.0, 1H, H-C_{iPr}O), 4.77 (s, 2H, H_2N), 1.60 (d, J = 1.4, 9H, $(H_3C)_3$), 1.40 (d, J = 6.1, 6H, $(H_3C)_2C$).

^{13}C NMR (HSQC, HMBC, 176 MHz, CD_3CN): δ = 165.8 (C=O), 165.6 (C=O), 161.1 ($C_{Ar,D}$-C=O), 153.2 (C_{Ar}-NH_2), 146.1 (H-C(=N)(N)), 142.5 (C_{Ar}), 142.2 ($C_{Ar,D}$-N), 141.5 ($C_{Ar,D}$-OiPr), 139.3 ($C_{Ar,D}$-N), 133.3 (C_{Ar}), 131.1 (C_{Ar}-H), 130.0 (C_{Ar}-H), 128.3 (C_{Ar}-H), 123.0 (C_{Ar}), 122.6 ($C_{Ar,D}$-H), 119.6 ($C_{Ar,D}$-H), 119.3 ($C_{Ar,D}$), 114.5 (C_{Ar}-H), 82.4 ($\underline{C}(CH_3)_3$), 78.4 ($\underline{C}(CH_3)_2$), 28.3 ($C(\underline{C}H_3)_3$), 22.9 ($(\underline{C}H_3)_2C$).

HRMS (ESI): calculated for $[M+H]^+$: 515.2289, found: 515.2288.

*Synthesis of tert-butyl 4-(2-amino-4-(4-aminobenzamido)-3-isopropoxybenzamido) benzoate **(S37)***

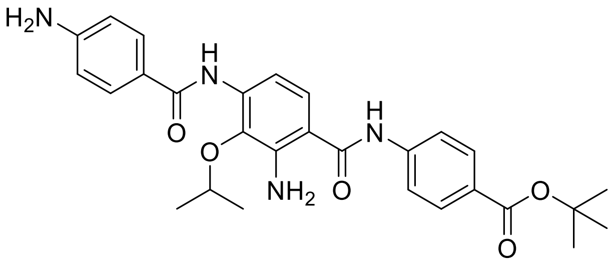

a) The nitro-CDE-fragment was obtained according to general procedure A, involving the reaction of the DE-fragment **S24** (30 mg, 78 µmol, 1.0 Eq), 4-nitrobenzoyl chloride (7.2+7.9 mg, 0.50+0.55 Eq, directly and after 30 min) and dry pyridine (19 µL, 3.0 Eq) in dry DCM (0.80 mL) for 1 h 15 min. EA was used as extraction solvent.

b) Modified from[6, 13]: The crude material was suspended in a THF/EtOH mixture (0.60 mL each) and Zn dust (51 mg, 10 Eq) was added while stirring the reaction vigourously. Then AcOH (2x45 µL, 2x10 Eq) was added directly and after 30 min while the reaction was screened by LCMS. After 2 h Zn dust (25 mg, 5.0 Eq) and AcOH (45 µL, 10 Eq) were added again. After 3 h the reaction mixture was put in -70°C freezer overnight before the reaction was warmed to RT, AcOH (45 µL, 10 Eq) was added again and the reaction was stirred vigourously for 45 min. The mixture was then diluted between EA/sat. $NaHCO_3$ sol. (25 mL each) and the aqueous phase was extracted with EA (2x20 mL) before washing the combined organic phases with brine (5 mL) and drying over Na_2SO_4. The crude material was purified by FCC (solid loading, 100x theoretical product mass, cyclohexane/EA, 70/30->50/50) to give an almost colorless solid after solvent removal and coevaporation with cyclohexane (yield: 29.2 mg, 74% o2s, NMR: 67% o2s).

^{1}H NMR (700 MHz, THF): δ = 9.37 (s, 1H, H-NCO), 8.55 (s, 1H, H-NCO), 7.91 (d, J = 8.7, 2H), 7.83 – 7.79 (m, 3H), 7.70 (d, J = 8.6, 2H), 7.42 (d, J = 8.9, 1H, H-$C_{Ar,D}$), 6.63 (d, J = 8.6, 2H), 6.11 (s, 2H, H_2N), 5.18 (s, 2H, H_2N), 4.43 (hept, J = 6.1, 1H), 1.58 (s, 9H), 1.35 (d, J = 6.1, 6H).

^{13}C NMR (176 MHz, THF): δ = 168.7, 165.6, 164.8, 153.3, 145.3, 144.7, 137.5, 134.4, 130.9, 129.6, 127.4, 124.5, 123.1, 119.9, 114.0, 112.4, 108.5, 80.6, 74.8, 28.5, 22.9.

HRMS (ESI): calculated for $[M+H]^+$: 505.2446, found: 505.2448.

Synthesis of tert-butyl 4-(5-(4-aminobenzamido)-6-isopropoxypicolinamido)benzoate ***(S38)***

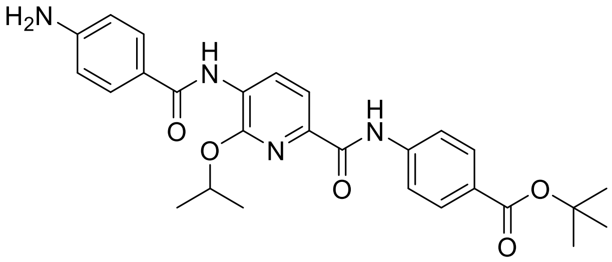

a) Note: *tert*-butyl 4-(5-amino-6-isopropoxypicolinamido)benzoate **S25** (354 mg crude DE-fragment) contained cyclohexane and was freeze-dried under vacuum to remove cyclohexane residues for this purpose, ending in a mass amount of 308 mg.

The nitro-CDE-fragment was obtained according to general procedure A, involving the reaction of the DE-fragment **S25** (218 mg, assumed as 1.00 Eq), 4-nitrobenzoyl chloride (123 mg, 1.13 Eq) and dry pyridine (0.14 mL, 3.0 Eq) in dry DCM (6.0 mL) for 45 min. EA was used as extraction solvent.

b) Modified from[6, 13]: The crude product was solved in THF/EtOH (3.0 mL each) under Argon atmosphere and AcOH (0.67 mL, 20 Eq) and Zn powder (384 mg, 10.0 Eq) were added. The mixture was stirred vigourously at RT while being monitored with LCMS. After 2.5 h the mixture was suspended between EA/sat. $NaHCO_3$ sol. (40 mL each) and the aqueous phase was extracted with EA (2x20 mL). The combined organic phases were dried over Na_2SO_4 and solvents were removed u.r.p.. The crude product was purified by FCC (solid loading, 80x theoretical product mass, cyclohexane/EA, 70/30->60/40->PE/acetone, 60/40) and solvents were removed u.r.p. to give two fractions, a pure fraction showing a beige fine powdered solid, 180 mg, 63% o2s.

^{1}H NMR (700 MHz, DMSO): δ = 10.24 (s, 1H, H-NCO), 8.95 (s, 1H, H-NCO), 8.60 (d, *J* = 8.1, 1H), 7.97 (d, *J* = 8.7, 2H), 7.92 (d, *J* = 8.7, 2H), 7.77 (d, *J* = 8.0, 1H), 7.67 (d, *J* = 8.7, 2H), 6.64 (d, *J* = 8.6, 2H), 5.93 (s, 2H, H_2N), 5.78 (hept, *J* = 6.2, 1H), 1.55 (s, 9H), 1.43 (d, *J* = 6.2, 6H).

^{13}C NMR (176 MHz, DMSO): δ = 165.0, 164.6, 162.5, 152.9, 152.0, 142.3, 139.9, 129.9, 129.3, 128.1, 126.7, 126.3, 119.7, 119.7, 116.4, 112.8, 80.4, 69.5, 27.9, 21.8.

HRMS (ESI): calculated for $[M+H]^+$: 491.2289, found: 491.2292.

*Synthesis of tert-butyl 4-(4-(4-aminobenzamido)-2-(difluoromethyl)-3-isopropoxybenzamido)benzoate (**S39**)*

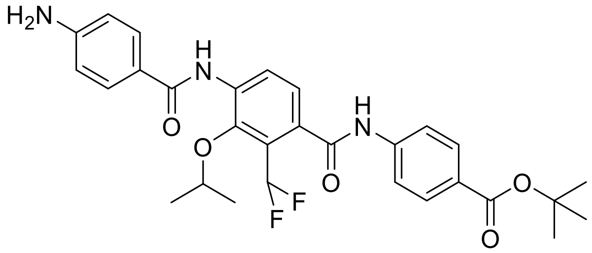

a) 4-Nitrobenzoyl chloride (107 mg, 0.57 mmol, 1.60 Eq) was added in portions to a mixture of tert-Butyl 4-(4-amino-2-(difluoromethyl)-3-isopropoxybenzamido)benzoate **S26** (151 mg, 0.36 mmol, 1.0 Eq) and pyridine (116 µL, 1.44 mmol, 4.00 Eq) in DCM (4 mL). The mixture was stirred at RT for 1 h. Then, the mixture was diluted with a 1 M $KHSO_4$ solution. The aq. phase was extracted with DCM (3x). The combined organic phases were dried over $MgSO_4$, filtered and concentrated to furnish the crude product (244 mg), which was used in the next step without further purification.

b) The crude (205 mg, 0.36 mmol, 1.0 Eq) was dissolved in THF (0.6 mL) and EtOH (0.5 mL). Zinc dust (352 mg, 5.39 mmol, 15.0 Eq) was added. The mixture was cooled to 0°C and AcOH (0.30 mL, 5.39 mmol, 15.0 Eq) was slowly added dropwise. The mixture was warmed to RT and stirred for 1 h, before Et_2O and sat. $NaHCO_3$ solution were added. The mixture was filtered and the phases were separated. The aq. phase was extracted with Et_2O (3x). The combined organic phases were washed with brine, dried over $MgSO_4$, filtered and concentrated under reduced pressure to furnish the title compound (188 mg) as yellowish amorphous solid, which was used in the next step without further purification.

HRMS (ESI): calculated for $[M+Na]^+$: 562.2129; found: 562.2124.

*Synthesis of tert-butyl 4-(2-(allyloxy)-4-(4-aminobenzamido)-3-methoxybenzamido) benzoate **(S40)***

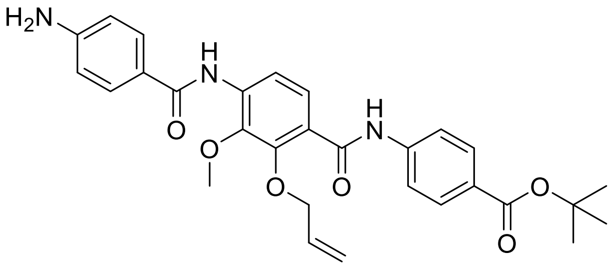

a) 2-(allyloxy)-3-methoxy-4-(4-nitrobenzamido)benzoic acid **S33** (300 mg, 0.806 mmol, 1.00 Eq) and *tert*-butyl 4-aminobenzoate (164 mg, 1.05 Eq) were suspended in dry EA (4.0 mL) under Argon atmosphere. Then dry pyridine (0.33 mL) and T3P (sol. in EA, 50%, 0.48 mL) were added and the reaction was stirred at RT. After 2.0 h EA (4.0 mL) was added to the mixture to enhance solubility of the reactants. After 3 h reaction and monitoring by TLC/LCMS the suspension was diluted between EA/HCl (0.5 M) (30 mL each) and the aqueous phase was extracted with EA (1x20 mL) (Note: The product may form a precipitate on top of the organic phase). Combined organic phases were washed with brine (1x10 mL) before removing solvent u.r.p.. The yellow crude product (498 mg) was directly used in the next step.

b) Modified from[6, 13]: The crude product was suspended in THF/EtOH (8 mL/4 mL) and AcOH (0.92 mL, 20 Eq) was added. Then Zn dust (0.53 mg, 10 Eq) was added and the reaction was stirred vigourously at RT. After 1 h 45 min AcOH (0.46 mL, 10 Eq) and Zn dust (0.26 mg, 5.0 Eq) were added again. The reaction was screened by LCMS and after 2.0 h the suspension was filtrated and the filtrate was diluted between EA/sat. $NaHCO_3$ sol. (40 mL each). The aqueous phase was extracted with EA (2x20 mL), combined organic phases were dried over Na_2SO_4 and solvents were removed u.r.p.. The material was purified by FCC (solid loading, 75x theoretical product mass, PE/acetone, 80/20->70/30->0/100) to give a brownish crystalline solid (301 mg, 72% o2s, NMR: 68% o2s). The product contained ~10 mol% of a byproduct (LCMS: 313 m/z, C-E coupling product).

^{1}H NMR (700 MHz, DMSO): δ = 10.49 (s, 1H, H-NCO), 9.16 (s, 1H, H-NCO), 7.89 (d, J = 8.8, 2H), 7.86 (d, J = 8.5, 1H), 7.83 (d, J = 8.8, 2H), 7.71 (d, J = 8.7, 2H), 7.39 (d, J = 8.5, 1H), 6.62 (d, J = 8.7, 2H), 6.03 (ddt, J = 17.3, 10.5, 5.6, 1H), 5.85 (s, 2H), 5.39

(dq, J = 17.2, 1.7, 1H), 5.20 (dq, J = 10.4, 1.3, 1H), 4.60 (dt, J = 5.6, 1.5, 2H), 3.90 (s, 3H), 1.55 (s, 9H).

^{13}C NMR (176 MHz, DMSO): δ = 165.0, 164.6, 164.4, 152.6, 149.1, 143.9, 143.0, 135.4, 133.6, 130.1, 129.4, 126.0, 125.9, 123.8, 120.2, 118.8, 118.1, 117.9, 112.7, 80.3, 74.5, 60.8, 27.8.

HRMS (ESI): calculated for $[M+H]^+$: 518.2304, found: 518.2289.

*Synthesis of allyl 4-(2-(allyloxy)-4-(4-aminobenzamido)-3-isobutylbenzamido)benzoate **(S41)***

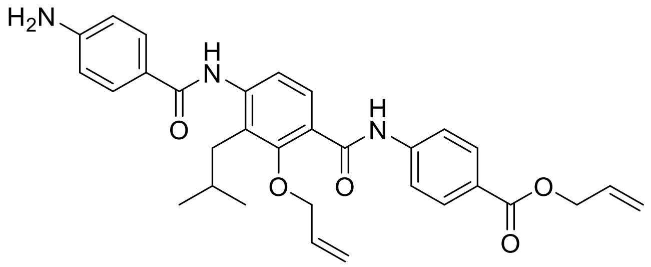

a) The nitro-CDE-fragment was obtained according to general procedure A, involving the reaction of the DE-fragment **S27** (57.9 mg, 142 µmol, 1.00 Eq), 4-nitrobenzoyl chloride (32 mg, 1.2 Eq) and dry pyridine (23 µL, 2.0 Eq) in dry DCM (2.8 mL) for 2 h. EA was used as extraction solvent.

b) Modified from[6, 13]: The crude product was solved in THF/EtOH (1.5 mL each) and AcOH (0.16 mL, 20 Eq) and Zn powder (93 mg, 10 Eq) were added. After 30 min AcOH (0.10 mL, 12 Eq) and Zn powder (56 mg, 6.0 Eq) were added and after 130 min AcOH (50 µL, 6.0 Eq) was added again. After 2.5 h the mixture was diluted between EA/sat. $NaHCO_3$ sol. (30 mL each) and the aqueous phase was extracted with EA (2x20 mL) before drying the combined organic phases over Na_2SO_4 and removing solvent u.r.p.. The material was purified by FCC (solid loading, 100x theoretical product mass, PE/EA, 50/50) to obtain an off colorless powder after coevaporation with heptane (59.9 mg, 80% o2s). NMR contains ~10 mol% impurity.

Note: When the substance was solved in $CDCl_3$ the solution turned into a solid gel-like mass (formation of salt?).

^{1}H NMR (700 MHz, CD_3CN): δ = 9.76 (s, 1H, H-NCO), 8.19 (s, 1H, H-NCO), 8.02 (d, J = 8.7, 2H), 7.84 – 7.79 (m, 3H), 7.72 – 7.67 (m, 3H), 6.71 (d, J = 8.7, 2H), 6.13 – 6.04

(m, 2H), 5.48 (dq, J = 17.2, 1.7, 1H), 5.42 (dq, J = 17.2, 1.6, 1H), 5.28 (ddq, J = 10.4, 5.9, 1.5, 2H), 4.80 (dt, J = 5.5, 1.6, 2H), 4.70 (s, 2H, H_2N), 4.42 (dt, J = 5.2, 1.6, 2H), 2.64 (d, J = 7.2, 2H), 0.92 (d, J = 6.6, 6H).

^{13}C NMR (176 MHz, CD_3CN): δ = 166.4, 166.4, 165.2, 156.7, 152.9, 144.2, 142.3, 134.1, 133.8, 133.7, 131.5, 130.3, 130.1, 129.5, 126.2, 125.4, 123.2, 122.4, 120.2, 114.4, 114.3, 76.7, 66.1, 34.9, 29.9, 23.0.

HRMS (ESI): calculated for $[M+H]^+$: 528.2493, found: 528.2493.

Synthesis of tert-butyl 4-(2-(allyloxy)-4-((4-aminobenzyl)amino)-N-(tert-butoxycarbonyl)-3-isopropoxybenzamido)benzoate ***(51)***

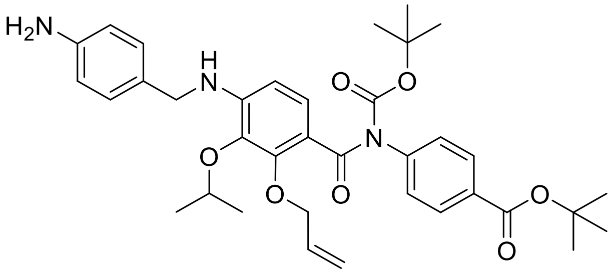

(proposed structure)

a) *tert*-butyl 4-(2-(allyloxy)-4-amino-3-isopropoxybenzamido)benzoate **50** (standard DE-fragment, provided by Evotec, 300 mg, 0.703 mmol, 1.00 Eq) and 4-nitrobenzaldehyde (106 mg, 1.00 Eq) were solved in dry DCM (2.0 mL) under Argon atmosphere. The mixture was stirred 4 d and monitored by LCMS (reaction showed no significant progress). Then AcOH (0.36 mL, 9.0 Eq) was added and $NaBH_4$ (80 mg, 3.0 Eq) was added portionwise at 0°C. The reaction mixture was stirred 1.5 h, before it was diluted between DCM/sat. $NaHCO_3$ sol. (15 mL each) and the aqueous phase was extracted with DCM (15 mL). The combined organic phases were washed with H_2O (15 mL) and the washing solution was reextracted with DCM (5 mL). All organic phases were combined and dried over Na_2SO_4 before the solvent was removed u.r.p..

To the crude material, 4-nitrobenzaldehyde **49** (106 mg, 1.00 Eq) was added and the mixture was solved in DCM (2.0 mL). AcOH (0.36 mL, 9.0 Eq) was added and the reaction was stirred for 1.5 h at RT, then at 40°C overnight. The next day, DiPEA (1.62 mL, 13.5 Eq) was added in two portions directly and after 2 h and the mixture was stirred further under reflux, still showing no significant process (analysis by LCMS).

The workup of the reaction mixture was performed as known, but with DCM/ HCl (1 M) and the solvent was removed from the combined organic phases u.r.p.. Molecular sieve (4Å), AcOH (0.36 mL, 9.0 Eq) and DCM (4 mL) were added to the concentrated solution and then $NaBH_4$ (80 mg, 3.0 Eq) was added at 0°C. The reaction solution was again stirred overnight at RT, subsequently diluted between DCM/sat. $NaHCO_3$ sol. (30 mL each) and the aqueous phase was extracted with DCM (2x15 mL). The combined organic phases were washed with H_2O (15 mL) and dried over Na_2SO_4. The crude product was purified by FCC (solid loading, 40x reactants mass, PE/EA, 80/20->70/30) and used as a mixed fraction with 4-benzylalcohol directly in the next step.

b) The mixed fraction and DMAP (spatula tip) were solved in dry THF (1.0 mL) under Argon atmosphere. Boc_2O (0.16 mL, 3.0 Eq) and dry TEA (0.11 mL, 3.5 Eq) were added dropwise and the reaction was stirred for 5.5 h at RT while being screened by LCMS. MeOH (5 mL) was added to the reaction mixture and solvents were removed u.r.p.. The crude material was directly used in next step.

c) Modified from[6, 13]: The crude material was suspended in THF/EtOH (1 mL each) and AcOH (0.26 mL, 20 Eq) and Zn (149 mg, 10.0 Eq) were added. After 30 min AcOH (0.13 mL, 10 Eq) and Zn (75 mg, 5.0 Eq) were added again and the reaction was stirred overnight. The reaction solution was diluted between DCM/water (20 mL each). The aqueous phase was extracted with DCM (2x15 mL) and the collected organic phases were dried over Na_2SO_4 before removing solvents u.r.p.. The product was isolated by FCC (solid loading, 80x reactant mass, PE/acetone, 90/10->85/15). The product fraction was identified by LCMS and solvents were removed u.r.p. to give a light-yellow gum, yield: 41.1 mg, 9.2% o3s (air sensitive!).

^{1}H NMR (500 MHz, $CDCl_3$): δ = 7.98 (d, J = 8.7, 2H), 7.28 – 7.21 (m, 3H), 7.11 (d, J = 8.5, 2H), 6.66 (d, J = 8.4, 2H), 6.44 – 6.35 (m, 1H, H-$C_{Ar,D}$), 6.12 – 6.03 (m, 1H), 5.40 (dq, J = 17.2, 1.6, 1H), 5.23 (dq, J = 10.4, 1.3, 1H), 4.89 (br s, 1H, $\underline{H}$-N-CH_2), 4.59 – 4.49 (m, 3H), 3.84 (br s, 2H, H_2N), 1.59 (s, 9H), 1.32 (s, 9H), 1.23 (d, J = 6.1, 6H).

^{13}C NMR (126 MHz, $CDCl_3$): δ= 169.7, 165.2, 152.7, 149.6, 146.9, 145.5, 143.5, 136.3, 133.9, 130.5, 129.9, 128.5, 127.7, 126.5, 118.9, 117.4, 115.3 (2x), 105.8, 82.6, 81.0, 75.0, 74.3, 47.1, 28.1, 27.6, 22.6.

HRMS (ESI): calculated for $[M+H]^+$: 632.3330, found: 632.3324.

Synthesis of tert-butyl 4-(2-(allyloxy)-4-((4-aminophenyl)carbamoyl)-3-isopropoxybenzamido)benzoate ***(59)***

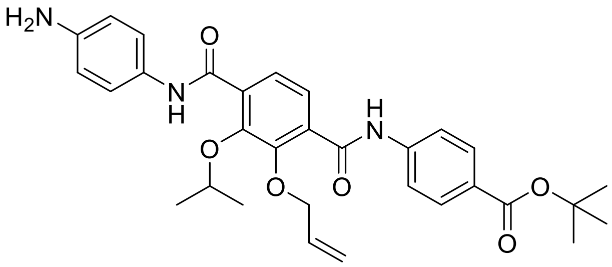

a) 3-(allyloxy)-4-((4-(*tert*-butoxycarbonyl)phenyl)carbamoyl)-2-isopropoxybenzoic acid **58** (402 mg, 0.883 mmol, 1.00 Eq) and 4-nitroaniline (146 mg, 1.20 Eq) were solved in dry EA (6.0 mL) under Argon atmosphere and dry pyridine (0.21 mL, 3.0 Eq) was added. The solution was stirred for few minutes before adding T3P (sol. in EA, 50%, 1.05 mL, 2.00 Eq) dropwise. The reaction was stirred at RT while being screened with LCMS. After 7 h the reaction mixture was diluted between EA/HCl (0.1 M) (30 mL each) and the aqueous phase was extracted with EA (2x20 mL). Organic phases were combined and dried over Na_2SO_4 before removing solvents u.r.p.. The crude product was directly used in the next step.

b) Modified from[6, 13]: The crude material was solved in THF/EtOH (7.0 mL each) and AcOH (1.01 mL, 20.0 Eq) and Zn powder (0.58 g, 10 Eq) were added. After 1.5 h stirring at RT the mixture was diluted between EA/sat. $NaHCO_3$ sol. (40 mL each) and the aqueous phase was extracted with EA (2x30 mL) before drying over Na_2SO_4 and removing solvent u.r.p.. The product was purified by FCC (solid loading, 50x reactant mass, cyclohexane/EA, 60/40->50/50) to obtain a yellow solid after coevaporation with MTBE/heptane (1x) and freeze pumping with liquid N_2 (2x). The yellow solid was stored under Argon atmosphere and its container was wrapped in Aluminium foil in the fridge (450 mg, 93% o2s, air/light sensitive!).

^{1}H NMR (500 MHz, CDCl3): δ = 10.18 (s, 1H, H-NCO), 9.68 (s, 1H, H-NCO), 8.09 (s, 2H), 8.00 (d, *J* = 8.9, 2H), 7.74 (d, *J* = 8.9, 2H), 7.50 (d, *J* = 8.9, 2H), 6.72 (d, *J* = 8.9, 2H), 6.16 (ddt, *J* = 17.1, 10.4, 6.0, 1H), 5.51 (dq, *J* = 17.1, 1.4, 1H), 5.43 (dq, *J* = 10.4, 1.1, 1H), 4.83 (hept, *J* = 6.3, 1H), 4.73 (dt, *J* = 6.0, 1.3, 2H), 3.66 (s, 2H, H_2N), 1.60 (s, 9H), 1.40 (d, *J* = 6.3, 6H).

^{13}C NMR (126 MHz, $CDCl_3$): δ = 165.5, 162.2, 161.8, 150.2, 148.6, 143.6, 141.9, 132.9, 132.2, 130.8, 129.9, 129.5, 127.8, 127.2, 126.9, 121.7, 120.4, 119.3, 115.7, 81.1, 78.3, 75.6, 28.4, 22.5.

HRMS (ESI): calculated for $[M+H]^+$: 546.2599, found: 546.2599.

*Synthesis of tert-butyl 4-((2-(allyloxy)-4-(4-aminobenzamido)-3-isopropoxybenzyl)(tert-butoxycarbonyl)amino)benzoate (**54**)*

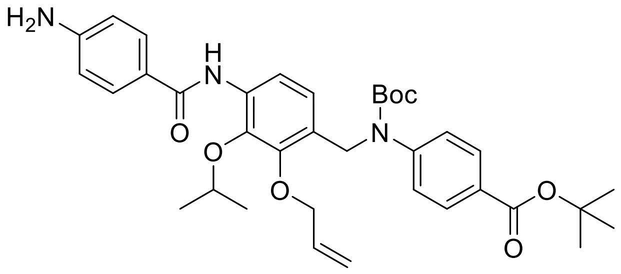

tert-butyl 4-((2-(allyloxy)-4-amino-3-isopropoxybenzyl)(tert-butoxycarbonyl)amino)benzoate **53** (133 mg, 0.26 mmol, 1.0 Eq) and 4-nitrobenzoyl chloride (72 mg, 0.39 mmol, 1.5 Eq) were added to a dry flask and further dried under high vacuum. Dry DCM (2 mL) was added and the mixture was cooled down to 0° C. Dry pyridine (80 µL, 1.0 mmol, 3.8 Eq) was slowly added to the stirring mixture. The reaction was slowly warmed up to room temperature. After completion, the reaction was quenched with saturated $NaHCO_3$ (4 mL) and brine (14 mL). The organic phase was extracted with DCM (3x8 mL). The solvent was concentrated under reduced pressure and dried under high vacuum. The crude residue was dissolved in THF (1.1 mL) and EtOH (0.9 mL) and cooled down to 0°C. Zinc dust (250 mg, 15 Eq) was added. Acetic acid (0.23 mL, 16 Eq) was added to the stirring mixture over the timespan of 1 h and the mixture was allowed to take room temperature. The reaction was controlled over LCMS. After 3 h AcOH (0.1 mL) was added dropwise over the timespan of 1 h. After completion, the zinc dust was filtered off. Saturated $NaHCO_3$ (10 mL) was added to the solution and the aqueous phase was extracted with EtOAc (3x5 mL). The combined organic phases were put on silica and the product was purified by chromatography. A yellow foam was obtained, yield: 123.8 mg, 76 % o2s.

^{1}H NMR (500 MHz, ACN): δ = 8.45 (br s, 1H), 8.05 (d, 1H, *J* = 8.5 Hz), 7.83 (d, 2H, *J* = 8.7 Hz), 7.64 (d, 2H, *J* = 8.7 Hz), 7.27 (d, 2H, *J* = 8.7 Hz), 6.97 (d, 1H, *J* = 8.6 Hz), 6.70 (d, 2H, *J* = 8.7 Hz), 6.04 (ddt, 1H, *J* = 5.7 Hz, 10.5 Hz, 16.2 Hz), 5.32 (ddd, 1H, *J*

= 1.6 Hz, 3.2 Hz, 17.2 Hz), 5.20 (dd, 1H, *J* = 1.6 Hz, 10.5 Hz), 4.91 (s, 2H), 4.69 (br s, 2H), 4.54 (quart., 1H, *J* = 6.2 Hz), 4.39 (dt, 2H, *J* = 1.3 Hz, 5.7 Hz), 1.53 (s, 9H), 1.42 (s, 9H), 1.22 (d, 6H, *J* = 6.2 Hz)

^{13}C NMR (126 MHz, ACN): *δ* = 165.9, 165.4, 155.0, 152.7, 150.0, 147.4, 140.6, 135.1, 134.3, 130.3, 129.8, 129.6, 127.7, 127.0, 124.0, 123.4, 118.0, 116.3, 114.5, 81.7, 81.5, 76.3, 74.1, 48.6, 28.4, 28.3, 22.7

Synthesis of allyl 4-(4-(2-(allyloxy)-4-(4-aminobenzamido)-3-isopropoxyphenyl)-1H-1,2,3-triazol-1-yl)benzoate ***(65)***

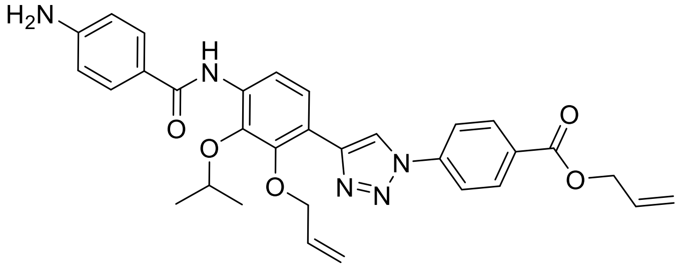

a) The nitro-CDE-fragment was obtained according to general procedure A, involving the reaction of the DE-fragment **64** (130 mg, 0.299 mmol, 1.00 Eq), 4-nitrobenzoyl chloride (58 mg, 0.31 mmol, 1.05 Eq) and dry pyridine (45 µL, 2.0 Eq) in dry DCM (6.0 mL) for 16 h.

b) Modified from[6, 13]: For the following step the material was splitted in two equally sized batches: The crude material was suspended in EtOH (5.1 mL) and AcOH (0.63 µL, 37 Eq) under Argon atmosphere and the solution was cooled in an ice bath. Zn powder (0.21 g, 11 Eq) was added in two portions over 15 min and the mixture was vigourously stirred at RT. The reaction was screened by LCMS and after 2 h or 3.5 h both approaches were combined. The reaction solution was diluted between MTBE (50 mL) and sat. $NaHCO_3$ sol. (50 mL). The aqueous phase was extracted again with MTBE (1x50 mL) and the combined organic phases were washed with brine (50 mL). After drying over Na_2SO_4 and rinsing of the Na_2SO_4-solid with EA the solvents were removed u.r.p.. Because the reaction was not completed, the solid was again suspended in a EtOH/THF (5 mL/2 mL) under Argon atmosphere. AcOH (0.65 mL, 35 Eq) and Zn powder (0.21 g, 10 Eq) were added in 2 portions over 15 min and the reaction was stirred for 1 h 45 min. After reaction control by TLC and LCMS the mixture was diluted between EA and sat. $NaHCO_3$ sol.(50 mL each) and the aqueous phase

was extracted with EA (2x50 mL). The combined organic phases were washed with brine (1x70 mL) and dried over Na_2SO_4. The crude product was purified by FCC (solid loading, 75x reactants mass, PE/EA, 65/35->60/40) to give a yield of 130 mg, 79% o2s.

^{1}H NMR (700 MHz, Acetone): δ = 8.87 (s, 1H), 8.73 (s, 1H), 8.43 (d, J = 8.7, 1H), 8.28 (d, J = 8.8, 2H), 8.12 (d, J = 8.8, 2H), 8.04 (d, J = 8.7, 1H), 7.76 (d, J = 8.6, 2H), 6.79 (d, J = 8.6, 2H), 6.24 – 6.16 (m, 1H), 6.16 – 6.09 (m, 1H), 5.51 (dq, J = 17.2, 1.7, 1H), 5.47 (dq, J = 17.2, 1.7, 1H), 5.38 (s, 2H), 5.32 – 5.29 (m, 2H), 4.88 (dt, J = 5.6, 1.5, 2H), 4.79 (hept, J = 6.2, 1H), 4.66 (dt, J = 5.6, 1.5, 2H), 1.39 (d, J = 6.1, 6H).

^{13}C NMR (176 MHz, Acetone): δ = 165.6, 165.2, 153.4, 149.1, 144.9, 141.5, 141.1, 135.8, 135.4, 133.6, 132.2, 131.0, 129.8, 123.3, 123.1, 121.2, 120.9, 120.6, 118.5, 118.2, 116.8, 114.5, 76.8, 73.8, 66.5, 23.0.

HRMS (ESI): calculated for $[M+H]^+$: 554.2398, found: 554.2388.

*Synthesis of tert-butyl 4'-(4-aminobenzamido)-3'-isopropoxy-2'-(methoxymethoxy)-[1,1'-biphenyl]-4-carboxylate (**68**)*

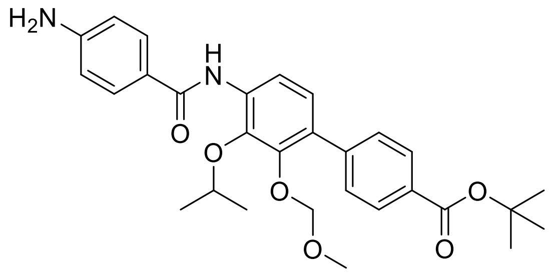

tert-butyl 4'-amino-3'-isopropoxy-2'-(methoxymethoxy)-[1,1'-biphenyl]-4-carboxylate **67** (73 mg, 0.19 mmol, 1.0 Eq) and 4-nitrobenzoyl chloride (53.0 mg) were added to a dry flask under nitrogen atmosphere. Dry DCM (1.6 mL) was added and the mixture was cooled down to 0°C. Dry pyridine (61µL, 0.73 mmol, 4 Eq) was slowly added to the stirring mixture. After completion, the reaction was quenched with HCl (2 mL, 1M) and brine (8 mL). The organic phase was extracted with EtOAc (4x4 mL). The organic phases were combined and washed with saturated $NaHCO_3$ solution (2x5 mL). The solvent was evaporated under reduced pressure. To the crude residue, zinc dust (187 mg, 15 Eq), THF (0.9 ml) and EtOH (0.7 mL) were added. The mixture was cooled down to 0°C. Acetic acid (0.17 mL, 16 Eq) was slowly added to the stirring mixture at 0°C over 1 h. The mixture was allowed to reach RT. After completion, the zinc dust

was filtered off. Saturated $NaHCO_3$ solution (10 mL) was added and the aqueous phase was extracted with EtOAc (3x8 mL). The organic solvent was removed under reduced pressure. The crude product was purified by chromatography (PE/ EtOAc). A brown semi-solid was obtained, yield: 66.8 mg, 70 %.

^{1}H NMR (500 MHz, $CDCl_3$): δ = 8.59 (s, 1H), 8.38 (d, 1H, J = 8.6 Hz), 8.02 (d, 2H, J = 8.6 Hz), 7.75 (d, 2H, J = 8.7 Hz), 7.60 (d, 2H, J = 8.5 Hz), 7.13 (d, 1H, J = 8.6 Hz), 6.74 (d, 2H, J = 8.7 Hz), 4.86 (s, 2H), 4.76 (hept., 1H, J = 6.1 Hz), 3.03 (s, 3H), 1.62 (s, 9H), 1.38 (d, 6H, J = 6.2 Hz)

^{13}C NMR (126 MHz, $CDCl_3$): δ = 166.0, 164.9, 150.1, 147.0, 142.8, 139.8, 134.1, 130.9, 130.5, 129.6, 129.4, 129.0, 125.7, 124.5, 115.8, 114.5, 99.0, 81.1, 76.2, 57.5, 28.4, 23.0

HRMS (ESI): calculated for $[M+H]^+$: 507.2495, found: 507.2490.

*Synthesis of tert-butyl 4-(2-(allyloxy)-3-isopropoxy-N-methyl-4-(4-nitrobenzamido)benzamido)benzoate (**S42**)*

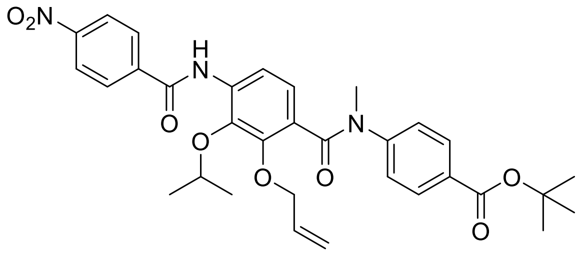

tert-Butyl 4-(2-(allyloxy)-4-amino-3-isopropoxy-*N*-methylbenzamido)benzoate (**83**) (200 mg, 0.45 mmol, 1.0 Eq) and pyridine (150 µL, 1.82 mmol, 4.00 Eq) was dissolved in DCM (3 mL). 4-Nitrobenzoyl chloride (135 mg, 0.73 mmol, 1.60 Eq) was added in small portions. The solution was stirred at RT für 3 h. A sat. $KHSO_4$ solution was added and the aq. phase was extracted with DCM (2x). The combined organic phases were dried over $MgSO_4$, filtered and concentrated under reduced pressure. The crude product was purified by column chromatography (dry load, PE/EtOAc = 3:1) to furnish the title compound (245 mg, 92%) as yellow amorphous solid.
The *N*-methyl group is not visible in the ^{13}C NMR spectrum and may appear underneath the solvent peak.
R_f (PE/EtOAc = 3:1) = 0.18;

^{1}H NMR (400 MHz, DMSO-d_6): δ = 9.87 (s, 1H, NH), 8.36-8.34 (d, J = 8.8 Hz, 2H, H_{Ar}), 8.14-8.12 (d, J = 8.6 Hz, 2H, H_{Ar}), 7.72-7.71 (d, J = 6.3 Hz, 2H, H_{Ar}), 7.54-7.52 (d, J = 8.2 Hz, 1H, H_{Ar}), 7.24 (bs, 2H, H_{Ar}), 7.06-7.04 (d, J = 8.2 Hz, 1H, H_{Ar}), 6.07-5.97 (m, 1H, C<u>H</u>CH$_2$), 5.39-5.34 (dd, J = 1.6, 17.2 Hz, 1H, CHC<u>H</u>$_2$), 5.26-5.23 (dd, J = 1.5, 10.5 Hz, 1H, CHC<u>H</u>$_2$), 4.43 (bs, 2H, OCH$_2$), 4.05-4.00 (m, 1H, C<u>H</u>(CH$_3$)$_2$), 3.39 (s, 3H, NCH$_3$), 1.49 (s, 9H, C(CH$_3$)$_3$), 0.97 (bs, 6H, CH(C<u>H</u>$_3$)$_2$)
^{13}C NMR (101 MHz, DMSO-d_6): δ = 167.1 (CO), 164.2 (CO), 163.5 (CO), 149.2 (C_{Ar}), 147.8 (C_{Ar}), 147.3 (C_{Ar}), 142.7 (C_{Ar}), 139.9 (C_{Ar}), 134.0 (C_{Ar}), 133.6 (<u>C</u>HCH$_2$), 129.1 (C_{Ar}), 129.0 (C_{Ar}), 128.9 (C_{Ar}), 126.5 (C_{Ar}), 123.7 (C_{Ar}), 122.6 (C_{Ar}), 119.8 (C_{Ar}), 117.1 (C-12), 80.7 (<u>C</u>(CH$_3$)$_3$), 75.2 (<u>C</u>H(CH$_3$)$_2$), 73.9 (OCH$_2$), 27.7 (C(<u>C</u>H$_3$)$_3$), 21.8 (CH(<u>C</u>H$_3$)$_2$)
HRMS (ESI): calculated for $[M+Na]^+$: 612.2322; found: 612.2311.

*Synthesis of tert-butyl 4-(2-(allyloxy)-4-(4-aminobenzamido)-3-isopropoxy-N-methylbenzamido)benzoate (**84**)*

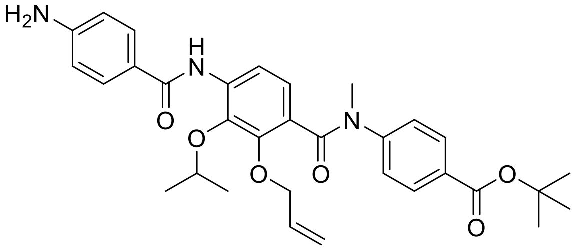

tert-Butyl 4-(2-(allyloxy)-4-(4-aminobenzamido)-3-isopropoxy-*N*-methylbenzamido)benzoate (**S42**) (239 mg, 0.40 mmol, 1.0 Eq) was dissolved in THF (1 mL) and EtOH (1 mL). Zinc dust (397 mg, 6.07 mmol, 15.0 Eq) was added. The mixture was cooled to 0°C and AcOH (350 µL, 6.07 mmol, 15.0 Eq) was added dropwise over 1 h. The mixture was warmed to RT and stirred for 1 h. After completion of the reaction, Et_2O was added and the reaction was terminated with a sat. $NaHCO_3$ solution. The mixture was filtered and the phases were separated. The aq. phase was extracted with Et_2O (2x). The combined organic phases were washed with brine, dried over $MgSO_4$, filtered and concentrated under reduced pressure. The crude product was purified by column chromatography (PE/EtOAc = 1:1) to furnish the title compound (163 mg, 72%) as colorless amorphous solid.
The *N*-methyl group is not visible in the ^{13}C NMR spectrum and may appear underneath the solvent peak.
R_f (PE/EtOAc = 1:1) = 0.44;

^{1}H NMR (400 MHz, DMSO-d$_6$): *δ* = 8.81 (s, 1H, NH), 7.79-7.69 (m, *J* = 8.6 Hz, 4H, H$_{Ar}$), 7.61-7.59 (d, *J* = 8.6 Hz, 2H, H$_{Ar}$), 7.22 (bs, 2H, H$_{Ar}$), 7.01-6.99 (d, *J* = 8.6 Hz, 1H, H$_{Ar}$), 6.87-6.85 (d, *J* = 8.6 Hz, 1H, H$_{Ar}$), 6.60-6.58 (d, *J* = 8.6 Hz, 2H, NH$_2$) 6.06-5.96 (m, 1H, C$\underline{H}$CH$_2$), 5.38-5-33 (dd, *J* = 1.7, 17.2 Hz, 1H, CHC$\underline{H}_2$), 5.25-5.22 (dq, *J* = 1.6, 10.5 Hz, 1H, CHC$\underline{H}_2$), 4.41-40 (d, *J* = 4.2 Hz, 2H, OCH$_2$), 4.02 (bs, 1H, C$\underline{H}$(CH$_3$)$_2$), 3.38 (s, 3H, NCH$_3$), 1.48 (s, 9H, C(CH$_3$)$_3$), 0.99 (bs, 6H, CH(C$\underline{H}_3$)$_2$)
^{13}C NMR (101 MHz, DMSO-d$_6$): *δ* = 167.2 (CO), 164.3 (CO), 164.2 (CO), 152.5 (C$_{Ar}$), 147.4 (C$_{Ar}$), 134.8 (C$_{Ar}$), 134.6 (C$_{Ar}$), 134.1 ($\underline{C}$HCH$_2$), 129.1 (C$_{Ar}$), 128.9 (C$_{Ar}$), 128.7 (C$_{Ar}$), 128.3 (C$_{Ar}$), 127.5 (C$_{Ar}$), 127.0 (C$_{Ar}$), 126.4 (C$_{Ar}$), 122.8(C$_{Ar}$), 120.1 (C$_{Ar}$), 117.1 (CH$\underline{C}$H$_2$), 112.8(C$_{Ar}$), 80.7 ($\underline{C}$(CH$_3$)$_3$), 75.1 ($\underline{C}$H(CH$_3$)$_2$), 73.9 (OCH$_2$), 27.7 (C($\underline{C}$H$_3$)$_3$), 21.8 (CH($\underline{C}$H$_3$)$_2$)
HRMS (ESI): calculated for [M+Na]$^+$: 582.2580; found: 582.2585.

*Synthesis of 1-allyl 5-methyl 2-(2-(allyloxy)-4-(4-aminobenzamido)-3-isopropoxyphenyl)-benzo[d]imidazole-1,5-dicarboxylate and 1-allyl 6-methyl 2-(2-(allyloxy)-4-(4-aminobenzamido)-3-isopropoxyphenyl)-1H-benzo[d]imidazole-1,6-dicarboxylate (**S43**)*

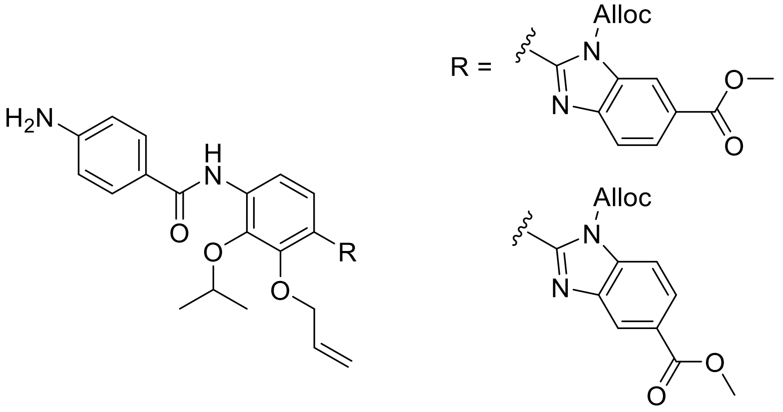

a) A mixture of 1-allyl 5-methyl 2-(2-(allyloxy)-4-amino-3-isopropoxyphenyl)-1H-benzo[d]imidazole-1,5-dicarboxylate and 1-allyl 6-methyl 2-(2-(allyloxy)-4-amino-3-isopropoxyphenyl)-1H-benzo[d]imidazole-1,6-dicarboxylate (**S43**, 120 mg, 0.26 mmol, 1.0 Eq) as well as 4-nitrobenzoyl chloride (72.0 mg, 0.39 mmol, 1.5 Eq) was added to a dry flask and further dried under high vacuum. Dry DCM (2.0 mL) was added and the mixture was cooled down to 0° C. Dry pyridine (80 µL, 0.99 mmol, 3.9 Eq) was slowly added to the stirring mixture. The reaction was slowly warmed up to RT. After completion, the reaction was quenched with saturated NaHCO$_3$ solution (4 mL) and

brine (14 mL). The organic layer was extracted with DCM (3x8 mL). The solvent was concentrated under reduced pressure and dried under high vacuum.

b) The crude product was dissolved in THF (1.1 mL) and EtOH (0.9 mL) and cooled down to 0 °C. Zinc dust (240 mg, 14 Eq) was added. AcOH (0.21 mL, 14 Eq) was added to the stirring mixture over 30 min and the mixture was allowed to take RT. After completion, the zinc dust was filtered off. Saturated $NaHCO_3$ solution (10 mL) was added to the solution and it was extracted with EtOAc (3x5 mL). The combined organic phases were purified by chromatography. A yellow foam was obtained (101.2 mg) that was directly used in the next step.

AA-CDE-fragments and cystobactamid precursors

General procedure **B** (coupling between the standard AB-central AA-fragment and CDE-fragments):

The CDE-fragment and the standard AB-central AA-fragment **26** were suspended under Argon atmosphere in dry EA and pyridine and T3P (50% solution in EA) were added. The reaction was stirred at RT for the given amount of time while being screened by TLC or LCMS. Clarification of the original solution was often observed. The reaction solution was diluted between EA (20-30 mL) and an adequate amount of HCl (0.1-1 M, 15-30 mL) and the aqueous phase was extracted with EA (2x15-20 mL). The combined organic phases were dried over Na_2SO_4 and the solvent was removed u.r.p. to give the crude product or intermediate. If a workup was omitted, only the solvent was removed u.r.p..

Synthesis of tert-butyl (S)-4-(4-(4-(2-((((9H-fluoren-9-yl)methoxy)carbonyl)amino)pent-4-ynamido)benzamido)-2-(allyloxy)-5-isopropoxybenzamido)benzoate ***(S44)***

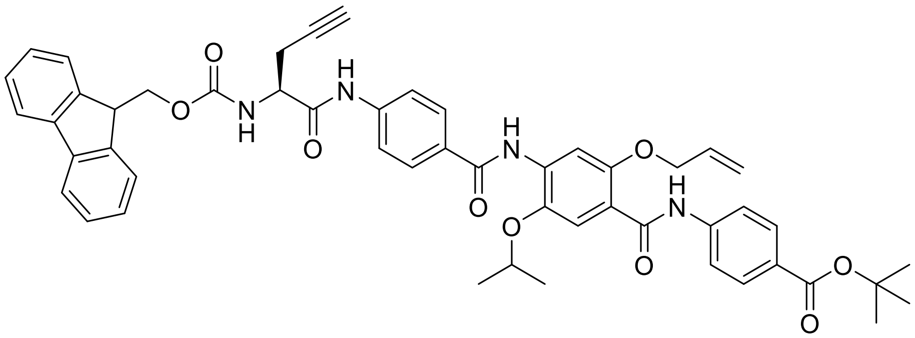

Modified from[18]:

CDE-fragment **S34** (36 mg, 66 µmol, 1.0 Eq) was solved in dry EA (1.4 mL) under N_2 atmosphere. This solution was added to an ice cold solution of (S)-2-((((9H-fluoren-9-yl)methoxy)carbonyl)amino)pent-4-ynoic acid (Fmoc-L-Pra-OH, 33 mg, 1.5 Eq) and pyridine (16 µL, 3.0 Eq) in dry EA (0.16 mL). The reaction mixture was cooled to 0°C and T3P (50% sol. in EA, 82 µL, 2.0 Eq) was added dropwise. The yellow solution was stirred for 2 h at 0°C, and was screened by TLC. After 2.2 h the reaction was stopped by solving the reaction solution between H_2O (2 mL), HCl (1 M, 1.5 mL) and EA (2 mL). The aqueous phase was extracted with EA (2x) and the combined organic phases were washed with brine (2 mL). The solvent was removed u.r.p. and the residue was purified by FCC (solid loading, 50x reactants mass, PE/EA, 65/35). To remove residual

amounts of carboxylic acid, solvents were removed u.r.p. from the solution, MTBE (15 mL) was added and the resulting solution was washed with sat. $NaHCO_3$ sol. (3x10 mL). After solvent removal a colorless solid was obtained, yield: 45 mg, 79%.

^{1}H NMR (500 MHz, $CDCl_3$): δ = 10.36 (s, 1H, H-NCO), 8.84 (s, 1H, H-NCO), 8.52 (s, 1H), 8.47 (br s, 1H, H-NCO), 7.98 (d, J = 8.8, 2H), 7.87 (d, J = 8.8, 3H), 7.77 (d, J = 7.6, 2H), 7.71 (d, J = 8.8, 2H), 7.69 (d, J = 8.8, 2H), 7.59 (d, J = 7.4, 2H), 7.40 (t, J = 7.5, 2H, H-$C_{fluoren}$), 7.30 (t, J = 7.2, 2H, H-$C_{fluoren}$), 6.22 (ddt, J = 17.2, 10.4, 5.9, 1H, <u>H</u>-C(=CH_2)), 5.63 – 5.54 (m, 2H, H-NCOO, H_2C=C), 5.49 (dq, J = 10.4, 1.0, 1H, H_2C=C), 4.81 (dt, J = 5.9, 1.1, 2H, H_2C-O), 4.75 (hept, J = 6.0, 1H, H-C_{iPr}O), 4.58 – 4.44 (m, 3H, H-C(Ar)$_2$, H_2C-O), 4.25 (t, J = 6.7, 1H, H-C), 2.97 – 2.64 (m, 2H, <u>H</u>$_2$C(CCH)), 2.17 (t, J = 2.6, 1H, H-CC), 1.60 (s, 9H, (H_3C)$_3$C), 1.42 (d, J = 6.1, 6H, (<u>H</u>$_3$C)$_2$CH).

^{13}C NMR (126 MHz, $CDCl_3$): δ = 168.4, 165.6, 164.6, 163.2, 156.7, 151.4, 143.6, 143.5, 142.6, 141.5, 140.9, 133.4, 131.9, 130.8, 130.5, 128.3, 128.1, 128.0, 127.3, 125.0, 120.7, 120.3, 120.0, 119.1, 116.0, 115.6, 104.9, 80.9, 79.1, 77.4, 72.5, 71.3, 67.7, 54.3, 47.3, 28.4, 27.1, 22.5.

Synthesis of allyl/tBu-protected p-hydroquinone cystobactamid ***(S45)***

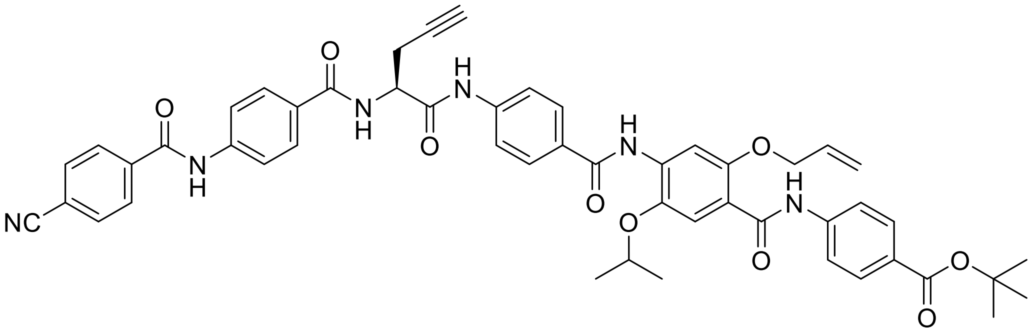

Modified from[6, 18]:

a) Note: following reactions were conducted in one small (6 µmol) and one large (41 µmol reactant) batch that were combined before column purification.

The Fmoc-protected AA-CDE-fragment **S44** (40 mg, 41 +5.6 µmol, 1.0 Eq) was suspended in MeCN (0.8 mL) under nitrogen atmosphere and $HNEt_2$ (81 µL, 17 Eq) was added at 0°C. Reaction stirred 1 h at 0°C and 1 h at RT. Reactant was fully converted as shown with LCMS. Solvents were removed from the mixture u.r.p. and

by multiple coevaporation with MeCN. The crude product was further dried u.r.p. and used directly in next step.

b) The crude material, 4-(4-cyanobenzamido)benzoic acid **86** (16 mg, 60 µmol, 1.3 Eq) and HATU (23 mg, 60 µmol, 1.3 Eq) were solved in DMF (0.6 mL) under N_2 atmosphere at 0°C. After 5 min, DiPEA was added (24 µL, 3.0 Eq) and the reaction mixture stirred further at 0°C until LCMS showed full reactant conversion. The reaction was quenched with HCl (0.1 M, 3 mL) and brine (12 mL) was added before the aqueous phase was extracted with EA and the organic phase was washed with brine. Solvents were removed u.r.p. to get a yellowish solid that was purified by FCC (solid loading, PE/EA, 40/60->50/50). A colorless solid was obtained, yield: 33 mg, 80% o2s, NMR: <70% o2s. The product contained ~50 mol% tetramethylurea and minor impurities and was used without further purification.

^{1}H NMR (500 MHz, Acetone): *δ* = 10.34 (s, 1H, H-NCO), 10.01 (s, 1H, H-NCO), 9.90 (s, 1H, H-NCO), 9.01 (s, 1H, H-NCO), 8.51 (s, 1H, H-$C_{Ar,D}$), 8.18 (d, *J* = 8.7, 2H), 8.13 (d, *J* = 7.6, 1H, H-NCO), 8.02 (d, *J* = 9.0, 2H), 7.98 – 7.92 (m, 8H), 7.91 – 7.83 (m, 4H), 7.77 (s, 1H), 6.34 (ddt, *J* = 17.2, 10.4, 5.8, 1H), 5.64 (dq, *J* = 17.2, 1.4, 1H), 5.47 (dq, *J* = 10.4, 1.0, 1H), 4.97 (q, *J* = 7.5, 1H), 4.86 (d, *J* = 5.8, 2H), 4.81 – 4.72 (m, 1H), 3.00 – 2.86 (m, 2H), 2.52 (t, *J* = 2.6, 1H), 1.59 (s, 9H), 1.43 (d, *J* = 6.1, 6H).

^{13}C NMR (126 MHz, Acetone): *δ* = 170.1, 167.3, 165.6, 165.2, 165.0, 163.6, 152.1, 143.9, 143.3, 143.0, 141.8, 139.8, 134.6, 133.5, 133.3, 131.2, 129.3 (3x), 129.0, 127.7, 120.4, 120.3 (2x), 120.2, 119.7, 118.7, 116.9, 116.3, 115.9, 105.9, 81.0, 80.7, 73.2, 72.4, 71.6, 54.6, 28.4, 22.3, 22.2.

HRMS (ESI): calculated for $[M+H]^{+}$: 889.3556, found: 889.3540.

*Synthesis of tert-butyl 4-(4-(4-((2S,3R)-4-amino-2-((tert-butoxycarbonyl)amino)-3-methoxy-4-oxobutan amido)benzamido)-2-(difluoromethyl)-3-isopropoxybenzamido)benzoate (**S46**)*

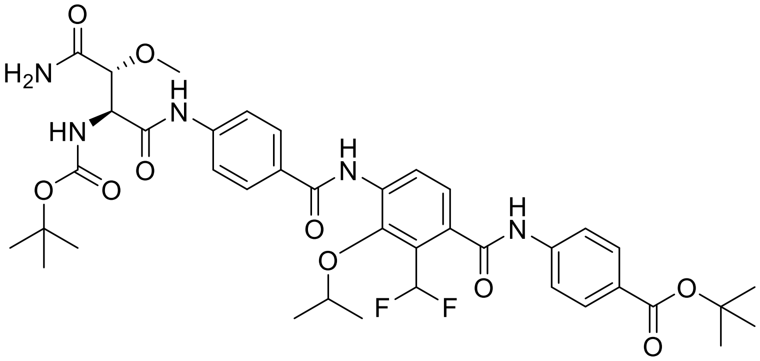

tert-Butyl 4-(4-(4-aminobenzamido)-2-(difluoromethyl)-3-isopropoxybenzamido)benzoate **S39** (90.6 mg, 0.17 mmol, 1.0 Eq), the *N*-Boc-methoxy asparagine (74.9 mg, 0.29 mmol, 1.70 Eq) and EEDQ (66.4 mg, 0.27 mmol, 1.60 Eq) were dissolved in precooled $CHCl_3$ (1 mL) at 0°C. The mixture was stirred for 16 h while warming to RT. The mixture was concentrated under reduced pressure and the residue was purified by column chromatography (dry load, washing with 20% Et_2O in DCM, elution with 3% MeOH in DCM) to furnish the product (45.9 mg, 33% o3s) as orange amorphous solid.

R*f* (3% MeOH in DCM) = 0.10;

$[\alpha]_D^{25}$ = -0.5 ° (c 0.1, MeOH);

^{1}H NMR (500 MHz, DMSO-d_6): δ = 10.76 (s, 1H, NH), 10.35 (s, 1H, NH), 9.96 (s, 1H, NH), 8.03-8.02 (d, J = 8.8 Hz, 2H, H_{Ar}), 7.90-7.88 (d, J = 8.8 Hz, 2H, H_{Ar}), 7.88-7.86 (d, J = 8.8 Hz, 1H, H_{Ar}), 7.83-7.81 (d, J = 8.8 Hz, 2H, H_{Ar}), 7.80-7.78 (d, J = 8.7 Hz, 2H, H_{Ar}), 7.45-7.41 (d, J = 18.6 Hz, 2H, NH_2), 7.39-7.38 (d, J = 8.2 Hz, 1H, H_{Ar}), 7.30-7.08 (t, J = 53.6 Hz, 1H, CHF_2), 6.80-6.79 (d, J = 8.2 Hz, 1H, CHN<u>H</u>), 4.41-4.35 (m, 2H, C<u>H</u>NH, C<u>H</u>(CH_3)$_2$), 3.86-3.84 (d, J = 7.4 Hz, 1H, C<u>H</u>OCH_3), 3.25 (s, 3H, OCH_3), 1.55 (s, 9H, C(CH_3)$_3$), 1.38 (s, 9H, C(CH_3)$_3$), 1.23-1.22 (d, J = 6.2 Hz, 6H, CH(C<u>H</u>$_3$)$_2$)

^{13}C NMR (126 MHz, DMSO-d_6): δ = 170.6 (CO), 168.9 (CO), 166.2 (CO), 164.6 (CO), 164.6 (CO), 154.9 (CO), 150.0 (C_{Ar}), 143.2 (C_{Ar}), 142.2 (C_{Ar}), 134.2 (C_{Ar}), 133.2 (C_{Ar}), 130.0 (C_{Ar}), 129.4 (C_{Ar}), 128.6 (C_{Ar}), 128.2 (C_{Ar}), 126.1 (C_{Ar}), 124.9 (C_{Ar}), 123.3 (C_{Ar}), 118.8 (C_{Ar}), 118.7 (C_{Ar}), 113.8-110.1 (t, J = 236.5 Hz, CHF_2), 80.4 (2C, <u>C</u>(CH_3)$_3$), 80.1 (<u>C</u>HOCH_3), 78.7 (CHNH), 77.0 (<u>C</u>H(CH_3)$_2$), 57.6 (OCH_3), 28.1 (C(<u>C</u>H$_3$)$_3$), 27.9 (C(<u>C</u>H$_3$)$_3$), 21.9 (CH(<u>C</u>H$_3$)$_2$)

HRMS (ESI): calculated for $[M+Na]^+$: 806.3189; found: 806.3204.

*Synthesis of tert-butyl 4-(4-(4-((2S,3R)-4-amino-2-(4-(4-cyanobenzamido)benzamido)-3-methoxy-4-oxobutanamido)benzamido)-2-(difluoromethyl)-3-isopropoxybenzamido)benzoate (**S47**)*

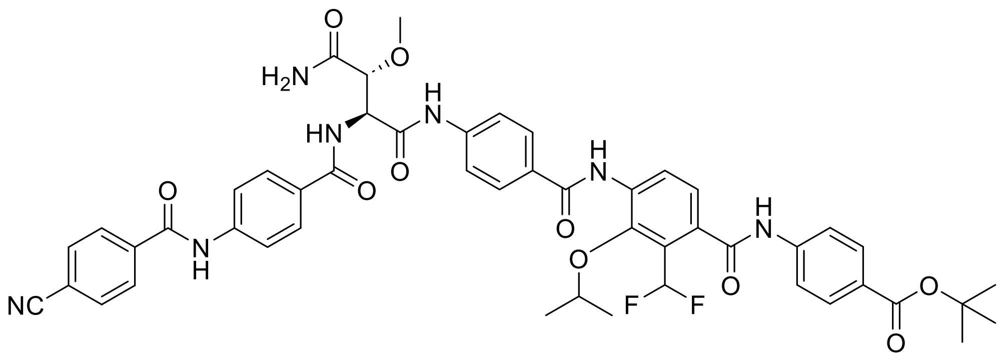

a) Carbamate **S46** (44.0 mg, 0.06 mmol) was dissolved in HCl (4 M in 1,4-dioxane, 1.40 mL, 5.61 mmol, 100 Eq) at 0°C. The mixture was warmed up to RT and stirred for 15 min. The solution was transferred to a stirred suspension of EtOAc (40 mL) and a sat. $NaHCO_3$ solution (40 mL). The aq. phase was extracted with EtOAc and the combined organic phases were washed with brine, dried over $MgSO_4$, filtered and concentrated under reduced pressure. The crude product was used in the next step without further purification.

b) DIPEA (28 µL, 0.16 mmol, 3.00 Eq) was added dropwise to a stirred solution of HATU (24.6 mg, 0.06 mmol, 1.20 Eq) and the carboxylic acid **86** (17.2 mg, 0.06 mmol, 1.20 Eq) in DMF (1.4 mL). The solution was stirred for 5 min and was then transferred to a stirred solution of the product from a) (36.8 mg, 0.05 mmol) in DMF (0.8 mL). The reaction mixture was stirred at RT for 18 h. The mixture was diluted with EtOAc and washed with HCl (1M), brine, dried over $MgSO_4$, filtered and concentrated under reduced pressure. The crude product was purified by column chromatography (dry load, MeOH in DCM = 3%, 4%, 5%) to furnish the title compound (16.9 mg, 33% o2s) as colorless amorphous solid.

R*f* (5% MeOH in DCM) = 0.19;

[*α*]$_D^{23}$ = +2.4 ° (c 0.1, MeOH);

^{1}H NMR (600 MHz, DMSO-d$_6$): *δ* = 10.76 (s, 1H, NH), 10.72 (s, 1H, NH), 10.55 (s, 1H, NH), 9.97 (s, 1H, NH), 8.46-8.44 (d, *J* = 8.2 Hz, 2H, CHNH), 8.13-8.11 (m, 2H, H_{Ar}), 8.05-8.03 (m, 4H, H_{Ar}), 7.91-7.81 (m, 11H, H_{Ar}), 7.54-7.48 (d, *J* = 39.3 Hz, 2H, NH_2), 7.39-7.38 (d, *J* = 8.2 Hz, 1H, H_{Ar}), 7.28-7.10 (t, *J* = 53.7 Hz, 1H, CHF_2), 4.93-4.90 (t, *J* = 8.1 Hz, 1H, CHNH), 4.40-4.34 (sept, *J* = 6.1 Hz, 1H, $CH(CH_3)_2$), 4.10-4.08 (d,

J = 8.0 Hz, 1H, C<u>H</u>OCH3), 3.31 (s, 3H, OCH3), 1.55 (s, 9H, C(C<u>H</u>3)3), 1.23-1.22 (d, J = 6.2 Hz, 6H, CH(C<u>H</u>3)2)

^{13}C NMR (151 MHz, DMSO-d6): δ = 170.9 (CO), 168.7 (CO), 166.2 (CO), 165.5 (CO), 164.6 (CO), 164.5 (CO), 164.5 (CO), 150.0 (C_{Ar}), 143.2 (C_{Ar}), 142.1 (C_{Ar}), 141.8 (C_{Ar}), 138.7 (C_{Ar}), 134.2 (C_{Ar}), 133.2 (C_{Ar}), 132.5 (C_{Ar}), 130.0 (C_{Ar}), 129.4 (C_{Ar}), 128.9 (C_{Ar}), 128.6 (C_{Ar}), 128.6 (C_{Ar}), 128.3 (C_{Ar}), 128.2 (C_{Ar}), 126.1, (C_{Ar}), 124.9 (C_{Ar}), 123.3 (C_{Ar}), 119.6 (C_{Ar}), 118.8 (C_{Ar}), 118.8 (C_{Ar}), 118.3 (CN), 114.1 (C_{Ar}), 113.5-110.4 (t, J = 237.5 Hz, CHF_2), 80.4 (<u>C</u>(CH3)3), 80.0 (<u>C</u>HOCH3), 77.0 (<u>C</u>H(CH3)2), 57.6 (OCH3), 55.8 (CHNH), 27.9 (C(<u>C</u>H3)3), 21.9 (CH(<u>C</u>H3)2)

HRMS (ESI): calculated for $[M+Na]^+$: 954.3250; found: 954.3248.

*Synthesis of tert-butyl 4-(2-(allyloxy)-4-(4-((2S,3R)-4-amino-2-((tert-butoxycarbonyl)amino)-3-methoxy-4-oxobutanamido)benzamido)-3-isopropoxy-N-methylbenzamido)benzoate (**S48**)*

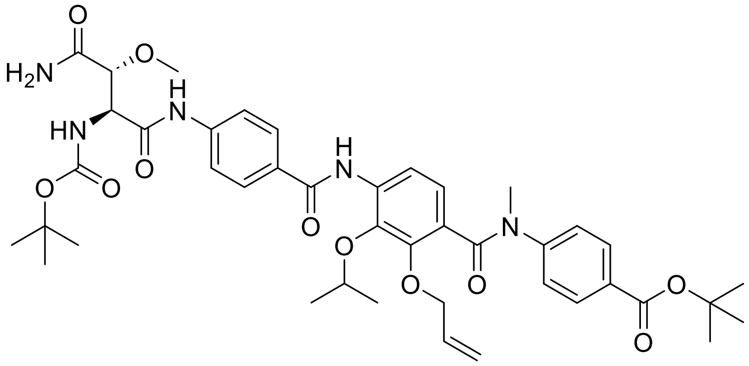

tert-Butyl 4-(2-(allyloxy)-4-(4-aminobenzamido)-3-isopropoxy-*N*-methylbenzamido)benzoate **84** (100 mg, 0.18 mmol), the *N*-Boc protected amino acid (80.0 mg, 0.30 mmol, 1.70 Eq) and EEDQ (71.0 mg, 0.29 mmol, 1.60 Eq) were dissolved in precooled $CHCl_3$ (1 mL). The mixture was stirred for 18 h while warming to RT. The mixture was concentrated and the crude product was purified by column chromatography (dry load, wash with 20% Et_2O in DCM, elution with 3% MeOH in DCM) to furnish the product (75.1 mg, 52%) as colorless amorphous solid.

$[\alpha]_D^{23}$ = -0.5 ° (c 0.1, MeOH);

^{1}H NMR (400 MHz, DMSO-d6): δ = 10.33 (s, 1H, NH), 9.26 (s, 1H, NH), 7.89-7.87 (d, J = 8.7 Hz, 2H, H_{Ar}), 7.77-7.75 (d, J = 8.7 Hz, 2H, H_{Ar}), 7.71-7.70 (d, J = 7.5 Hz, 2H, H_{Ar}), 7.67-7.65 (d, J = 8.4 Hz, 1H, H_{Ar}), 7.44-7.40 (d, J = 14.4 Hz, 2H, NH_2), 7.23 (bs, 2H, H_{Ar}), 7.04-7.02 (d, J = 8.3 Hz, 1H, H_{Ar}), 6.79-6.77 (d, J = 8.3 Hz, 1H, CHN<u>H</u>), 6.06-5.97 (m, 1H, C<u>H</u>CH2), 5.39-5.33 (dq, J = 1.7, 17.2 Hz, 1H, CHC<u>H</u>2), 5.26-5.23 (dq,

J = 1.6, 10.5 Hz, 1H, $CHCH_2$), 4.43-4.42 (d, J = 4.4 Hz, 2H, OCH_2), 4.40-4.36 (t, J = 7.8 Hz, 1H, CHNH), 4.03 (bs, 1H, $CH(CH_3)_2$), 3.84-3.83 (d, J = 7.6 Hz, 1H, $CHOCH_3$), 3.38 (s, 3H, NCH_3), 3.33 (s, 3H, OCH_3), 1.48 (s, 9H, $C(CH_3)_3$), 1.37 (s, 9H, $C(CH_3)_3$), 0.98 (bs, 6H, $CH(CH_3)_2$)

^{13}C NMR (101 MHz, DMSO-d_6): δ = 170.6 (CO), 168.9 (CO), 167.2 (CO), 164.2 (CO), 164.1 (CO), 154.8 (CO), 147.6 (C_{Ar}), 147.3 (C_{Ar}), 142.1 (C_{Ar}), 141.5 (C_{Ar}), 134.2 (C_{Ar}), 134.0 ($CHCH_2$), 129.1 (C_{Ar}), 128.8 (C_{Ar}), 128.5 (C_{Ar}), 128.2 (C_{Ar}), 128.1 (C_{Ar}), 126.4 (C_{Ar}), 122.7 (C_{Ar}), 118.8 (C_{Ar}), 118.4 ($CHCH_2$), 117.1 (C_{Ar}), 80.7 ($C(CH_3)_3$), 80.1 ($CHOCH_3$), 78.7 ($C(CH_3)_3$), 75.2 ($CH(CH_3)_2$), 73.9 (OCH_2), 57.6 (CHNH), 56.6 (OCH_3), 36.5 (NCH_3), 28.1 ($C(CH_3)_3$), 27.7 ($C(CH_3)_3$), 21.8 ($CH(CH_3)_2$)

HRMS (ESI): calculated for $[M+Na]^+$: 826.3639; found: 826.3648.

*Synthesis of tert-butyl 4-(2-(allyloxy)-4-(4-((2S,3R)-4-amino-2-(4-(4-cyanobenzamido)benzamido)-3-methoxy-4-oxobutanamido)benzamido)-3-isopropoxy-N-methylbenzamido)benzoate (**S49**)*

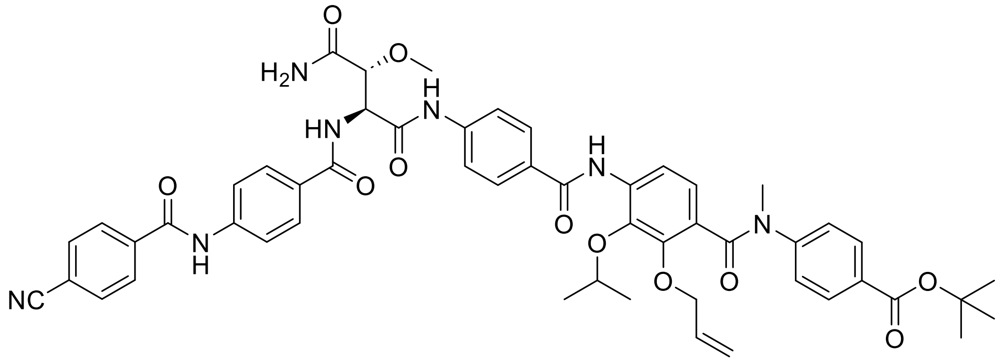

a) *tert*-Butyl 4-(2-(allyloxy)-4-(4-((2S,3R)-4-amino-2-((tert-butoxycarbonyl)amino)-3-methoxy-4-oxobutanamido)benzamido)-3-isopropoxy-N-methylbenzamido)benzoate **S48** (71.3 mg, 0.09 mmol, 1.0 Eq) was dissolved in HCl (4 M in 1,4-dioxane, 2.20 mL, 8.87 mmol, 100.0 Eq) at 0°C. The mixture was warmed up to RT and stirred for 15 min. The solution was transferred to a stirred suspension of EtOAc (200 mL) and a sat. $NaHCO_3$ solution (200 mL). The aq. phase was extracted with EtOAc (3x100 mL) and the combined organic phases were washed with brine, dried over $MgSO_4$, filtered and concentrated under reduced pressure. The crude product was used in the next step without further purification.

b) The crude product (62.4 mg, 0.09 mmol) was dissolved in DCM (1 mL) and NMM (20 µL, 0.18 mmol, 2.00 Eq) was added. The AB-fragment (25.3 mg, 0.09 mmol, 1.00 Eq) was added at 0°C and the suspension was stirred for 18 h while warming to

RT. The mixture was concentrated. The crude product was purified by column chromatography (dry load, 5% MeOH in DCM) to furnish the product (39.1 mg, 46%) as beige amorphous solid, which contained minor impurities of a diastereomer.

The *N*-methyl group is not visible in the ^{13}C NMR spectrum and may appear underneath the solvent peak.

R*f* (5% MeOH in DCM) = 0.54

^{1}H NMR (600 MHz, DMSO-d$_6$): δ = 10.71 (s, 1H, NH), 10.53 (s, 1H, NH), 9.26 (s, 1H, NH), 8.45-8.43 (d, *J* = 8.1 Hz, 1H, CHN<u>H</u>), 8.13-8.12 (d, *J* = 8.6 Hz, 2H, H$_{Ar}$), 8.05-8.04 (d, *J* = 8.6 Hz, 2H, H$_{Ar}$), 7.91-7.87 (m, 5H, H$_{Ar}$), 7.80-7.78 (d, *J* = 8.8 Hz, 2H, H$_{Ar}$), 7.71 (bs, 2H, H$_{Ar}$), 7.66-7.65 (d, *J* = 8.2 Hz, 1H, H$_{Ar}$), 7.53-7.47 (d, *J* = 37.9 Hz, 2H, NH$_2$), 7.22 (bs, 2H, H$_{Ar}$), 7.04-7.02 (d, *J* = 8.2 Hz, 1H, H$_{Ar}$), 6.05-5.98 (m, 1H, C<u>H</u>CH$_2$), 5.38-5.34 (dq, *J* = 1.7, 17.2 Hz, 1H, CHC<u>H</u>$_2$), 5.25-5.23 (dq, *J* = 1.7, 10.5 Hz, 1H, CHC<u>H</u>$_2$), 4.91-4.89 (t, *J* = 8.1 Hz, 1H, C<u>H</u>NH), 4.42 (bs, 2H, OCH$_2$), 4.08-4.07 (d, *J* = 8.1 Hz, 1H, C<u>H</u>OCH$_3$), 4.03 (bs, 1H, C<u>H</u>(CH$_3$)$_2$), 3.38 (s, 3H, NCH$_3$), 3.30 (s, 3H, OCH$_3$), 1.48 (s, 9H, C(CH$_3$)$_3$), 0.99 (bs, 6H, CH(C<u>H</u>$_3$)$_2$)

^{13}C NMR (151 MHz, DMSO-d$_6$): δ = 170.9 (CO), 168.7 (CO), 167.2 (CO), 165.4 (CO), 164.5 (CO), 164.2 (CO), 164.1 (CO), 147.6 (C$_{Ar}$), 147.3 (C$_{Ar}$), 142.0 (C$_{Ar}$), 141.8 (C$_{Ar}$), 141.5 (C$_{Ar}$), 138.7 (C$_{Ar}$), 134.2 (C$_{Ar}$), 134.0 (<u>C</u>HCH$_2$), 132.5 (C$_{Ar}$), 130.0 (C$_{Ar}$), 129.1 (C$_{Ar}$), 128.9 (C$_{Ar}$), 128.8 (C$_{Ar}$), 128.6 (C$_{Ar}$), 128.6 (C$_{Ar}$), 128.3 (C$_{Ar}$), 128.2 (C$_{Ar}$), 128.1 (C$_{Ar}$), 126.4 (C$_{Ar}$) 122.7 (C$_{Ar}$), 119.6 (C$_{Ar}$), 118.9 (C$_{Ar}$), 118.3 (CH<u>C</u>H$_2$), 117.1 (CN), 114.0 (C$_{Ar}$), 80.7 (<u>C</u>(CH$_3$)$_3$), 80.0 (<u>C</u>HOCH$_3$), 75.1 (<u>C</u>H(CH$_3$)$_2$), 73.9 (OCH$_2$), 57.7 (CHNH), 55.7 (OCH$_3$), 27.7 (C(<u>C</u>H$_3$)$_3$), 21.8 (CH(<u>C</u>H$_3$)$_2$)

HRMS (ESI): calculated for [M+Na]$^+$: 974.3701; found: 974.3693.

*Synthesis of tert-butyl 4-(4-(4-((2S,3R)-4-amino-2-(4-(4-cyanobenzamido)benzamido)-3-methoxy-4-oxobutanamido)benzamido)-2-hydroxy-3-isopropoxybenzamido)benzoate (**S50**)*

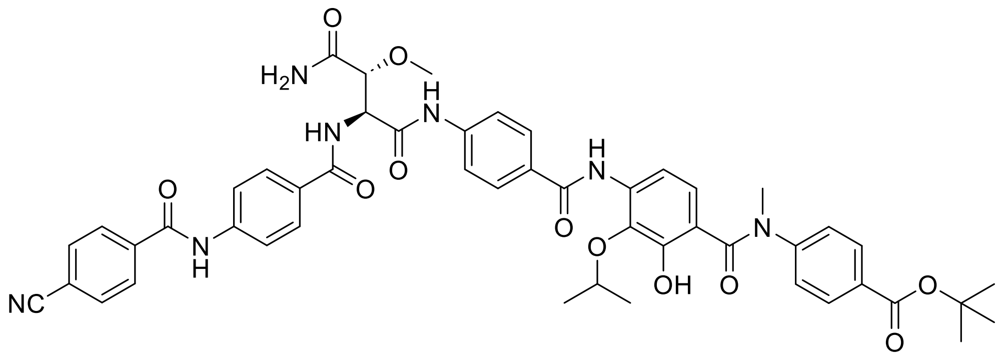

The precursor **S49** (35.9 mg, 0.04 mmol, 1.0 Eq) was dissolved in THF (1.7 mL). Aniline (11 µL, 0.12 mmol, 3.30 Eq) and $Pd(PPh_3)_4$ (4.4 mg, 4 µmol, 0.10 Eq) were added subsequently and the resulting mixture was stirred at RT for 2 h. The mixture was concentrated onto silica. The crude product was purified by column chromatography (dry load, 5% MeOH in DCM) to furnish the product (19.6 mg, 0.02 mmol, 57%) as beige amorphous solid.

R_f (5% MeOH in DCM) = 0.22

$[\alpha]_D^{21}$ = +29.2 ° (c 1.0, MeOH)

^{1}H NMR (600 MHz, DMSO-d_6): δ = 10.71 (s, 1H, OH), 10.52 (s, 1H, NH), 9.30 (s, 1H, NH), 9.28 (s, 1H, NH), 8.44-8.43 (d, J = 8.1 Hz, 1H, CHN<u>H</u>), 8.13-8.12 (d, J = 8.6 Hz, 2H, H_{Ar}), 8.05-8.04 (d, J = 8.6 Hz, 2H, H_{Ar}), 7.91-7.87 (m, 5H, H_{Ar}), 7.79-7.78 (d, J = 8.9 Hz, 2H, H_{Ar}), 7.73-7.71 (d, J = 8.6 Hz, 2H, H_{Ar}), 7.53-7.46 (d, J = 39.2 Hz, 2H, NH_2), 7.27-26 (d, J = 8.6 Hz, 2H, H_{Ar}), 7.25-7.24 (d, J = 8.5 Hz, 1H, H_{Ar}), 6.88-6.86 (d, J = 8.4 Hz, 1H, H_{Ar}), 4.91-4.88 (t, J = 8.1 Hz, 1H, C<u>H</u>NH), 4.09-4.07 (d, J = 8.1 Hz, 1H, C<u>H</u>OCH$_3$), 4.07-4.01 (sept, J = 6.1 Hz, 1H, C<u>H</u>(CH$_3$)$_2$), 3.37 (s, 3H, NCH$_3$), 3.30 (s, 3H, OCH$_3$), 1.49 (s, 9H, C(CH$_3$)$_3$), 1.02-1.01 (d, J = 6.1 Hz, 6H, CH(C<u>H</u>$_3$)$_2$)

^{13}C NMR (151 MHz, DMSO-d_6): δ = 170.9 (CO), 168.6 (CO), 168.0 (CO), 165.4 (CO), 164.5 (CO), 164.3 (CO), 164.0 (CO), 147.8 (C_{Ar}), 147.3 (C_{Ar}), 141.9 (C_{Ar}), 141.8 (C_{Ar}), 138.7 (C_{Ar}), 137.5 (C_{Ar}), 133.4 (C_{Ar}), 132.5 (C_{Ar}), 129.2 (C_{Ar}), 128.9 (C_{Ar}), 128.8 (C_{Ar}), 128.6 (C_{Ar}), 128.3 (C_{Ar}), 126.3 (C_{Ar}), 123.0 (C_{Ar}), 121.7 (C_{Ar}), 119.6 (C_{Ar}), 118.8 (C_{Ar}) 118.3 (CN), 114.5 (C_{Ar}), 114.0 (C_{Ar}), 80.7 (<u>C</u>(CH$_3$)$_3$), 80.0 (<u>C</u>HOCH$_3$), 74.6 (<u>C</u>H(CH$_3$)$_2$), 57.7 (CHNH), 55.7 (OCH$_3$), 36.8 (NCH$_3$). 27.7 (C(<u>C</u>H$_3$)$_3$), 21.7 (CH(<u>C</u>H$_3$)$_2$)

HRMS (ESI): calculated for $[M+Na]^+$: 934.3388; found: 934.3394.

*Synthesis of tert-butyl (S)-4-((2-(allyloxy)-4-(4-(2-aminopent-4-ynamido)benzamido)-3-isopropoxybenzyl)(tert-butoxycarbonyl)amino)benzoate (**S51**)*

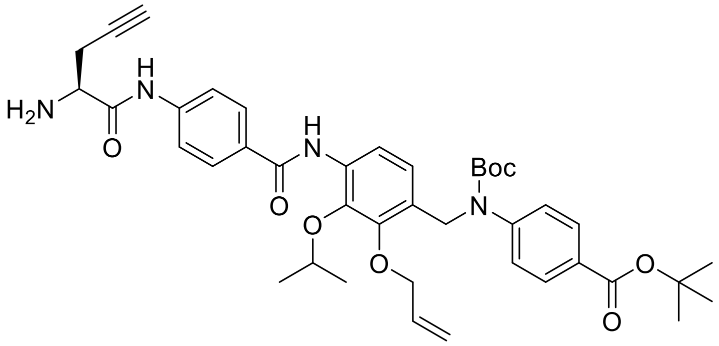

a) *tert*-butyl 4-((2-(allyloxy)-4-(4-aminobenzamido)-3-isopropoxybenzyl)(tert-butoxycarbonyl)amino)benzoate **54** (60 mg, 0.09 mmol, 1.0 Eq) and (S)-2-((((9H-fluoren-9-yl)methoxy)carbonyl)amino)pent-4-ynoic acid (*N*-Fmoc-Pra-OH, 45 mg, 0.13 mmol, 1.4 Eq) were added to a dry flask and were further dried under high vacuum. Dry pyridine (24 µL, 3.1 Eq) and dry EtOAc (0.2 mL) were added under nitrogen atmosphere. The reaction mixture was cooled down to 0°C. T3P solution (0.12 mL, 50 wt % in EtOAc, 2.1 Eq) was added very slowly while keeping the temperature below 0°C. The reaction was stirred at 0°C and controlled by LCMS. After completion, the reaction was quenched with brine (14 mL) and extracted with EtOAc (3x6 mL). The combined organic phases were washed with brine and dried. The crude product was purified by chromatography. A yellow foam was obtained (65.7 mg crude) that was used directly in next step.

b) The crude product (69.2 µmol, 1.0 Eq) was dissolved in MeCN (0.4 mL) and diethylamine (0.11 mL, 15 Eq) at 0°C and stirred for 1 h. The solvent was evaporated under reduced pressure. MeCN (1 mL) was added to the residue and the solvent was removed again. This was repeated twice. n-heptane (1 mL) was added and the solvent was removed under reduced pressure. The crude product was purified by RP HPLC. A colorless solid was obtained, yield: 32.0 mg, 52 % o2s.

^{1}H NMR (700 MHz, $CDCl_3$): δ = 9.79 (br s, 1H), 8.59 (s, 1H), 8.23 (d, 1H, J = 8.6 Hz), 7.87 – 7.84 (m, 4H), 7.75 (d, 2H, J = 8.7 Hz), 7.22 (d, 2H, J = 8.5 Hz), 7.02 (d, 1H, J = 8.6 Hz), 5.98 (ddt, 1H, J = 5.7 Hz, 10.6 Hz, 16.9 Hz), 5.31 (dq, 1H, J = 1.5 Hz, 17.2 Hz), 5.20 (dq, 1H, J = 1.4 Hz, 10.4 Hz), 4.91 (s, 2H), 4.60 (hept., 1H, J = 6.2 Hz), 4.37 (d, 2H, J = 5.7 Hz), 3.67 (dd, 1H, J = 4.5 Hz, 7.1 Hz), 2.79 (dddd, 2H, J = 2.6 Hz, 5.9 Hz, 10.0 Hz, 17.0 Hz), 2.09 (t, 1H, J = 2.6 Hz), 1.56 (s, 9H), 1.43 (s, 9H), 1.25 (dd, 6H, J = 2.5 Hz, 6.2 Hz)

^{13}C NMR (176 MHz, $CDCl_3$): δ = 171.4, 165.4, 164.3, 154.4, 148.7, 146.5, 140.9, 139.3, 133.8, 132.8, 130.5, 129.9, 128.9, 128.1, 127.3, 125.6, 123.2, 119.3, 117.8, 115.2, 81.2, 81.0, 80.0, 75.6, 73.3, 71.8, 54.0, 48.3, 28.4, 28.3, 24.9, 22.8

Synthesis of methoxy-cystobactamid (tBu protected) ***(S52)***

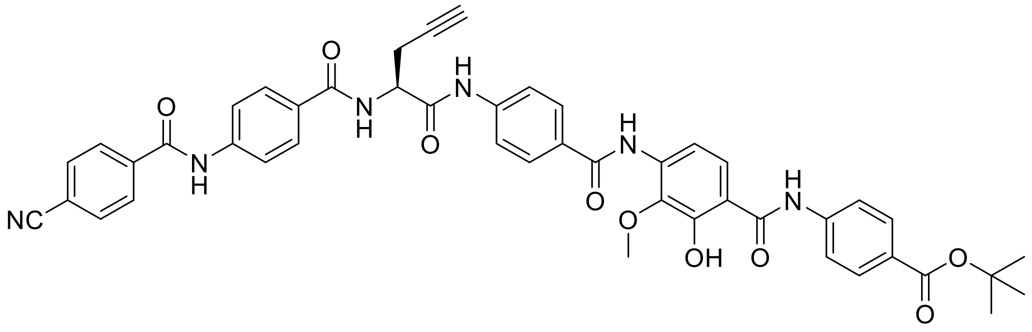

a) The protected cystobactamid precursor was obtained according to general procedure B, involving reaction of the CDE-fragment **S40** (80 mg, 0.15 mmol, 1.0 Eq), the standard AB-central AA-fragment **88** (61 mg, 1.1 Eq), dry pyridine (62 µL, 5.0 Eq) and T3P-solution (0.18 mL, 2.0 Eq) in dry EA (1.5 mL) for 2 h 15 min.

b) Modified from[18]: The crude product, meldrum acid (71 mg, 3.2 Eq) and $Pd(PPh_3)_4$ (8.9 mg, 0.05 Eq) were solved in dry THF (2.3 mL) under Argon atmosphere. The reaction was stirred for 1 h at RT, monitored by LCMS and solvents were then removed u.r.p.. The material was purified by FCC (solid loading, 150x reactant mass, PE/EA/AcOH, 60/35/5->55/40/5->50/45/5->35/60/5). Product fractions were identified by LCMS. Solvents were removed u.r.p. and by coevaporation with heptane from the purified product to give a yellow solid, yield: 96 mg, 76% o2s, NMR:68% o2s.

^{1}H NMR (500 MHz, Acetone): δ = 10.99 (br s, 1H), 10.06 (s, 1H), 9.90 (s, 1H), 9.03 (s, 1H), 8.19 (d, J = 8.7, 2H), 8.15 (d, J = 7.3, 1H), 8.04 – 7.94 (m, 11H), 7.92 – 7.86 (m, 4H), 7.81 (d, J = 8.9, 1H), 5.00 – 4.93 (m, 1H), 4.01 (s, 3H), 2.99 – 2.85 (m, 2H), 2.51 (t, J = 2.7, 1H), 1.58 (s, 9H).

^{13}C NMR (126 MHz, Acetone): δ = 172.2, 170.2 – 170.0 (m), 169.6, 167.3 – 167.1 (m), 165.6, 165.3 – 165.1 (m), 143.1, 143.0, 142.9, 139.9, 139.8, 133.3, 131.0, 130.6, 130.2, 129.4, 129.3, 129.3, 129.2, 128.0, 123.9, 120.9, 120.4, 120.3, 120.1, 120.0, 118.7, 115.9, 109.4, 81.0, 80.8, 72.4, 60.7, 54.5, 28.3, 22.2.

HRMS (ESI): calculated for $[M+H]^+$: 821.2930, found: 821.2927.

Synthesis of diallyl protected triazol cystobactamid ***(S53)***

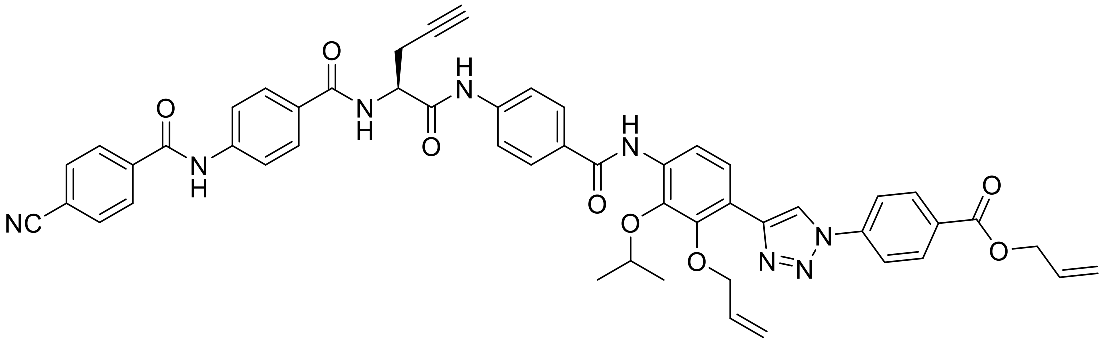

The protected cystobactamid precursor was obtained according to general procedure B, involving reaction of the CDE-fragment **65** (51 mg, 92 µmol, 0.95 Eq), the standard AB-central AA-fragment **88** (35 mg, 97 µmol, 1.0 Eq), dry pyridine (after 30 min, 39 µL, 5.0 Eq) and T3P-solution (0.12 mL, 2.0 Eq) in dry EA (1.5 mL) for 3 h.

The material was purified with FCC (solid loading, 100x reactants mass, PE/EA, 50/50) and a mixed fraction was purified again with FCC (solid loading, PE/EA, 60/40). Total yield: 47 mg, 54%.

^{1}H NMR (700 MHz, Acetone): δ = 9.99 (s, 1H, H-NCO), 9.85 (s, 1H, H-NCO), 8.93 (s, 1H, H-NCO), 8.88 (s, 1H), 8.38 (d, J = 8.7, 1H, H-$C_{Ar,D}$), 8.28 (d, J = 8.9, 2H), 8.19 (d, J = 8.8, 2H), 8.11 (d, J = 8.9, 2H), 8.09 (d, J = 7.9, 1H, H-NCO), 8.06 (d, J = 8.7, 1H, H-$C_{Ar,D}$), 8.02 (d, J = 8.9, 2H), 8.00 (d, J = 8.9, 2H), 7.98 – 7.96 (m, 2H), 7.95 (d, J = 6.6, 2H), 7.90 (d, J = 8.9, 2H), 6.20 (ddt, J = 17.3, 10.5, 5.6, 1H), 6.12 (ddt, J = 17.3, 10.5, 5.6, 1H), 5.51 (dq, J = 17.2, 1.7, 1H), 5.47 (dq, J = 17.2, 1.6, 1H), 5.32 – 5.29 (m, 2H), 4.97 (td, J = 7.6, 6.3, 1H), 4.88 (dt, J = 5.6, 1.5, 2H), 4.79 (hept, J = 6.2, 1H), 4.66 (dt, J = 5.6, 1.5, 2H), 2.96 (ddd, J = 17.0, 6.3, 2.7, 1H), 2.89 (ddd, J = 17.0, 7.6, 2.7, 1H), 2.51 (t, J = 2.7, 1H), 1.39 (d, J = 6.1, 6H).

^{13}C NMR (176 MHz, Acetone): δ = 170.1, 167.3, 165.5, 165.2, 164.7, 149.0, 144.6, 143.1, 143.0, 141.5, 141.4, 139.9, 135.3, 135.1, 133.5, 133.3, 132.1, 130.9, 130.6, 130.3, 129.3 (2x), 128.9, 123.1, 121.3 (2x), 120.8, 120.4, 120.2, 118.7, 118.4, 118.1, 117.1, 115.9, 80.7, 76.9, 73.7, 72.4, 66.3, 54.5, 22.9, 22.2.

HRMS (ESI): calculated for $[M+H]^+$: 897.3355, found: 897.3339.

Synthesis of tert-butyl (S)-4'-(4-(2-aminopent-4-ynamido)benzamido)-3'-isopropoxy-2'-(methoxymethoxy)-[1,1'-biphenyl]-4-carboxylate ***(S54)***

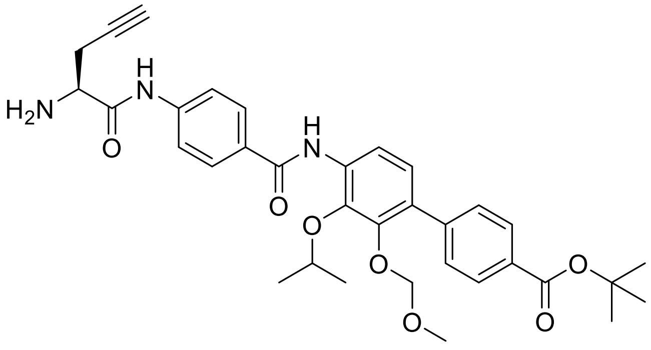

(S)-2-((((9H-fluoren-9-yl)methoxy)carbonyl)amino)pent-4-ynoic acid (*N*-Fmoc-Pra-OH, 69mg, 0.21 mmol, 1.8 Eq) and *tert*-butyl 4'-(4-aminobenzamido)-3'-isopropoxy-2'-(methoxymethoxy)-[1,1'-biphenyl]-4-carboxylate **68** (57.5 mg, 0.11 mmol, 1.0 Eq) were added to a dry flask further dried under high vacuum. Dry DCM (0.4 mL) was added under nitrogen atmosphere and the mixture was cooled down to 0 °C. 51.0 mg EEDQ (51 mg, 0.21 mmol, 1.8 Eq) was added to the stirring solution under nitrogen atmosphere. The reaction was kept at 0 °C for 30 min and subsequently warmed up to room temperature. The reaction was stirred overnight and controlled with LCMS. After completion, EtOAc (4 mL) was added. The organic phase was washed 3 times with a mixture of HCl (1 M, 0.4 mL) and brine (1.6 mL). The organic phase was concentrated under reduced pressure. The crude residue was dissolved in MeCN (0.9 mL) and diethylamine (0.4 mL, 3.9 mmol, 34 Eq) at 0 °C and stirred for 1 h and controlled over LCMS. The solvent was evaporated under reduced pressure and coevapoated with MeCN 3 times. The crude product was purified by HPLC. A faint yellow solid was obtained, yield: 42.8 mg (58 % o2s).

^{1}H NMR (500 MHz, $CDCl_3$): δ = 9.79 (s, 1H), 8.69 (s, 1H), 8.38 (d, 1H, J = 8.6 Hz), 8.02 (d, 2H, J = 8.5 Hz), 7.91 (d, 2H, J = 8.7 Hz), 7.78 (d, 2H, J = 8.7 Hz), 7.61 (d, 2H, J = 8.5 Hz), 7.14 (d, 1H, J = 8.6 Hz), 4.86 (s, 2H), 4.78 (hept., 1H, J = 6.1 Hz), 3.67 (dd, 1H, J = 4.5 Hz, 7.4 Hz), 3.03 (s, 3H), 2.80 (dddd, 2H, J = 2.7 Hz, 5.9 Hz, 10.1 Hz, 17.0 Hz), 2.09 (t, 1H, J = 2.6 Hz), 1.62 (s, 9H), 1.38 (dd, 6H, J = 1.6 Hz, 6.2 Hz).

^{13}C NMR (126 MHz, $CDCl_3$): δ = 171.4, 165.9, 164.3, 147.0, 142.7, 141.0, 139.9, 133.7, 131.4, 130.6, 130.4 129.6, 129.4, 128.2 125.7, 119.4, 115.8, 99.0, 81.2, 80.1, 76.3, 71.8, 57.5, 54.1, 28.4, 24.9, 23.0.

Synthesis of 1-allyl 5-methyl (S)-2-(4-(4-(2-((((9H-fluoren-9-yl)methoxy)carbonyl)amino)pent-4-ynamido)benzamido)-2-(allyloxy)-3-isopropoxyphenyl)-benzo[d]imidazole-1,5-dicarboxylate ***(S55)***

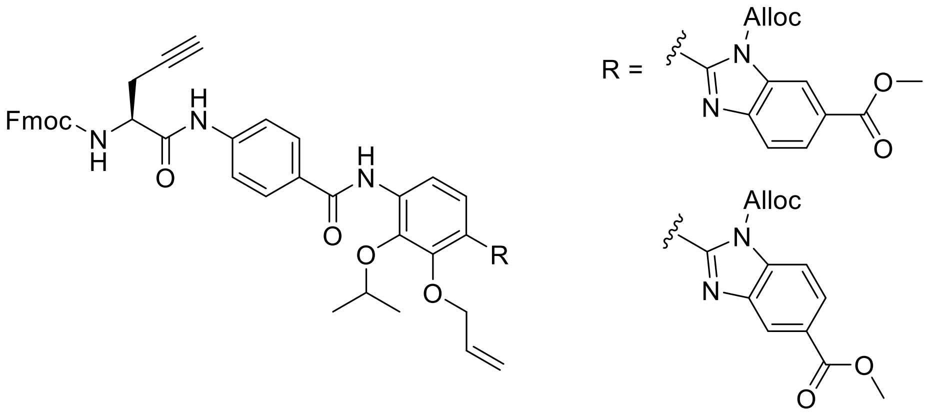

(S)-2-((((9H-fluoren-9-yl)methoxy)carbonyl)amino)pent-4-ynoic acid (*N*-Fmoc-Pra-OH, 48 mg, 0.14 mmol, 1.4 Eq) and a mixture **S43** of crude 1-allyl 5-methyl 2-(2-(allyloxy)-4-(4-aminobenzamido)-3-isopropoxyphenyl)-benzo[d]imidazole-1,5-dicarboxylate and 1-allyl 6-methyl 2-(2-(allyloxy)-4-(4-aminobenzamido)-3-isopropoxyphenyl)-1H-benzo[d]imidazole-1,6-dicarboxylate (60 mg, 0.1 mmol, 1 Eq) were added to a dry flask and were further dried at reduced pressure. Dry pyridine (25 µL, 0.31 mmol, 3 eq) and dry EtOAc (0.3 mL) were added under nitrogen atmosphere. The reaction mixture was cooled down to 0 °C. T3P solution (50 wt % in ethyl acetate, 0.14 mL, 2.3 Eq) was added very slowly while keeping the temperature below 0 °C. The reaction was stirred at 0 °C for 4 h and controlled with LCMS. After completion, the reaction was quenched with saturated $NaHCO_3$–solution (4 mL) and brine (12 mL) and extracted with EtOAc (3x6 mL). The combined organic phases were washed with brine and dried under reduced pressure. The crude product was purified by chromatography. A faint yellow solid was obtained. Yield: 128.9 mg (83 %).

^{1}H NMR (700 MHz, $CDCl_3$): δ = 8.76 (d, 1H, J = 8.0 Hz), 8.70 (dd, 0.5 H, J = 0.5 Hz, 1.6 Hz), 8.52 (d, 0.5 H, J = 1.1 Hz), 8.48 (dd, 1H, J = 5.3 Hz, 8.5 Hz), 8.13 (dd, 0.5 H, J = 1.6 Hz, 8.6 Hz), 8.12 (dd, 0.5 H, J = 1.6 Hz, 8.4 Hz), 8.04 – 8.02 (m, 0.5 H), 7.89 (d, 2H, J = 8.6 Hz), 7.87 (d, 0.5 H, J = 8.4 Hz), 7.78 – 7.75 (m, 2H), 7.69 (dd, 2H, J = 1.6 Hz, 8.6 Hz), 7.61 – 7.58 (m, 2H), 7.54 (d, 0.5 H, J = 8.6 Hz), 7.52 (d, 0.5 H, J = 8.5 Hz), 7.40 (t, 2H, J = 7.4 Hz), 7.32 – 7.28 (m, 2H), 5.89 – 5.81 (m, 1H), 5.66 – 5.60 (m, 1H), 5.25 – 5.17 (m, 2H), 5.03 (ddd, 1H, J = 1.5 Hz, 6.2 Hz, 17.1 Hz), 4.97 (ddd, 1H, J = 1.2 Hz, 4.2 Hz, 10.4 Hz), 4.81 (d, 2H, J = 16.3 Hz), 4.72 – 4.66 (m, 1H), 4.56 – 4.48

(m, 3H), 4.28 (br s, 1H), 4.24 (t, 2H, J = 6.7 Hz), 3.97 (d, 3H, J = 1.7 Hz), 2.95 – 2.70 (m, 2H), 2.18 – 2.16 (m, 1H), 1.35 (br s, 6H)

Final cystobactamids

Preparative HPLC and NMR analysis:

the crude product was solved in THF and a NH_4HCO_3-solution (10 mM) (<2 mL each). If a precipitate was observed DMSO or NaOH (1 M) was added dropwise until it was solved. The solution was then injected manually. After purification and LCMS control of the product fractions, the fractions were combined. The solvent was removed after freezing in liquid nitrogen and lyophillization over at least 2 days. Afterwards, a sample of the product was solved in d6-DMSO for NMR measurement. In addition to the final yield, the purity is also given, if it was significantly lower than 95%. For the final cystobactamids, NMR assignments were verified by 2D NMR spectroscopy.

Preparation for MIC-testing: After NMR analysis, the concentration of the NMR-solution was adjusted to 5.0 mg/mL with d6-DMSO. Details for MIC-testing are listed in biological section.

General procedure **B** (coupling between the standard AB-central AA-fragment and CDE-fragments):

The CDE-fragment and the standard AB-central AA-fragment **88** were suspended under Argon atmosphere in dry EA and pyridine and T3P (50% solution in EA) were added. The reaction was stirred at RT for the given amount of time while being screened by TLC or LCMS. Clarification of the original solution was often observed. The reaction solution was diluted between EA (20-30 mL) and an adequate amount of HCl (0.1-1 M, 15-30 mL) and the aqueous phase was extracted with EA (2x15-20 mL). The combined organic phases were dried over Na_2SO_4 and the solvent was removed u.r.p. to give the crude product or intermediate. If a workup was omitted, only the solvent was removed u.r.p..

General procedure **C** (final deprotection of the *t*Bu-protecting group):

Modified from[18]: The crude material was suspended or solved in dry DCM under Argon atmosphere and TFA was added dropwise at 0°C or RT. The reaction was stirred at RT for the given amount of time while being screened by LCMS. The reaction solution

was diluted with DCM or MeCN and solvents were then removed u.r.p. and by multiple coevaporation with DCM, MeCN or THF. The material was then purified by preparative HPLC by using the given HPLC method. Usually, fluffy colorless solids with low density were obtained.

Synthesis of hydroquinone (ring D) cystobactamid ***(3)***

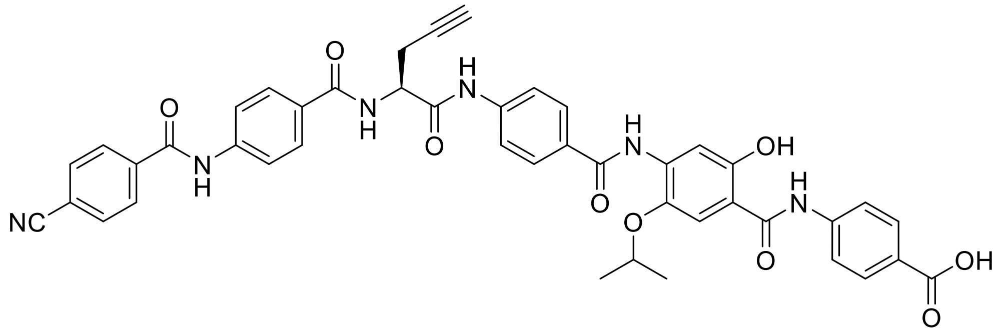

a) Modified from[18]: The cystobactamid-precursor **S44** (20 mg, 22 µmol, 1.0 Eq) and aniline (6.0µL, 3.0 Eq) were solved subsequently in dry THF (0.7 mL). $Pd(PPh_3)_4$ (2.6 mg, 0.1 Eq) was added and the solution was stirred for 1.5 h changing its color from orange to red. TLC showed nearly full reactant conversion after 30 min. The reaction mixture was diluted with acetone and was concentrated on silica followed by FCC (100x reactant mass, PE/EA/AcOH, 50/50/1->60/40/1). Solvent was removed u.r.p. to give a yellowish solid directly used in next step.

b) The final product was obtained according to the general *t*Bu-deprotection procedure using DCM (0.85 mL) and TFA (160 µL, 100 Eq). The reaction was conducted at RT for 1 h and HPLC method 1 was used. Yield: 7.2 mg, 43% o2s.

^{1}H NMR (500 MHz, DMSO): *δ* = 12.76 (br s, 1H), 11.63 (br s, 1H), 10.71 (s, 1H, H-NCO), 10.66 – 10.53 (m, 2H), 9.16 (s, 1H), 8.78 (d, *J* = 7.6, 1H, H-NCO), 8.13 (d, *J* = 8.9, 2H, $H\text{-}C_{Ar}$), 8.05 (d, *J* = 8.5, 2H, $H\text{-}C_{Ar}$), 7.99 – 7.94 (m, 5H, $H\text{-}C_{Ar}$), 7.93 – 7.88 (m, 4H, $H\text{-}C_{Ar}$), 7.85 – 7.81 (m, 4H, $H\text{-}C_{Ar}$), 7.65 (s, 1H, , $H\text{-}C_{Ar,D}$), 4.84 – 4.77 (m, 1H, <u>H</u>-C(NH)$(C)_2$), 4.61 (hept, *J* = 6.3, 1H, $H\text{-}C_{iPr}O$), 2.94 (t, *J* = 2.6, 1H, H-CC), 2.85 – 2.69 (m, 2H, <u>H</u>$_2$C(CCH)), 1.35 (s, 6H, $(H_3C)_2C$).

^{13}C NMR (176 MHz, DMSO): *δ* = 169.7 (C=O), 166.9 (C=O), 165.9 (C=O), 165.5 (C=O), 164.5 (C=O), 164.1 (C=O), 152.8 (C-OH), 142.4 (C_{Ar}), 142.3 (C_{Ar}), 141.7 (C_{Ar}), 140.1 (C_{Ar}), 138.7 (C_{Ar}), 134.1 (C_{Ar}), 132.5 (C_{Ar}-H), 130.3 (C_{Ar}), 128.9 (C_{Ar}), 128.6 (C_{Ar}-

H), 128.4 (C_{Ar}-H), 128.3 (C_{Ar}-H), 125.8 (C_{Ar}), 119.9 (C_{Ar}), 119.5 (2x, C_{Ar}-H), 119.0 (C_{Ar}), 118.3 (CN), 114.7 (C_{Ar}-H), 114.0 (C_{Ar}), 112.1 (C_{Ar}), 109.0 (C_{Ar}-H), 80.6 (C̲CH), 73.2 (C_{iPr}-H), 72.6 (CC̲H), 53.5 (C̲H-$(C)_2$(N)), 21.8 ((C̲H_3)$_2$C), 21.4 (C̲H_2-CCH).

HRMS (ESI): calculated for $[M+H]^+$: 793.2617, found: 793.2606.

Synthesis of fluoro (ring D) cystobactamid ***(4)***

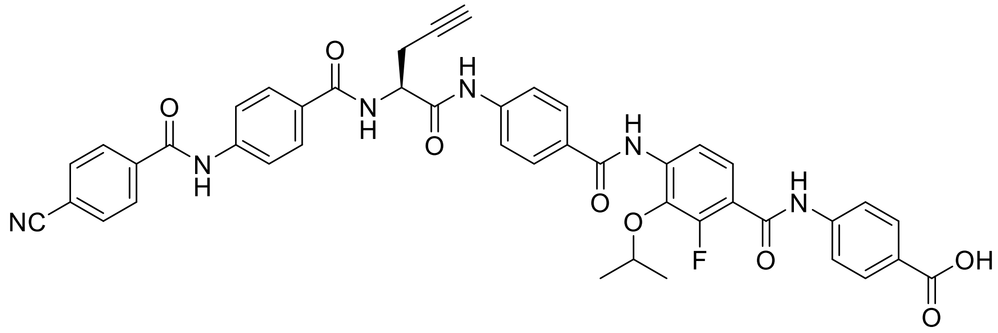

a) The protected cystobactamid precursor was obtained according to general procedure B, involving reaction of the CDE-fragment **S35** (47.2 mg, 93.0 µmol, 1.00 Eq), the standard AB-central AA-fragment **88** (37.0 mg, 1.10 Eq), dry pyridine (38 µL, 5.0 Eq) and T3P-solution (0.11 mL, 2.0 Eq) in dry EA (1.4 mL) for 2.5 h.

b) The final product was obtained according to the general *t*Bu-deprotection procedure using DCM (1.4 mL) and TFA (0.36 mL, 50 Eq). The reaction was conducted at RT for 2 h and HPLC method 1 was used. Yield: 51.3 mg, 69% o2s.

logD$_{7.4}$ = 3.0 ±0.2

^{1}H NMR (700 MHz, DMSO): δ = 12.90 (br s, 1H), 10.73 (s, 1H, H-NCO), 10.66 (s, 1H, H-NCO), 10.64 (s, 1H, H-NCO), 9.67 (s, 1H, H-NCO), 8.85 (d, J = 7.5, 1H, H-NCO), 8.13 (d, J = 8.6, 2H, H-C_{Ar}), 8.05 (d, J = 8.6, 2H, H-C_{Ar}), 7.99 (d, J = 9.0, 2H, H-C_{Ar}), 7.97 (d, J = 8.8, 2H, H-C_{Ar}), 7.93 (d, J = 8.8, 2H, H-C_{Ar}), 7.90 (d, J = 8.8, 2H, H-C_{Ar}), 7.84 – 7.78 (m, 5H, H-C_{Ar}, H-$C_{Ar,D}$), 7.42 (dd, J = 8.4, 7.1, 1H, H-$C_{Ar,D}$), 4.80 (td, J = 8.1, 6.2, 1H, H̲-C(NH)$(C)_2$), 4.40 (hept, J = 5.9, 1H, H-C_{iPr}O), 2.93 (t, J = 2.6, 1H, HCC), 2.84 – 2.74 (m, 2H, H̲$_2$C(CCH)), 1.29 (d, J = 6.0, 6H, $(H_3C)_2$C).

^{13}C NMR (176 MHz, DMSO): δ = 169.7 (C=O), 167.2 (C=O), 165.9 (C=O), 164.5 (C=O), 164.5 (C=O), 162.6 ($C_{Ar,D}$-C=O), 152.7 (d, J = 249.0, $C_{Ar,D}$-F), 142.3 (C_{Ar}), 142.2

(C_{Ar}), 141.7 (C_{Ar}), 138.7 (C_{Ar}), 137.9 (d, J = 13.2, $C_{Ar,D}$-OiPr), 135.8 ($C_{Ar,D}$), 132.5 (C_{Ar}-H), 130.3 (C_{Ar}-H), 128.9 (C_{Ar}), 128.6 (C_{Ar}-H), 128.6 (C_{Ar}-H), 128.4 (C_{Ar}-H), 128.4 (C_{Ar}), 123.4 ($C_{Ar,D}$-H), 121.8 (d, J = 13.5, $C_{Ar,D}$), 119.6 ($C_{Ar,D}$-H), 119.5 (C_{Ar}-H), 118.9 (C_{Ar}-H), 118.8 (C_{Ar}-H), 118.3 (CN), 114.0 (C_{Ar}), 80.7 ($\underline{C}$CH), 77.3 (C_{iPr}-H), 73.2 (C_{iPr}-H, C$\underline{C}$H), 53.6 ($\underline{C}$H-$(C)_2$(N)), 22.3 (($\underline{C}H_3)_2$C), 21.4 ($\underline{C}H_2$-CCH).

^{19}F NMR (471 MHz, DMSO): δ = -130.3.

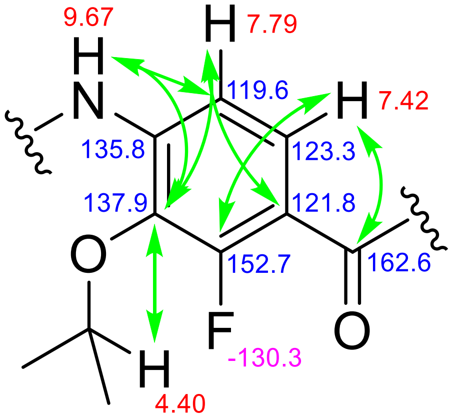

HRMS (ESI): calculated for $[M+H]^+$: 795.2573, found: 795.2574.

*Synthesis of chloro (ring D) cystobactamid **(5)***

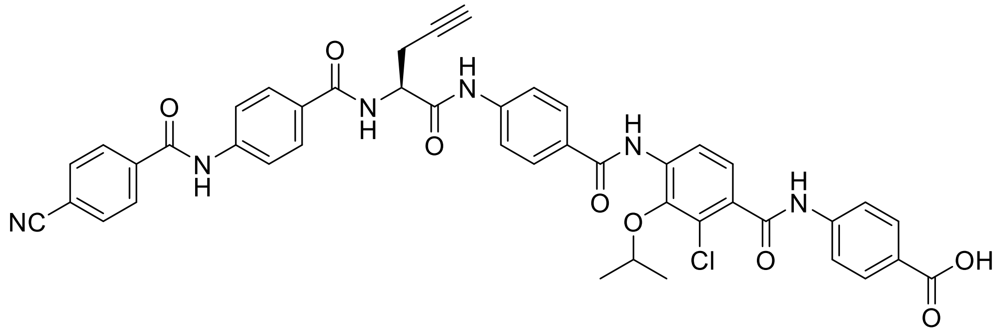

a) The protected cystobactamid precursor was obtained according to general procedure B, involving reaction of the CDE-fragment **93** (63.8 mg, 0.122 mmol, 1.00 Eq), the standard AB-central AA-fragment **88** (48.4 mg, 1.10 Eq), dry pyridine (49 µL, 5.0 Eq) and T3P-solution (0.14 mL, 2.0 Eq) in dry EA (1.8 mL) for 2 h.

b) The final product was obtained according to the general *t*Bu-deprotection procedure using DCM (1.8 mL) and TFA (0.47 mL, 50 Eq). The reaction was conducted at RT for 2 h and HPLC method 1 was used. Yield: 54.9 mg, 56% o2s.

^{1}H NMR (700 MHz, DMSO): δ = 12.78 (br s, 1H), 10.82 (s, 1H, H-NCO), 10.72 (s, 1H, H-NCO), 10.60 (s, 1H, H-NCO), 9.81 (s, 1H, H-$N_{D\text{-}ring}$CO), 8.80 (d, J = 7.5, 1H, H-NCO), 8.13 (d, J = 8.6, 2H, H-C_{Ar}), 8.05 (d, J = 8.4, 2H, H-C_{Ar}), 8.02 (d, J = 8.8, 2H, H-C_{Ar}), 7.97 (d, J = 8.8, 2H, H-C_{Ar}), 7.94 (d, J = 8.6, 2H, H-C_{Ar}), 7.90 (d, J = 8.8, 2H, H-C_{Ar}), 7.85 – 7.79 (m, 5H, H-C_{Ar}, H-$C_{Ar,D}$), 7.38 (d, J = 8.2, 1H, H-$C_{Ar,D}$), 4.80 (td, J = 8.3, 6.2, 1H, $\underline{H}$-C(NH)$(C)_2$), 4.40 (hept, J = 6.1, 1H, H-C_{iPr}O), 2.94 (t, J = 2.7, 1H, HCC), 2.85 – 2.73 (m, 2H, $\underline{H}_2$C(CCH)), 1.26 (d, J = 6.0, 6H, $(H_3C)_2$C).

^{13}C NMR (176 MHz, DMSO): δ = 169.7 (C=O), 167.0 (C=O), 165.9 (C=O), 164.9 ($C_{Ar,D}$-$\underline{C}$=O), 164.5 (C=O), 164.5 (C=O), 147.1 (C_{Ar}-OiPr), 142.7 (C_{Ar}), 142.1 (C_{Ar}), 141.7 (C_{Ar}), 138.7 (C_{Ar}), 134.7 ($C_{Ar,D}$), 134.5 ($C_{Ar,D}$), 132.5 (C_{Ar}-H), 130.4 (C_{Ar}-H), 128.9 (C_{Ar}), 128.6 (C_{Ar}-H), 128.6 (C_{Ar}-H), 128.4 (C_{Ar}-H), 128.3 (C_{Ar}), 126.3 (C_{Ar}), 124.7 ($C_{Ar,D}$), 124.2 ($C_{Ar,D}$-H), 123.2 ($C_{Ar,D}$-H), 119.5 (C_{Ar}-H), 118.8 (C_{Ar}-H), 118.8 (C_{Ar}-H), 118.3 (CN), 114.0 (C_{Ar}), 80.7 ($\underline{C}$CH), 76.9 (C_{iPr}-H), 73.2 (C_{iPr}-H, C$\underline{C}$H), 53.5 ($\underline{C}$H-$(C)_2$(N)), 22.2 ($(\underline{C}H_3)_2$C), 21.4 ($\underline{C}H_2$-CCH).

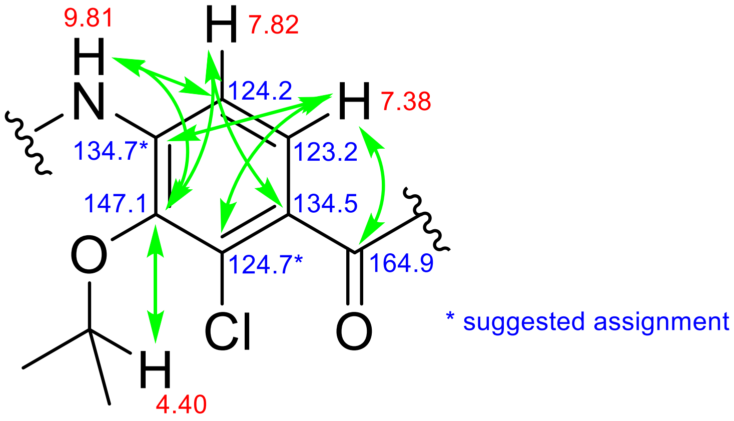

HRMS (ESI): calculated for $[M+H]^+$: 811.2278, found: 811.2278.

Synthesis of bromo (ring D) cystobactamid ***(6)***

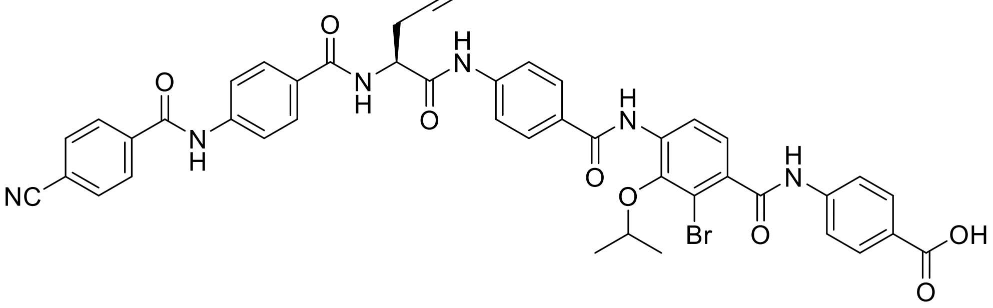

a) The protected cystobactamid precursor was obtained according to general procedure B, involving reaction of the CDE-fragment **S36** (38.3 mg, 67.4 µmol, 1.00 Eq), the standard AB-central AA-fragment **88** (26.8 mg, 1.10 Eq), dry pyridine (27 µL, 5.0 Eq) and T3P-solution (80 µL, 2.0 Eq) in dry EA (1.0 mL) for 2 h.

b) The final product was obtained according to the general *t*Bu-deprotection procedure using DCM (1.0 mL) and TFA (0.26 mL, 50 Eq). The reaction was conducted at RT for 2 h and HPLC method 2 was used. Because the isolation of the targeted compound from byproduct was not successful, the product mixture was separated again with the half amount of the material by using acidic preparative HPLC (method 5, Note: The behavior of the product mixture was modelled before with reverse phase TLC). The product fractions were again collected and lyophillized. Yield (for half of the product): 6.5 mg, 22%.

^{1}H NMR (700 MHz, DMSO): *δ* = 12.78 (br s, 1H), 10.82 (s, 1H, H-NCO), 10.71 (s, 1H, H-NCO), 10.57 (s, 1H, H-NCO), 9.83 (s, 1H H-$N_{D\text{-ring}}$CO), 8.78 (d, *J* = 7.5, 1H, H-NCO), 8.13 (d, *J* = 8.6, 2H, H-C_{Ar}), 8.05 (d, *J* = 8.6, 2H, H-C_{Ar}), 8.02 (d, *J* = 8.8, 2H, H-C_{Ar}), 7.96 (d, *J* = 9.0, 2H, H-C_{Ar}), 7.94 (d, *J* = 8.6, 2H, H-C_{Ar}), 7.90 (d, *J* = 8.8, 2H, H-C_{Ar}), 7.85 – 7.82 (m, 3H, H-C_{Ar}, H-$C_{Ar,D}$), 7.81 (d, *J* = 8.8, 2H, H-C_{Ar}), 7.34 (d, *J* = 8.2, 1H, H-$C_{Ar,D}$), 4.83 – 4.77 (m, 1H, <u>H</u>-C(NH)$(C)_2$), 4.42 (hept, *J* = 6.2, 1H, H-C_{iPr}O), 2.94 (t, *J* = 2.6, 1H, HCC), 2.85 – 2.72 (m, 2H, <u>H</u>$_2$C(CCH)), 1.26 (d, *J* = 6.0, 6H, $(H_3C)_2$C)).

^{13}C NMR (176 MHz, DMSO): *δ* = 169.7 (C=O), 166.9 (C=O), 165.9 ($C_{Ar,D}$-C=O), 165.9 (C=O), 164.5 (C=O), 164.5 (C=O), 148.2 (C_{Ar}-OiPr), 142.9 (C_{Ar}), 142.1 (C_{Ar}), 141.7 (C_{Ar}), 138.7 (C_{Ar}), 136.9 ($C_{Ar,D}$), 134.3 ($C_{Ar,D}$), 132.5 (C_{Ar}-H), 130.4 (C_{Ar}-H), 128.9 (C_{Ar}), 128.6 (C_{Ar}-H), 128.6 (C_{Ar}-H), 128.4 (C_{Ar}-H), 128.3 (C_{Ar}), 126.0 (C_{Ar}) 125.2 ($C_{Ar,D}$-H), 123.2 ($C_{Ar,D}$-H), 119.5 (C_{Ar}-H), 118.8 (C_{Ar}-H), 118.8 (C_{Ar}-H), 118.3 (CN), 115.1 ($C_{Ar,D}$), 114.0 (C_{Ar}), 80.7 (<u>C</u>CH), 76.9 (C_{iPr}-H), 73.2 (C_{iPr}-H, C<u>C</u>H), 53.5 (<u>C</u>H-$(C)_2$(N)), 22.2 ((<u>C</u>H$_3$)$_2$C), 21.4 (<u>C</u>H$_2$-CCH).

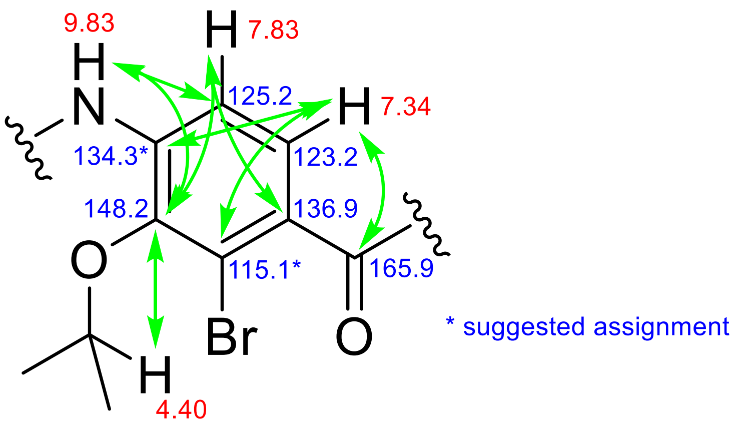

HRMS (ESI): calculated for $[M+H]^+$: 855.1773/ 857.1752, found: 855.1771/ 857.1760.

*Synthesis of Me-oxoquinazoline (ring D) cystobactamid **(45a)***

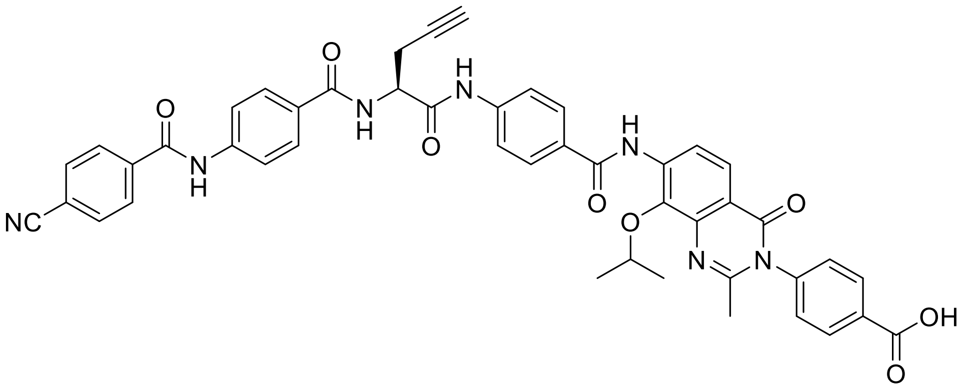

a) The protected cystobactamid precursor was obtained according to general procedure B, involving reaction of the CDE-fragment **71** (6.2 mg, 12 µmol, 1.0 Eq), the standard AB-central AA-fragment **88** (5.7 mg, 1.35 Eq), dry pyridine (4.7 µL, 5.0 Eq) and T3P-solution (14 µL, 2.0 Eq) in dry EA (0.18 mL) for 2 h. An aqueous workup was omitted.

b) The final product was obtained according to the general *t*Bu-deprotection procedure using DCM (0.18 mL) and TFA (45 µL, 50 Eq). The reaction was conducted at 0°C for 2 h and HPLC method 1 was used, including lyophillization over 7 days. Yield: 4.9 mg, 51% o2s.

^{1}H NMR (700 MHz, DMSO): δ = 13.21 (br s, 1H, HOOC), 10.71 (s, 1H, H-NCO), 10.61 (s, 1H, H-NCO), 9.55 (s, 1H, H-NCO), 8.78 (d, J = 7.5, 1H, H-NCO), 8.25 (d, J = 8.7, 1H, H-$C_{Ar,D}$), 8.13 (d, J = 8.7, 2H), 8.11 (d, J = 8.6, 2H), 8.05 (d, J = 8.6, 2H), 7.99 (d, J = 8.9, 2H), 7.97 (d, J = 8.9, 2H), 7.90 (d, J = 9.0, 2H), 7.86 (d, J = 8.7, 1H, H-$C_{Ar,D}$),

7.84 (d, J = 8.9, 2H), 7.60 (d, J = 8.4, 2H), 5.00 (hept, J = 6.1, 1H, H-C_{iPr}O), 4.81 (q, J = 7.7, 1H, H-C(NH)$(C)_2$), 2.94 (t, J = 2.6, 1H, H-CC), 2.85 – 2.73 (m, 2H, H_2C(CCH)), 2.16 (s, 3H, H_3C-C), 1.34 (d, J = 6.2, 6H, $(H_3C)_2C$).

^{13}C NMR (176 MHz, DMSO): δ = 169.7 (C=O), 166.7 (C=O), 166.0 (C=O), 164.5 (C=O), 164.3 (C=O), 160.9 (C=O), 153.1 ($C_{pyrim.}$-CH_3), 142.2 (C_{Ar}), 142.2 (C_{Ar}), 141.7 (C_{Ar}), 141.6 (C_{Ar}), 141.1 (C_{Ar}), 138.7 (C_{Ar}), 136.9 (C_{Ar}), 132.5 (C_{Ar}-H), 130.5 (C_{Ar}-H), 128.9 (C_{Ar}), 128.9 (C_{Ar}-H), 128.6 (C_{Ar}-H), 128.5 (C_{Ar}), 128.4 (C_{Ar}-H), 128.4 (C_{Ar}-H), 121.0 ($C_{Ar,D}$-H), 120.3 ($C_{Ar,D}$-H), 119.5 (C_{Ar}-H), 119.0 (C_{Ar}-H), 118.3 (CN), 117.6 (C_{Ar}), 114.0 (C_{Ar}), 80.6 (CCH), 77.1 (C_{iPr}-H), 73.2 (CCH), 53.5 (CH-$(C)_2$(N)), 24.6 (CH_3-$C_{pyrim.}$), 22.5 ($(CH_3)_2C$), 21.4 (CH_2-CCH).

HRMS (ESI): calculated for $[M+H]^+$: 816.2776, found: 816.2776.

Synthesis of oxoquinazoline (ring D) cystobactamid ***(45b)***

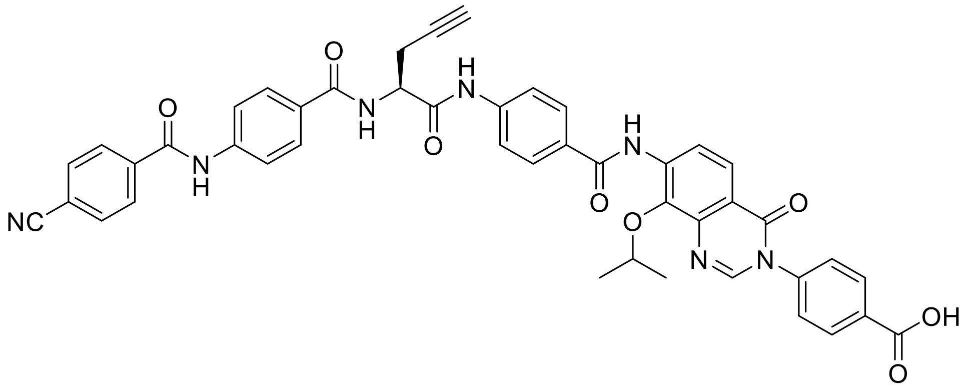

a) The protected cystobactamid precursor was obtained according to general procedure B, involving reaction of the CDE-fragment **75** (28 mg, 50 µmol, 1.0 Eq), the standard AB-central AA-fragment **88** (19.7 mg, 1.10 Eq), dry pyridine (20 µL, 5.0 Eq) and T3P-solution (59 µL, 2.0 Eq) in dry EA (0.75 mL) for 2 h.

b) The final product was obtained according to the general *t*Bu-deprotection procedure using DCM (0.75 mL) and TFA (0.19 mL, 50 Eq). The reaction was conducted at RT for 2 h and HPLC method 1 was used. Yield: 29.5 mg, 74%.

$logD_{7.4}$ = 2.4 ±0.1

^{1}H NMR (500 MHz, DMSO): δ = 13.28 (s, 1H, COOH), 10.72 (s, 1H, H-NCO), 10.63 (s, 1H, H-NCO), 9.62 (s, 1H, H-NCO), 8.81 (d, J = 7.5, 1H, H-NCO), 8.40 (s, 1H, H-

C$_{pyrim.}$), 8.32 (d, *J* = 8.7, 1H, H-C$_{Ar,D}$), 8.13 (d, *J* = 8.7, 2H, H-C$_{Ar}$), 8.10 (d, *J* = 8.5, 2H, H-C$_{Ar}$), 8.05 (d, *J* = 8.5, 2H, H-C$_{Ar}$), 8.03 – 7.94 (m, 5H, H-C$_{Ar}$), 7.90 (d, *J* = 9.0, 2H, H-C$_{Ar}$), 7.85 (d, *J* = 8.9, 2H, H-C$_{Ar}$), 7.68 (d, *J* = 8.5, 2H, H-C$_{Ar}$), 4.99 (hept, *J* = 6.3, 1H, H-C$_{iPr}$O), 4.85 – 4.77 (m, 1H, H-C(NH)(C)$_2$), 2.94 (t, *J* = 2.6, 1H, H-CC), 2.79 (qdd, *J* = 16.6, 7.5, 2.7, 2H, H_2C(CCH)), 1.32 (d, *J* = 6.1, 6H, (H_3C)$_2$C).

^{13}C NMR (126 MHz, DMSO): *δ* = 169.7 (C=O), 166.8 (C=O), 166.0 (C=O), 164.5 (C=O), 164.4 (C=O), 159.5 (C=O), 146.1 (C$_{pyrim.}$-H), 142.6 (C$_{Ar}$), 142.3 (C$_{Ar}$), 141.7 (C$_{Ar}$), 141.6 (C$_{Ar}$), 140.8 (C$_{Ar}$), 138.7 (C$_{Ar}$), 137.3 (C$_{Ar}$), 132.5 (C$_{Ar}$-H), 130.1 (C$_{Ar}$-H), 128.9 (C$_{Ar}$), 128.6 (C$_{Ar}$-H), 128.5 (C$_{Ar}$-H), 128.4 (C$_{Ar}$-H), 127.6 (C$_{Ar}$-H), 121.5 (C$_{Ar,D}$-H), 121.3 (C$_{Ar}$-H), 119.5 (C$_{Ar}$-H), 119.0 (C$_{Ar}$, C$_{Ar}$-H), 118.3 (CN), 114.0 (C$_{Ar}$), 80.7 (CCH), 77.1 (C$_{iPr}$-H), 73.2 (CCH), 53.5 (CH-(C)$_2$(N)), 22.3 ((CH$_3$)$_2$C), 21.4 (CH$_2$-CCH).

HRMS (ESI): calculated for $[M+H]^+$: 802.2620, found: 802.2620.

Synthesis of free amine (ring D) cystobactamid ***(7)***

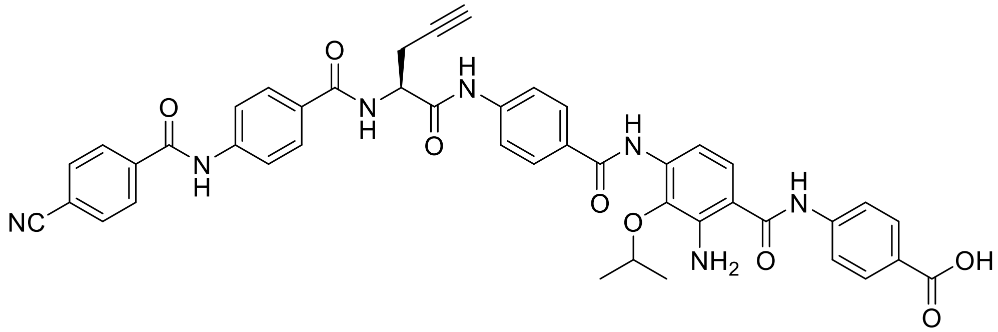

a) The protected cystobactamid precursor was obtained according to general procedure B, involving reaction of the CDE-fragment **S37** (26.5 mg, 52.5 µmol, 1.00 Eq), the standard AB-central AA-fragment **88** (19 mg, 1.0 Eq), dry pyridine (13 µL, 3.0 Eq) and T3P-solution (47 µL, 1.5 Eq) in dry EA (0.80 mL) for 1 h.

b) The final product was obtained according to the general *t*Bu-deprotection procedure using DCM (0.80 mL) and TFA (0.20 mL, 50 Eq). Anisol (11.5 µL, 2.0 Eq) was added as scavenger. The reaction was conducted at RT for 1 h and HPLC method 1 was used. Yield: 21.8 mg, 52% o2s.

^{1}H NMR (700 MHz, DMSO): *δ* = 12.71 (s, 1H, COOH), 10.71 (s, 1H, H-NCO), 10.56 (s, 1H, H-NCO), 10.28 (s, 1H, H-NCO), 9.50 (s, 1H, H-N$_{D\text{-}ring}$CO), 8.78 (d, *J* = 7.5, 1H, H-NCO), 8.13 (d, *J* = 8.4, 2H, H-C$_{Ar}$), 8.05 (d, *J* = 8.4, 2H, H-C$_{Ar}$), 8.00 (d, *J* = 8.8, 2H,

H-C$_{Ar}$), 7.96 (d, J = 8.8, 2H, H-C$_{Ar}$), 7.94 – 7.88 (m, 4H, H-C$_{Ar}$), 7.85 (d, J = 8.8, 2H, H-C$_{Ar}$), 7.80 (d, J = 8.7, 2H, H-C$_{Ar}$), 7.51 (d, J = 8.9, 1H, H-C$_{Ar,D}$-C$_{Ar,D}$-CO), 7.14 (d, J = 8.6, 1H, H-C$_{Ar,D}$-C$_{Ar,D}$-NCO), 6.15 (s, 2H, H$_2$N), 4.80 (q, J = 7.9, 1H, H-C(NH)(C)$_2$), 4.25 (hept, J = 6.3, 1H, H-C$_{iPr}$O), 2.94 (t, J = 2.6, 1H, H-CC), 2.84 – 2.73 (m, 2H, H$_2$C(CCH)), 1.24 (d, J = 6.1, 6H, (H$_3$C)$_2$C).

^{13}C NMR (176 MHz, DMSO): δ = 169.7 (C=O), 167.7 (C$_{Ar,D}$-C=O), 167.0 (C=O), 165.9 (C=O), 164.5 (C=O), 164.2 (C=O), 144.4 (C$_{Ar}$-NH$_2$), 143.3 (C$_{Ar}$), 142.0 (C$_{Ar}$), 141.7 (C$_{Ar}$), 138.7 (C$_{Ar}$), 136.4 (C$_{Ar,D}$-OiPr), 134.7 (C$_{Ar,D}$-NCO), 132.5 (C$_{Ar}$-H), 130.1 (C$_{Ar}$-H), 128.9 (C$_{Ar}$), 128.7 (C$_{Ar}$), 128.6 (C$_{Ar}$-H), 128.4 (C$_{Ar}$-H), 128.4 (C$_{Ar}$-H), 125.4 (C$_{Ar}$), 123.9 (H-C$_{Ar,D}$-C$_{Ar,D}$-C=O), 119.6 (C$_{Ar}$-H), 119.5 (C$_{Ar}$-H), 118.8 (C$_{Ar}$-H), 118.3 (CN), 114.0 (C$_{Ar}$), 112.4 (C$_{Ar,D}$-C=O), 110.9 (H-C$_{Ar,D}$-C$_{Ar,D}$-NCO), 80.7 (CCH), 74.2 (C$_{iPr}$-H), 73.2 (CCH), 53.5 (CH-(C)$_2$(N)), 22.2 ((CH$_3$)$_2$C), 21.4 (CH$_2$-CCH).

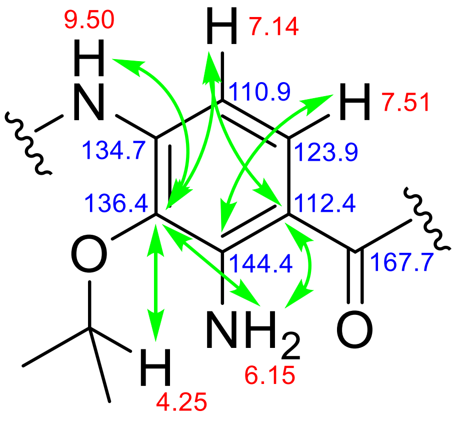

HRMS (ESI): calculated for [M+H]$^+$: 792.2777, found: 792.2775.

Synthesis of pyridine (ring D) cystobactamid ***(8)***

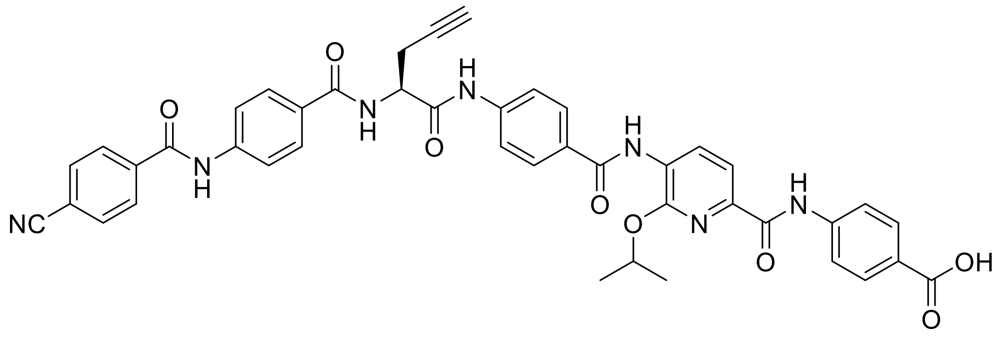

a) The protected cystobactamid precursor was obtained according to general procedure B, involving reaction of the CDE-fragment **S38** (27.1 mg, 55.2 µmol,

1.00 Eq), the standard AB-central AA-fragment **88** (21.4 mg, 1.07 Eq), dry pyridine (13 µL, 3.0 Eq) and T3P-solution (66 µL, 2.0 Eq) in dry EA (0.60 mL) for 1.5 h.

b) The final product was obtained according to the general *t*Bu-deprotection procedure using DCM (0.83 mL) and TFA (0.21 mL, 50 Eq). The reaction was conducted at RT for 1 h and HPLC method 1 was used, including lyophillization over 4 d. Yield: 28.4 mg, 66% o2s.

^{1}H NMR (700 MHz, DMSO): δ = 10.74 (s, 1H, H-NCO), 10.69 (s, 1H, H-NCO), 10.21 (s, 1H, H-NCO), 9.41 (s, 1H, H-N-$C_{Ar,D}$), 8.88 (d, *J* = 7.6, 1H, H-NCO), 8.52 (d, *J* = 8.0, 1H, H-$C_{Ar,D}$), 8.13 (d, *J* = 8.6, 2H, H-C_{Ar}), 8.04 (d, *J* = 8.7, 2H, H-C_{Ar}), 7.99 – 7.94 (m, 6H, H-C_{Ar}), 7.92 (d, *J* = 8.7, 2H, H-C_{Ar}), 7.90 (d, *J* = 8.9, 2H, H-C_{Ar}), 7.84 (d, *J* = 8.8, 2H, H-C_{Ar}), 7.79 (d, *J* = 7.9, 1H, H-$C_{Ar,D}$), 5.78 (hept, *J* = 6.1, 1H, H-C_{iPr}O), 4.83 – 4.78 (m, 1H, $\underline{H}$-C(NH)$(C)_2$), 2.93 (t, *J* = 2.6, 1H, HCC), 2.85 – 2.74 (m, 2H, $\underline{H}_2$C(CCH)), 1.42 (d, *J* = 6.1, 6H, $(H_3C)_2C$).

^{13}C NMR (176 MHz, DMSO): δ = 169.7 (C=O), 167.4 (C=O), 165.9 (C=O), 164.9 (C=O), 164.5 (C=O), 162.3 ($C_{Ar,D}$-$\underline{C}$=O), 152.9 ($C_{Ar,D}$-OiPr), 142.3 (C_{Ar}), 141.7 (C_{Ar}), 141.4 (C_{Ar}), 141.2 ($\underline{C}_{Ar,D}$-CO), 138.7 (C_{Ar}), 132.5 (H-C_{Ar}), 130.1 (H-C_{Ar}), 130.0 (H-$C_{Py,4}$), 128.9 (C_{Ar}), 128.6 (H-C_{Ar}), 128.4 (H-C_{Ar}), 128.6 (H-C_{Ar}), 128.4 (C_{Ar}), 126.0 ($C_{Ar,D}$-NH), 119.5 (H-C_{Ar}), 119.5 (H-C_{Ar}), 118.9 (H-C_{Ar}), 118.3 (CN), 116.1 (H-$C_{Py,3}$), 114.0 (C_{Ar}), 80.7 ($\underline{C}$CH), 73.2 (C$\underline{C}$H), 69.6 (C_{iPr}-H), 53.6 ($\underline{C}$H-$(C)_2$(N)), 21.8 ($(\underline{C}H_3)_2C$), 21.4 ($\underline{C}H_2$-CCH).

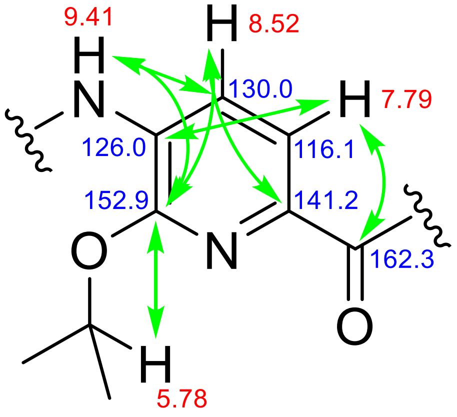

HRMS (ESI): calculated for $[M+H]^+$: 778.2620, found: 778.2619.

Synthesis of methoxy (ring D) cystobactamid ***(10)***

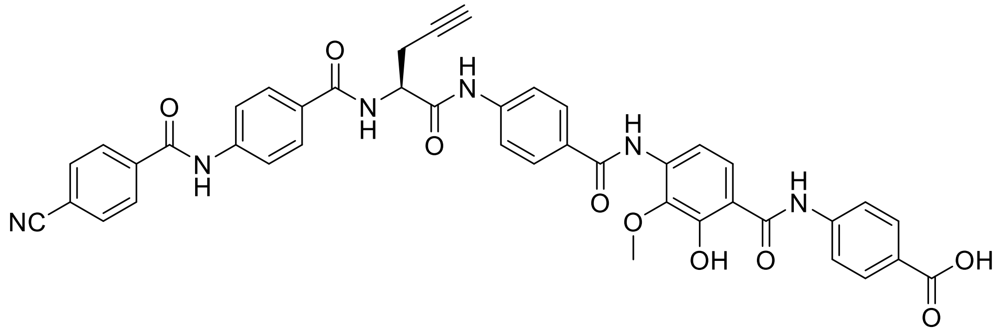

The final product was obtained according to the general *t*Bu-deprotection procedure using the cystobactamid-precursor **S52** (84.4 mg, 103 µmol, 1.00 Eq), DCM (1.5 mL) and TFA (0.40 mL, 50 Eq). The reaction was conducted at RT for 1 h 40 min and HPLC method 1 was used, including lyophillization over 3 d. Yield: 37.9 mg, 48%.

logD$_{7.4}$ = 1.1 ± 0.2

^{1}H NMR (500 MHz, DMSO): *δ* = 12.59 (br s, 2H), 10.73 (s, 1H, H-NCO), 10.58 (s, 1H, H-NCO), 9.28 (br s, 1H, H-NCO), 8.79 (d, *J* = 7.6, 1H, H-NCO), 8.13 (d, *J* = 8.9, 2H, H-C$_{Ar}$), 8.05 (d, *J* = 8.7, 2H, H-C$_{Ar}$), 7.98 – 7.95 (m, 2H, H-C$_{Ar}$), 7.95 – 7.89 (m, 6H, H-C$_{Ar}$), 7.84 – 7.77 (m, 4H, H-C$_{Ar}$), 7.65 (d, *J* = 9.0, 1H, H-C$_{Ar,D}$), 7.40 – 7.25 (m, 1H, H-C$_{Ar,D}$), 4.81 (td, *J* = 8.2, 6.3, 1H, $\underline{H}$-C(NH)(C)$_2$), 3.85 (s, 3H, H$_3$CO), 2.94 (t, *J* = 2.7, 1H, HCC), 2.85 – 2.71 (m, 2H, $\underline{H}_2$C(CCH)).

^{13}C NMR (126 MHz, DMSO): *δ* = 169.7 (C=O), 167.6 (C$_{Ar,D}$-C=O), 167.1 (C=O), 166.0 (C=O), 164.5 (C=O), 164.3 (C=O), 143.7 (C$_{Ar}$), 141.9 (C$_{Ar}$), 141.7 (C$_{Ar}$), 140.0 (C$_{Ar,D}$-OMe), 138.7 (C$_{Ar}$), 134.9 (C$_{Ar}$), 132.6 (C$_{Ar}$-H), 130.4 (C$_{Ar}$-H), 129.2 (C$_{Ar}$), 129.0 (C$_{Ar}$), 128.7 (C$_{Ar}$-H), 128.5 (C$_{Ar}$-H), 128.5 (C$_{Ar}$-H), 124.8 (C$_{Ar}$), 123.5 (C$_{Ar,D}$-H), 119.5 (C$_{Ar}$-H), 119.5 (C$_{Ar}$-H), 118.8 (C$_{Ar}$-H), 118.7 (C$_{Ar,D}$), 118.3 (CN), 114.5 (C$_{Ar}$), 114.1 (C$_{Ar}$), 80.7 ($\underline{C}$CH), 73.2 (C$\underline{C}$H), 59.4 (OCH$_3$), 53.5 ($\underline{C}$H-(C)$_2$(N)), 21.5 ($\underline{C}$H$_2$-CCH).

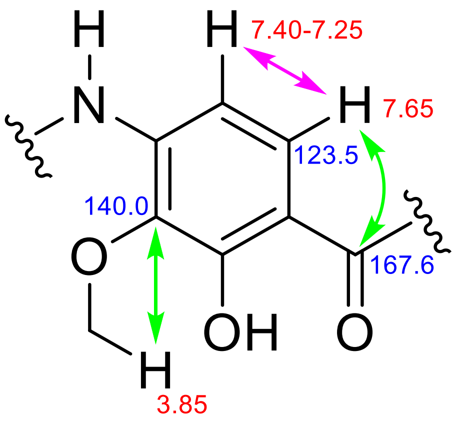

HRMS (ESI): calculated for $[M+H]^+$: 765.2304, found: 765.2303.

*Synthesis of difluoromethyl (ring D) cystobactamid (**9**)*

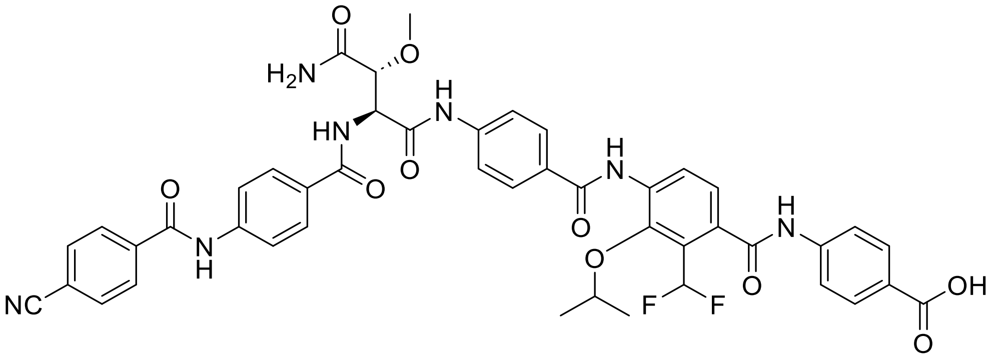

The cystobactamid precursor **S46** (14.4 mg, 0.02 mmol, 1.0 Eq) was dissolved in precooled TFA (1 mL) at 0°C with stirring. The solution was warmed up to RT over 30 min. Et_2O was added at 0°C. The precipitate was filtered off, washed with an excess of Et_2O and dried *in vacuo* to furnish the title compound (9.9 mg, 73%) as beige amorphous solid.

[*α*]$_D^{23}$ = -0.5° (c 0.1, MeOH)

^{1}H NMR (600 MHz, DMSO-d$_6$): *δ* = 12.74 (bs, 1H, CO_2H), 10.76 (s, 1H, NH), 10.72 (s, 1H, NH), 10.55 (s, 1H, NH), 9.97 (s, 1H, NH), 8.46-8.44 (d, *J* = 8.2 Hz, 2H, CHN<u>H</u>), 8.13-8.12 (d, *J* = 8.6 Hz, 2H, H_{Ar}), 8.05-8.03 (m, 4H, H_{Ar}), 7.94-7.92 (d, *J* = 8.3 Hz, 2H, H_{Ar}), 7.91-7.89 (m, 4H, H_{Ar}), 7.88-7.86 (d, *J* = 8.3 Hz, 1H, H_{Ar}), 7.83-7.81 (m, 4H, H_{Ar}), 7.54-7.48 (d, *J* = 39.7 Hz, 2H, NH_2), 7.39-7.38 (d, *J* = 8.2 Hz, 1H, H_{Ar}), 7.28-7.10 (t, *J* = 53.7 Hz, 1H, CHF_2), 4.93-4.90 (t, *J* = 8.1 Hz, 1H, C<u>H</u>NH), 4.40-4.34 (sept, *J* = 6.2 Hz, 1H, C<u>H</u>(CH_3)$_2$), 4.10-4.08 (d, *J* = 8.0 Hz, 1H, C<u>H</u>OCH_3), 3.31 (s, 3H, OCH_3), 1.23-1.22 (d, *J* = 6.2 Hz, 6H, CH(C<u>H</u>$_3$)$_2$)

^{13}C NMR (151 MHz, DMSO-d$_6$): *δ* = 170.9 (CO), 168.7 (CO), 166.9 (CO), 166.2 (CO), 165.5 (CO), 164.6 (CO), 164.5 (CO), 150.0 (C_{Ar}), 143.1 (C_{Ar}), 142.1 (C_{Ar}), 141.8 (C_{Ar}),

138.7 (C_{Ar}), 134.2 (C_{Ar}), 133.2 (C_{Ar}), 132.5 (C_{Ar}), 130.3 (C_{Ar}), 129.5 (C_{Ar}), 128.9 (C_{Ar}), 128.6 (C_{Ar}), 128.6 (C_{Ar}), 128.3 (C_{Ar}), 128.2 (C_{Ar}), 125.5, (C_{Ar}), 124.9 (C_{Ar}), 123.3 (C_{Ar}), 119.6 (C_{Ar}), 118.9 (C_{Ar}), 118.8 (C_{Ar}), 118.3 (CN), 114.1 (C_{Ar}), 113.5-110.4 (t, *J* = 236.5 Hz, CHF_2), 80.0 ($\underline{C}HOCH_3$), 77.0 ($\underline{C}H(CH_3)_2$), 57.6 (OCH_3), 55.8 (CHNH), 21.9 ($CH(\underline{C}H_3)_2$)

^{19}F NMR (376 MHz, DMSO-d_6): δ = -110.11- -110.25 (d, *J* = 53.7 Hz, 2F, CHF_2)

HRMS (ESI): calculated for $[M-H]^-$: 874.2648; found: 874.2642.

Synthesis of isobutyl (ring D) cystobactamid ***(11)***

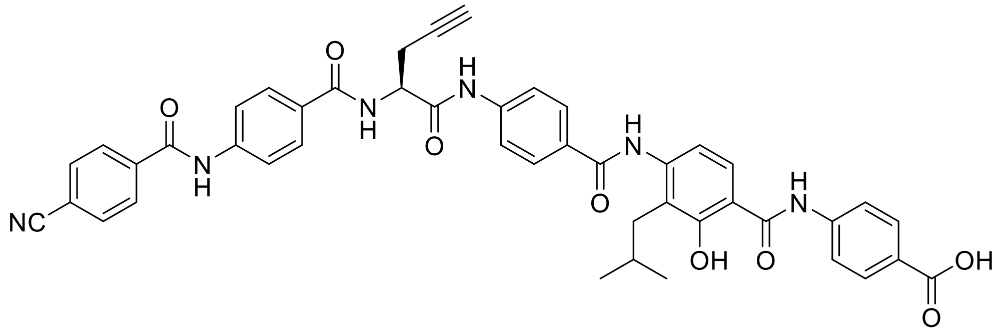

a) The protected cystobactamid precursor was obtained according to general procedure B, involving reaction of the CDE-fragment **S41** (30.7 mg, 58.2 µmol, 1.00 Eq), the standard AB-central AA-fragment **88** (23.1 mg, 1.10 Eq), dry pyridine (23 µL, 5.0 Eq) and T3P-solution (69 µL, 2.0 Eq) in dry EA (1.0 mL) for 1 h. Precipitation of the product was observed during the workup, thus drying over Na_2SO_4 was omitted.

b) Modified from[18]: The crude product and $Pd(PPh_3)_4$ (3.4 mg, 0.05 Eq) were solved in dry THF (1.0 mL) under Argon atmosphere before adding aniline (32 µL, 6.0 Eq). The reaction was stirred at RT while being monitored with LCMS. Solvents were removed from the reaction mixture after 1.5 h and the crude material was purified by preparative HPLC using HPLC method 1. The combined product fractions were lyophillized to give a nearly colorless solid: 24.9 mg, 54% o2s, (purity: 95 mol%).

$logD_{7.4}$ = 2.4 ± 0.2

^{1}H NMR (700 MHz, DMSO): δ = 12.74 (br s, 2H), 10.73 (s, 1H, H-NCO), 10.56 (s, 1H, H-NCO), 9.71 (br s, 1H), 8.80 (d, *J* = 7.5, 1H, H-NCO), 8.13 (d, *J* = 8.6, 2H, H-C_{Ar}), 8.04 (d, *J* = 8.6, 2H, H-C_{Ar}), 7.99 – 7.95 (m, 4H, H-C_{Ar}), 7.94 (d, *J* = 8.6, 2H, H-C_{Ar}), 7.90 (d, *J* = 8.8, 2H, H-C_{Ar}), 7.89 – 7.86 (m, 1H, H-$C_{Ar,D}$), 7.83 (d, *J* = 8.7, 2H, H-C_{Ar}),

7.79 (d, J = 8.9, 2H, H-C$_{Ar}$), 6.87 (br s, 1H, H-C$_{Ar,D}$), 4.81 (td, J = 8.1, 6.4, 1H, H-C(NH)(C)$_2$), 2.93 (t, J = 2.6, 1H, HCC), 2.86 – 2.72 (m, 2H, H$_2$C(CCH)), 2.58 (d, J = 7.1, 2H, H$_2$C-C$_{Ar,D}$), 1.89 – 1.81 (m, 1H, H-C(CH$_3$)$_2$), 0.81 (d, J = 6.7, 6H, (H$_3$C)$_2$CH).

^{13}C NMR (176 MHz, DMSO): δ = 169.6 (C=O), 168.9 (C=O), 167.1 (C=O), 165.9 (C=O), 164.6 (C=O), 164.5 (C=O), 141.7 (C$_{Ar}$), 141.6 (C$_{Ar}$), 141.3 (C$_{Ar}$), 138.7 (C$_{Ar}$), 132.5 (C$_{Ar}$-H), 130.2 (C$_{Ar}$-H), 129.3 (C$_{Ar}$), 129.0 (C$_{Ar}$), 128.6 (C$_{Ar}$-H), 128.5 (C$_{Ar}$-H), 128.4 (C$_{Ar}$-H), 126.3 (C$_{Ar}$), 125.9 (C$_{Ar,D}$-iBu), 125.5 (C$_{Ar}$-H), 120.2 (C$_{Ar}$), 119.5 (C$_{Ar}$-H), 119.4 (C$_{Ar}$), 118.7 (C$_{Ar}$-H), 118.6 (C$_{Ar}$), 118.3 (CN), 114.0 (C$_{Ar}$), 113.8 (C$_{Ar}$), 80.7 (CCH), 73.2 (CCH), 53.4 (CH-(C)$_2$(N)), 33.4 (CH$_2$-C$_{Ar,D}$), 27.7 (C(CH$_3$)$_2$), 22.7 ((CH$_3$)$_2$C), 21.4 (CH$_2$-CCH).

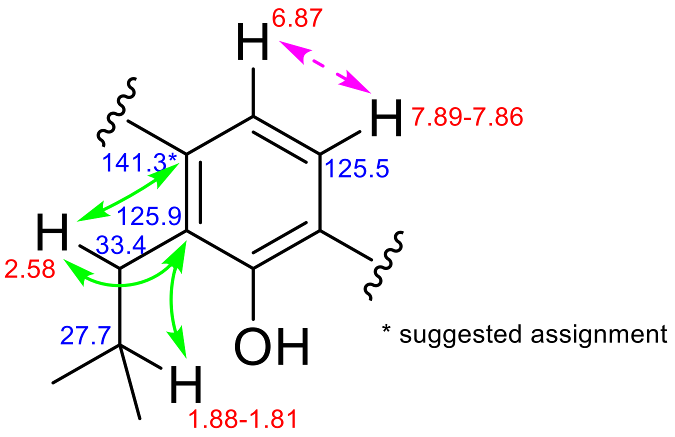

HRMS (ESI): calculated for [M+H]$^+$: 791.2824, found: 791.2824.

*Synthesis of benzylamine (CD-amide) cystobactamid **(40)***

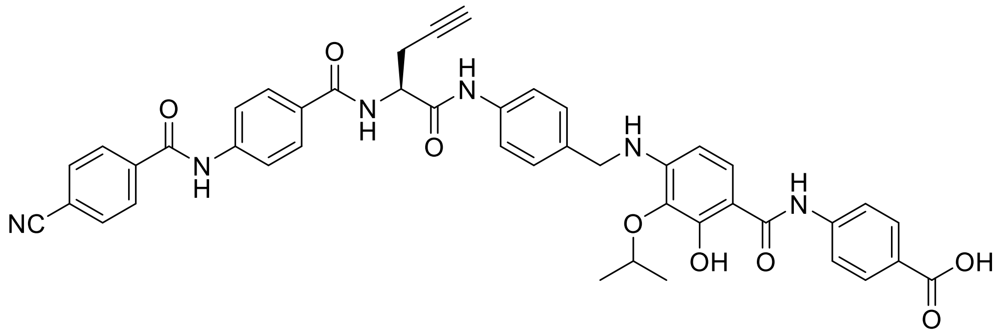

a) The protected cystobactamid precursor was obtained according to general procedure B, involving reaction of the CDE-fragment **51** (40 mg, 63 µmol, 1.0 Eq), the standard AB-central AA-fragment **88** (24.0 mg, 1.05 Eq), dry pyridine (26µL, 5.0 Eq) and T3P-solution (75 µL, 2.0 Eq) in dry EA (1.0 mL) for 1 h 45 min.

b) Modified from[18]: The crude material and aniline (17 µL, 3.0 Eq) were solved in dry THF (0.63 mL). $Pd(PPh_3)_4$ (7.3 mg, 0.10 Eq) was added under Argon atmosphere and the solution was stirred while being screened with LCMS and TLC. After 2.5 h the temperature was increased to 40°C. Aniline (17 µL, 3.0 Eq) was added after 30 min and the reaction was stirred overnight at 40°C. Subsequently, solvents were removed from the reaction solution and the material was purified by FCC (solid loading, PE/acetone, 80/20->70/30). A brownish gum was obtained (15 mg) that was directly used in the next step. Note: An unsuccessful attempt was made to remove impurities by extraction with DCM/HCl (0.1 M) and sat. $NaHCO_3$ sol.

c) The final product was obtained according to the general *t*Bu-deprotection procedure using the crude precursor material (15 mg, 16 µmol, 1.0 Eq), DCM (0.18 mL) and TFA (45 µL, 50 Eq). The reaction was conducted at RT for 1 h 45 min and HPLC method 1 was used, including lyophillization over 5 d. Yield: 4.7 mg, 9.5% o3s.

^{1}H NMR (700 MHz, DMSO): *δ* = 12.70 (br s, 1H, HOOC), 12.42 (s, 1H, HO), 10.69 (s, 1H, H-NCO), 10.20 (s, 1H, H-NCO), 10.14 (s, 1H, H-NCO), 8.68 (d, *J* = 7.6, 1H, H-NCO), 8.12 (d, *J* = 8.1, 2H, H-C_{Ar}), 8.04 (d, *J* = 7.7, 2H, H-C_{Ar}), 7.94 (d, *J* = 8.5, 2H, H-C_{Ar}), 7.90 (d, *J* = 8.5, 2H, H-C_{Ar}), 7.88 (d, *J* = 8.6, 2H, H-C_{Ar}), 7.77 (d, *J* = 8.7, 2H, H-C_{Ar}), 7.55 (m, 3H, H-C_{Ar}, H-$C_{Ar,D}$), 7.27 (d, *J* = 8.7, 2H, H-C_{Ar}), 6.42 (s, 1H, <u>H</u>N-CH_2), 6.10 (d, *J* = 8.9, 1H, H-$C_{Ar,D}$), 4.77 – 4.72 (m, 1H, <u>H</u>-C(NH)$(C)_2$), 4.57 (hept, *J* = 6.1, 1H, H-C_{iPr}O), 4.39 (d, *J* = 6.2, 2H, <u>H</u>$_2$C-NH), 2.90 (dt, *J* = 2.7, 1.3, 1H, HCC), 2.73 (qdd, *J* = 16.7, 7.4, 2.7, 2H, <u>H</u>$_2$C(CCH)), 1.26 (d, *J* = 6.2, 6H, $(H_3C)_2C$).

^{13}C NMR (176 MHz, DMSO): *δ* = 169.4 (C=O), 169.0 (C=O), 167.0 (C=O), 165.8 (C=O), 164.4 (C=O), 154.4 (C_{Ar}-OH), 147.3 (C_{Ar}), 142.5 (C_{Ar}), 141.6 (C_{Ar}), 138.7 (C_{Ar}), 137.4 (C_{Ar}), 135.0 (C_{Ar}), 132.5 (C_{Ar}-H), 130.1 (C_{Ar}-H), 129.8 ($C_{Ar,D}$-OiPr), 129.0 (C_{Ar}), 128.6 (C_{Ar}-H), 128.4 (C_{Ar}-H), 127.2 (C_{Ar}-H), 125.6 (C_{Ar}), 123.9 ($C_{Ar,D}$-H), 120.2 (C_{Ar}-H), 119.5 (C_{Ar}-H), 119.4 (C_{Ar}-H), 118.3 (CN), 114.0 (C_{Ar}), 103.8 (C_{Ar}), 101.6 ($C_{Ar,D}$-H), 80.7 (<u>C</u>CH), 73.1 (C<u>C</u>H), 72.9 (C_{iPr}-H), 53.3 (<u>C</u>H-$(C)_2$(N)), 45.1 (CH_2-NH), 22.2 ((<u>C</u>H$_3$)$_2$C), 21.5 (<u>C</u>H$_2$-CCH).

HRMS (ESI): calculated for $[M+H]^+$: 779.2824, found: 779.2829.

Synthesis of terephthalic acid (ring D) cystobactamid ***(42)***

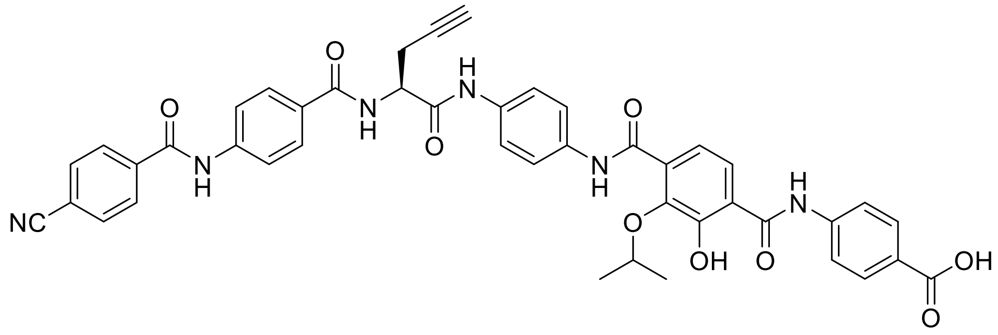

a) The protected cystobactamid precursor was obtained according to general procedure B, involving reaction of the CDE-fragment **59** (68.8 mg, 126 µmol, 1.00 Eq), the standard AB-central AA-fragment **88** (52.4 mg, 1.15 Eq), dry pyridine (51 µL, 5.0 Eq) and T3P-solution (0.15 mL, 2.0 Eq) in dry EA (2.0 mL) for 2 h.

b) Modified from[18]: The crude product, fresh $Pd(PPh_3)_4$ (7.3 mg, 0.05 Eq) and aniline (35 µL, 3.0 Eq) were solved in dry THF (1.9 mL) under Argon atmosphere and the reaction was stirred at RT while being monitored with LCMS. After 1.5 h the solvent was removed u.r.p. and the product was isolated by FCC (solid loading, 100x theoretical product mass, PE/EA/AcOH, 50/50/2->40/60/2->60/40/10) to give a brownish solid that was used directly in the next step.

c) The final product was obtained according to the general *t*Bu-deprotection procedure using DCM (1.5 mL) and TFA (0.39 mL, 40 Eq). The reaction was conducted at RT for 1.5 h and HPLC method 1 was used. Yield: 45.7 mg, 46% o3s.

logD$_{7.4}$ = 1.5 ± 0.5

^{1}H NMR (500 MHz, DMSO): δ = 12.87 (br s, 1H, HOOC), 12.01 (br s, 1H, HO-C$_{Ar,D}$), 10.89 (br s, 1H, H-NCO), 10.72 (s, 1H, H-NCO), 10.30 (s, 1H, H-NCO), 10.26 (s, 1H, H-NCO), 8.73 (d, J = 7.8, 1H, H-NCO), 8.13 (d, J = 8.7, 2H, H-C$_{Ar}$), 8.05 (d, J = 8.7, 2H, H-C$_{Ar}$), 8.01 – 7.94 (m, 4H, H-C$_{Ar}$), 7.92 – 7.85 (m, 4H, H-C$_{Ar}$), 7.84 (d, J = 8.5, 1H, H-C$_{Ar,D}$), 7.68 (d, J = 9.2, 2H, H-C$_{Ar}$), 7.62 (d, J = 9.2, 2H, H-C$_{Ar}$), 7.13 (d, J = 8.2, 1H, H-C$_{Ar,D}$), 4.81 – 4.75 (m, 1H, <u>H</u>-C(NH)(C)$_2$), 4.53 – 4.44 (m, 1H, H-C$_{iPr}$O), 2.93 (t, J = 2.6, 1H, HCC), 2.83 – 2.68 (m, 2H, <u>H</u>$_2$C(CCH)), 1.21 (d, J = 6.1, 6H, (H$_3$C)$_2$C).

^{13}C NMR (126 MHz, DMSO): δ = 168.9 (C=O), 167.8 (HO-C$_{Ar,D}$-C$_{Ar,D}$-<u>C</u>=O), 166.9 (C=O), 165.9 (C=O), 164.5 (C=O), 164.0 (iPrO-C$_{Ar,D}$-C$_{Ar,D}$-<u>C</u>=O), 154.2 (C$_{Ar,D}$-OH),

143.5 ($C_{Ar,D}$-OiPr), 142.0 (C_{Ar}), 141.7 (C_{Ar}), 138.7 (C_{Ar}), 135.1 ($C_{Ar,D}$), 134.7 (C_{Ar}), 134.6 (C_{Ar}), 132.6 (C_{Ar}-H), 130.3 (C_{Ar}-H), 129.1 (C_{Ar}), 128.7 (C_{Ar}-H), 128.5 (C_{Ar}-H), 126.3 (C_{Ar}), 122.9 ($C_{Ar,D}$-H), 120.6 (C_{Ar}-H), 119.9 (C_{Ar}-H), 119.9 (C_{Ar}-H), 119.5 (C_{Ar}-H), 118.6 ($C_{Ar,D}$), 118.4 (CN), 117.8 ($C_{Ar,D}$-H), 114.1 (C_{Ar}), 80.9 ($\underline{C}$CH), 75.9 (C_{iPr}-H), 73.1 (C$\underline{C}$H), 53.3 ($\underline{C}$H-(C)$_2$(N)), 22.2 (($\underline{C}H_3$)$_2$C), 21.6 ($\underline{C}H_2$-CCH).

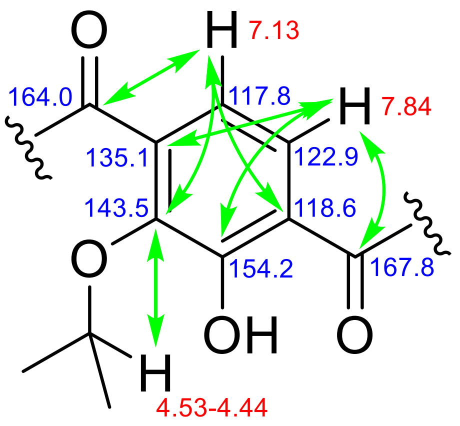

HRMS (ESI): calculated for $[M+H]^+$: 793.2617, found: 793.2616.

*Synthesis of benzylamine (DE-amide) cystobactamid (**41**)*

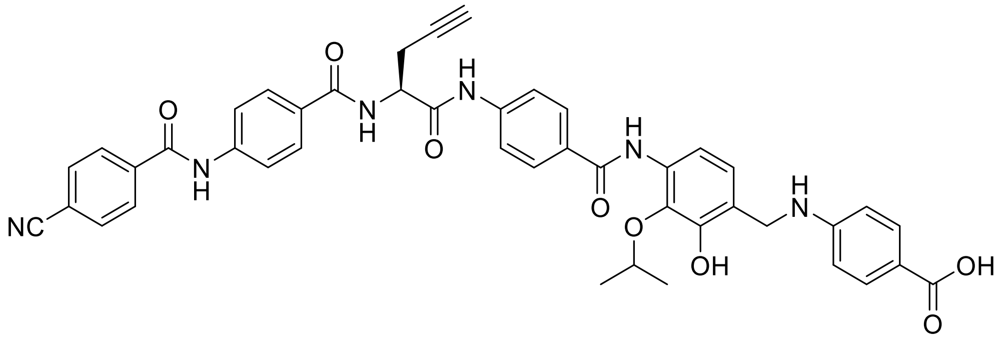

tert-butyl (S)-4-((2-(allyloxy)-4-(4-(2-aminopent-4-ynamido)benzamido)-3-isopropoxybenzyl)(tert-butoxycarbonyl)amino)benzoate **S51** (32 mg, 44 µmol, 1.0 Eq), HATU (18.4 mg, 1.1 Eq) and 4-(4-cyanobenzamido)benzoic acid **86** (13 mg, 49 µmol, 1.1 Eq) were added to a dry flask and further dried under high vacuum. Dry DMF (0.3 mL) and DIPEA (23 µl, 132 µmol, 3.0 eq) were added under nitrogen atmosphere at 0°C. The solution was stirred at 0°C and controlled over LCMS. After full conversion to the amide, aniline (12 µL, 3.0 Eq), tetrakis(triphenylphosphine)palladium(0) (1.0 mg, 0.02 Eq) and dry THF (0.8 mL) were added under nitrogen atmosphere. The reaction was stirred at 0°C and controlled over LCMS. After completion, the solvent was concentrated under reduced pressure and coevaporated with n-heptane. The residue

was directly purified by flash chromatography with PE/EtOAc + 2 % AcOH. The crude residue was dried under high vacuum. Dry DCM (0.35 mL) was added under argon atmosphere and the solution was cooled down to 0°C. Trifluoracetic acid (0.21 mL, 62 Eq) was added under argon atmosphere. The solution was stirred for 3 hat 0°C and controlled over LCMS. After completion, the solvent was removed under reduced pressure. The residue was coevaporated with DCM twice. The crude product was purified by reversed-phase HPLC. A colorless solid was obtained, yield: 24.1 mg, 70 % o3s).

^{1}H NMR (700 MHz, DMSO): δ = 11.97 (br s, 1H), 10.70 (s, 1H), 10.52 (s, 1H), 9.43 (s, 1H), 8.80 (s, 1H), 8.75 (d, 1H, J = 7.5 Hz), 8.13 (d, 2H, J = 8.5 Hz), 8.05 (d, 2H, J = 8.5 Hz), 7.97 – 7.93 (m, 4H), 7.90 (d, 2H, J = 8.8 Hz), 7.77 (d, 2H, J = 8.8 Hz), 7.65 (d, 2H, J = 8.8 Hz), 7.12 (d, 1H, J = 8.3 Hz), 6.91 (d, 1H, J = 8.4 Hz), 6.86 (t, 1H, J = 5.9 Hz), 6.60 (d, 2H, J = 8.8 Hz), 4.79 (dd, 1H, J = 7.7 Hz, 14.6 Hz), 4.28 (d, 2H, J = 5.8 Hz), 4.24 (quint., 1H, J = 6.2 Hz), 2.93 (t, 1H, J = 2.6 Hz), 2.78 (dddd, 2H, J = 2.6 Hz, 7.4 Hz, 11.1 Hz, 16.8 Hz), 1.21 (d, 6H, J = 6.2 Hz)

^{13}C NMR (176 MHz, DMSO): δ = 169.6, 167.5, 165.9, 164.5, 164.2, 152.6, 148.1, 141.7, 141.7, 138.8, 138.7, 132.5, 131.1, 130.8, 129.1, 129.0, 128.6, 128.4, 128.3, 123.6, 122.2, 119.5, 118.8, 118.3, 117.0, 116.0, 114.0, 111.0, 80.7, 75.1, 73.2, 53.4, 40.8, 22.1, 21.4.

HRMS (ESI): calculated from $[M+H]^+$: 779.2824, found: 779.2824.

Synthesis of triazole (DE-amide) cystobactamid ***(43)***

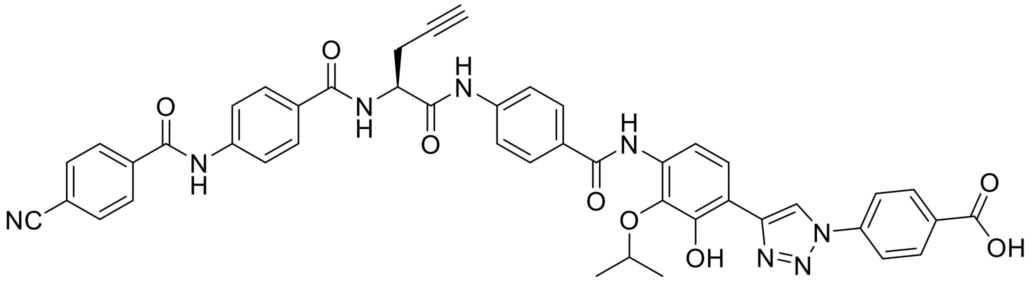

Note: The following reaction was divided in two equal-sized batches and is treated here as one batch:

Modified from[18]: The cystobactamid-precursor **S53** (36 mg, 40 µmol, 1.0 Eq) and aniline (24 µL, 6.5 Eq) were solved in dry THF (1.1 mL). Fresh $Pd(PPh_3)_4$ (5.1 mg,

0.11 Eq) was added under Argon atmosphere and the reaction solution was stirred for 1.5-2 h. After reaction control by LCMS the reaction mixture was concentrated u.r.p. and the residue was purified by preparative HPLC (method 1). After removing solvents u.r.p. at the rotary evaporator and by lyophillization the product was obtained as an almost colorless solid. Yield: 19.6 mg, 60%.

^{1}H NMR (500 MHz, DMSO): *δ* = 10.72 (s, 1H), 10.59 (s, 1H), 9.52 (s, 1H), 9.13 (s, 1H, H-CNN), 8.81 (d, *J* = 7.6, 1H, HNCO), 8.16 (d, *J* = 8.9, 2H), 8.13 (d, *J* = 8.7, 2H), 8.10 (d, *J* = 8.9, 2H), 8.05 (d, *J* = 8.9, 2H), 8.01 – 7.95 (m, 4H), 7.90 (d, *J* = 9.0, 2H), 7.81 (d, *J* = 9.0, 2H), 7.77 (d, *J* = 8.5, 1H, H-$C_{Ar,D}$), 7.46 (d, *J* = 8.5, 1H, H-$C_{Ar,D}$), 4.80 (q, *J* = 7.9, 1H, <u>H</u>-C(NH)$(C)_2$), 4.39 (hept, *J* = 6.0, 1H, H-C_{iPr}O), 2.94 (t, *J* = 2.6, 1H, H-CC), 2.86 – 2.72 (m, 2H, <u>H</u>$_2$C(CCH)), 1.26 (d, *J* = 6.3, 6H, $(H_3C)_2C$).

^{13}C NMR (126 MHz, DMSO): *δ* = 169.7 (C=O), 166.7 (C=O), 166.0 (C=O), 164.5 (C=O), 164.2 (C=O), 147.9 (C_{Ar}), 144.8 (C_{Ar}), 141.9 (C_{Ar}), 141.7 (C_{Ar}), 139.0 (C_{Ar}), 138.7 (C_{Ar}), 138.6 (C_{Ar}), 132.5 (C_{Ar}-H), 132.4 (C_{Ar}), 131.0 (C_{Ar}-H), 129.0 (C_{Ar}), 128.9 (C_{Ar}), 128.6 (C_{Ar}-H), 128.4 (C_{Ar}-H), 128.4 (C_{Ar}-H), 121.1 ($C_{Ar,D}$-H), 120.6 ($C_{triazol}$-H), 119.9 (C_{Ar}-H), 119.5 (C_{Ar}-H), 118.8 (C_{Ar}-H), 118.3 (CN), 115.9 ($C_{Ar,D}$-H), 114.2 (C_{Ar}), 114.0 (C_{Ar}), 80.7 (<u>C</u>CH), 75.1 (C_{iPr}-H), 73.2 (C<u>C</u>H), 53.5 (<u>C</u>H-$(C)_2$(N)), 22.1 ((<u>C</u>H$_3$)$_2$C), 21.4 (<u>C</u>H$_2$-CCH).

HRMS (ESI): calculated for $[M+H]^+$: 817.2729, found: 817.2737.

*Synthesis of biphenyl (DE-amide) cystobactamid (**44**)*

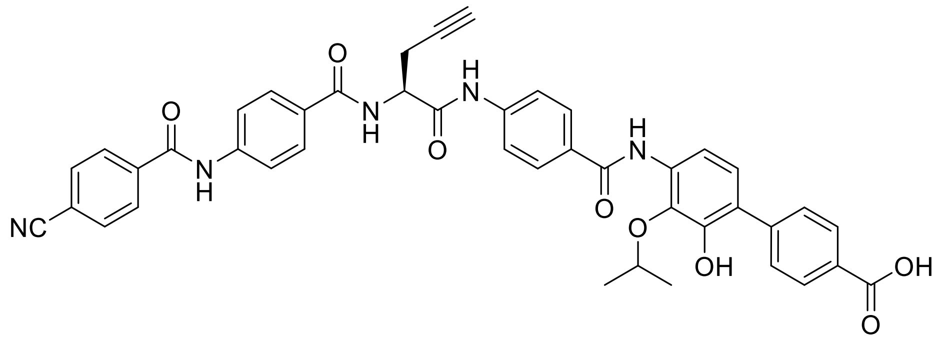

tert-butyl (S)-4'-(4-(2-aminopent-4-ynamido)benzamido)-3'-isopropoxy-2'-(methoxymethoxy)-[1,1'-biphenyl]-4-carboxylate **S54** (37.8 mg, 0.06 mmol, 1 Eq), HATU (29 mg, 1.2 Eq) and 4-(4-cyanobenzamido)benzoic acid **86** (20.0 mg, 0.08 mmol, 1.2 eq) were added to a dry flask and further dried at the vacuum pump. Dry DMF (0.3 mL) and DIPEA (34 µL, 3.1 Eq) were added under nitrogen atmosphere at

0°C. The solution was stirred at 0°C and controlled over LCMS. After the reaction was complete EtOAc (10 mL) was added. The organic phase was washed 3 times with a mixture of HCl (1 mL, 0.1 M) and brine (3 mL). The organic solvent was concentrated under reduced pressure and the crude product was dried under high vacuum. Dry DCM (0.55 mL) was added under argon atmosphere and the solution was cooled down to 0°C. Trifluoracetic acid (0.25 mL, 52 Eq) was added under argon atmosphere. The solution was stirred for 3 h at 0°C and controlled over LCMS. After the reaction was completed, the solvent was concentrated under reduced pressure. The residue was coevaporated with DCM. The crude product was purified by reversed-phase HPLC. A colorless solid was obtained, yield: 16.4 mg, 37% o2s).

^{1}H NMR (700 MHz, DMSO): *δ* = 12.85 (br s, 1H), 10.71 (s, 1H), 10.55 (s, 1H), 9.55 (s, 1H), 8.86 (s, 1H), 8.77 (d, 1H, J = 7.5 Hz), 8.13 (d, 2H, J = 8.4 Hz), 8.05 (d, 2H, J = 8.2 Hz), 8.00 – 7.95 (m, 6H), 7.91 (d, 2H, J = 8.8 Hz), 7.80 (d, 2H, J = 8.7 Hz), 7.71 (d, 1H, J = 8.3 Hz), 7.34 (d, 1H, J = 8.4 Hz), 7.11 (d, 1H, J = 8.4 Hz), 4.81 (dd, 1H, J = 7.7 Hz, 14.7 Hz), 4.29 (hept., 1H, J = 6.1 Hz), 2.94 (t, 1H, J = 2.4 Hz), 2.79 (dddd, 2H, J = 2.5 Hz, 7.4 Hz, 11.0 Hz, 16.8 Hz), 1.24 (d, 6H, J = 6.2 Hz)

^{13}C NMR (176 MHz, DMSO): *δ* = 169.7, 167.3, 166.0, 164.5, 164.3, 147.9, 142.7, 141.9, 141.7, 139.1, 138.7, 132.5, 132.1, 129.1, 129.1, 129.0, 128.8, 128.6, 128.5, 128.4, 125.0, 124.4, 119.5, 118.8, 118.3, 116.3, 114.1, 80.7, 75.3, 73.2, 53.5, 22.0, 21.4.

HRMS (ESI): calculated for $[M+H]^+$: 750.2564, found: 750.2557.

*Synthesis of N-Me (DE-amide) cystobactamid (**46**)*

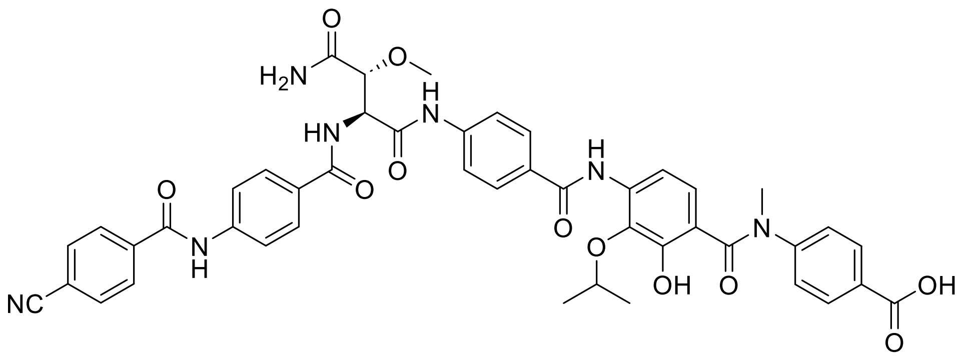

The *tert*-Butyl ester precursor **S50** (16.0 mg, 0.02 mmol) was dissolved in precooled TFA (1 mL) at 0°C with stirring. The solution was warmed up to RT over 30 min. Et_2O was added at 0°C. The precipitate was filtered off, washed with an excess of Et_2O and

dried *in vacuo* to furnish the title compound (7.4 mg, 49%) as colorless amorphous solid.

[*α*]$_D$^{21} = +2.1 ° (c 0.1, MeOH)

^{1}H NMR (600 MHz, DMSO-d_6): δ = 12.89 (s, 1H, CO_2H), 10.71 (s, 1H, OH), 10.51 (s, 1H, NH), 9.31 (s, 1H, NH), 9.28 (s, 1H, NH), 8.44-8.43 (d, *J* = 8.2 Hz, 1H, CHNH), 8.13-8.12 (d, *J* = 8.7 Hz, 2H, H_{Ar}), 8.05-8.04 (d, *J* = 8.6 Hz, 2H, H_{Ar}), 7.91-7.87 (m, 6H, H_{Ar}), 7.79-7.76 (m, 4H, H_{Ar}), 7.53-7.46 (d, *J* = 39.0 Hz, 2H, NH_2), 7.28-27 (d, *J* = 8.6 Hz, 2H, H_{Ar}), 7.23-7.22 (d, *J* = 8.4 Hz, 1H, H_{Ar}), 6.87-6.86 (d, *J* = 8.4 Hz, 1H, H_{Ar}), 4.91-4.88 (t, *J* = 8.1 Hz, 1H, CHNH), 4.08-4.04 (m, 2H, $CHOCH_3$, $CH(CH_3)_2$), 3.38 (s, 3H, NCH_3), 3.30 (s, 3H, OCH_3), 1.02-1.01 (d, *J* = 6.1 Hz, 6H, $CH(CH_3)_2$)

^{13}C NMR (151 MHz, DMSO-d_6): δ = 170.9 (CO), 168.6 (CO), 168.0 (CO), 166.7 (CO), 165.4 (CO), 164.5 (CO), 164.0 (CO), 147.8 (C_{Ar}), 147.3 (C_{Ar}), 141.9 (C_{Ar}), 141.8 (C_{Ar}), 138.7 (C_{Ar}), 137.5 (C_{Ar}), 133.4 (C_{Ar}), 132.5 (C_{Ar}), 129.5 (C_{Ar}), 128.9 (C_{Ar}), 128.8 (C_{Ar}), 128.6 (C_{Ar}), 128.3 (C_{Ar}), 128.1 (C_{Ar}), 126.3 (C_{Ar}), 122.9 (C_{Ar}), 121.7 (C_{Ar}), 119.6 (C_{Ar}), 118.8 (C_{Ar}) 118.3 (CN), 114.6 (C_{Ar}), 114.0 (C_{Ar}), 80.0 ($CHOCH_3$), 74.6 ($CH(CH_3)_2$), 57.7 (CHNH), 55.7 (OCH_3), 36.7 (NCH_3). 21.7 ($CH(CH_3)_2$)

HRMS (ESI): calculated for $[M-H]^-$: 854.2786; found: 854.2778.

*Synthesis of quinazoline (ring E) cystobactamid (**47**)*

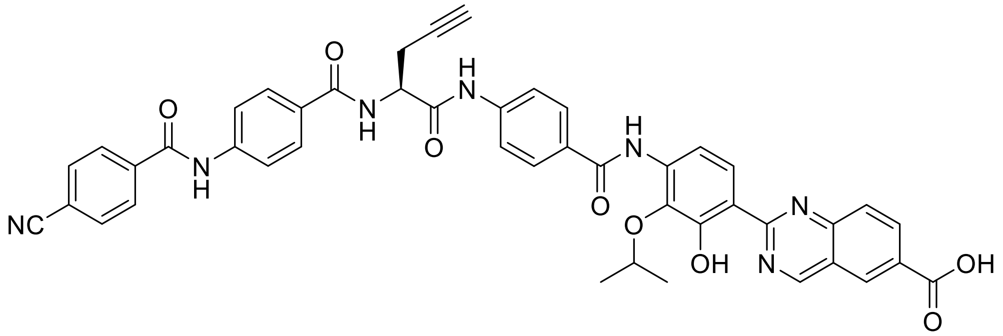

a) (S)-4-(2-(4-(4-cyanobenzamido)benzamido)pent-4-ynamido)benzoic acid (90 mg, 0.19 mmol, 2.7 Eq) and methyl 2-(2-(allyloxy)-4-amino-3-isopropoxyphenyl)quinazoline-6-carboxylate **78** (27.6 mg, 70.2 µmol, 1.0 Eq) were added to a dry vial and further dried under reduced pressure. Dry THF (0.7 mL) and dry DIPEA (98 µL, 8.0 Eq) were added under nitrogen atmosphere and the mixture was cooled down to 0°C. Phosphoryl chloride (17.0 µL, 0.18 mmol, 2.6 Eq) was dissolved in dry DCM (0.15 mL) and added very slowly under nitrogen atmosphere. The reaction was kept at 0°C and controlled over LCMS. After completion, saturated

NH_4Cl solution (1 mL) and brine (19 mL) were added. The aqueous phase was extracted with EtOAc (3x10 mL). The solvent was removed under reduced pressure.

b) Dry THF (2.2 mL) and aniline (35.0 µL, 3.0 Eq) were added under nitrogen atmosphere. Tetrakis(triphenylphosphine)palladium(0) (10 mg, 0.07 Eq) was added to the mixture and it was stirred for 3 h at room temperature. The reaction was controlled over LCMS. After completion, the crude product was purified by RP flash chromatography. The product was directly used in further reactions. A yellow solid (13.7 mg crude) was obtained.

c) The crude product (13.7 mg, 16.8 µmol, 1.0 Eq) was suspended in water (0.1 mL) and THF (0.05 mL). Lithium hydroxide (1.5 mg, 3.7 Eq) was added. The reaction was controlled via LCMS. After completion, the crude product was purified by RP HPLC. The product was a yellow solid, yield: 3.5 mg, 6 % o3s.

^{1}H NMR (700 MHz, DMSO): δ = 14.07 (s, 1H), 10.72 (br s, 1H), 10.61 (br s, 1H), 9.92 (br s, 1H), 9.36 (br s, 1H), 8.83 (br s, 1H), 8.80 (d, 1H, J = 7.6 Hz), 8.48 (dd, 1H, J = 1.5 Hz, 8.8 Hz), 8.37 (d, 1H, J = 9.0 Hz), 8.18 (d, 1H, J = 8.6 Hz), 8.13 (d, 2H, J = 8.5 Hz), 8.05 (d, 2H, J = 8.2 Hz), 7.98 – 7.96 (m, 4H), 7.90 (d, 2H, J = 8.8 Hz), 7.85 – 7.81 (m, 3H), 4.81 (dd, J = 7.7 Hz, 14.7 Hz), 4.72 – 4.66 (m, 1H), 2.93 (t, 1H, J = 2.6 Hz), 2.79 (dddd, 2H, J = 2.6 Hz, 7.4 Hz, 11.2 Hz, 16.8 Hz), 1.32 (d, 6H, J = 6.1 Hz)

^{13}C NMR (156 MHz, DMSO): δ = 169.7, 166.4, 166.0, 164.5, 164.1, 163.2, 162.0, 154.1, 149.0, 142.2, 141.7, 138.7, 136.6, 136.0, 135.1, 132.5, 130.6, 129.6, 128.7, 128.6, 128.4, 128.3, 126.6, 124.2, 122.2, 119.5, 119.0, 118.3, 115.6, 114.0, 112.1, 80.7, 74.6, 73.2, 53.3, 22.5, 21.4.

HRMS (ESI): calculated for $[M+H]^+$: 802.2625, found: 802.2619.

*Synthesis of benzimidazol (ring E) cystobactamid (**48**)*

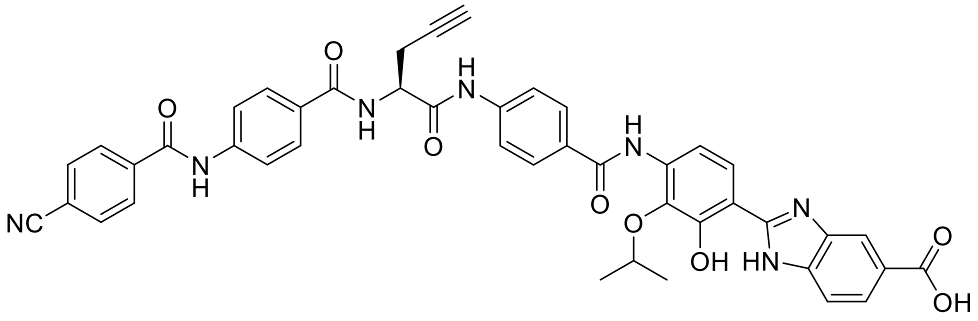

a) 1-allyl 5-methyl (S)-2-(4-(4-(2-((((9H-fluoren-9-yl)methoxy)carbonyl)amino)pent-4-ynamido)benzamido)-2-(allyloxy)-3-isopropoxyphenyl)-1H-benzo[d]imidazole-1,5-dicarboxylate **S55** (120 mg, 0.13 mmol, 1 Eq) was dissolved in MeCN (0.8 mL) and diethylamine (0.21 mL, 15 Eq) at 0°C and stirred for 1 h. The solvent was evaporated under reduced pressure. MeCN (1 mL) was added to the residue and the solvent was removed again. This was repeated twice. The flask was further dried under high vacuum. 4-(4-cyanobenzamido)benzoic acid **86** (39 mg, 0.15 mmol, 1.1 Eq) and HATU (56 mg, 1.1 Eq) were added to the crude residue under argon atmosphere. Dry DMF (1.0mL) was added under argon atmosphere and the solution was cooled down to 0°C. DIPEA (70 µL, 3.1 Eq) was added under argon atmosphere. The solution was stirred under argon atmosphere and controlled by LCMS. After the reaction was completed it was quenched with saturated $NaHCO_3$ (2 mL) and brine (16 mL). The inorganic layer was extracted with EtOAc (3x5 mL). The organic phases were combined and the solvent was concentrated under reduced pressure and coevaporated with n-heptane. The residue was dried under high vacuum. To the crude product tetrakis(triphenylphosphine)palladium(0) (7.7 mg, 0.05 Eq) and dry THF (2 mL) were added under argon atmosphere. N,N-dimethylbarbituric acid (104 mg, 5 Eq) was added to the mixture and it was stirred for 3 h at RT. The reaction was controlled by LCMS. After completion the crude mixture was directly purified by flash chromatography with PE/EtOAc + 2 % AcOH. A brown to red solid was obtained (55.5 mg, crude).

b) The crude material (55.5 mg, 1.0 Eq) was dissolved in water (0.6 mL) and THF (0.5 mL). Lithium hydroxide hydrate (23 mg, 7.9 Eq) was added to the mixture. The reaction was controlled via LCMS. After completion, the crude product was directly purified by HPLC. A yellow to orange solid was obtained, yield: 4.6 mg, 4 % o3s)

^{1}H NMR (700 MHz, DMSO): *δ* = 10.73 (br s, 1H), 10.63 (br s, 1H), 9.35 (s, 1H), 8.82 (d, 1H, *J* = 7.4 Hz), 8.20 (br s, 1H), 8.13 (d, 2H, *J* = 8.4 Hz), 8.05 (d, 2H, *J* = 8.4 Hz), 7.98 – 7.95 (m, 4H, *J* = 8.8 Hz, 3.5 Hz, 4H), 7.92 – 7.88 (m, 4H), 7.85 – 8.82 (m, 3H), 7.75 (d, 1H, *J* = 8.6 Hz), 7.66 (d, 1H, *J* = 8.1 Hz), 4.81 (dd, 1H, *J* = 14.7 Hz, 7.7 Hz), 4.66 (hept., 1H, *J* = 6.0 Hz), 2.94 (t, 1H, *J* = 2.6 Hz), 2.79 (dddd, 2H, *J* = 16.8 Hz, 11.1 Hz, 7.5 Hz, 2.7 Hz), 1.30 (d, *J* = 6.1 Hz, 6H).

^{13}C NMR (176 MHz, DMSO): δ = 169.7, 168.1, 166.0, 164.5, 164.1, 142.1, 141.7, 138.7, 136.6, 134.8, 132.5, 129.0, 128.8, 128.6, 128.4, 128.3, 120.7, 119.5, 119.0, 118.3, 114.0, 112.5, 109.9, 80.7, 74.6, 73.2, 53.5, 22.5, 21.4, 14.0.

HRMS (ESI): calculated for $[M+H]^+$: 790.2620, found: 790.2606.

2 Biology

2.1 Materials and Methods

MIC testing

Strains were cultivated according to standard procedures. Bacterial cryo-cultures were plated on fresh CASO agar and incubated at appropriate conditions for 24 hours. The following day, 1-2 colonies were picked and suspended into 0.9% NaCl (Merck KGaA, Darmstadt, Germany) to reach a McFarland value of 0.2 - 0.5. The suspension was resuspended into fresh MHBII (cation adjusted) medium. The resuspension corresponding to approximately 5 x 10^6 colony-forming units (CFU)/mL. The test culture (75 µL) was added to 75 µL of a serial dilution of the test compounds in 96 well assay plate (Corning, #3788). All compound were used from a 5 mg/ml stock solution and were tested at final starting concentrations of 64 µg/ml. Ciprofloxacin was tested in parallel as a positive control. The highest DMSO concentration in the assay was 1%, which had no growth effect.

In the case of obviously ineffective cystobactamids the MIC-panel was not tested completely.

Determination of in vitro ADME properties

The plasma stability assay was conducted as described previously with procaine, procainamide and propoxycaine as controls.[21] The plasma protein binding assay and the metabolic stability assay were conducted as described previously with naproxen and verapamil as controls, respectively.[22] In vitro ADME assays were conducted for **4, 7, 8, 11** and **42**. All samples were analysed via HPLC-MS/MS.

HPLC-MS/MS analysis of cystobactamids

Samples were analyzed using an Agilent 1290 Infinity II HPLC system coupled to an AB Sciex QTrap 6500plus mass spectrometer. LC conditions were as follows: column: Agilent Zorbax Eclipse Plus C18, 50 × 2.1 mm, 1.8 µm; temperature: 30 °C; injection volume: 1 µL per sample; flow rate: 700 µL min^{-1}. Samples were run under acidic conditions. Solvents: A: water + 0.1% formic acid; solvent B: 95% acetonitrile/5% H_2O + 0.1% formic acid. The following gradient was applied: 99% A at 0 min, 99% A until 0.1 min, 99–50% A from 0.1 min to 3.5 min, 50-0% A from 3.5 min until 3.8 min, 0-99%

A from 3.8 min until 4.7 min. The mass spectrometer was run in positive and negative mode with multiple reaction monitoring (MRM). Mass transitions for controls and compounds are depicted in Table S3. Samples were analyzed using Multiquant 3.0 software (AB Sciex).

Table S3. Mass transitions of the internal standard (caffeine), controls and cystobactamids.

	Q1 mass	Q3 mass	DP [V]	CE [V]	CXP [V]
**caffeine**	195.024	138.0<br>110.0	130<br>130	25<br>25	14<br>14
**naproxen**	231.106	185.1<br>170.2	80<br>80	19<br>33	10<br>12
**procaine**	235.744	163.0<br>120.0	80<br>80	21<br>39	18<br>12
**procainamide**	236.773	100.0<br>120.0	80<br>80	21<br>31	12<br>14
**propoxycaine**	294.738	100.1<br>178.1	80<br>80	17<br>21	12<br>20
**verapamil**	454.688	165.0<br>303.1	1<br>1	35<br>35	28<br>18
**4**	793.092	297.9<br>341.9	-80<br>-80	-50<br>-38	-41<br>-25
**7**	790.151	431.0<br>263.9	-245<br>-245	-52<br>-46	-13<br>-21
**8**	776.012	264.0<br>297.8	-270<br>-270	-44<br>-44	-31<br>-7
**11**	789.022	387.2<br>505.1	-240<br>-240	-58<br>-48	-19<br>-25
**42**	791.167	431.9<br>748.1	-175<br>-175	-52<br>-38	-21<br>-39

DP: declustering potential; CE: collision energy; CXP: collision cell exit potential.

Table S4. In vitro ADME properties of selected cystobactamid derivatives.

	PPB (M) [%]	PPB (H) [%]	T1/2 PLASMA (M) [MIN]	T1/2 PLASMA (H) [MIN]	T1/2 MICROSOMES (M) [MIN]	T1/2 MICROSOMES (M) [MIN]
4	100	100	> 240	> 240	> 60	> 60
7	100	100	> 240	> 240	> 60	> 60
8	100	100	> 240	> 240	> 60	> 60
11	100	100	> 240	> 240	> 60	> 60
42	100	100	> 240	> 240	> 60	> 60

Human and mouse plasma

For plasma stability experiments, samples were procured from commercial sources. Human plasma was obtained from Antibodies-online (catalog no. ABIN5706569), and mouse plasma was obtained from Tebubio (catalog no. D408-04-0050).

The methods were performed in accordance with relevant guidelines and regulations and approved by the Helmholtz Centre for Infection Research.

Animals

For the pharmacokinetic experiment, outbred, male CD-1 mice (Charles River, Germany), 4-weeks-old, were used. The animal studies were conducted in accordance with the recommendations of the European Community (Directive 2010/63/EU, 1st January 2013). All animal procedures were performed in strict accordance with the German regulations of the Society for Laboratory Animal Science (GV-SOLAS) and the European Health Law of the Federation of Laboratory Animal Science Associations (FELASA). Animals were excluded from further analysis if sacrifice was necessary according to the human endpoints established by the ethical board. The PK study was approved by the ethical board of the Niedersächsisches Landesamt für Verbraucherschutz und Lebensmittelsicherheit, Oldenburg, Germany.

PK study

Compound **42** was administered at 1 mg/kg intravenously (IV). Up to 25 µl blood was collected from the lateral tail vein (n=2 per time point) and time points t= 0.25, 0.5, 1 and 3 hours.At t= 5 hours animals were euthanized to collect blood. Whole blood was collected into Eppendorf tubes coated with 0.5 M EDTA and immediately spun down at 15870 x *g* for 10 min at 4°C. Then, plasma was transferred into a new Eppendorf tube and stored at -80°C until analysis.

Bioanalysis of pharmacokinetic and pharmacodynamic samples

First, a calibration curve was prepared by spiking different concentrations of **42** into mouse plasma from CD-1 mice. Glipizide was used as an internal standard. In addition, quality control samples (QCs) were prepared for **42** in plasma. The following extraction procedure was used: 7.5 µl of a plasma sample (calibration samples, QCs, PK samples) was extracted with 35 µl of a mixture of acetonitrile containing 12.5 ng/ml of glipizide as internal standard for 10 min at 2000 rpm on an Eppendorf MixMate® vortex mixer. Then samples were spun down at 15870 x *g* for 10 min. Supernatants were

transferred to standard HPLC-glass vials. Samples were analyzed using LC-MS/MS conditions as described above. Peaks of PK samples were quantified using the calibration curve using AB Sciex Multiquant Software 3.0. The accuracy of the calibration curve was determined using QCs independently prepared. PK parameters were determined using a non-compartmental analysis with PKSolver.[23]

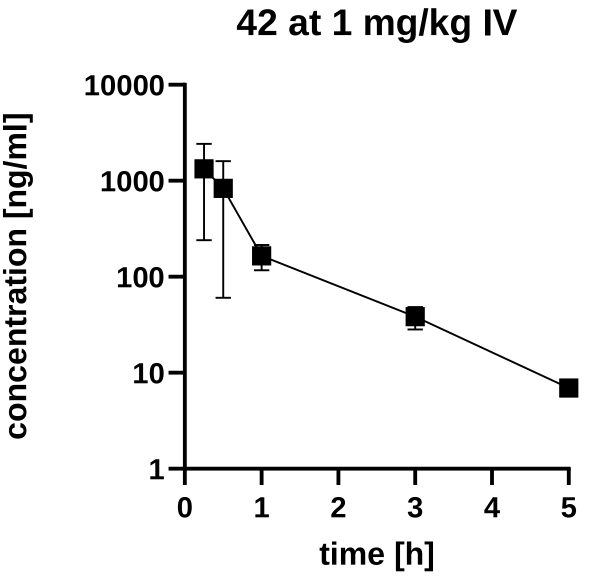

Figure S1: Plasma concentration curves for cystobactamid 42. Error bars were determined via standard deviation of two cassette experiments per compound (n=2).

AlbD enzyme assay

The AlbD-enzyme was isolated according to literature.[13] 120 µM test cystobactamids were incubated with 5 µM recombinant AlbD in 0.2 M phosphate buffer (pH 7.0).

Table S5: Pipet scheme used in the AlbD enzyme assay

Component \ Entry		Neg. Control	DMSO +AlbD	+**42**	+**42** +AlbD	+**11**	+**11** +AlbD	+**96** (CNDM 861)	+**96** (CNDM 861) +AlbD
phosphate buffer (pH=7.0)[a]		100 µL	79.6 µL	98.1 µL	79.6 µL	98.1 µL	79.6 µL	98.8 µL	80.3 µL
AlbD[b]		-	18.5 µL	-	18.5 µL	-	18.5 µL	-	18.5 µL
Compound to test	**42** (6.3 mM stock)	-	-	1.9 µL	1.9 µL	-	-	-	-
	**11** (6.3 mM stock)	-	-	-	-	1.9 µL	1.9 µL	-	-
	**96** (10 mM stock)	-	-	-	-	-	-	1.2 µL	1.2 µL
	DMSO	1.9 µL	-	-	-	-	-	-	-
	100 µL	100 µL	100 µL	100 µL	100 µL	100 µL	100 µL	100 µL	100 µL

[a] c=0.200 M, prepared from Na_2HPO_4 (anhydrous, 5.469 g) and $NaH_2PO_4*H_2O$ (1.587 g) in water (250 mL). [b] c(AlbD)=27 µM, in TrisHCl/ NaCl/ glycerol-buffer. [13]

After preparing the enzymatic reactions according to Table S and brief vortexing, the assay mixtures were incubated on a thermo-shaker, 28°C, 600 rpm, 24 h. Subsequently, the mixtures were shortly centrifuged. MeOH (350 µL) was added to stop the enzymatic reaction. The tubes were vortexed again and then centrifuged at 20000 g, 20°C for at least 20 min. Supernatant (250 µL) was carefully transferred to new vials and put in a speed vac at 30°C for 4 h to dry. The samples with glycerol residue were repeatedly coevaporated by diluting with MeOH (3x20 µL), followed by sonification and drying again with the speedvac (30°C, 15-30 min). MeOH (100 µL) was added to the dried samples, showing a precipitate afterwards. After thoroughly mixing the suspensions were centrifuged at 20000 g, 20°C for 10 min. The supernatant (50 µL) was transferred to an LCMS glass vial and measured by HPLC MS using following conditions:

Column: Phenomenex Kinetex 1.7µm, C18, 100A, 150x2.1mm

Gradient: 20min, 1-100% ACN/H_2O, with 0.1% HCOOH

Flow rate: 0.3 mL/min

UV/Vis-range: 190-400 nm

HPLC device: Thermo Scientific Dionex Ultimate 3000

HRMS-spectrometer: Bruker Maxis HD

Bruker Data Analysis software (Bruker Daltonics, Bremen, Germany) was used for data analysis.

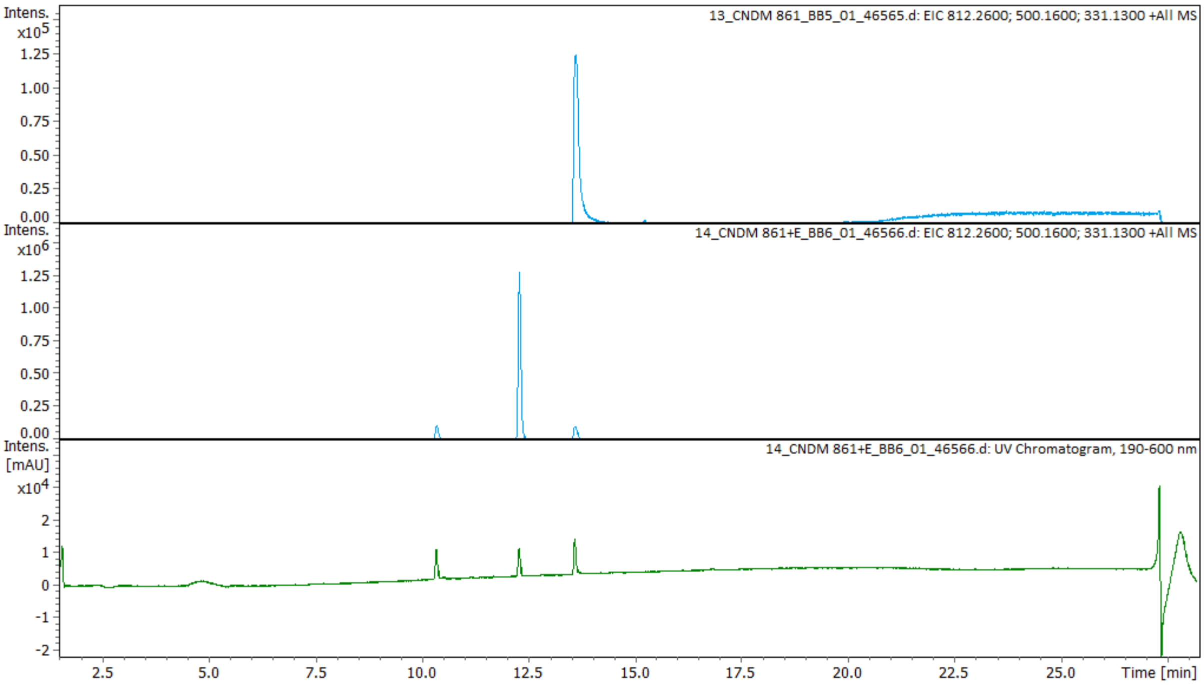

Figure S2: Stability of compound 96 to AlbD: before (top) and after exposure at 28°C, 24 h with extracted ion chromatogram and UV chromatogram (middle and bottom).

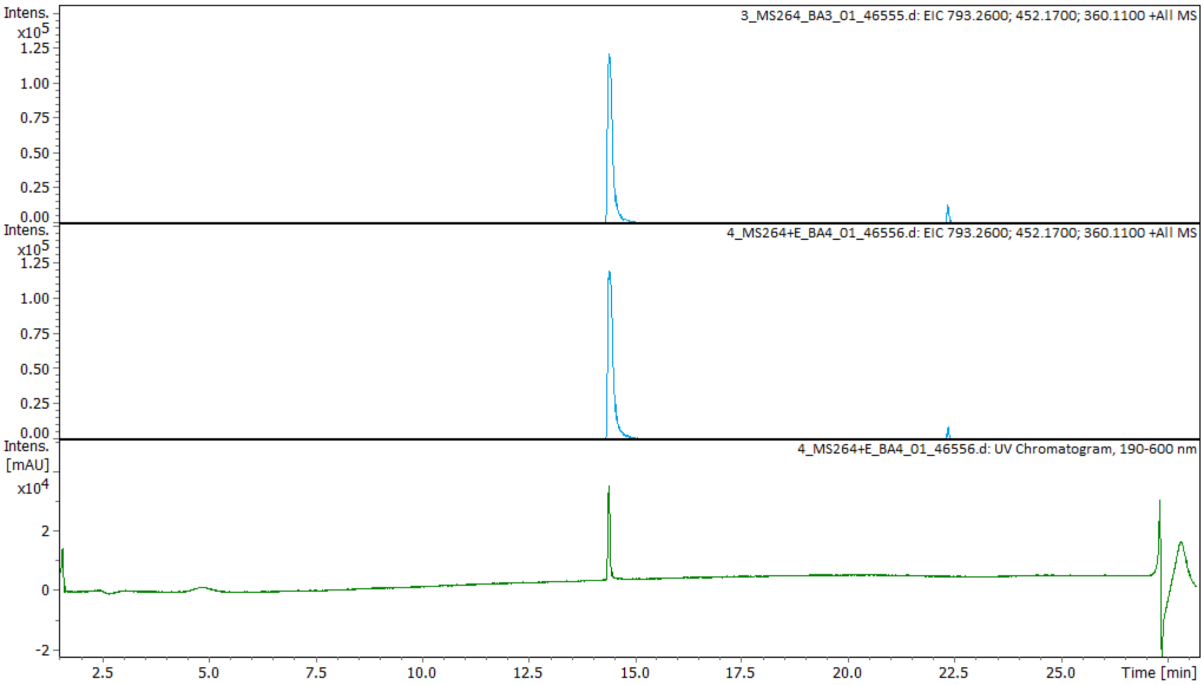

Figure S3: Stability of compound 42 to AlbD: before (top) and after exposure at 28°C, 24 h with extracted ion chromatogram and UV chromatogram (middle and bottom).

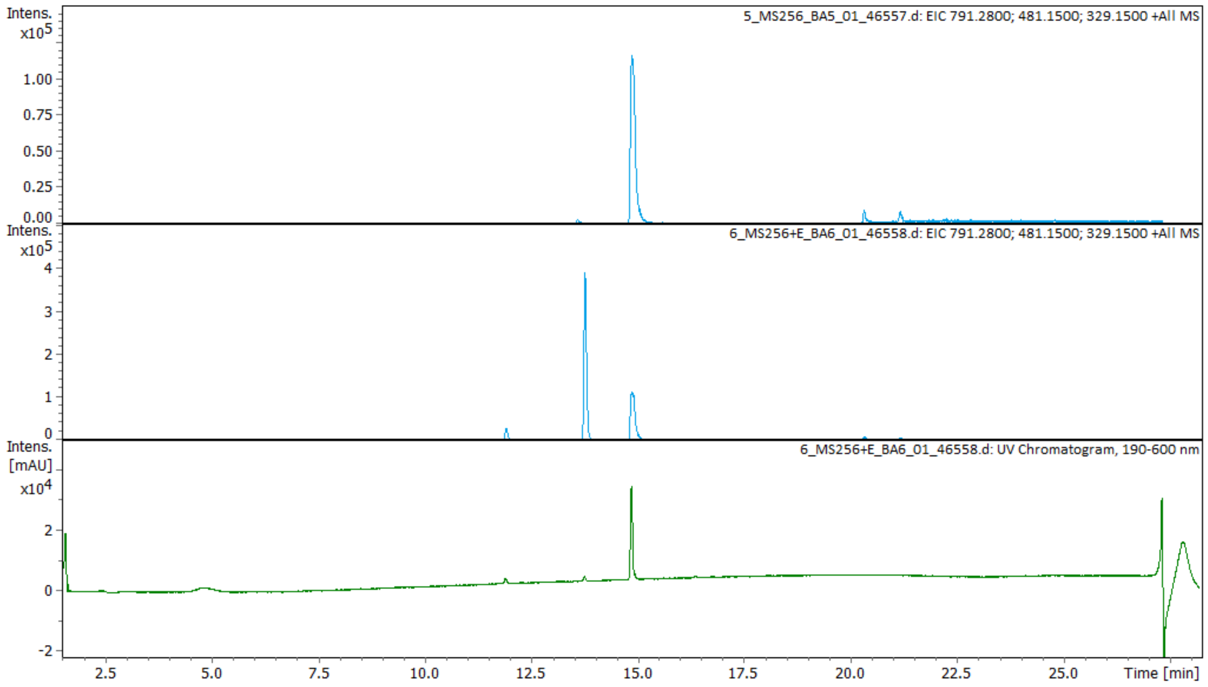

Figure S4: Stability of compound 11 to AlbD: before (top) and after exposure at 28°C, 24 h with extracted ion chromatogram and UV chromatogram (middle and bottom).

3 Supplementary references

1. Fulmer GR*, et al.* NMR Chemical Shifts of Trace Impurities: Common Laboratory Solvents, Organics, and Gases in Deuterated Solvents Relevant to the Organometallic Chemist. *Organometallics* **29**, 2176-2179 (2010).
2. Andrés A*, et al.* Setup and validation of shake-flask procedures for the determination of partition coefficients (logD) from low drug amounts. *Eur J Pharm Sci* **76**, 181-191 (2015).
3. Valkó K. *Separation methods in drug synthesis and purification*. Elsevier (2000).
4. Dong Y, Wang X, Cal M, Kaiser M, Vennerstrom JL. Activity of diimidazoline amides against African trypanosomiasis. *Bioorg Med Chem Lett* **24**, 944-948 (2014).
5. Kohnhäuser D*, et al.* Optimization of the central α-amino acid in cystobactamids to the broad-spectrum, resistance-breaking antibiotic CN-CC-861. *Submitted*, (2024).
6. Moeller M*, et al.* Scalable Syntheses of Methoxyaspartate and Preparation of the Antibiotic Cystobactamid 861-2 and Highly Potent Derivatives. *Org Lett* **21**, 8369-8372 (2019).
7. Prabhakaran P, Barnard A, Murphy NS, Kilner CA, Edwards TA, Wilson AJ. Aromatic Oligoamide Foldamers with a “Wet Edge” as Inhibitors of the α-Helix-Mediated p53–hDM2 Protein–Protein Interaction. *Eur J Org Chem* **2013**, 3504-3512 (2013).
8. Marimganti S, Cheemala MN, Ahn J-M. Novel Amphiphilic α-Helix Mimetics Based on a Bis-benzamide Scaffold. *Org Lett* **11**, 4418-4421 (2009).
9. Kamenecka TM*, et al.* Preparation of biphenylpropan-2-ol derivatives as modulators of the nuclear hormone receptor ROR Patent WO2012158784 (2012).
10. Hüttel S*, et al.* Discovery and Total Synthesis of Natural Cystobactamid Derivatives with Superior Activity against Gram-Negative Pathogens. *Angew Chem, Int Ed* **56**, 12760-12764 (2017).
11. Elgaher WAM*, et al.* Cystobactamid 507: Concise Synthesis, Mode of Action, and Optimization toward More Potent Antibiotics. *Chem Eur J* **26**, 7219-7225 (2020).
12. Yang F*, et al.* Synthesis, structure–activity relationships, and biological evaluation of a series of benzamides as potential multireceptor antipsychotics. *Bioorg Med Chem Lett* **26**, 3141-3147 (2016).
13. Testolin G*, et al.* Synthetic studies of cystobactamids as antibiotics and bacterial imaging carriers lead to compounds with high in vivo efficacy. *Chem Sci* **11**, 1316-1334 (2020).
14. Kretz J*, et al.* Total Synthesis of Albicidin: A Lead Structure from Xanthomonas albilineans for Potent Antibacterial Gyrase Inhibitors. *Angew Chem Int Ed* **54**, 1969-1973 (2015).
15. Skowronska-Ptasinska M, Verboom W, Reinhoudt DN. Effect of different dialkylamino groups on the regioselectivity of lithiation of O-protected 3-(dialkylamino)phenols. *J Org Chem* **50**, 2690-2698 (1985).
16. Gould SJ, Eisenberg RL. Studies on nitrogen metabolism using carbon-13 NMR spectroscopy. 6. Biosynthesis of sarubicin A. Synthesis and incorporation of 6-hydroxy[13CO15NH2]anthranilamide. *J Org Chem* **56**, 6666-6671 (1991).
17. Chung J, Capani JS, Jr., Göhl M, Roosen PC, Vanderwal CD. Enantioselective Syntheses of Wickerols A and B. *J Am Chem Soc* **145**, 6486-6497 (2023).

18. Kohnhäuser D*, et al.* Optimization of the central α-amino acid in cystobactamids to the broad-spectrum, resistance-breaking antibiotic CN-CC-861. (2024).
19. Wu L, Lajkiewicz N, Yao W. Preparation of heteroaryl amide compounds as STING activators Patent WO2020028566 (2020).
20. Ikeda T, Yamazaki T, Tsuchida H. Patent EP2409977 (A1) (2010).
21. Sommer R*, et al.* Glycomimetic, Orally Bioavailable LecB Inhibitors Block Biofilm Formation of Pseudomonas aeruginosa. *J Am Chem Soc* **140**, 2537-2545 (2018).
22. Mała P*, et al.* Discovery of N-β-l-Fucosyl Amides as High-Affinity Ligands for the Pseudomonas aeruginosa Lectin LecB. *J Med Chem* **65**, 14180-14200 (2022).
23. Zhang Y, Huo M, Zhou J, Xie S. PKSolver: An add-in program for pharmacokinetic and pharmacodynamic data analysis in Microsoft Excel. *Computer methods and programs in biomedicine* **99**, 306-314 (2010).

4 NMR Spectra

1D NMR spectra are sorted in the order ^{1}H, ^{13}C, ^{19}F

2D NMR spectra are sorted in the order: ^{1}H-^{1}H HSQC, ^{1}H-^{13}C HMBC, ^{1}H-^{1}H COSY, (^{1}H-^{1}H NOESY, ^{1}H-^{1}H ROESY)

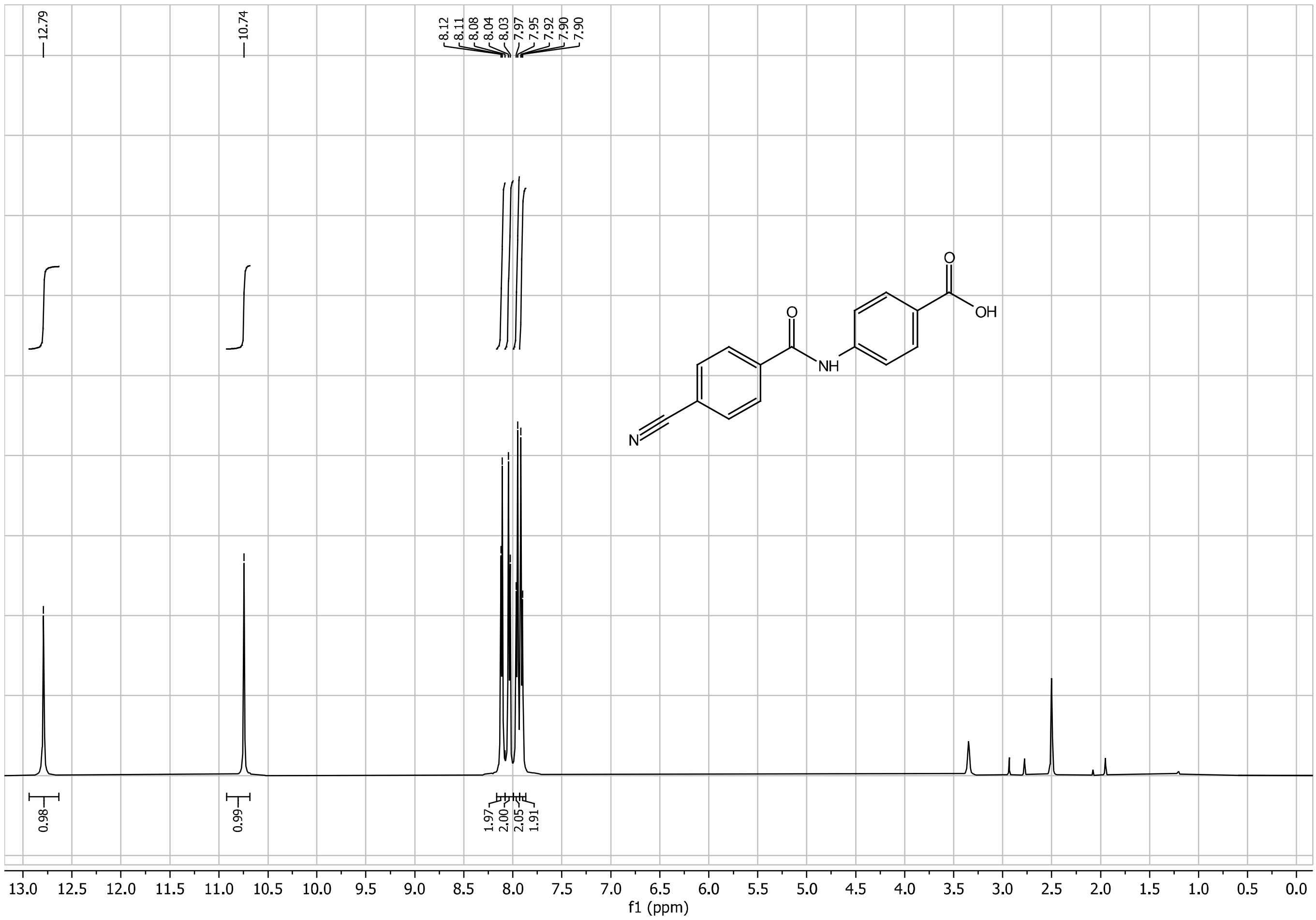

12.79
10.74
8.12 8.11 8.08 8.04 8.03 7.97 7.95 7.92 7.90 7.90
O
OH
O
NH
N
0.98
0.99
1.97 2.00 2.05 1.91
13.0 12.5 12.0 11.5 11.0 10.5 10.0 9.5 9.0 8.5 8.0 7.5 7.0 6.5 6.0 5.5 5.0 4.5 4.0 3.5 3.0 2.5 2.0 1.5 1.0 0.5 0.0
f1 (ppm)

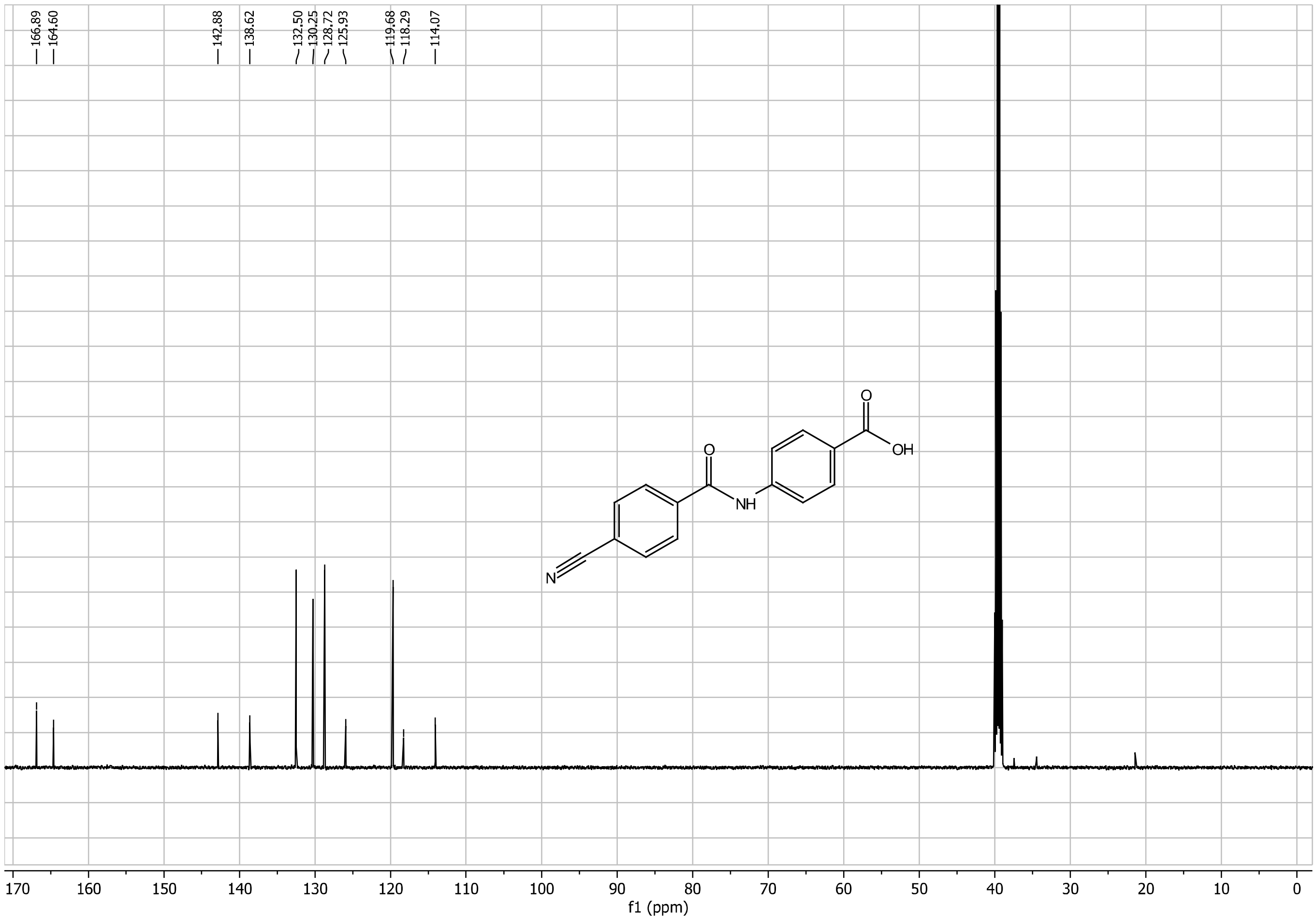

166.89 164.60
142.88
138.62
132.50 130.25 128.72 125.93
119.68 118.29
114.07
O
OH
O
NH
N
170 160 150 140 130 120 110 100 90 80 70 60 50 40 30 20 10 0
f1 (ppm)

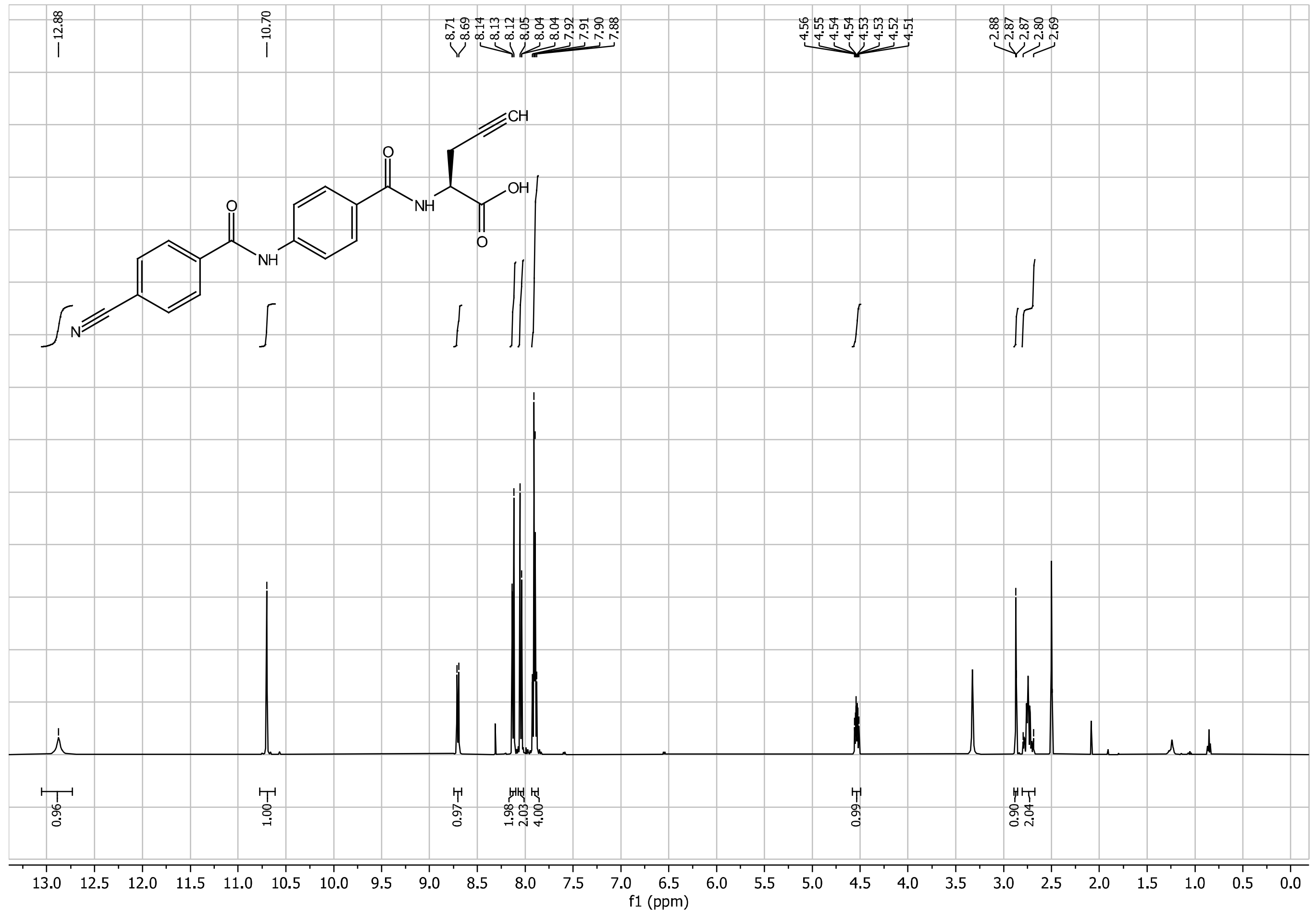

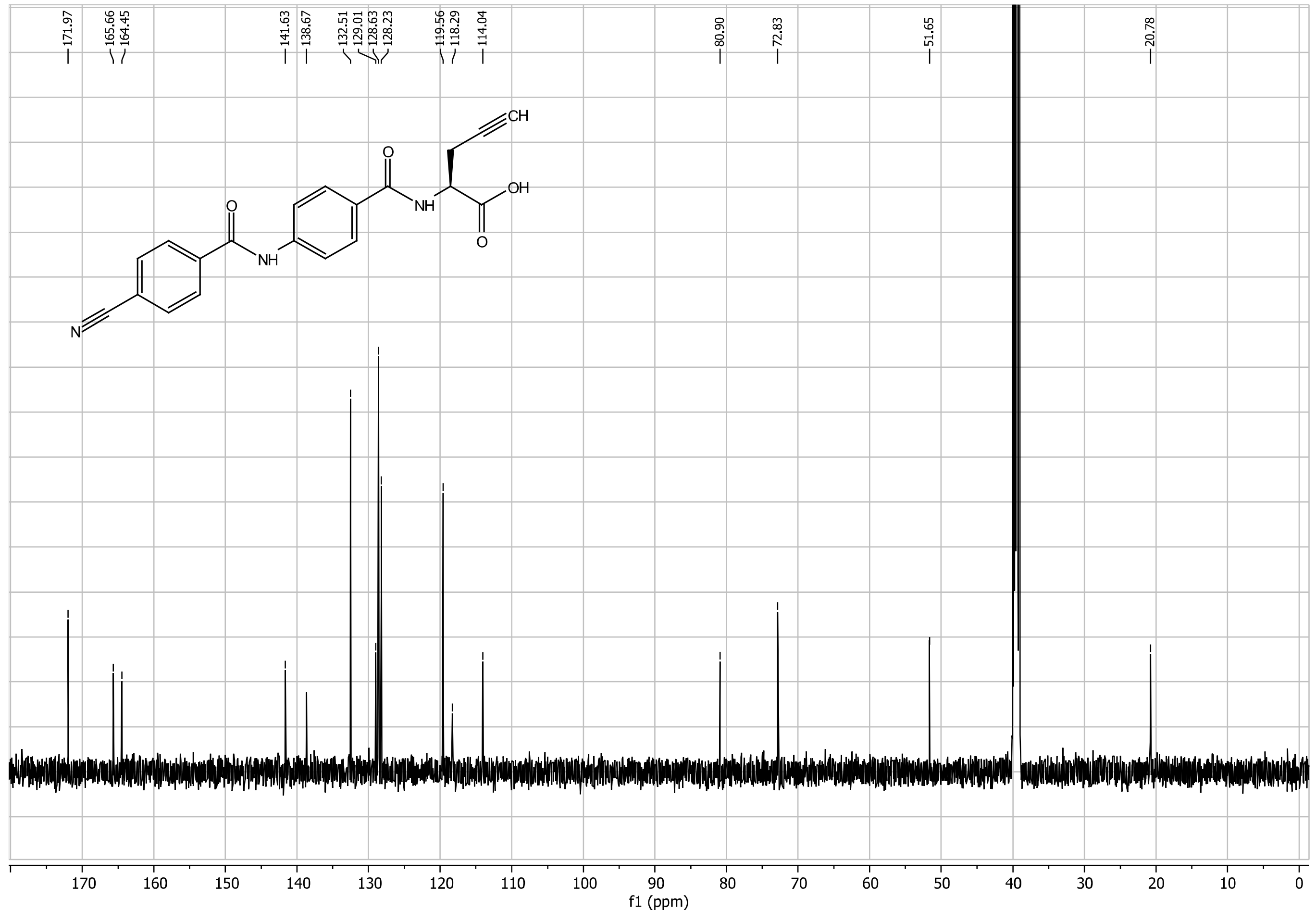

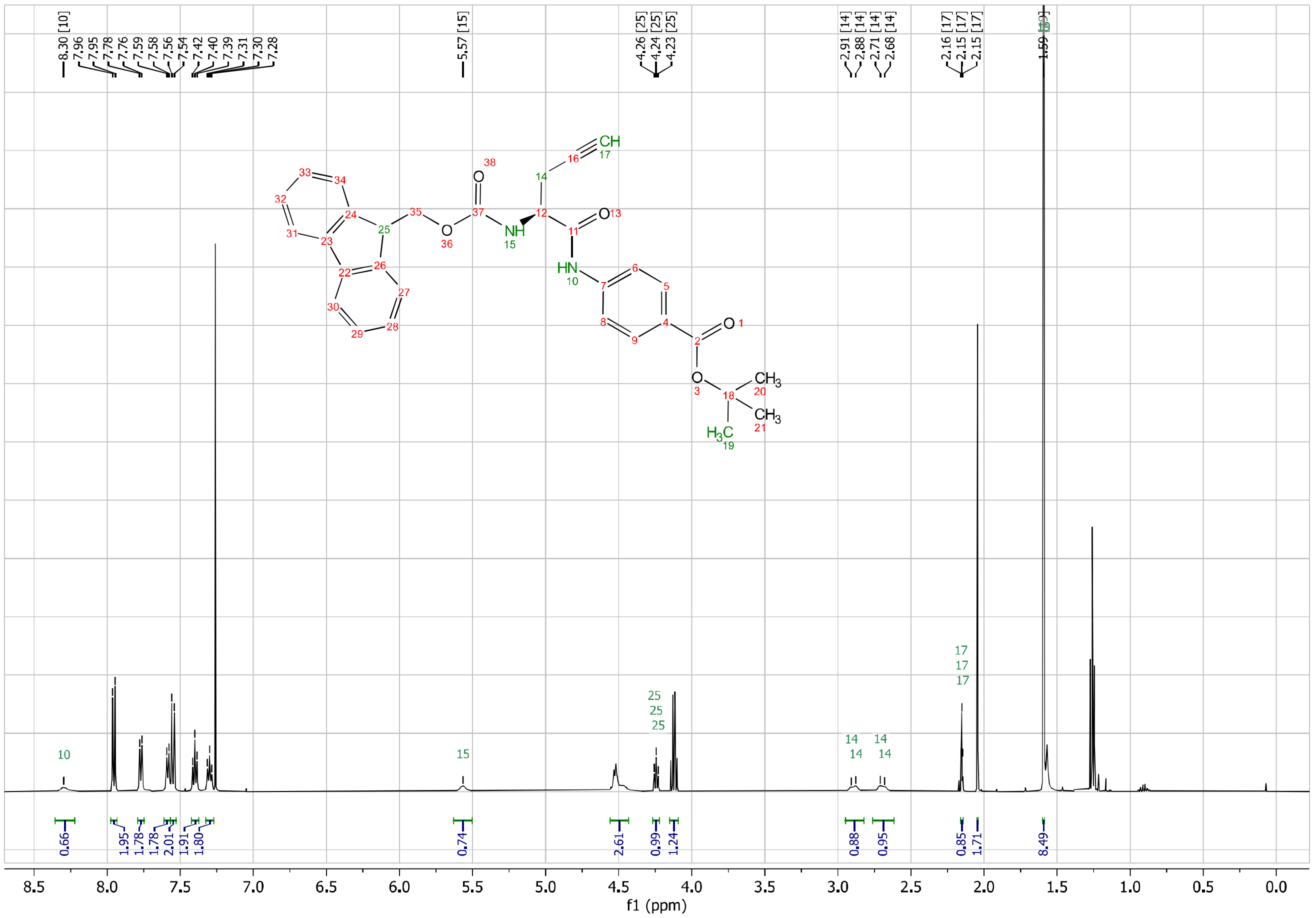

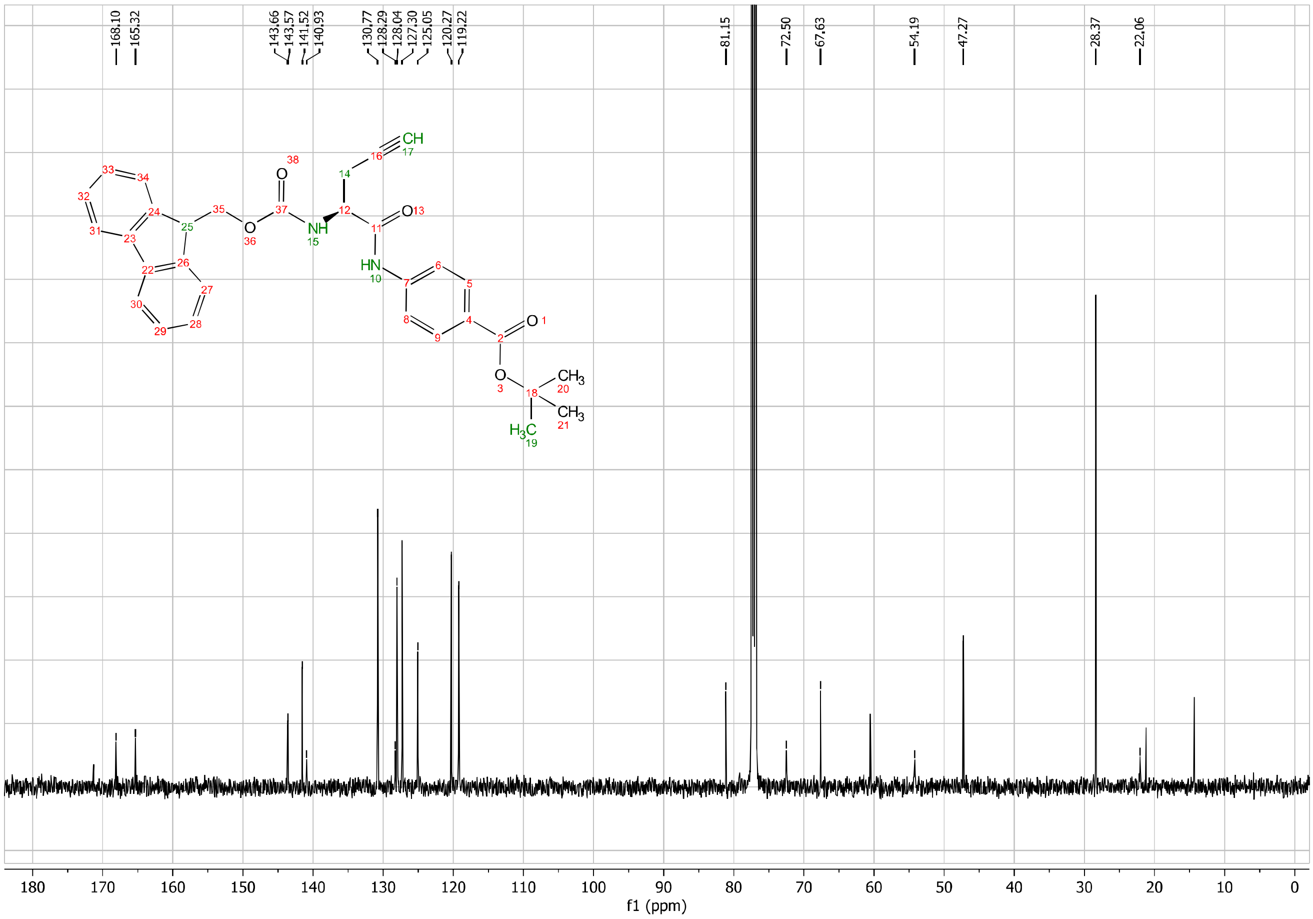

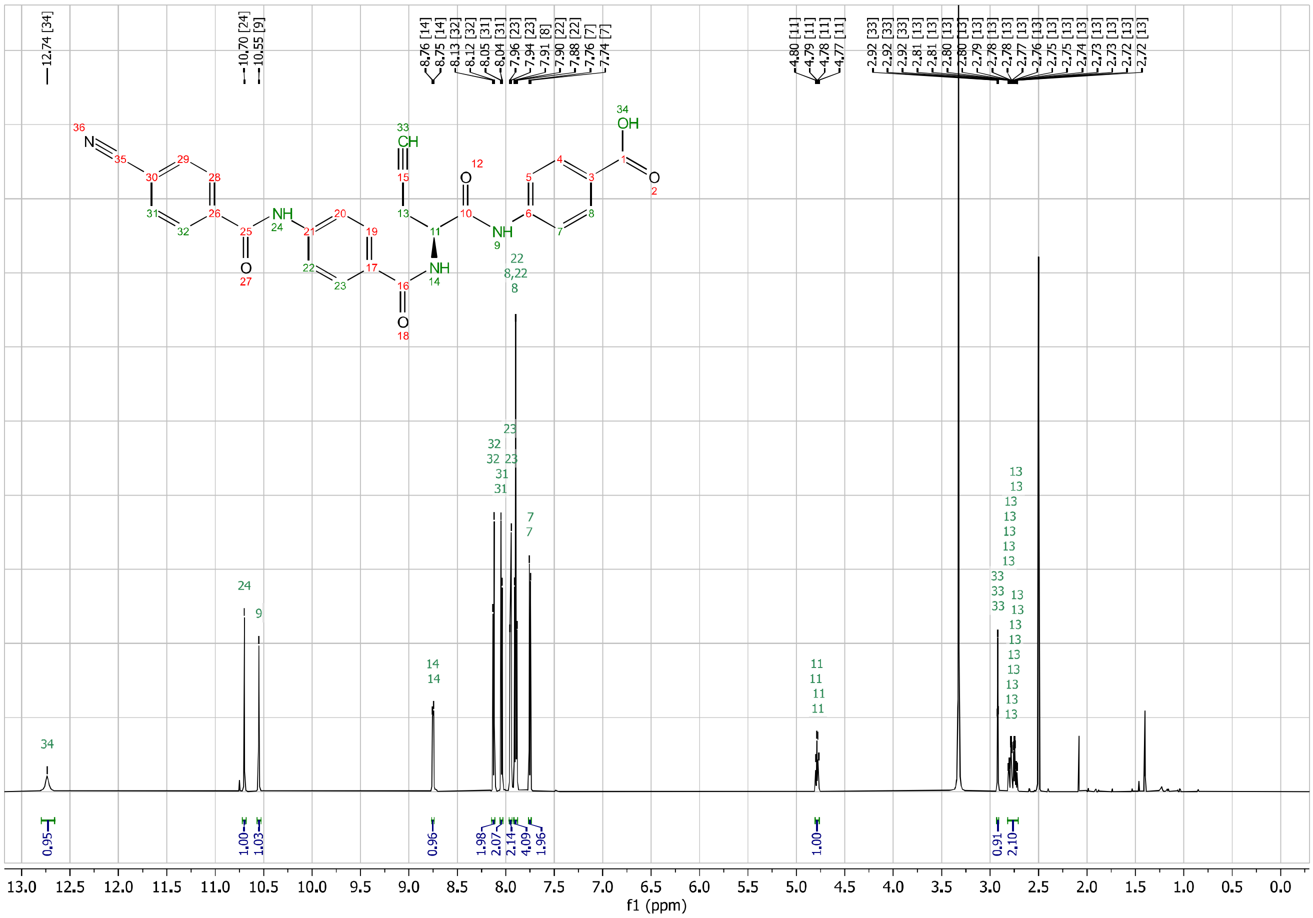

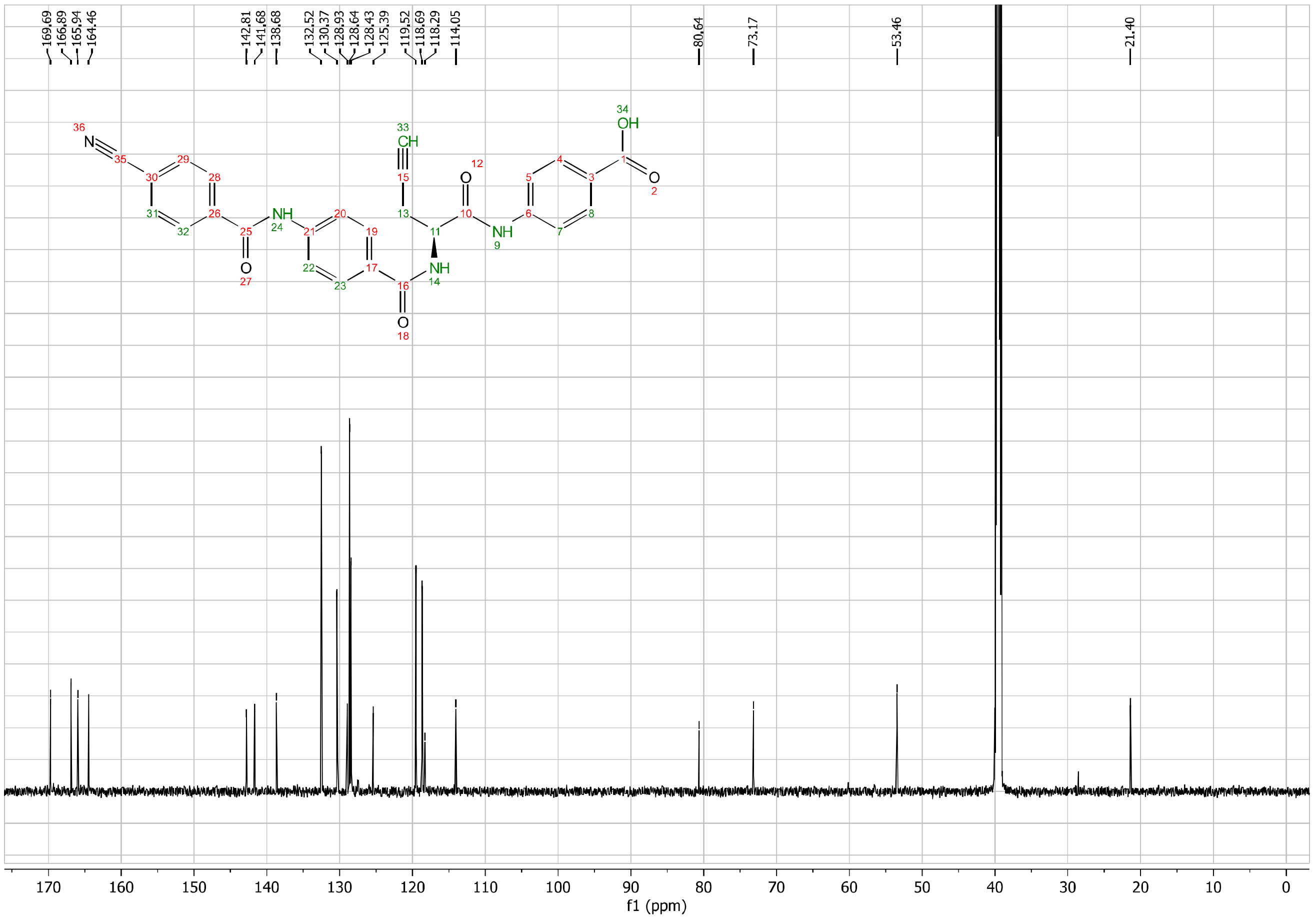

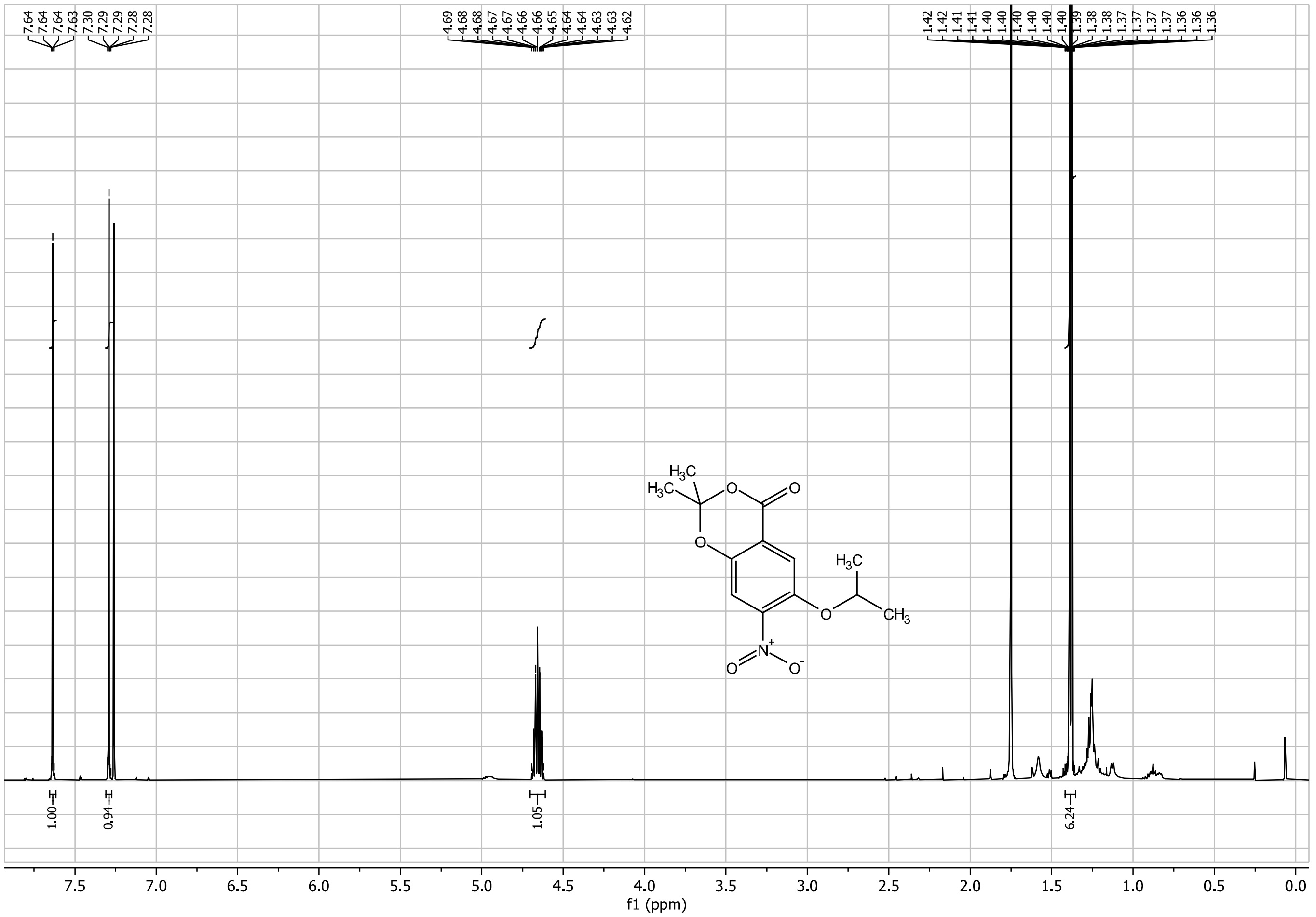

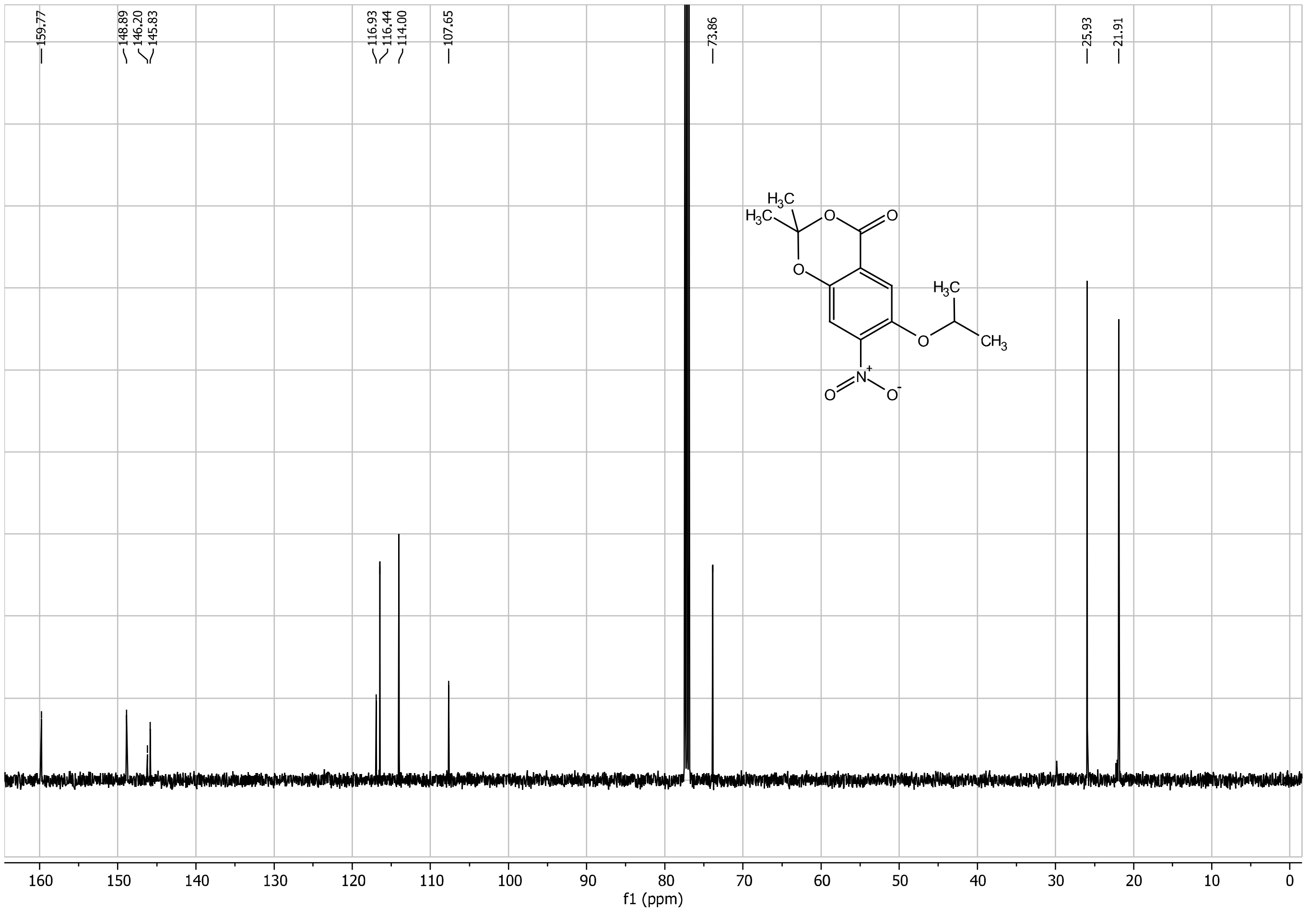

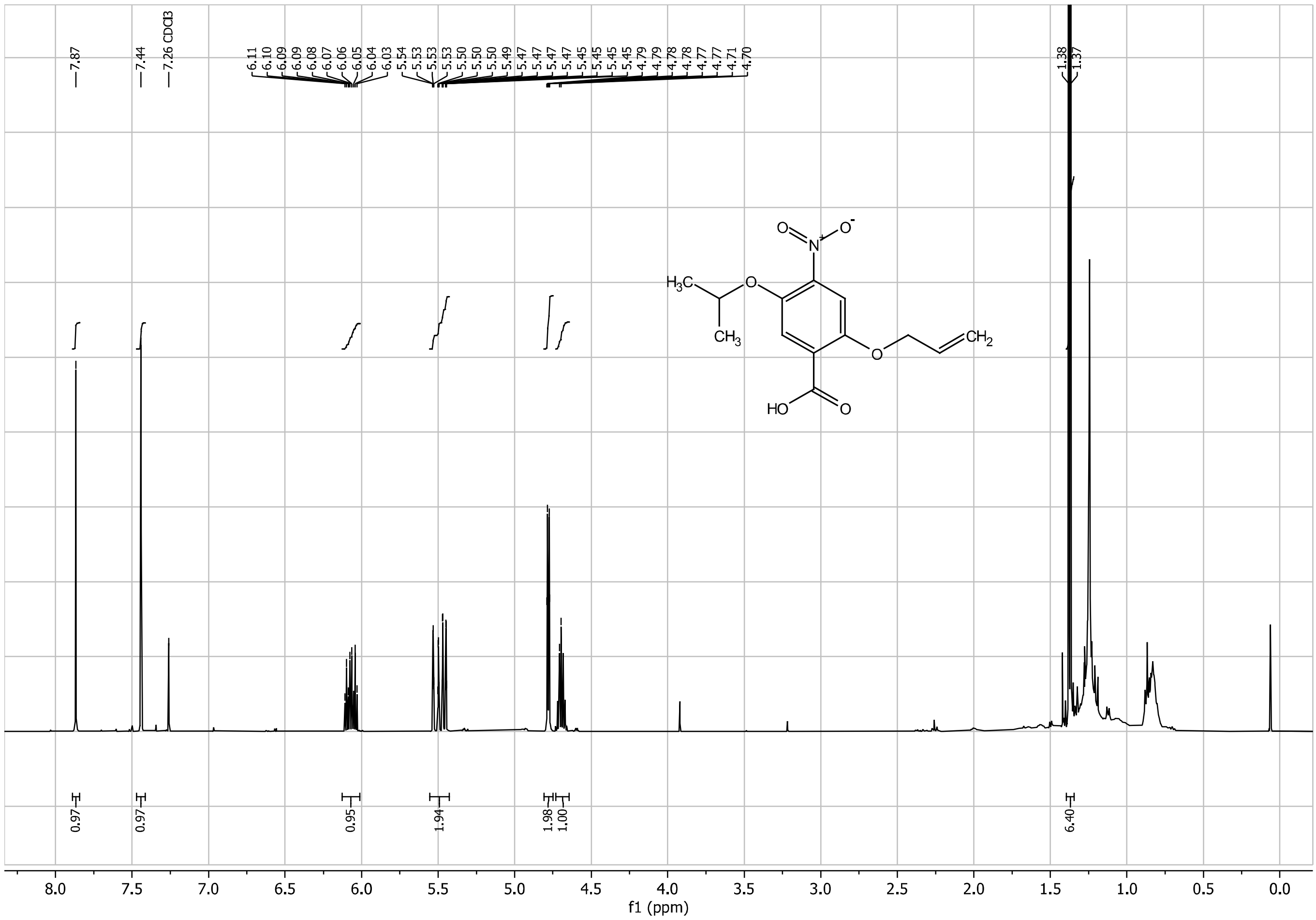
7.87
7.44
7.26 CDCl3
6.11
6.10
6.09
6.09
6.08
6.07
6.06
6.05
6.04
6.03
5.54
5.53
5.53
5.53
5.50
5.50
5.50
5.49
5.47
5.47
5.47
5.45
5.45
5.45
5.45
4.79
4.79
4.78
4.78
4.77
4.77
4.71
4.70
1.38
1.37
0.97
0.97
0.95
1.94
1.98
1.00
6.40
f1 (ppm)

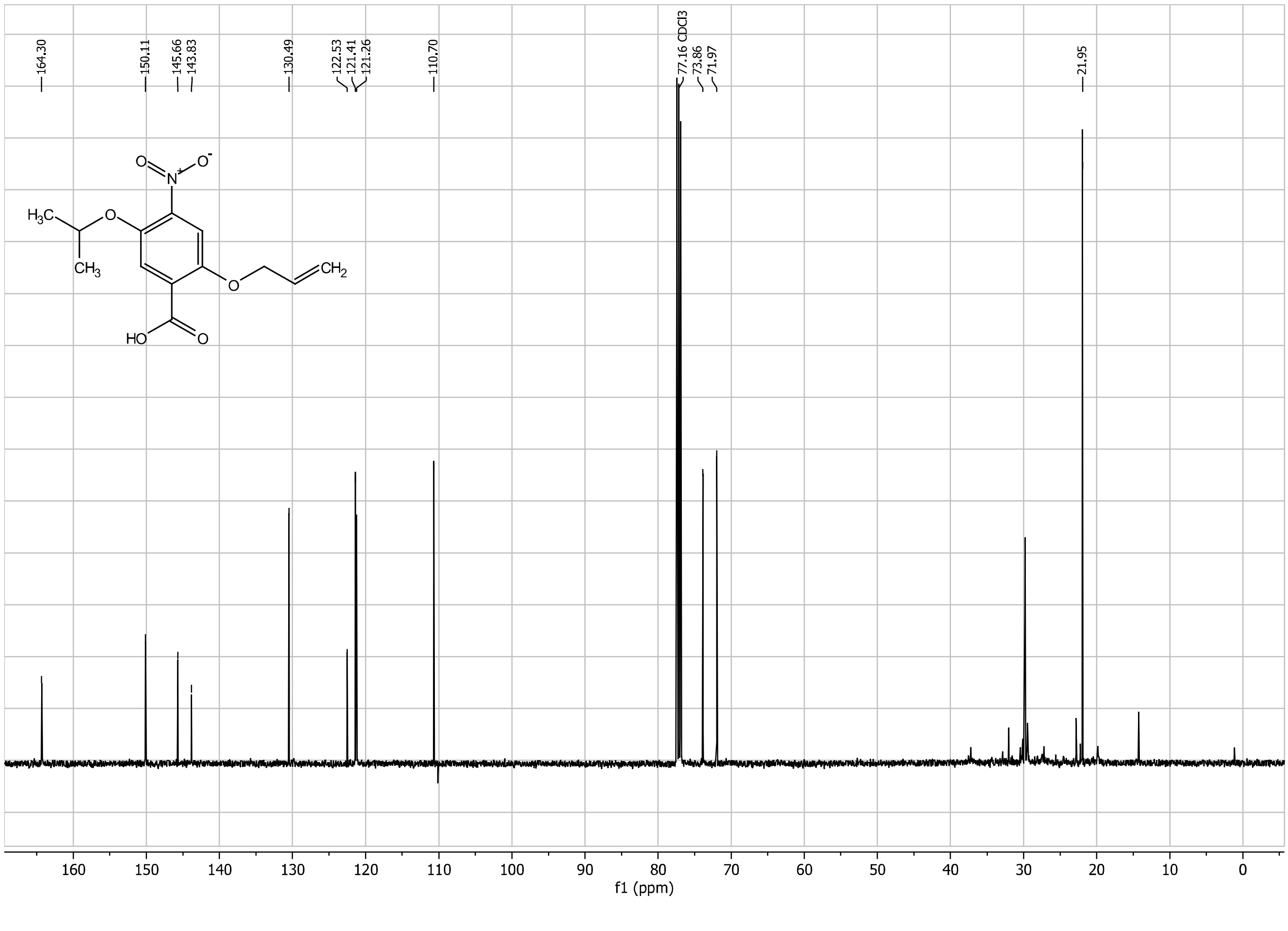
164.30
150.11
145.66
143.83
130.49
122.53
121.41
121.26
110.70
77.16 CDCl3
73.86
71.97
21.95
f1 (ppm)

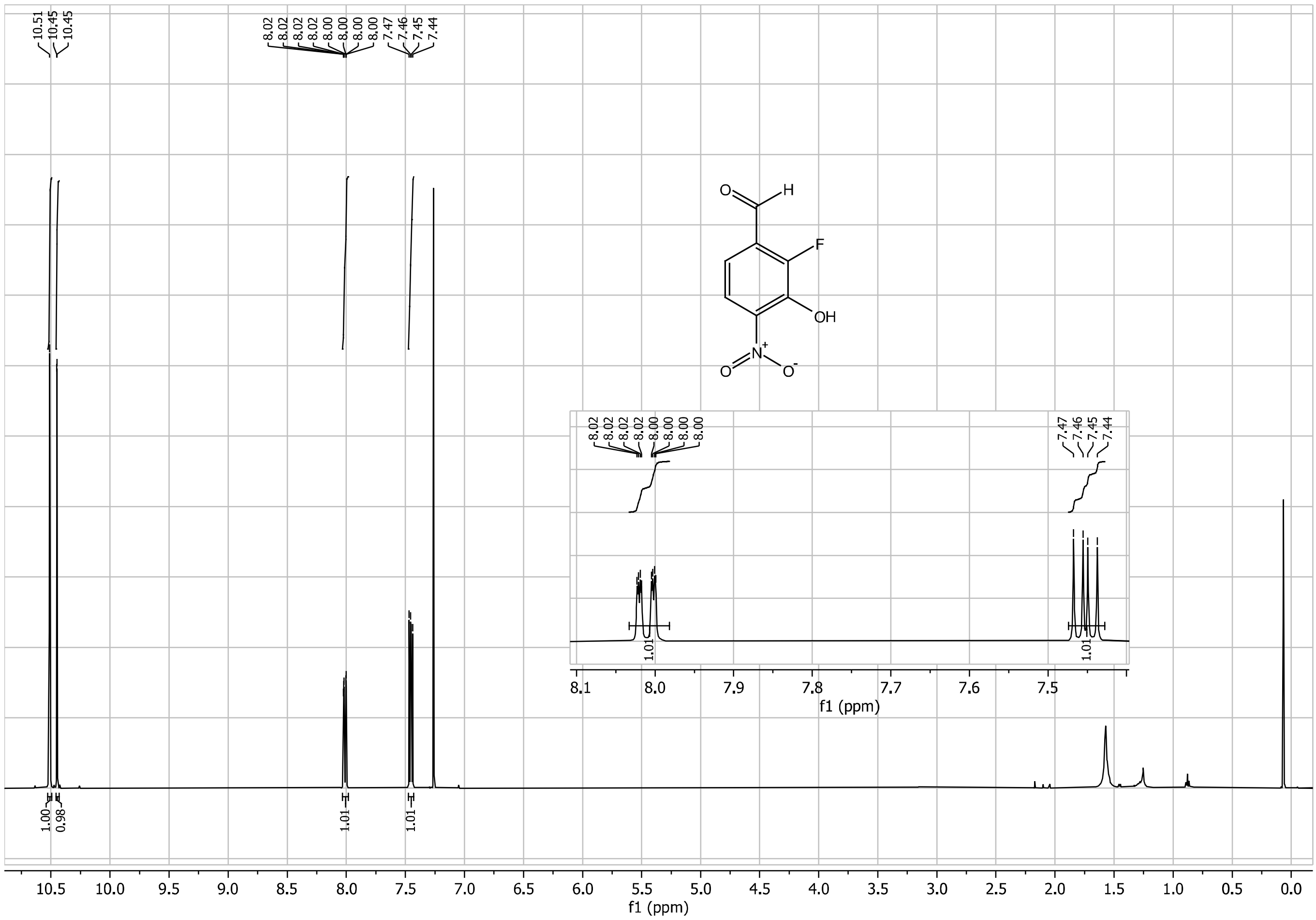
10.51
10.45
10.45
8.02
8.02
8.02
8.02
8.00
8.00
8.00
8.00
7.47
7.46
7.45
7.44
1.00
0.98
1.01
1.01
f1 (ppm)

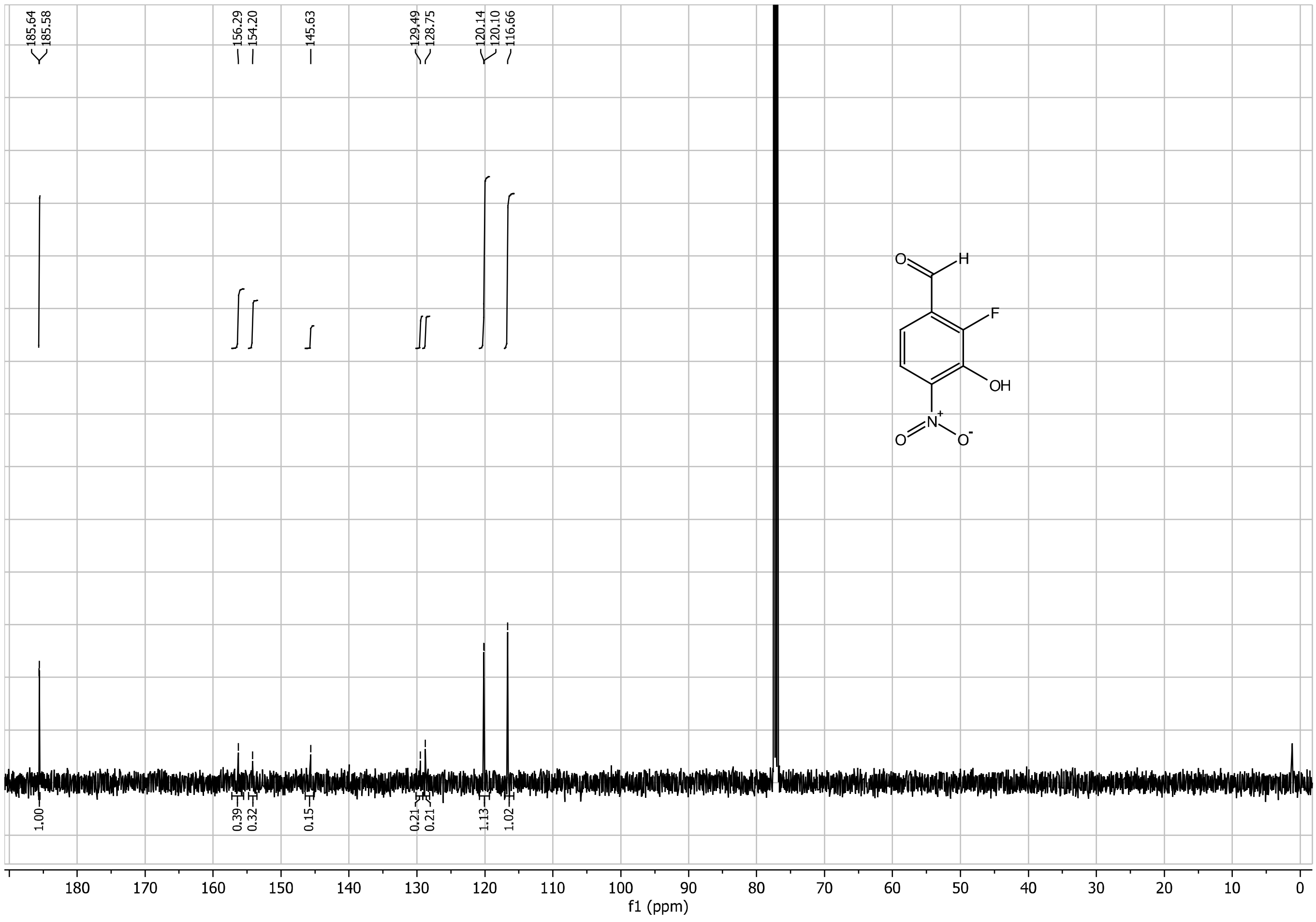
185.64
185.58
156.29
154.20
145.63
129.49
128.75
120.14
120.10
116.66
1.00
0.39
0.32
0.15
0.21
0.21
1.13
1.02
f1 (ppm)

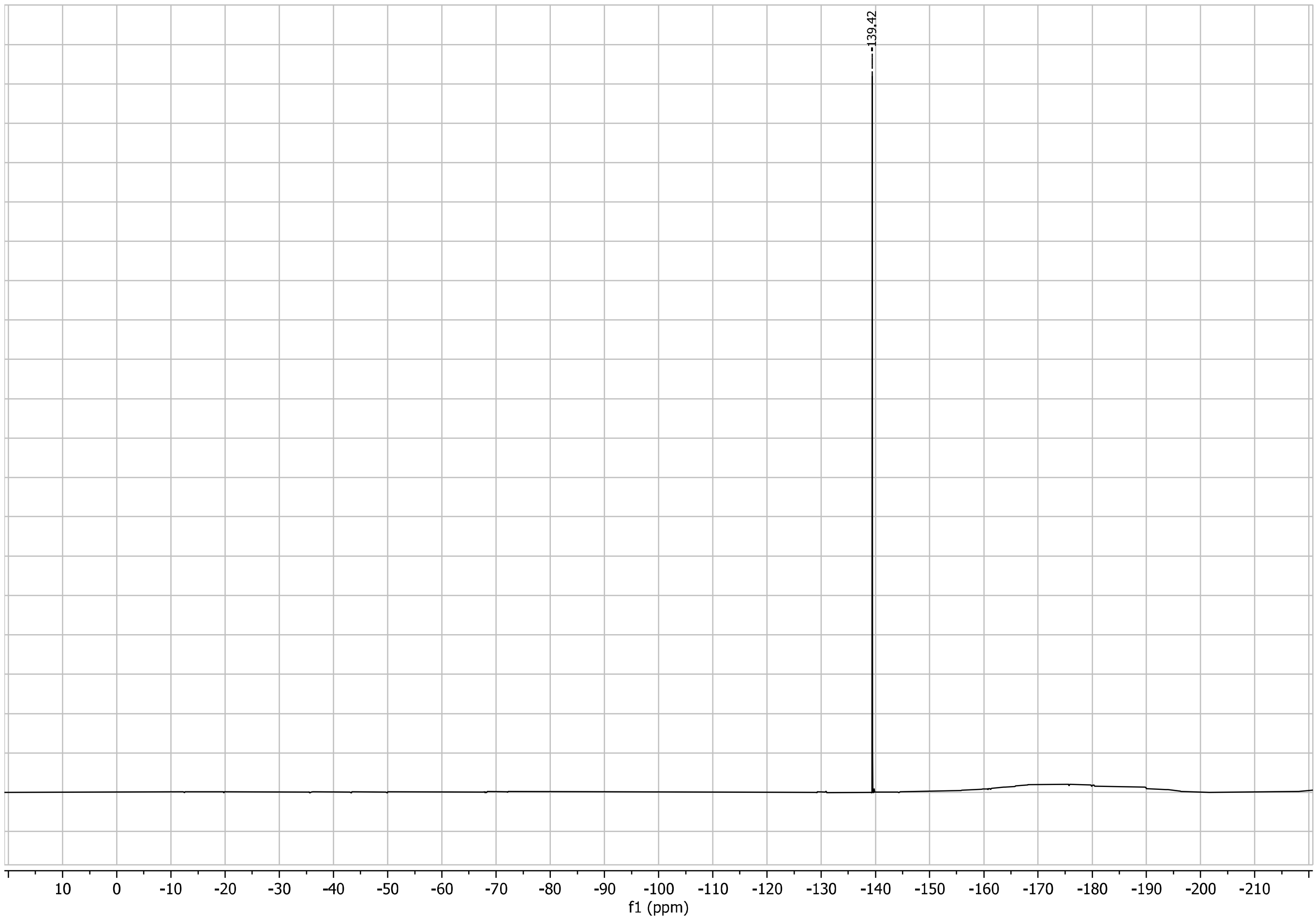

-139.42
10 0 -10 -20 -30 -40 -50 -60 -70 -80 -90 -100 -110 -120 -130 -140 -150 -160 -170 -180 -190 -200 -210
f1 (ppm)

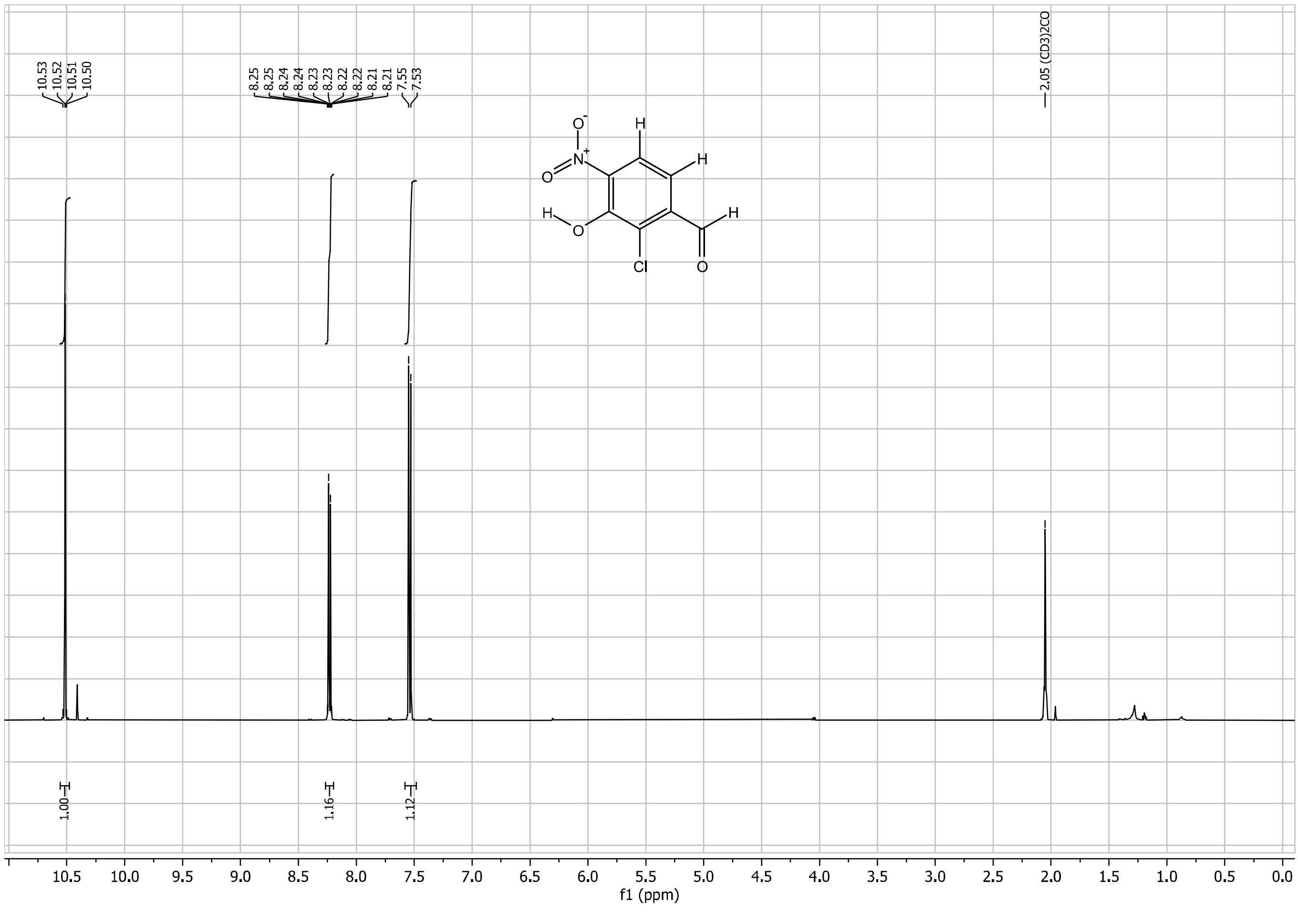

10.53 10.52 10.51 10.50
8.25 8.25 8.24 8.24 8.23 8.23 8.22 8.22 8.21 8.21 7.55 7.53
2.05 (CD3)2CO
Cl
1.00
1.16
1.12
10.5 10.0 9.5 9.0 8.5 8.0 7.5 7.0 6.5 6.0 5.5 5.0 4.5 4.0 3.5 3.0 2.5 2.0 1.5 1.0 0.5 0.0
f1 (ppm)

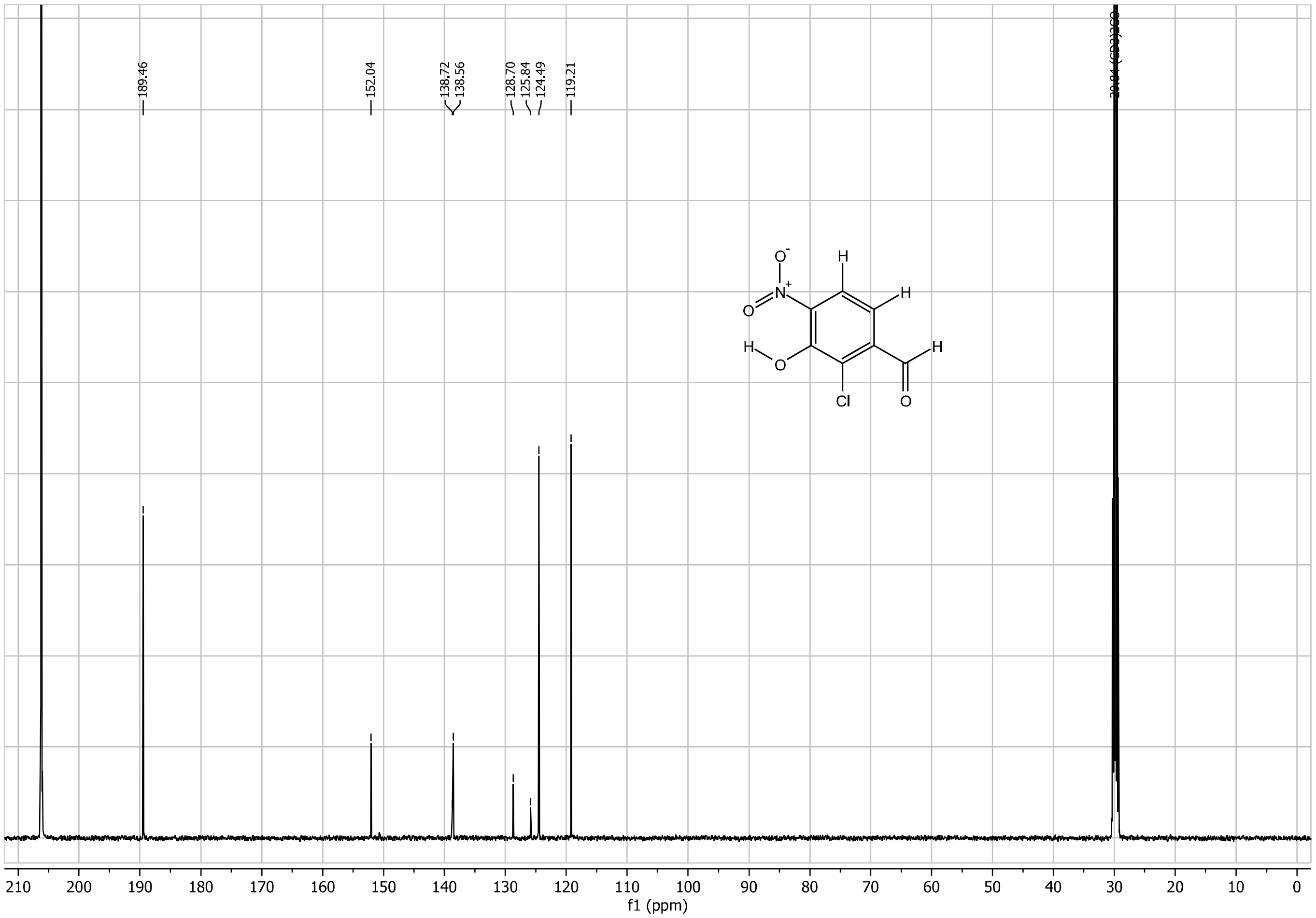
189.46
152.04
138.72
138.56
128.70
125.84
124.49
119.21
f1 (ppm)
Cl
H
O

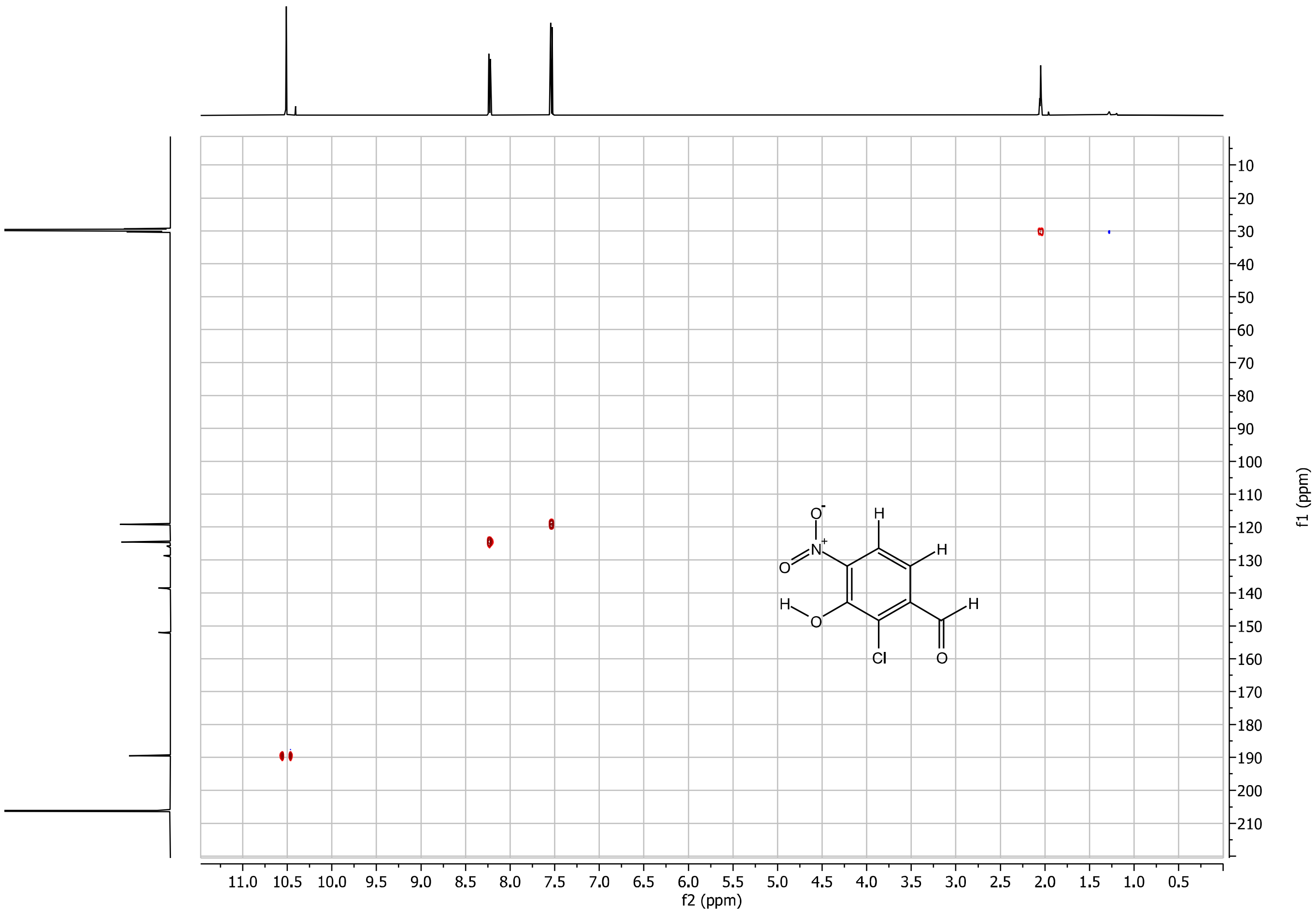
f2 (ppm)
f1 (ppm)
Cl
H
O

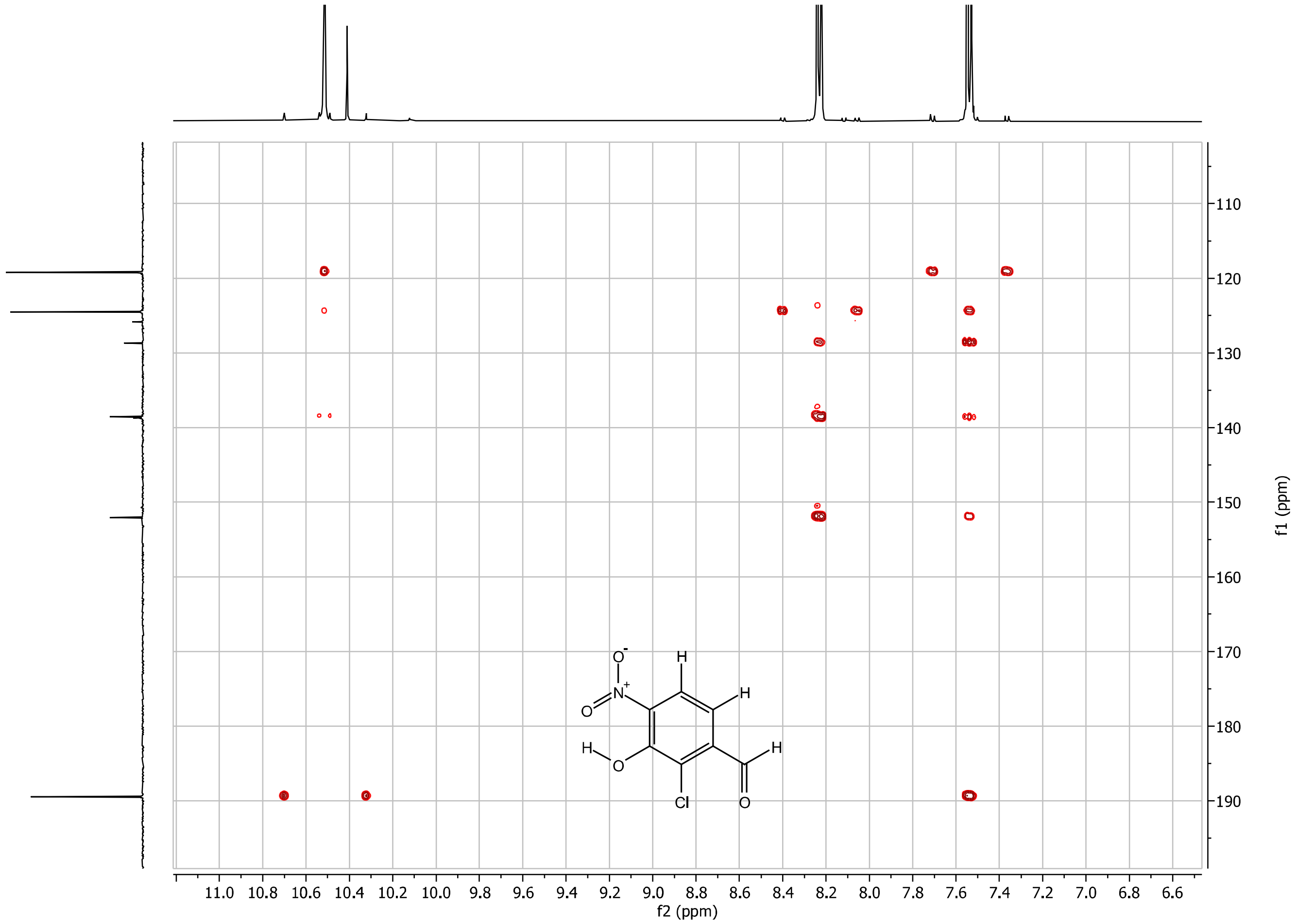
f2 (ppm)
f1 (ppm)
Cl

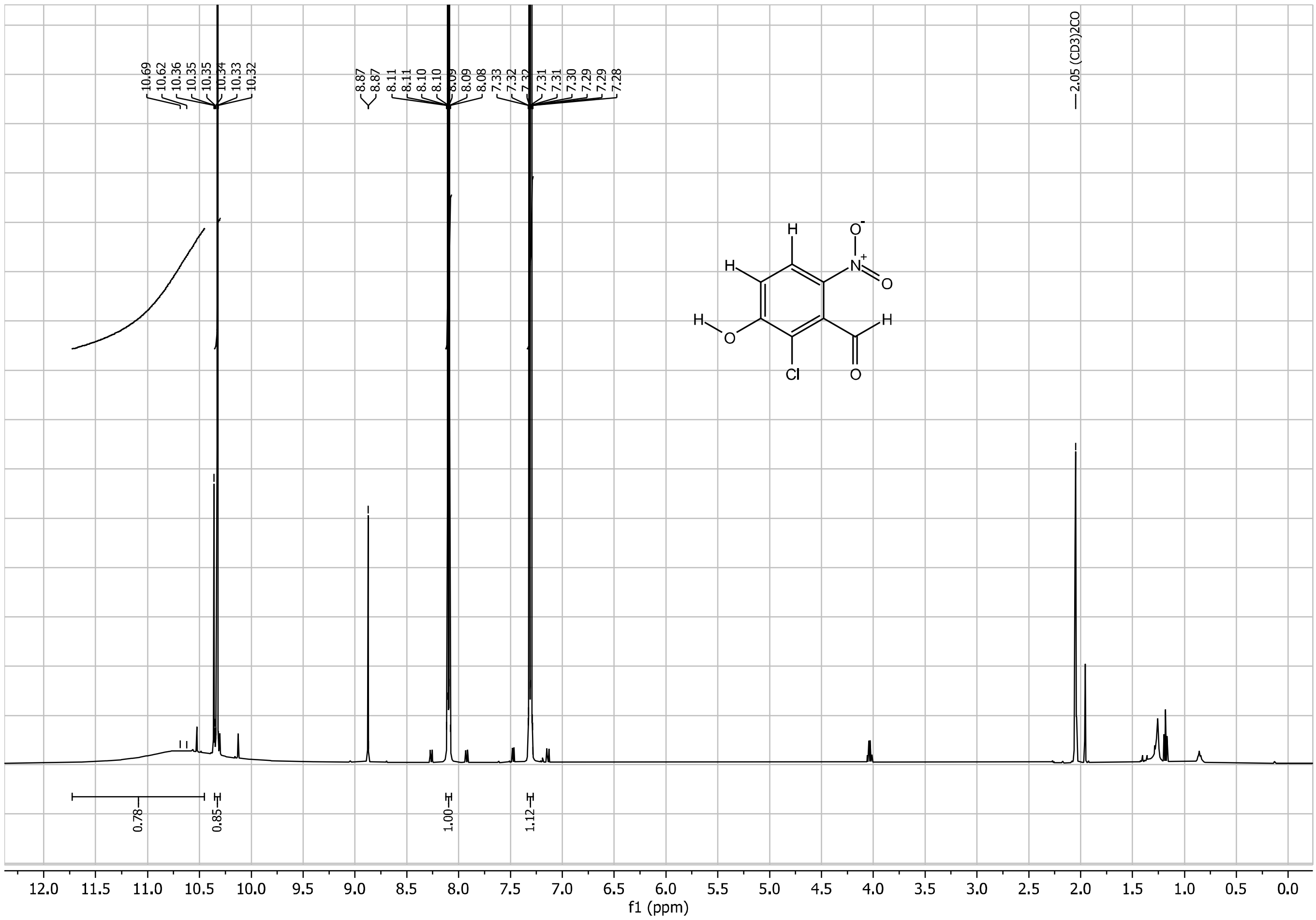
10.69
10.62
10.36
10.35
10.35
10.34
10.33
10.32
8.87
8.87
8.11
8.11
8.10
8.10
8.09
8.09
8.08
7.33
7.32
7.32
7.31
7.31
7.30
7.29
7.29
7.28
2.05 (CD3)2CO
0.78
0.85
1.00
1.12
Cl
f1 (ppm)

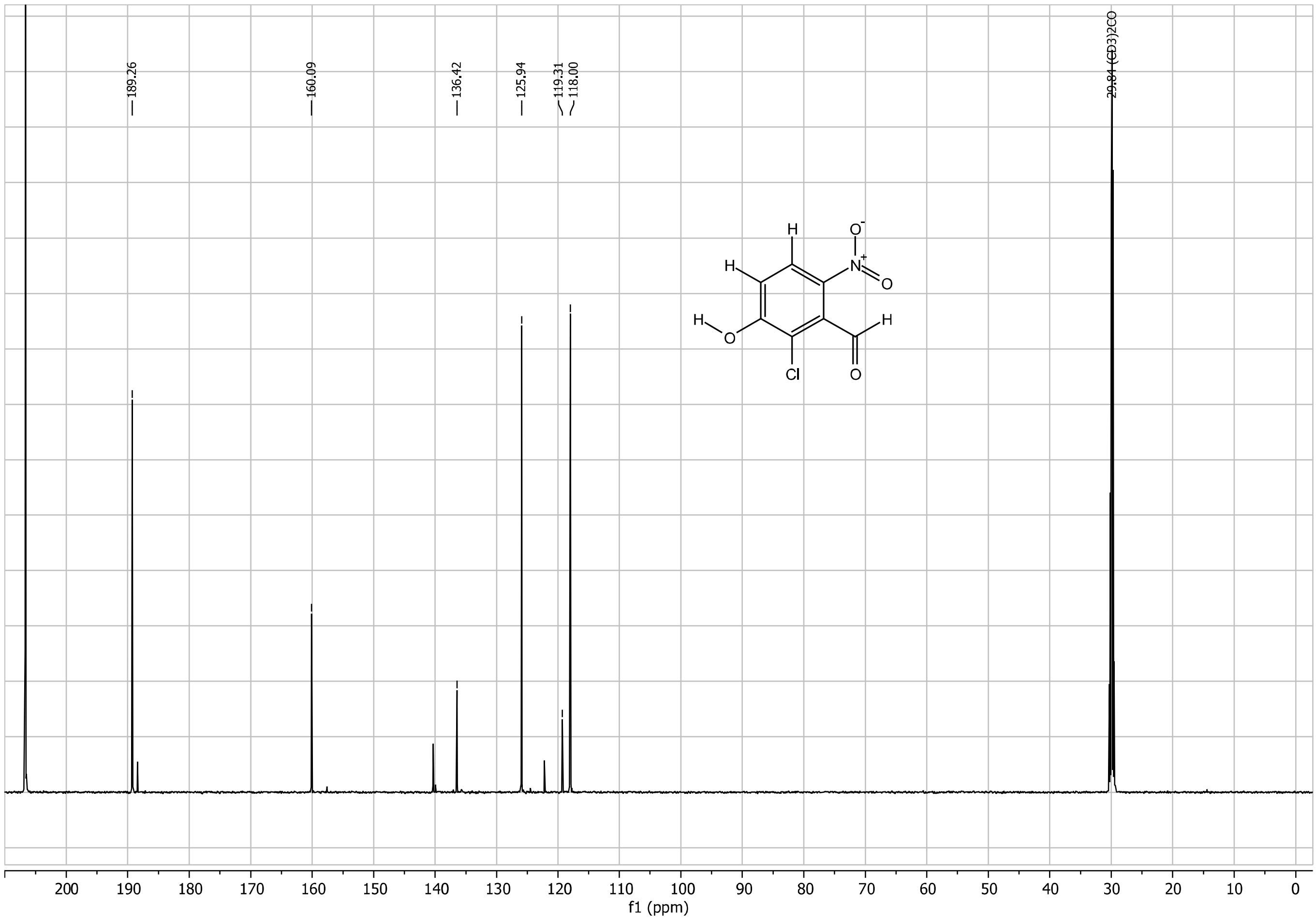

189.26
160.09
136.42
125.94
119.31
118.00
29.84 (CD3)2CO
H
O-
N+
O
H
H
O
H
Cl
O
200
190
180
170
160
150
140
130
120
110
100
90
80
70
60
50
40
30
20
10
0
f1 (ppm)

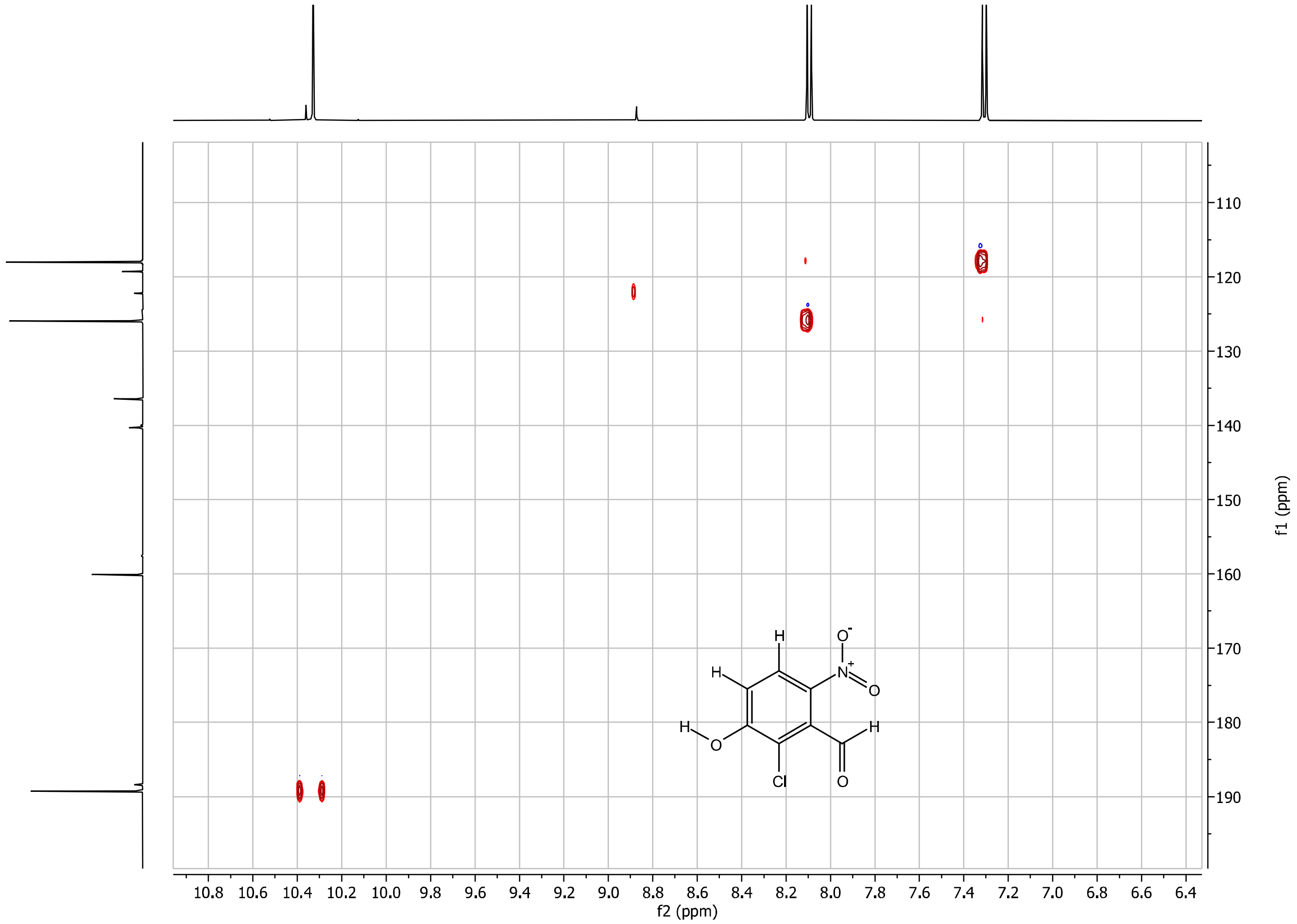

H
O-
N+
O
H
H
O
H
Cl
O
110
120
130
140
150
160
170
180
190
f1 (ppm)
10.8
10.6
10.4
10.2
10.0
9.8
9.6
9.4
9.2
9.0
8.8
8.6
8.4
8.2
8.0
7.8
7.6
7.4
7.2
7.0
6.8
6.6
6.4
f2 (ppm)

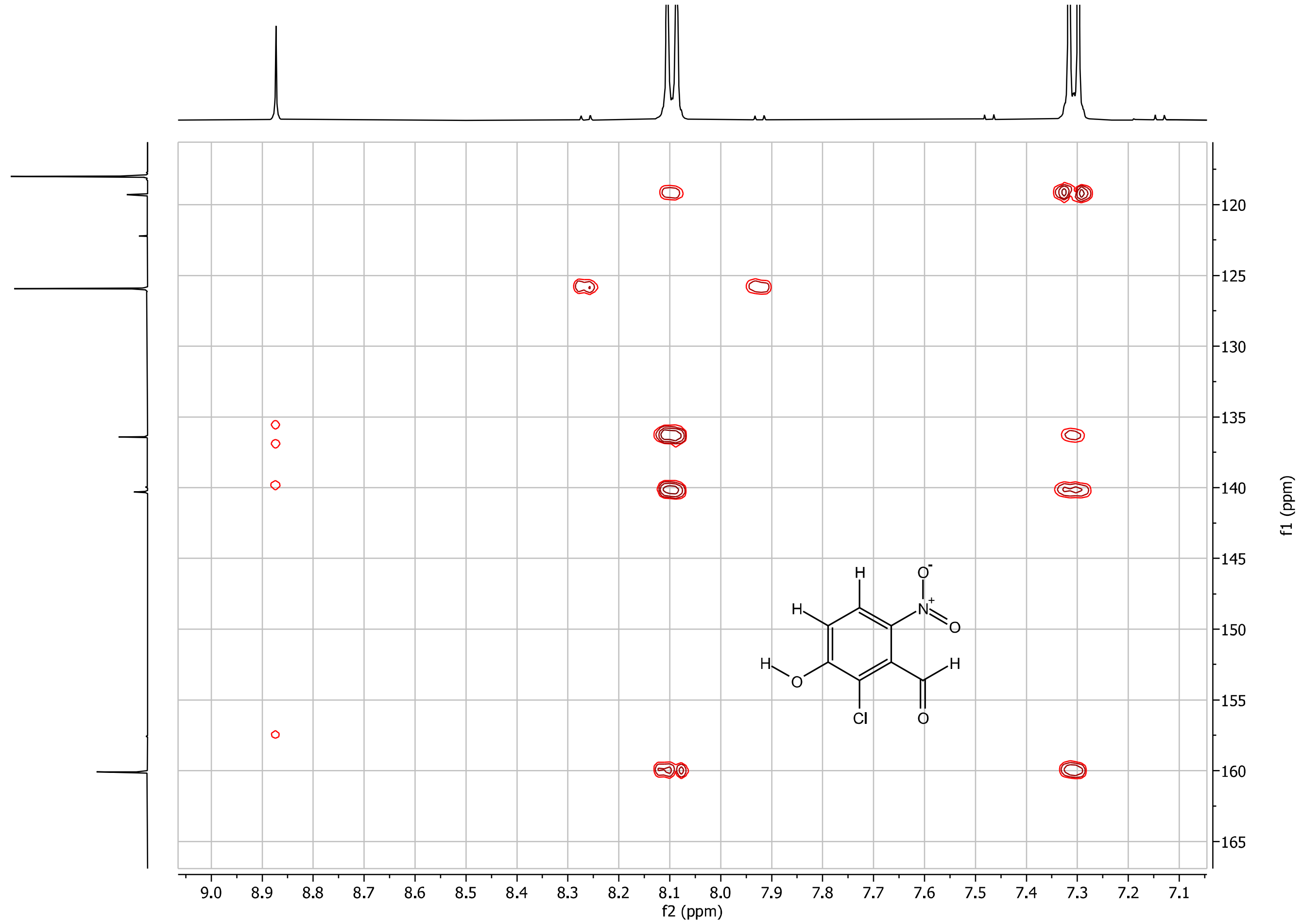
H
O-
N+
O
H
H
O
H
Cl
O
f1 (ppm)
f2 (ppm)
120
125
130
135
140
145
150
155
160
165
9.0
8.9
8.8
8.7
8.6
8.5
8.4
8.3
8.2
8.1
8.0
7.9
7.8
7.7
7.6
7.5
7.4
7.3
7.2
7.1

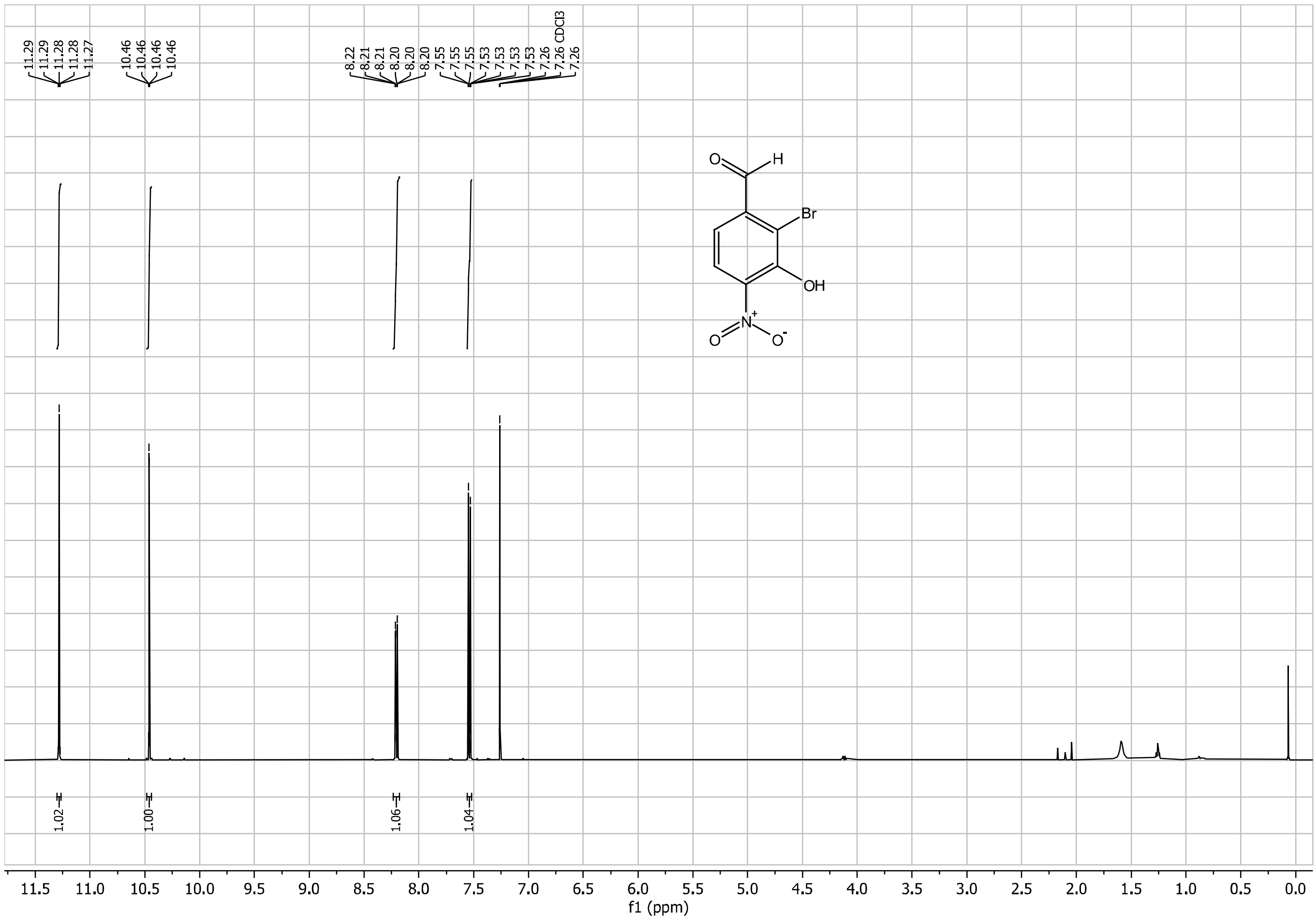
11.29
11.29
11.28
11.28
11.27
10.46
10.46
10.46
10.46
8.22
8.21
8.21
8.20
8.20
8.20
7.55
7.55
7.55
7.53
7.53
7.53
7.53
7.26
7.26 CDCl3
7.26
O
H
Br
OH
O
N+
O-
1.02
1.00
1.06
1.04
f1 (ppm)
11.5
11.0
10.5
10.0
9.5
9.0
8.5
8.0
7.5
7.0
6.5
6.0
5.5
5.0
4.5
4.0
3.5
3.0
2.5
2.0
1.5
1.0
0.5
0.0

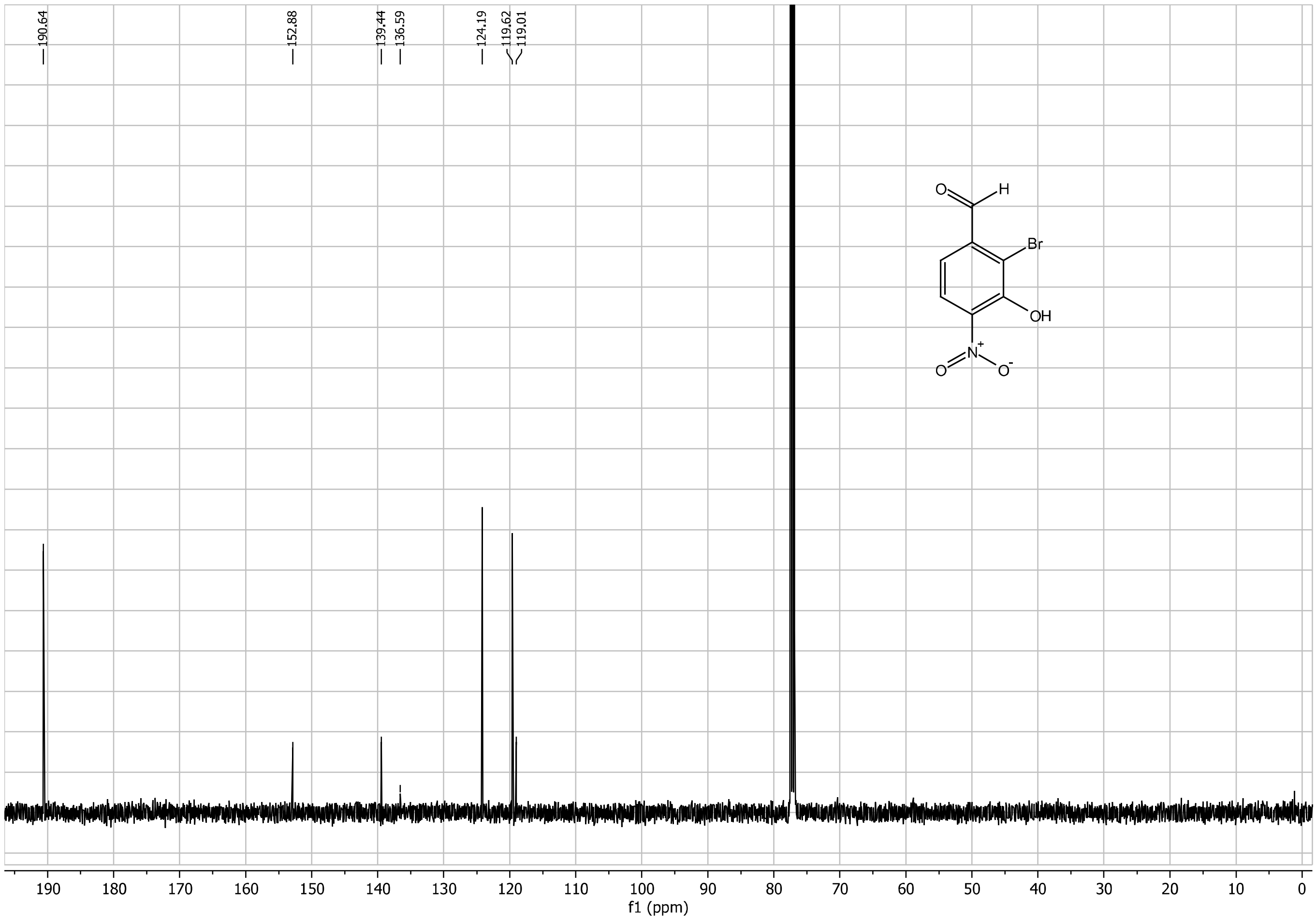
190.64
152.88
139.44
136.59
124.19
119.62
119.01
f1 (ppm)

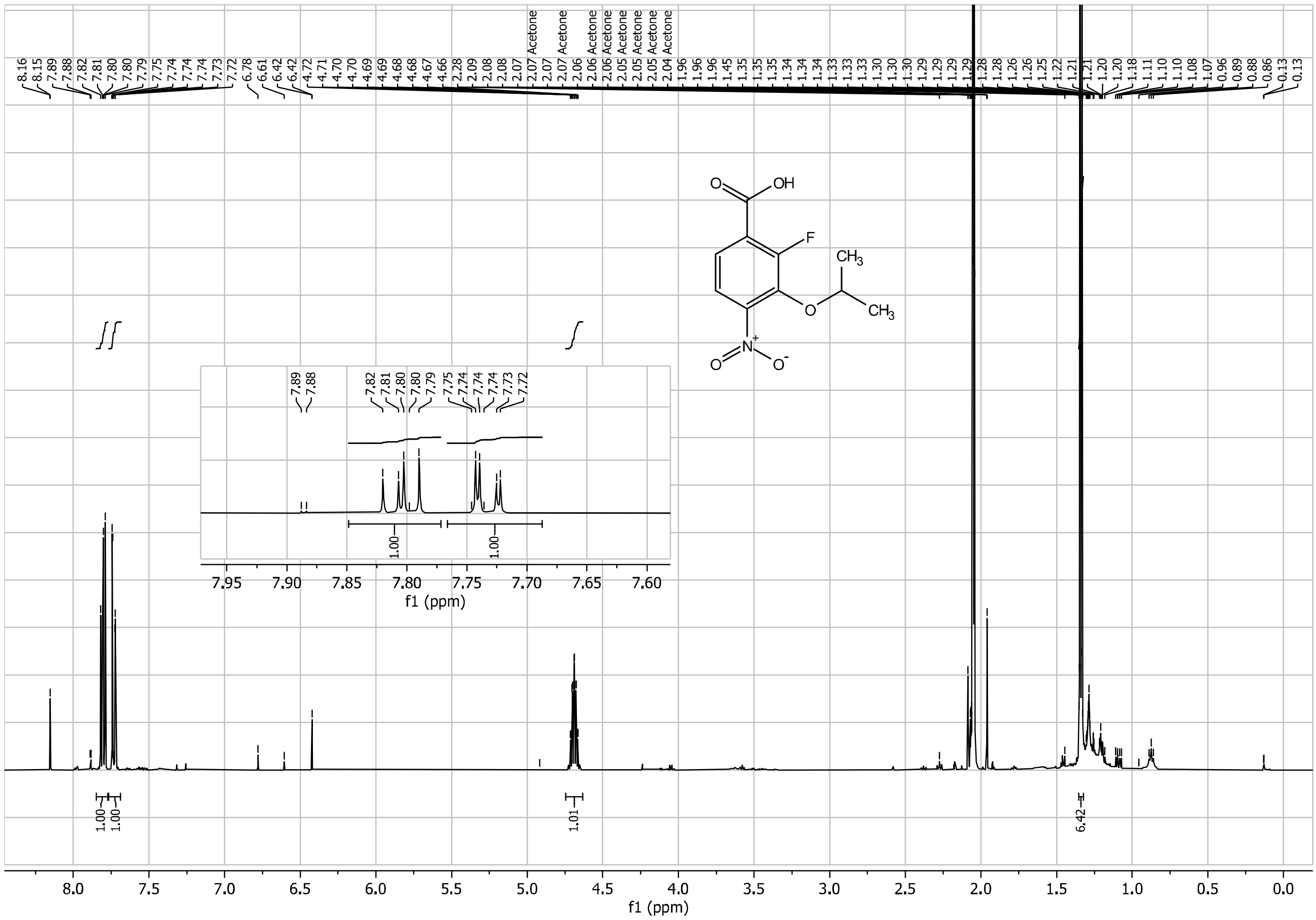
8.16
8.15
7.89
7.88
7.82
7.81
7.80
7.80
7.79
7.75
7.74
7.74
7.74
7.73
7.72
6.78
6.61
6.42
6.42
4.72
4.71
4.70
4.70
4.69
4.69
4.68
4.68
4.67
4.66
2.28
2.09
2.08
2.08
2.07
2.07 Acetone
2.07 Acetone
2.06 Acetone
2.06 Acetone
2.05 Acetone
2.05 Acetone
2.05 Acetone
2.04 Acetone
1.96
1.96
1.96
1.45
1.35
1.35
1.34
1.34
1.33
1.33
1.33
1.30
1.30
1.30
1.29
1.29
1.29
1.29
1.28
1.28
1.26
1.26
1.25
1.22
1.21
1.21
1.20
1.20
1.18
1.11
1.10
1.10
1.08
1.07
0.96
0.89
0.88
0.86
0.13
0.13
1.00
1.00
1.01
6.42
f1 (ppm)

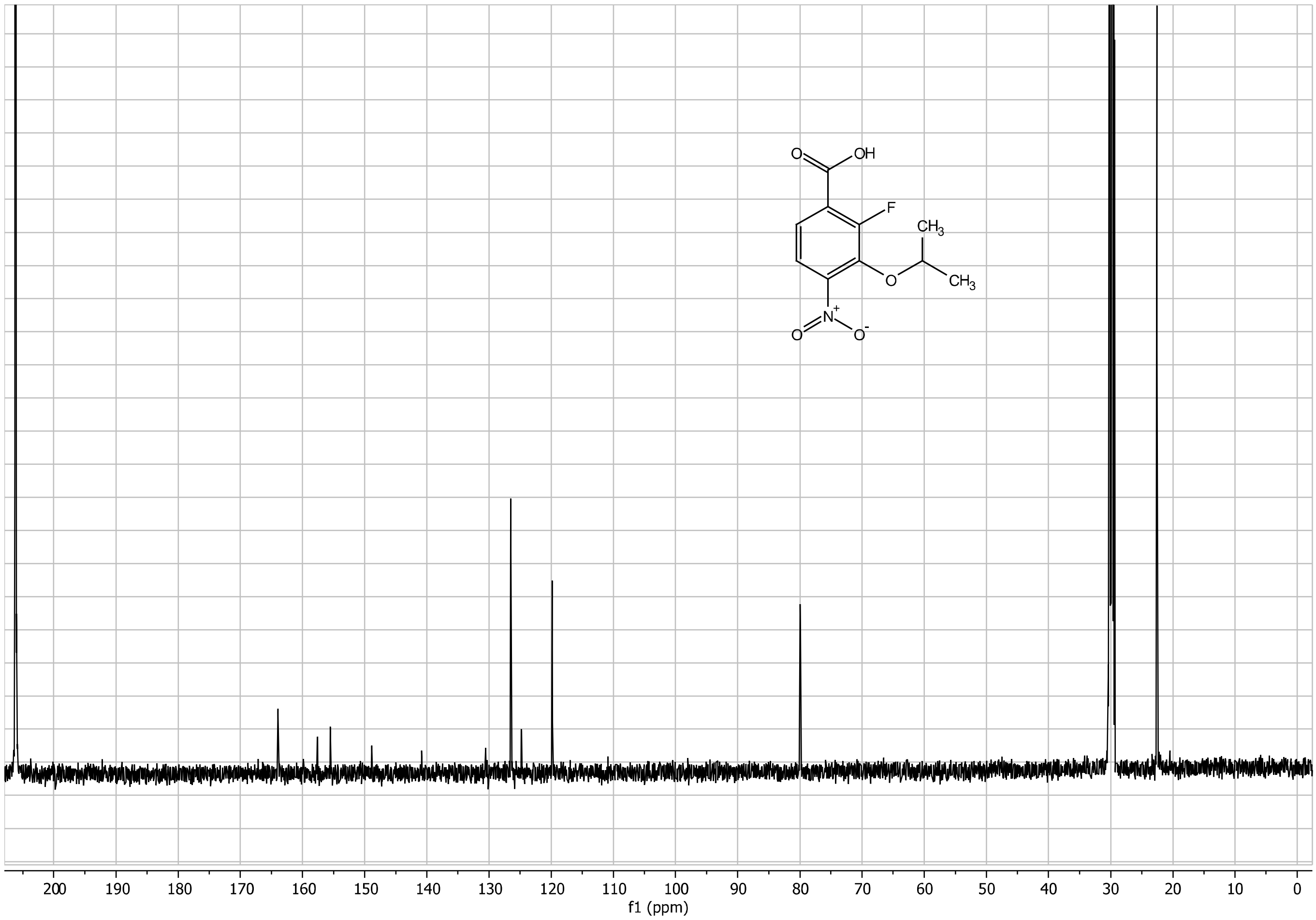
O
OH
F
CH3
O
CH3
N+
O
O-
200 190 180 170 160 150 140 130 120 110 100 90 80 70 60 50 40 30 20 10 0
f1 (ppm)

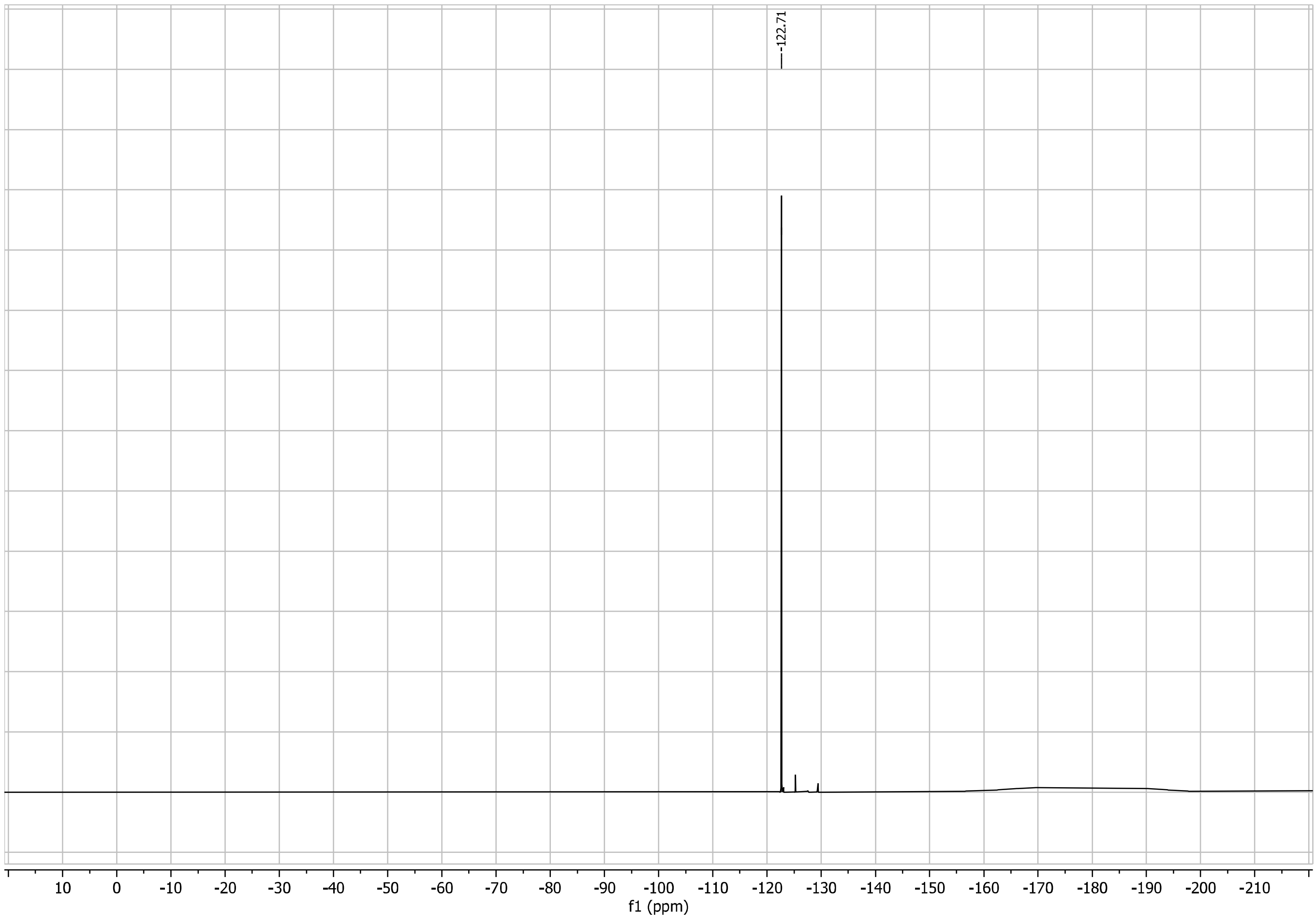
-122.71
10 0 -10 -20 -30 -40 -50 -60 -70 -80 -90 -100 -110 -120 -130 -140 -150 -160 -170 -180 -190 -200 -210
f1 (ppm)

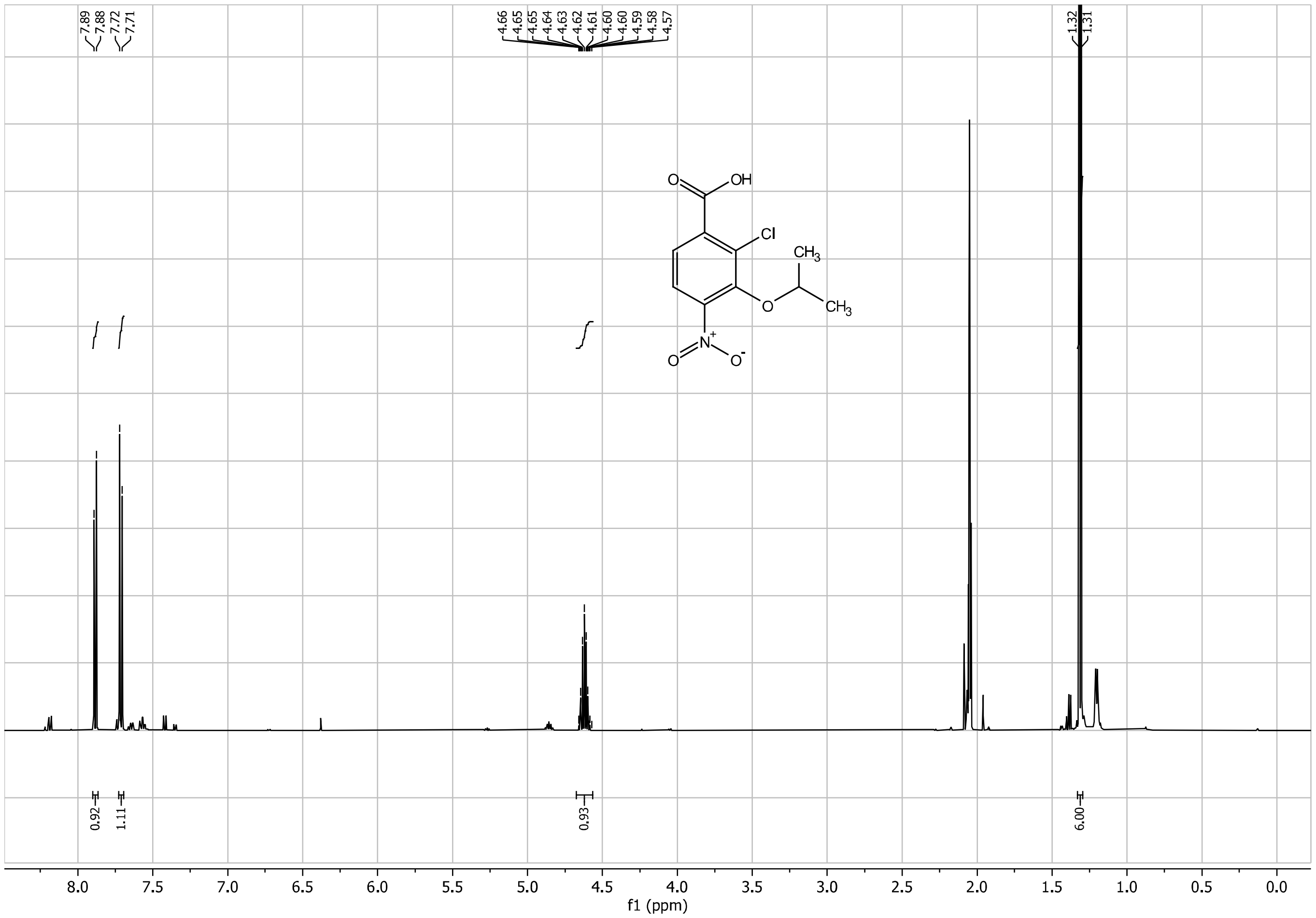

7.89
7.88
7.72
7.71
4.66
4.65
4.65
4.64
4.63
4.62
4.61
4.60
4.60
4.59
4.58
4.57
1.32
1.31
O
OH
Cl
CH3
O
CH3
N+
O
O-
0.92
1.11
0.93
6.00
8.0 7.5 7.0 6.5 6.0 5.5 5.0 4.5 4.0 3.5 3.0 2.5 2.0 1.5 1.0 0.5 0.0
f1 (ppm)

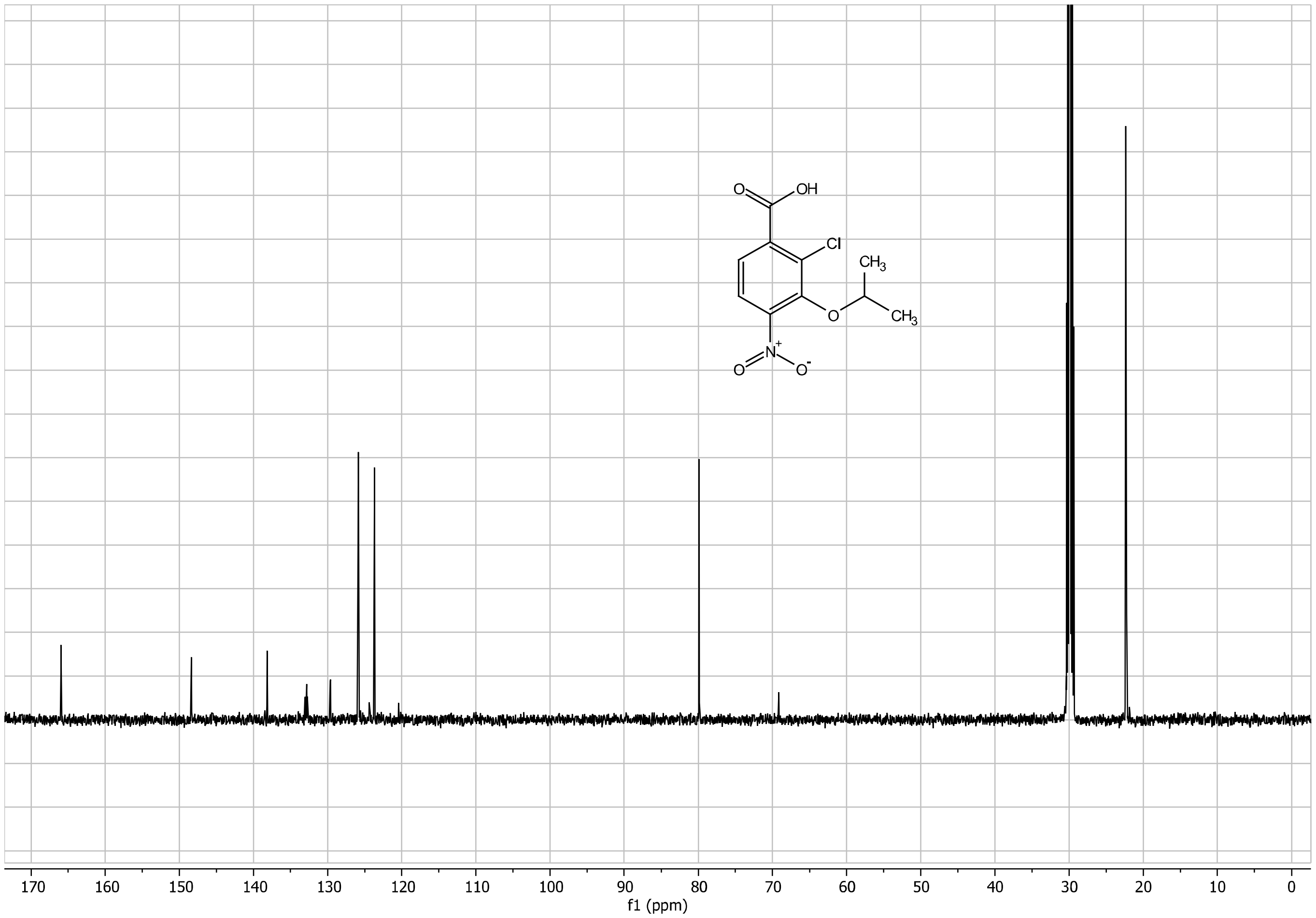

O
OH
Cl
CH3
O
CH3
N+
O
O-
170 160 150 140 130 120 110 100 90 80 70 60 50 40 30 20 10 0
f1 (ppm)

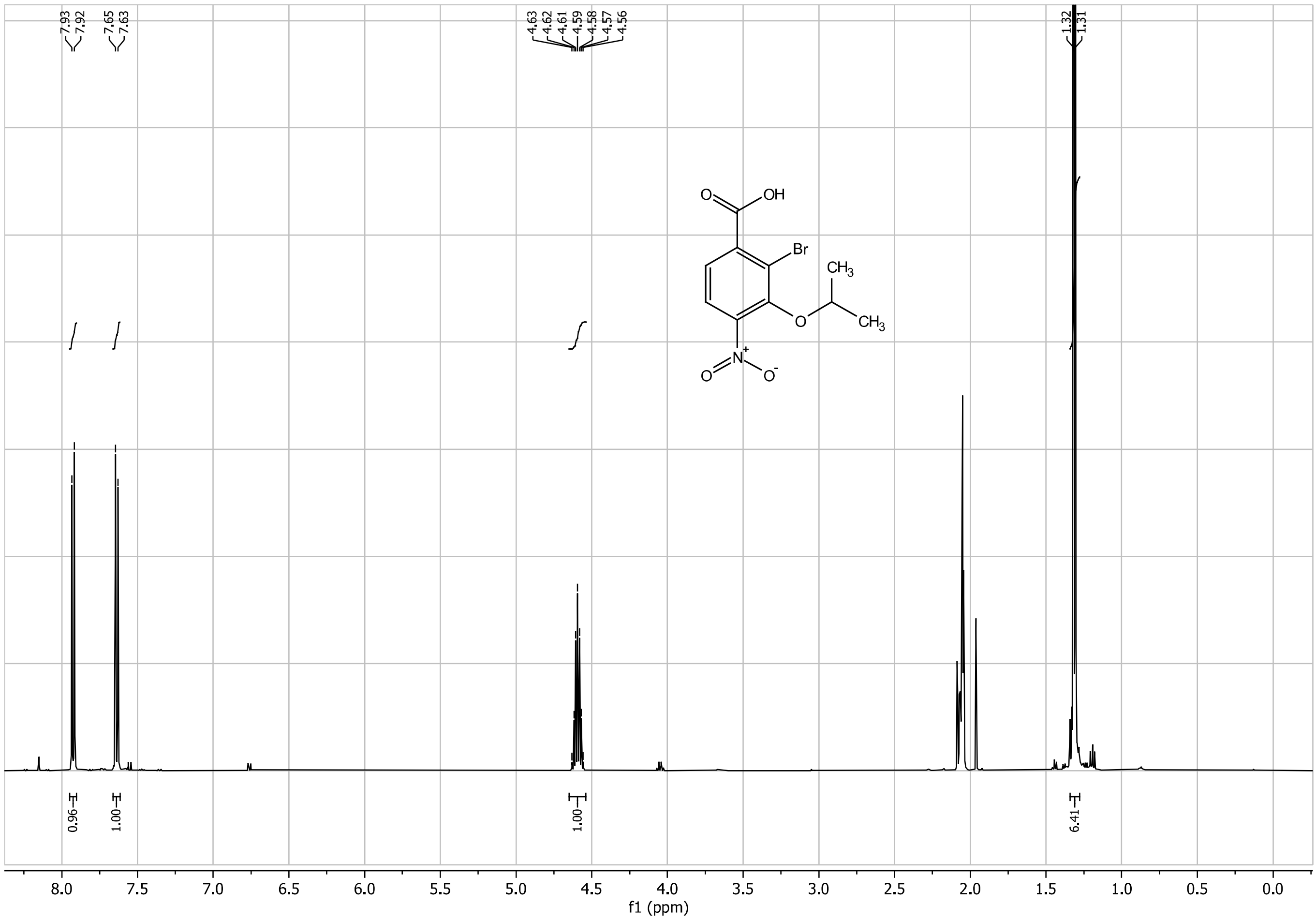
7.93
7.92
7.65
7.63
4.63
4.62
4.61
4.59
4.58
4.57
4.56
1.32
1.31
OH
Br
CH3
O
CH3
N
0.96
1.00
1.00
6.41
f1 (ppm)

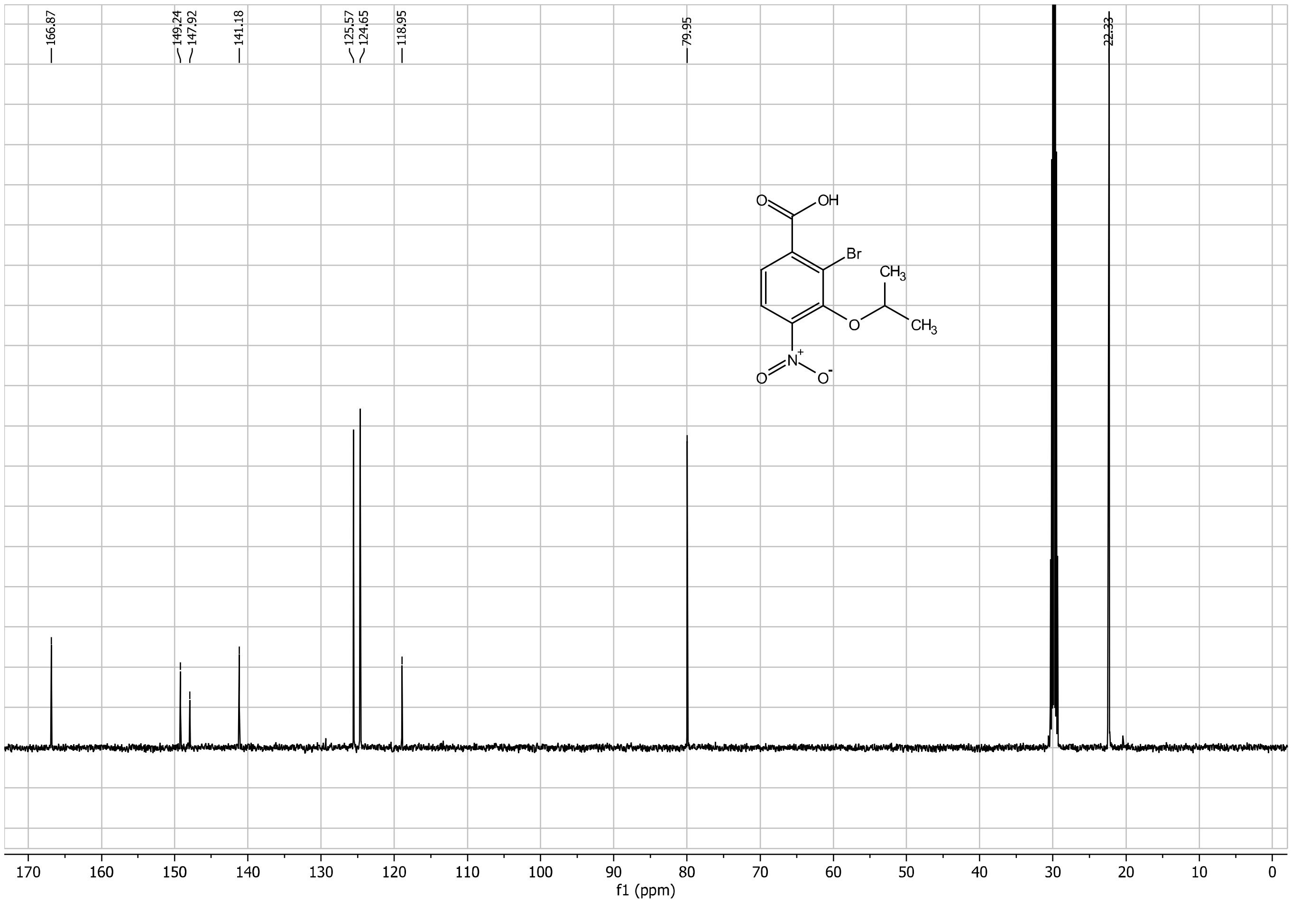
166.87
149.24
147.92
141.18
125.57
124.65
118.95
79.95
22.33
OH
Br
CH3
O
CH3
N
f1 (ppm)

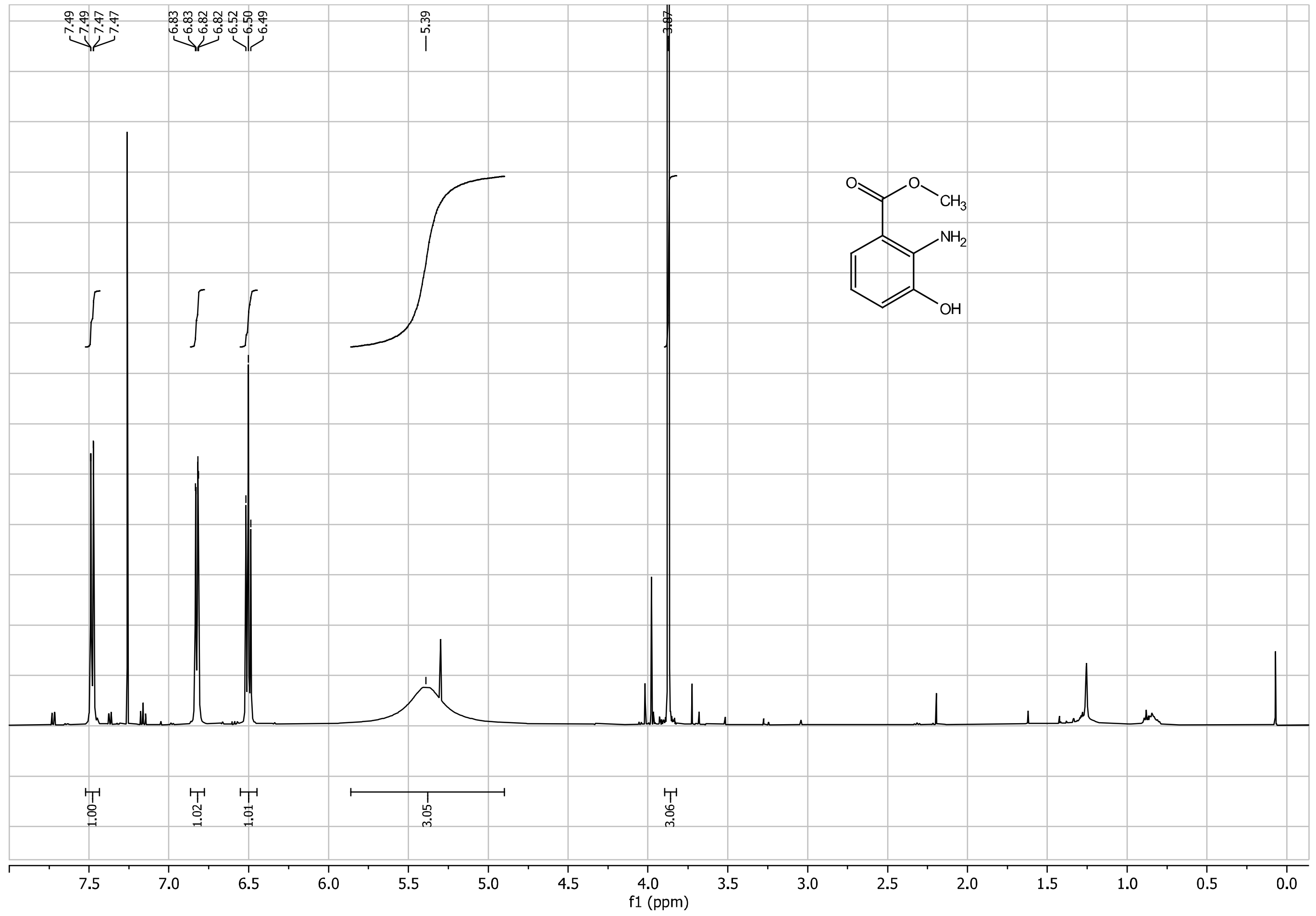
7.49
7.49
7.47
7.47
6.83
6.83
6.82
6.82
6.52
6.50
6.49
5.39
3.87
NH2
OH
CH3
1.00
1.02
1.01
3.05
3.06
f1 (ppm)

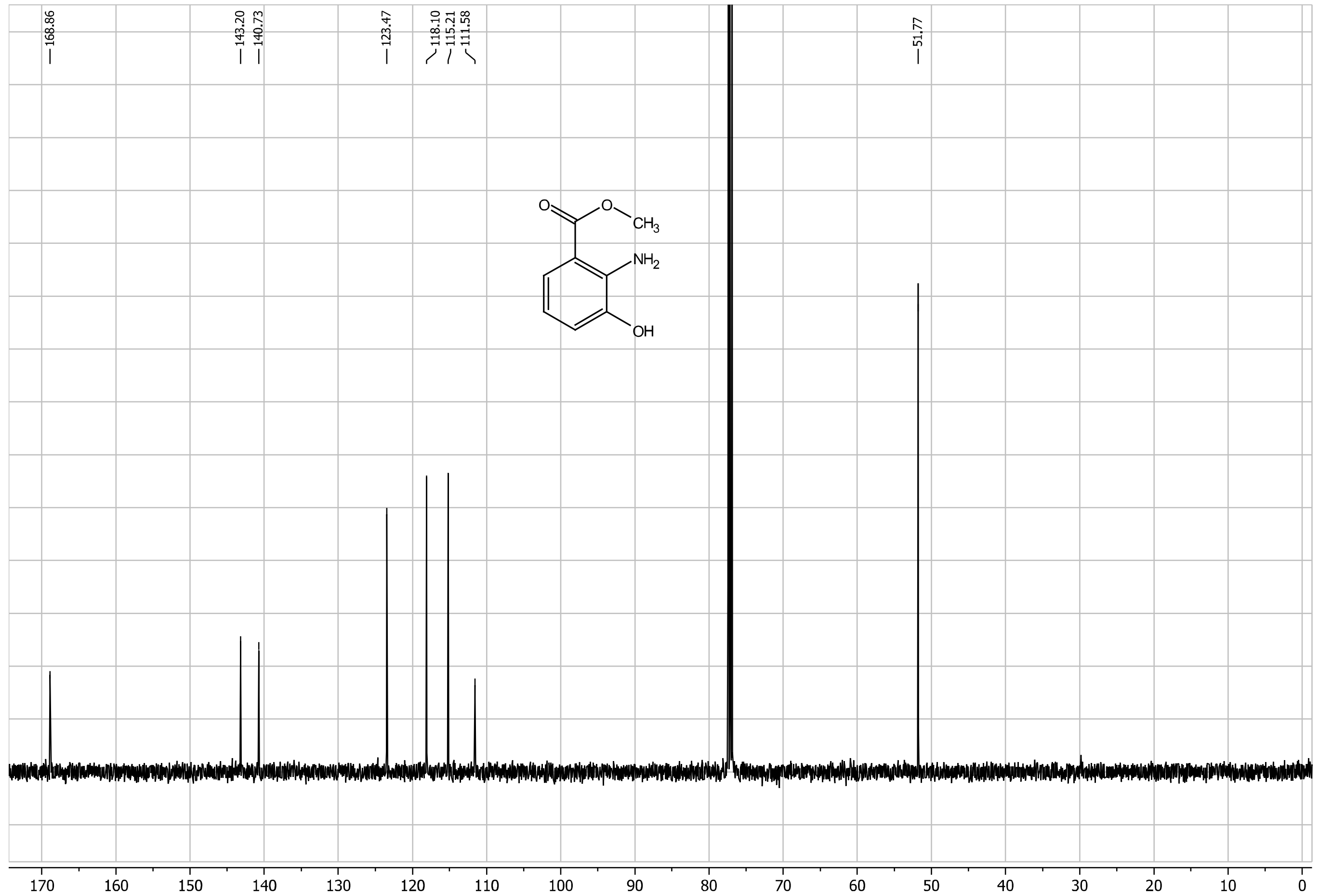
168.86
143.20
140.73
123.47
118.10
115.21
111.58
51.77
NH2
OH
CH3
f1 (ppm)

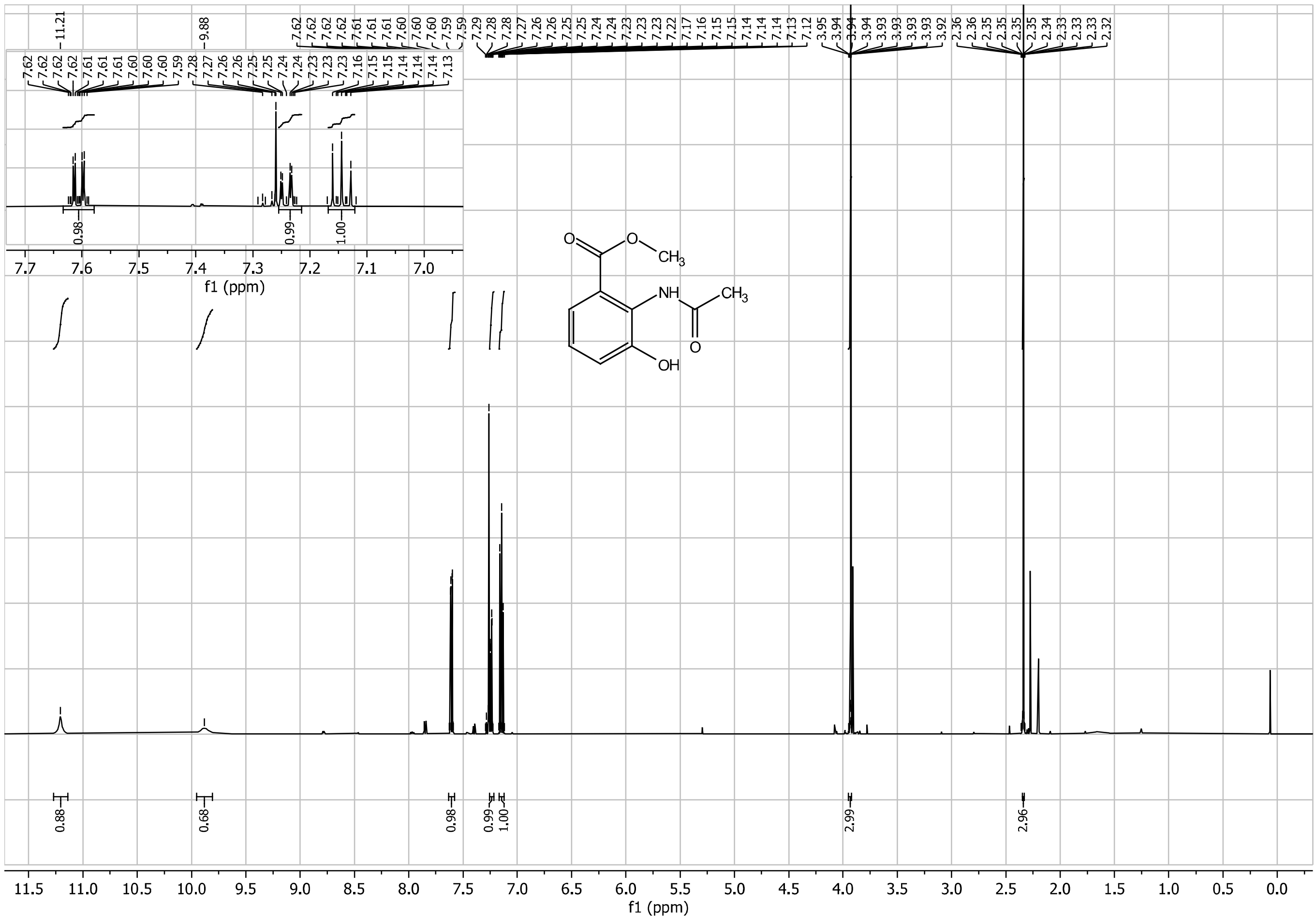

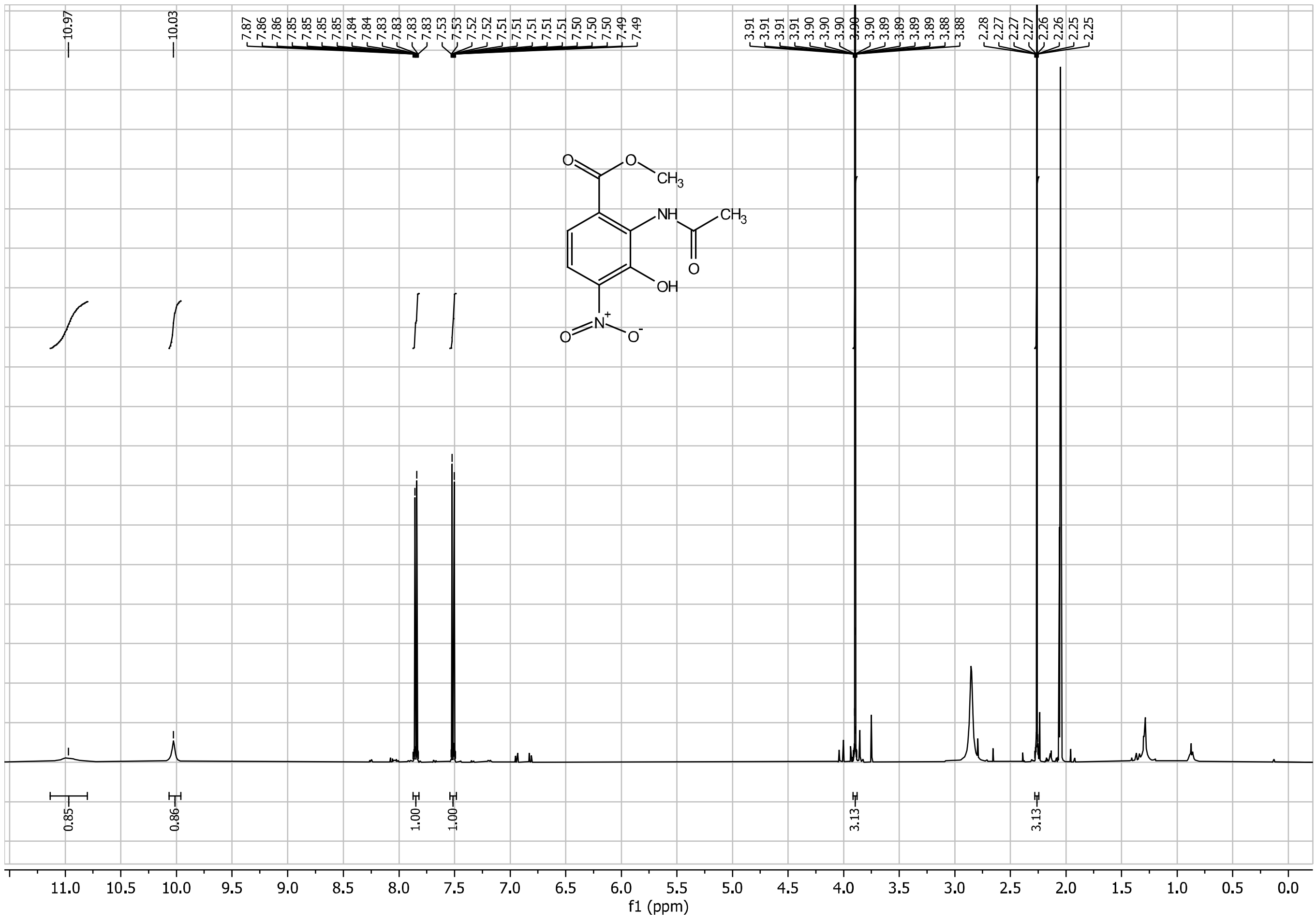

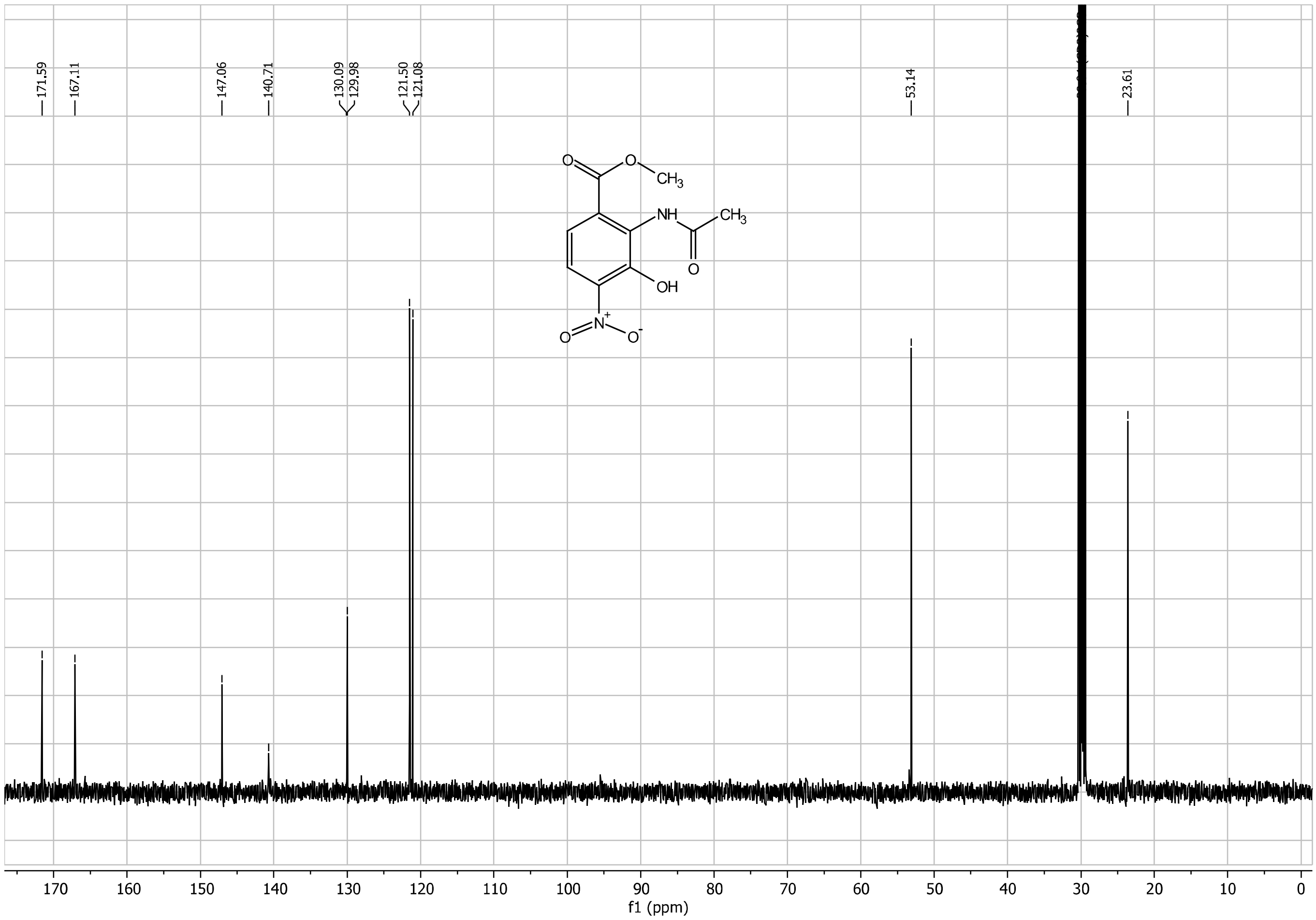
171.59
167.11
147.06
140.71
130.09
129.98
121.50
121.08
53.14
23.61
f1 (ppm)

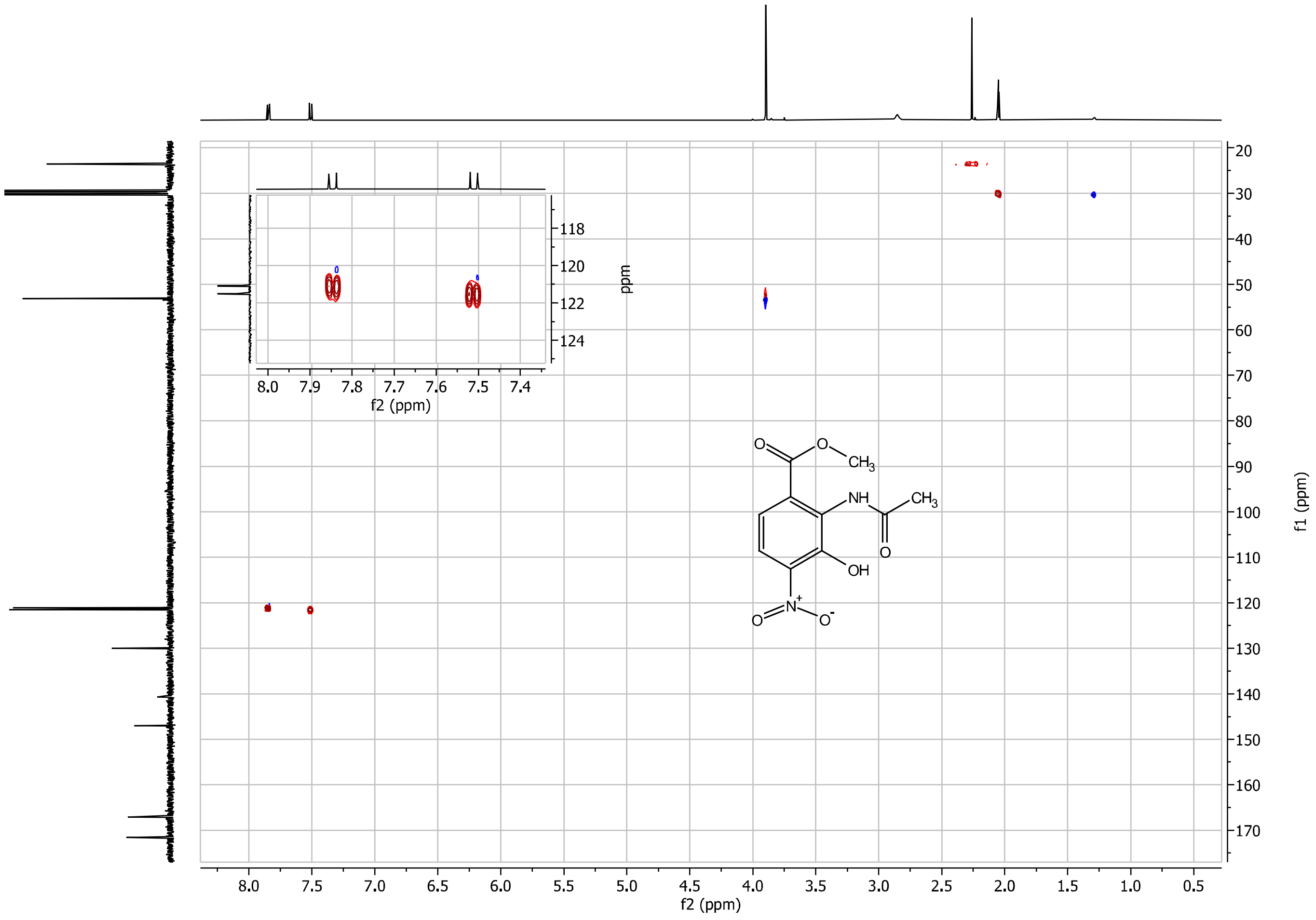
f2 (ppm)
f1 (ppm)
ppm

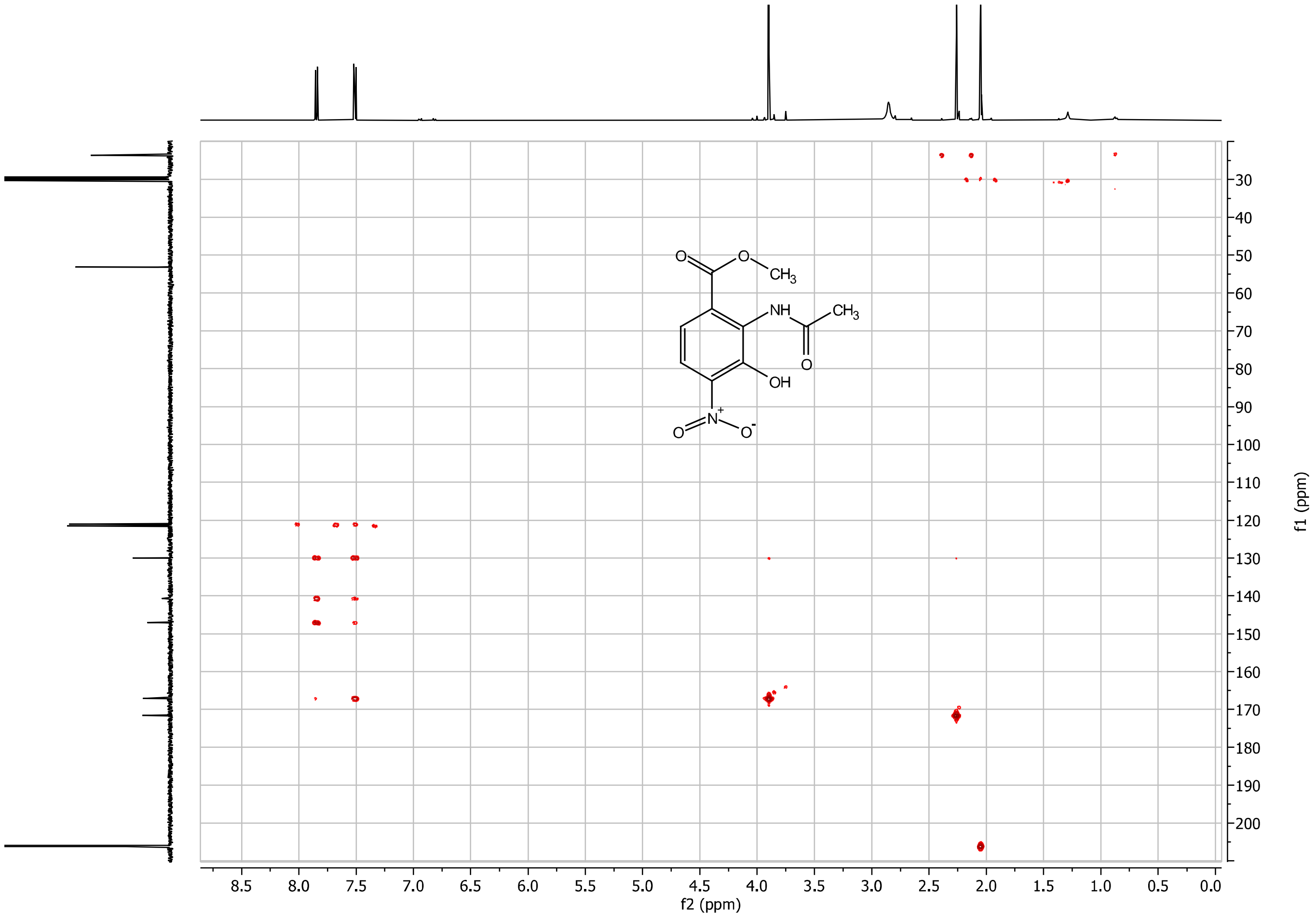

f2 (ppm)
f1 (ppm)

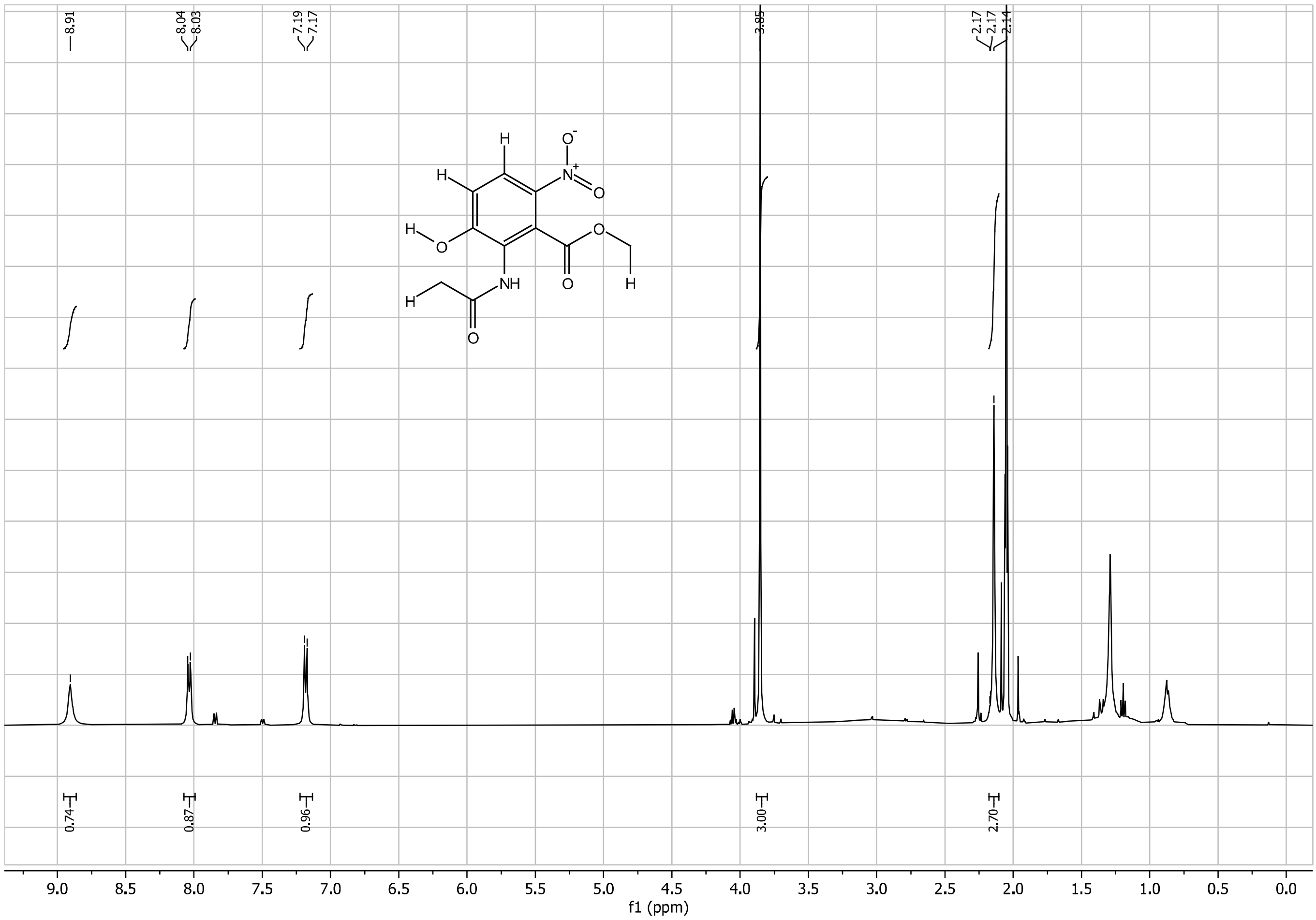

8.91
8.04
8.03
7.19
7.17
3.85
2.17
2.17
2.14
0.74
0.87
0.96
3.00
2.70
f1 (ppm)

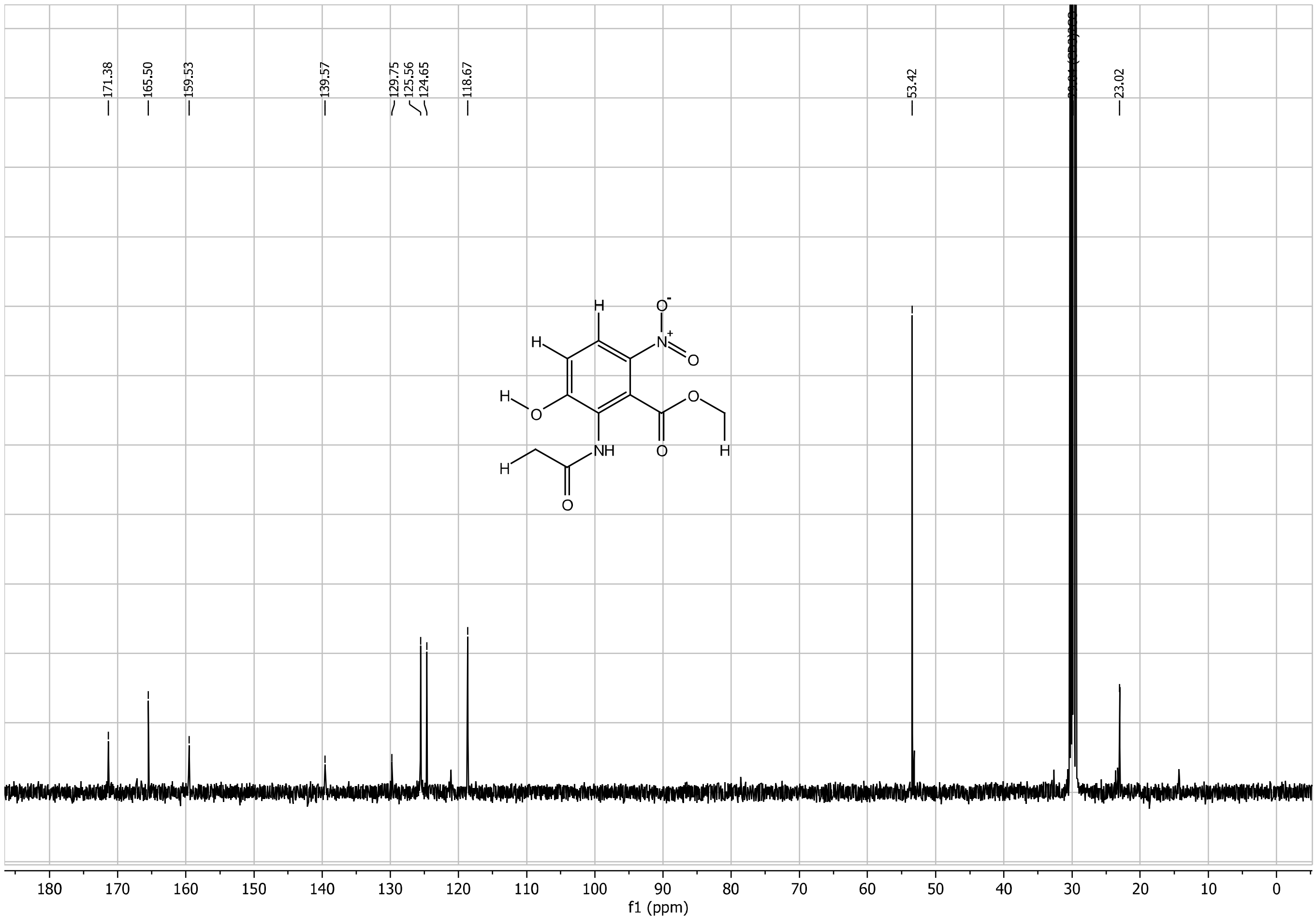
171.38
165.50
159.53
139.57
129.75
125.56
124.65
118.67
53.42
23.02
f1 (ppm)

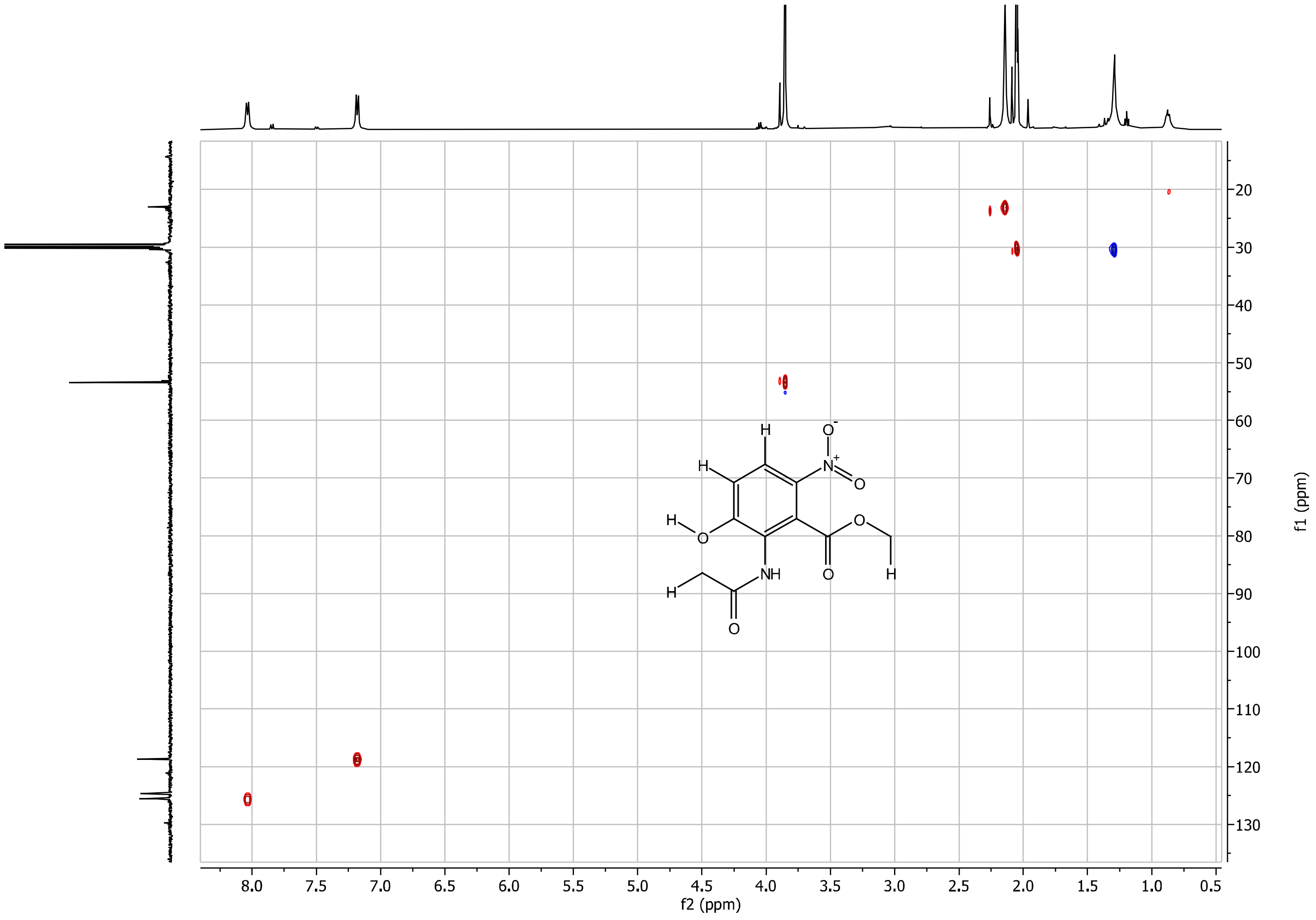
f2 (ppm)
f1 (ppm)

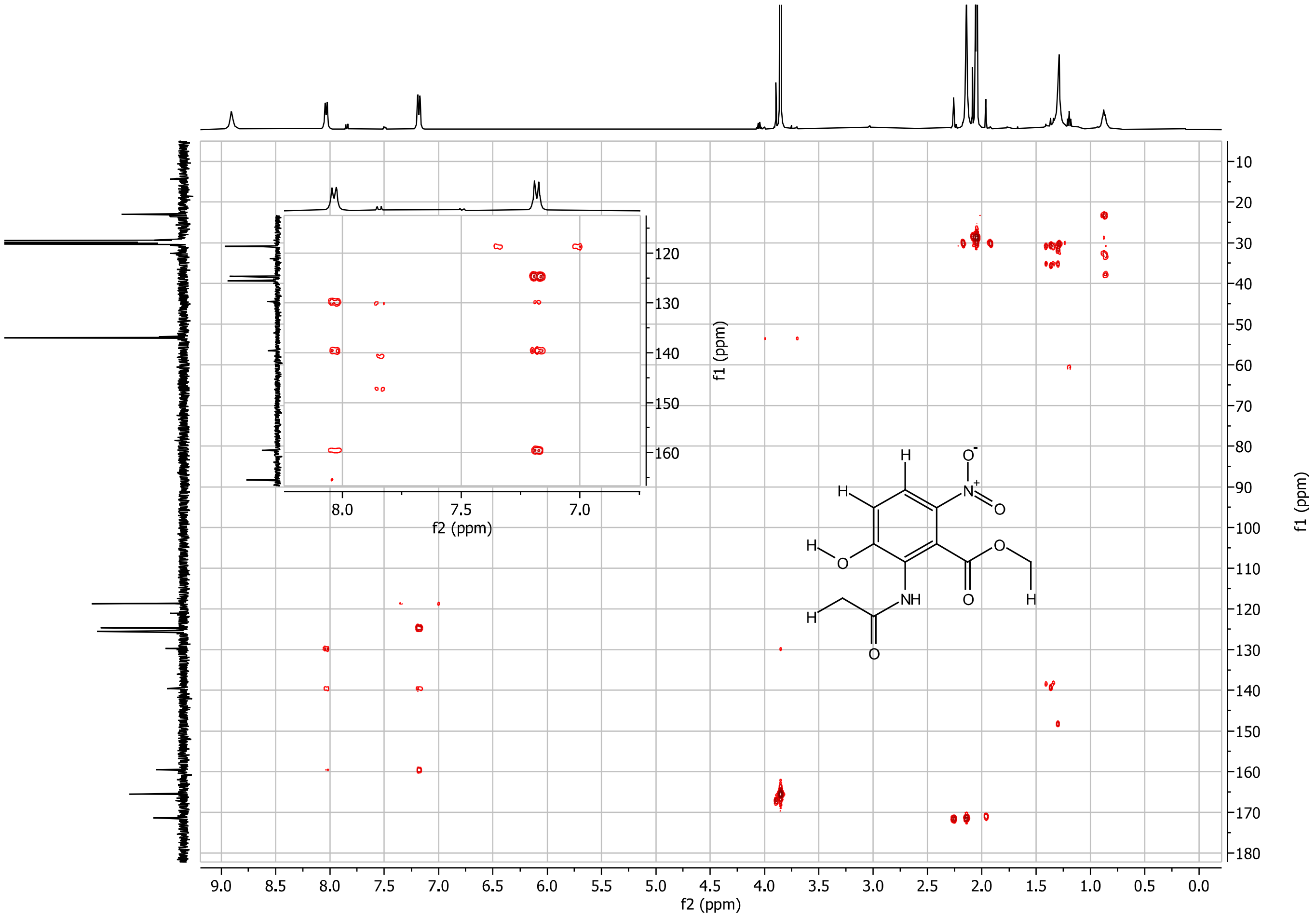
f2 (ppm)
f1 (ppm)

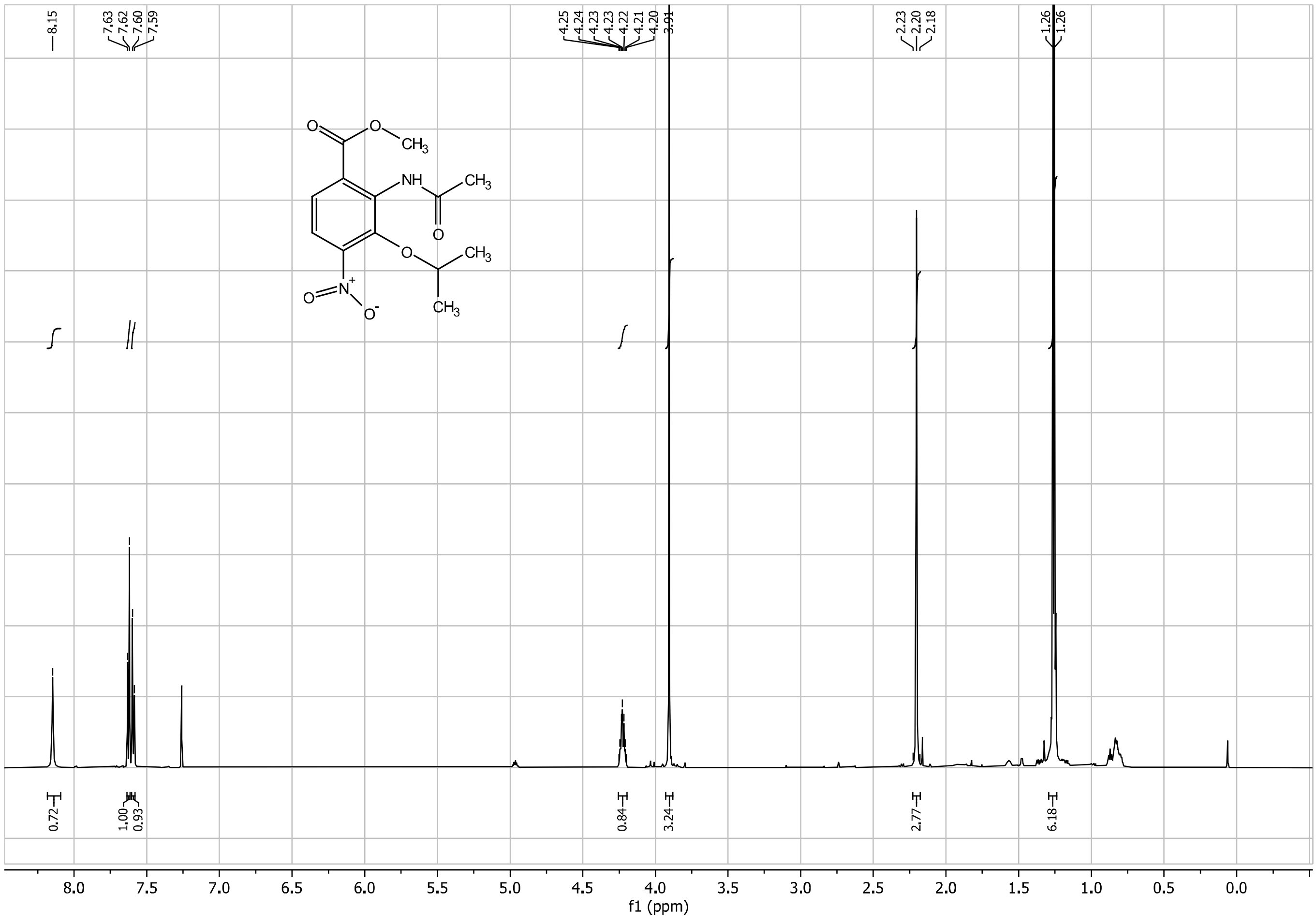
f1 (ppm)

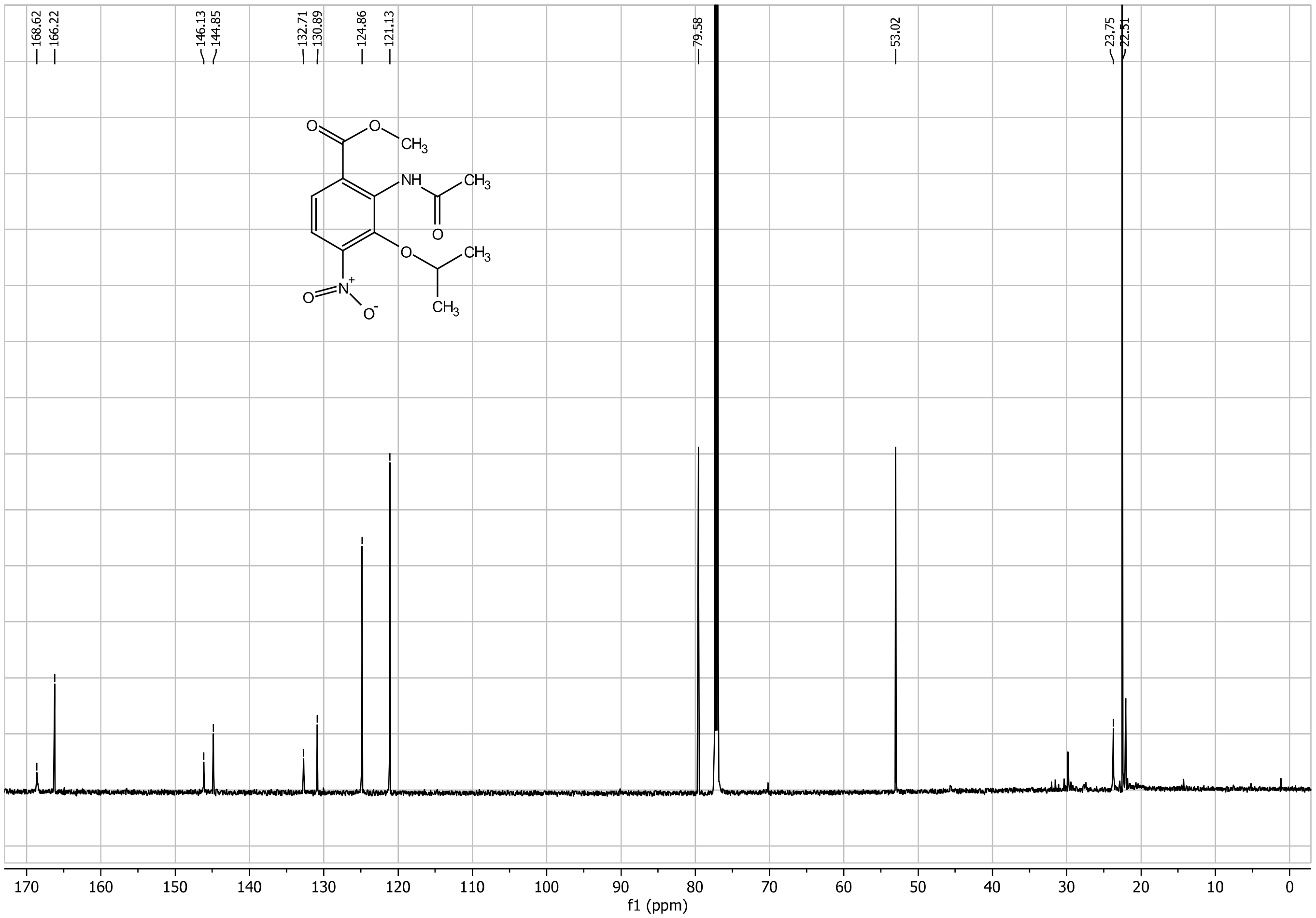
168.62
166.22
146.13
144.85
132.71
130.89
124.86
121.13
79.58
53.02
23.75
22.51
f1 (ppm)

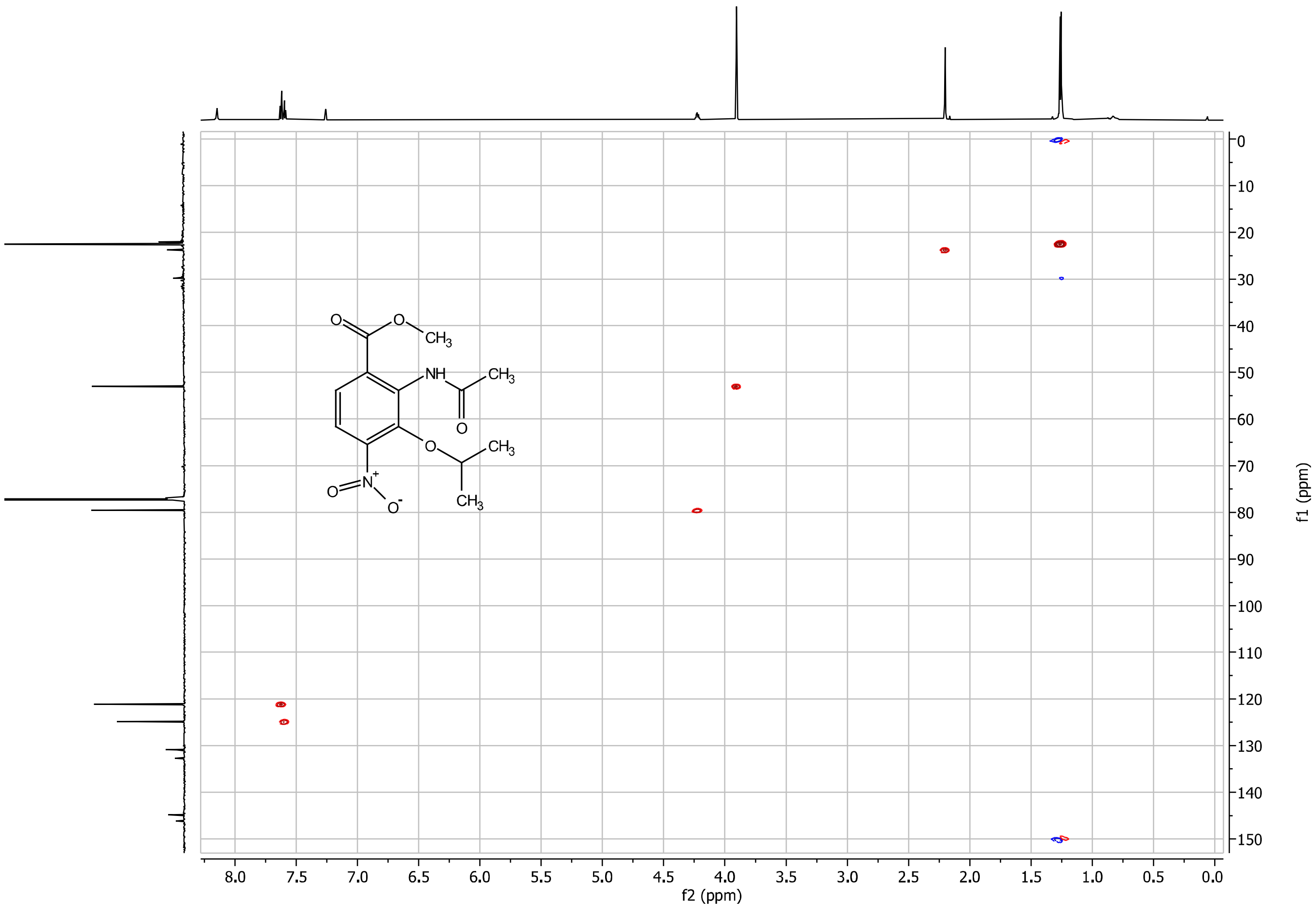
f2 (ppm)
f1 (ppm)

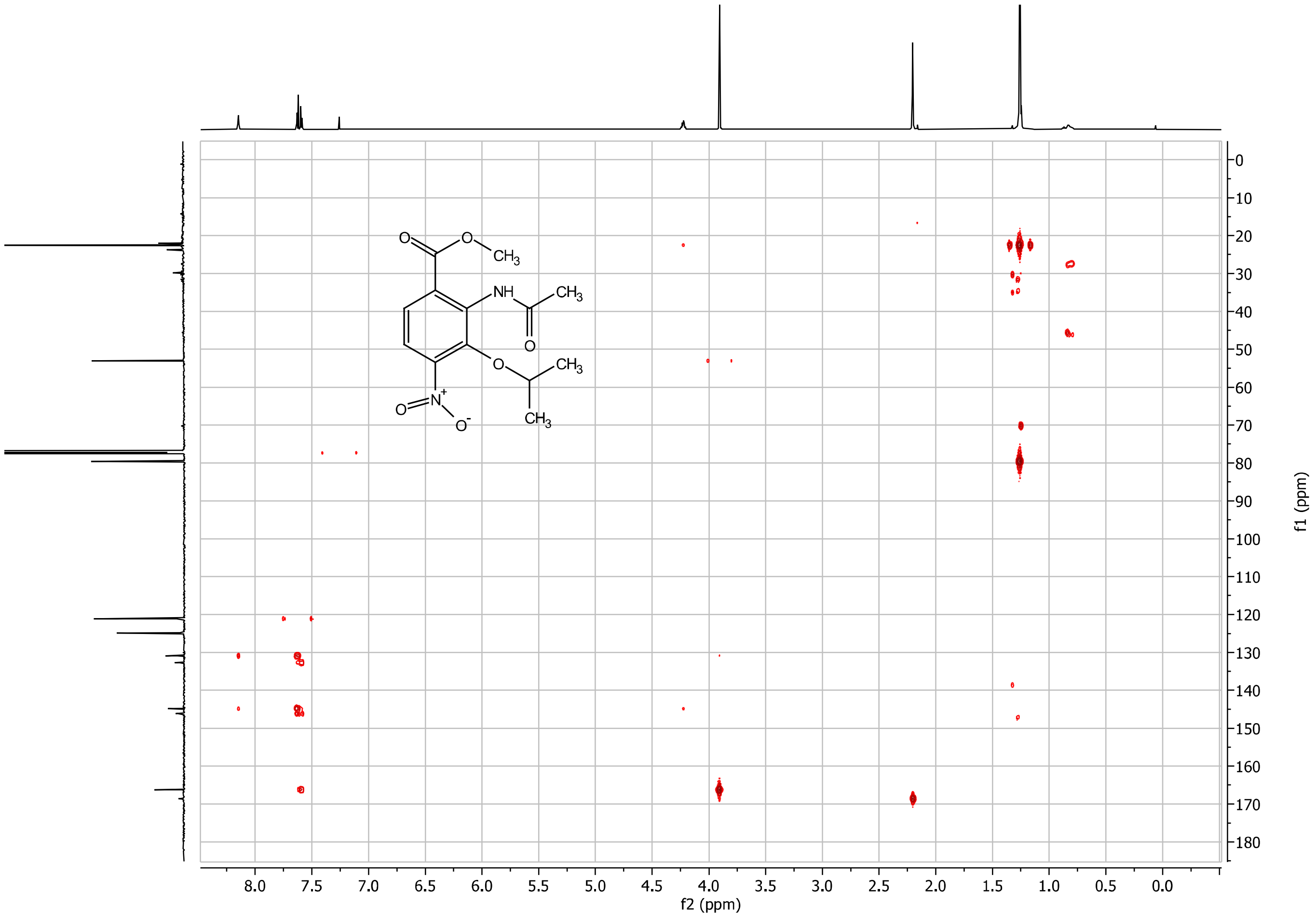

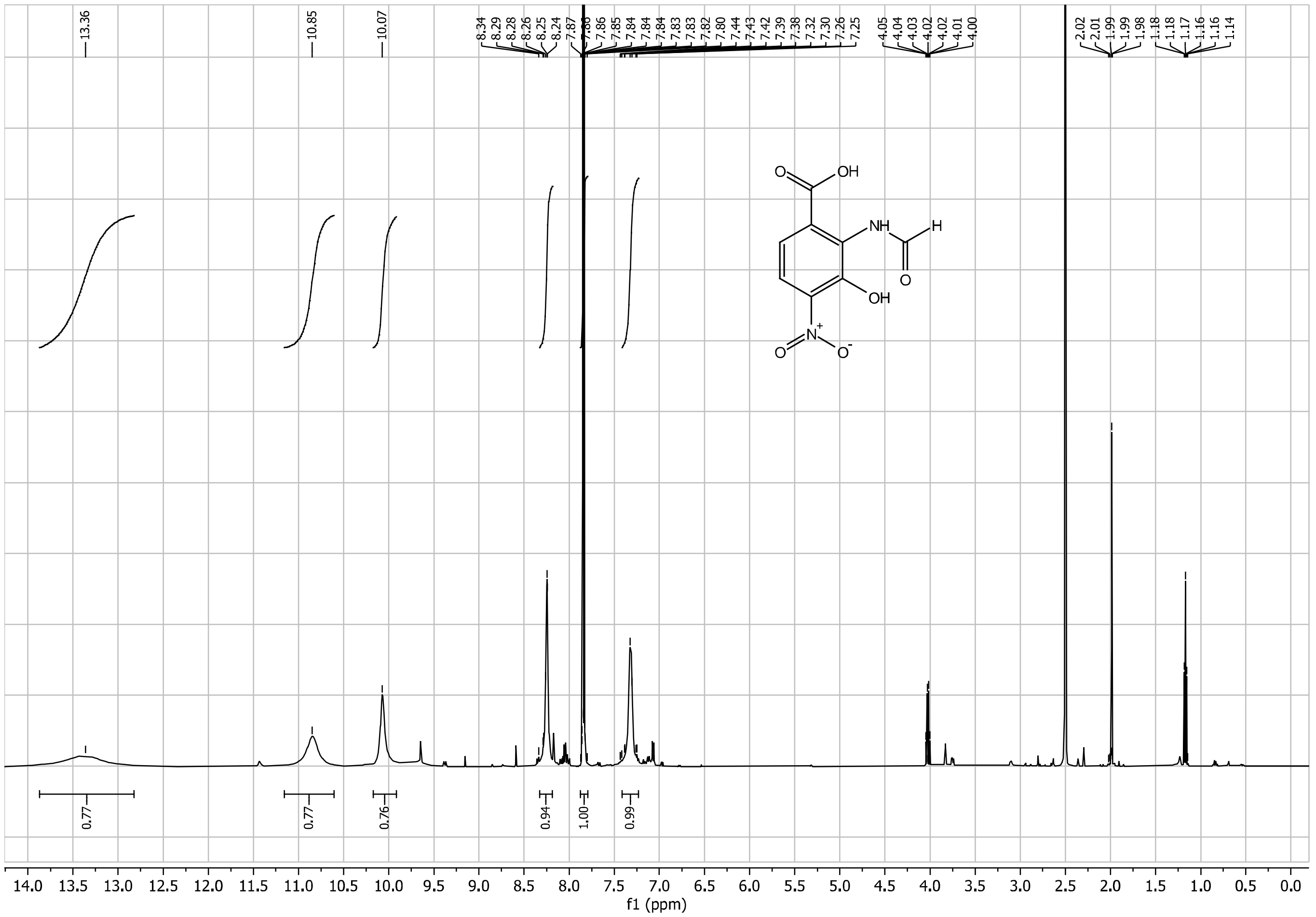

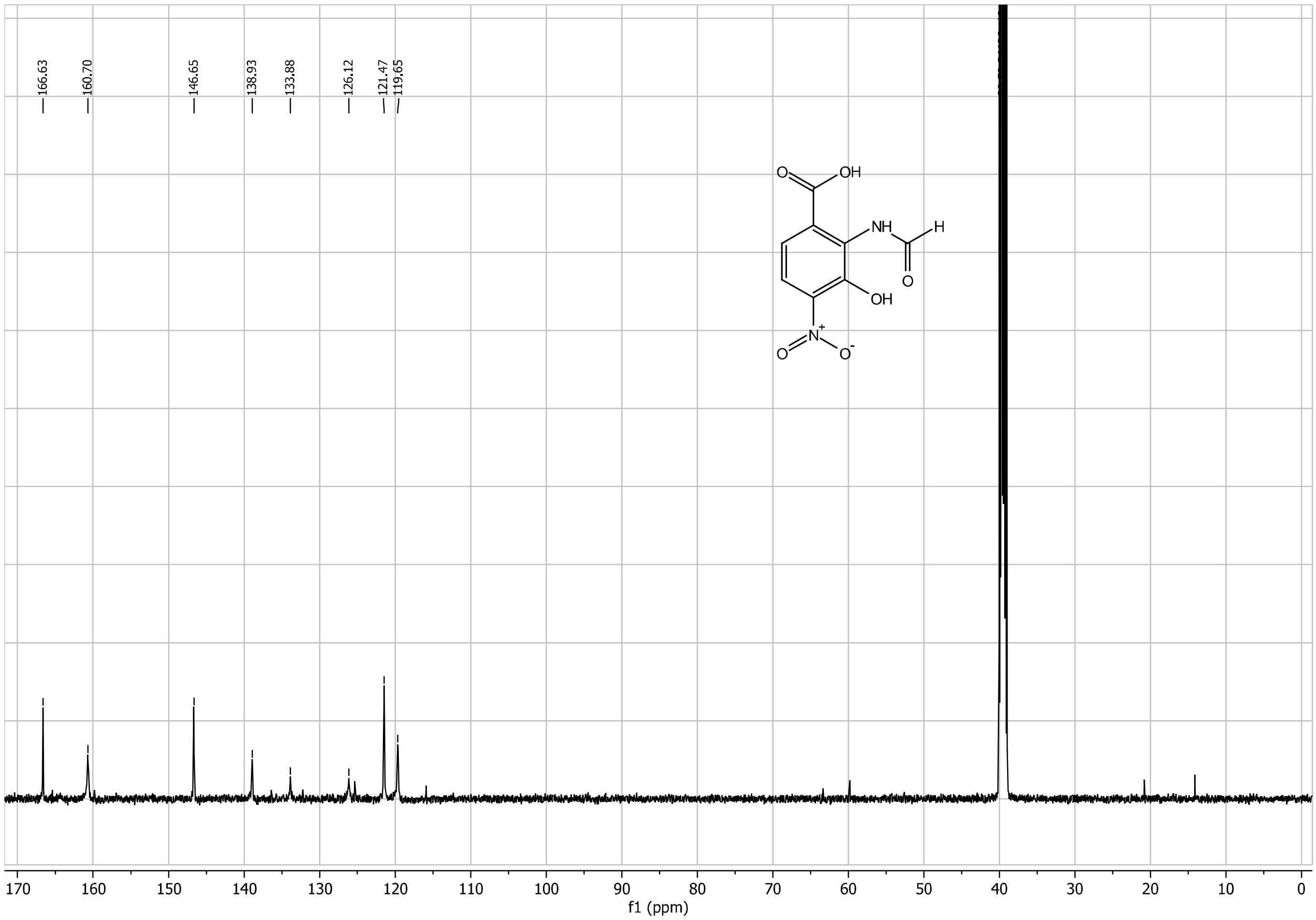
166.63
160.70
146.65
138.93
133.88
126.12
121.47
119.65
f1 (ppm)

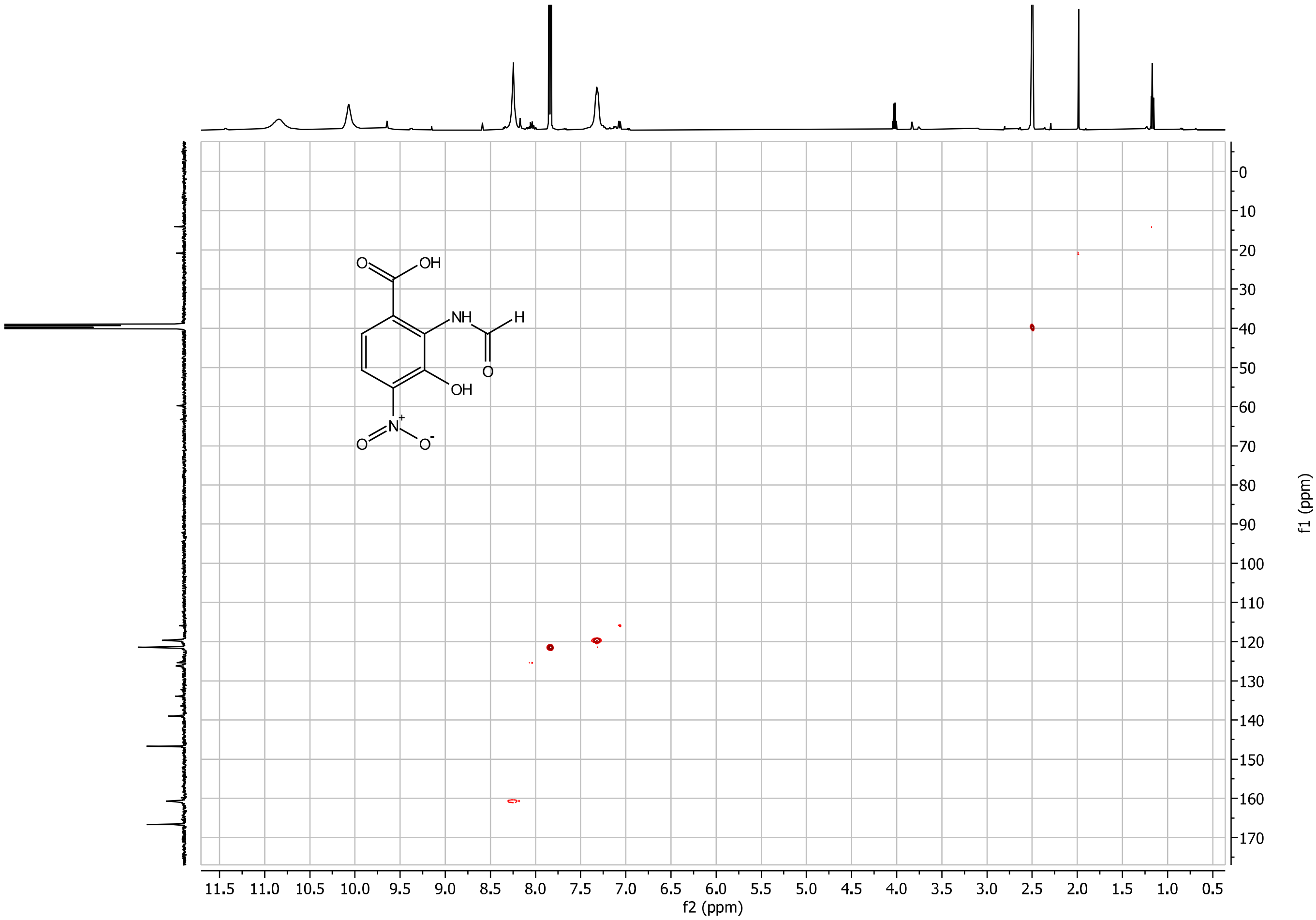
f2 (ppm)
f1 (ppm)

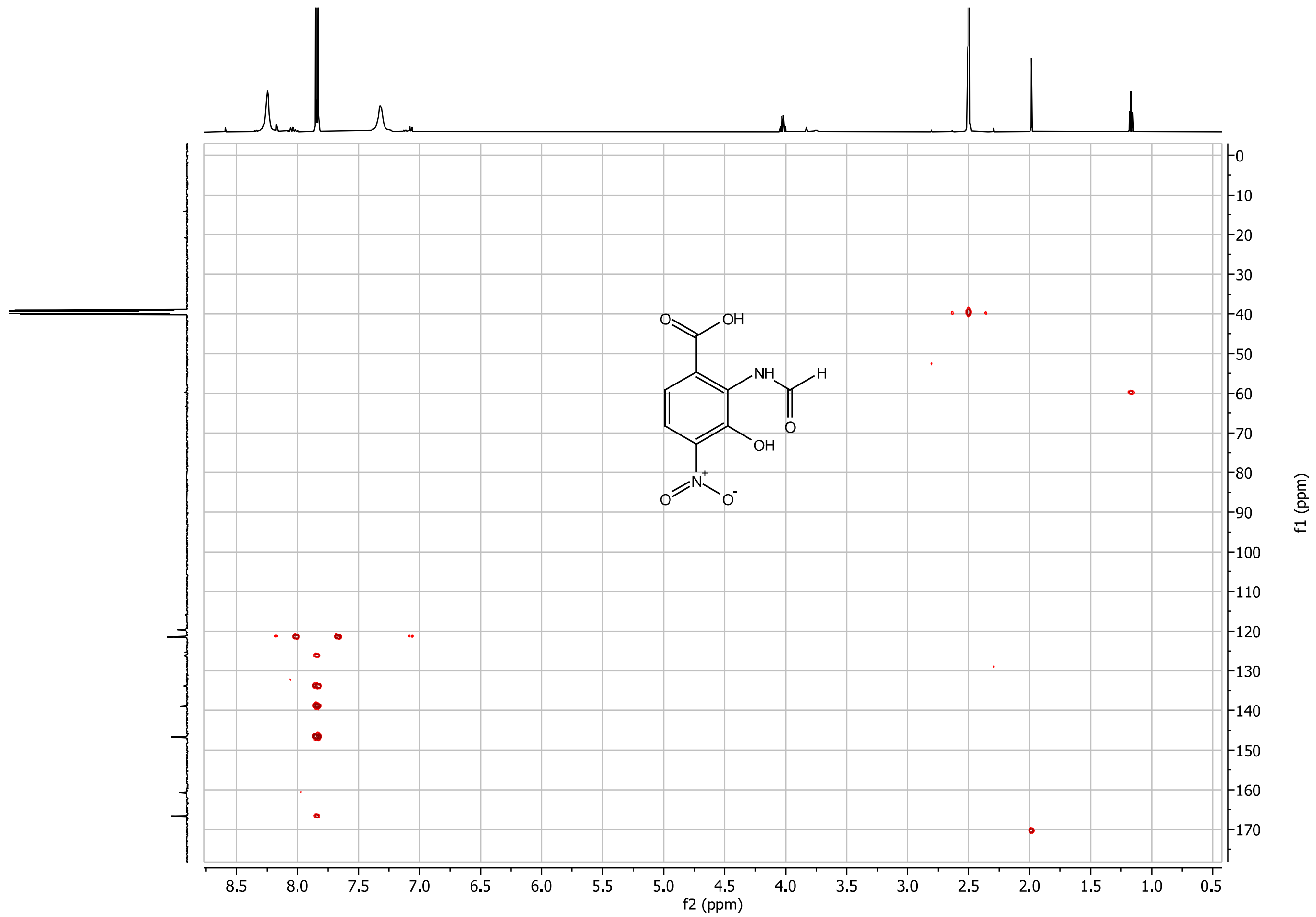
OH
NH
H
O
OH
O
N
O
f2 (ppm)
f1 (ppm)

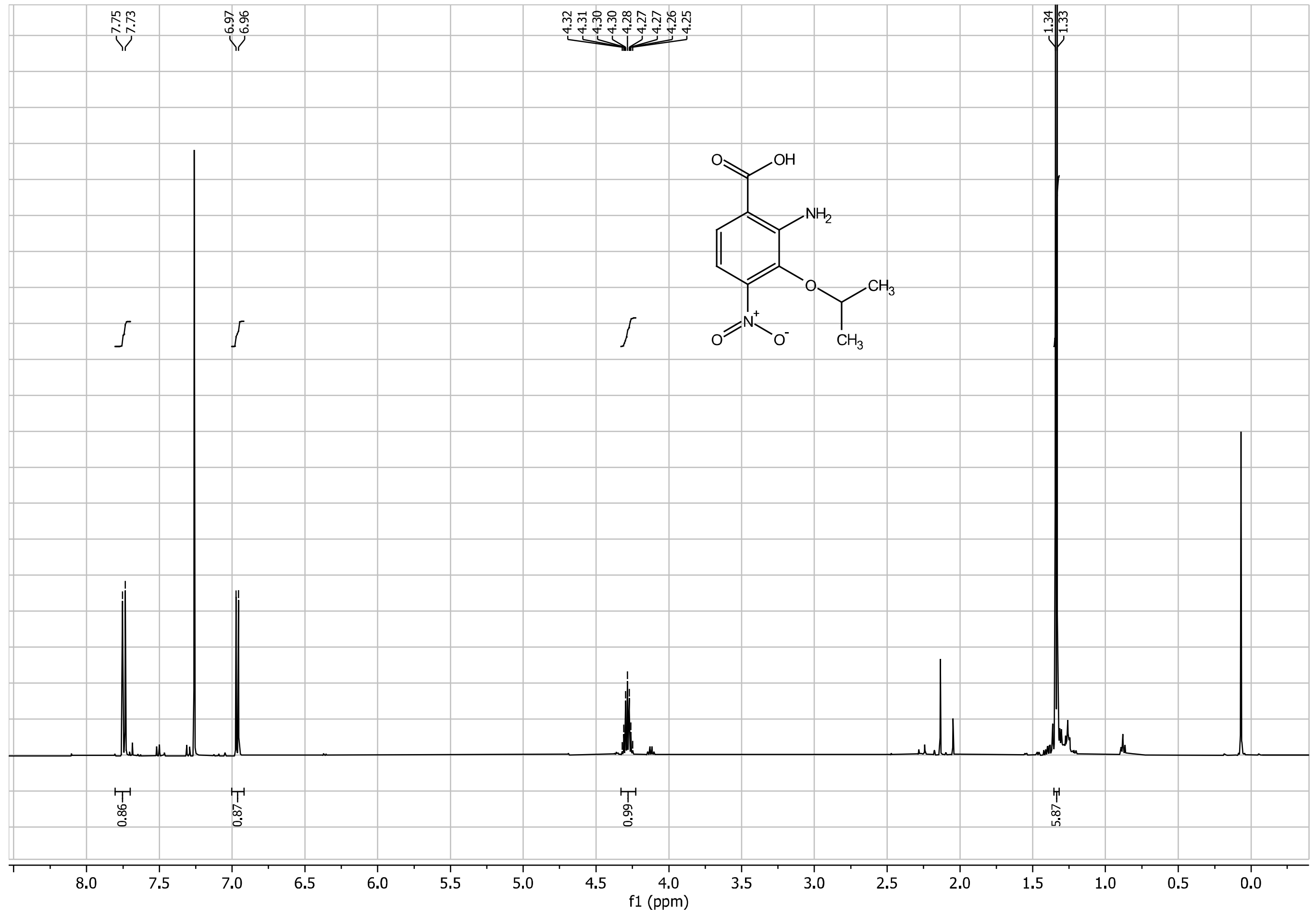
7.75
7.73
6.97
6.96
4.32
4.31
4.30
4.30
4.28
4.27
4.27
4.26
4.25
1.34
1.33
OH
NH2
O
CH3
CH3
0.86
0.87
0.99
5.87
f1 (ppm)

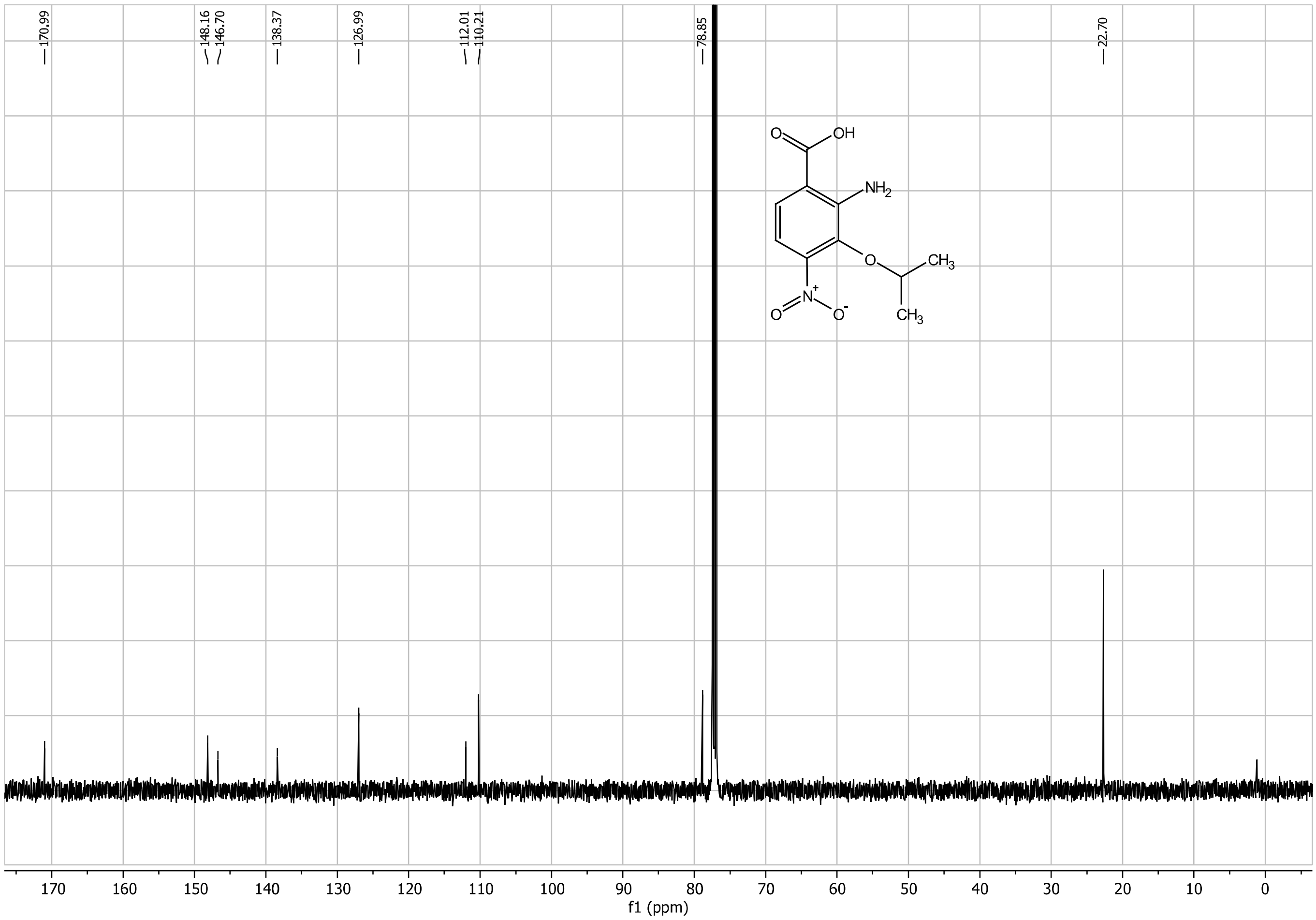
170.99
148.16
146.70
138.37
126.99
112.01
110.21
78.85
22.70
O
OH
NH2
O
CH3
CH3
N+
O-
f1 (ppm)

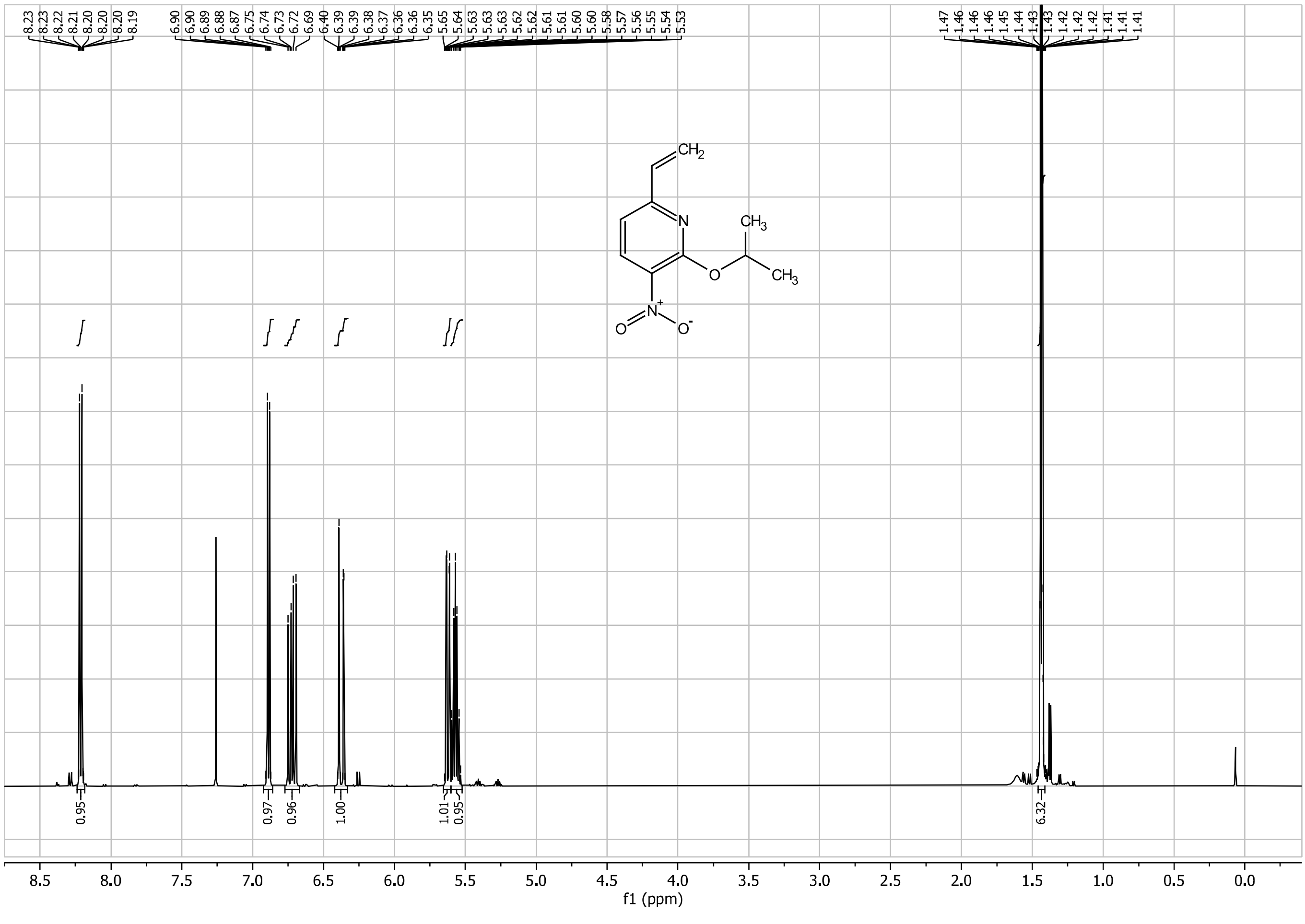
CH2
N
CH3
O
CH3
N+
O
O-
0.95
0.97
0.96
1.00
1.01
0.95
6.32
f1 (ppm)

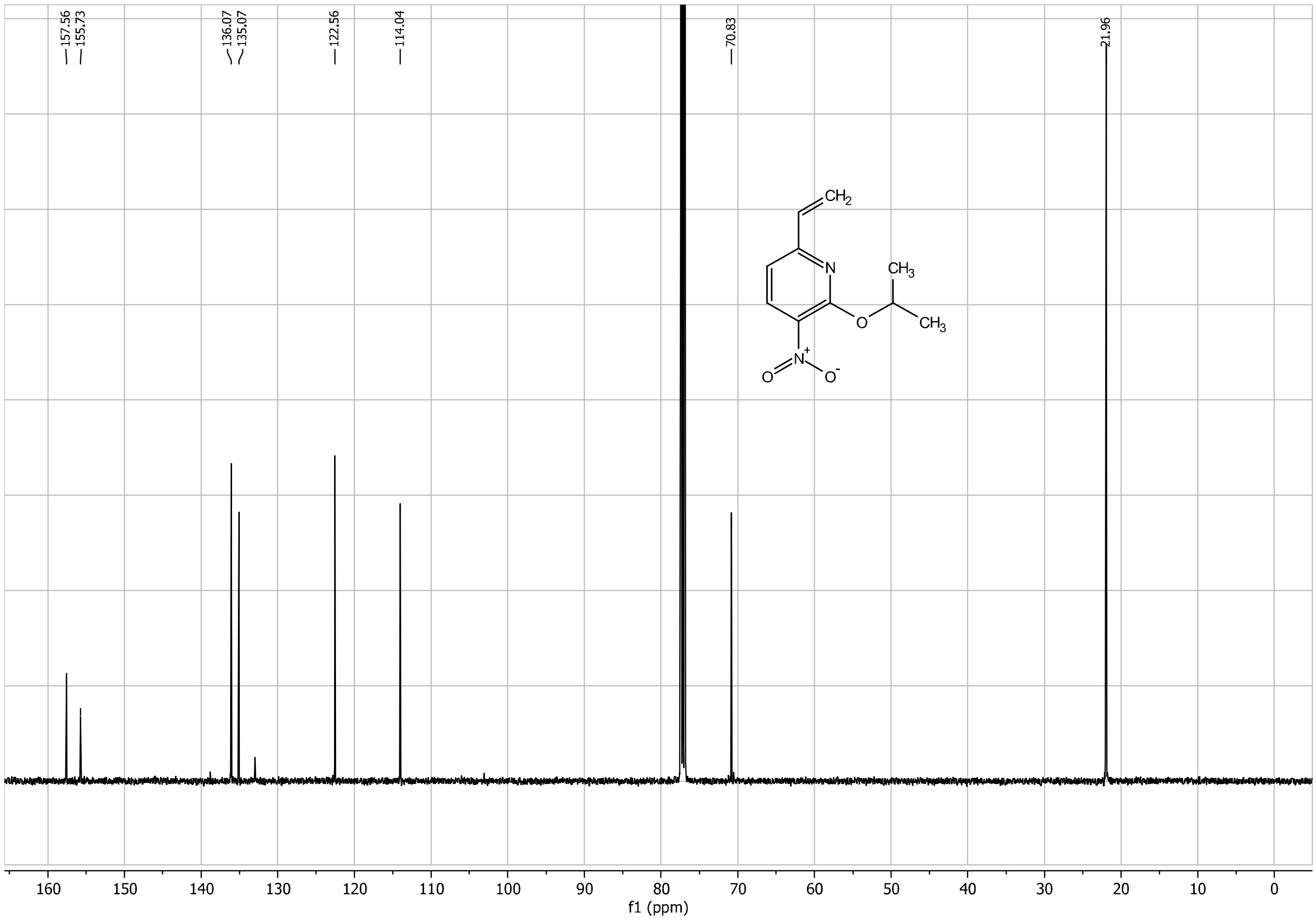
157.56
155.73
136.07
135.07
122.56
114.04
70.83
21.96
CH2
N
CH3
O
CH3
N+
O
O-
f1 (ppm)

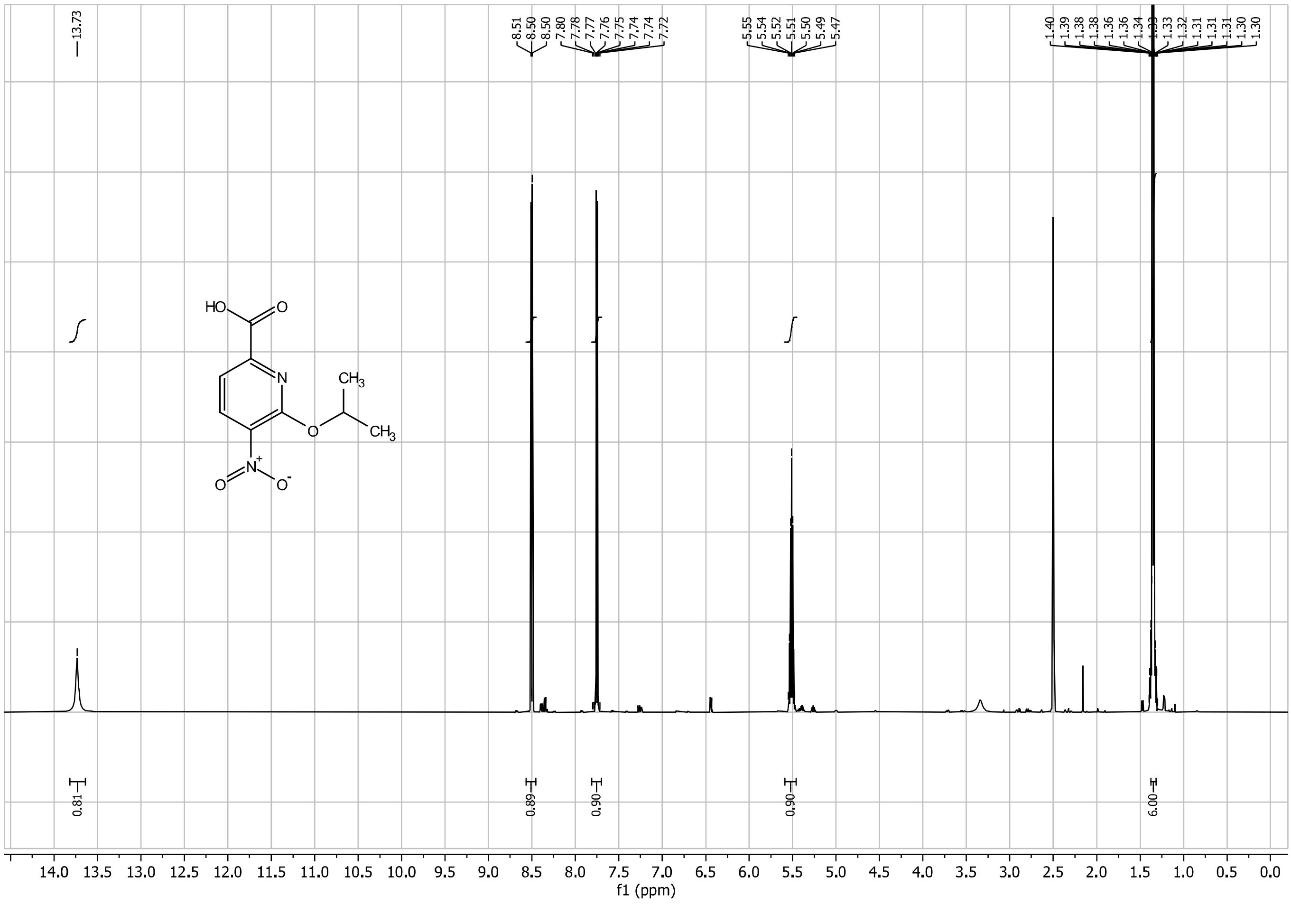
13.73
8.51
8.50
8.50
7.80
7.78
7.77
7.76
7.75
7.74
7.74
7.72
5.55
5.54
5.52
5.51
5.50
5.49
5.47
1.40
1.39
1.38
1.38
1.36
1.34
1.33
1.33
1.32
1.31
1.31
1.31
1.30
1.30
HO
O
N
CH3
O
CH3
N+
O
O-
0.81
0.89
0.90
0.90
6.00
f1 (ppm)

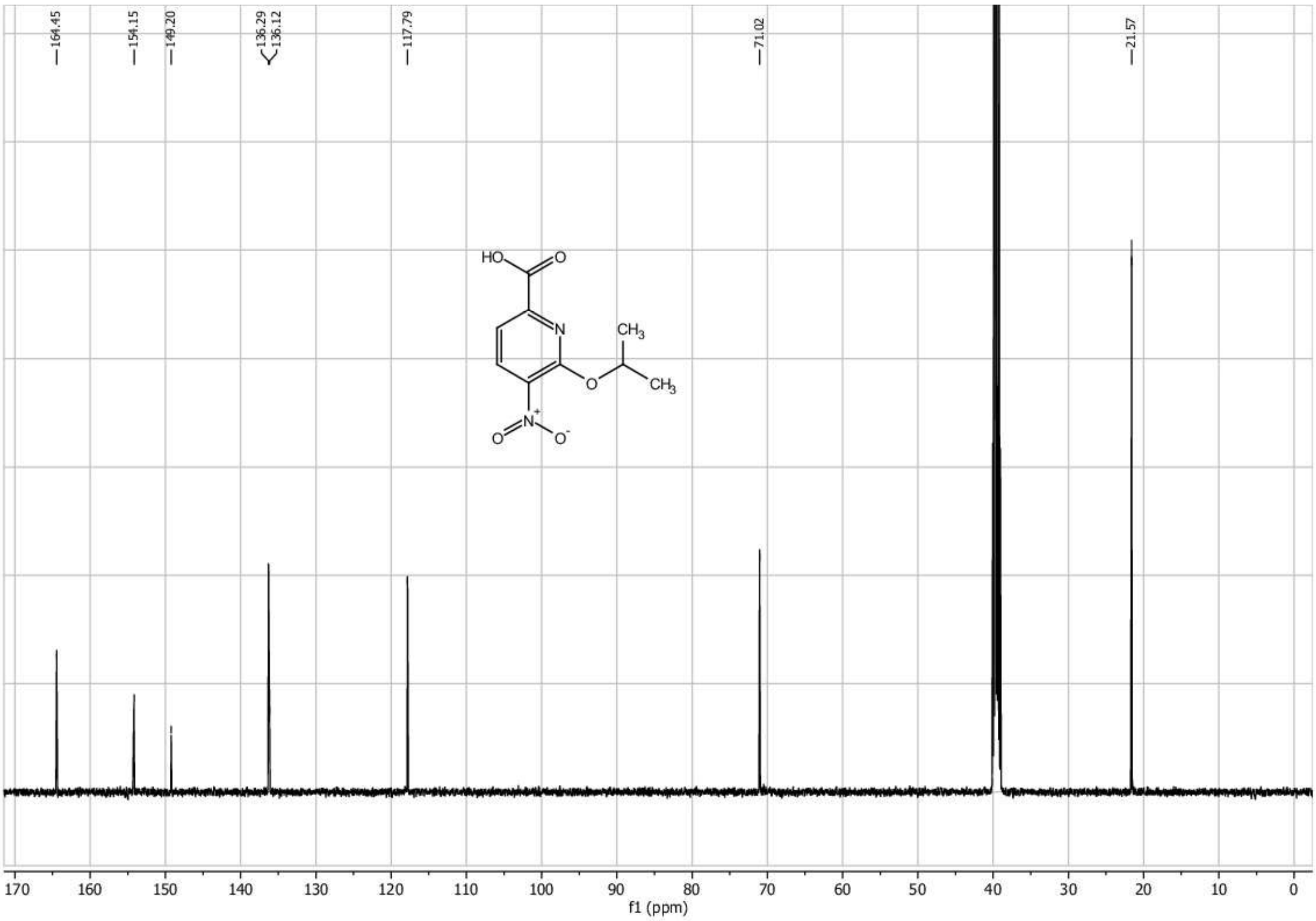
164.45
154.15
149.20
136.29
136.12
117.79
71.02
21.57
HO
O
N
CH3
O
CH3
N+
O
O-
170
160
150
140
130
120
110
100
90
80
70
60
50
40
30
20
10
0
f1 (ppm)

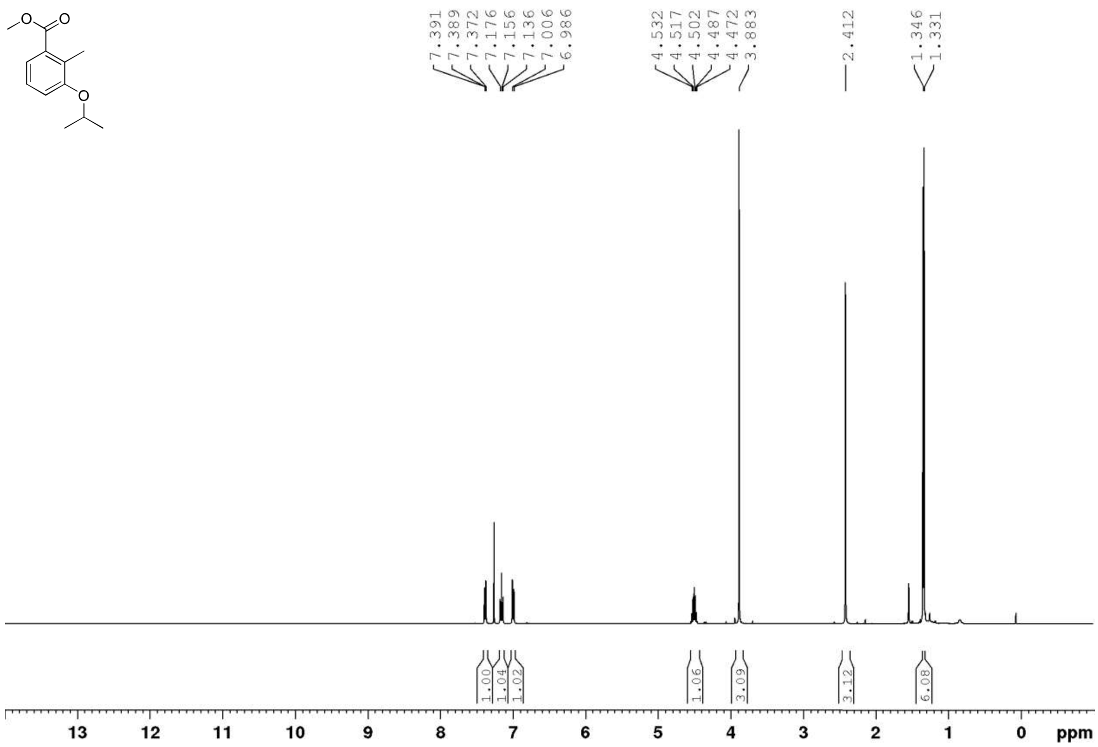
O
O
7.391
7.389
7.372
7.176
7.156
7.136
7.006
6.986
4.532
4.517
4.502
4.487
4.472
3.883
2.412
1.346
1.331
1.00
1.04
1.02
1.06
3.09
3.12
6.08
13
12
11
10
9
8
7
6
5
4
3
2
1
0
ppm

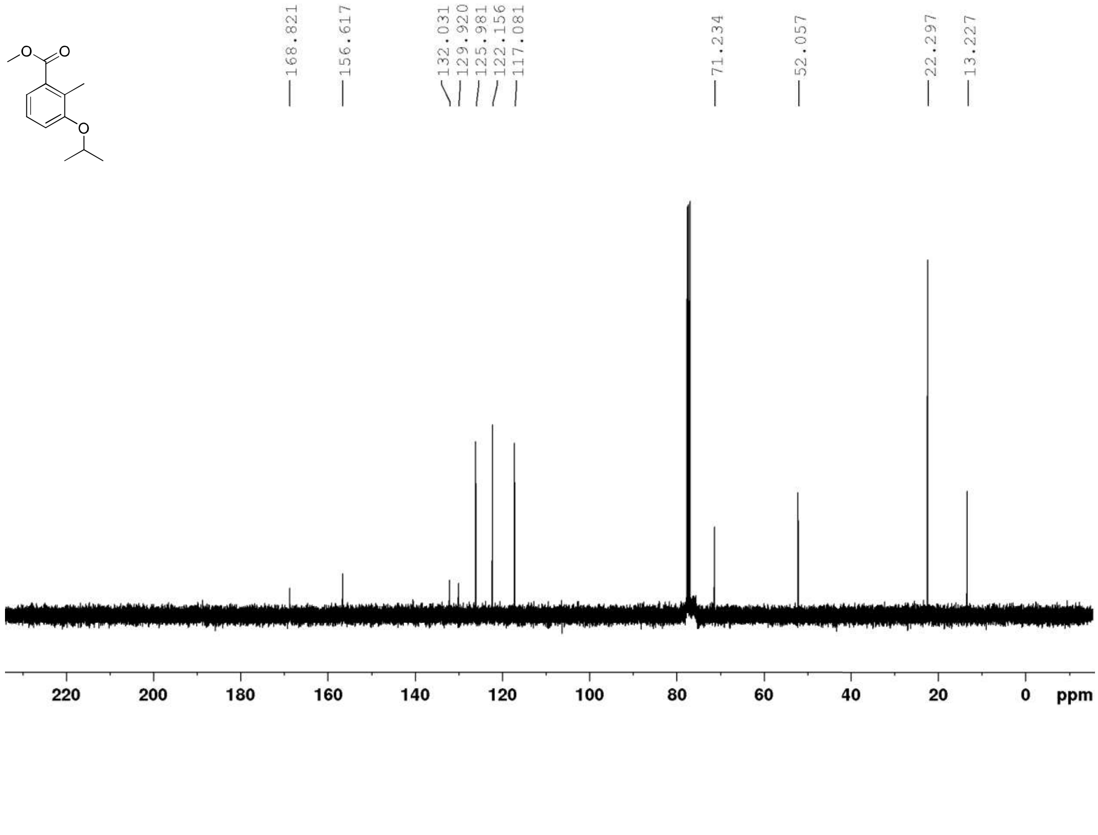
O
O
168.821
156.617
132.031
129.920
125.981
122.156
117.081
71.234
52.057
22.297
13.227
220
200
180
160
140
120
100
80
60
40
20
0
ppm

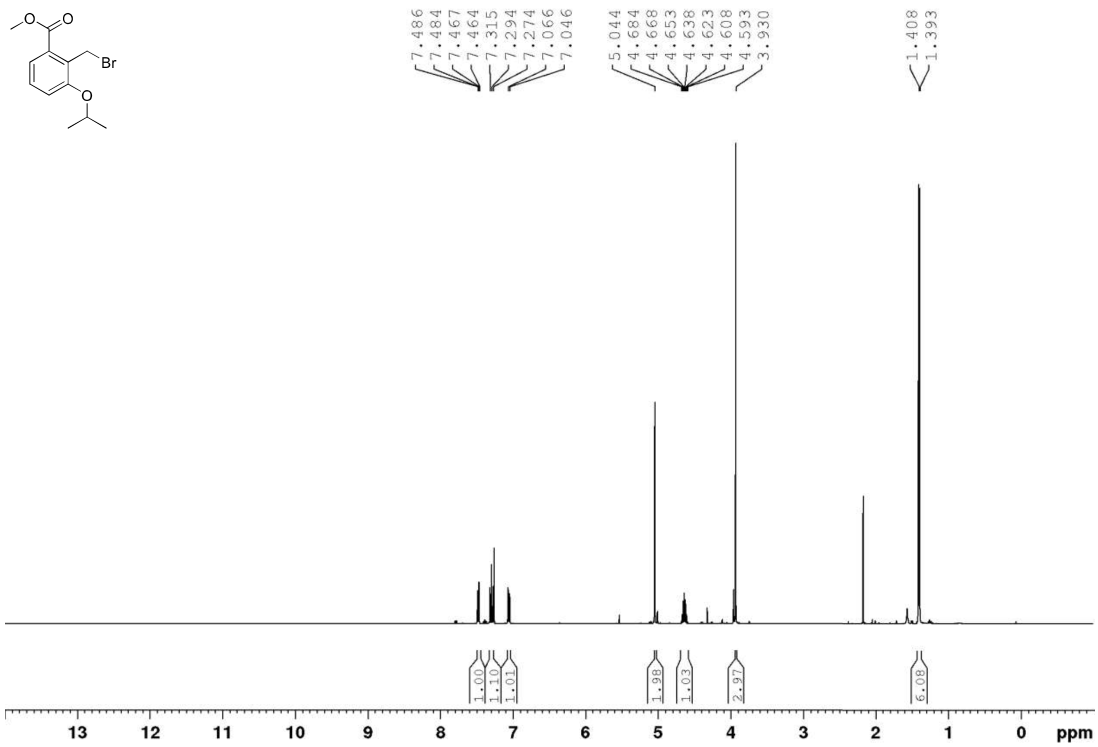
Br
O
7.486
7.484
7.467
7.464
7.315
7.294
7.274
7.066
7.046
5.044
4.684
4.668
4.653
4.638
4.623
4.608
4.593
3.930
1.408
1.393
1.00
1.10
1.01
1.98
1.03
2.97
6.08
13
12
11
10
9
8
7
6
5
4
3
2
1
0
ppm

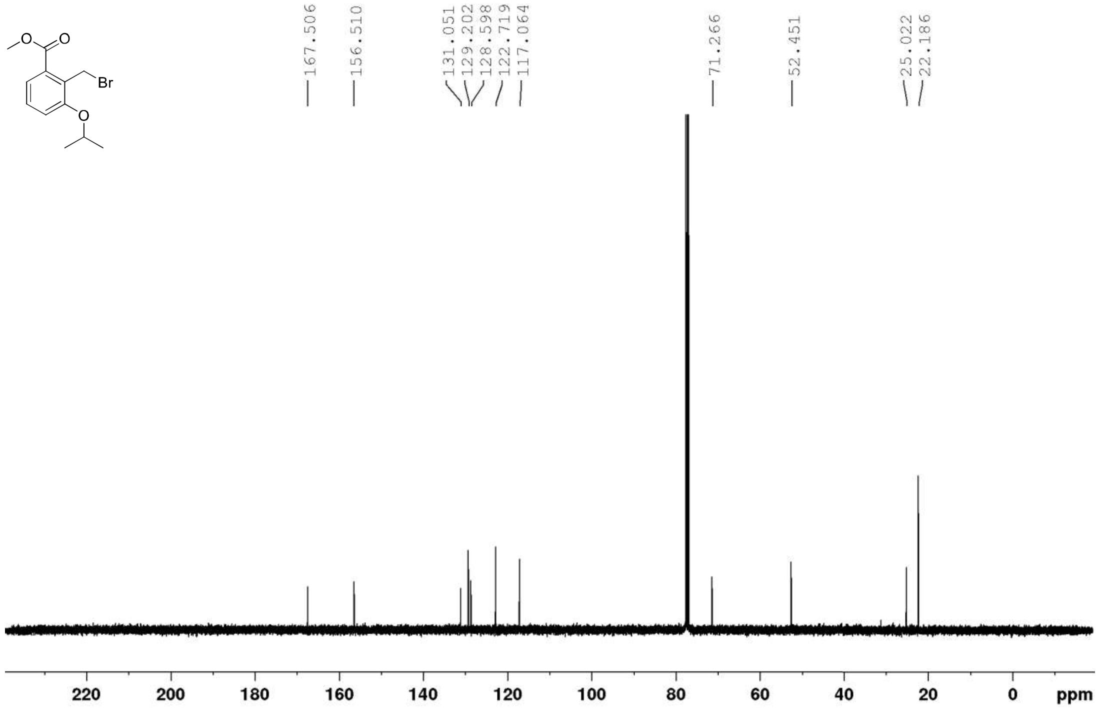
Br
O
167.506
156.510
131.051
129.202
128.598
122.719
117.064
71.266
52.451
25.022
22.186
220
200
180
160
140
120
100
80
60
40
20
0
ppm

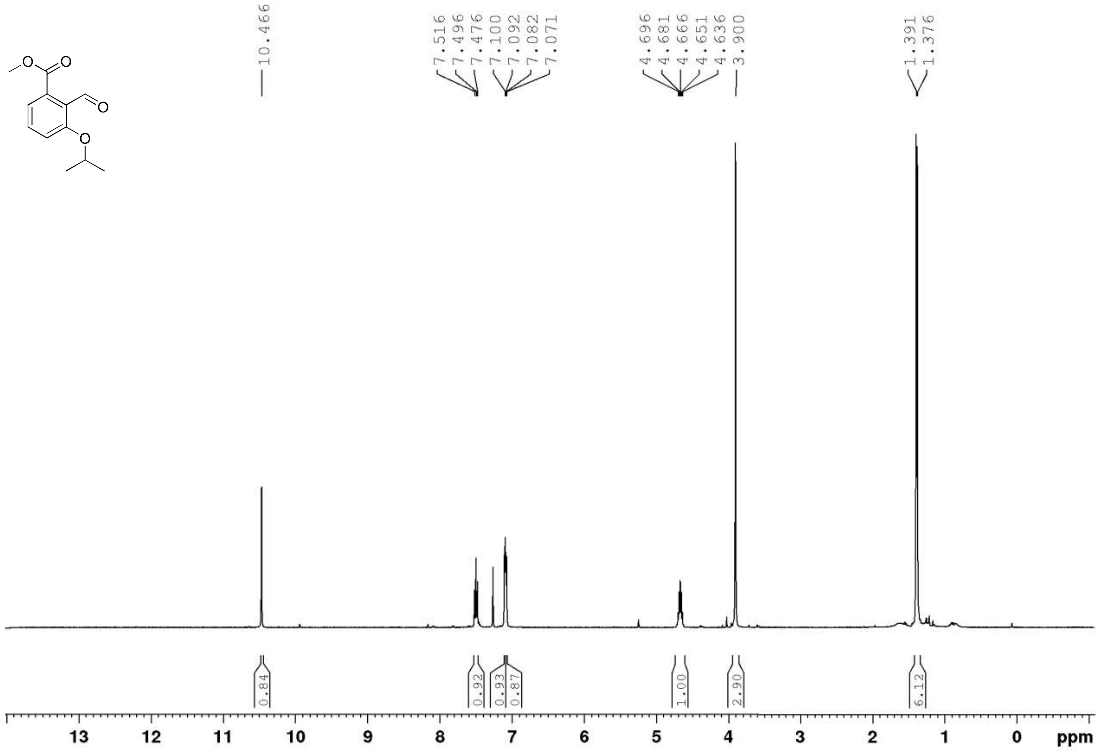

10.466
7.516
7.496
7.476
7.100
7.092
7.082
7.071
4.696
4.681
4.666
4.651
4.636
3.900
1.391
1.376
0.84
0.92
0.93
0.87
1.00
2.90
6.12
13 12 11 10 9 8 7 6 5 4 3 2 1 0 ppm

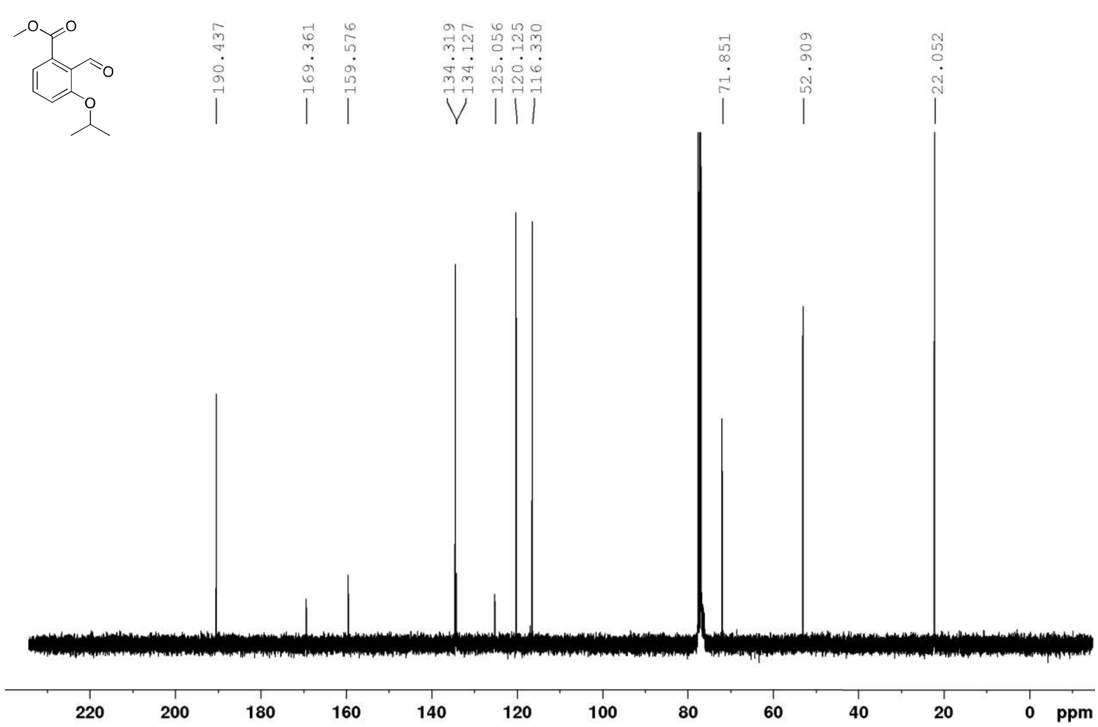

190.437
169.361
159.576
134.319
134.127
125.056
120.125
116.330
71.851
52.909
22.052
220 200 180 160 140 120 100 80 60 40 20 0 ppm

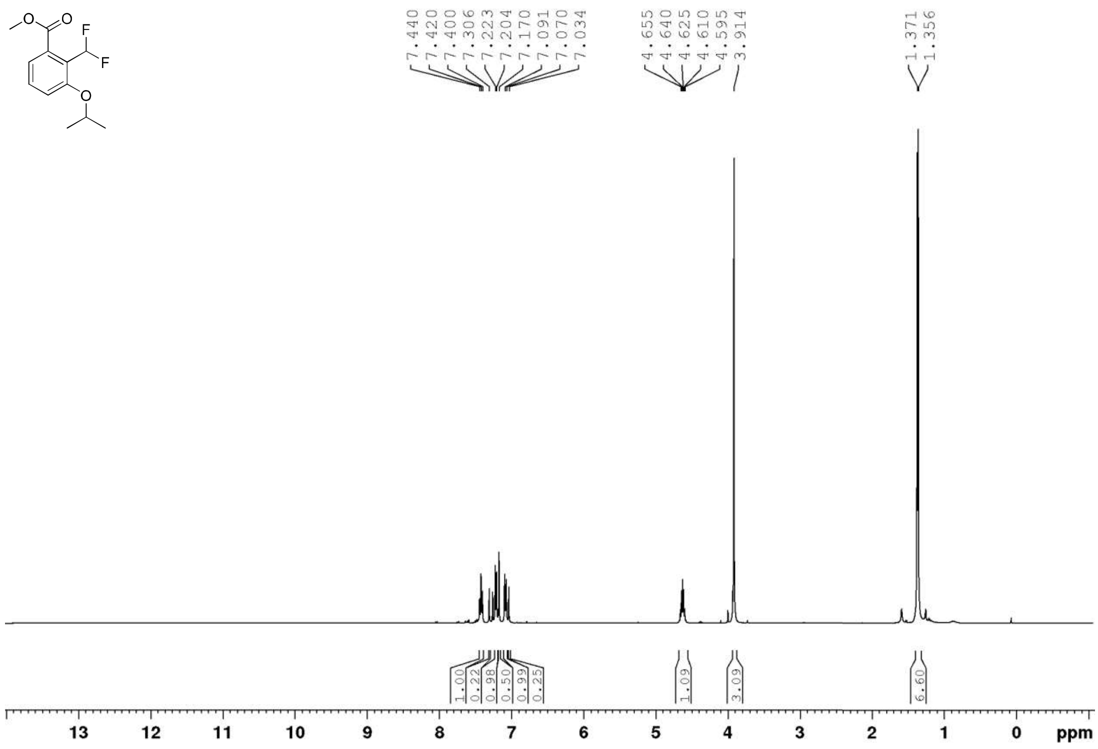
O O F F O
7.440 7.420 7.400 7.306 7.223 7.204 7.170 7.091 7.070 7.034
4.655 4.640 4.625 4.610 4.595 3.914
1.371 1.356
1.00 0.22 0.98 0.50 0.99 0.25
1.09
3.09
6.60
13 12 11 10 9 8 7 6 5 4 3 2 1 0 ppm

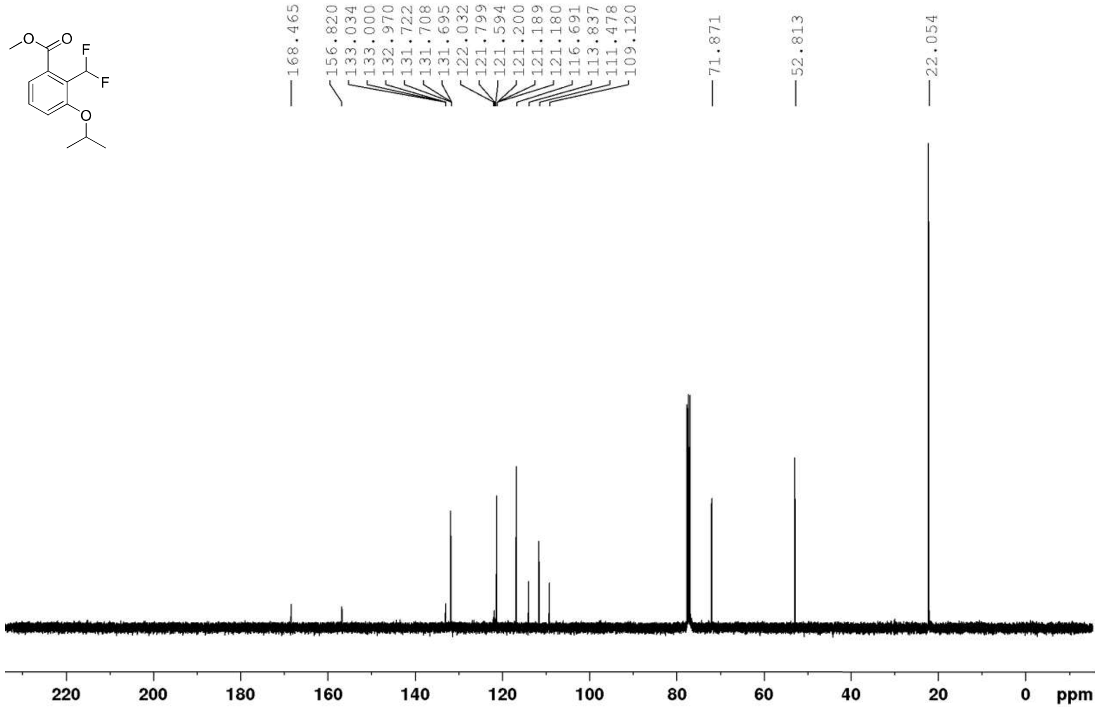
O O F F O
168.465
156.820 133.034 133.000 132.970 131.722 131.708 131.695 122.032 121.799 121.594 121.200 121.189 121.180 116.691 113.837 111.478 109.120
71.871
52.813
22.054
220 200 180 160 140 120 100 80 60 40 20 0 ppm

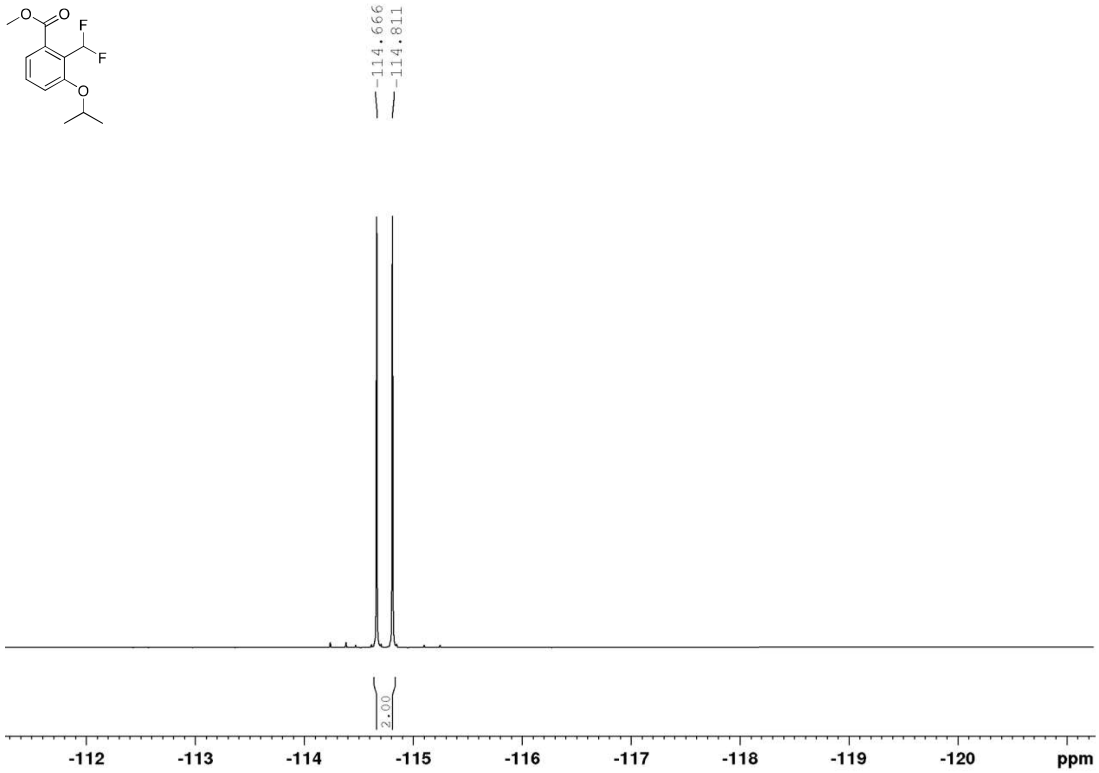
O
O
F
F
O
-114.666
-114.811
2.00
-112
-113
-114
-115
-116
-117
-118
-119
-120
ppm

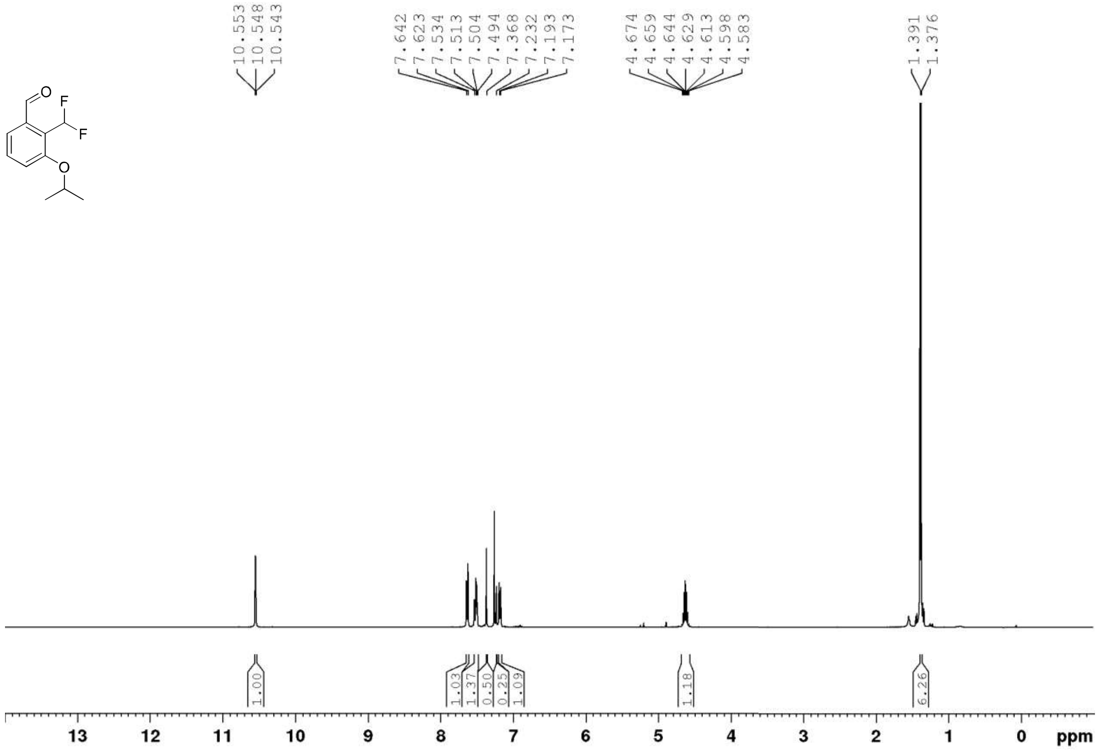
O
F
F
O
10.553
10.548
10.543
7.642
7.623
7.534
7.513
7.504
7.494
7.368
7.232
7.193
7.173
4.674
4.659
4.644
4.629
4.613
4.598
4.583
1.391
1.376
1.00
1.03
1.37
0.50
0.25
1.09
1.18
6.26
13
12
11
10
9
8
7
6
5
4
3
2
1
0
ppm

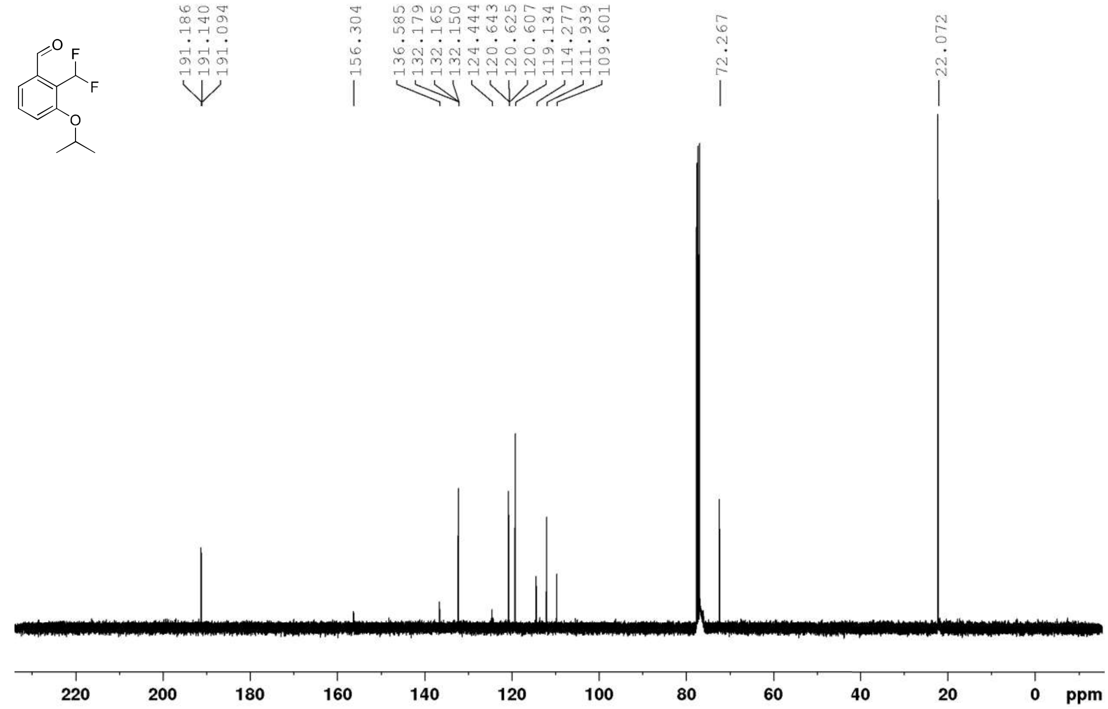
F
F
O
191.186
191.140
191.094
156.304
136.585
132.179
132.165
132.150
124.444
120.643
120.625
120.607
119.134
114.277
111.939
109.601
72.267
22.072
220 200 180 160 140 120 100 80 60 40 20 0 ppm

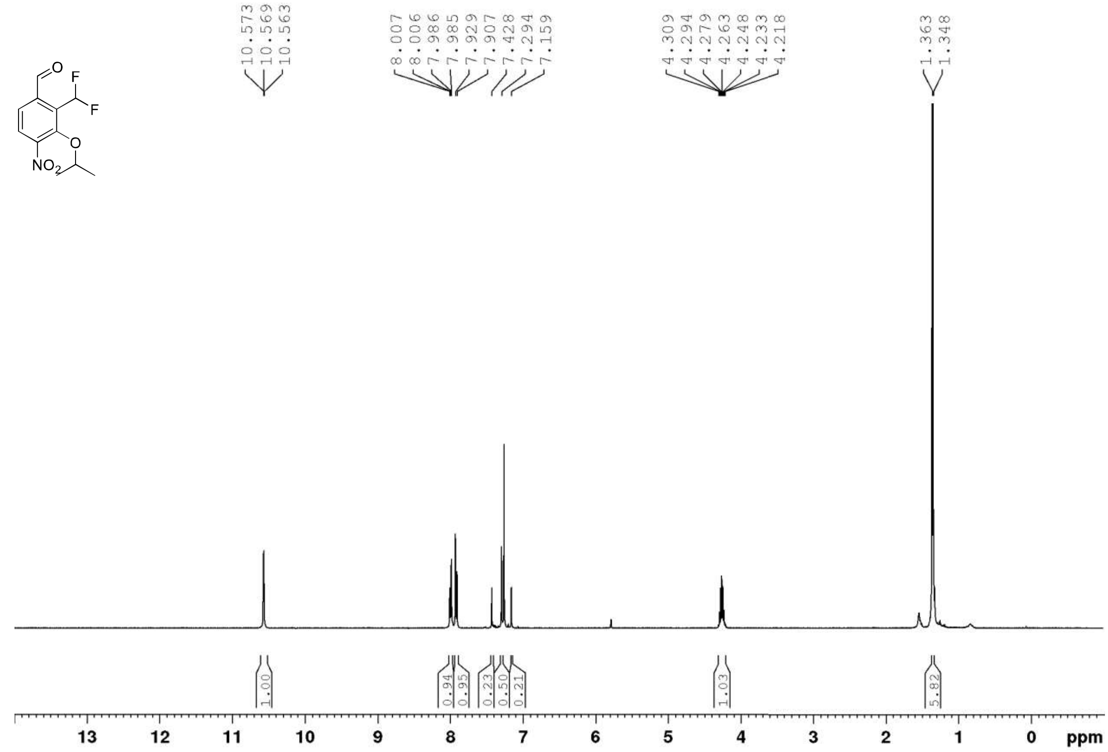
F
F
O
NO_2
10.573
10.569
10.563
8.007
8.006
7.986
7.985
7.929
7.907
7.428
7.294
7.159
4.309
4.294
4.279
4.263
4.248
4.233
4.218
1.363
1.348
1.00
0.94
0.95
0.23
0.50
0.21
1.03
5.82
13 12 11 10 9 8 7 6 5 4 3 2 1 0 ppm

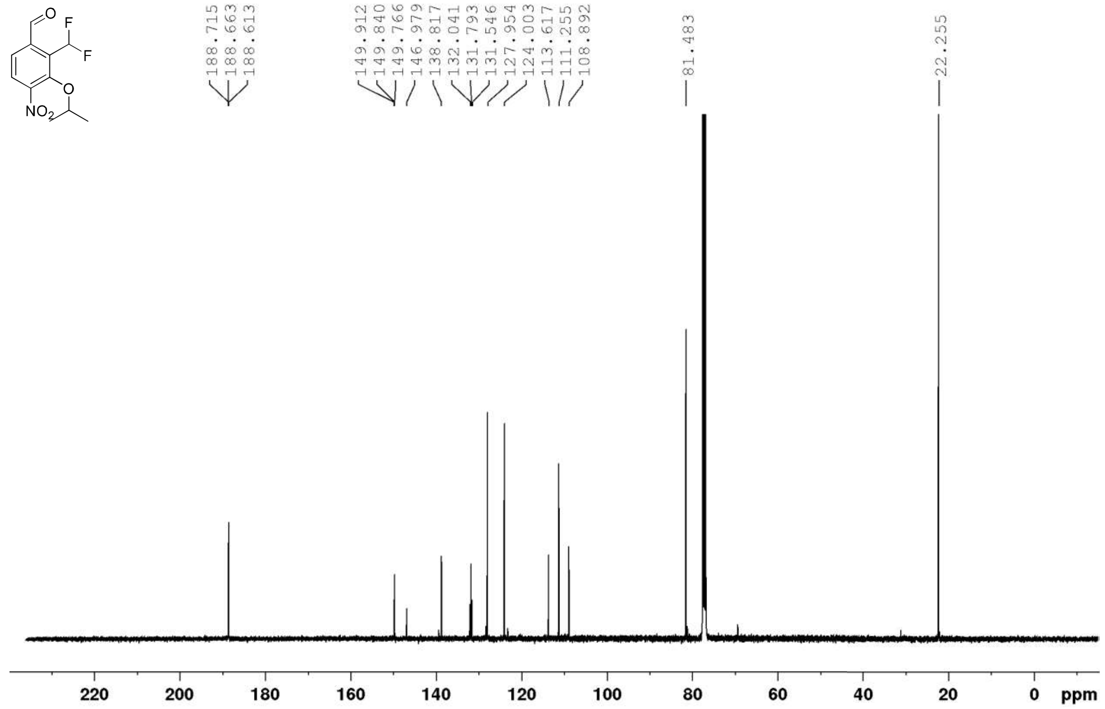
O
F
F
O
NO2
188.715
188.663
188.613
149.912
149.840
149.766
146.979
138.817
132.041
131.793
131.546
127.954
124.003
113.617
111.255
108.892
81.483
22.255
220 200 180 160 140 120 100 80 60 40 20 0 ppm

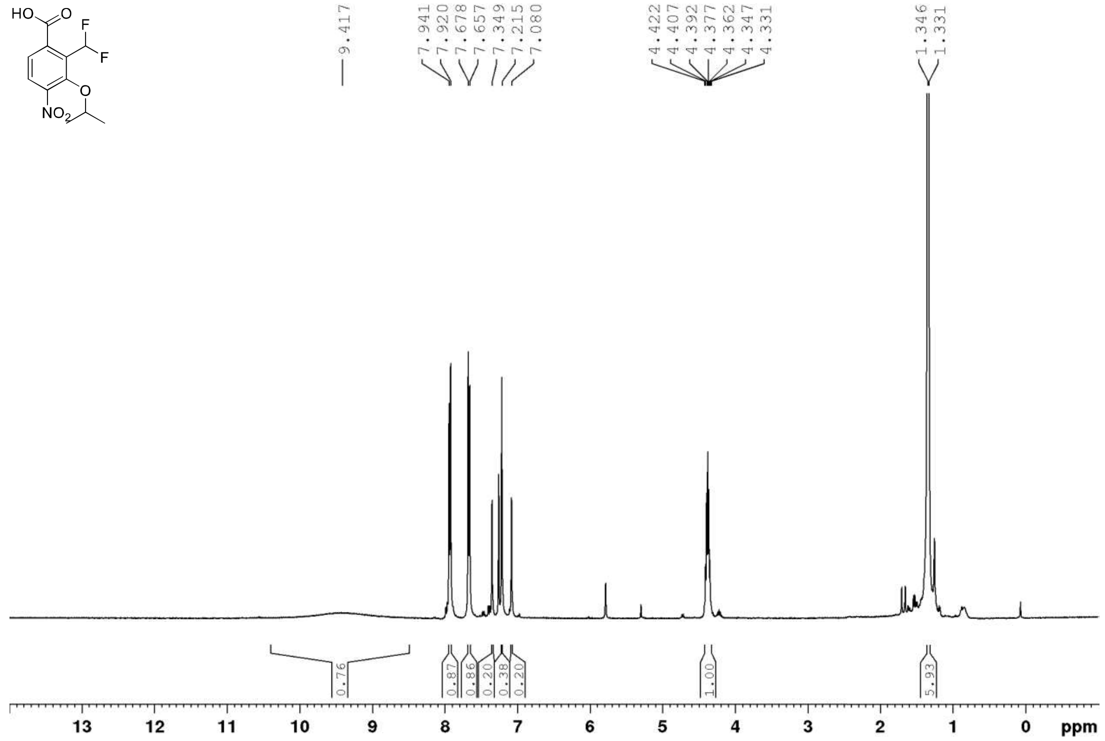
HO
O
F
F
O
NO2
9.417
7.941
7.920
7.678
7.657
7.349
7.215
7.080
4.422
4.407
4.392
4.377
4.362
4.347
4.331
1.346
1.331
0.76
0.87
0.86
0.20
0.38
0.20
1.00
5.93
13 12 11 10 9 8 7 6 5 4 3 2 1 0 ppm

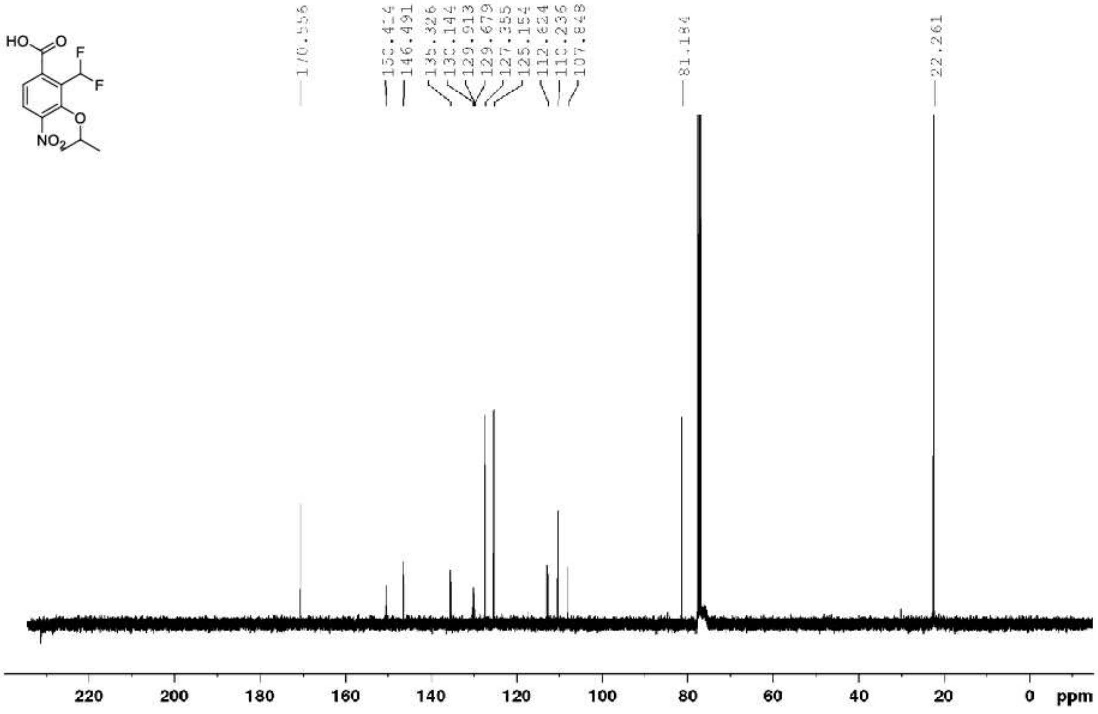
HO
O
F
F
O
NO2
146.491
135.326
129.913
129.679
127.355
112.624
81.184
22.261
220
200
180
160
140
120
100
80
60
40
20
0
ppm

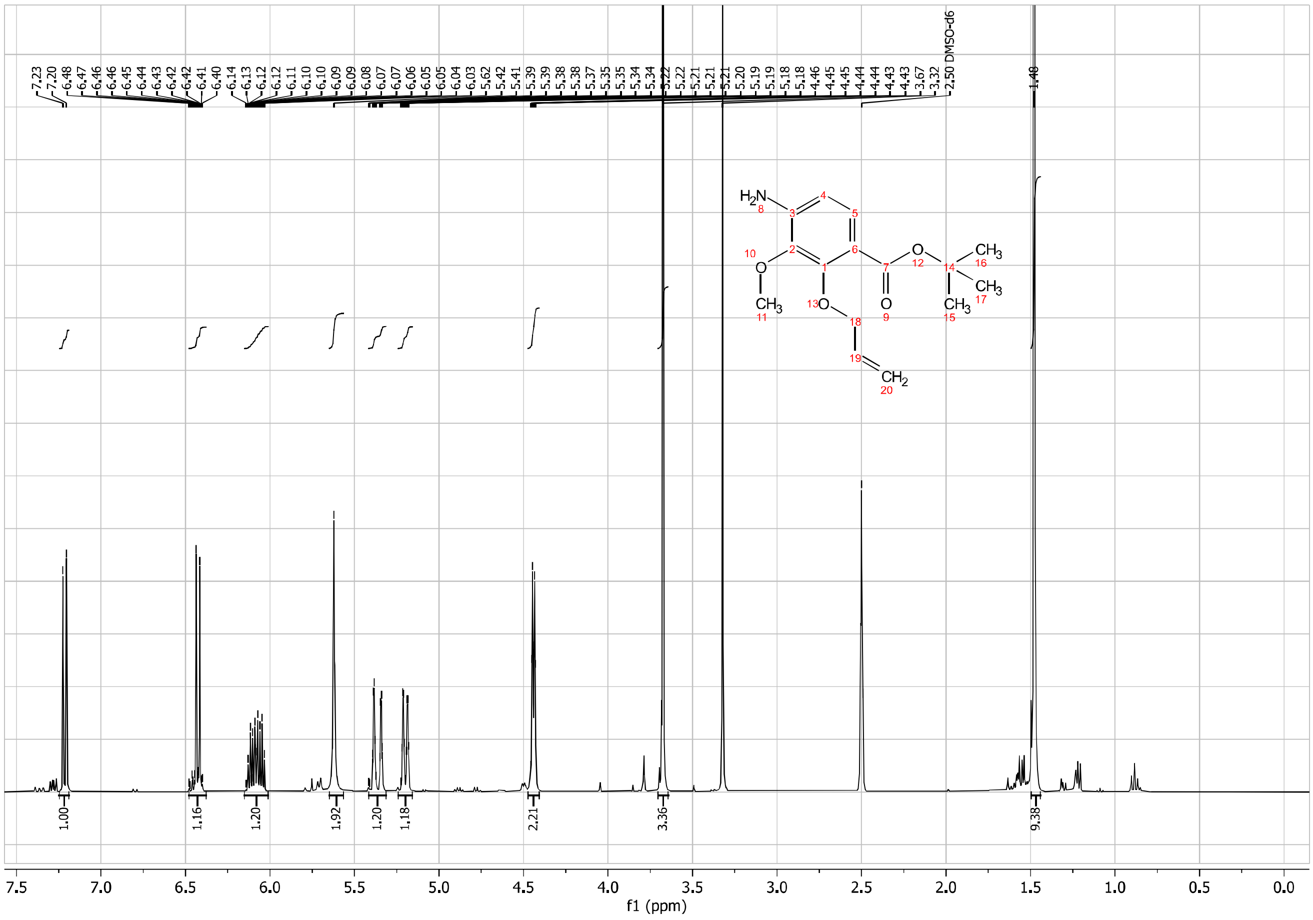
7.23
7.20
6.48
6.47
6.46
6.46
6.45
6.44
6.43
6.42
6.42
6.41
6.40
6.14
6.13
6.12
6.12
6.11
6.10
6.10
6.09
6.09
6.08
6.07
6.07
6.06
6.05
6.05
6.04
6.03
5.62
5.42
5.41
5.39
5.39
5.38
5.38
5.37
5.35
5.35
5.34
5.34
5.22
5.22
5.21
5.21
5.21
5.20
5.19
5.19
5.18
5.18
4.46
4.45
4.45
4.44
4.44
4.43
4.43
3.67
3.32
2.50 DMSO-d6
1.48
1.00
1.16
1.20
1.92
1.20
1.18
2.21
3.36
9.38
f1 (ppm)

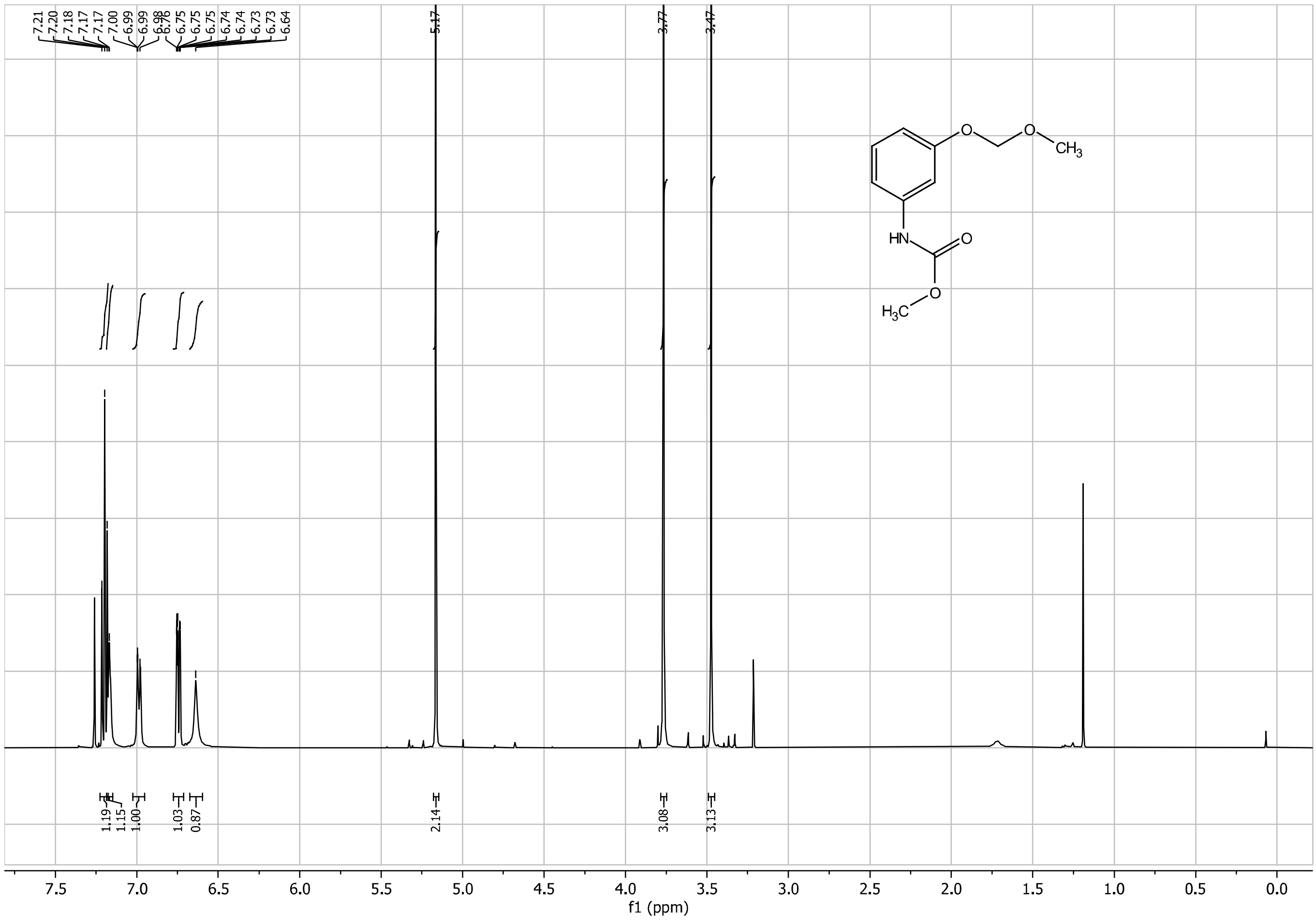
7.21
7.20
7.18
7.17
7.17
7.00
6.99
6.99
6.98
6.76
6.75
6.75
6.75
6.74
6.73
6.73
6.64
5.17
3.77
3.47
1.19
1.15
1.00
1.03
0.87
2.14
3.08
3.13
O
O
CH3
HN
O
O
H3C
7.5
7.0
6.5
6.0
5.5
5.0
4.5
4.0
3.5
3.0
2.5
2.0
1.5
1.0
0.5
0.0
f1 (ppm)

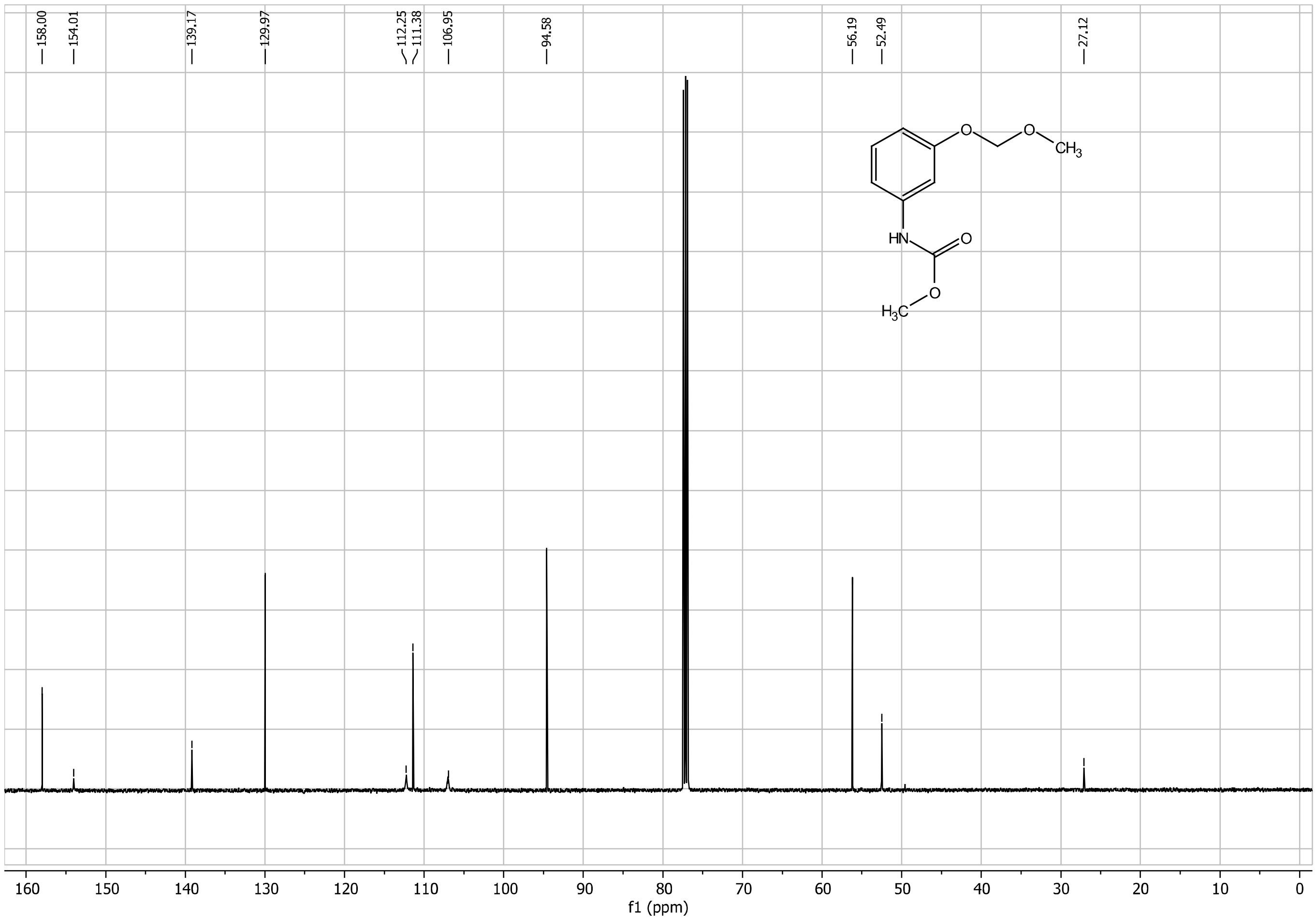
158.00
154.01
139.17
129.97
112.25
111.38
106.95
94.58
56.19
52.49
27.12
O
O
CH3
HN
O
O
H3C
160
150
140
130
120
110
100
90
80
70
60
50
40
30
20
10
0
f1 (ppm)

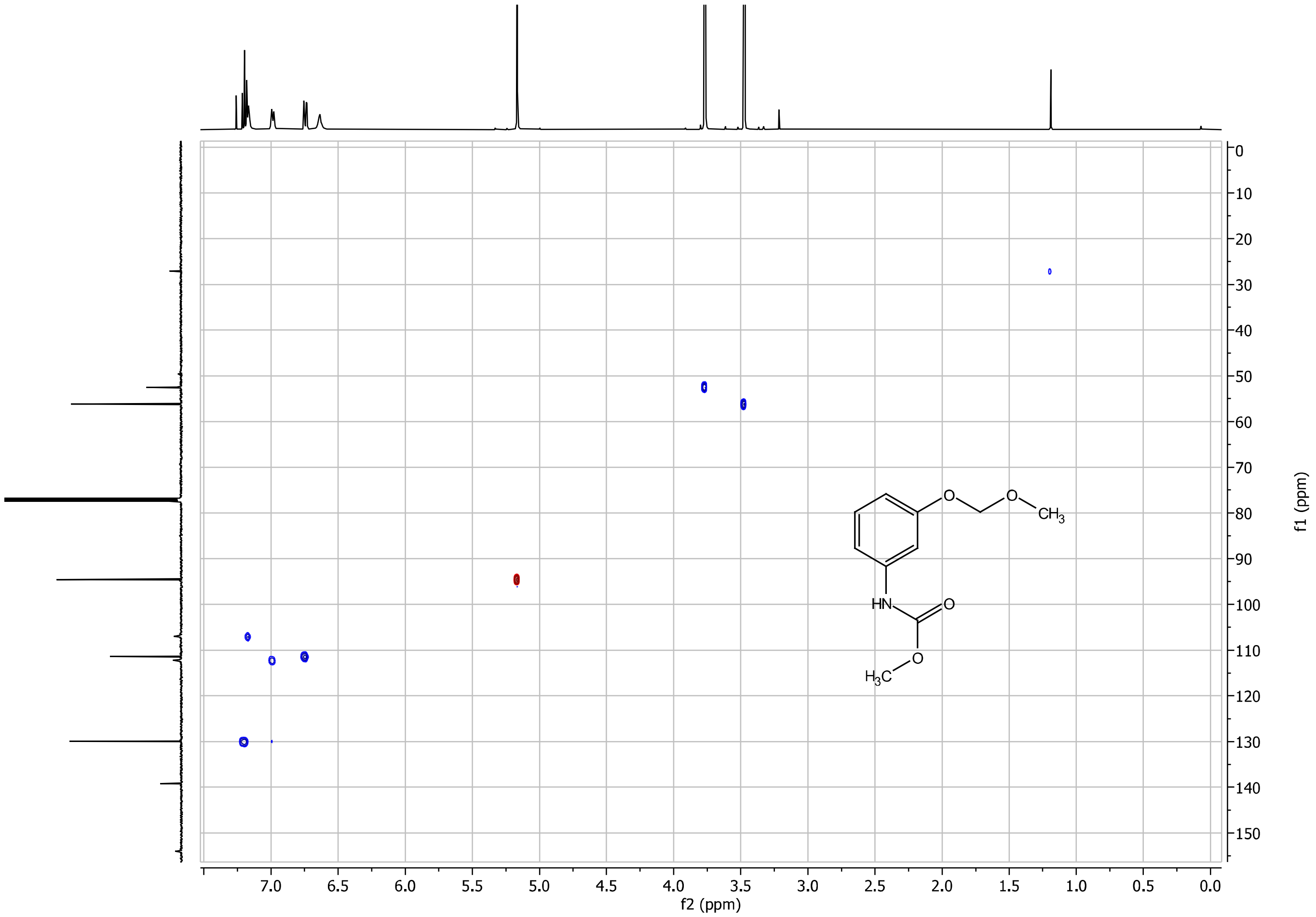

f2 (ppm)
f1 (ppm)
HN
O
H3C
CH3

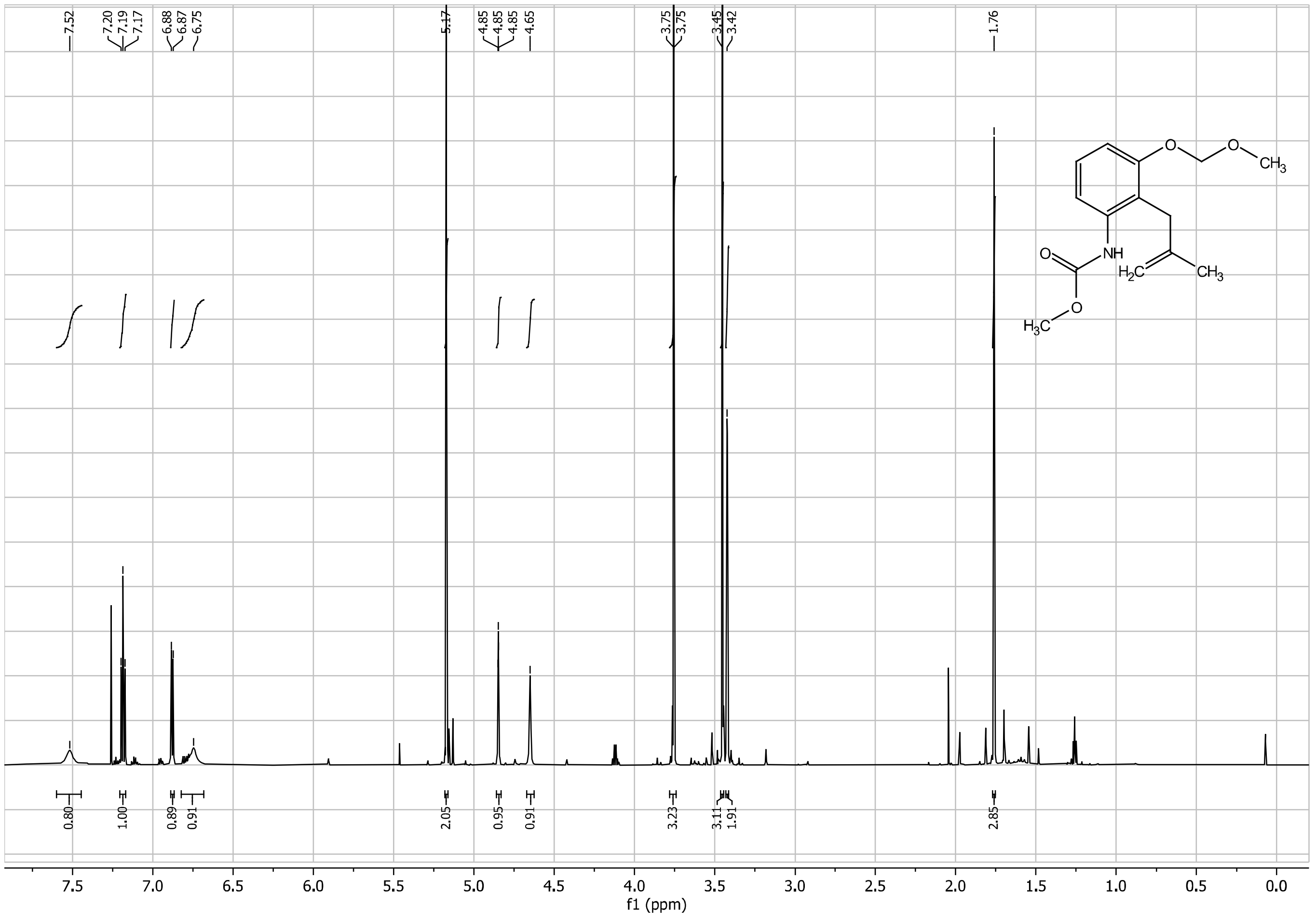

7.52
7.20
7.19
7.17
6.88
6.87
6.75
5.17
4.85
4.85
4.85
4.65
3.75
3.75
3.45
3.42
1.76
0.80
1.00
0.89
0.91
2.05
0.95
0.91
3.23
3.11
1.91
2.85
NH
H2C
CH3
H3C
f1 (ppm)

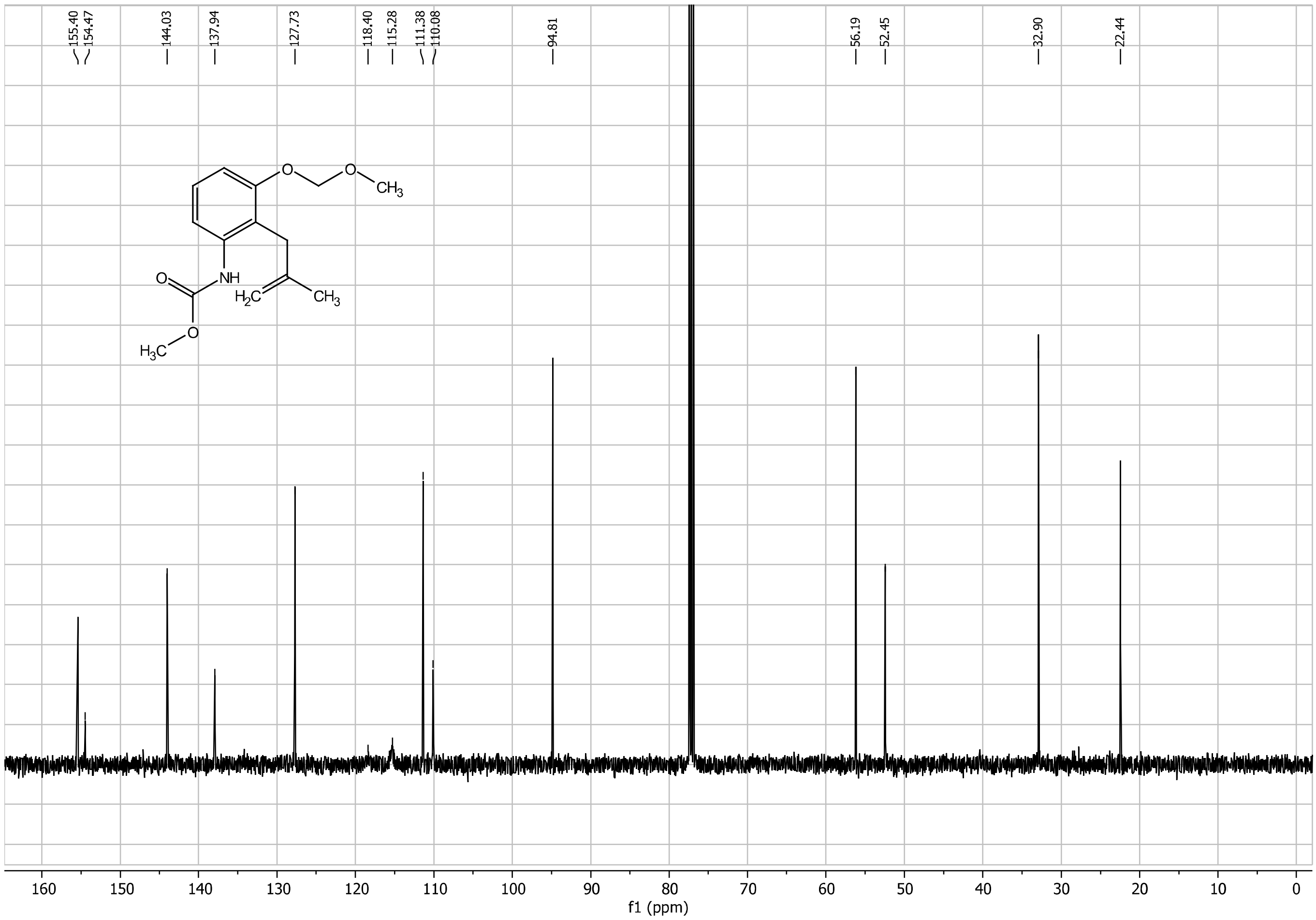
155.40
154.47
144.03
137.94
127.73
118.40
115.28
111.38
110.08
94.81
56.19
52.45
32.90
22.44
O
O
CH3
NH
O
H2C
CH3
O
H3C
160
150
140
130
120
110
100
90
80
70
60
50
40
30
20
10
0
f1 (ppm)

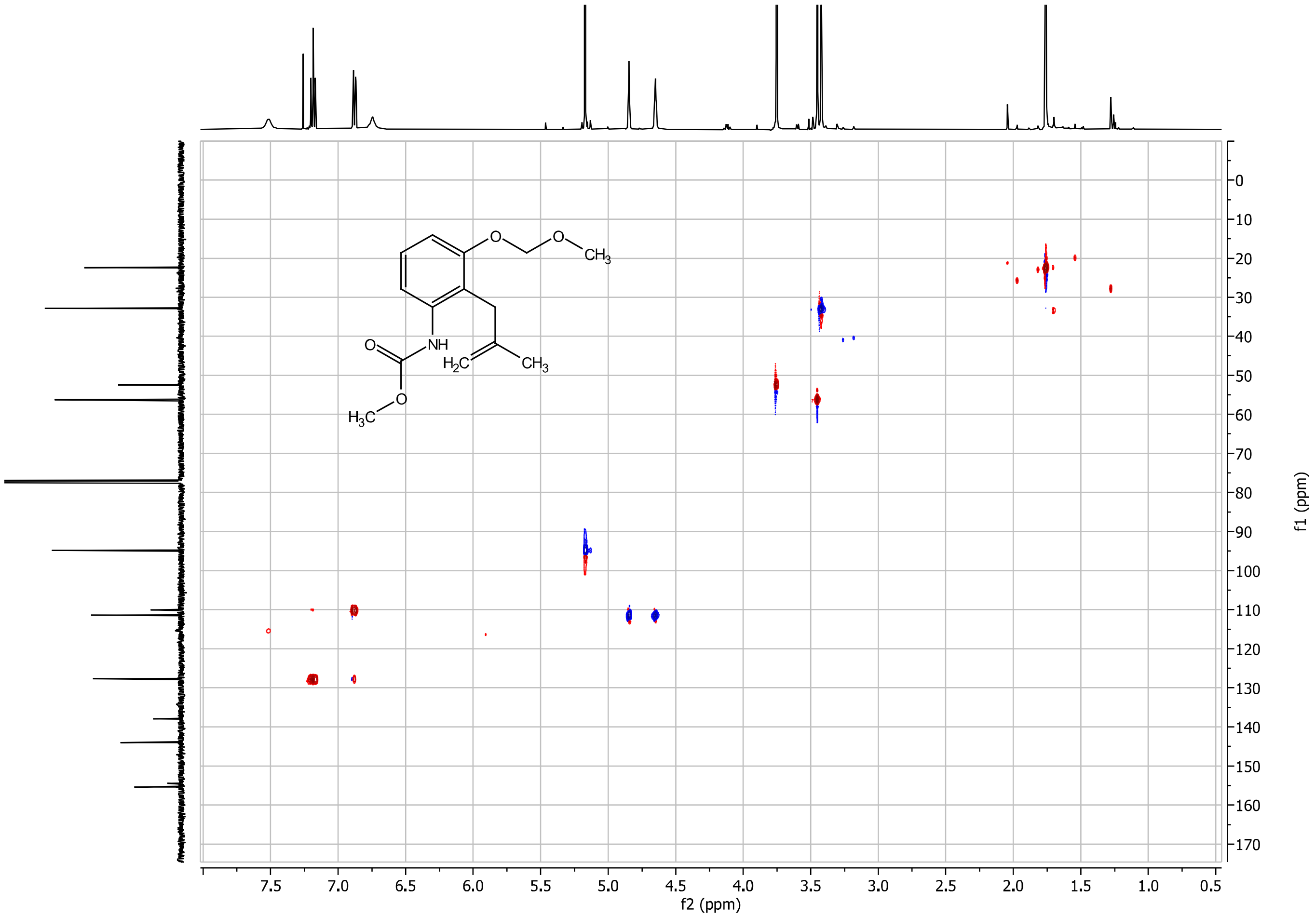
O
O
CH3
NH
O
H2C
CH3
O
H3C
7.5
7.0
6.5
6.0
5.5
5.0
4.5
4.0
3.5
3.0
2.5
2.0
1.5
1.0
0.5
f2 (ppm)
f1 (ppm)

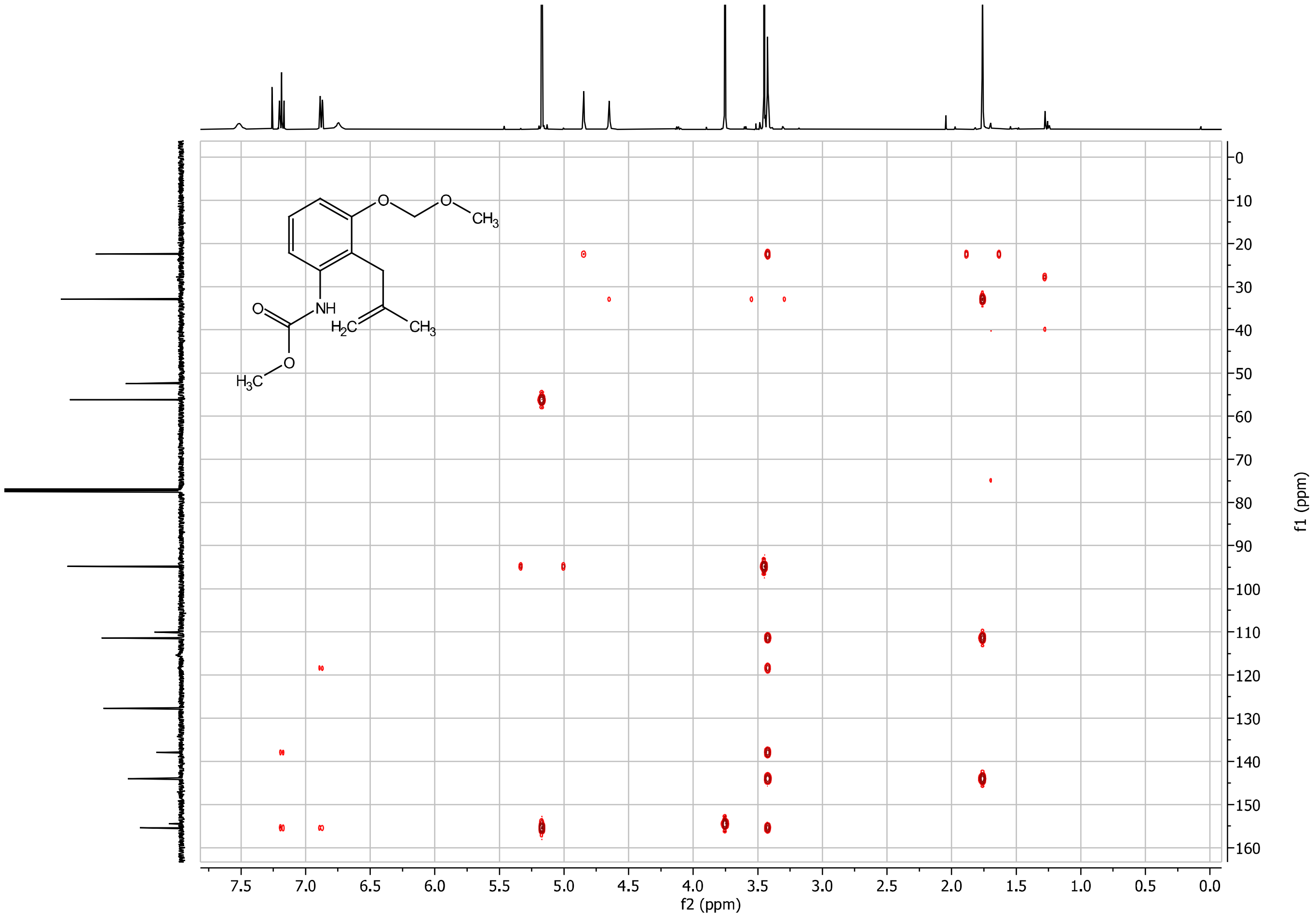

f2 (ppm)
f1 (ppm)

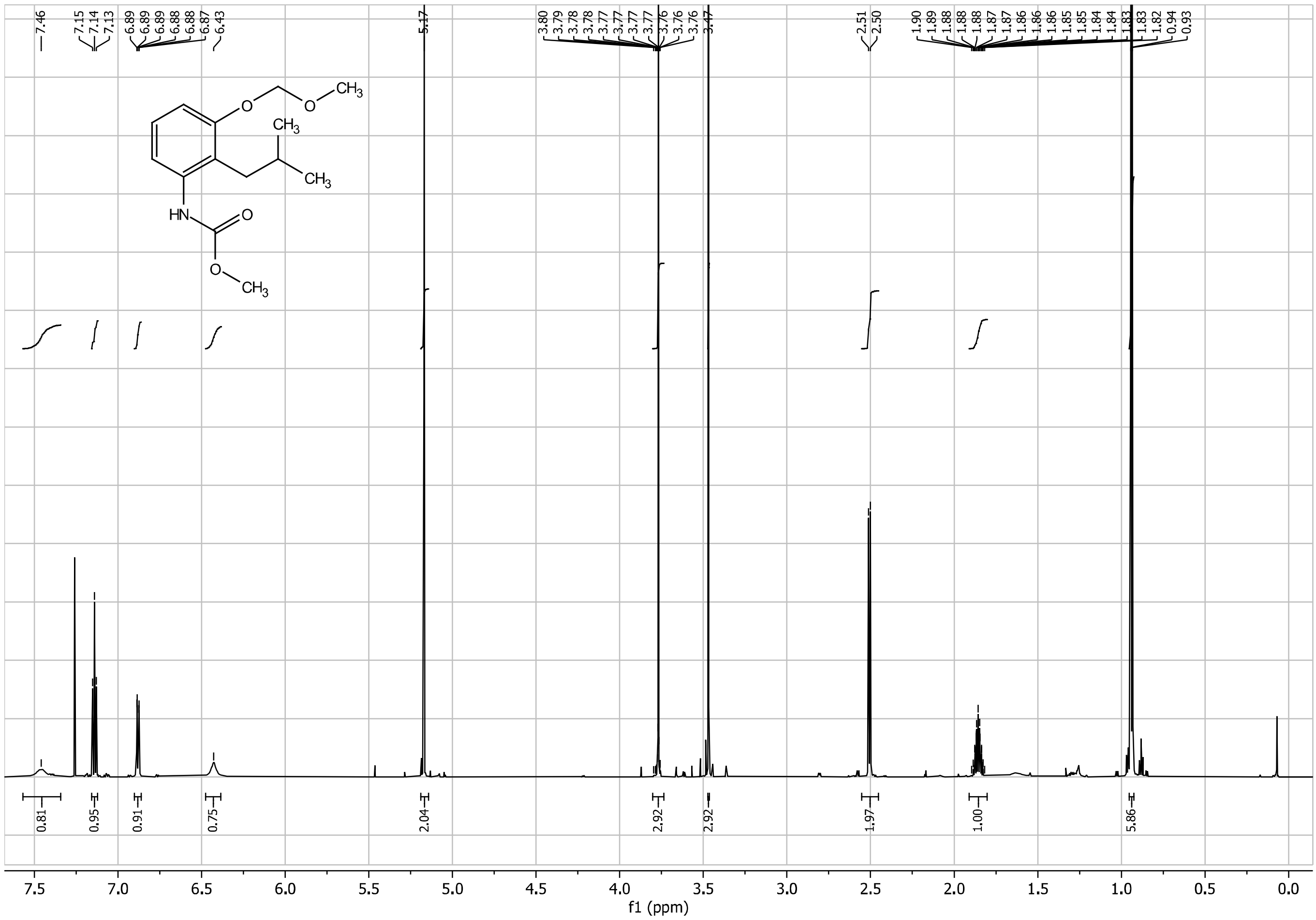

f1 (ppm)

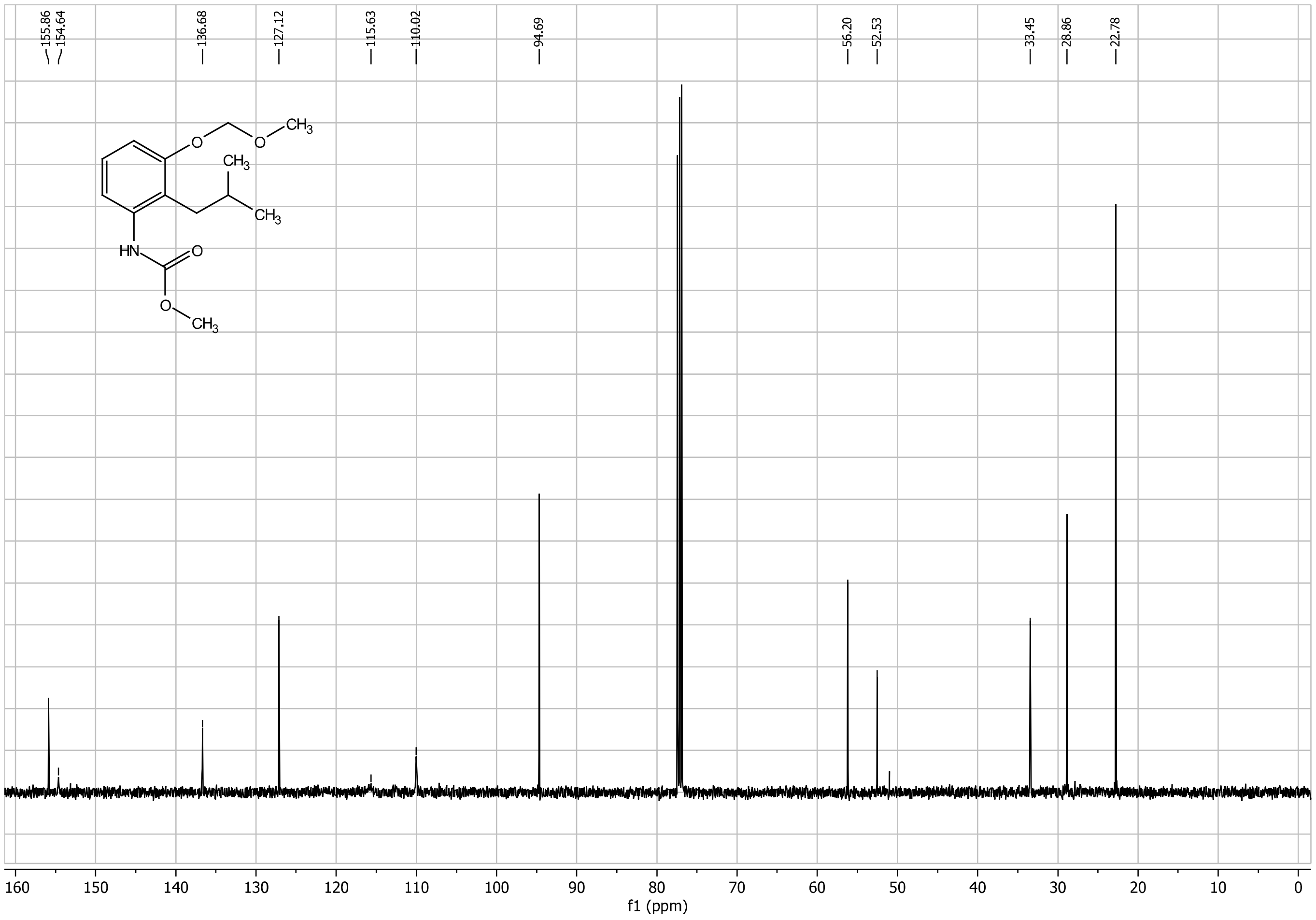
155.86
154.64
136.68
127.12
115.63
110.02
94.69
56.20
52.53
33.45
28.86
22.78
O
O
CH3
CH3
CH3
HN
O
O
CH3
f1 (ppm)

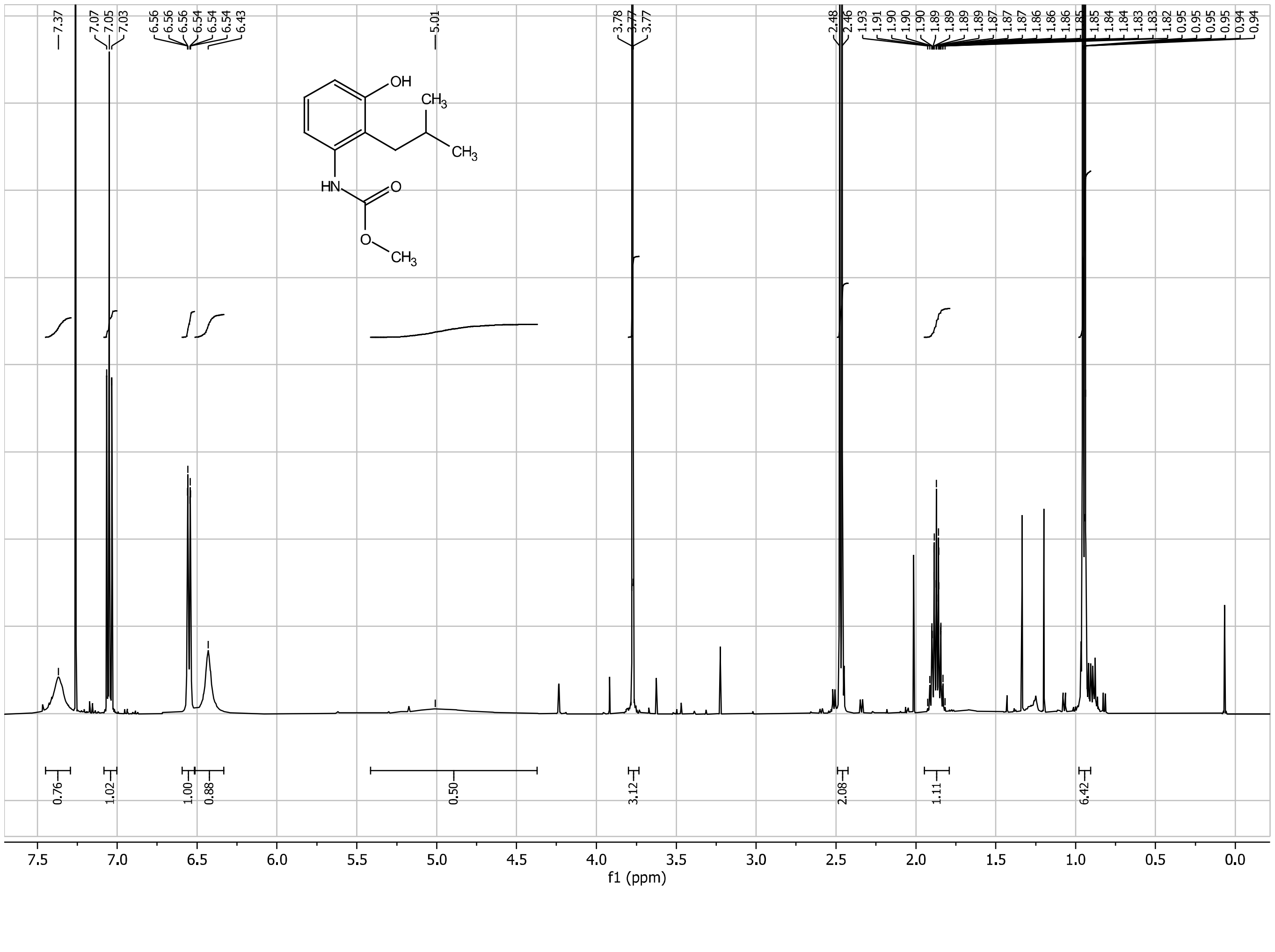
7.37
7.07
7.05
7.03
6.56
6.56
6.56
6.54
6.54
6.54
6.43
5.01
3.78
3.77
3.77
2.48
2.46
OH
CH3
CH3
HN
O
O
CH3
0.76
1.02
1.00
0.88
0.50
3.12
2.08
1.11
6.42
f1 (ppm)

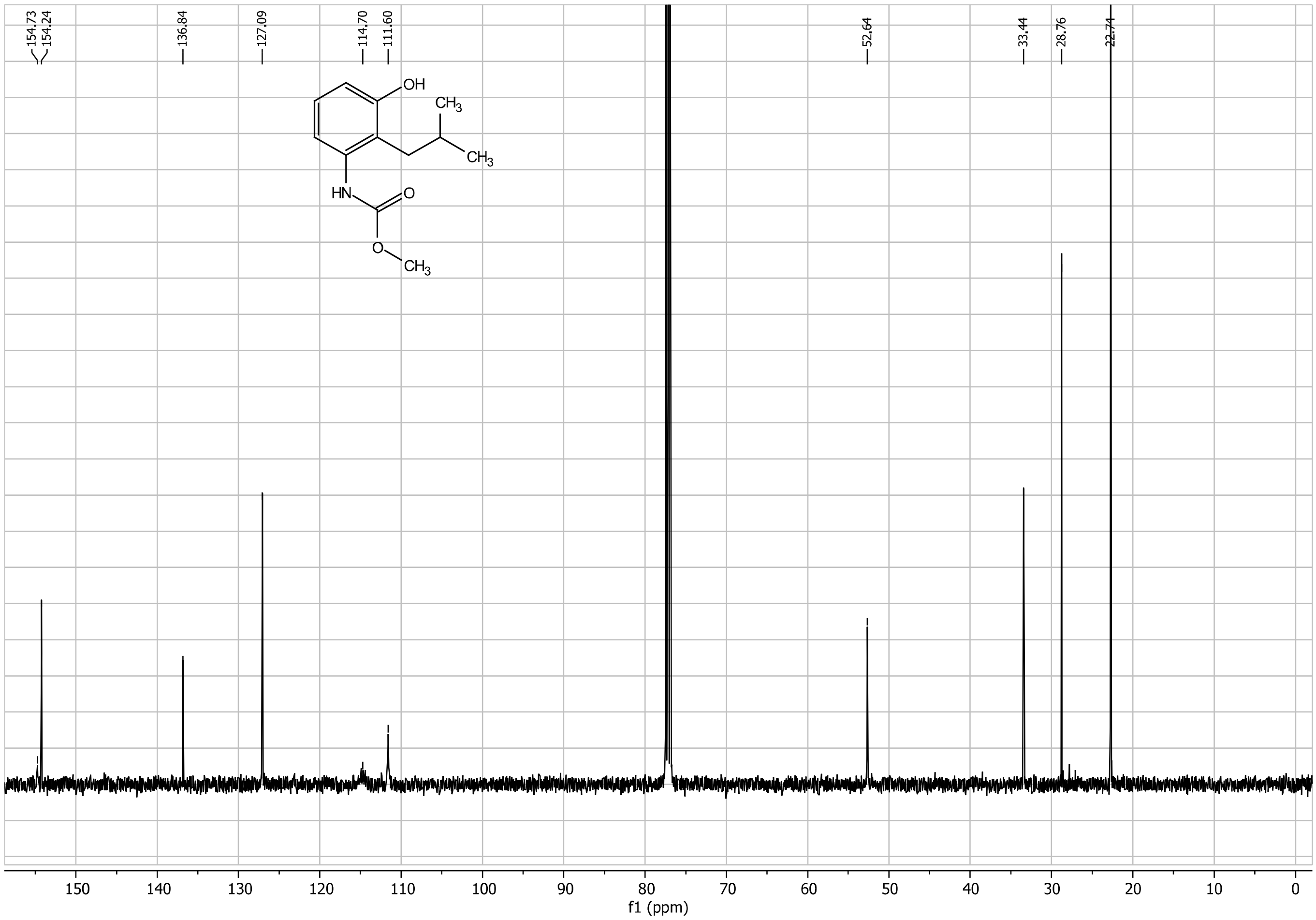
154.73
154.24
136.84
127.09
114.70
111.60
52.64
33.44
28.76
22.74
OH
CH3
CH3
HN
O
O
CH3
f1 (ppm)

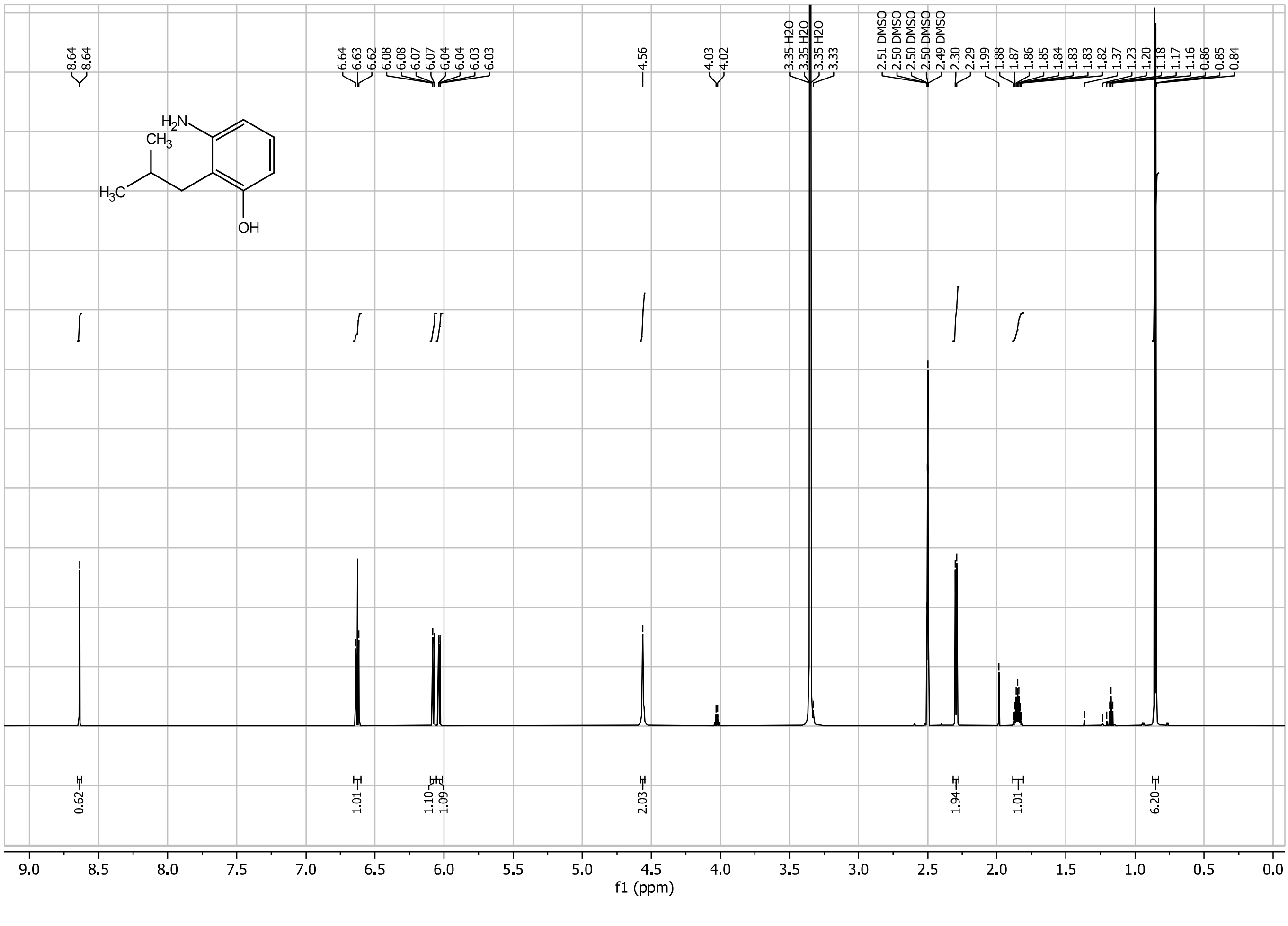
8.64
8.64
6.64
6.63
6.62
6.08
6.08
6.07
6.07
6.04
6.04
6.03
6.03
4.56
4.03
4.02
3.35 H2O
3.35 H2O
3.35 H2O
3.33
2.51 DMSO
2.50 DMSO
2.50 DMSO
2.50 DMSO
2.49 DMSO
2.30
2.29
1.99
1.88
1.87
1.86
1.85
1.84
1.83
1.83
1.82
1.37
1.23
1.20
1.18
1.17
1.16
0.86
0.85
0.84
H2N
CH3
H3C
OH
0.62
1.01
1.10
1.09
2.03
1.94
1.01
6.20
f1 (ppm)

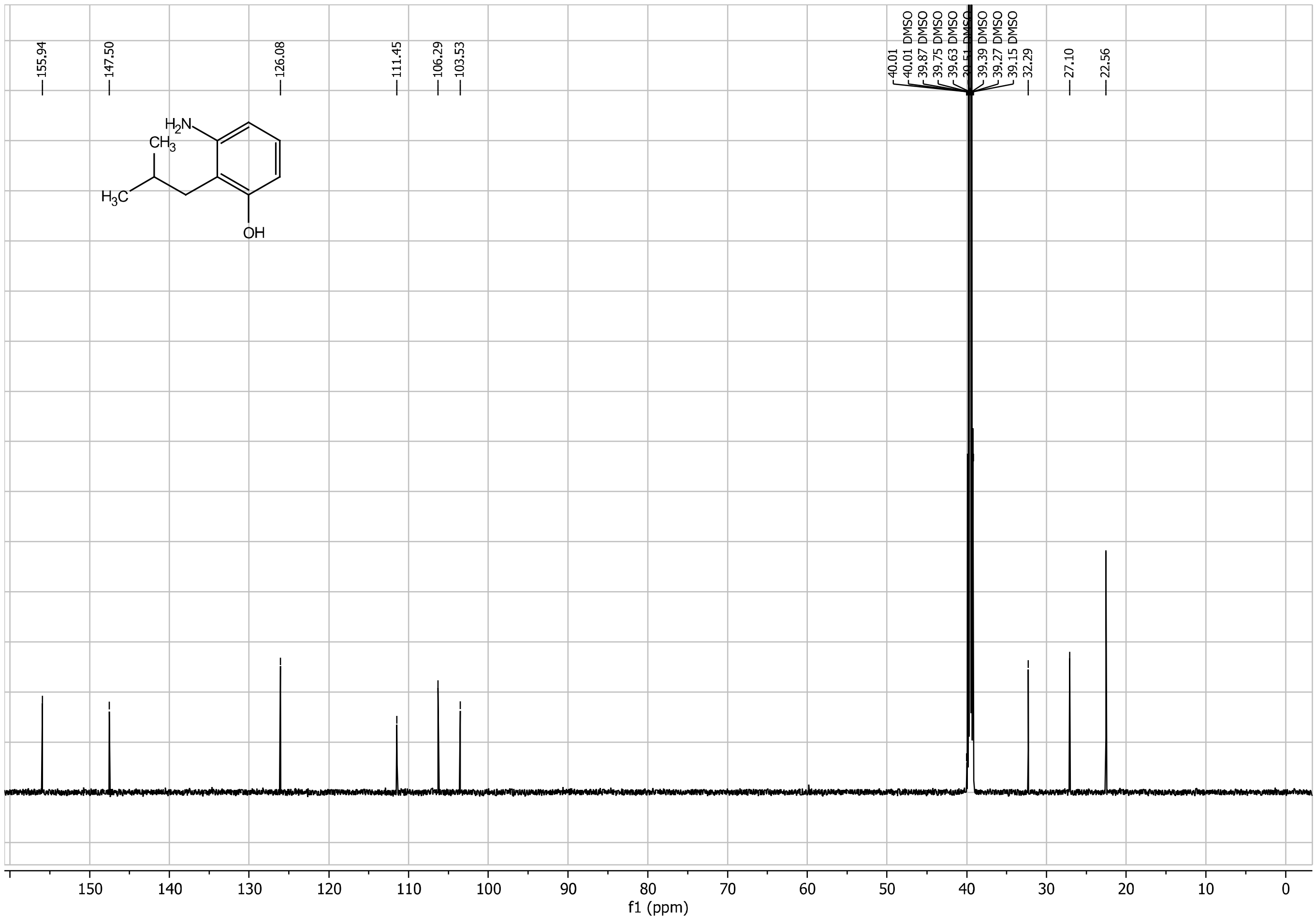
155.94
147.50
126.08
111.45
106.29
103.53
40.01
40.01 DMSO
39.87 DMSO
39.75 DMSO
39.63 DMSO
39.39 DMSO
39.27 DMSO
39.15 DMSO
32.29
27.10
22.56
H2N
CH3
H3C
OH
f1 (ppm)

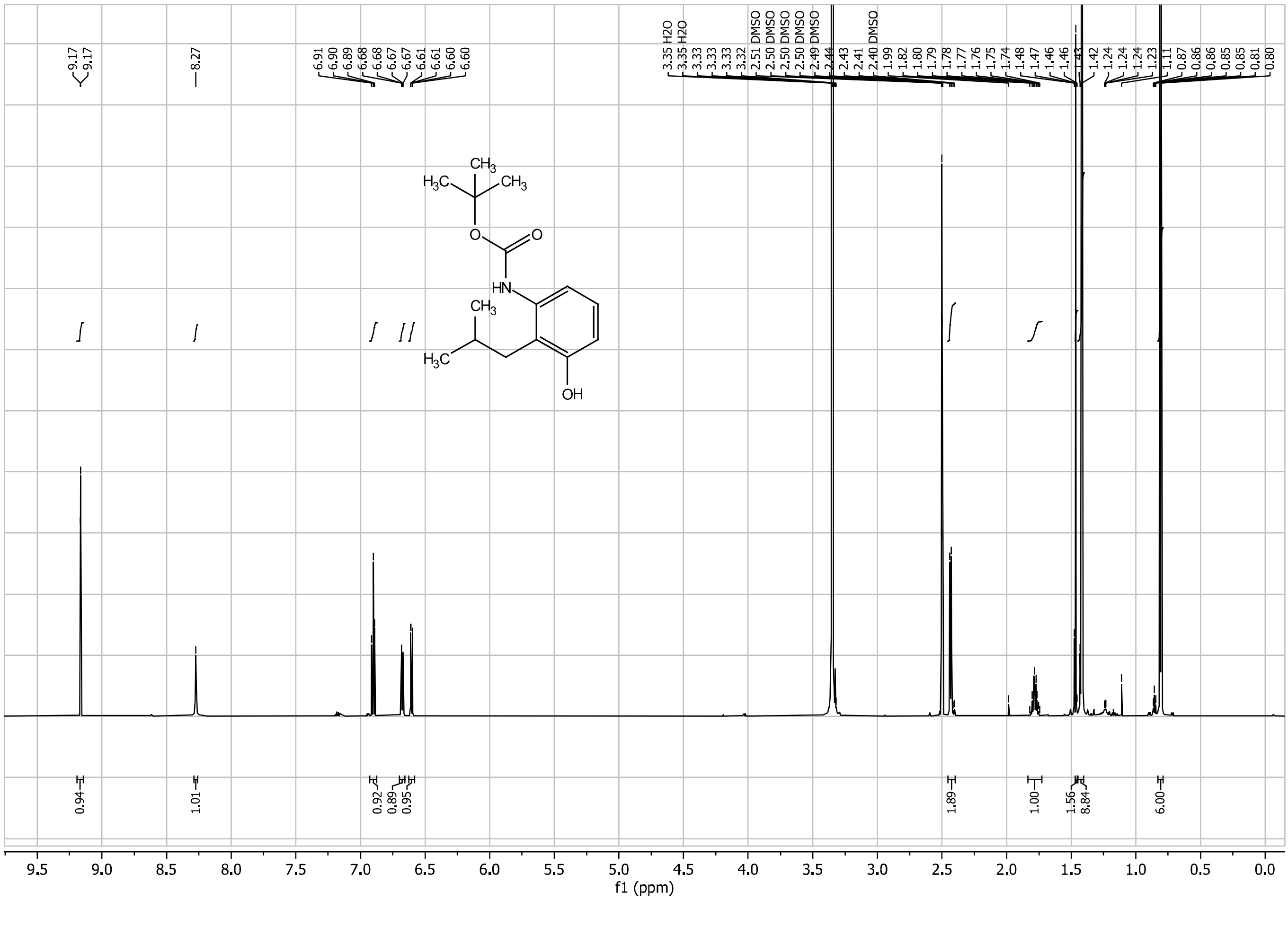
9.17
9.17
8.27
6.91
6.90
6.89
6.68
6.68
6.67
6.67
6.61
6.61
6.60
6.60
3.35 H2O
3.35 H2O
3.33
3.33
3.33
3.32
2.51 DMSO
2.50 DMSO
2.50 DMSO
2.50 DMSO
2.49 DMSO
2.44
2.43
2.41
2.40 DMSO
1.99
1.82
1.80
1.79
1.78
1.77
1.76
1.75
1.74
1.48
1.47
1.46
1.46
1.43
1.42
1.24
1.24
1.24
1.23
1.11
0.87
0.86
0.86
0.85
0.81
0.80
CH3
H3C
CH3
O
O
HN
CH3
H3C
OH
0.94
1.01
0.92
0.89
0.95
1.89
1.00
1.56
8.84
6.00
f1 (ppm)

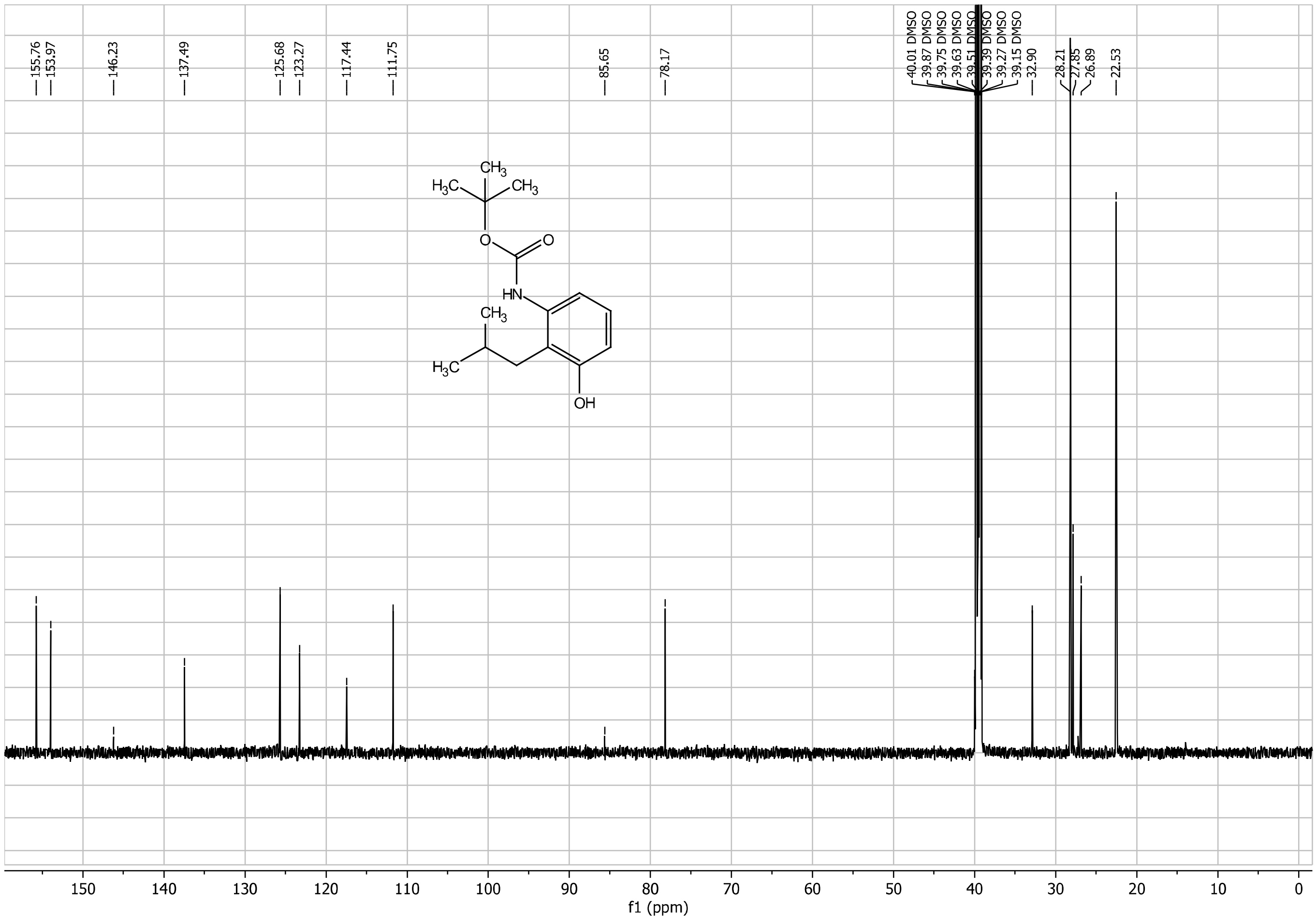
155.76
153.97
146.23
137.49
125.68
123.27
117.44
111.75
85.65
78.17
40.01 DMSO
39.87 DMSO
39.75 DMSO
39.63 DMSO
39.51 DMSO
39.39 DMSO
39.27 DMSO
39.15 DMSO
32.90
28.21
27.85
26.89
22.53
f1 (ppm)

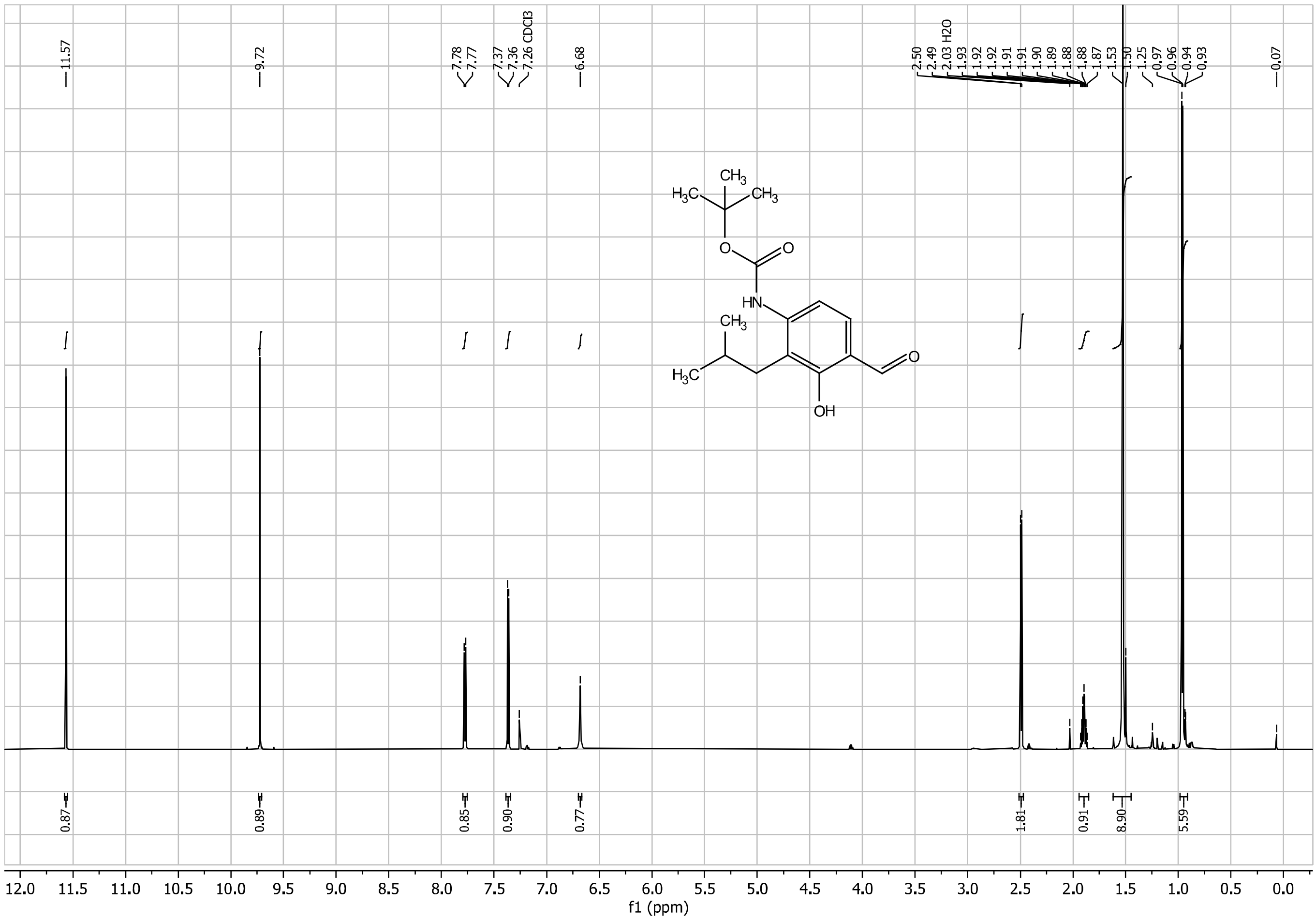
11.57
9.72
7.78
7.77
7.37
7.36
7.26 CDCl3
6.68
2.50
2.49
2.03 H2O
1.93
1.92
1.92
1.91
1.91
1.90
1.89
1.88
1.88
1.87
1.53
1.50
1.25
0.97
0.96
0.94
0.93
0.07
0.87
0.89
0.85
0.90
0.77
1.81
0.91
8.90
5.59
f1 (ppm)

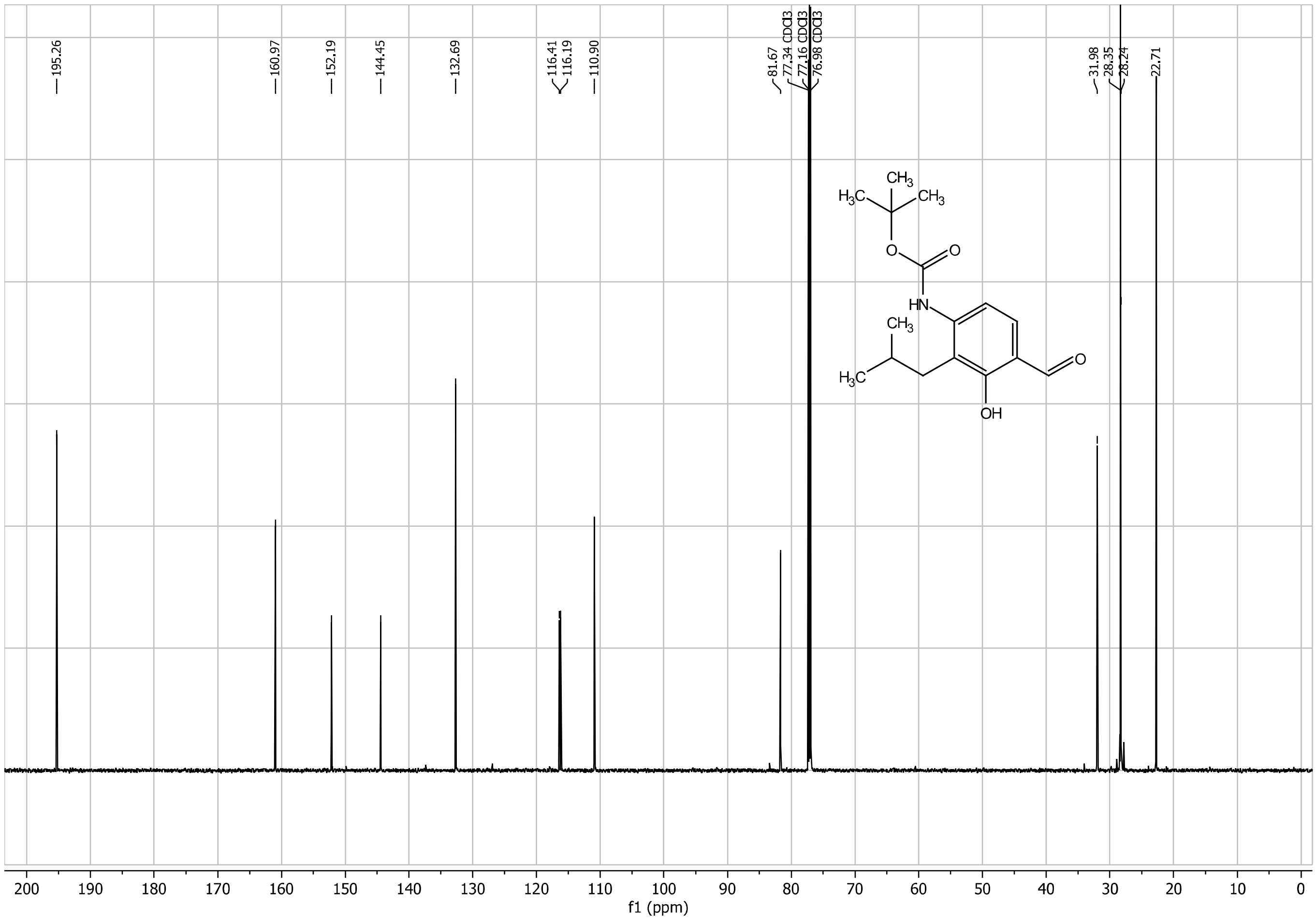

195.26
160.97
152.19
144.45
132.69
116.41
116.19
110.90
81.67
77.34 CDCl3
77.16 CDCl3
76.98 CDCl3
31.98
28.35
28.24
22.71
f1 (ppm)

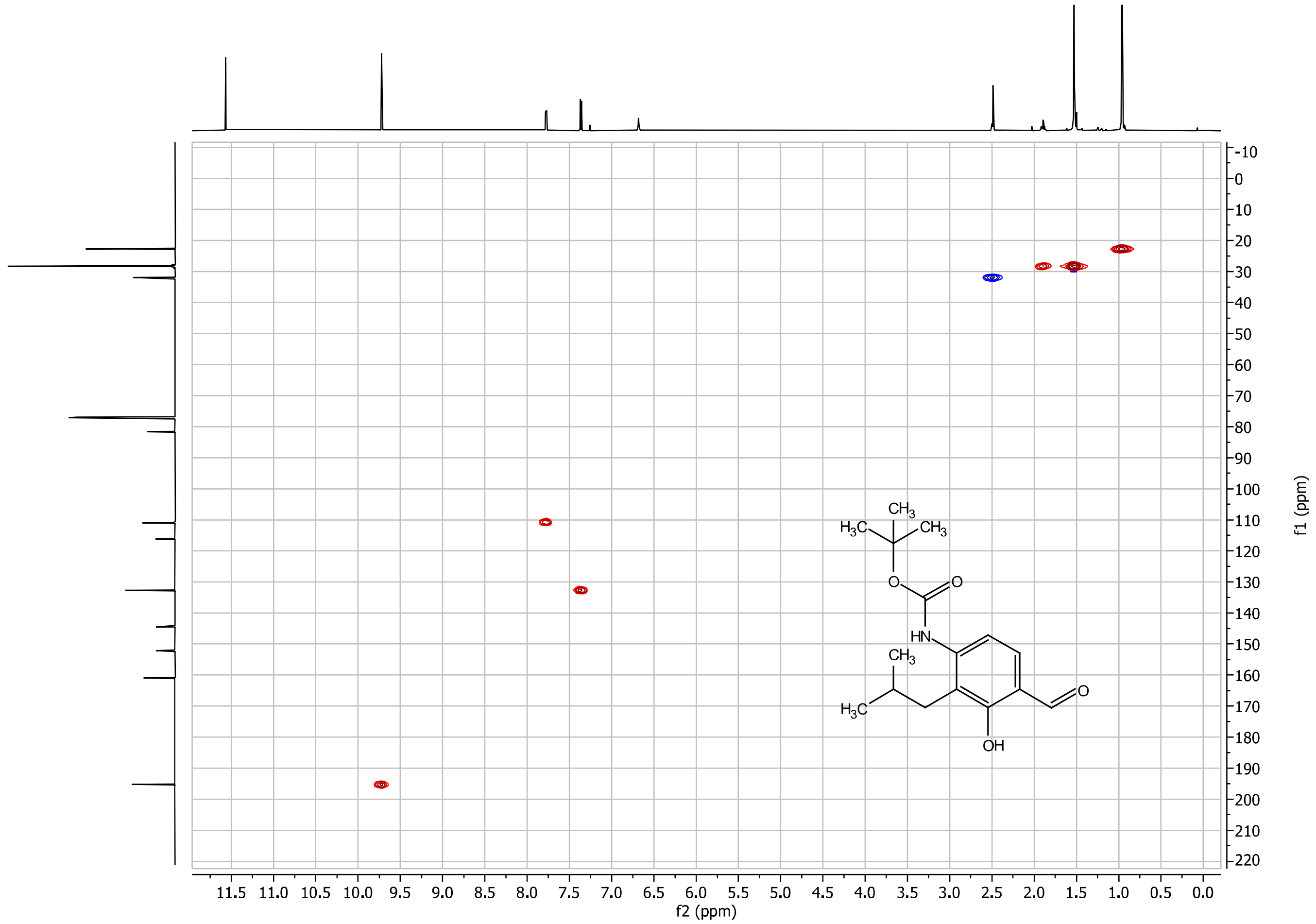

f2 (ppm)
f1 (ppm)

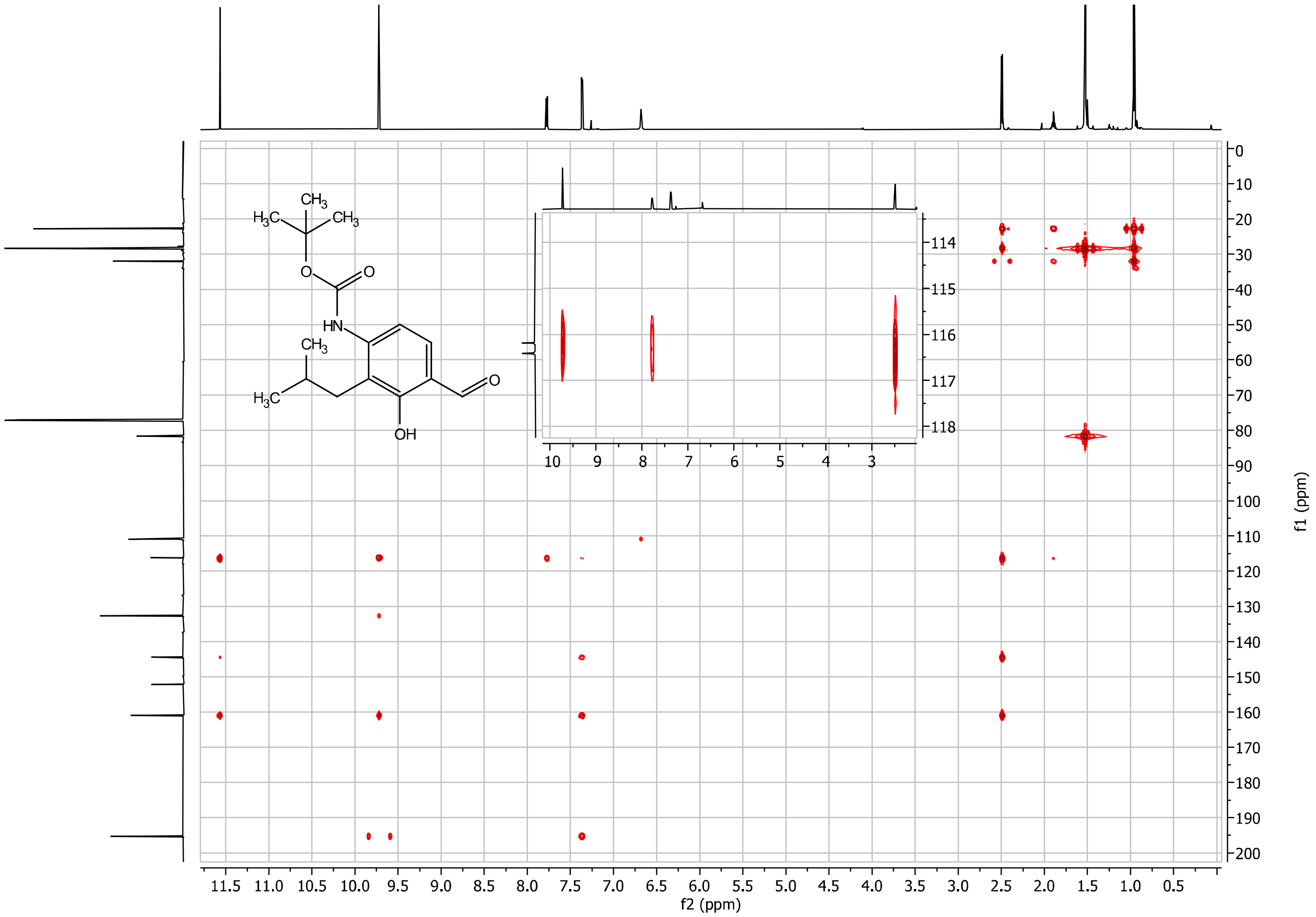

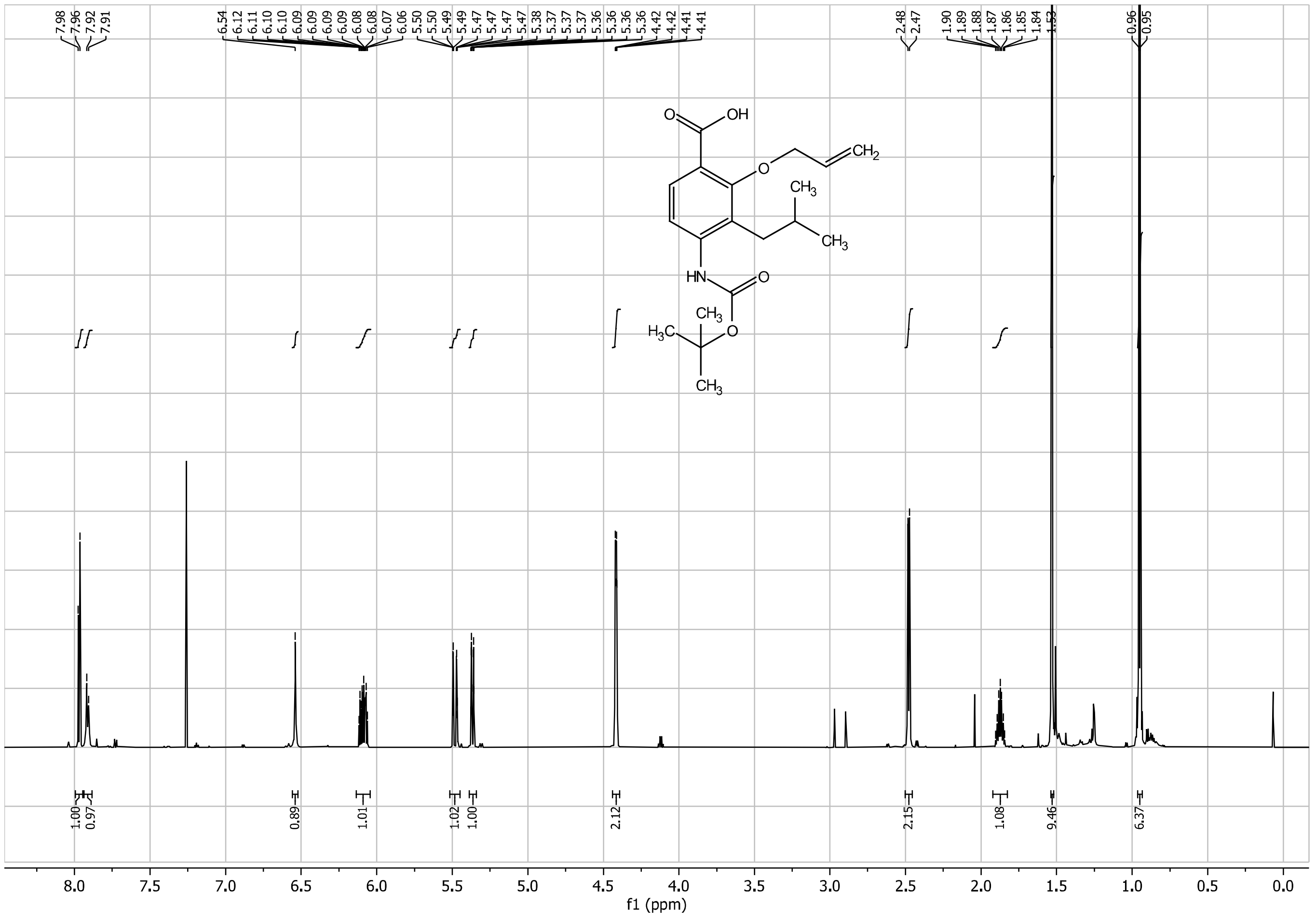

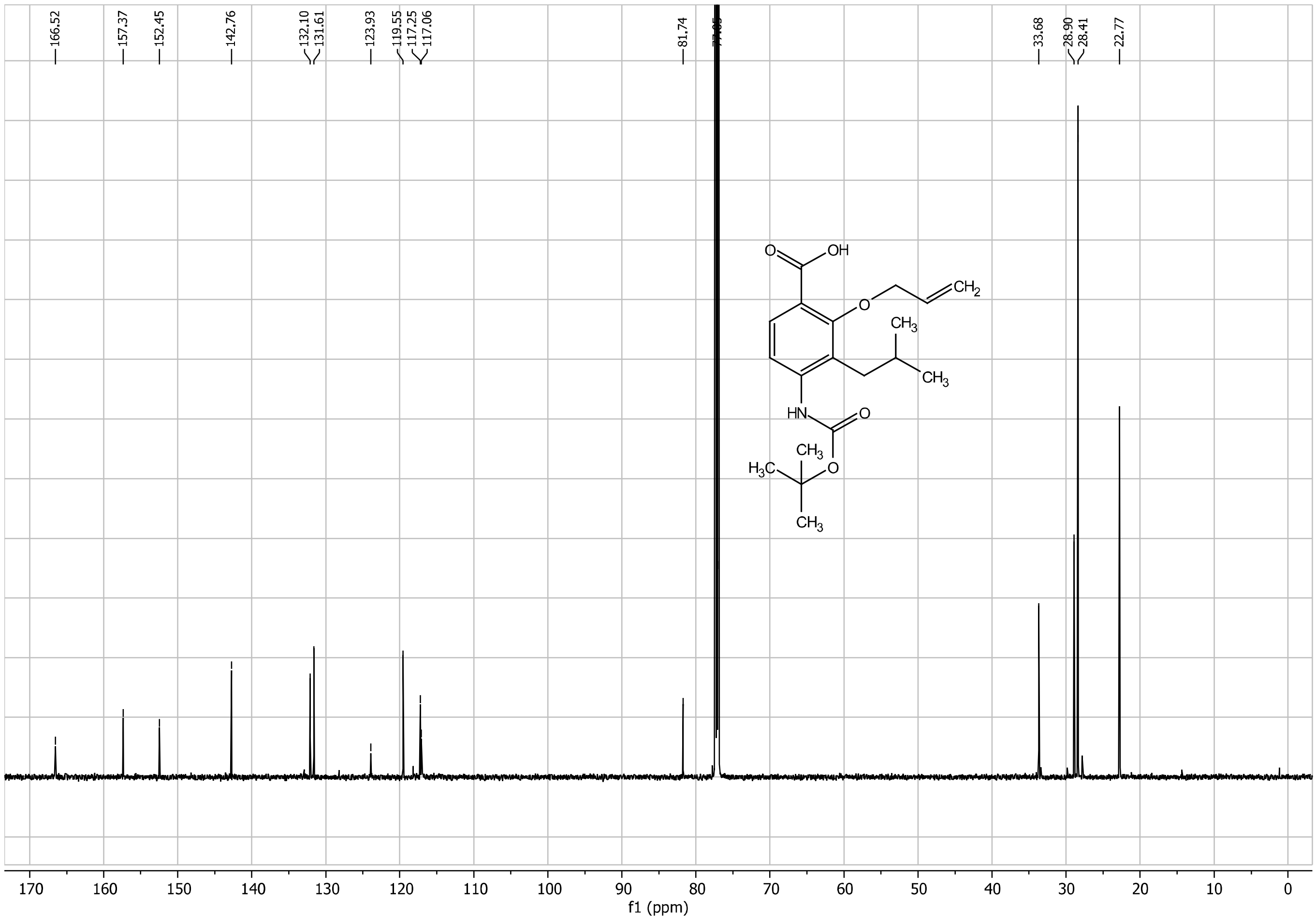
166.52
157.37
152.45
142.76
132.10
131.61
123.93
119.55
117.25
117.06
81.74
33.68
28.90
28.41
22.77
O
OH
O
CH2
CH3
CH3
HN
O
CH3
H3C
O
CH3
170
160
150
140
130
120
110
100
90
80
70
60
50
40
30
20
10
0
f1 (ppm)

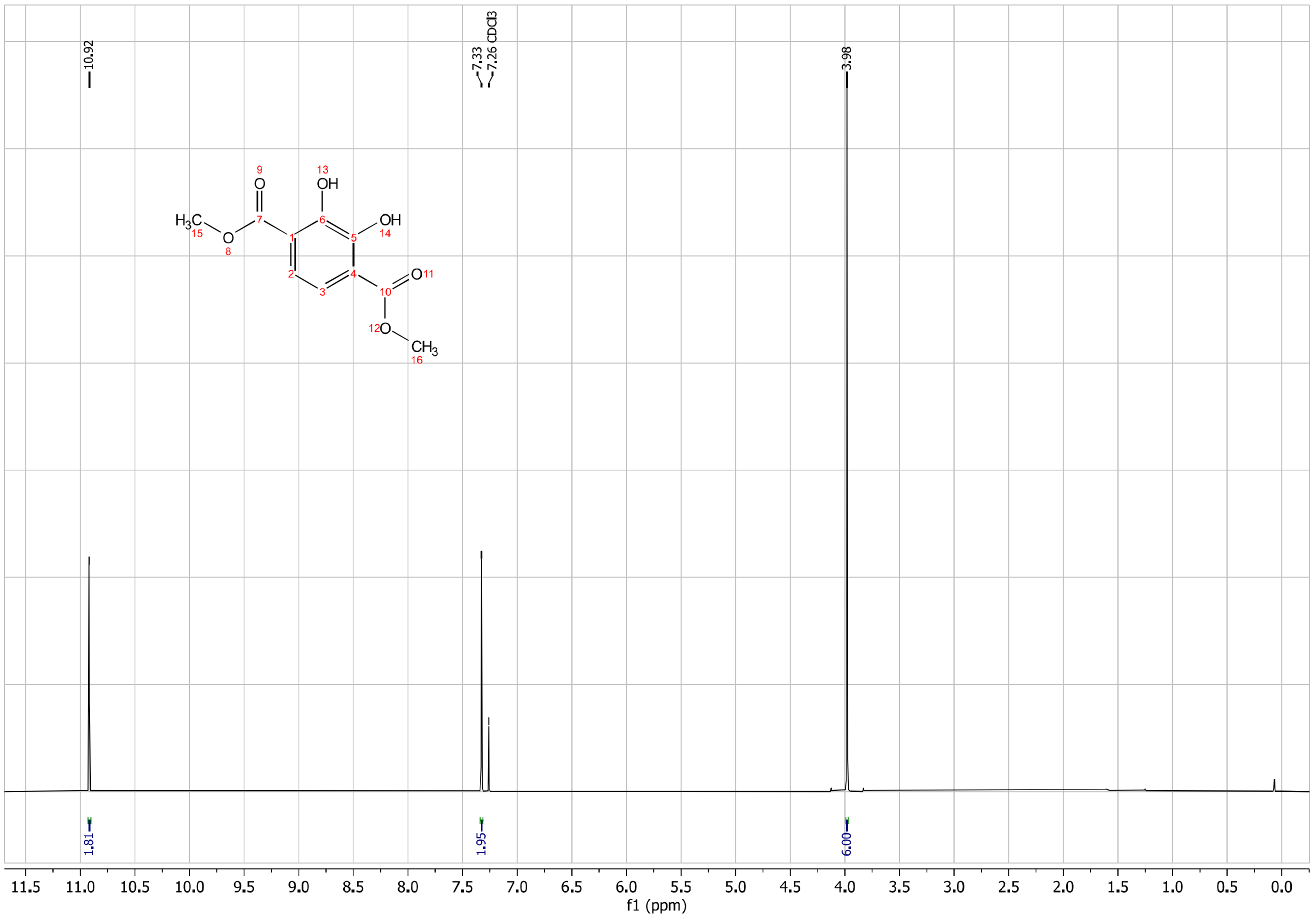
10.92
7.33
7.26 CDCl3
3.98
1.81
1.95
6.00
11.5 11.0 10.5 10.0 9.5 9.0 8.5 8.0 7.5 7.0 6.5 6.0 5.5 5.0 4.5 4.0 3.5 3.0 2.5 2.0 1.5 1.0 0.5 0.0
f1 (ppm)

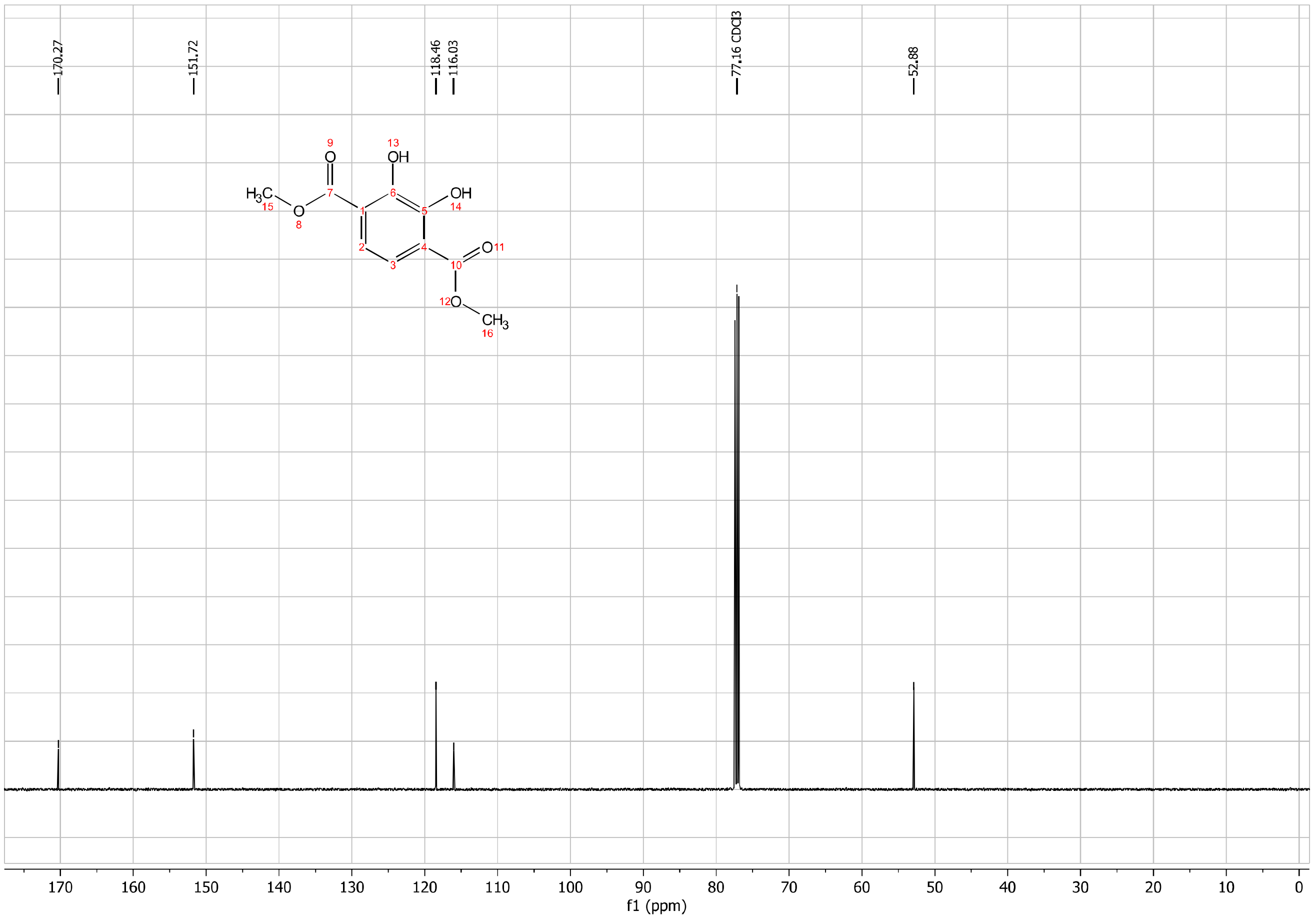
170.27
151.72
118.46
116.03
77.16 CDCl3
52.88
170 160 150 140 130 120 110 100 90 80 70 60 50 40 30 20 10 0
f1 (ppm)

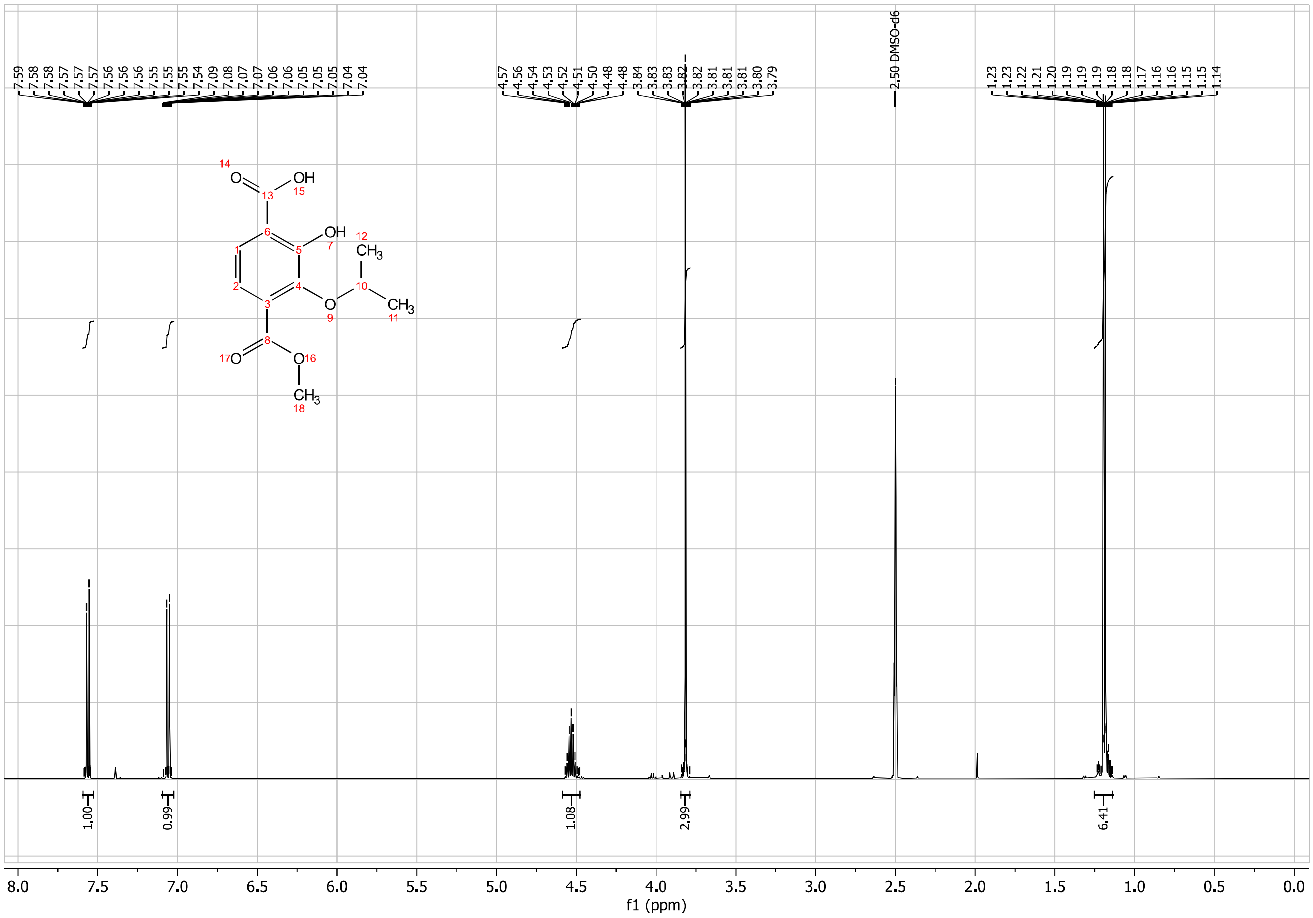
7.59
7.58
7.58
7.57
7.57
7.57
7.56
7.56
7.56
7.55
7.55
7.55
7.54
7.09
7.08
7.07
7.07
7.06
7.06
7.05
7.05
7.05
7.04
7.04
4.57
4.56
4.54
4.53
4.52
4.51
4.50
4.48
4.48
3.84
3.83
3.83
3.82
3.82
3.81
3.81
3.81
3.80
3.79
2.50 DMSO-d6
1.23
1.23
1.22
1.21
1.20
1.19
1.19
1.19
1.18
1.18
1.17
1.16
1.15
1.15
1.14
1.00
0.99
1.08
2.99
6.41
f1 (ppm)

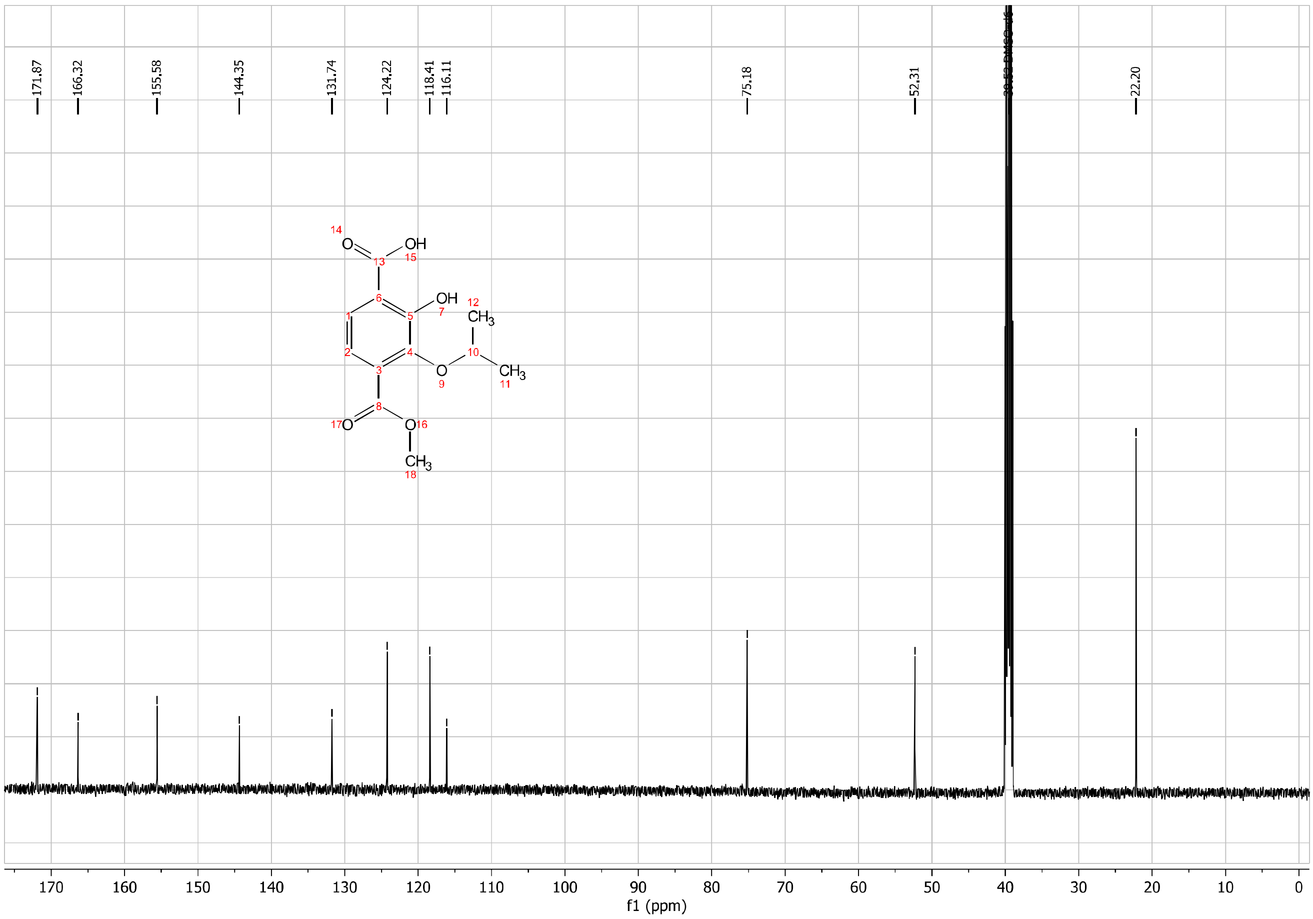
171.87
166.32
155.58
144.35
131.74
124.22
118.41
116.11
75.18
52.31
22.20
f1 (ppm)

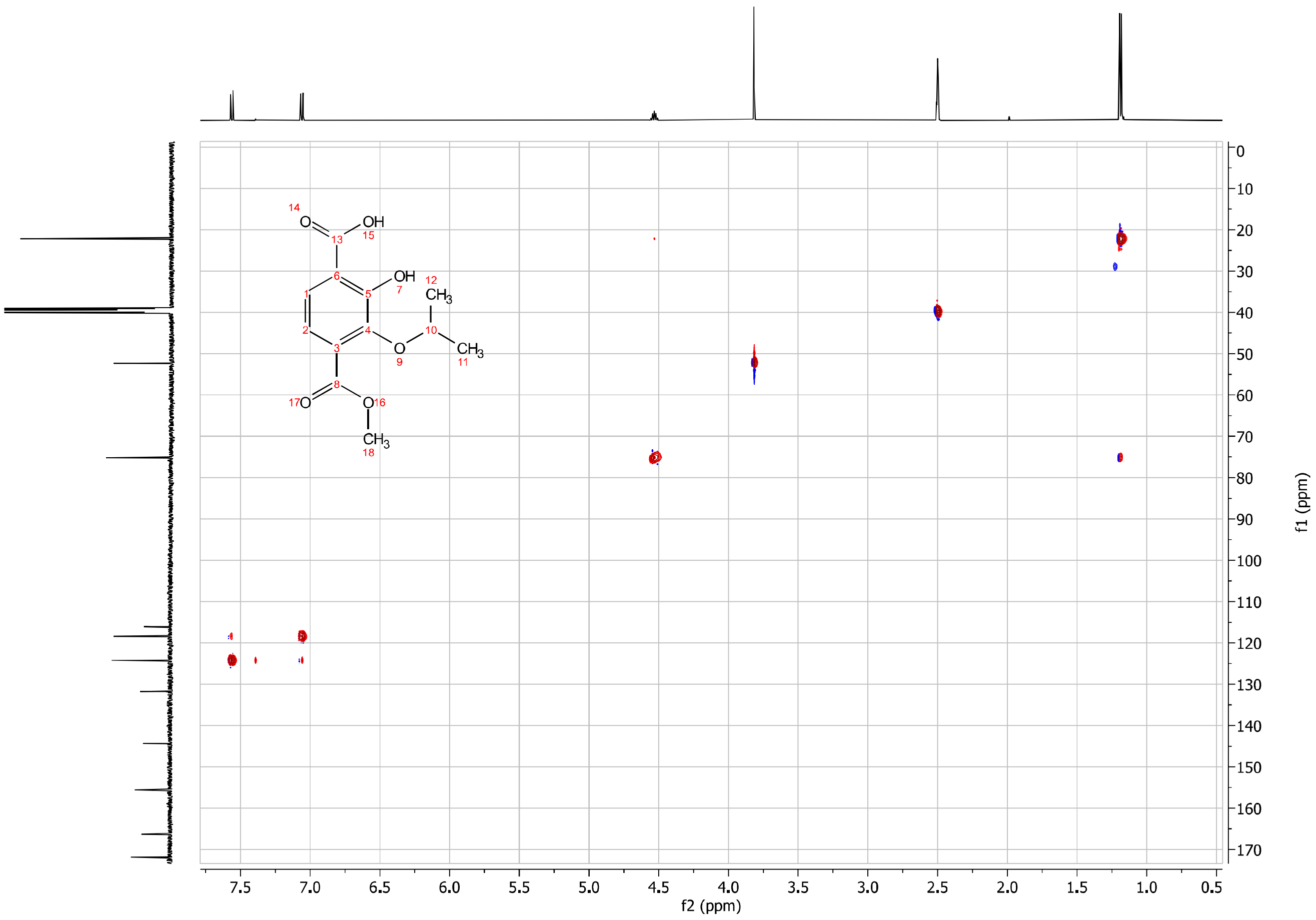
14
O
OH
15
13
6
OH
7
12
CH3
1
5
2
4
10
3
O
9
CH3
11
8
17O
O16
CH3
18
f1 (ppm)
f2 (ppm)

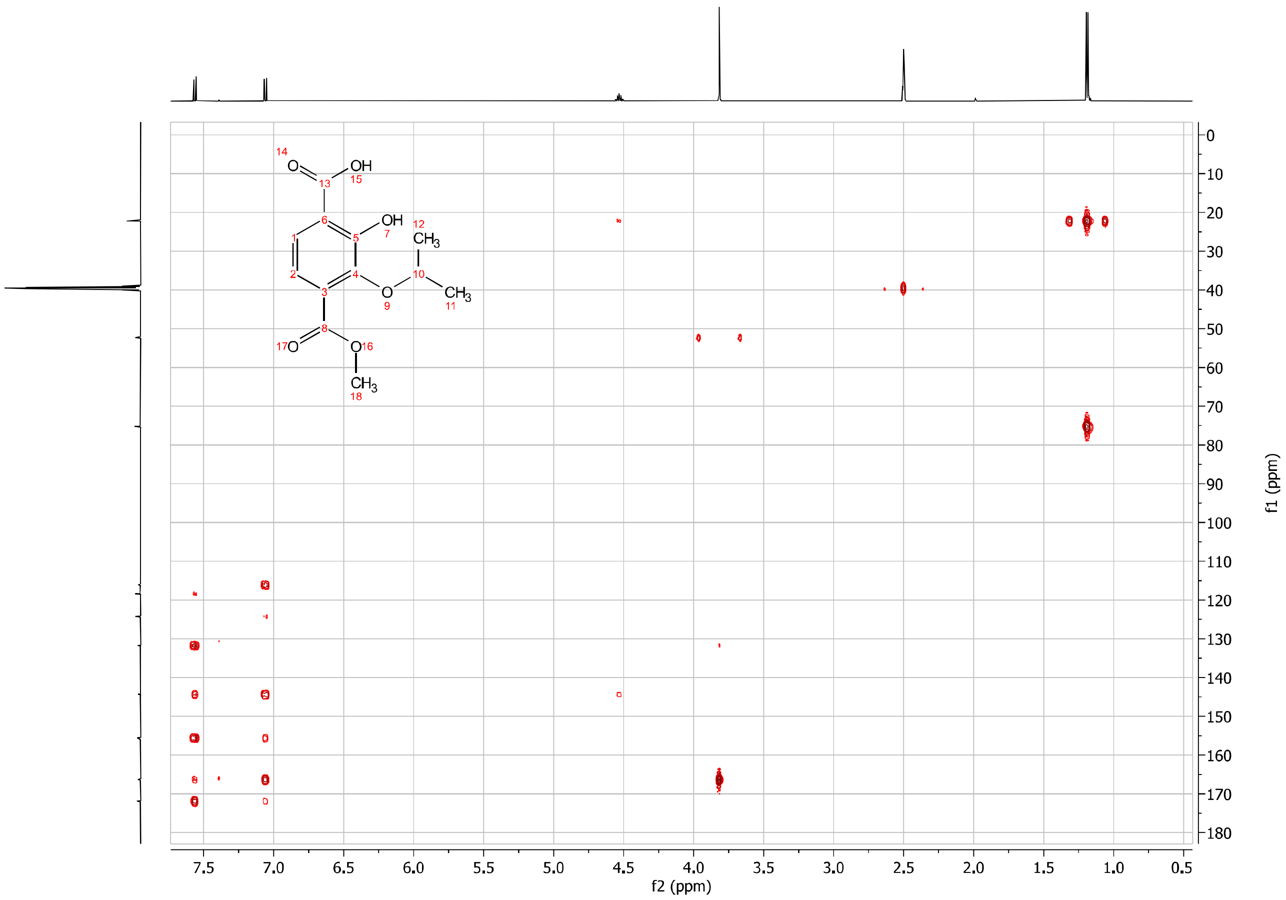
14
O
OH
15
13
6
OH
7
12
CH3
1
5
2
4
10
3
O
9
CH3
11
8
17O
O16
CH3
18
f1 (ppm)
f2 (ppm)

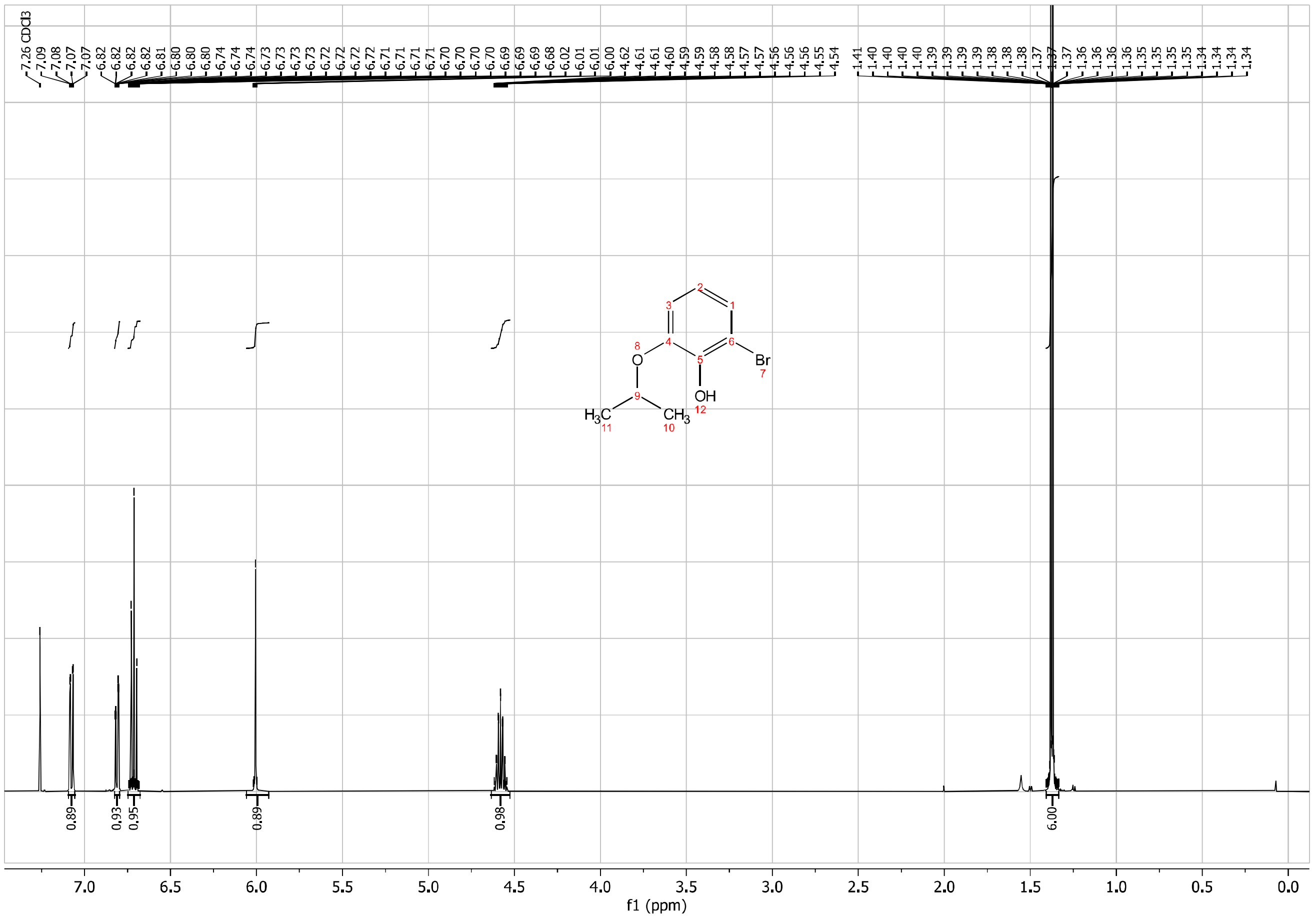
7.26 CDCl3
f1 (ppm)
Br
OH
H3C
CH3

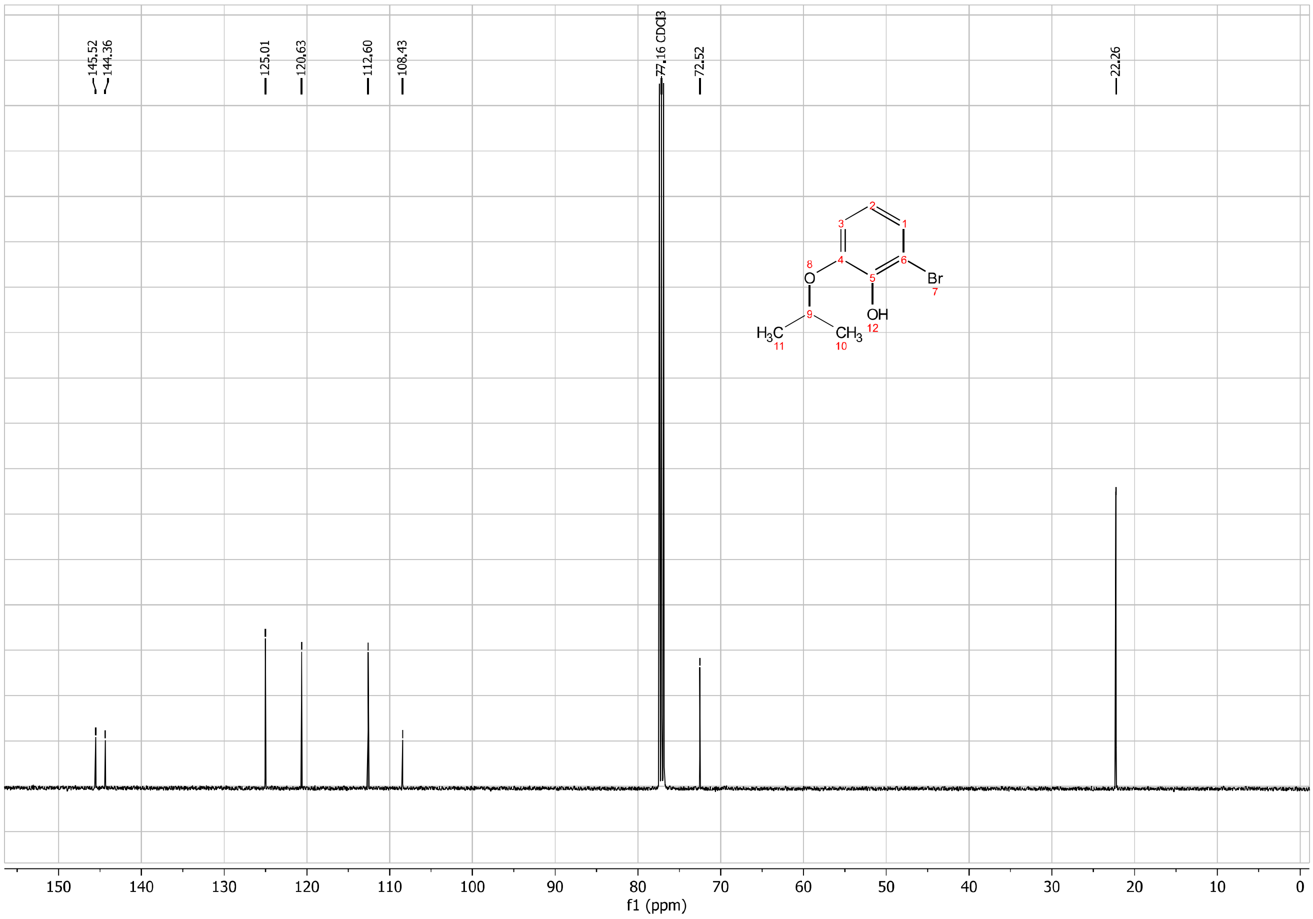
145.52
144.36
125.01
120.63
112.60
108.43
77.16 CDCl3
72.52
22.26
f1 (ppm)
Br
OH
H3C
CH3

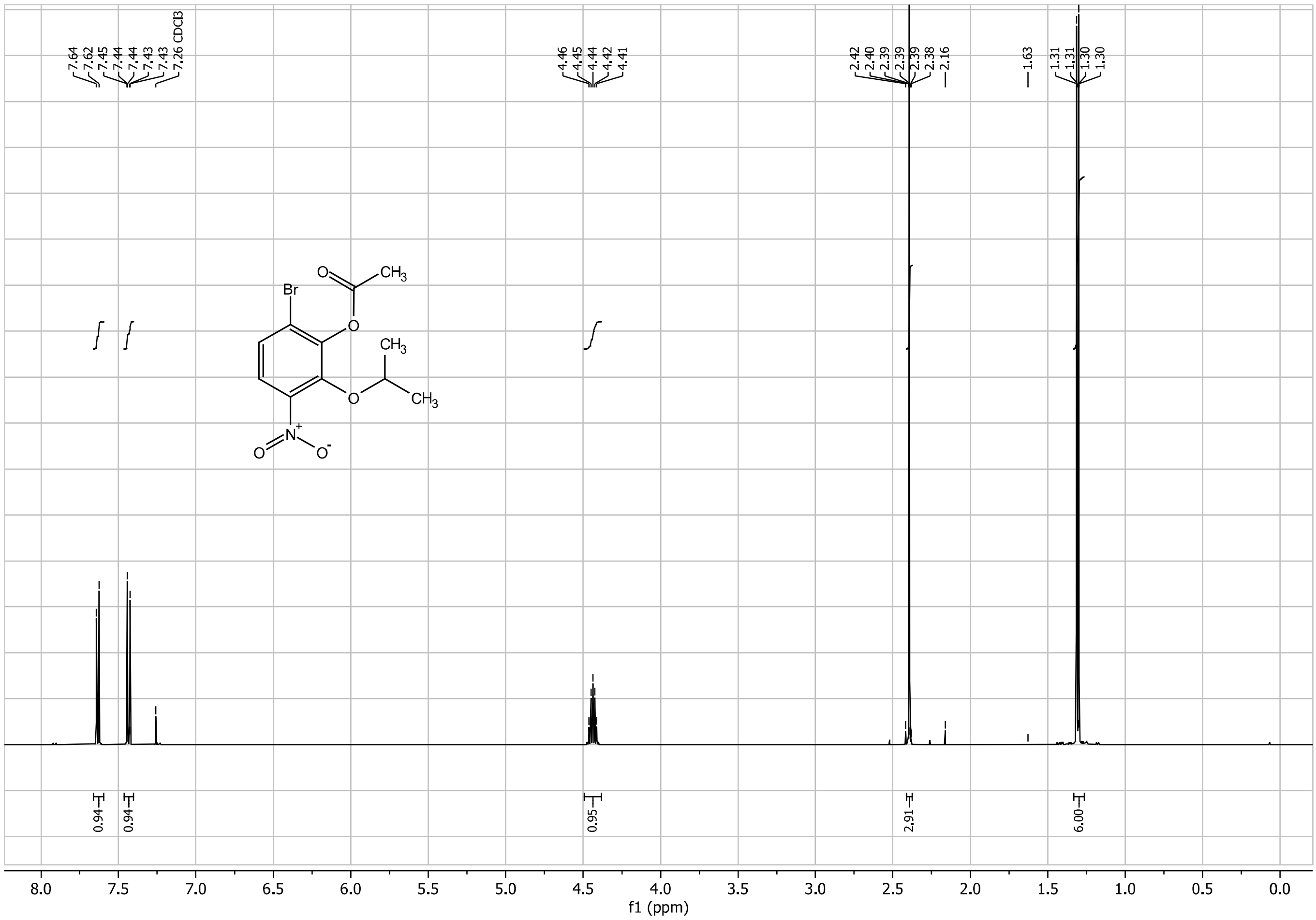
7.64
7.62
7.45
7.44
7.44
7.43
7.43
7.26 CDCl3
4.46
4.45
4.44
4.42
4.41
2.42
2.40
2.39
2.39
2.39
2.38
2.16
1.63
1.31
1.31
1.30
1.30
Br
O
CH3
O
CH3
O
CH3
N+
O
O-
0.94
0.94
0.95
2.91
6.00
f1 (ppm)

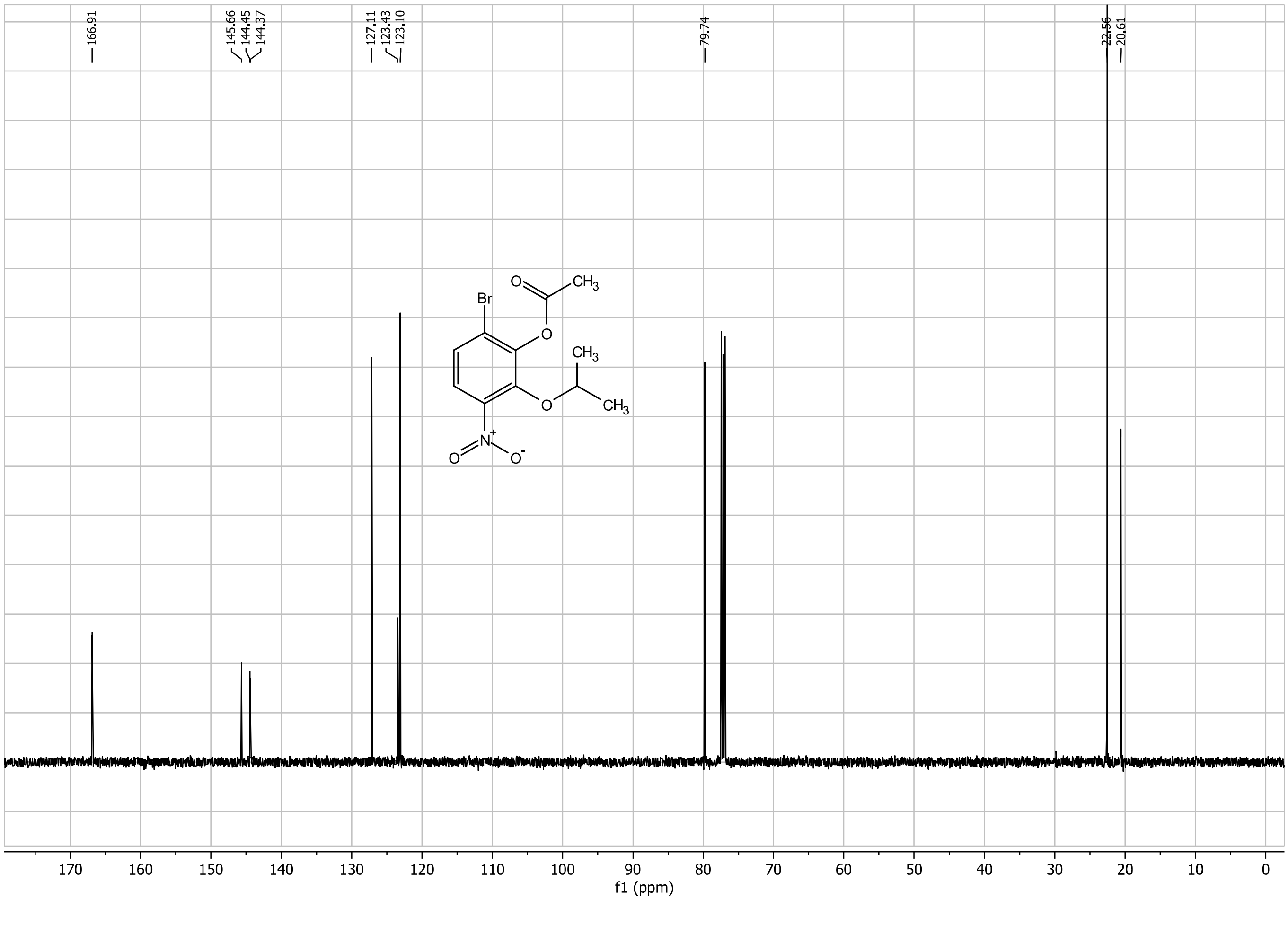
166.91
145.66
144.45
144.37
127.11
123.43
123.10
79.74
22.56
20.61
Br
O
CH3
O
CH3
O
CH3
N+
O
O-
f1 (ppm)

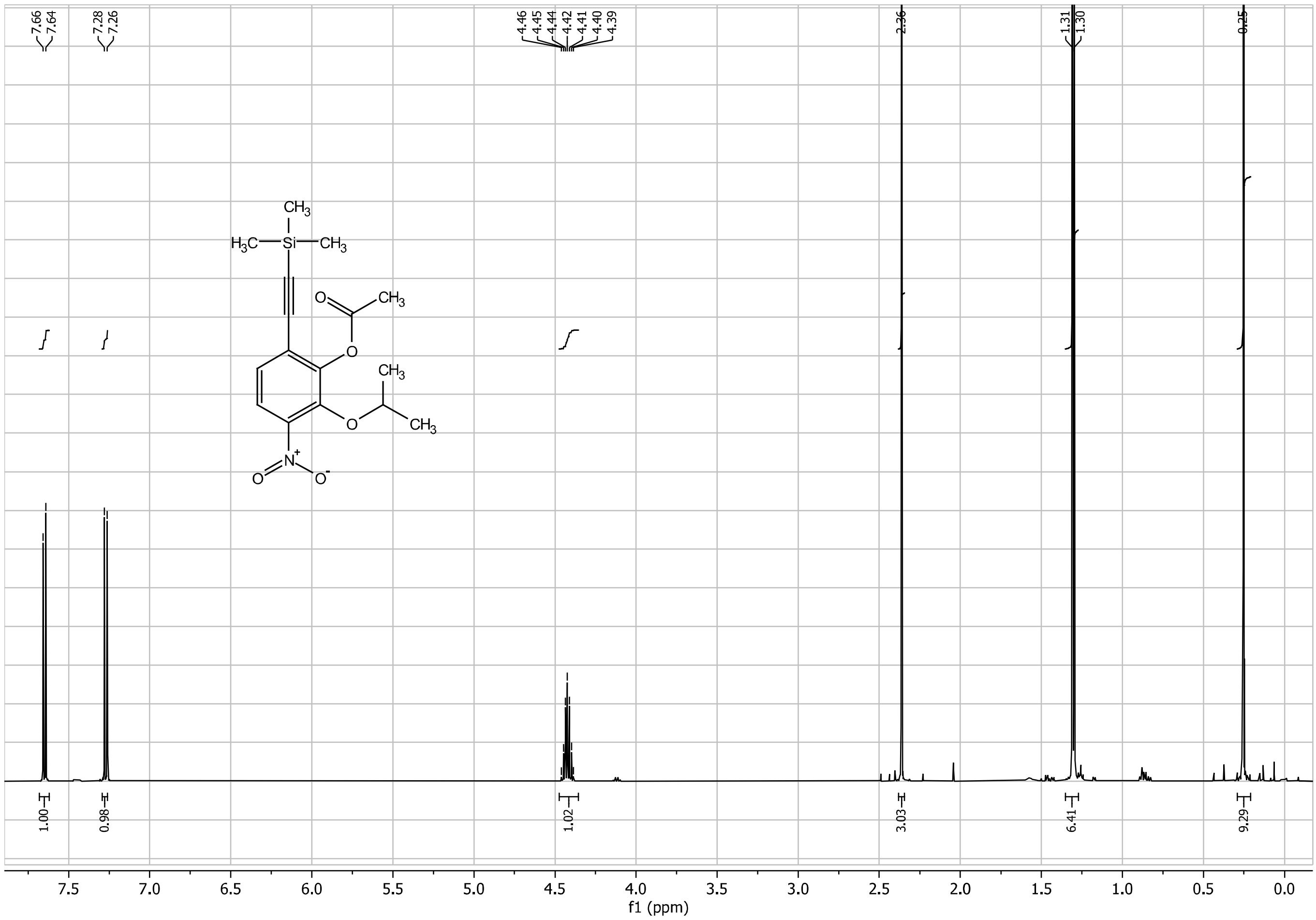
7.66
7.64
7.28
7.26
4.46
4.45
4.44
4.42
4.41
4.40
4.39
2.36
1.31
1.30
0.25
1.00
0.98
1.02
3.03
6.41
9.29
7.5 7.0 6.5 6.0 5.5 5.0 4.5 4.0 3.5 3.0 2.5 2.0 1.5 1.0 0.5 0.0
f1 (ppm)

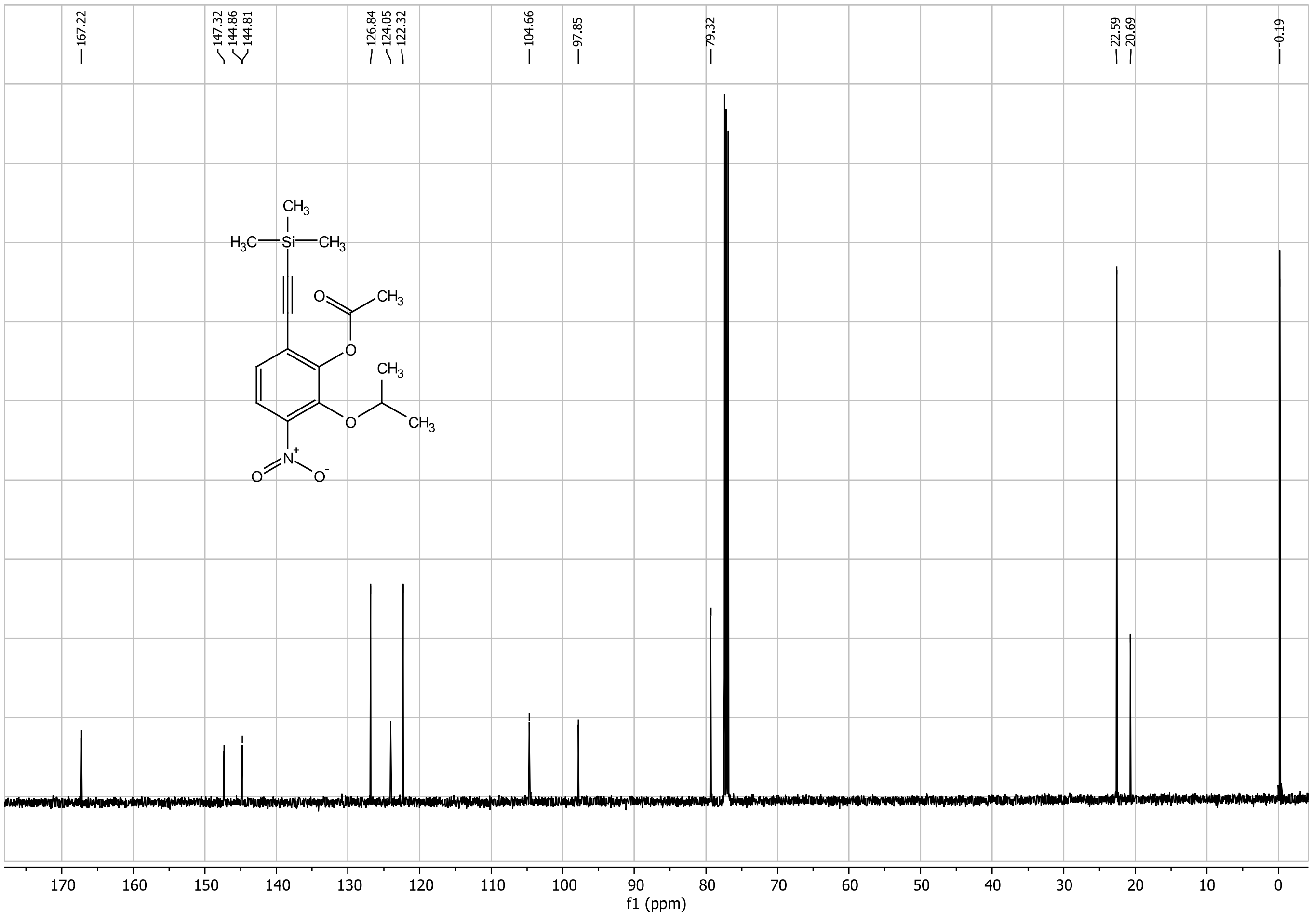
167.22
147.32
144.86
144.81
126.84
124.05
122.32
104.66
97.85
79.32
22.59
20.69
-0.19
170 160 150 140 130 120 110 100 90 80 70 60 50 40 30 20 10 0
f1 (ppm)

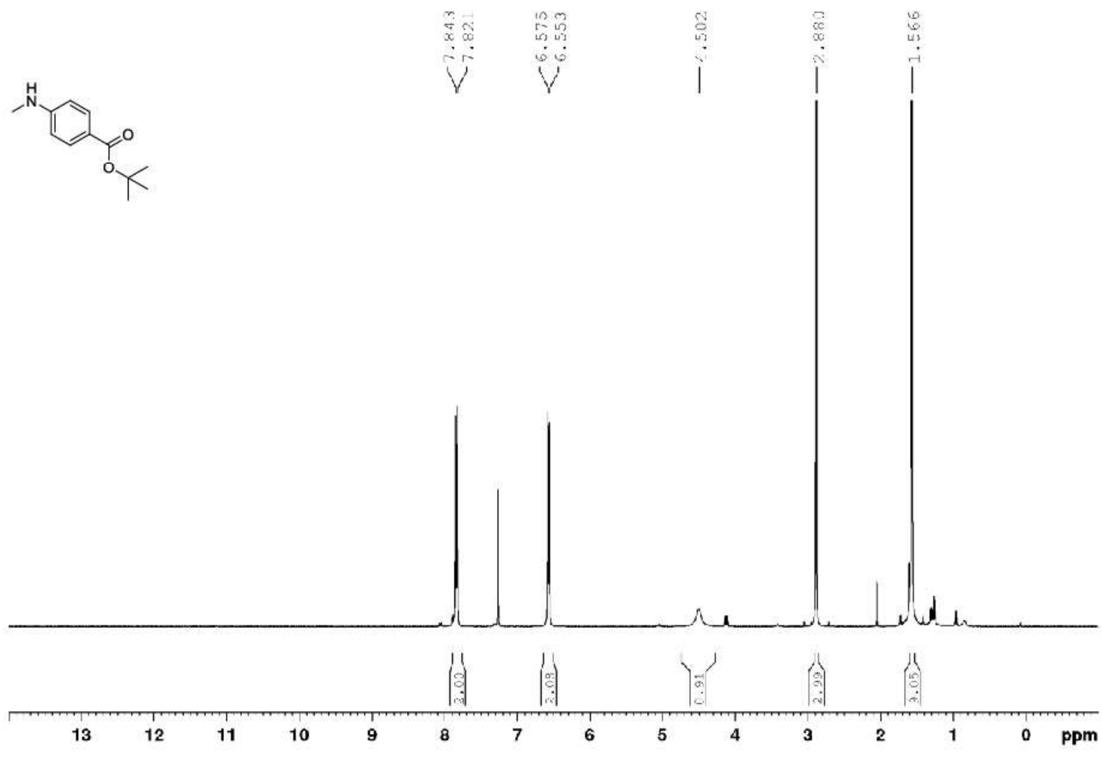
7.843
7.821
6.575
6.553
2.880
1.566
13 12 11 10 9 8 7 6 5 4 3 2 1 0 ppm

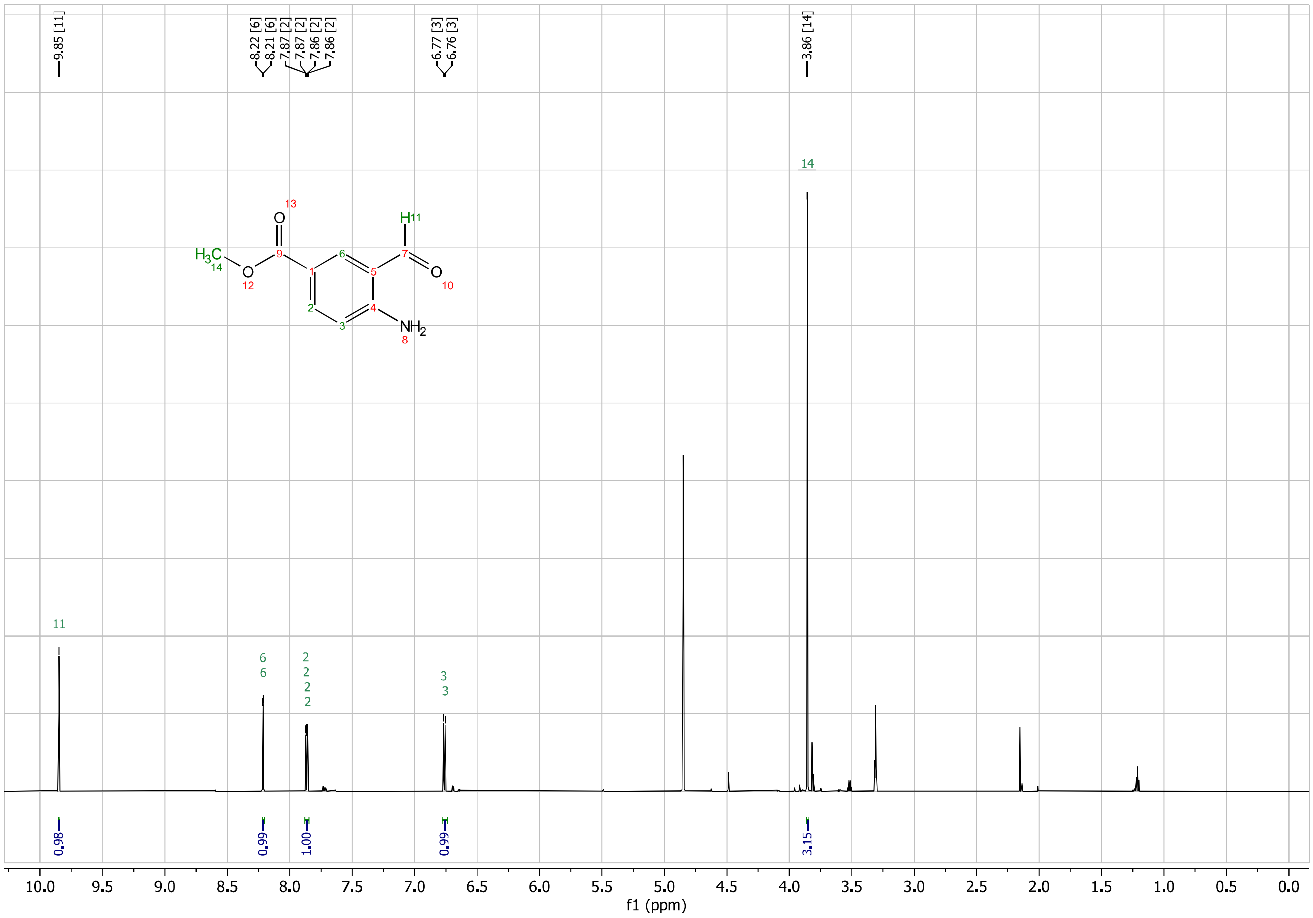
9.85 [11]
8.22 [6]
8.21 [6]
7.87 [2]
7.87 [2]
7.86 [2]
7.86 [2]
6.77 [3]
6.76 [3]
3.86 [14]
0.98
0.99
1.00
0.99
3.15
f1 (ppm)

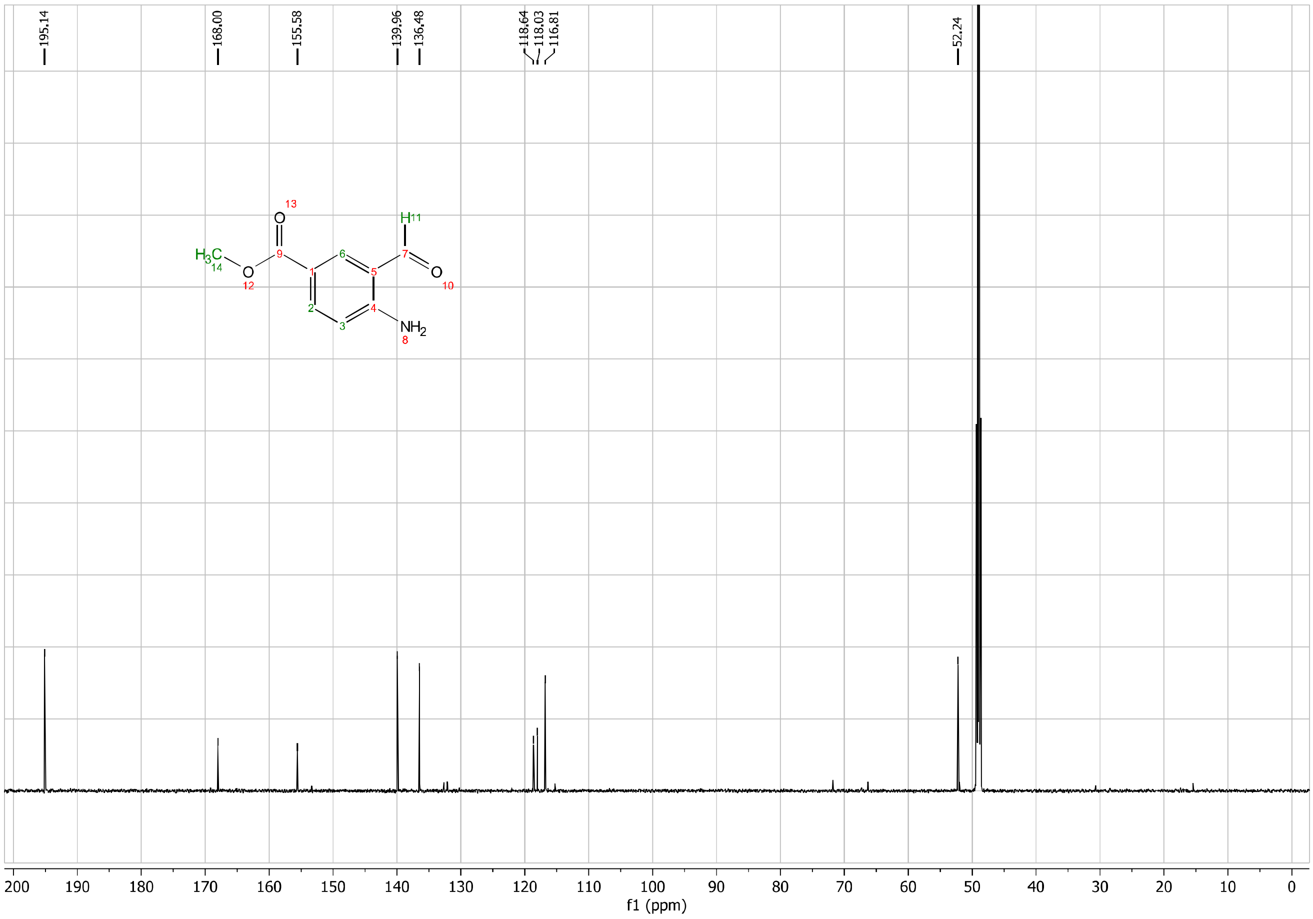
195.14
168.00
155.58
139.96
136.48
118.64
118.03
116.81
52.24
f1 (ppm)

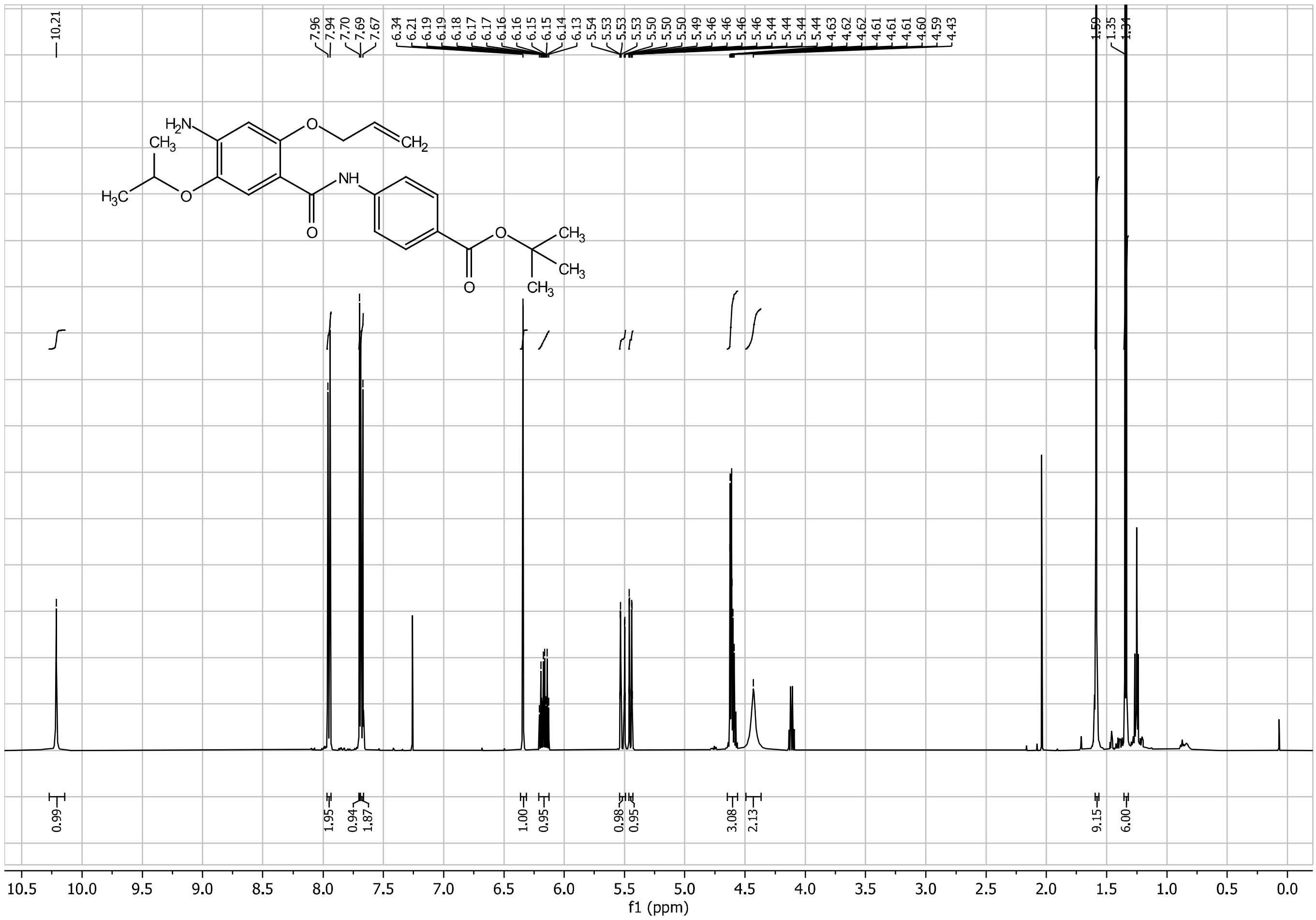
10.21
7.96
7.94
7.70
7.69
7.67
6.34
6.21
6.19
6.19
6.18
6.17
6.17
6.16
6.16
6.15
6.15
6.14
6.13
5.54
5.53
5.53
5.53
5.50
5.50
5.49
5.46
5.46
5.46
5.46
5.44
5.44
5.44
5.44
4.63
4.62
4.62
4.61
4.61
4.60
4.59
4.43
1.59
1.35
1.34
H2N
CH3
H3C
O
O
NH
CH2
O
O
CH3
CH3
CH3
0.99
1.95
0.94
1.87
1.00
0.95
0.98
0.95
3.08
2.13
9.15
6.00
10.5 10.0 9.5 9.0 8.5 8.0 7.5 7.0 6.5 6.0 5.5 5.0 4.5 4.0 3.5 3.0 2.5 2.0 1.5 1.0 0.5 0.0
f1 (ppm)

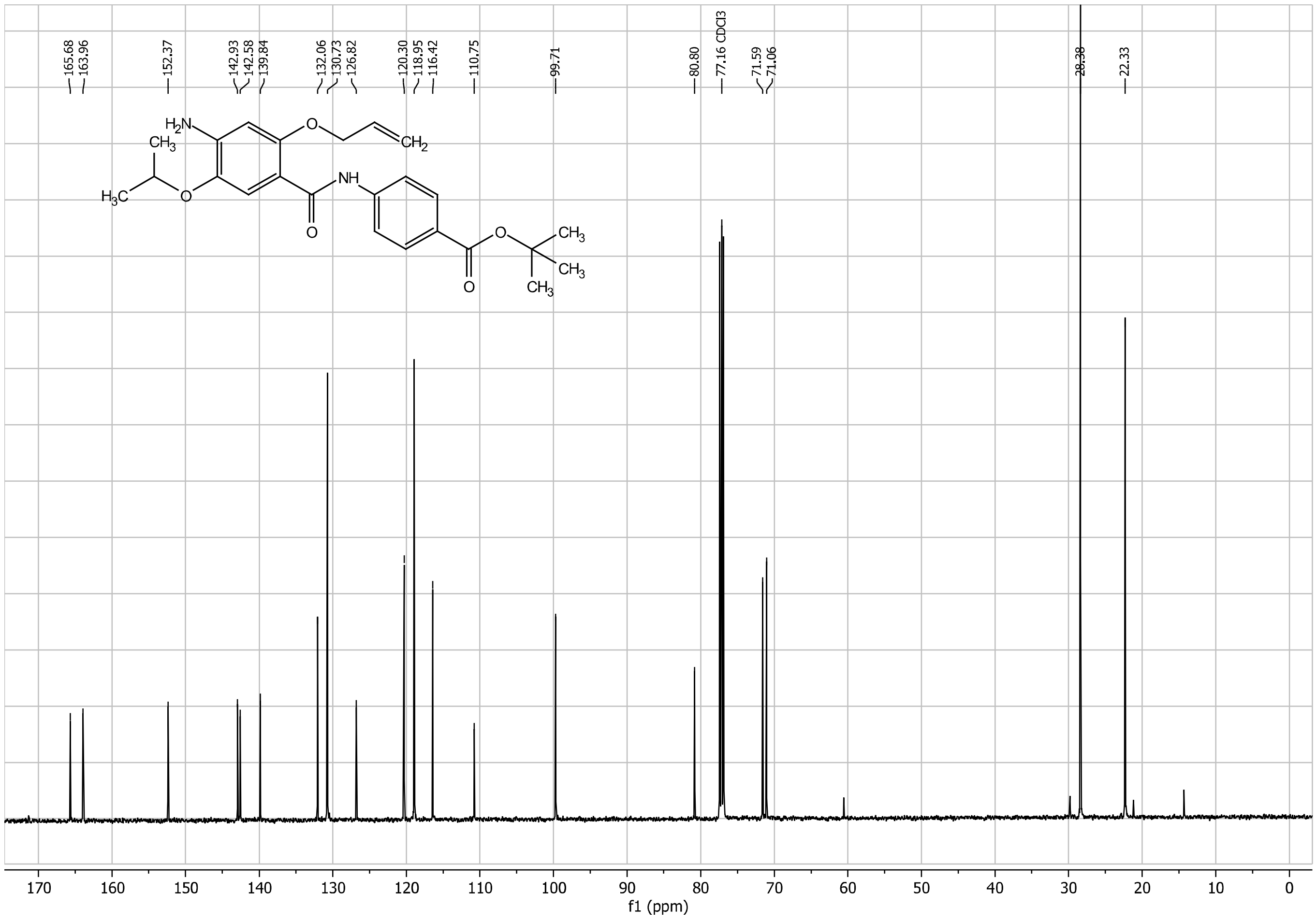
165.68
163.96
152.37
142.93
142.58
139.84
132.06
130.73
126.82
120.30
118.95
116.42
110.75
99.71
80.80
77.16 CDCl3
71.59
71.06
28.38
22.33
H2N
CH3
H3C
O
O
NH
CH2
O
O
CH3
CH3
CH3
170 160 150 140 130 120 110 100 90 80 70 60 50 40 30 20 10 0
f1 (ppm)

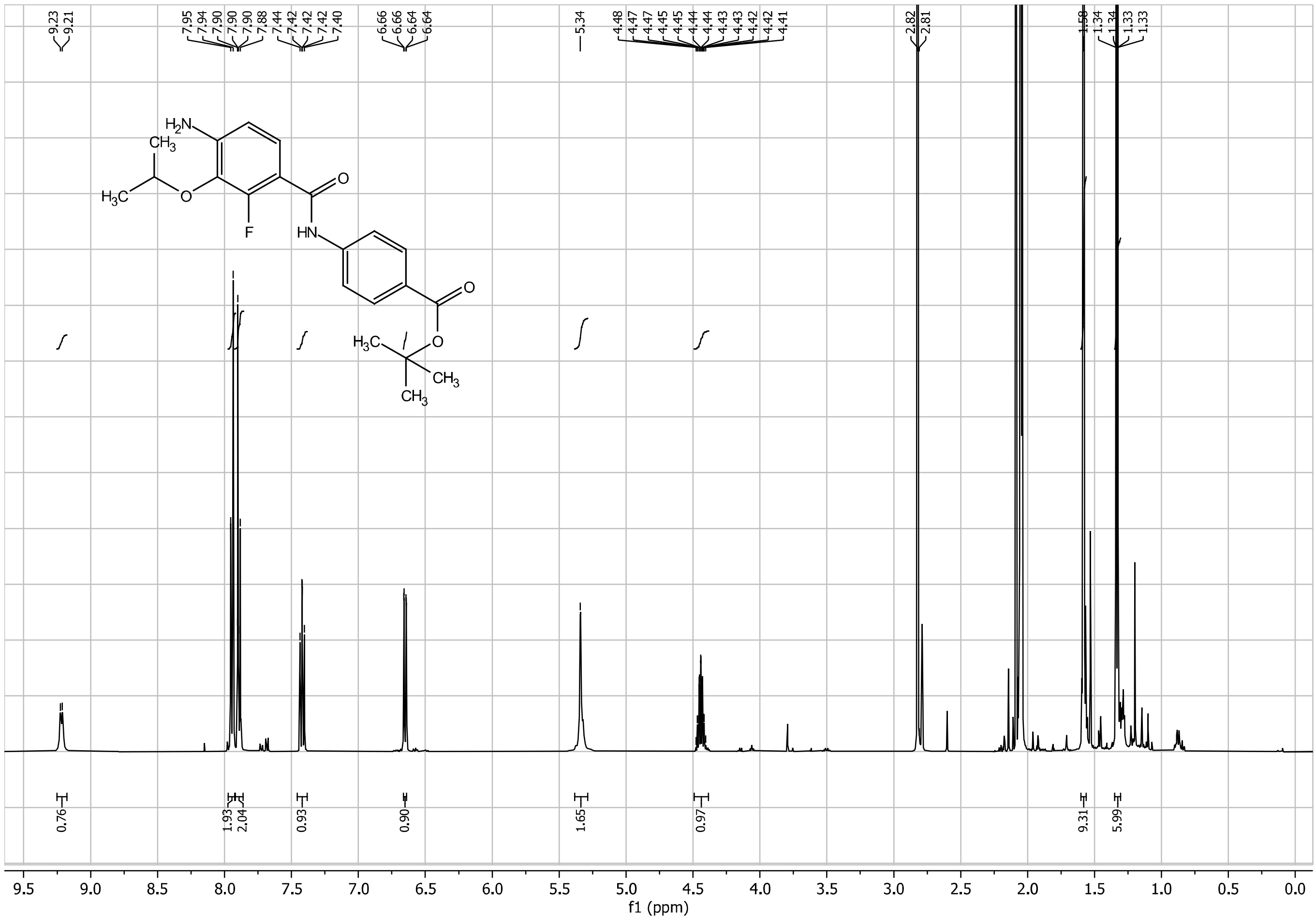
9.23
9.21
7.95
7.94
7.90
7.90
7.90
7.88
7.44
7.42
7.42
7.42
7.40
6.66
6.66
6.64
6.64
5.34
4.48
4.47
4.47
4.45
4.45
4.44
4.44
4.43
4.43
4.42
4.42
4.41
2.82
2.81
1.34
1.34
1.33
1.33
H_2N
CH_3
H_3C
O
F
HN
O
O
H_3C
O
CH_3
CH_3
0.76
1.93
2.04
0.93
0.90
1.65
0.97
9.31
5.99
9.5 9.0 8.5 8.0 7.5 7.0 6.5 6.0 5.5 5.0 4.5 4.0 3.5 3.0 2.5 2.0 1.5 1.0 0.5 0.0
f1 (ppm)

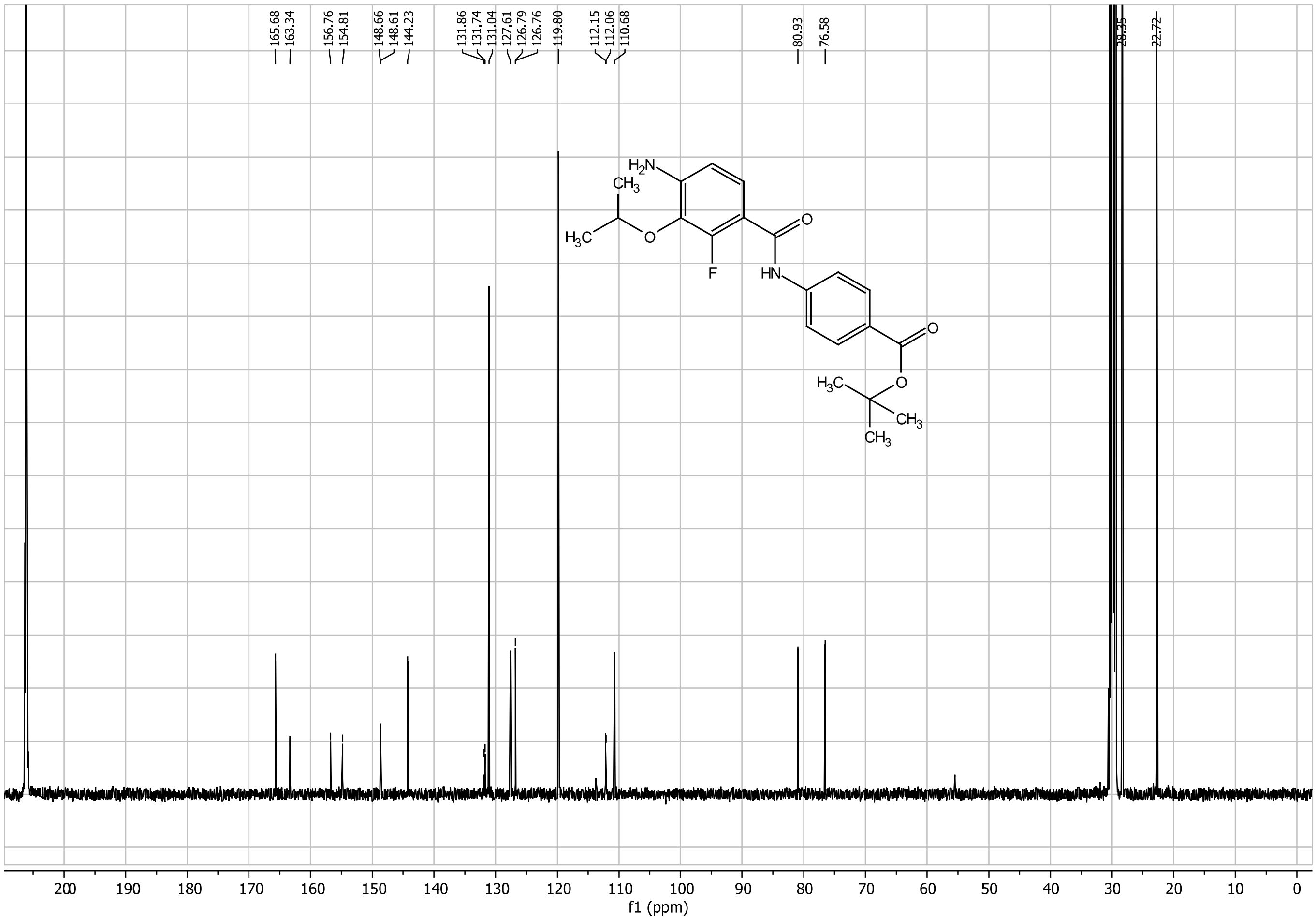
165.68
163.34
156.76
154.81
148.66
148.61
144.23
131.86
131.74
131.04
127.61
126.79
126.76
119.80
112.15
112.06
110.68
80.93
76.58
28.35
22.72
H_2N
CH_3
H_3C
O
F
HN
O
O
H_3C
O
CH_3
CH_3
200 190 180 170 160 150 140 130 120 110 100 90 80 70 60 50 40 30 20 10 0
f1 (ppm)

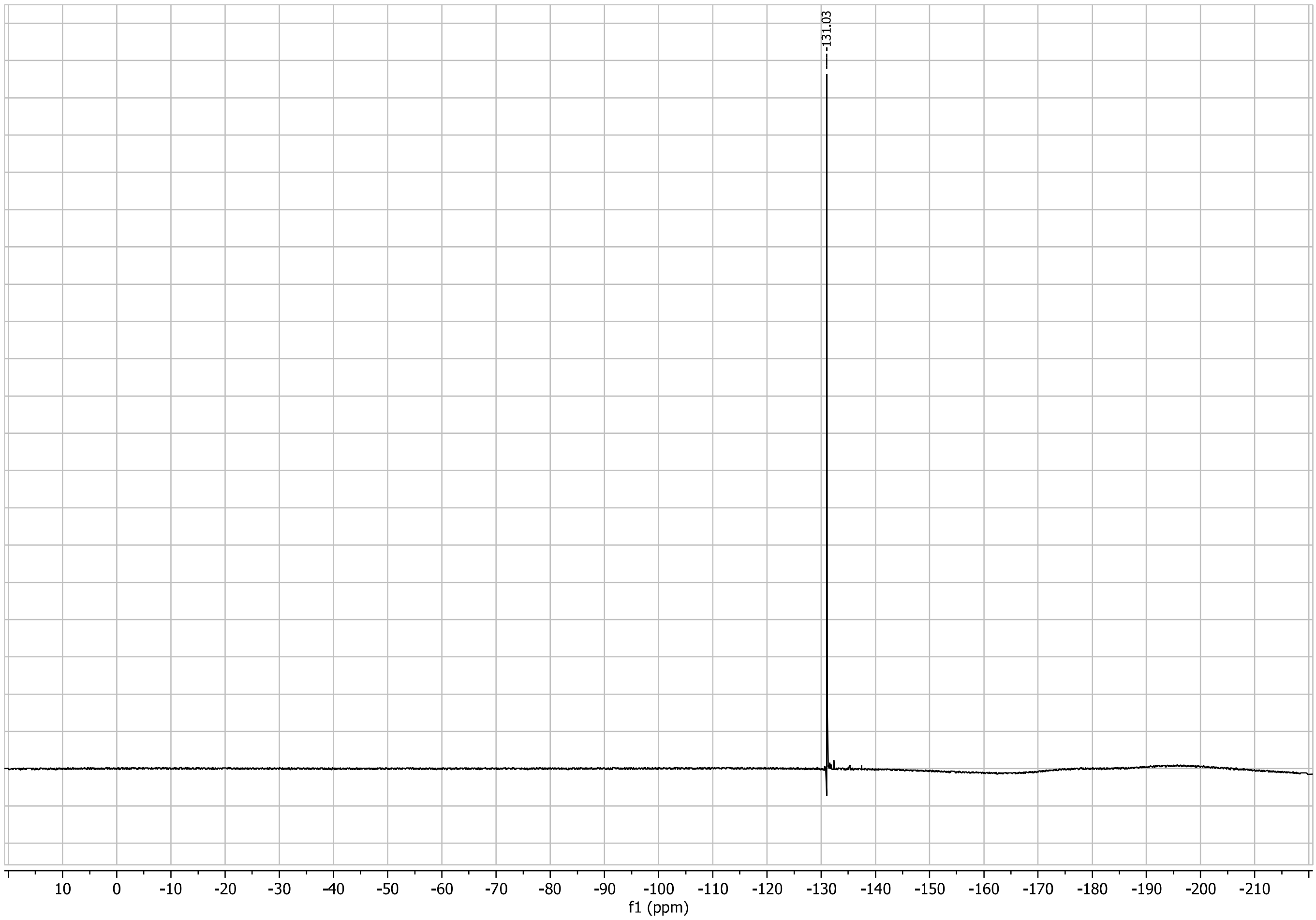
-131.03
10 0 -10 -20 -30 -40 -50 -60 -70 -80 -90 -100 -110 -120 -130 -140 -150 -160 -170 -180 -190 -200 -210
f1 (ppm)

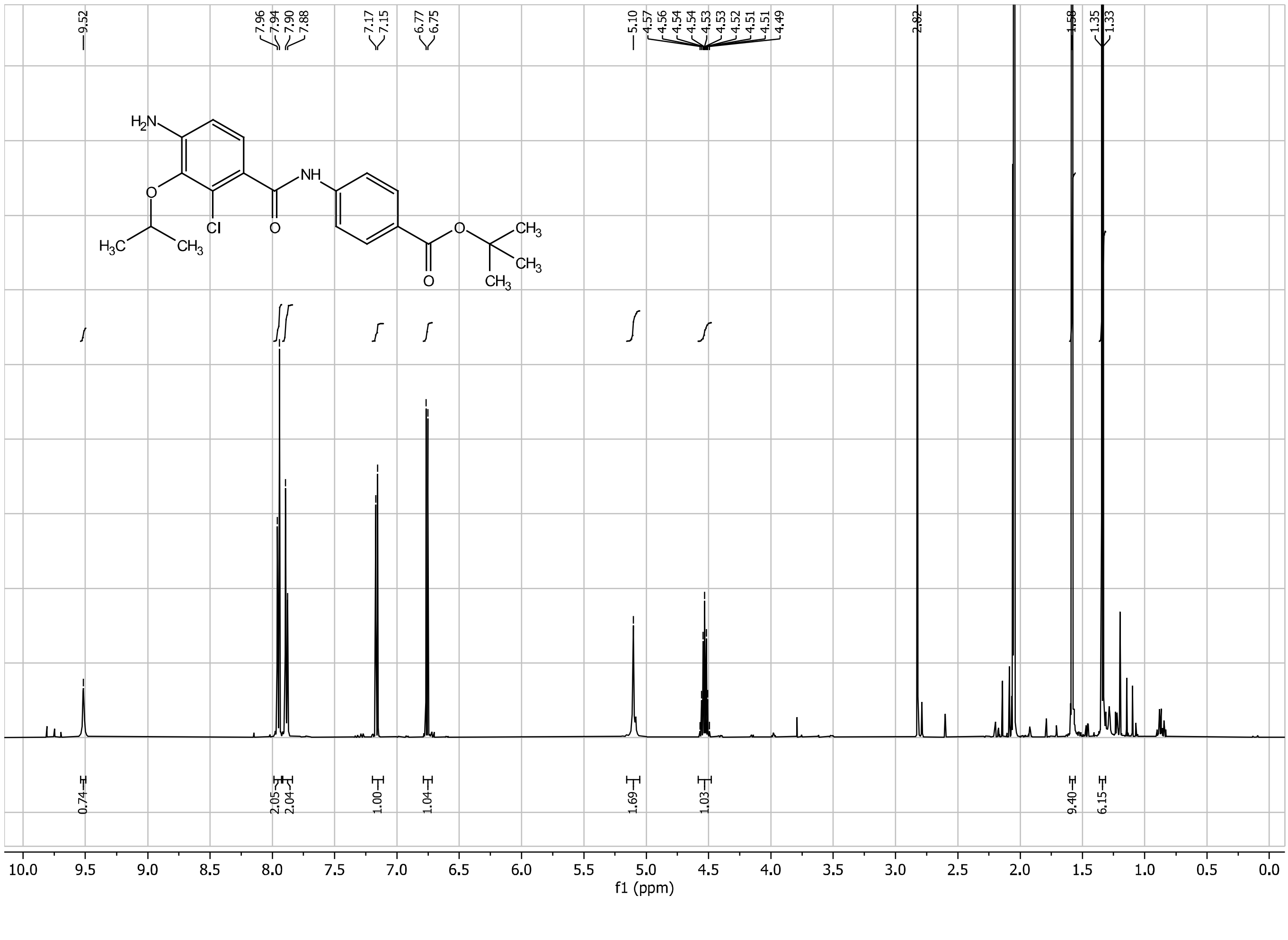
9.52
7.96 7.94 7.90 7.88
7.17 7.15
6.77 6.75
5.10 4.57 4.56 4.54 4.54 4.53 4.53 4.52 4.51 4.51 4.49
2.82
1.58
1.35 1.33
H2N
NH
O
Cl
O
H3C
CH3
O
CH3
CH3
O
CH3
0.74
2.05
2.04
1.00
1.04
1.69
1.03
9.40
6.15
10.0 9.5 9.0 8.5 8.0 7.5 7.0 6.5 6.0 5.5 5.0 4.5 4.0 3.5 3.0 2.5 2.0 1.5 1.0 0.5 0.0
f1 (ppm)

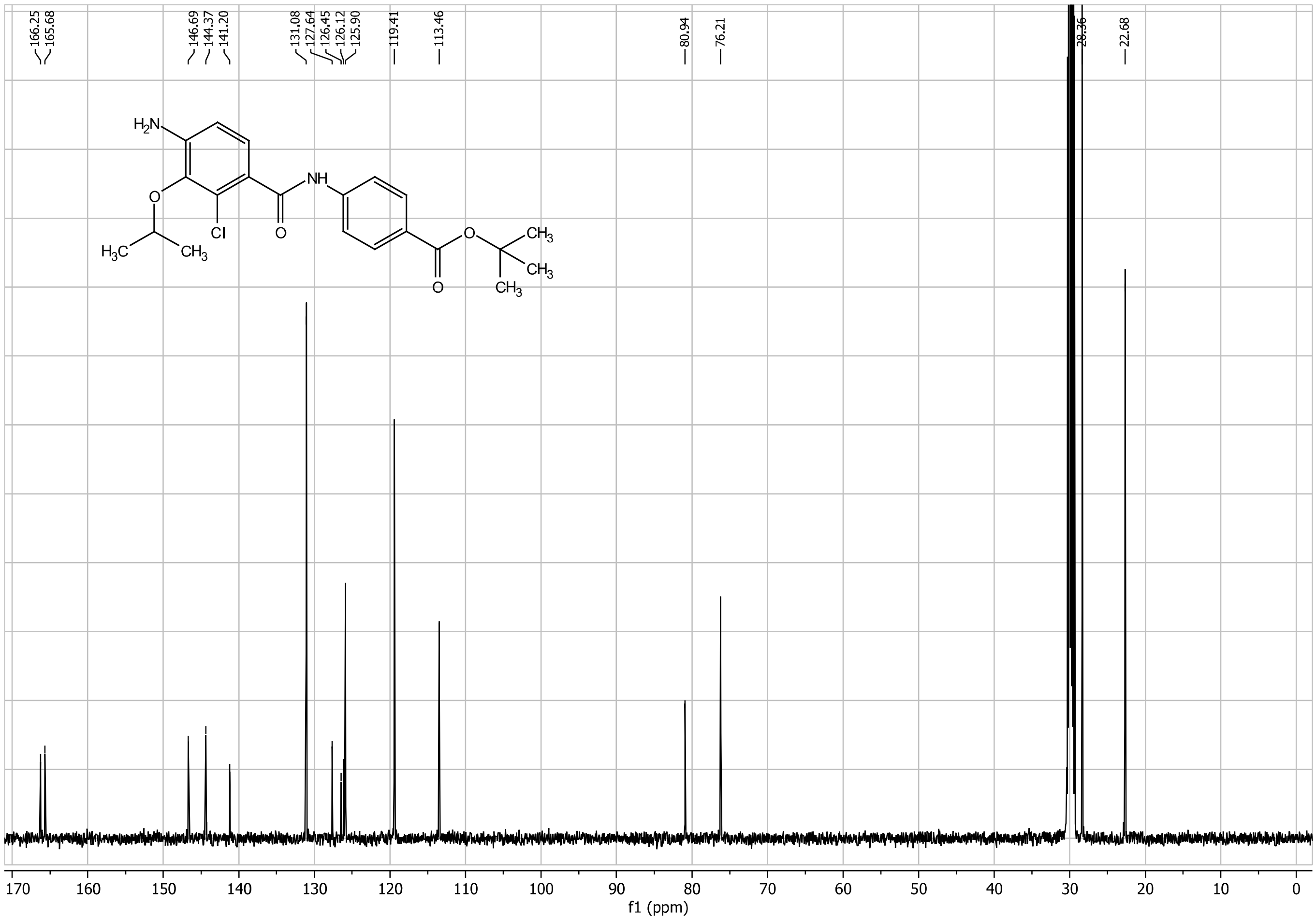
166.25
165.68
146.69
144.37
141.20
131.08
127.64
126.45
126.12
125.90
119.41
113.46
80.94
76.21
28.36
22.68
f1 (ppm)

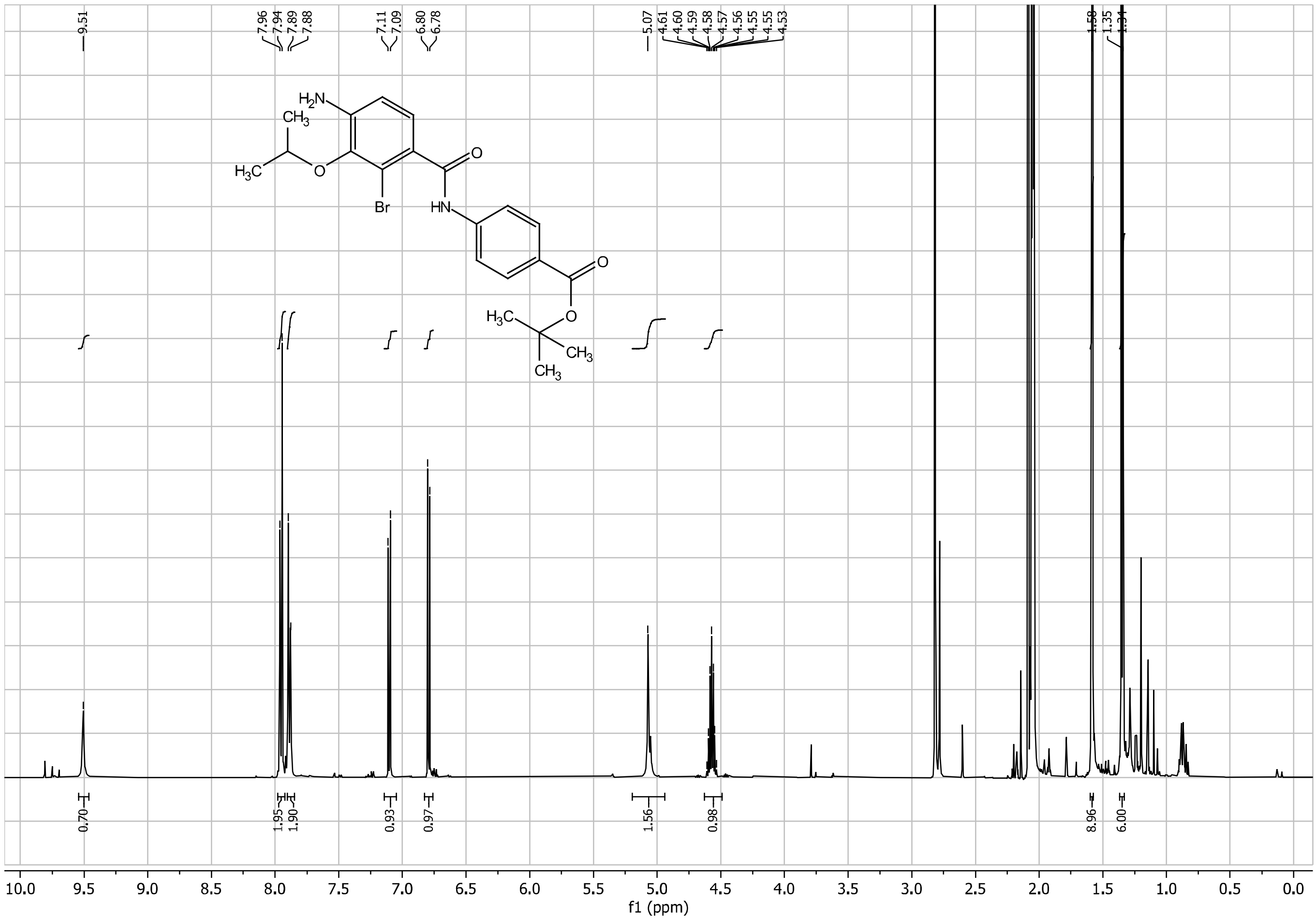
9.51
7.96
7.94
7.89
7.88
7.11
7.09
6.80
6.78
5.07
4.61
4.60
4.59
4.58
4.57
4.56
4.55
4.55
4.53
1.58
1.35
0.70
1.95
1.90
0.93
0.97
1.56
0.98
8.96
6.00
f1 (ppm)

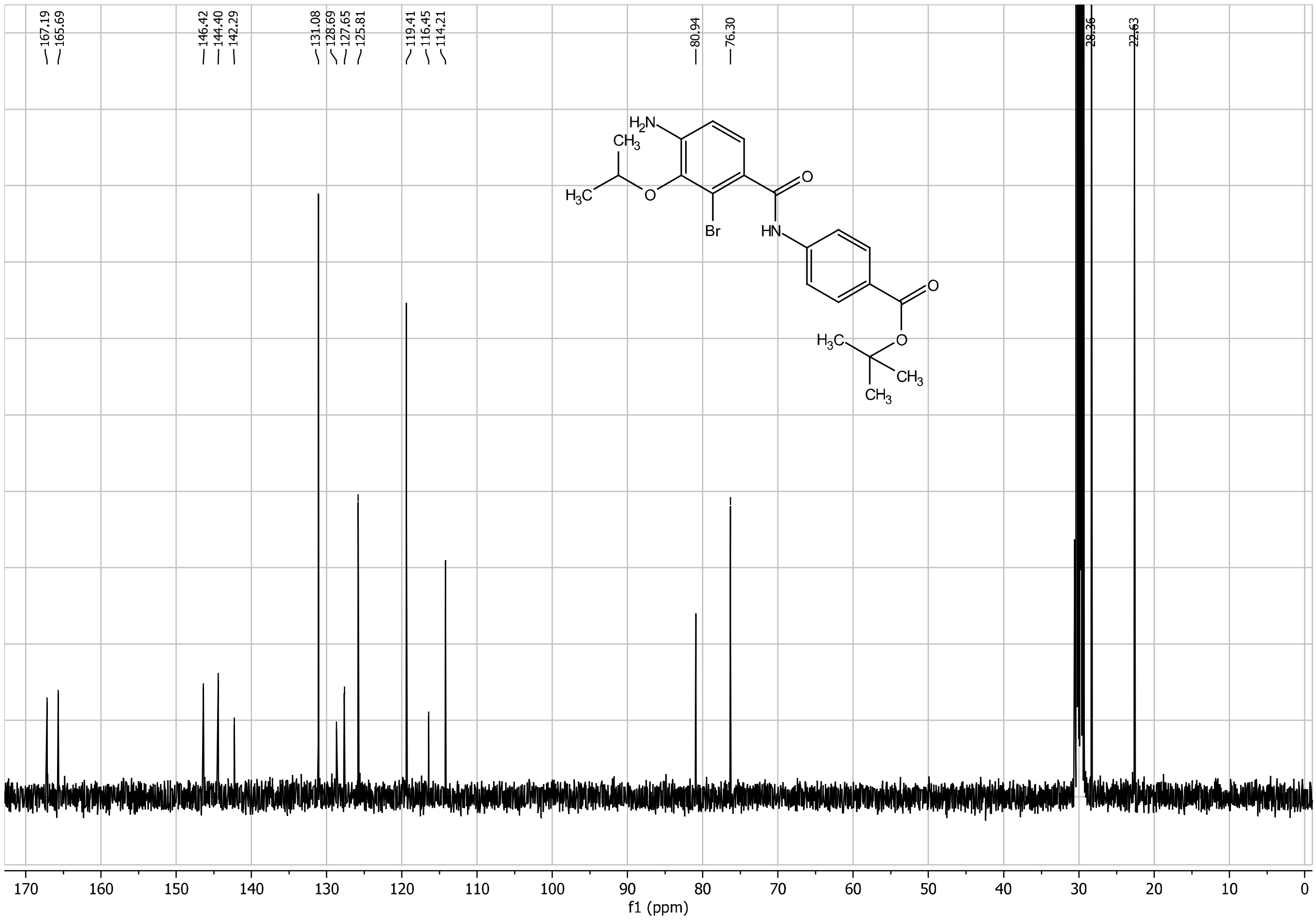
167.19
165.69
146.42
144.40
142.29
131.08
128.69
127.65
125.81
119.41
116.45
114.21
80.94
76.30
28.36
22.63
f1 (ppm)

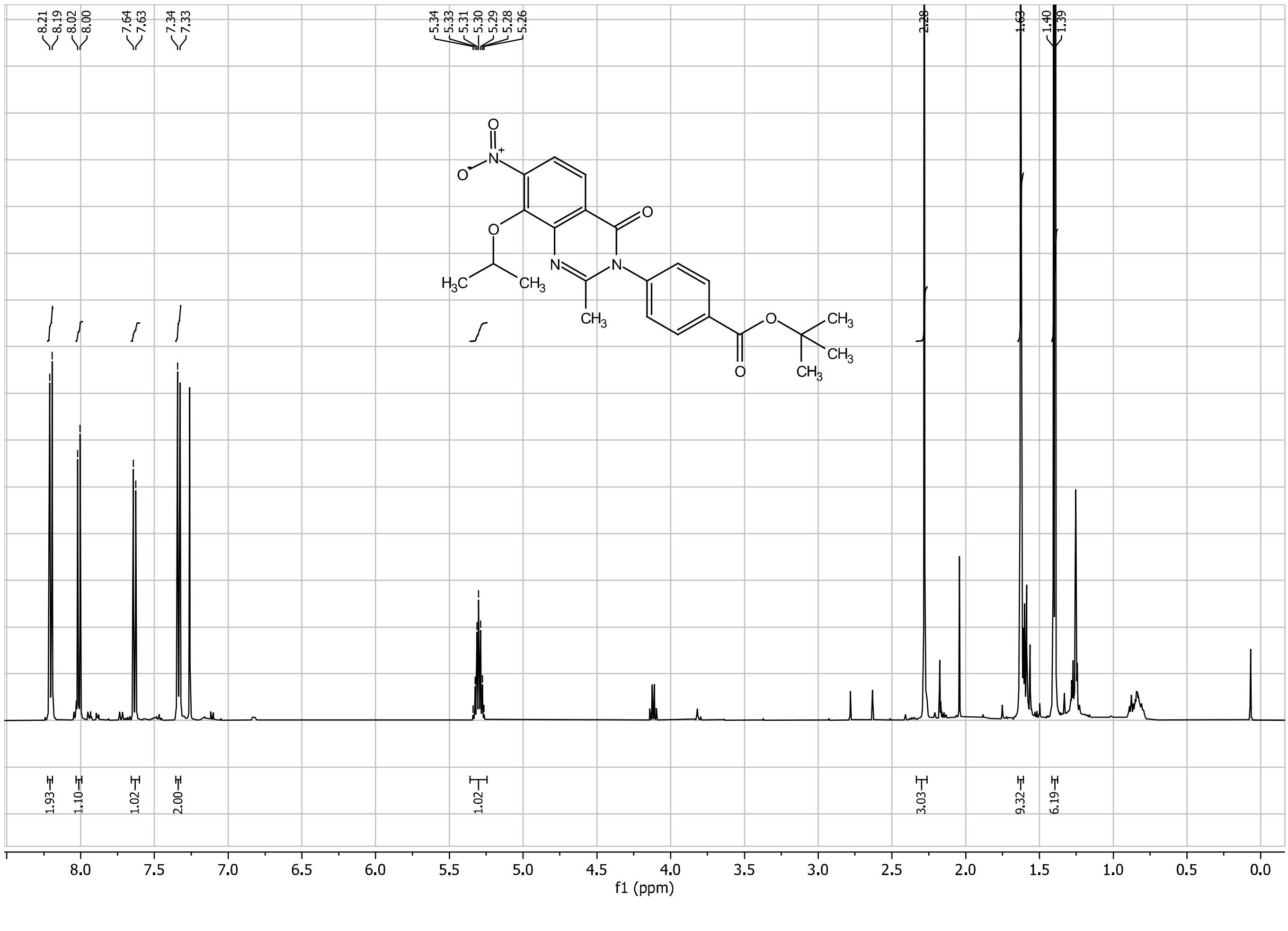
8.21
8.19
8.02
8.00
7.64
7.63
7.34
7.33
5.34
5.33
5.31
5.30
5.29
5.28
5.26
2.28
1.63
1.40
1.39
1.93
1.10
1.02
2.00
1.02
3.03
9.32
6.19
f1 (ppm)

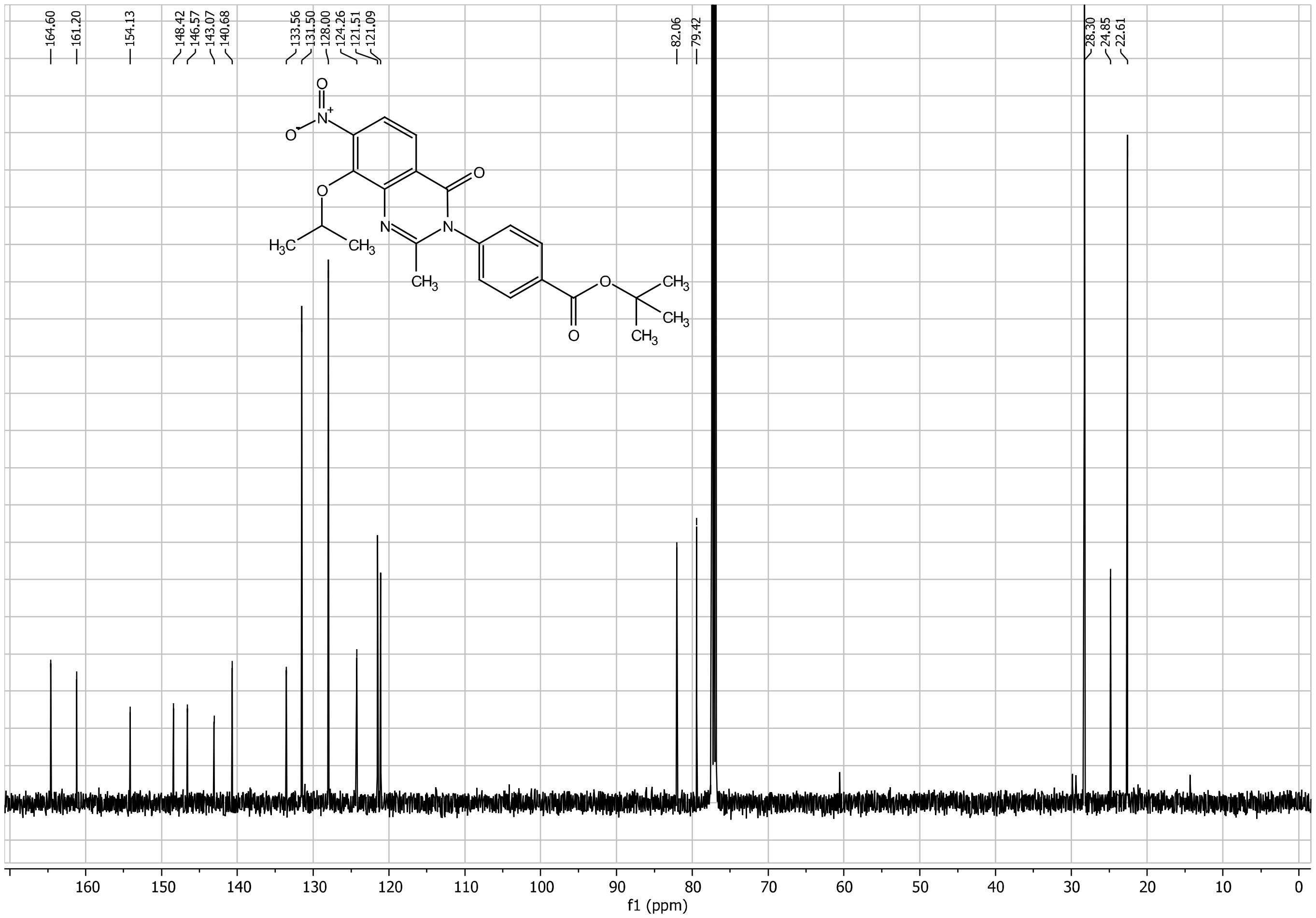
164.60
161.20
154.13
148.42
146.57
143.07
140.68
133.56
131.50
128.00
124.26
121.51
121.09
82.06
79.42
28.30
24.85
22.61
f1 (ppm)

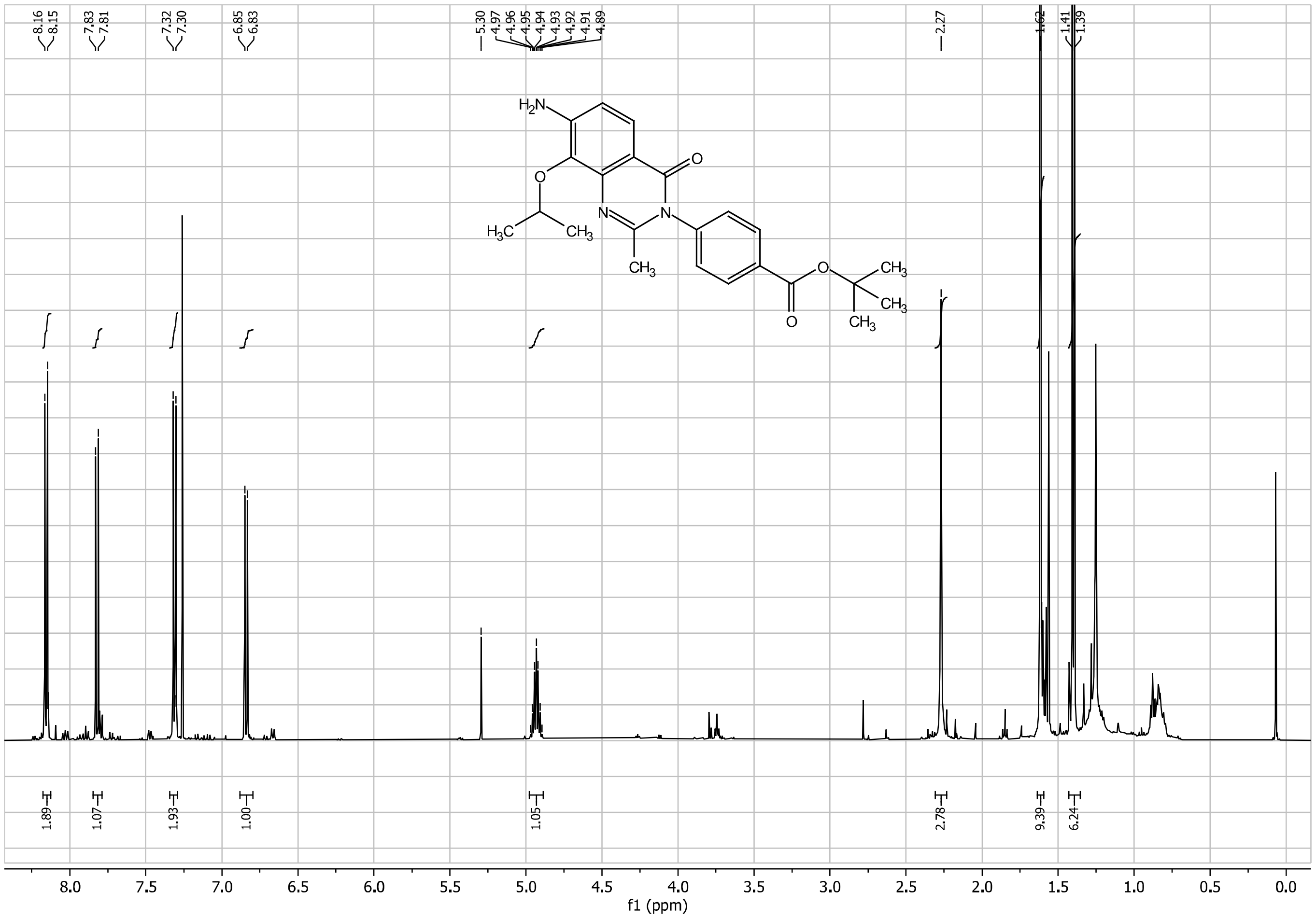
8.16
8.15
7.83
7.81
7.32
7.30
6.85
6.83
5.30
4.97
4.96
4.95
4.94
4.93
4.92
4.91
4.89
2.27
1.62
1.41
1.39
1.89
1.07
1.93
1.00
1.05
2.78
9.39
6.24
f1 (ppm)

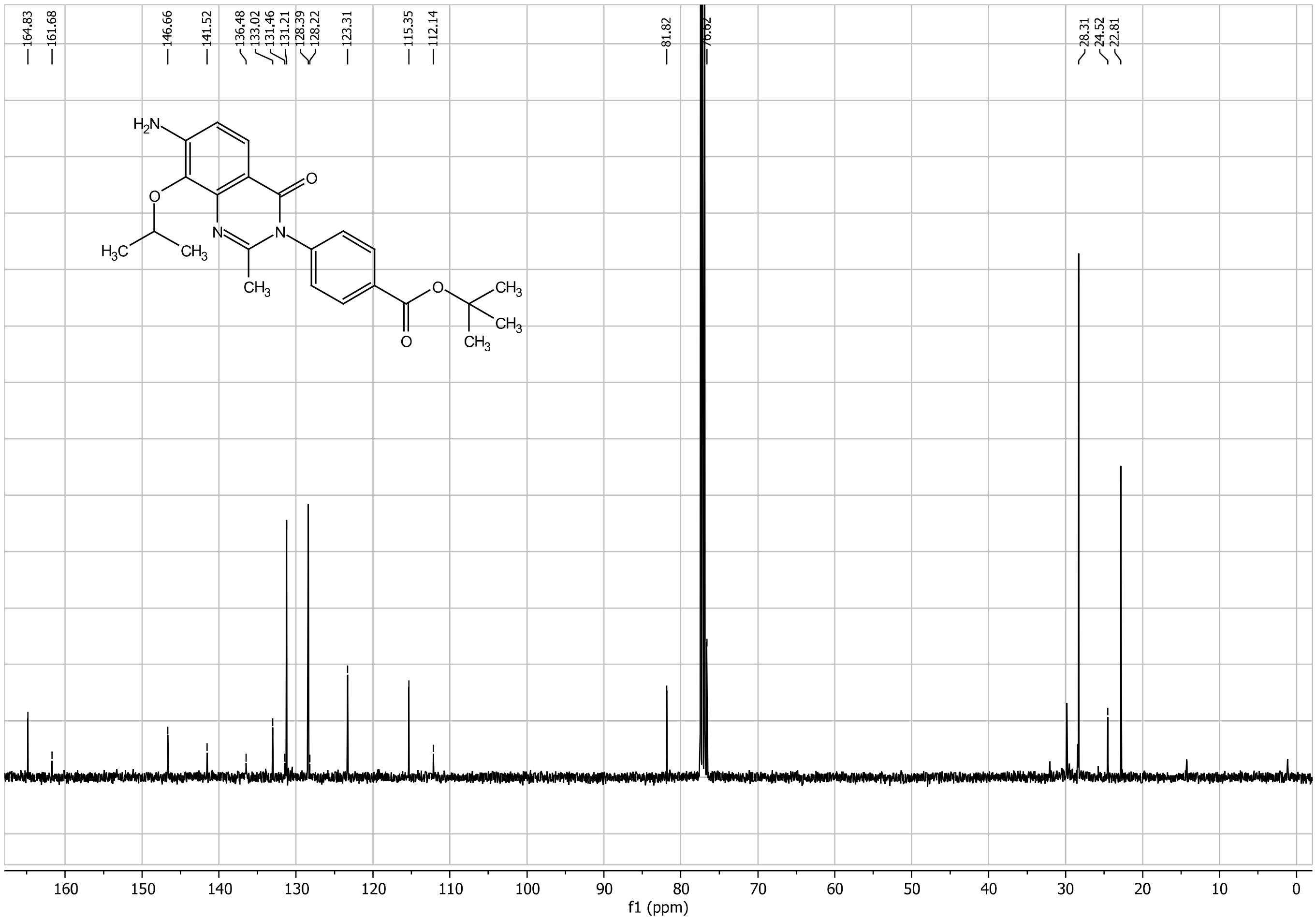
164.83
161.68
146.66
141.52
136.48
133.02
131.46
131.21
128.39
128.22
123.31
115.35
112.14
81.82
28.31
24.52
22.81
H2N
O
O
N
N
H3C
CH3
CH3
O
O
CH3
CH3
CH3
f1 (ppm)

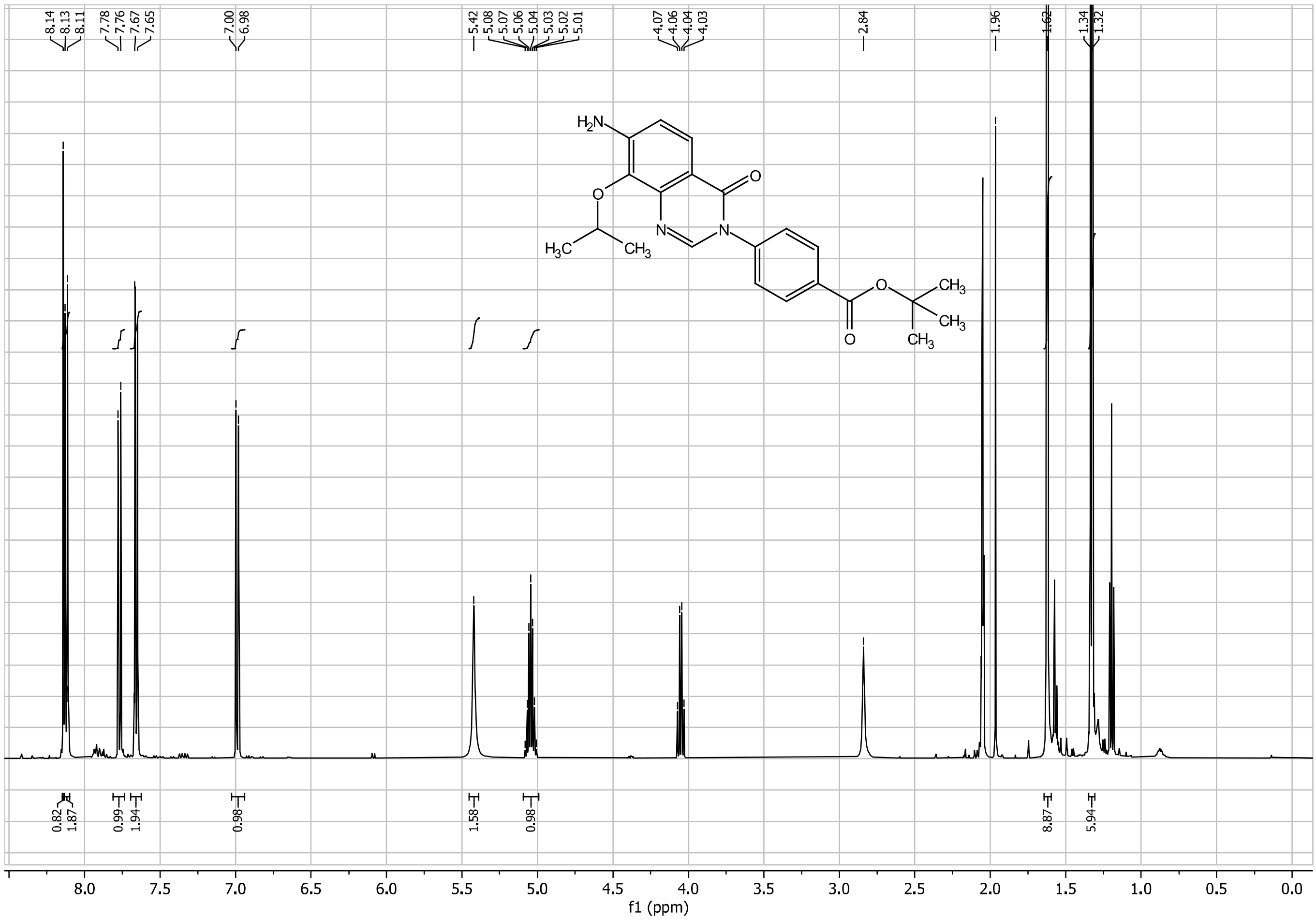
8.14
8.13
8.11
7.78
7.76
7.67
7.65
7.00
6.98
5.42
5.08
5.07
5.06
5.04
5.03
5.02
5.01
4.07
4.06
4.04
4.03
2.84
1.96
1.62
1.34
1.32
H2N
O
O
N
N
H3C
CH3
O
O
CH3
CH3
CH3
0.82
1.87
0.99
1.94
0.98
1.58
0.98
8.87
5.94
f1 (ppm)

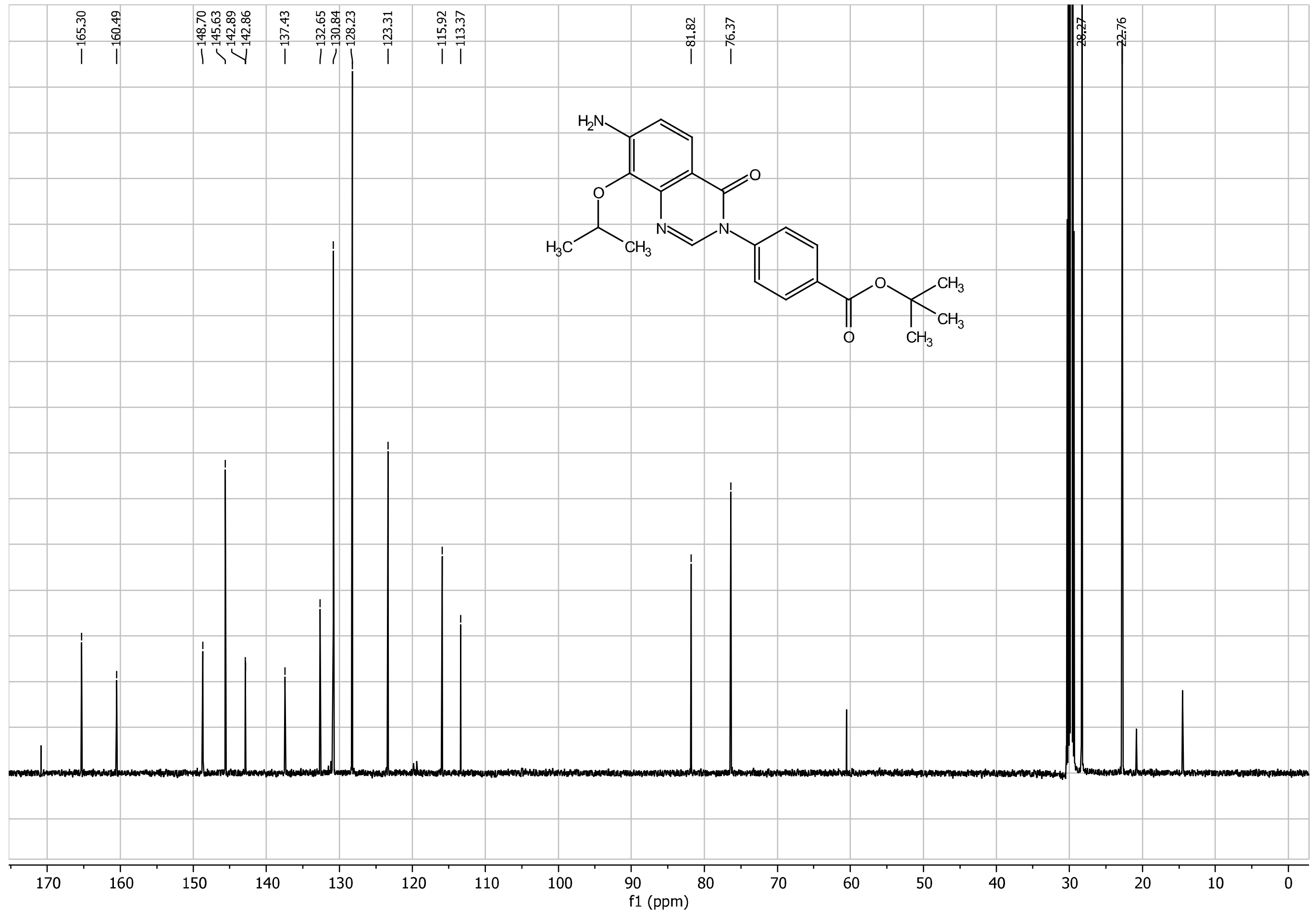

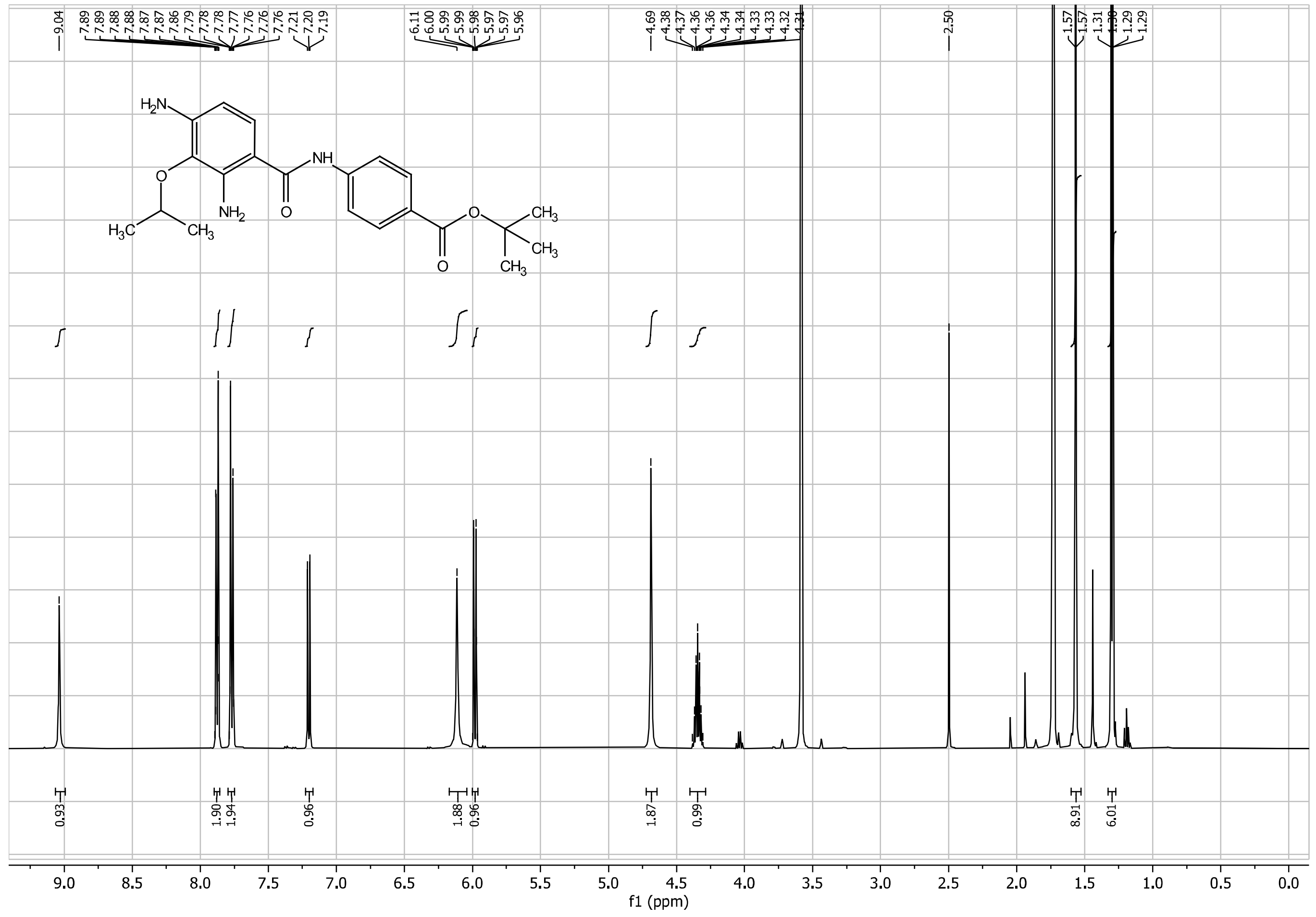

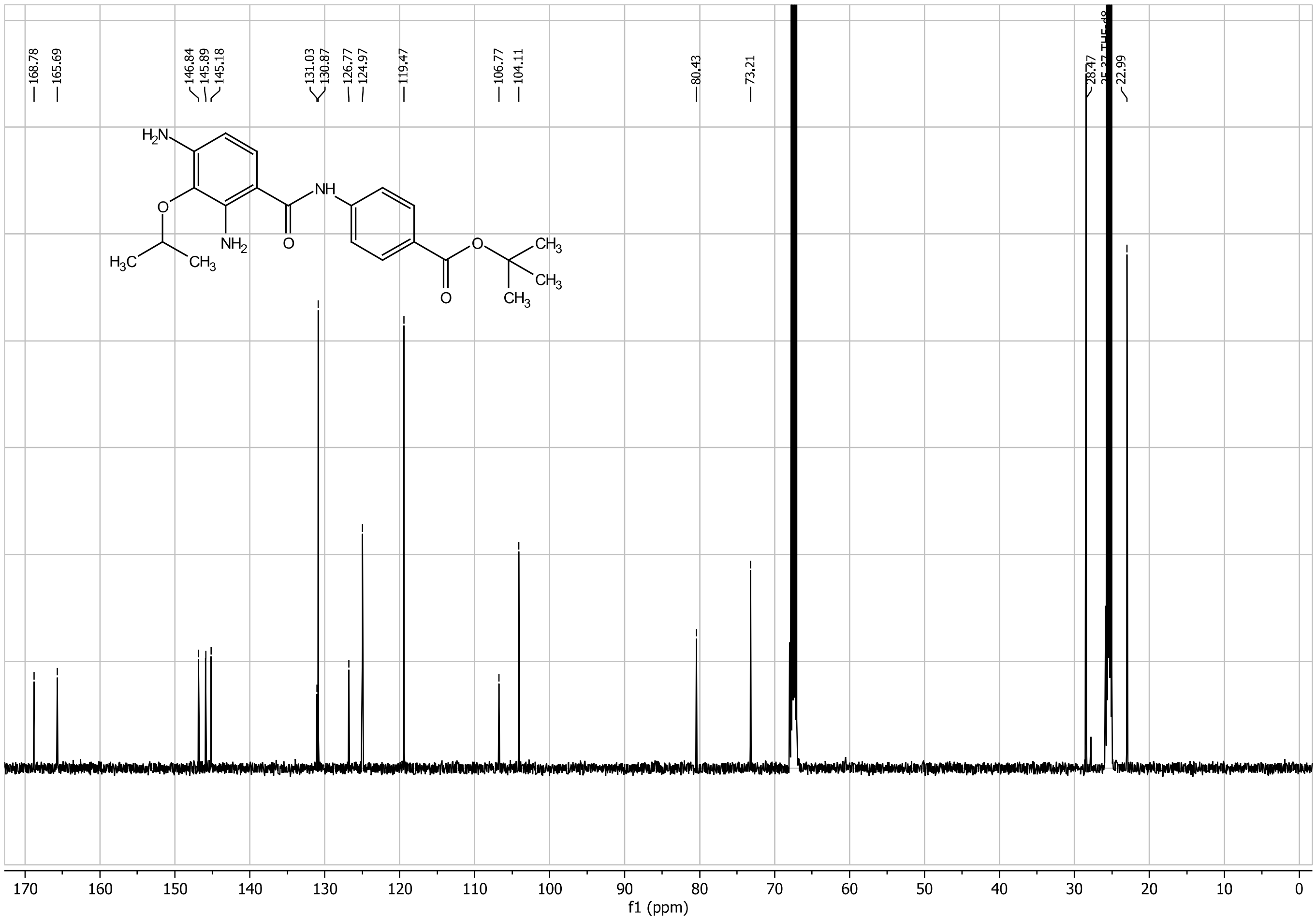

168.78
165.69
146.84
145.89
145.18
131.03
130.87
126.77
124.97
119.47
106.77
104.11
80.43
73.21
28.47
22.99
H2N
O
NH
NH2
O
H3C
CH3
O
O
CH3
CH3
CH3
O
170 160 150 140 130 120 110 100 90 80 70 60 50 40 30 20 10 0
f1 (ppm)

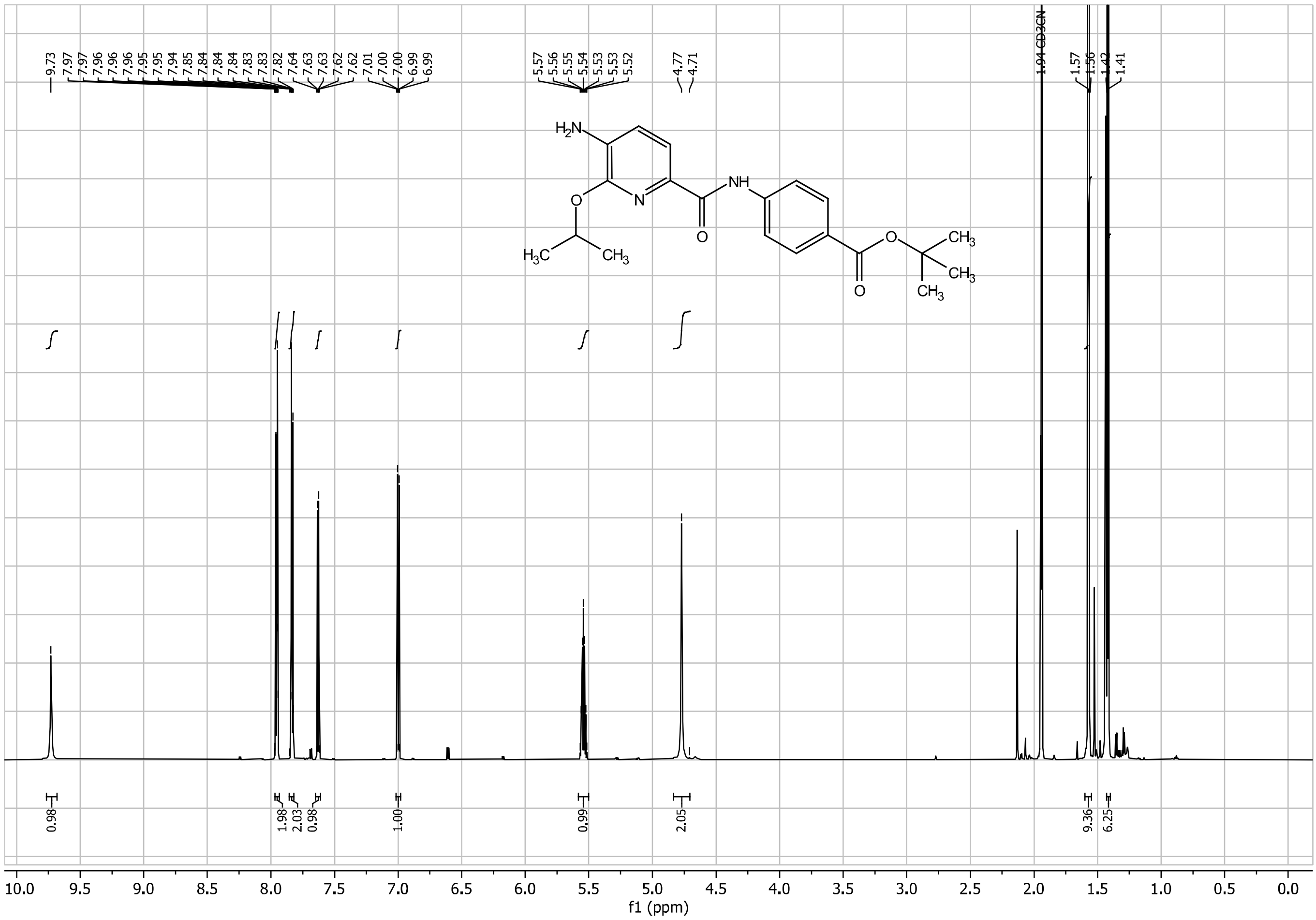

9.73
7.97
7.97
7.96
7.96
7.95
7.95
7.94
7.85
7.84
7.84
7.84
7.83
7.83
7.82
7.64
7.63
7.63
7.62
7.62
7.01
7.00
7.00
6.99
6.99
5.57
5.56
5.55
5.54
5.53
5.53
5.52
4.77
4.71
1.94 CD3CN
1.57
1.56
1.42
1.41
H2N
O
N
NH
O
H3C
CH3
O
O
CH3
CH3
CH3
O
0.98
1.98
2.03
0.98
1.00
0.99
2.05
9.36
6.25
10.0 9.5 9.0 8.5 8.0 7.5 7.0 6.5 6.0 5.5 5.0 4.5 4.0 3.5 3.0 2.5 2.0 1.5 1.0 0.5 0.0
f1 (ppm)

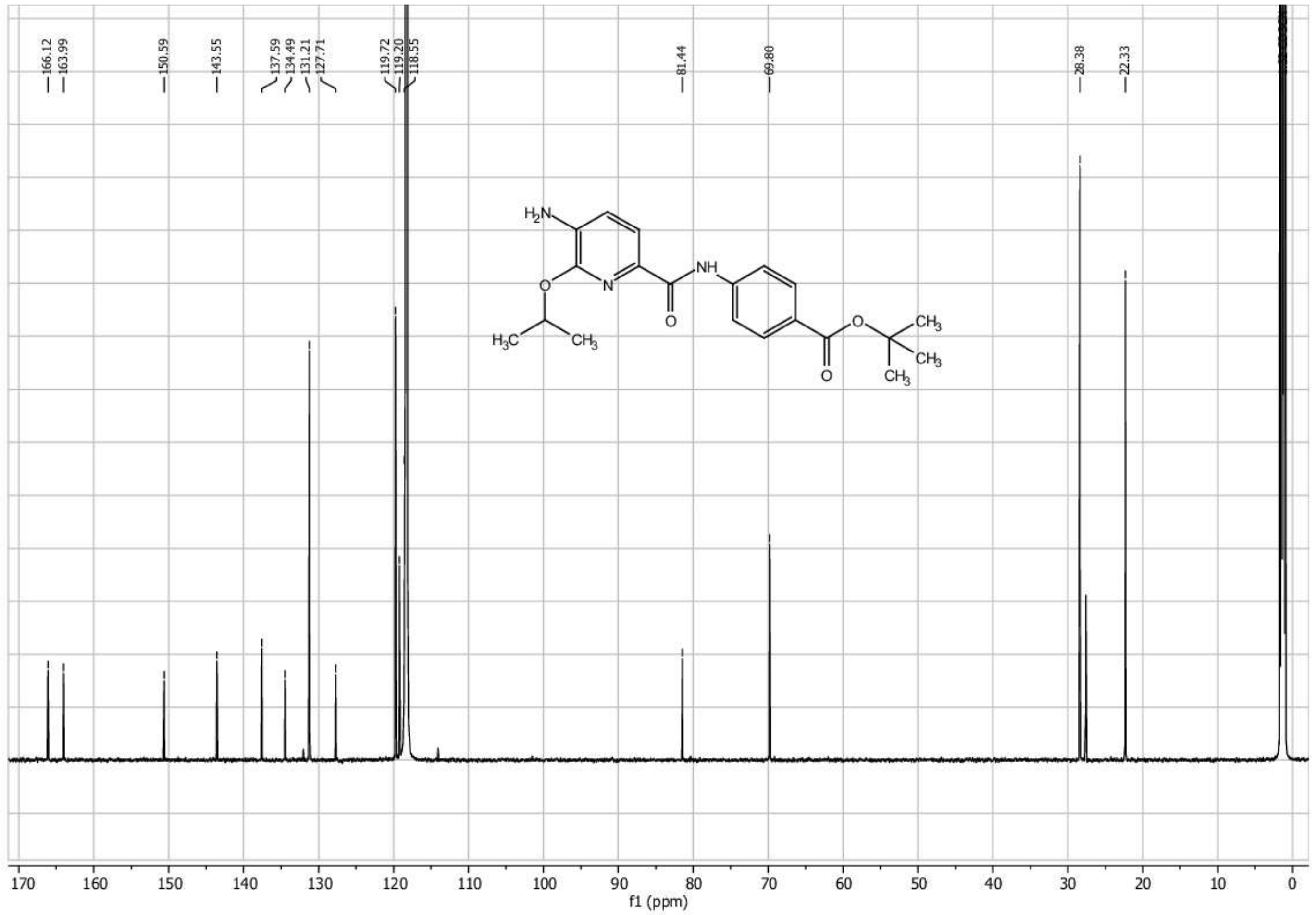
166.12
163.99
150.59
143.55
137.59
134.49
131.21
127.71
119.72
119.20
118.55
81.44
69.80
28.38
22.33
H2N
O
N
NH
O
H3C
CH3
O
CH3
CH3
O
CH3
170 160 150 140 130 120 110 100 90 80 70 60 50 40 30 20 10 0
f1 (ppm)

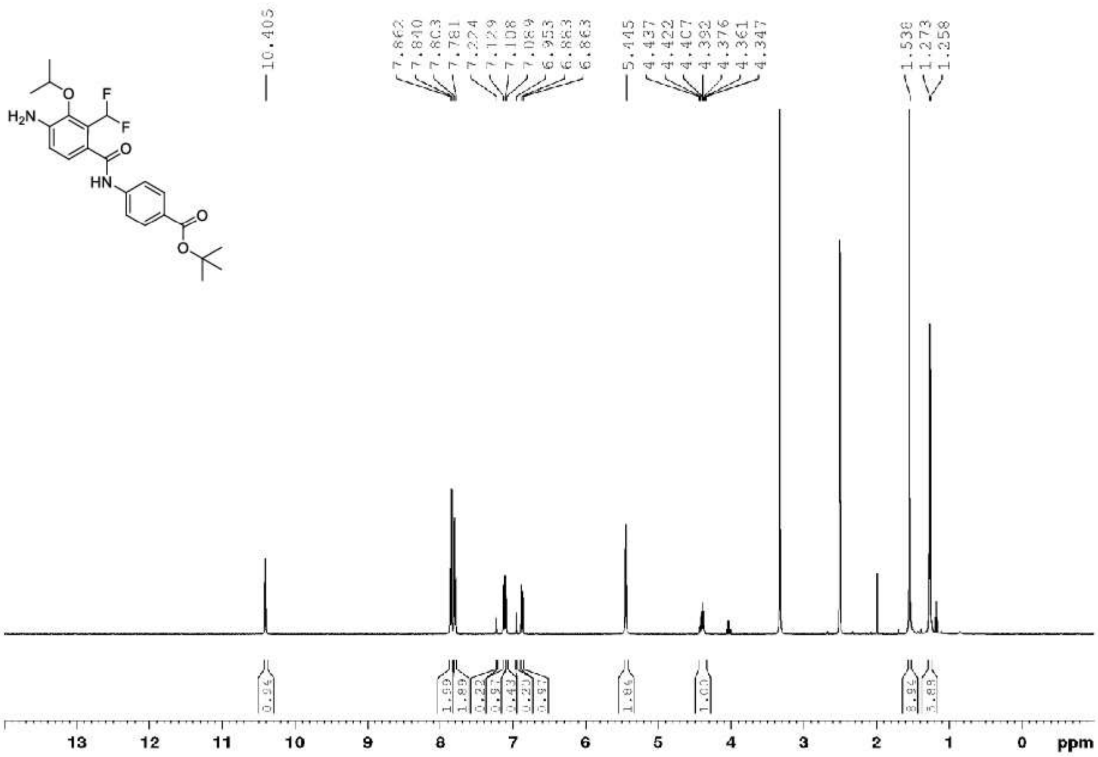
10.405
7.862
7.840
7.803
7.781
7.224
7.129
7.108
7.089
6.953
6.883
6.863
5.445
4.437
4.422
4.407
4.392
4.376
4.361
4.347
1.536
1.273
1.258
ppm

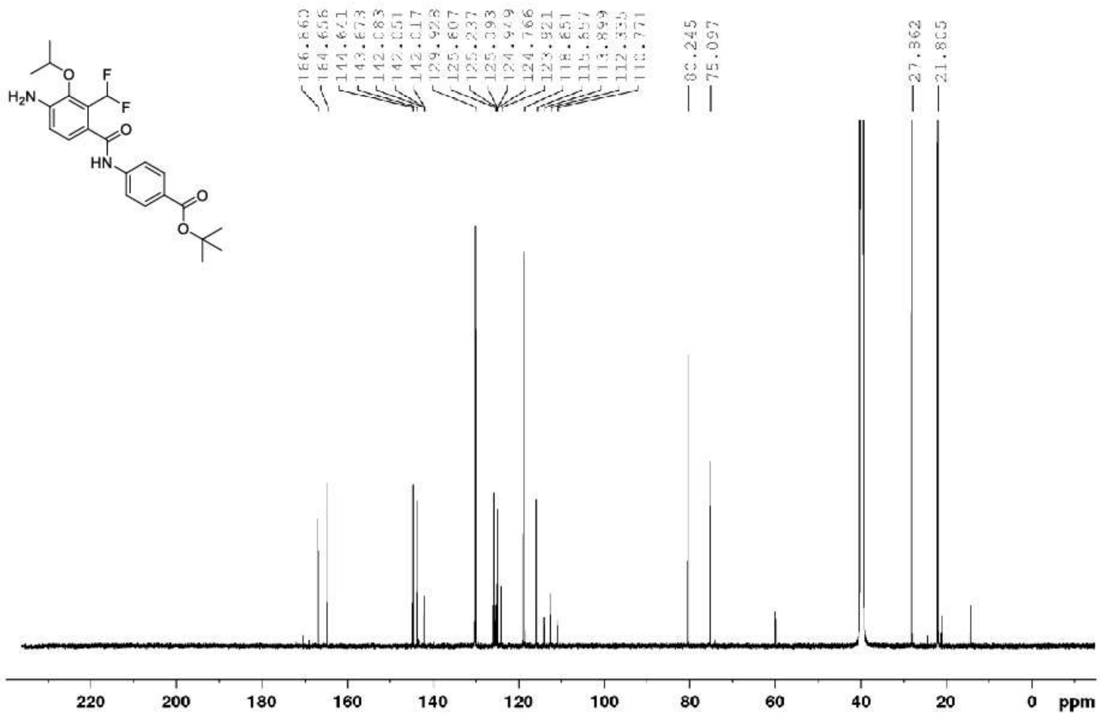

H2N
F
F
O
HN
O
O
166.860
164.656
144.641
143.673
142.083
142.051
142.017
129.928
125.607
125.237
125.393
124.979
124.766
123.921
118.651
115.557
113.899
112.335
110.771
80.245
75.097
27.362
21.805
220
200
180
160
140
120
100
80
60
40
20
0
ppm

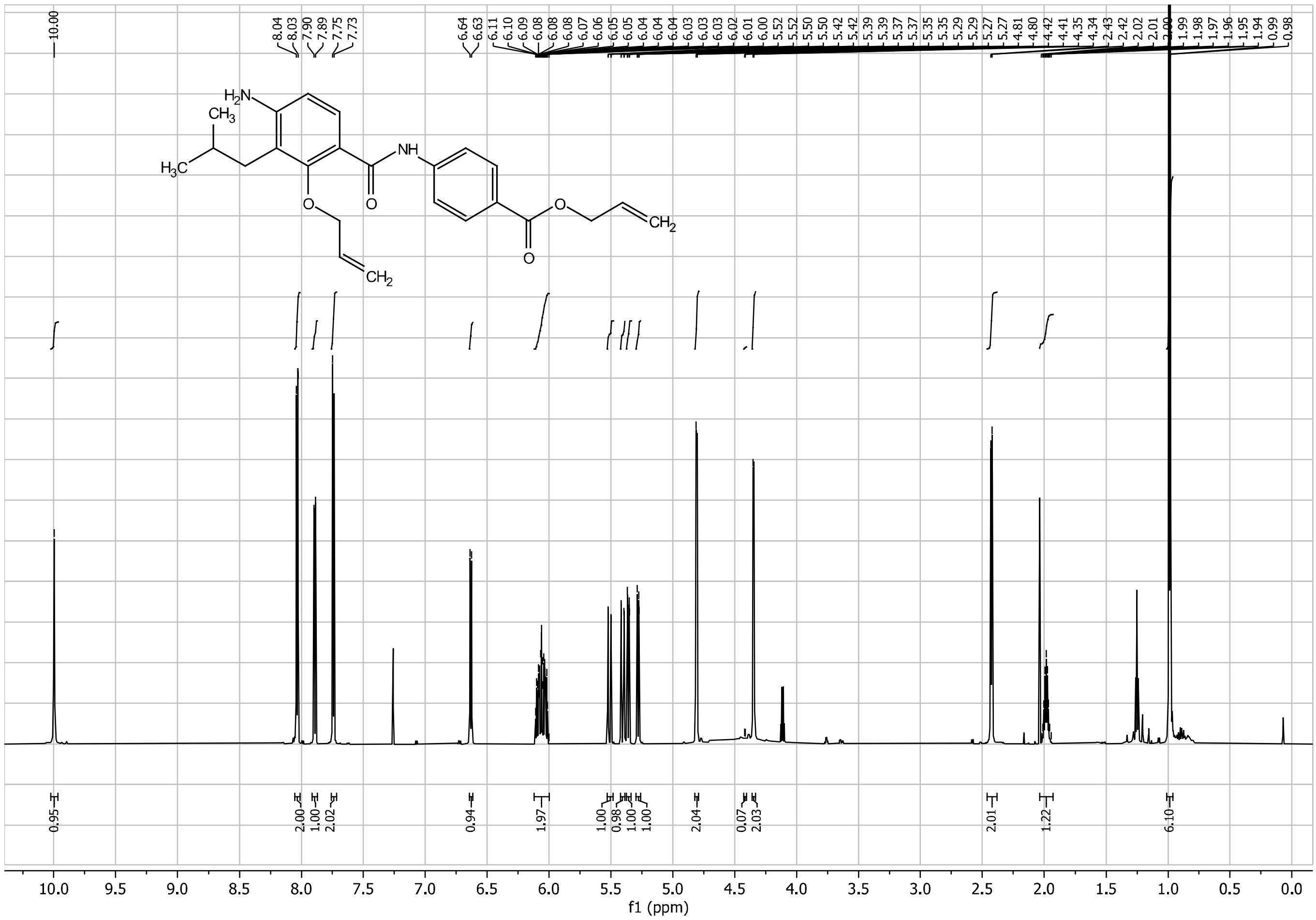

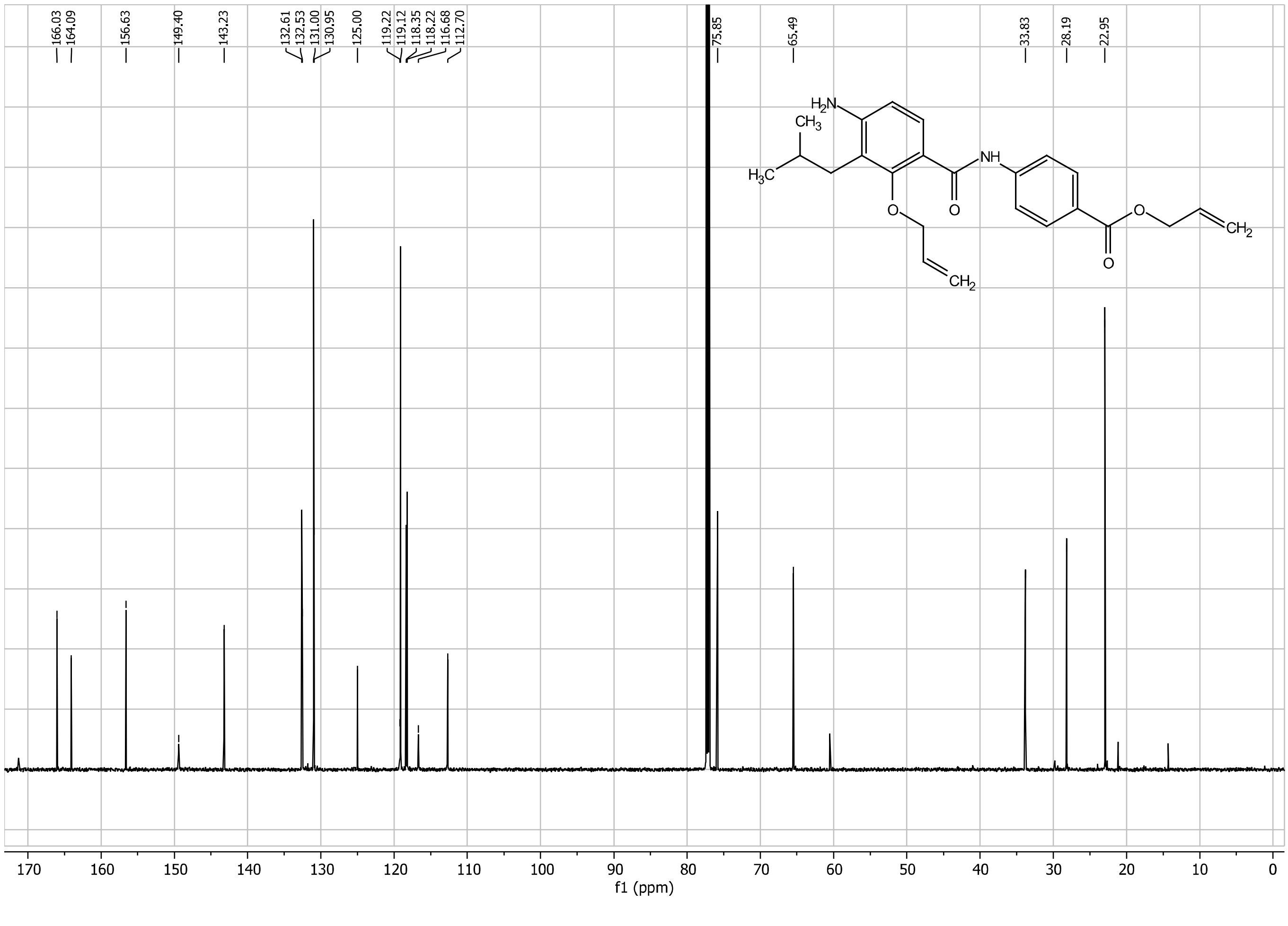

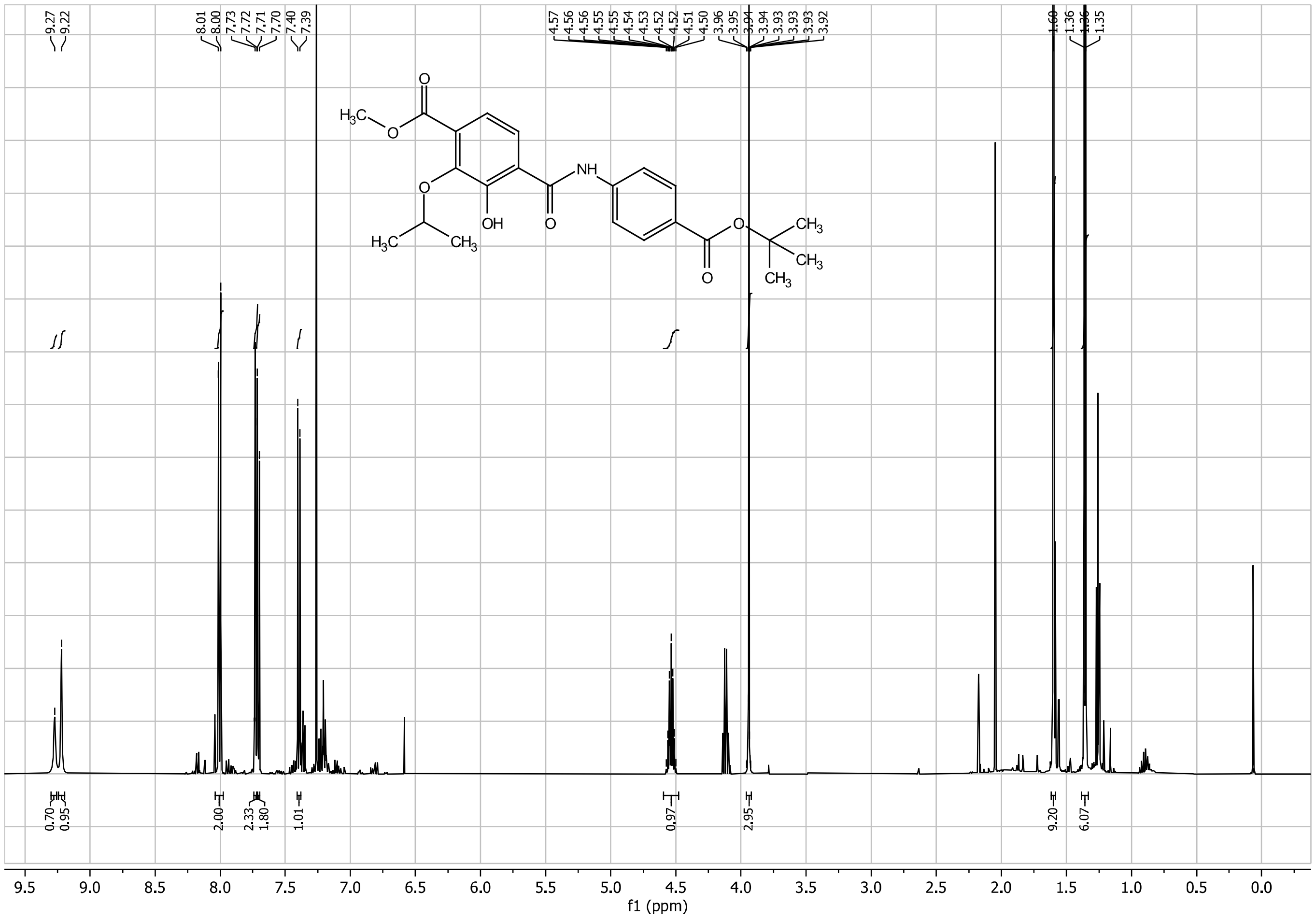
9.27
9.22
8.01
8.00
7.73
7.72
7.71
7.70
7.40
7.39
4.57
4.56
4.56
4.55
4.55
4.54
4.53
4.52
4.52
4.51
4.50
3.96
3.95
3.94
3.94
3.93
3.93
3.93
3.93
3.92
1.60
1.36
1.36
1.35
0.70
0.95
2.00
2.33
1.80
1.01
0.97
2.95
9.20
6.07
f1 (ppm)

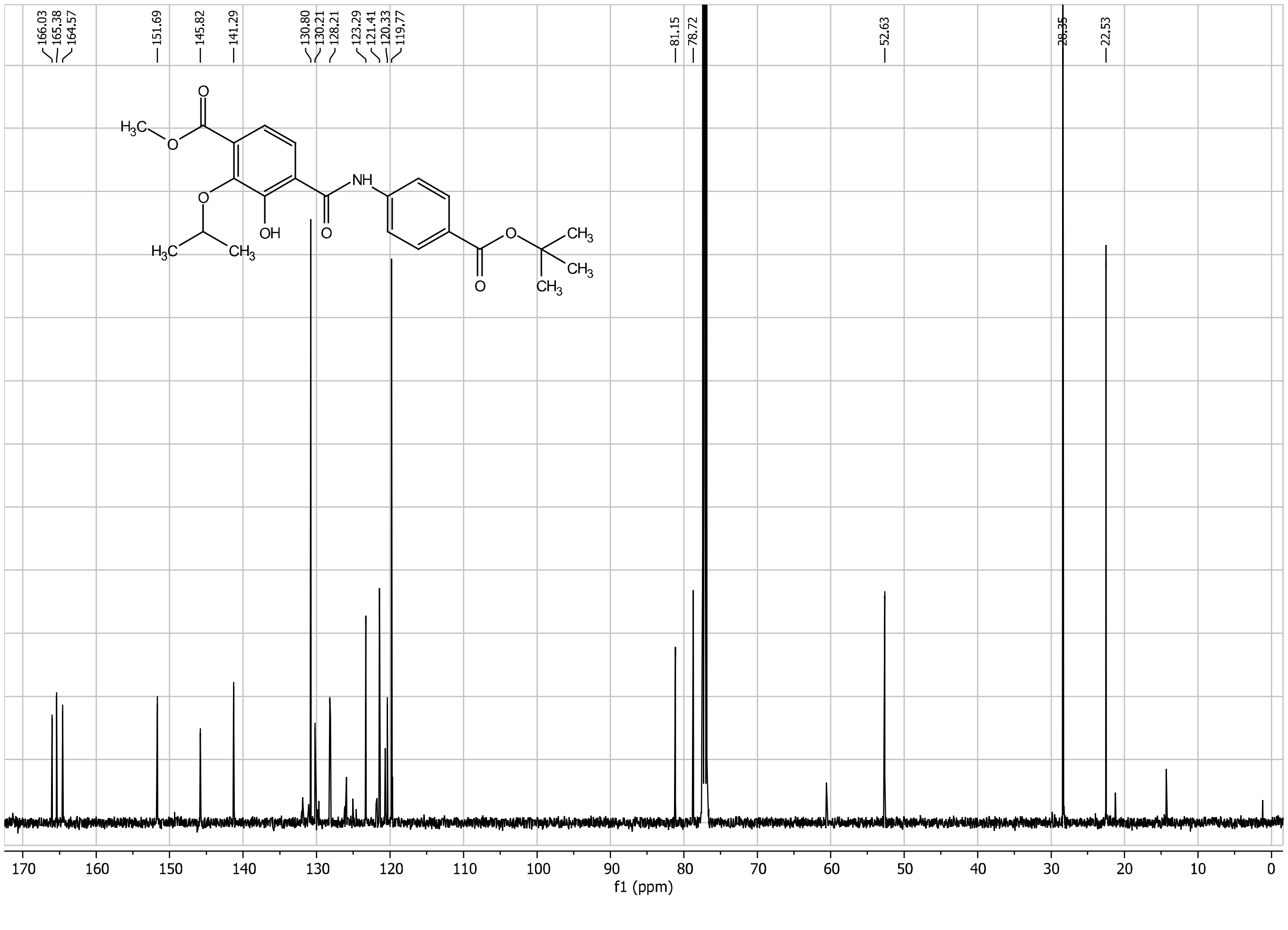
166.03
165.38
164.57
151.69
145.82
141.29
130.80
130.21
128.21
123.29
121.41
120.33
119.77
81.15
78.72
52.63
28.35
22.53
f1 (ppm)

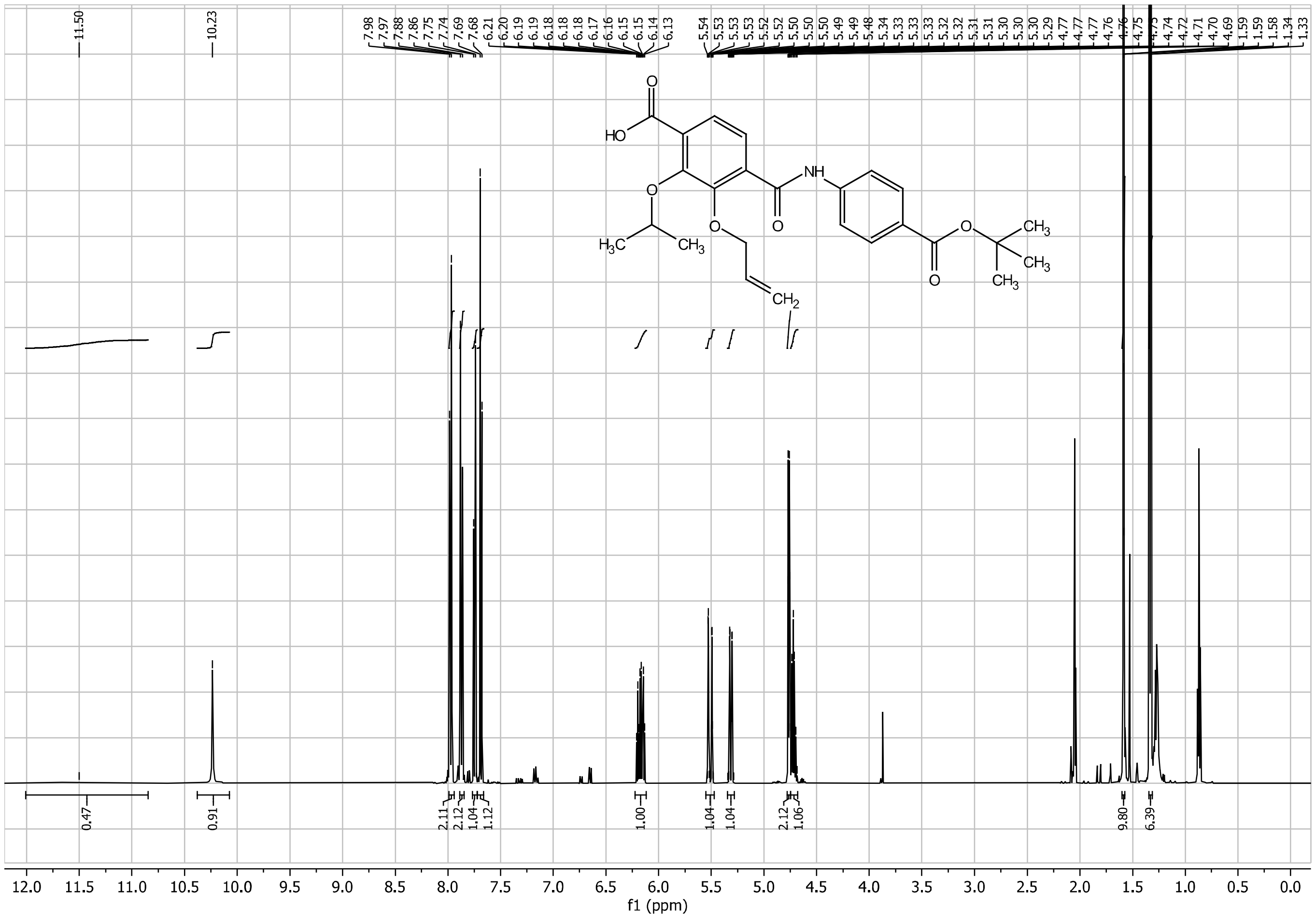
11.50
10.23
7.98 7.97 7.88 7.86 7.75 7.74 7.69 7.68 6.21 6.20 6.19 6.19 6.18 6.18 6.17 6.16 6.15 6.15 6.14 6.13
5.54 5.53 5.53 5.53 5.52 5.52 5.50 5.50 5.50 5.50 5.49 5.49 5.48 5.34 5.33 5.33 5.33 5.33 5.32 5.32 5.31 5.31 5.30 5.30 5.30 5.30 5.29 4.77 4.77 4.77 4.76 4.76 4.75 4.75 4.74 4.72 4.71 4.70 4.69 1.59 1.59 1.58 1.34 1.33
HO
O
NH
H3C
CH3
CH3
CH3
CH3
CH2
0.47
0.91
2.11 2.12 1.04 1.12
1.00
1.04 1.04
2.12 1.06
9.80 6.39
12.0 11.5 11.0 10.5 10.0 9.5 9.0 8.5 8.0 7.5 7.0 6.5 6.0 5.5 5.0 4.5 4.0 3.5 3.0 2.5 2.0 1.5 1.0 0.5 0.0
f1 (ppm)

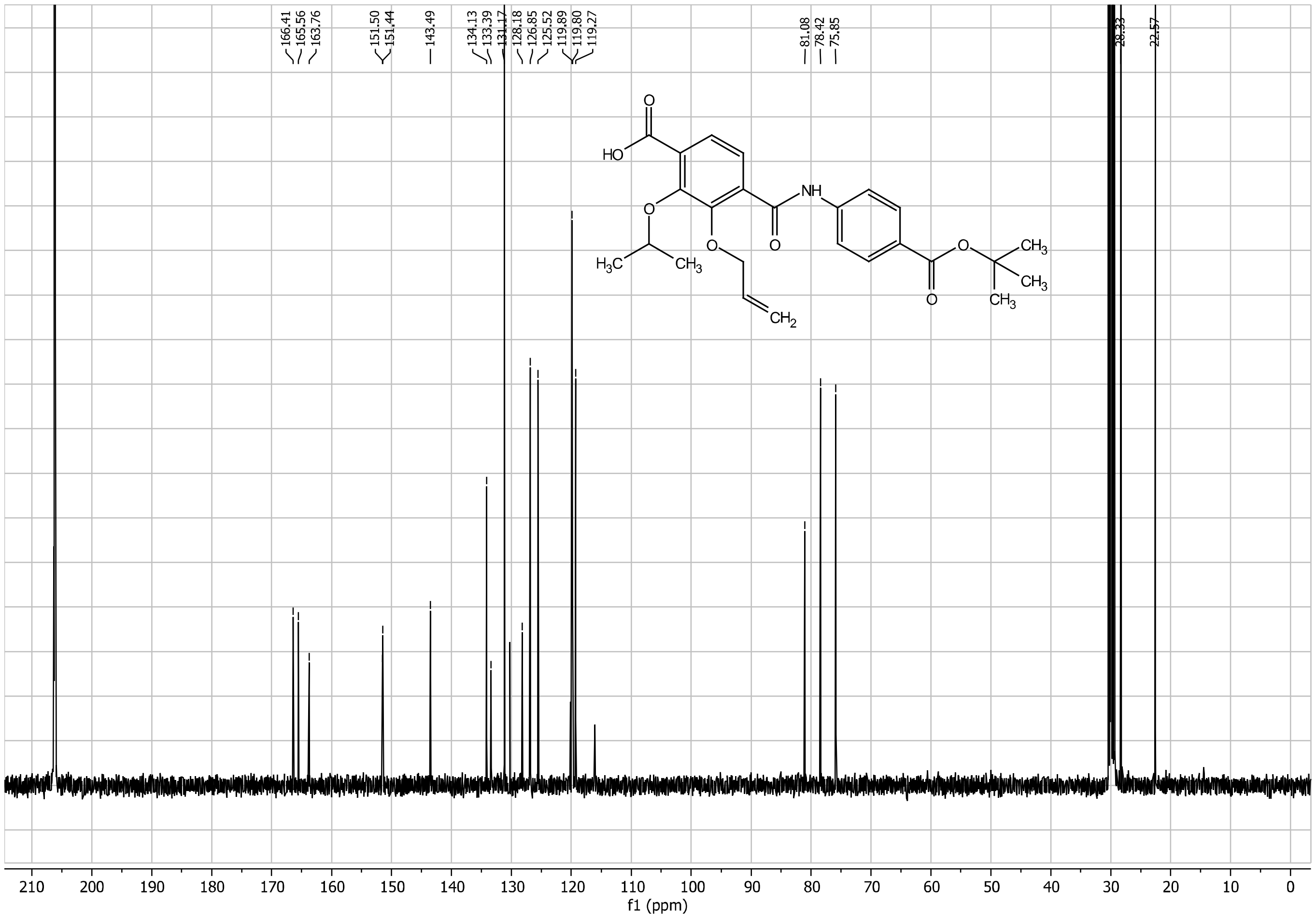
166.41 165.56 163.76
151.50 151.44
143.49
134.13 133.39 131.17 128.18 126.85 125.52 119.89 119.80 119.27
81.08 78.42 75.85
28.33
22.57
HO
O
NH
H3C
CH3
CH3
CH3
CH3
CH2
210 200 190 180 170 160 150 140 130 120 110 100 90 80 70 60 50 40 30 20 10 0
f1 (ppm)

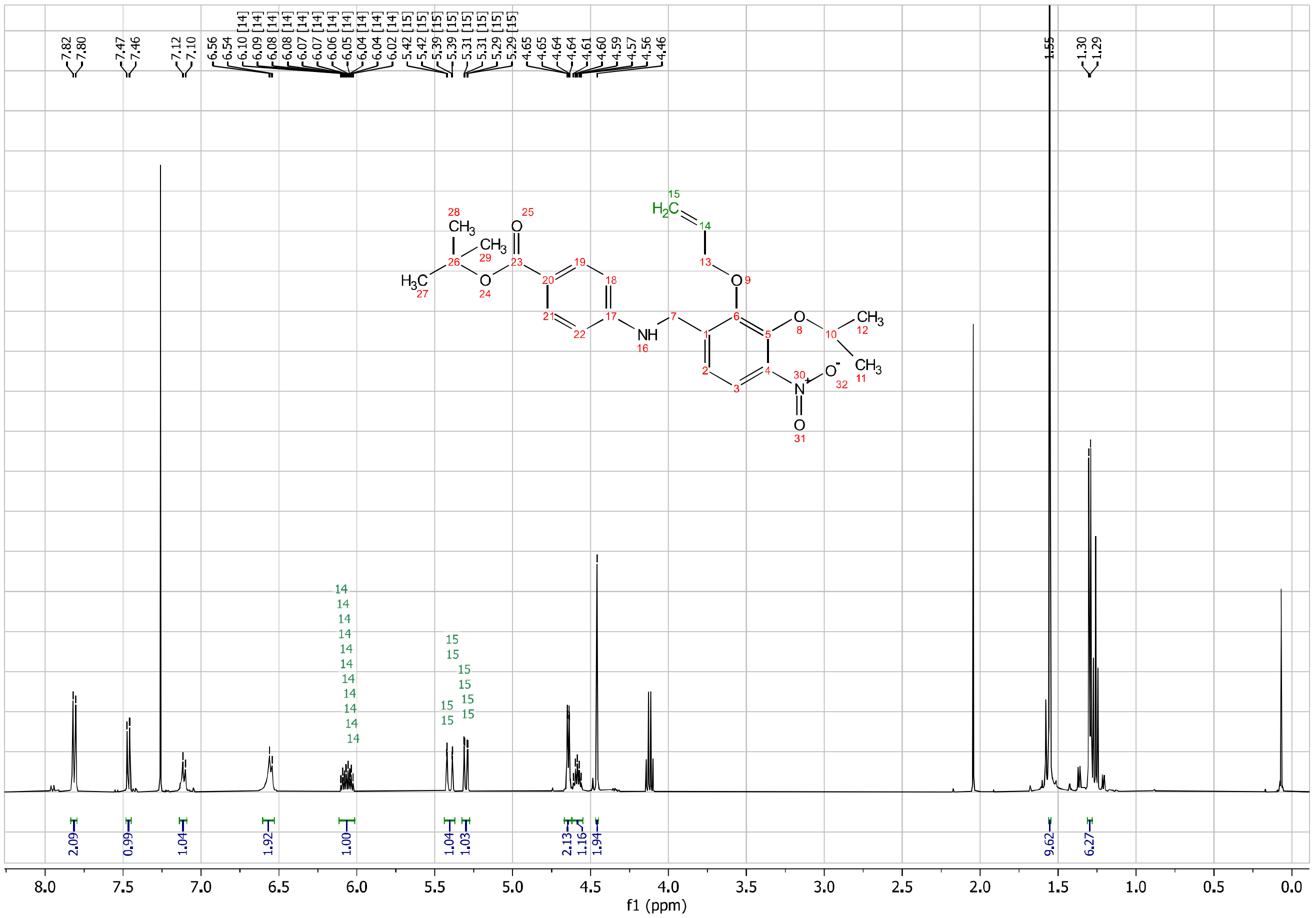

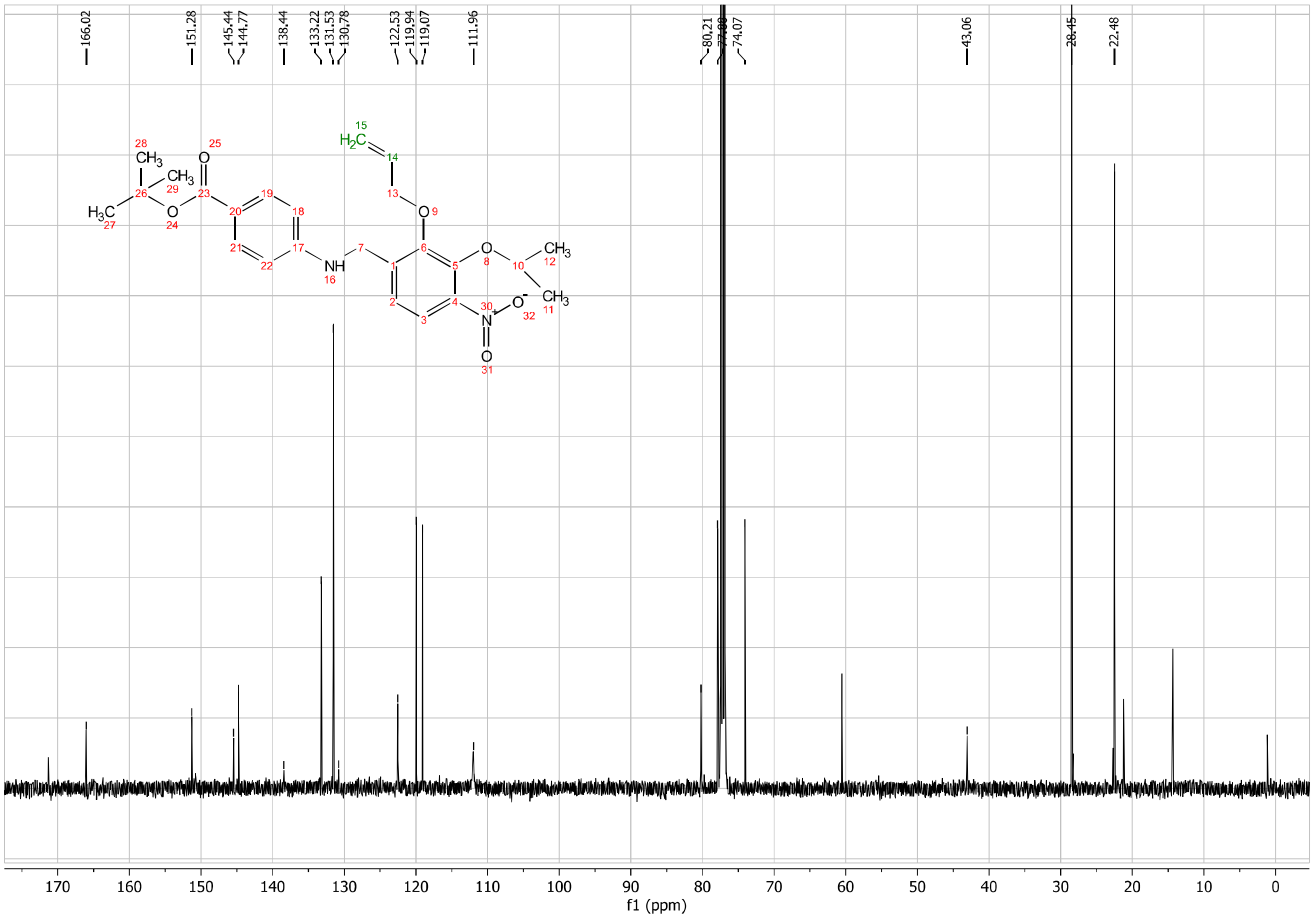

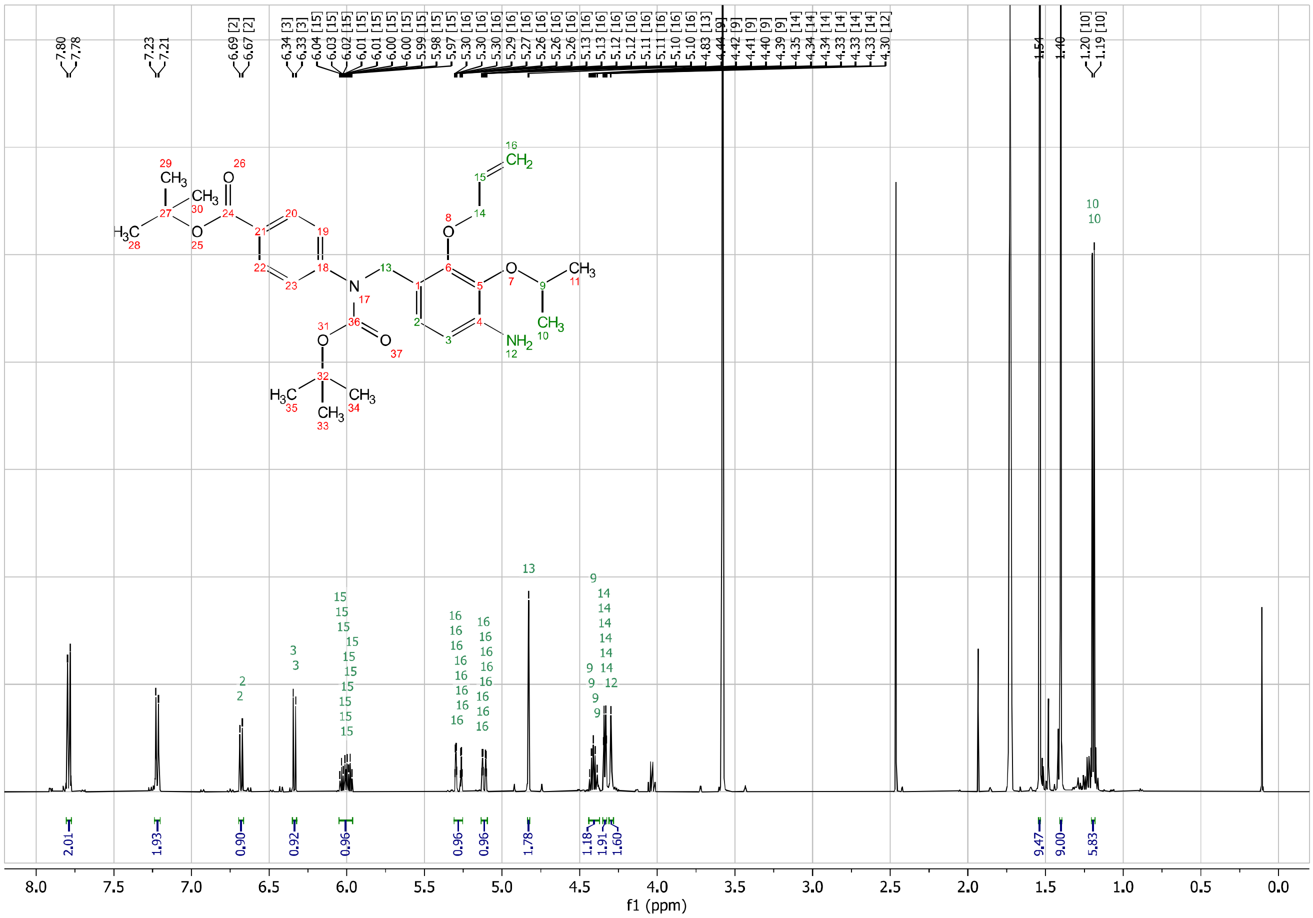

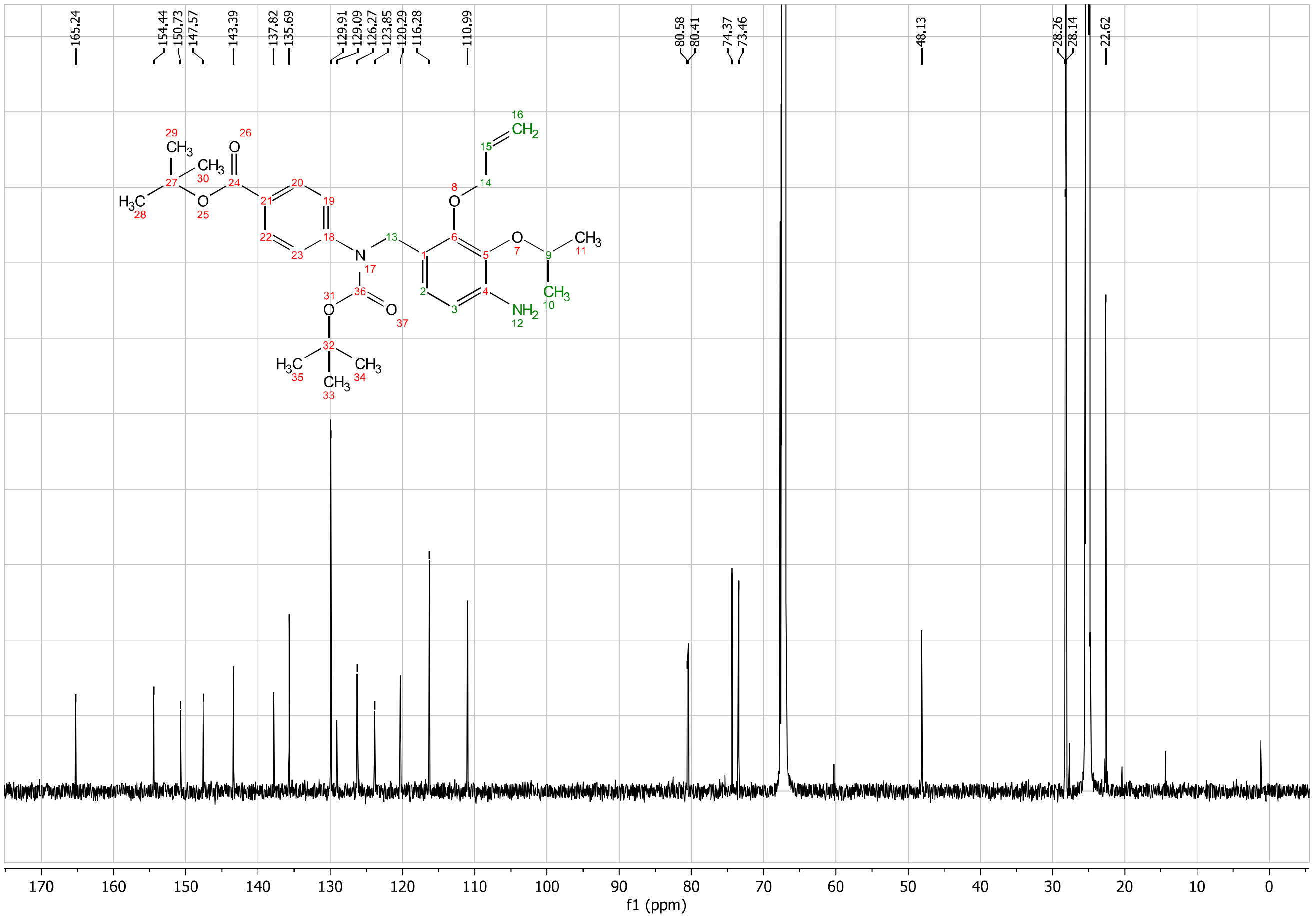

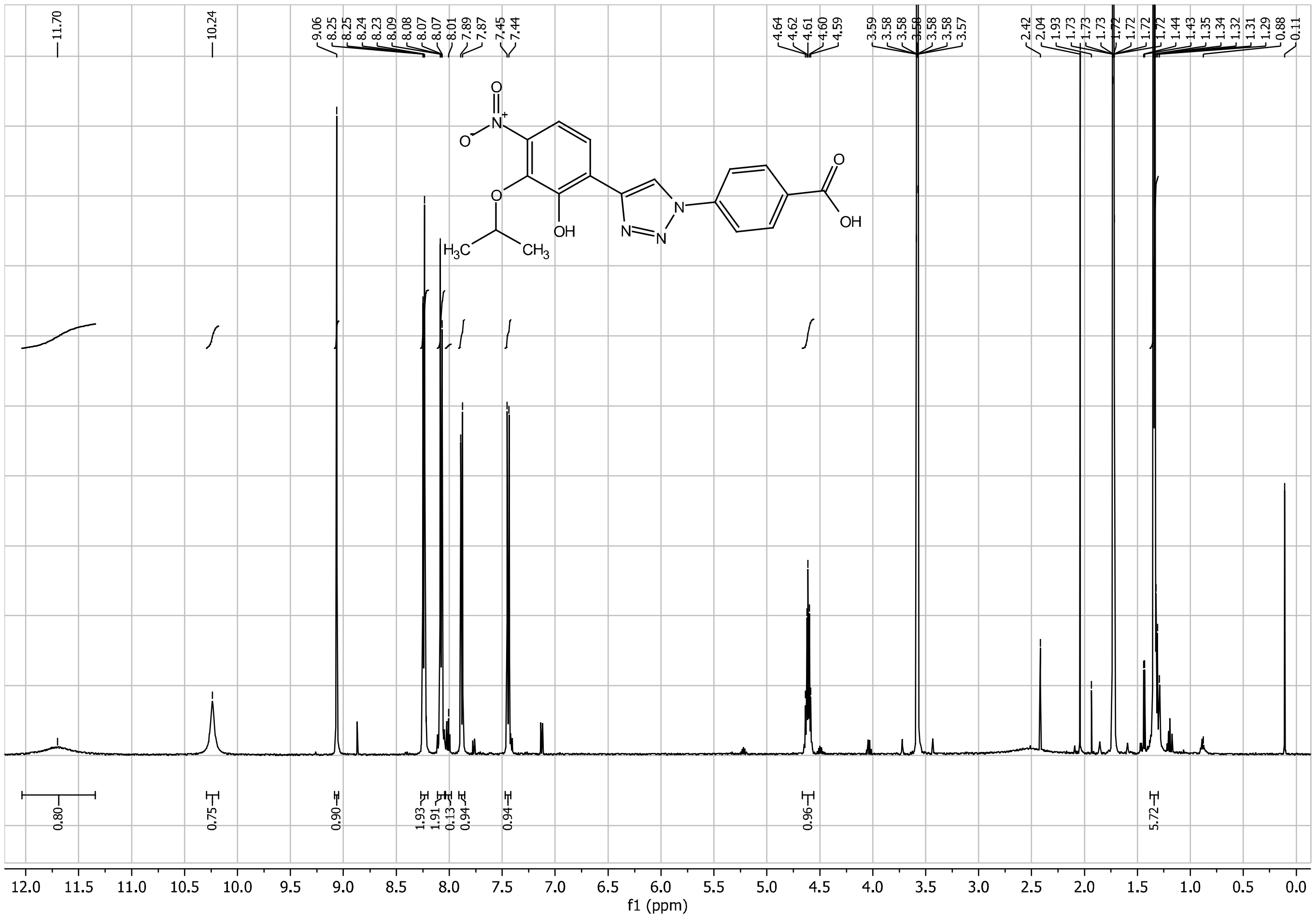
11.70
10.24
9.06
8.25
8.25
8.24
8.23
8.09
8.08
8.07
8.07
8.01
7.89
7.87
7.45
7.44
4.64
4.62
4.61
4.60
4.59
3.59
3.58
3.58
3.58
3.58
3.58
3.57
2.42
2.04
1.93
1.73
1.73
1.73
1.72
1.72
1.72
1.72
1.44
1.43
1.35
1.34
1.32
1.31
1.29
0.88
0.11
OH
N
N
N
OH
O
H_3C
CH_3
0.80
0.75
0.90
1.93
1.91
0.13
0.94
0.94
0.96
5.72
f1 (ppm)

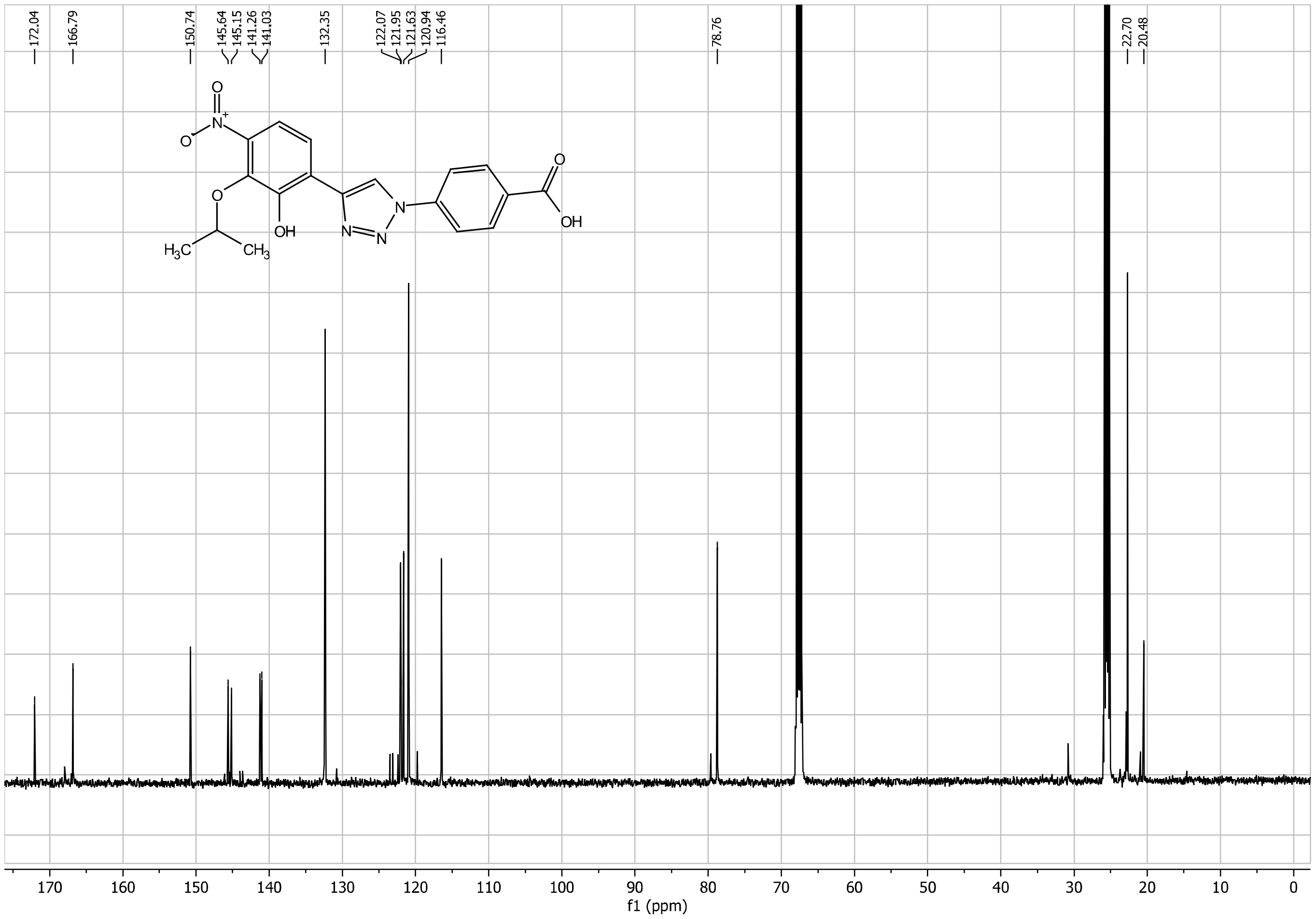
172.04
166.79
150.74
145.64
145.15
141.26
141.03
132.35
122.07
121.95
121.63
120.94
116.46
78.76
22.70
20.48
OH
N
N
N
OH
O
H_3C
CH_3
f1 (ppm)

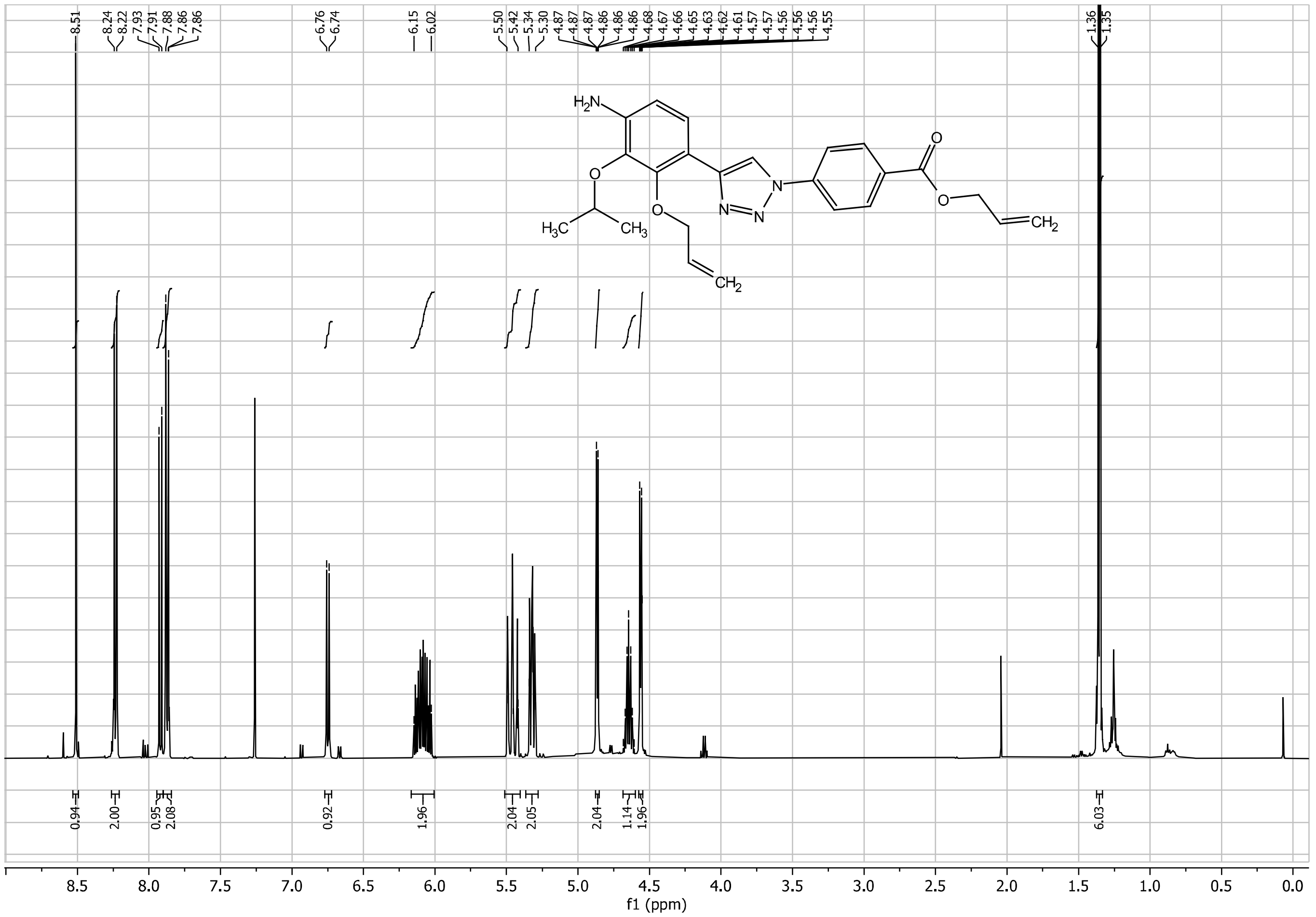

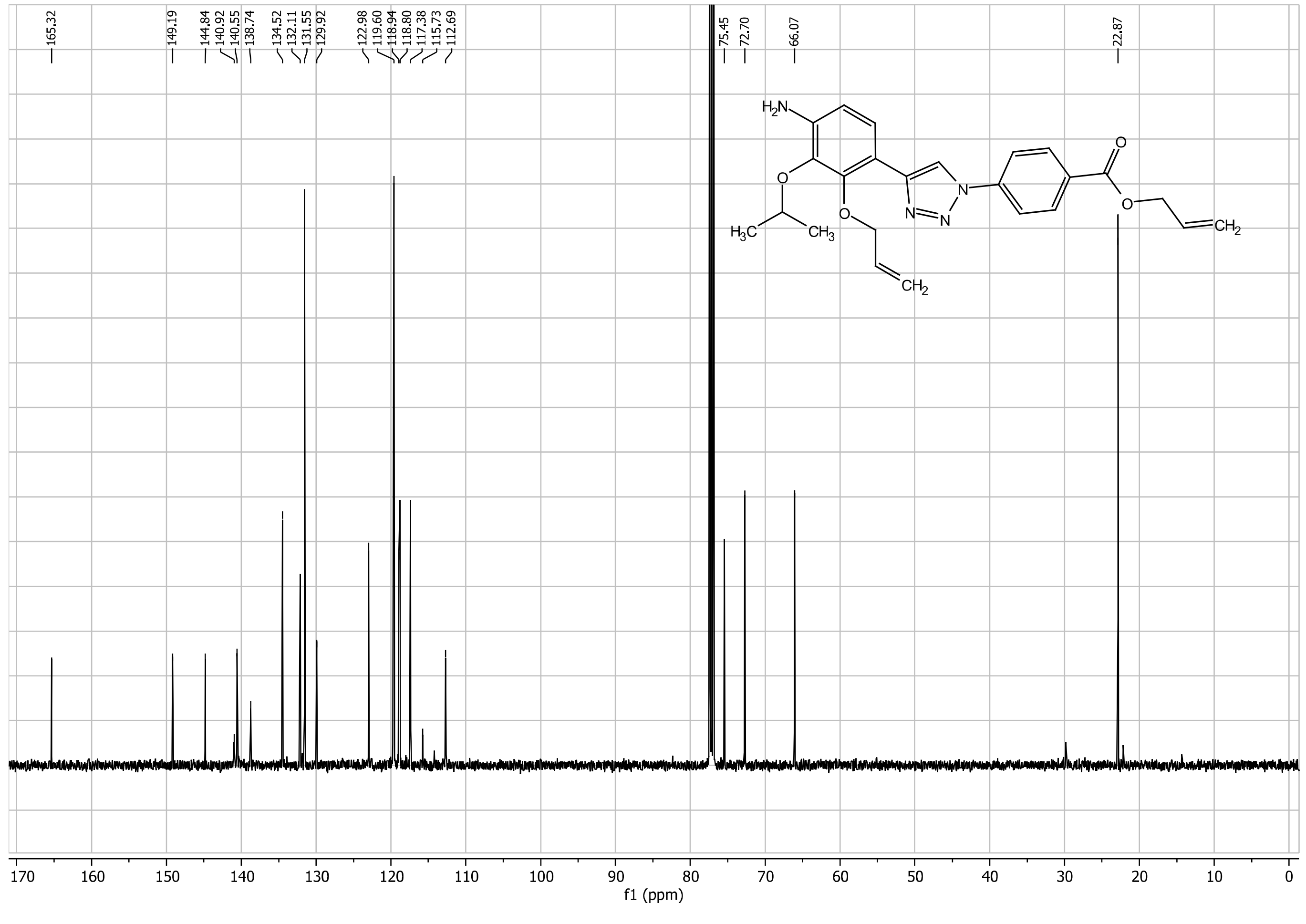

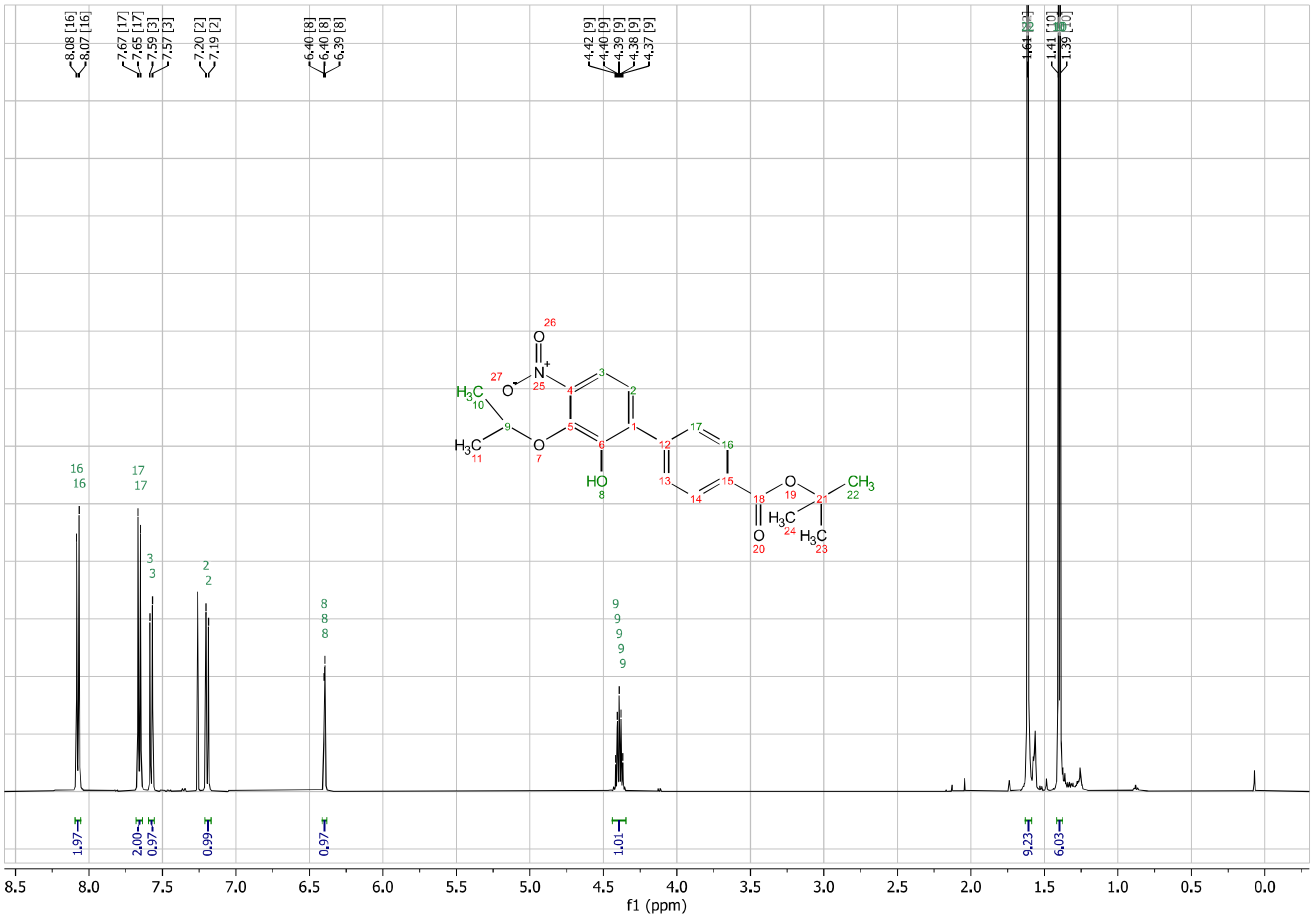
8.08 [16]
8.07 [16]
7.67 [17]
7.65 [17]
7.59 [3]
7.57 [3]
7.20 [2]
7.19 [2]
6.40 [8]
6.40 [8]
6.39 [8]
4.42 [9]
4.40 [9]
4.39 [9]
4.38 [9]
4.37 [9]
1.41 [10]
1.39 [10]
1.97
2.00
0.97
0.99
0.97
1.01
9.23
6.03
f1 (ppm)

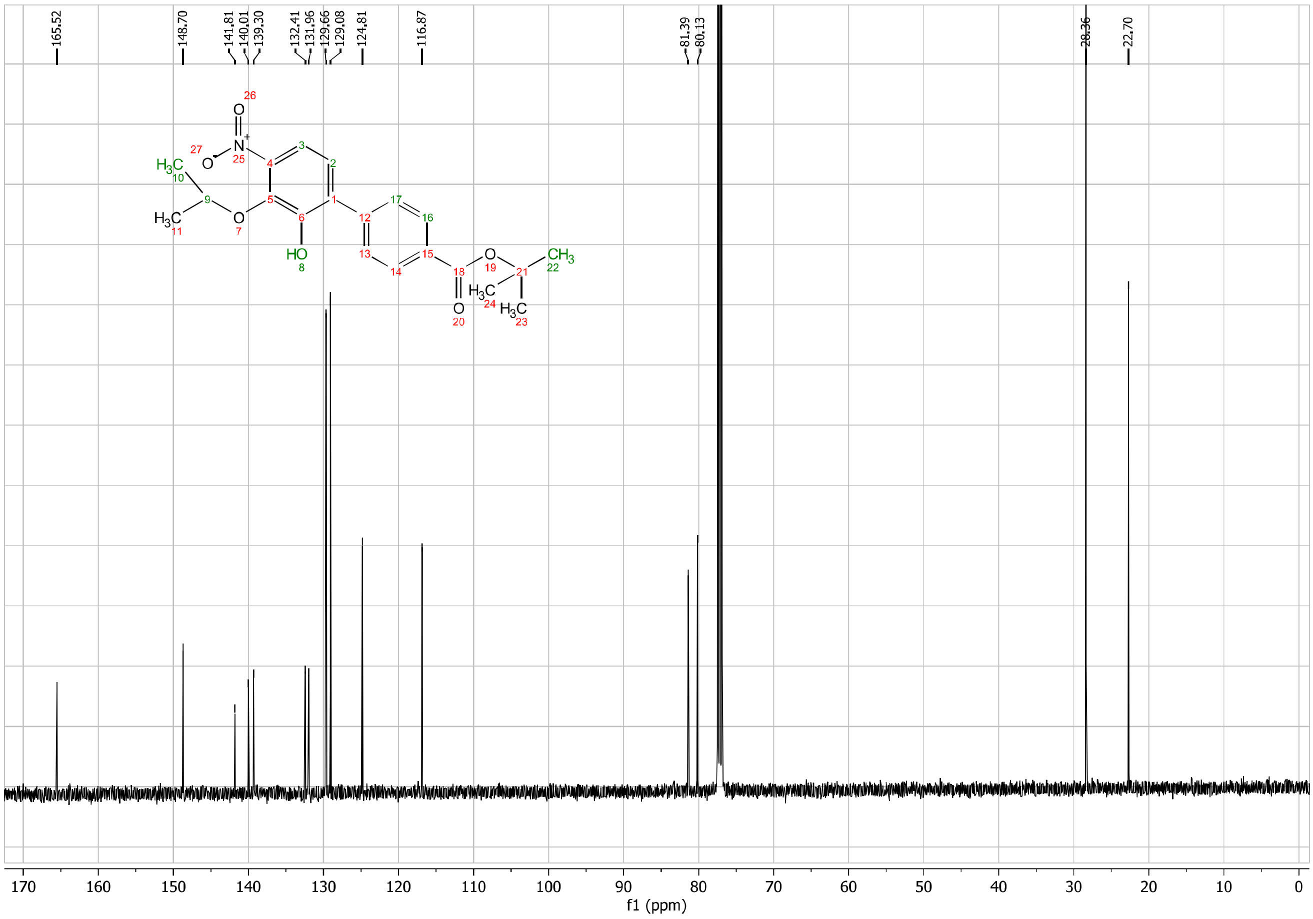
165.52
148.70
141.81
140.01
139.30
132.41
131.96
129.66
129.08
124.81
116.87
81.39
80.13
28.36
22.70
f1 (ppm)

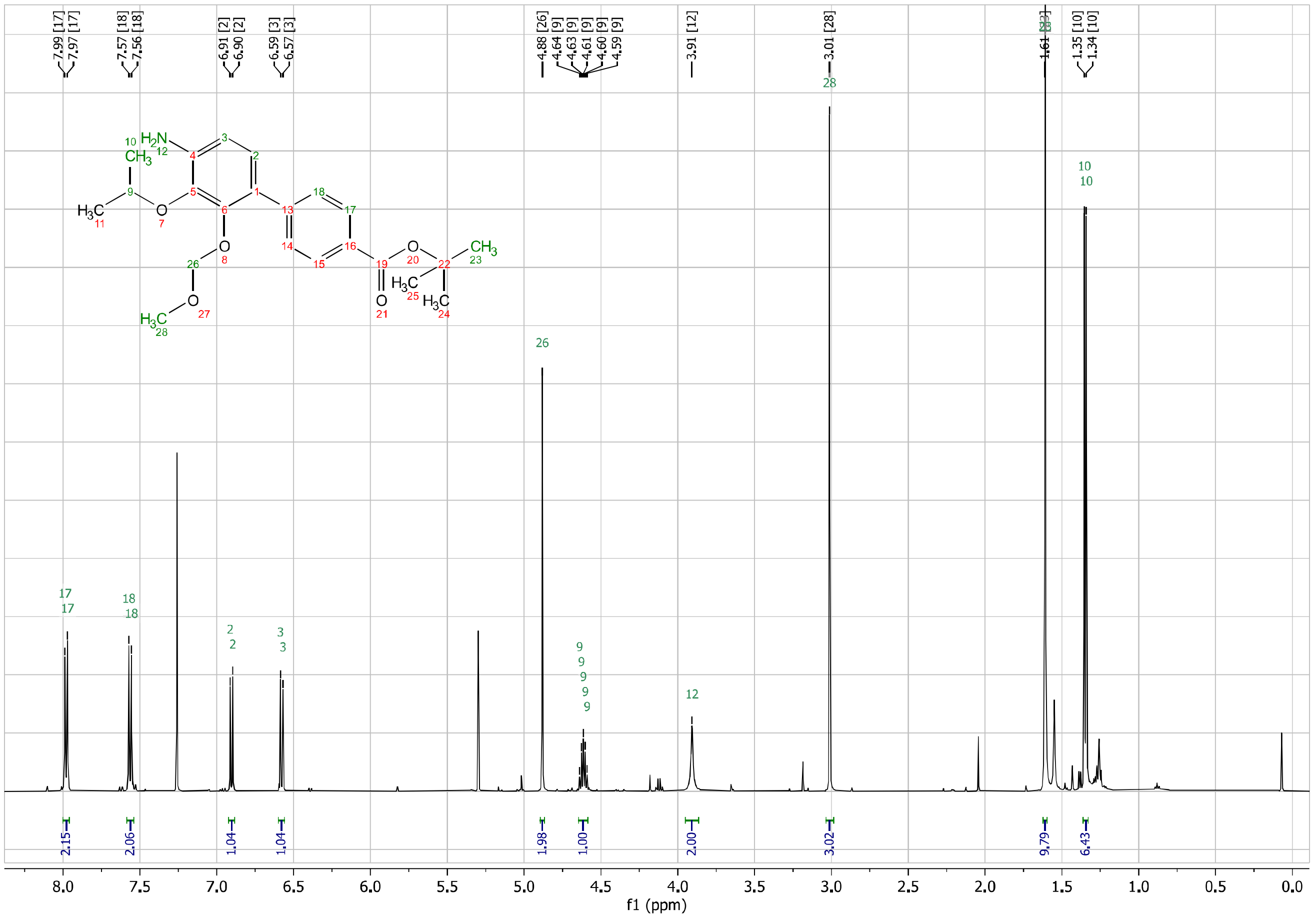
7.99 [17]
7.97 [17]
7.57 [18]
7.56 [18]
6.91 [2]
6.90 [2]
6.59 [3]
6.57 [3]
4.88 [26]
4.64 [9]
4.63 [9]
4.61 [9]
4.60 [9]
4.59 [9]
3.91 [12]
3.01 [28]
1.61
1.35 [10]
1.34 [10]
2.15
2.06
1.04
1.04
1.98
1.00
2.00
3.02
9.79
6.43
f1 (ppm)

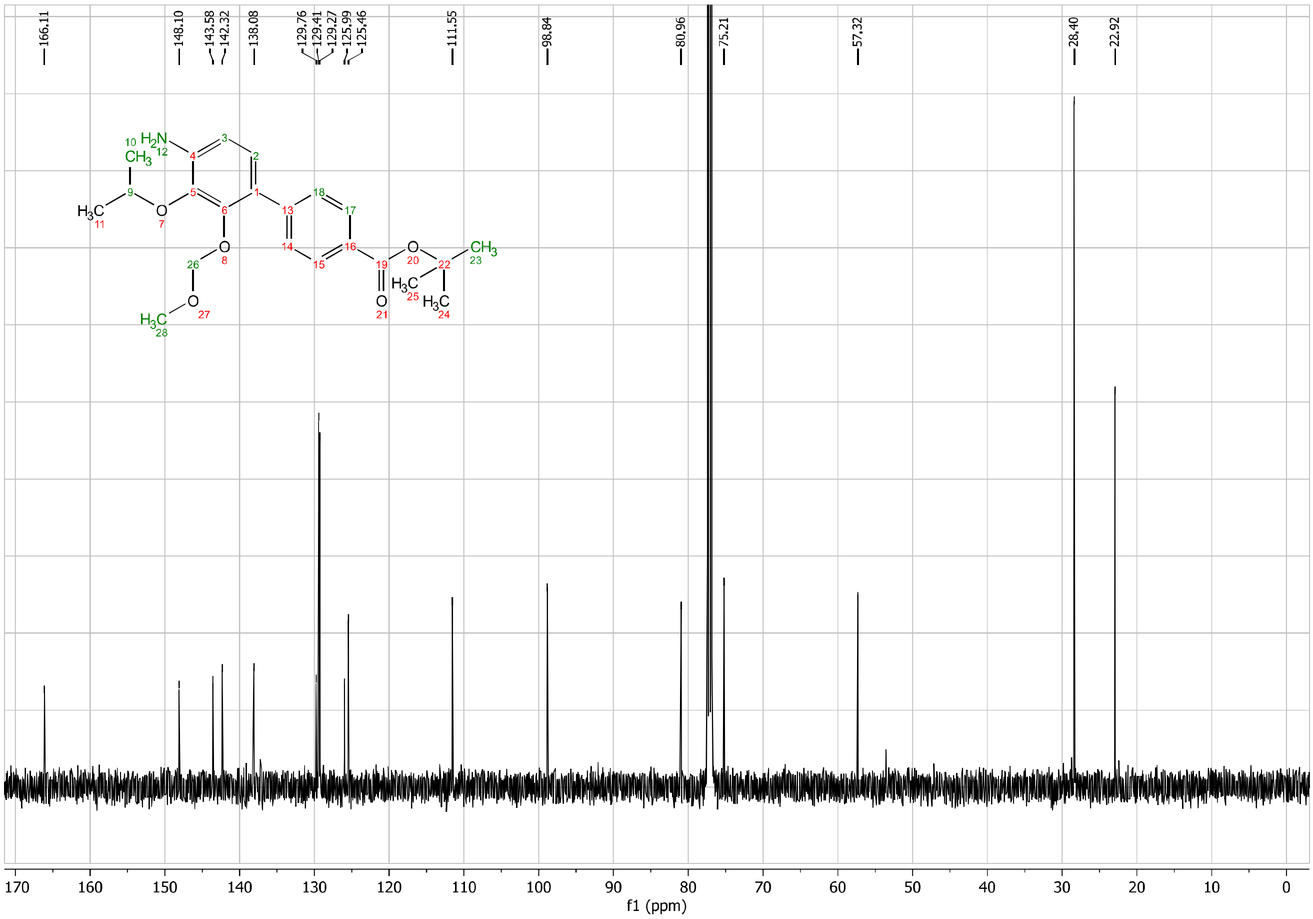
166.11
148.10
143.58
142.32
138.08
129.76
129.41
129.27
125.99
125.46
111.55
98.84
80.96
75.21
57.32
28.40
22.92
f1 (ppm)

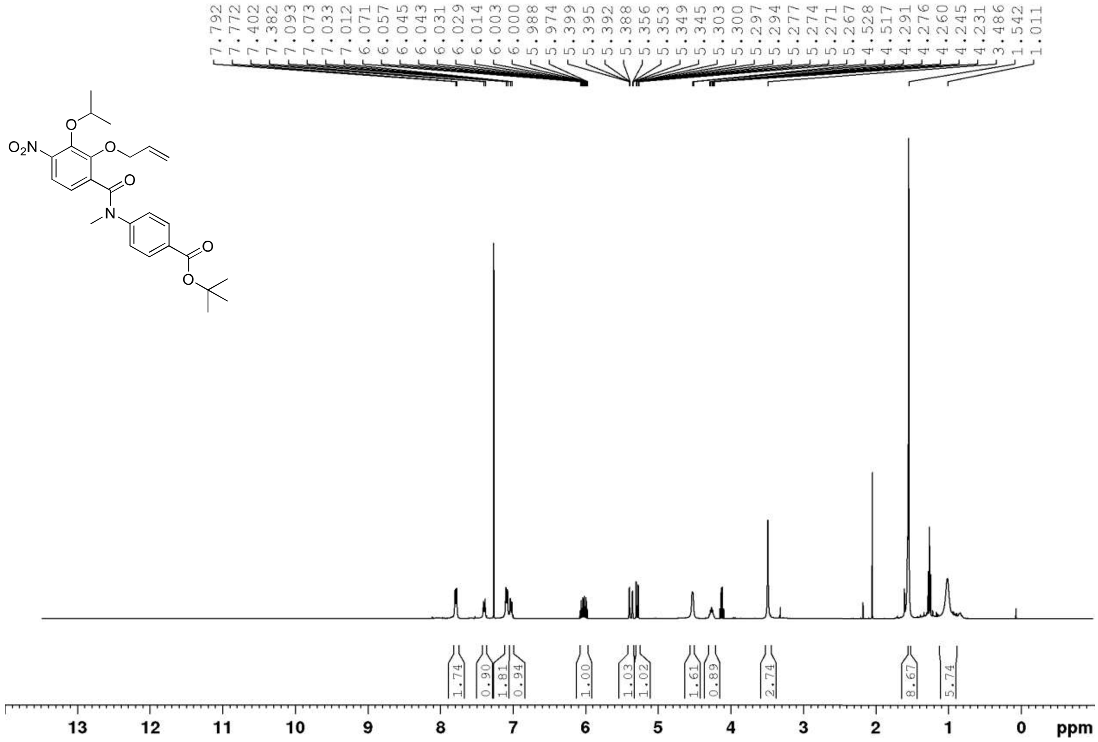
7.792 7.772 7.402 7.382 7.093 7.073 7.033 7.012 6.071 6.057 6.045 6.043 6.031 6.029 6.014 6.003 6.000 5.988 5.974 5.399 5.395 5.392 5.388 5.356 5.353 5.349 5.345 5.303 5.300 5.297 5.294 5.277 5.274 5.271 5.267 4.528 4.517 4.291 4.276 4.260 4.245 4.231 3.486 1.542 1.011
O_2N
O
N
1.74 0.90 1.81 0.94 1.00 1.03 1.02 1.61 0.89 2.74 8.67 5.74
13 12 11 10 9 8 7 6 5 4 3 2 1 0 ppm

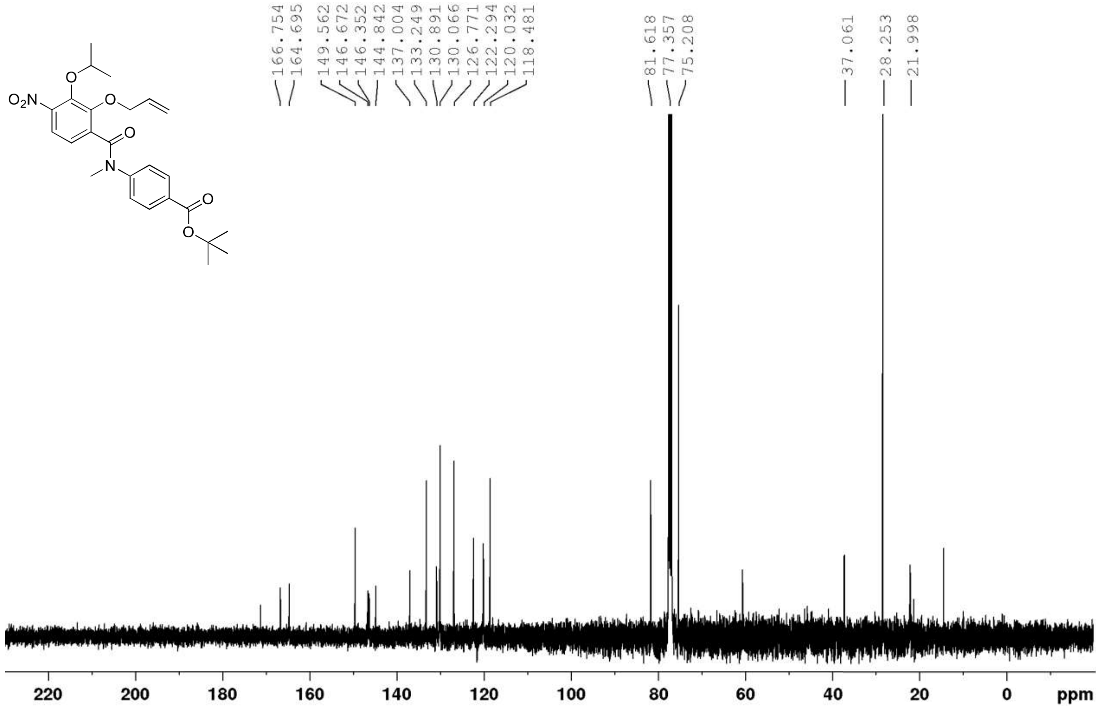
166.754 164.695 149.562 146.672 146.352 144.842 137.004 133.249 130.891 130.066 126.771 122.294 120.032 118.481 81.618 77.357 75.208 37.061 28.253 21.998
O_2N
O
N
220 200 180 160 140 120 100 80 60 40 20 0 ppm

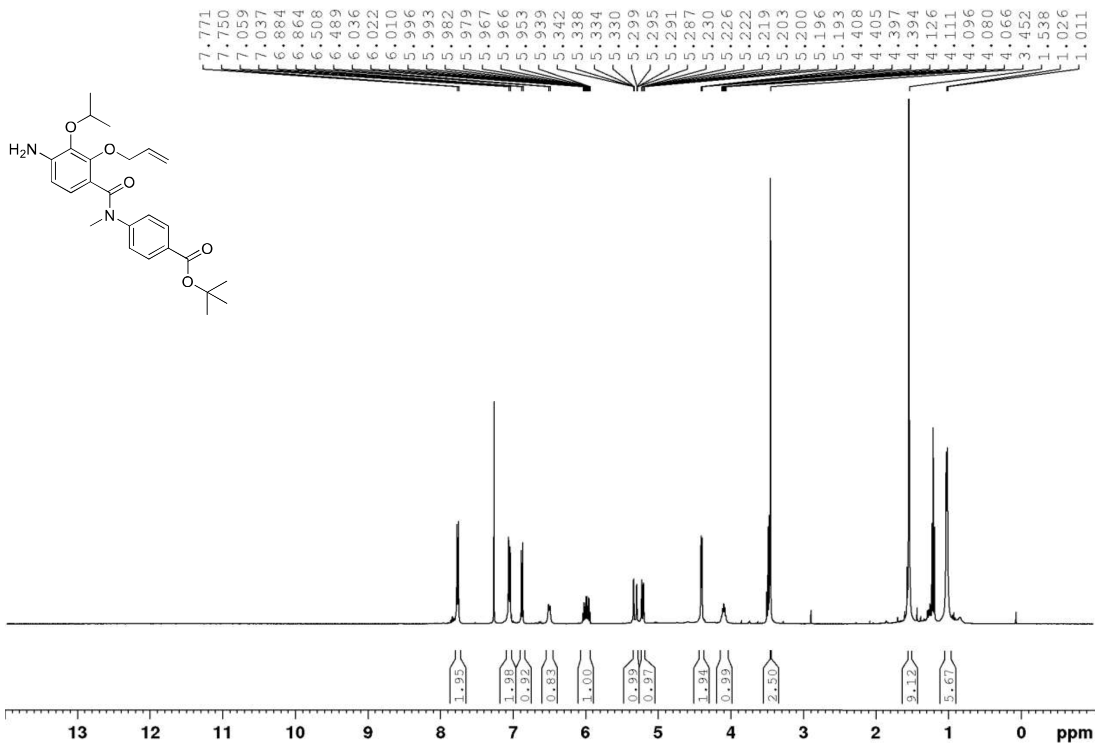

H2N
O
O
N
7.771
7.750
7.059
7.037
6.884
6.864
6.508
6.489
6.036
6.022
6.010
5.996
5.993
5.982
5.979
5.967
5.966
5.953
5.939
5.342
5.338
5.334
5.330
5.299
5.295
5.291
5.287
5.230
5.226
5.222
5.219
5.203
5.200
5.196
5.193
4.408
4.405
4.397
4.394
4.126
4.111
4.096
4.080
4.066
3.452
1.538
1.026
1.011
1.95
1.98
0.92
0.83
1.00
0.99
0.97
1.94
0.99
2.50
9.12
5.67
13
12
11
10
9
8
7
6
5
4
3
2
1
0
ppm

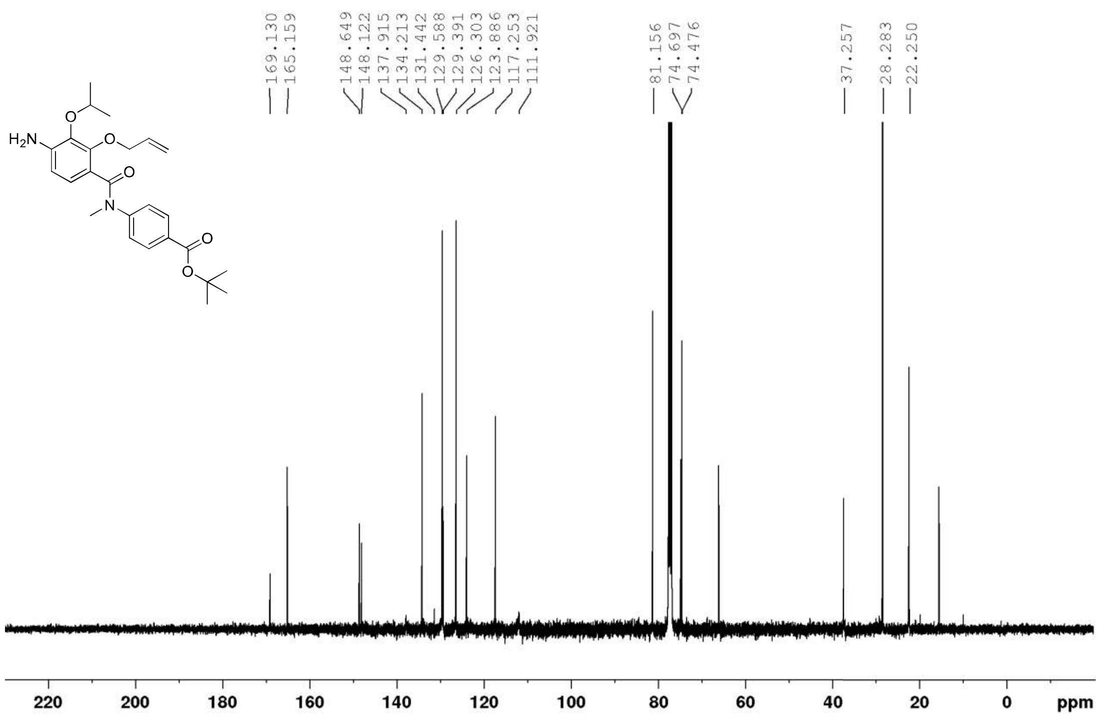

H2N
O
O
N
169.130
165.159
148.649
148.122
137.915
134.213
131.442
129.588
129.391
126.303
123.886
117.253
111.921
81.156
74.697
74.476
37.257
28.283
22.250
220
200
180
160
140
120
100
80
60
40
20
0
ppm

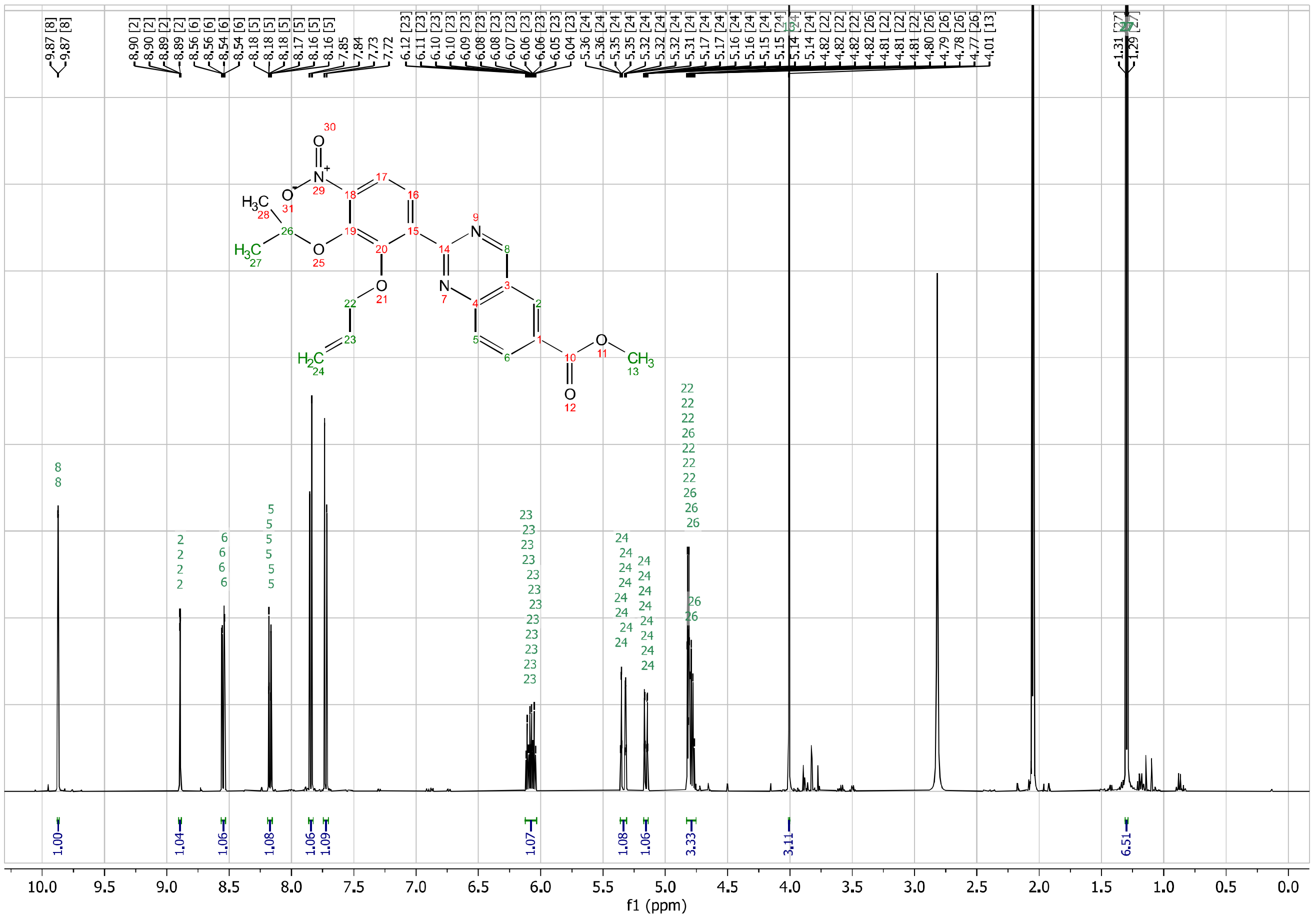

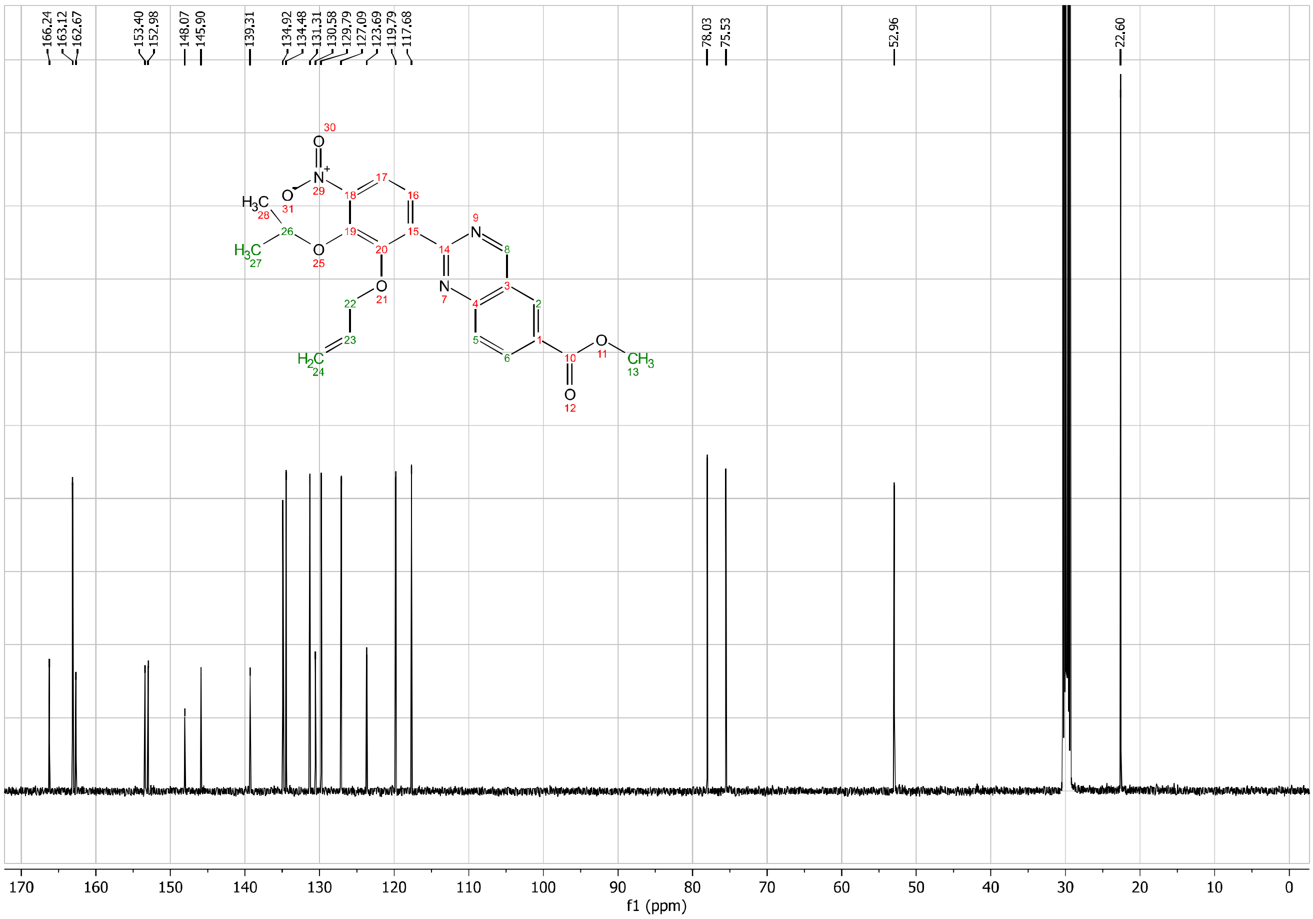

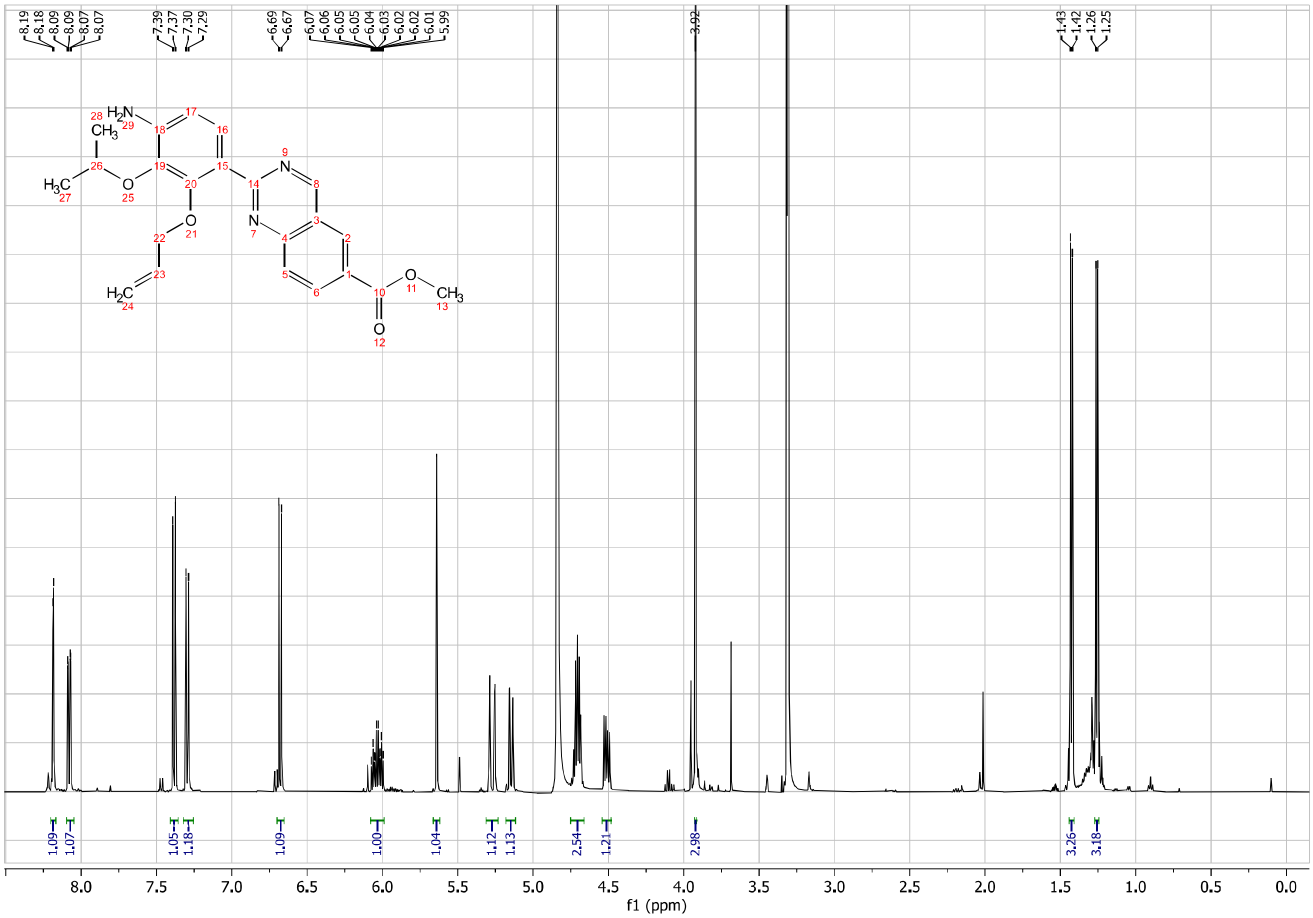
8.19
8.18
8.09
8.09
8.07
8.07
7.39
7.37
7.30
7.29
6.69
6.67
6.07
6.06
6.05
6.05
6.04
6.03
6.02
6.02
6.01
5.99
3.92
1.43
1.42
1.26
1.25
1.09
1.07
1.05
1.18
1.09
1.00
1.04
1.12
1.13
2.54
1.21
2.98
3.26
3.18
f1 (ppm)

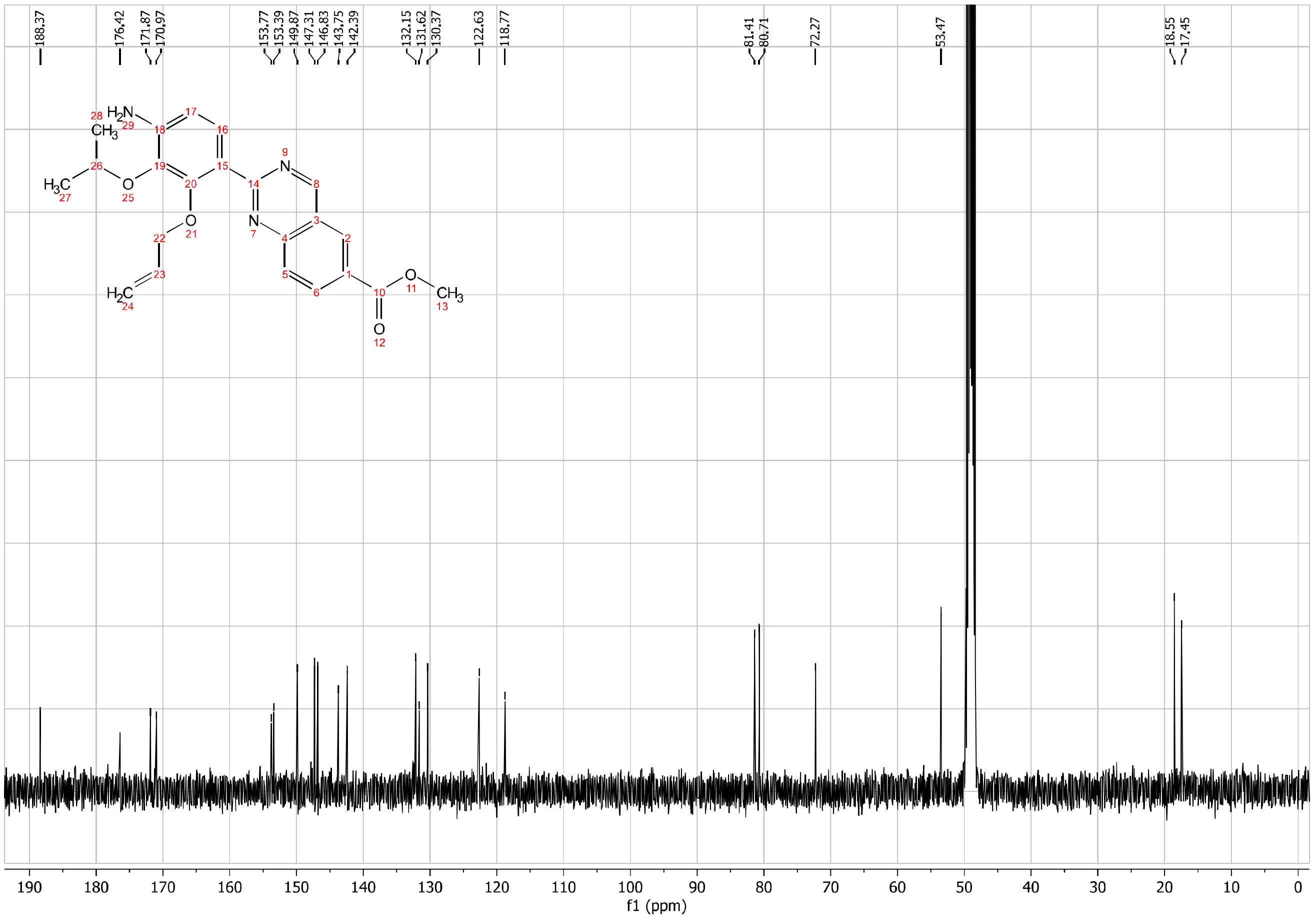
188.37
176.42
171.87
170.97
153.77
153.39
149.87
147.31
146.83
143.75
142.39
132.15
131.62
130.37
122.63
118.77
81.41
80.71
72.27
53.47
18.55
17.45
f1 (ppm)

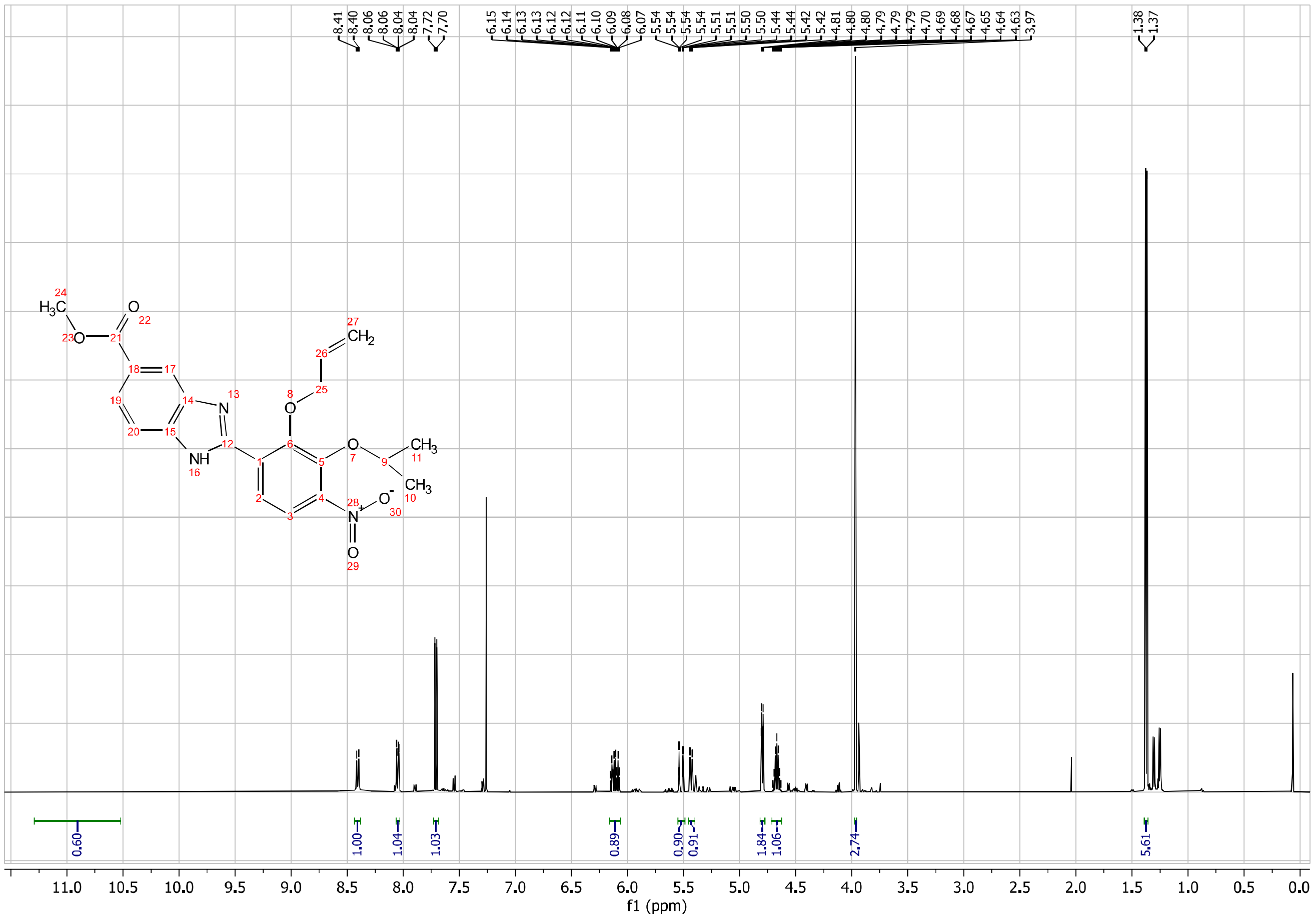
8.41
8.40
8.06
8.06
8.04
8.04
7.72
7.70
6.15
6.14
6.13
6.13
6.12
6.12
6.11
6.10
6.09
6.08
6.07
5.54
5.54
5.54
5.54
5.51
5.51
5.50
5.50
5.44
5.44
5.42
5.42
4.81
4.80
4.80
4.79
4.79
4.79
4.70
4.69
4.68
4.67
4.65
4.64
4.63
3.97
1.38
1.37
0.60
1.00
1.04
1.03
0.89
0.90
0.91
1.84
1.06
2.74
5.61
11.0
10.5
10.0
9.5
9.0
8.5
8.0
7.5
7.0
6.5
6.0
5.5
5.0
4.5
4.0
3.5
3.0
2.5
2.0
1.5
1.0
0.5
0.0
f1 (ppm)

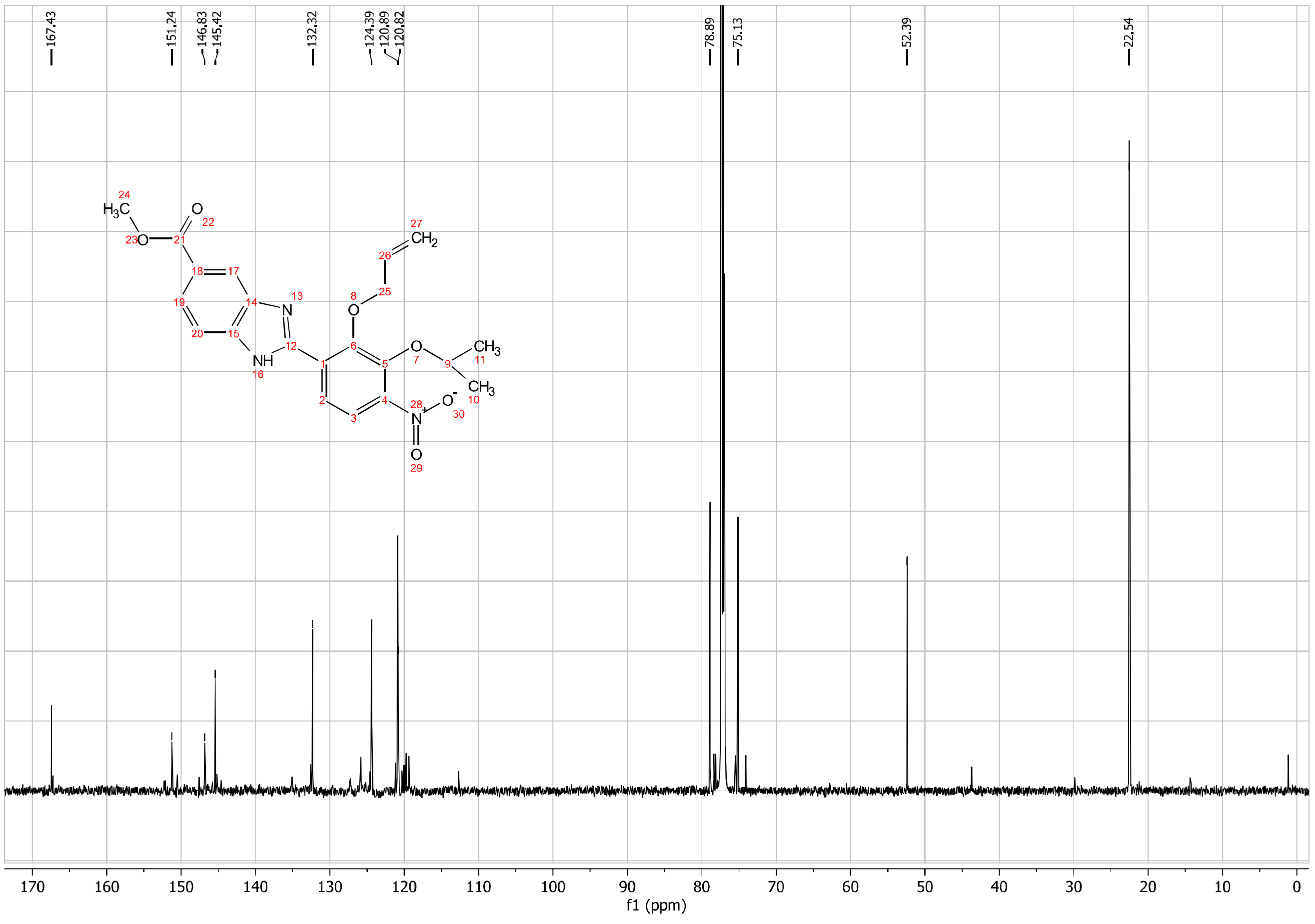
167.43
151.24
146.83
145.42
132.32
124.39
120.89
120.82
78.89
75.13
52.39
22.54
170
160
150
140
130
120
110
100
90
80
70
60
50
40
30
20
10
0
f1 (ppm)

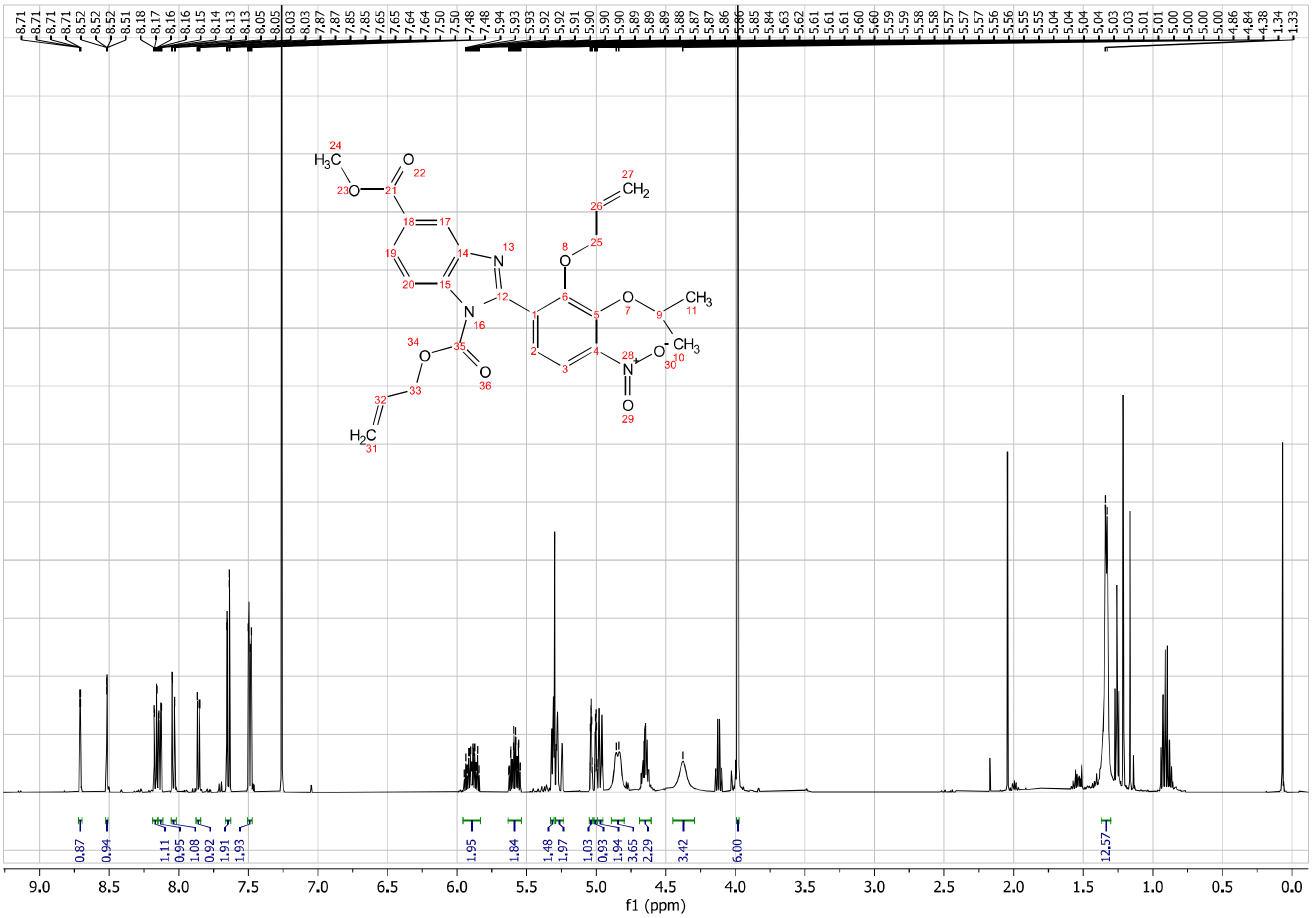
f1 (ppm)

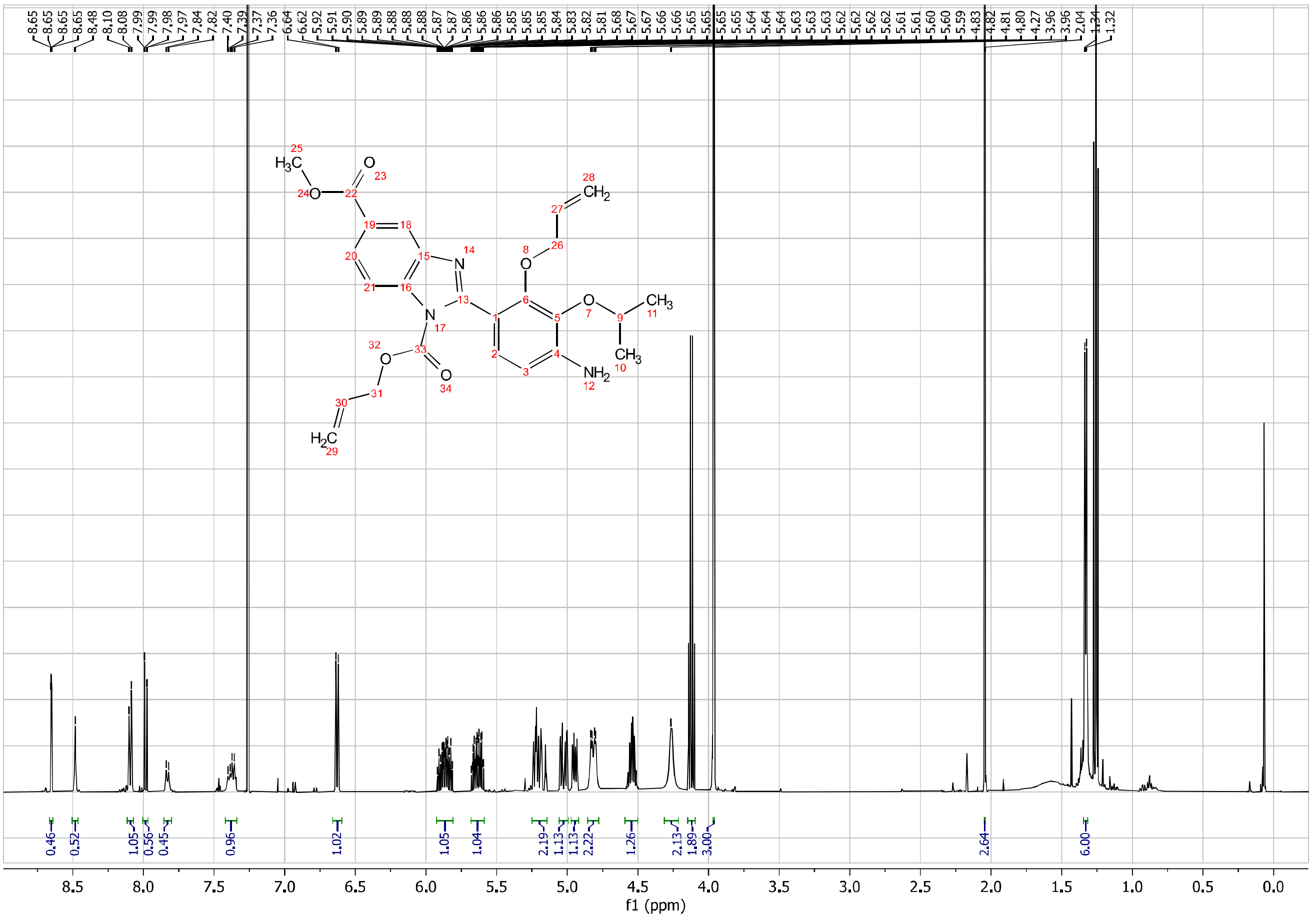
f1 (ppm)

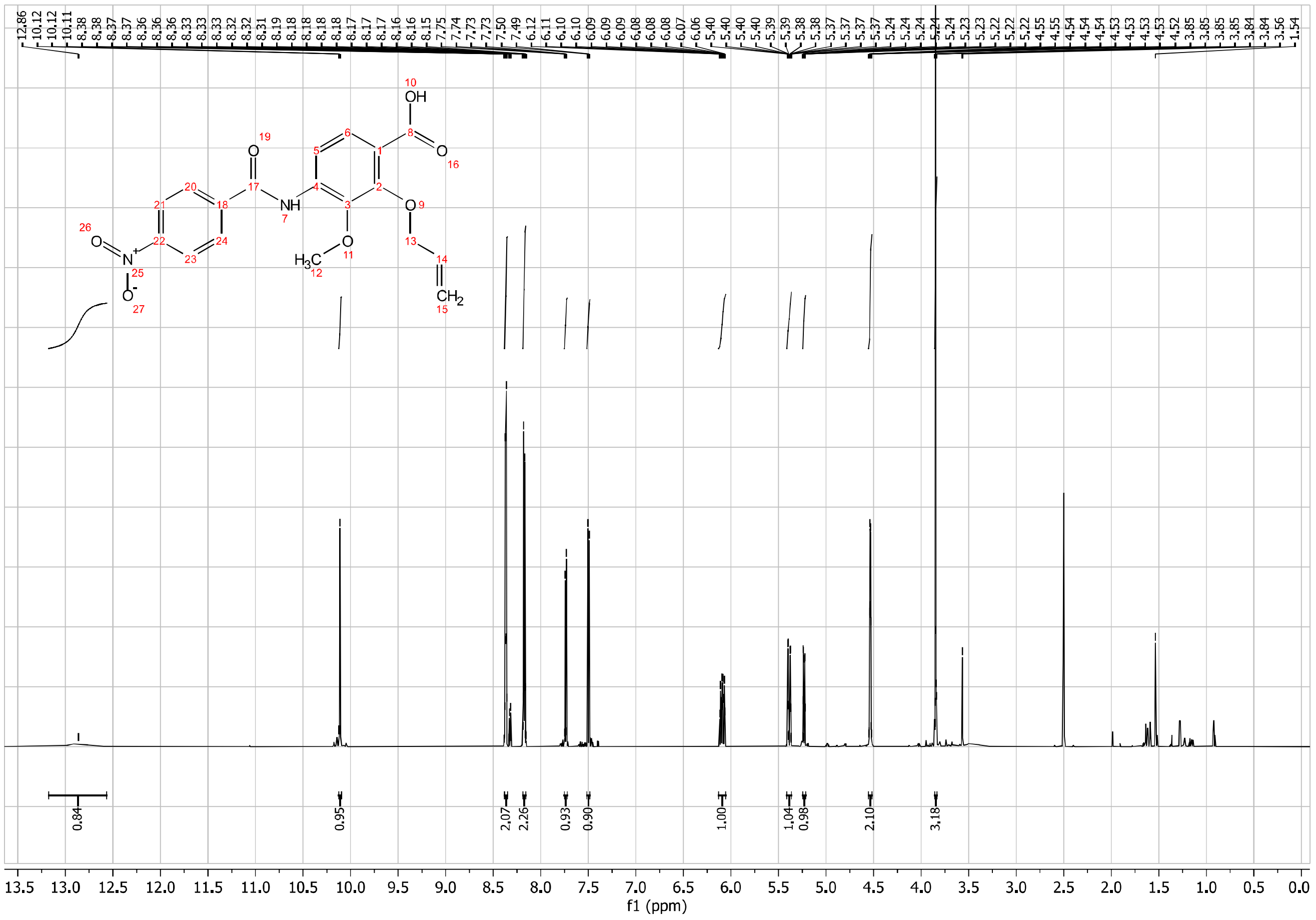

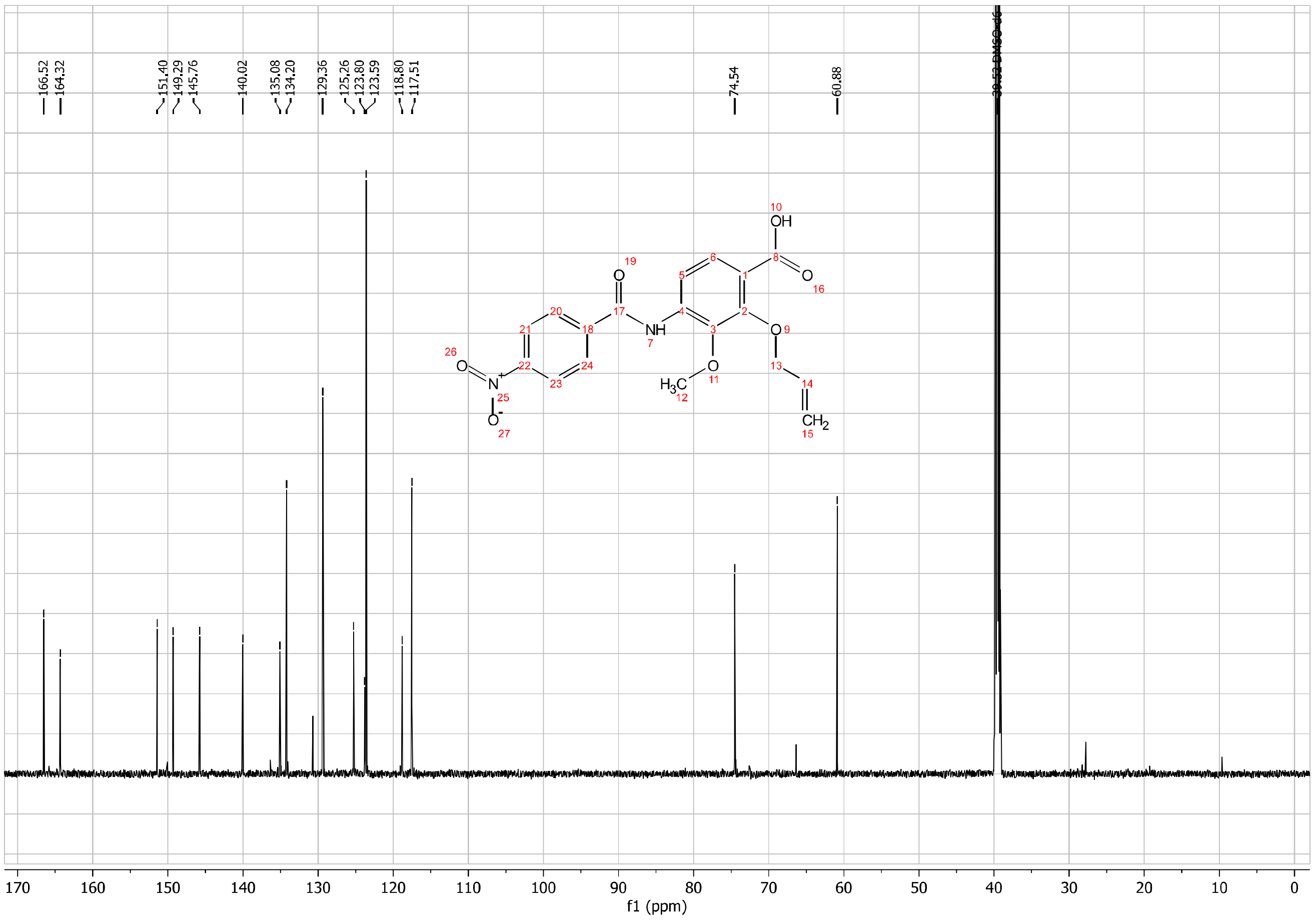

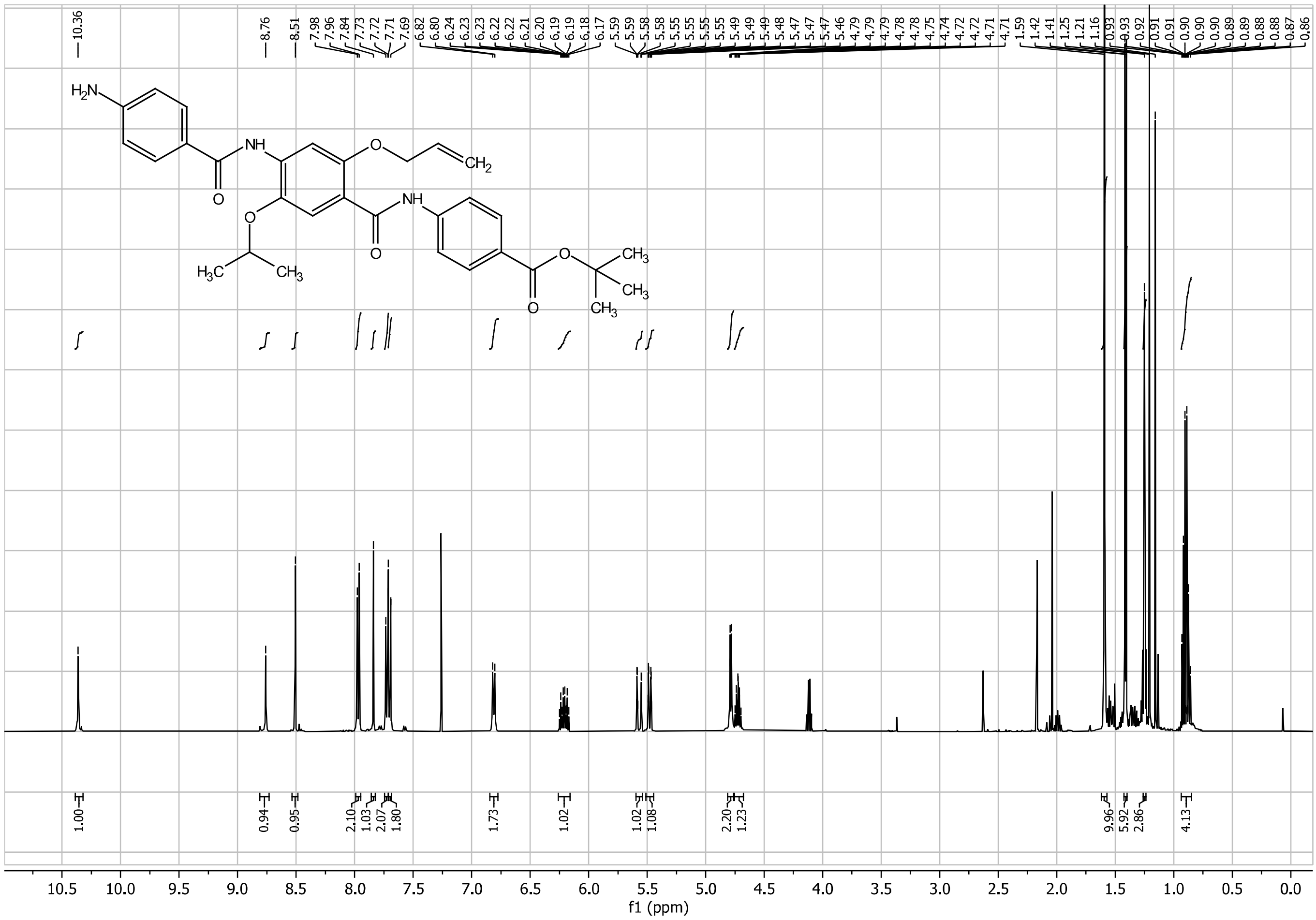
f1 (ppm)

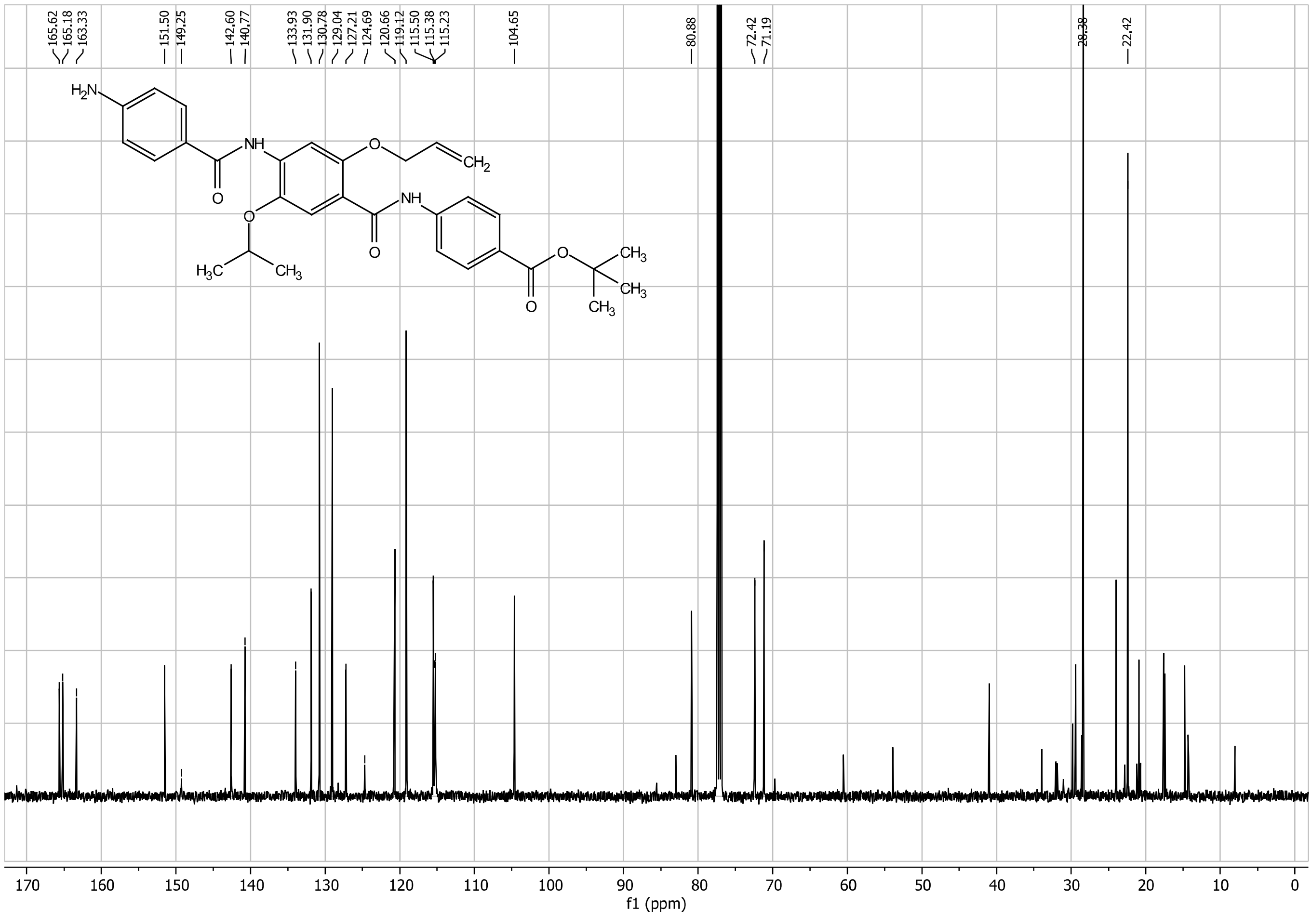
f1 (ppm)

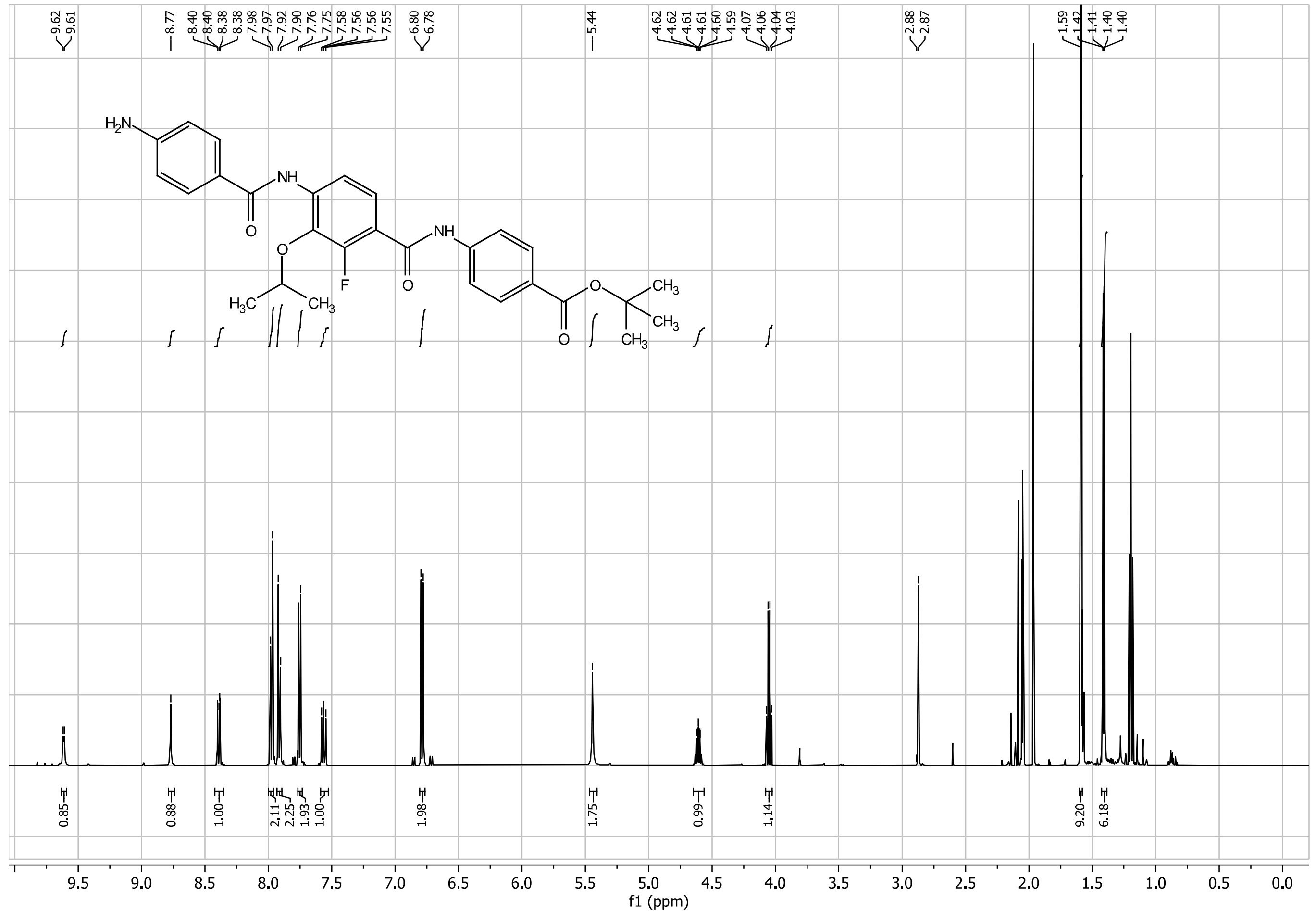

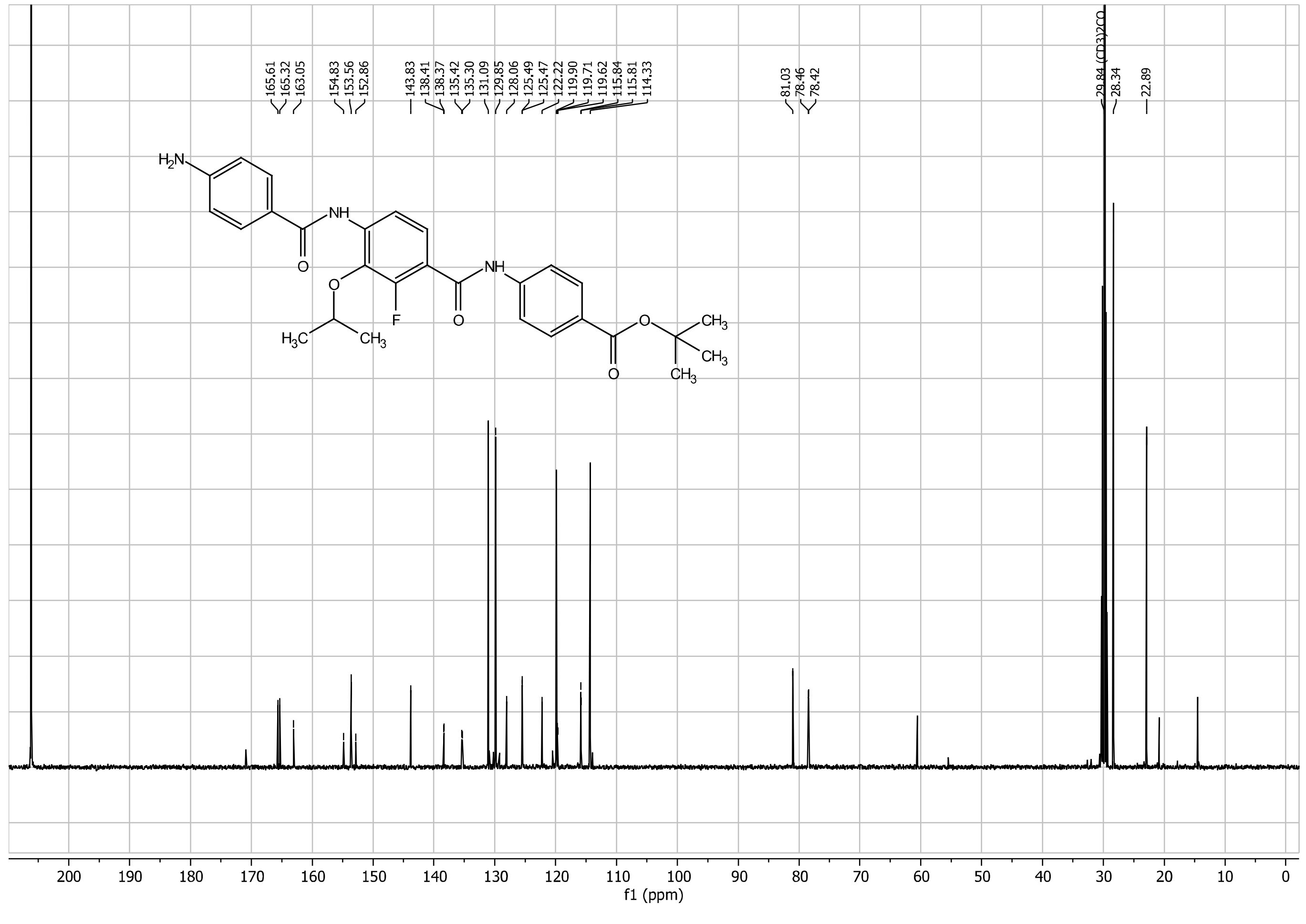

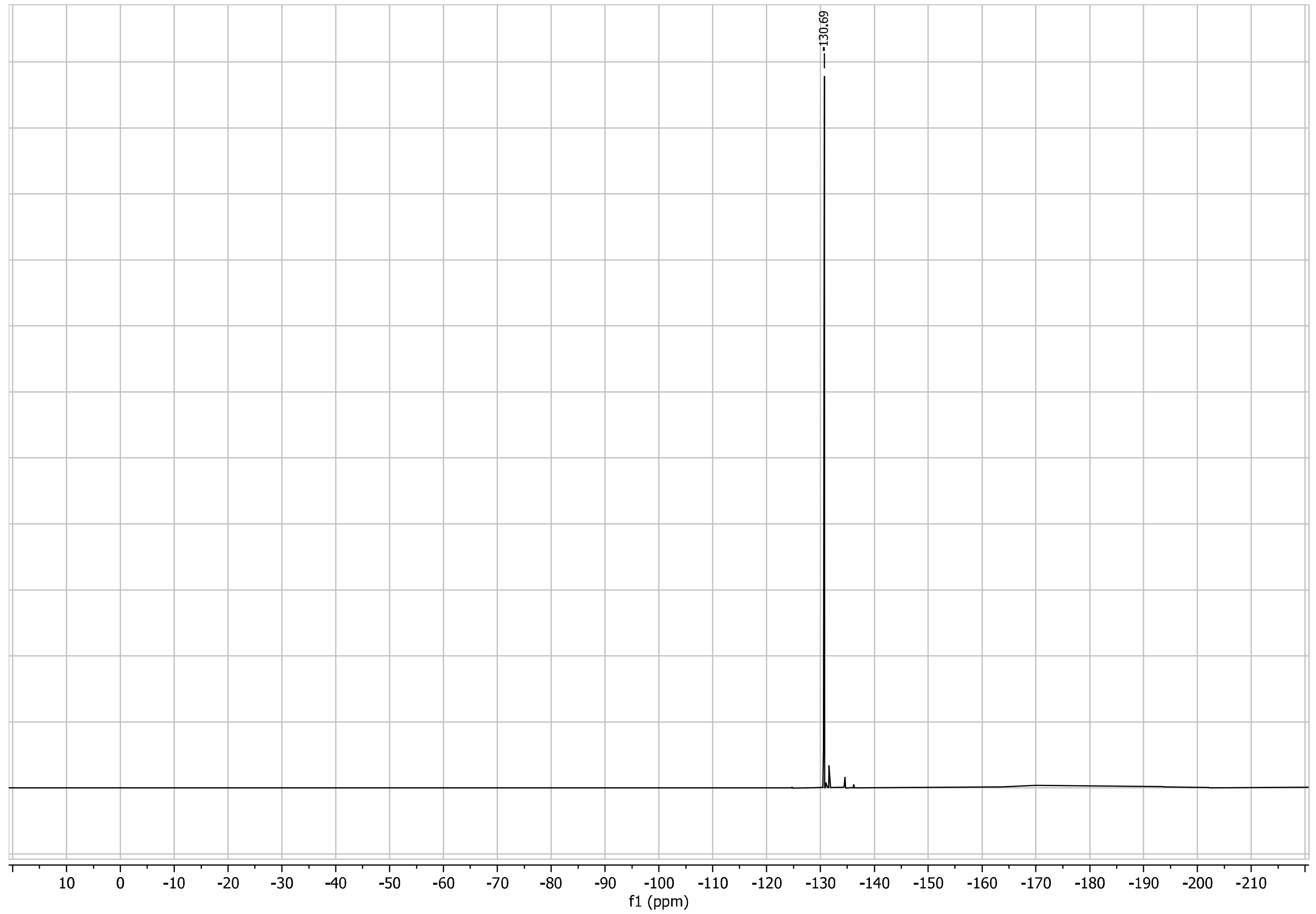
-130.69
f1 (ppm)

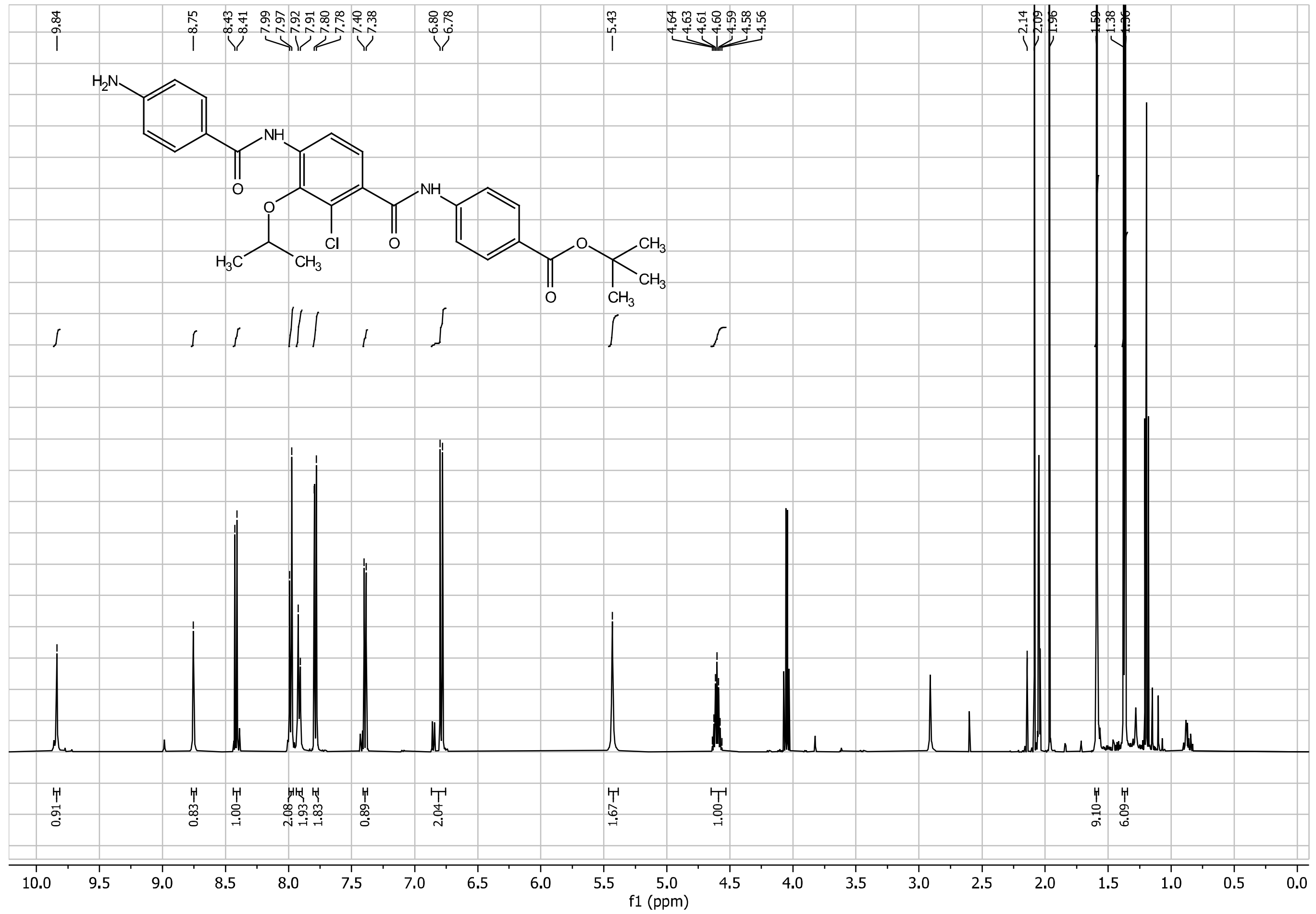
H2N
NH
O
NH
O
Cl
O
H3C
CH3
O
O
CH3
CH3
CH3
f1 (ppm)

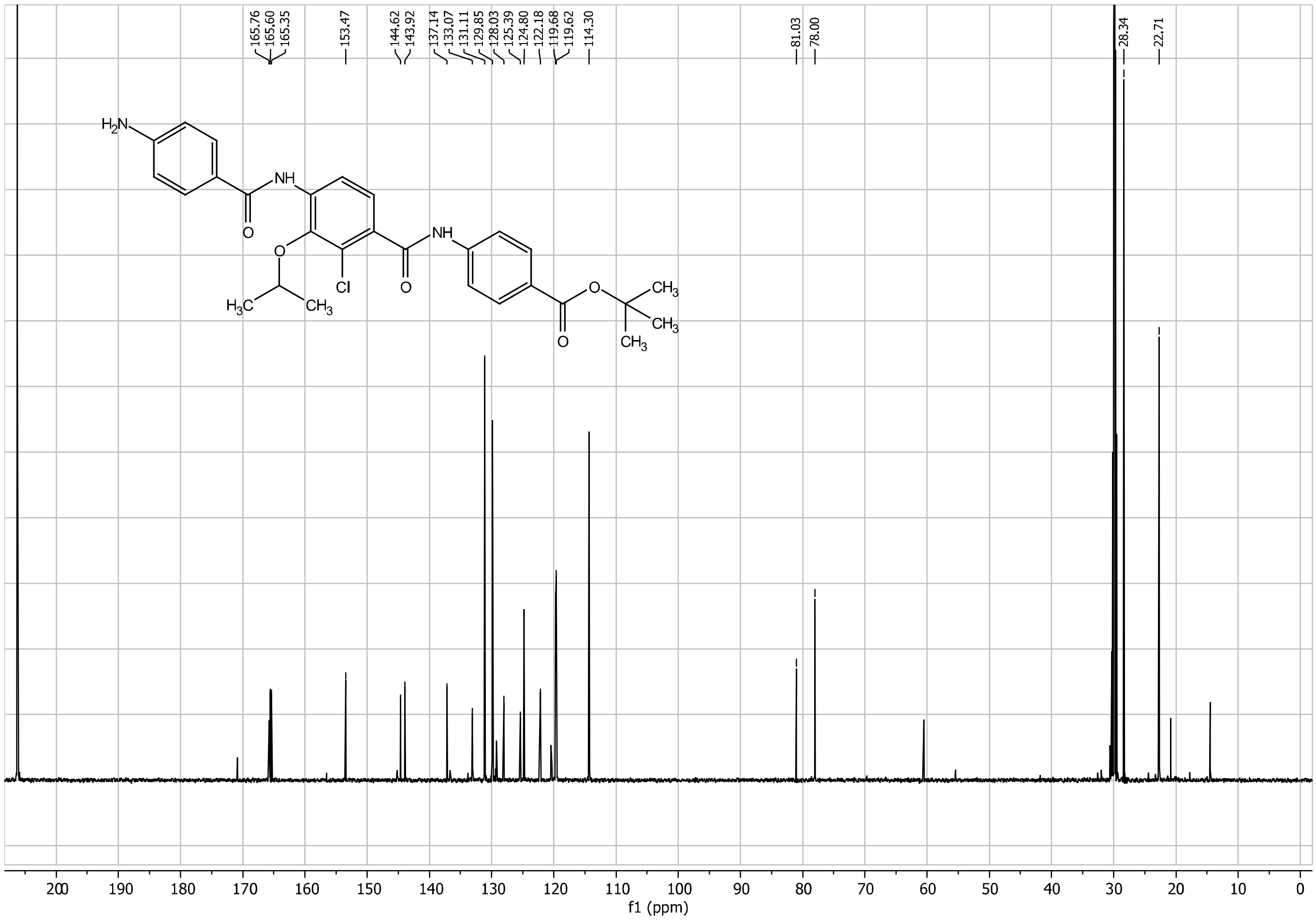

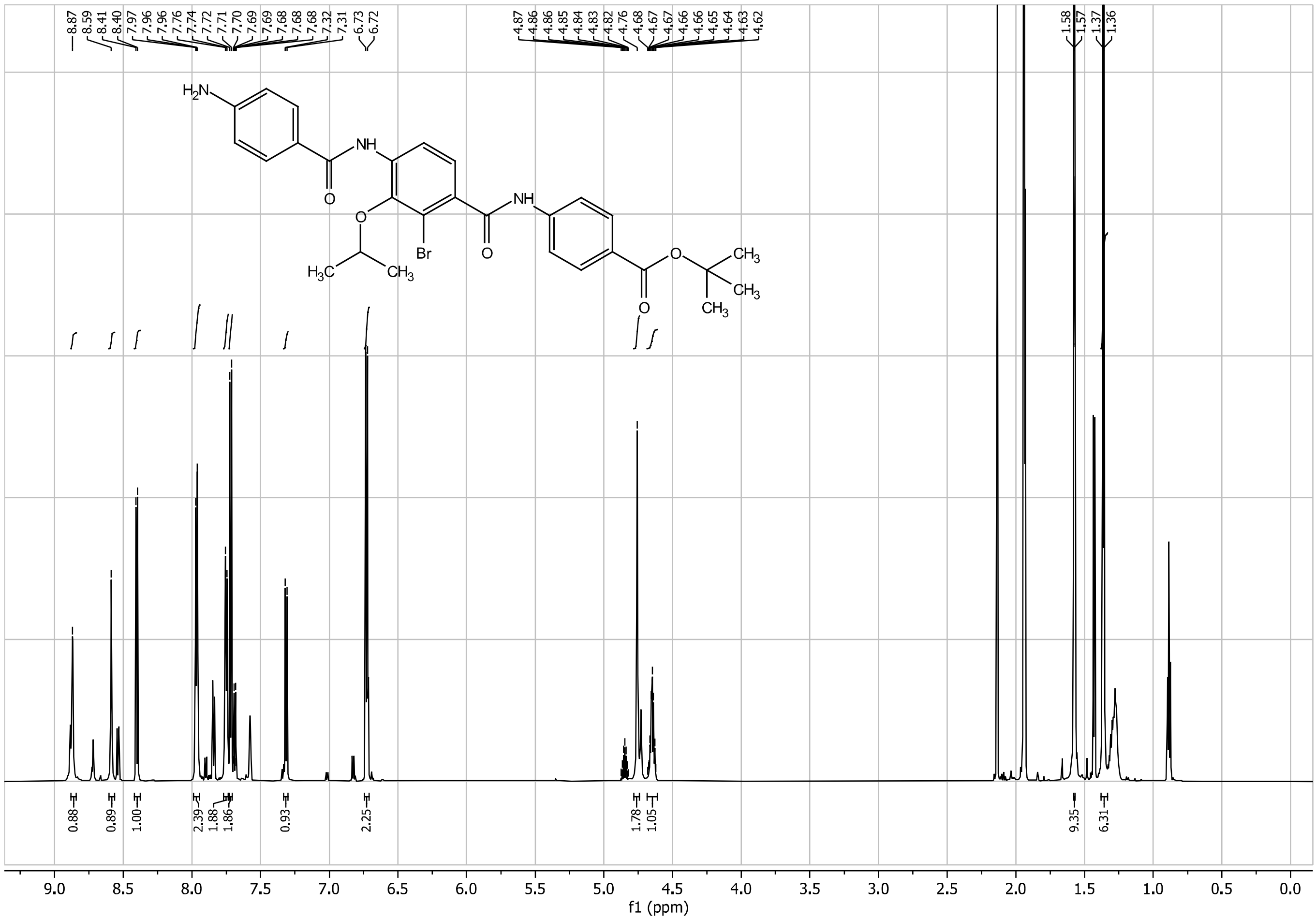

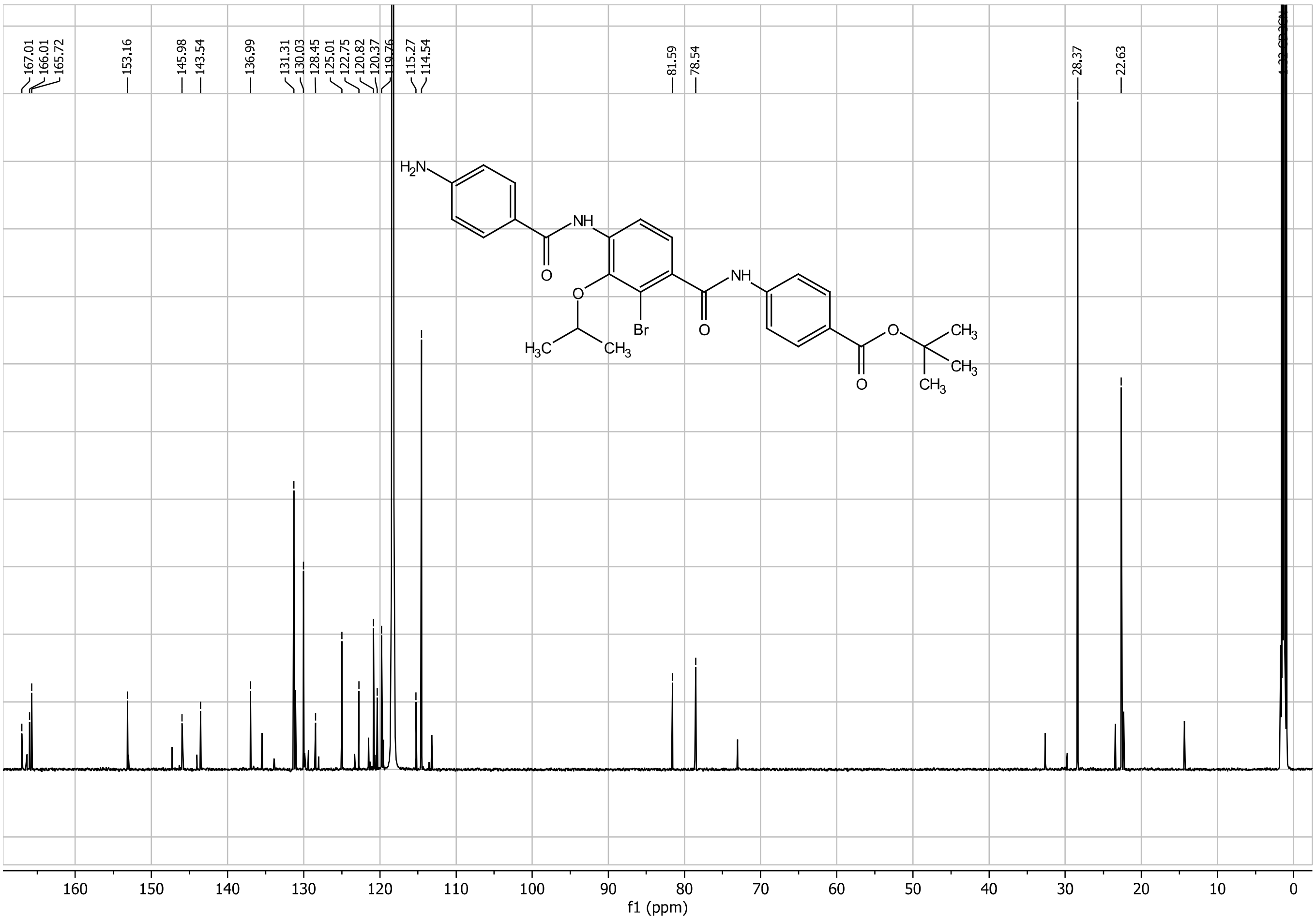
167.01
166.01
165.72
153.16
145.98
143.54
136.99
131.31
130.03
128.45
125.01
122.75
120.82
120.37
119.76
115.27
114.54
81.59
78.54
28.37
22.63
H2N
NH
O
O
NH
Br
O
H3C
CH3
O
CH3
CH3
O
CH3
f1 (ppm)

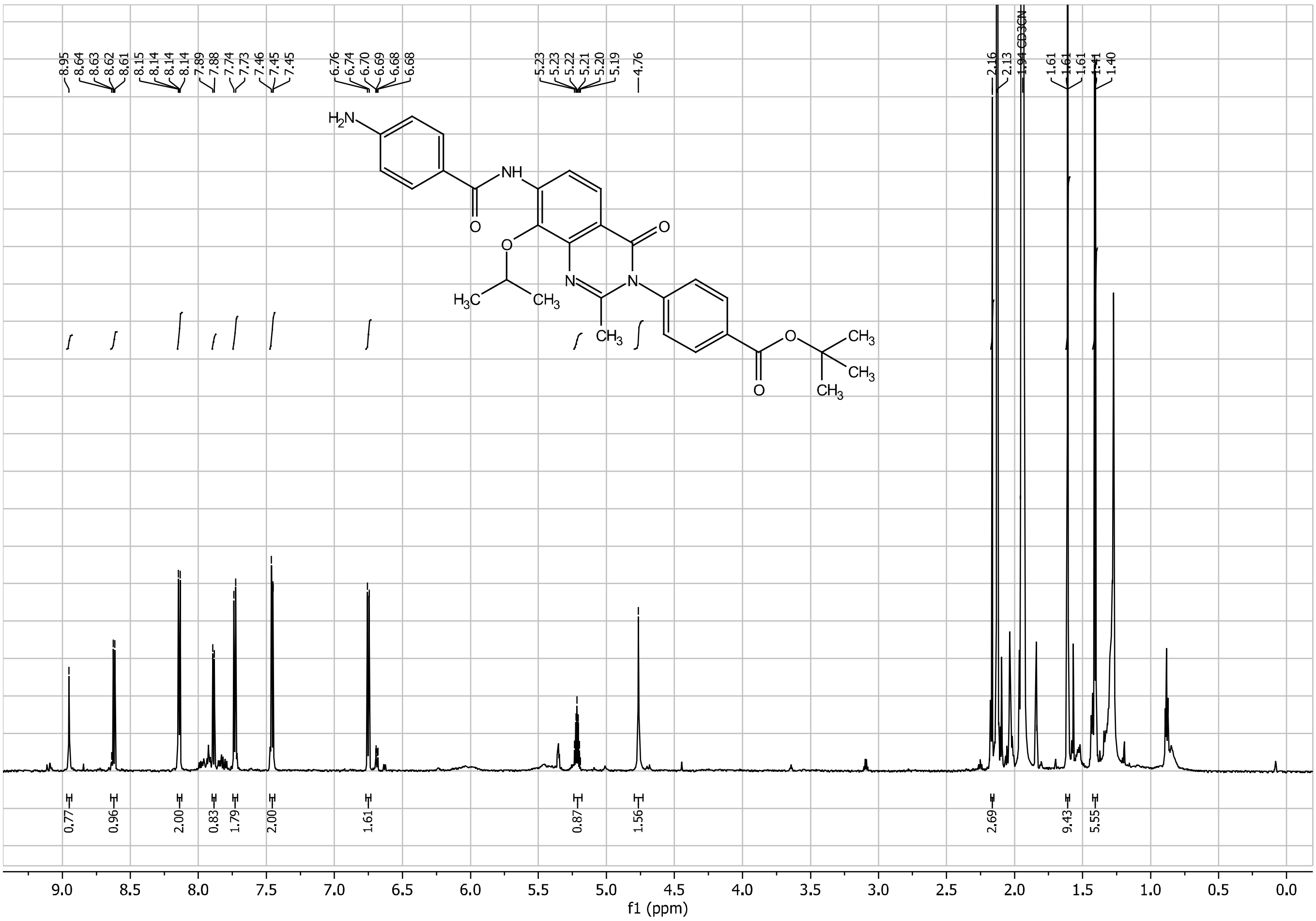
8.95
8.64
8.63
8.62
8.61
8.15
8.14
8.14
8.14
7.89
7.88
7.74
7.73
7.46
7.45
6.76
6.74
6.70
6.69
6.68
6.68
5.23
5.23
5.22
5.21
5.20
5.19
4.76
2.16
2.13
1.94 CD3CN
1.61
1.61
1.61
1.41
1.40
H2N
NH
O
O
O
N
N
H3C
CH3
CH3
O
CH3
CH3
O
CH3
0.77
0.96
2.00
0.83
1.79
2.00
1.61
0.87
1.56
2.69
9.43
5.55
f1 (ppm)

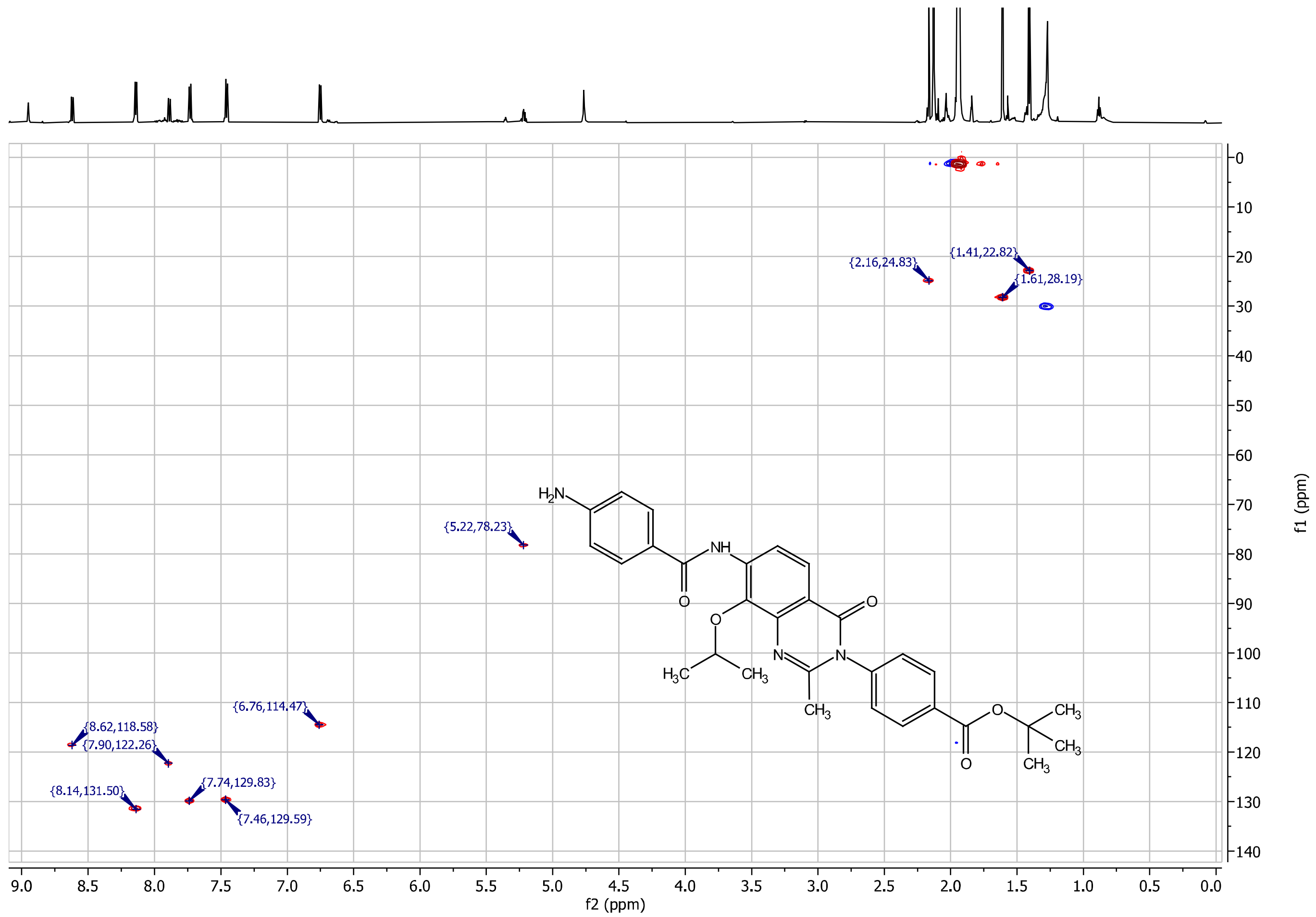
{2.16,24.83}
{1.41,22.82}
{1.61,28.19}
{5.22,78.23}
{6.76,114.47}
{8.62,118.58}
{7.90,122.26}
{7.74,129.83}
{8.14,131.50}
{7.46,129.59}
f2 (ppm)
f1 (ppm)

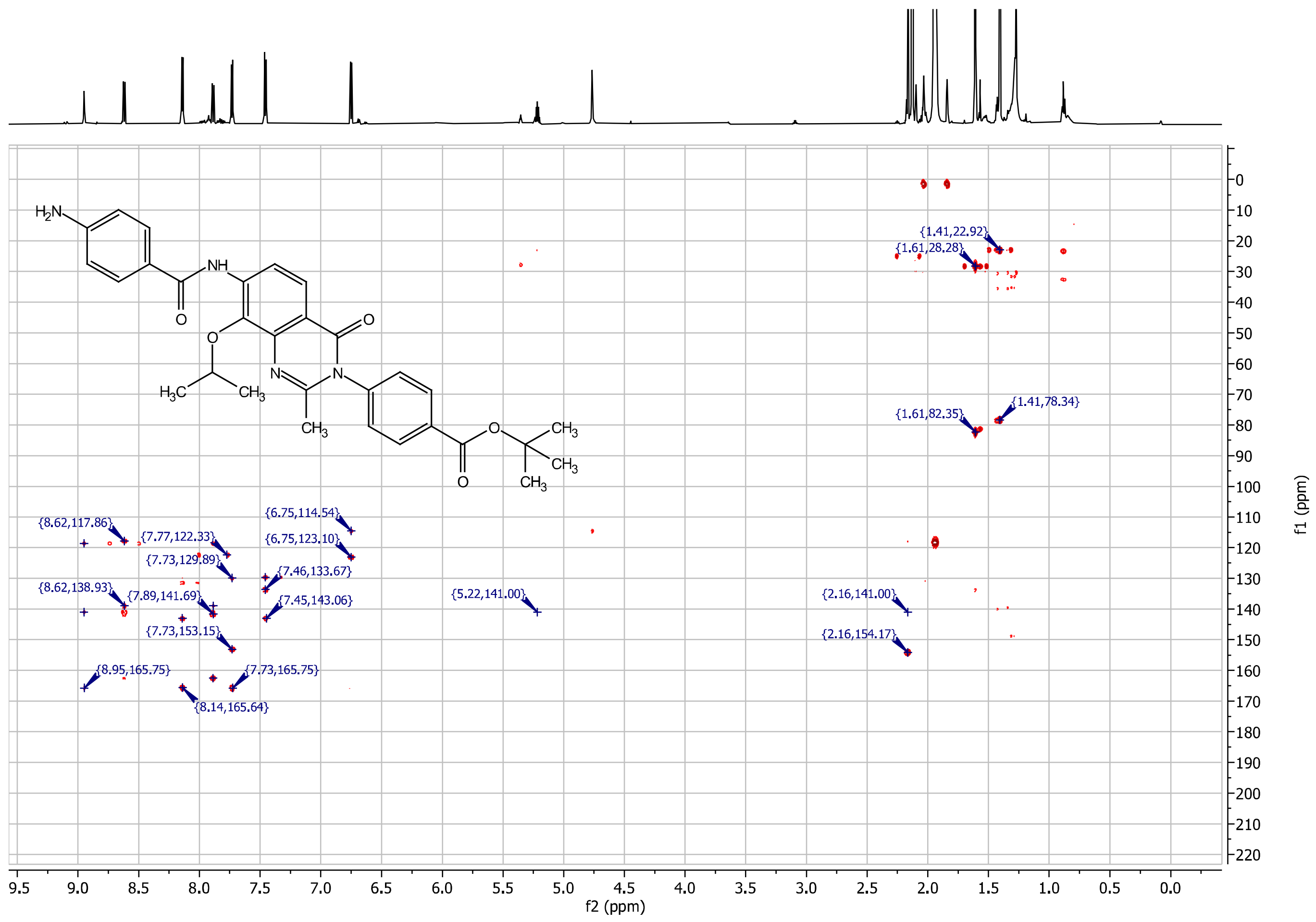
{1.41,22.92}
{1.61,28.28}
{1.41,78.34}
{1.61,82.35}
{6.75,114.54}
{8.62,117.86}
{7.77,122.33}
{6.75,123.10}
{7.73,129.89}
{7.46,133.67}
{8.62,138.93}
{7.89,141.69}
{7.45,143.06}
{5.22,141.00}
{2.16,141.00}
{7.73,153.15}
{2.16,154.17}
{8.95,165.75}
{7.73,165.75}
{8.14,165.64}
f2 (ppm)
f1 (ppm)

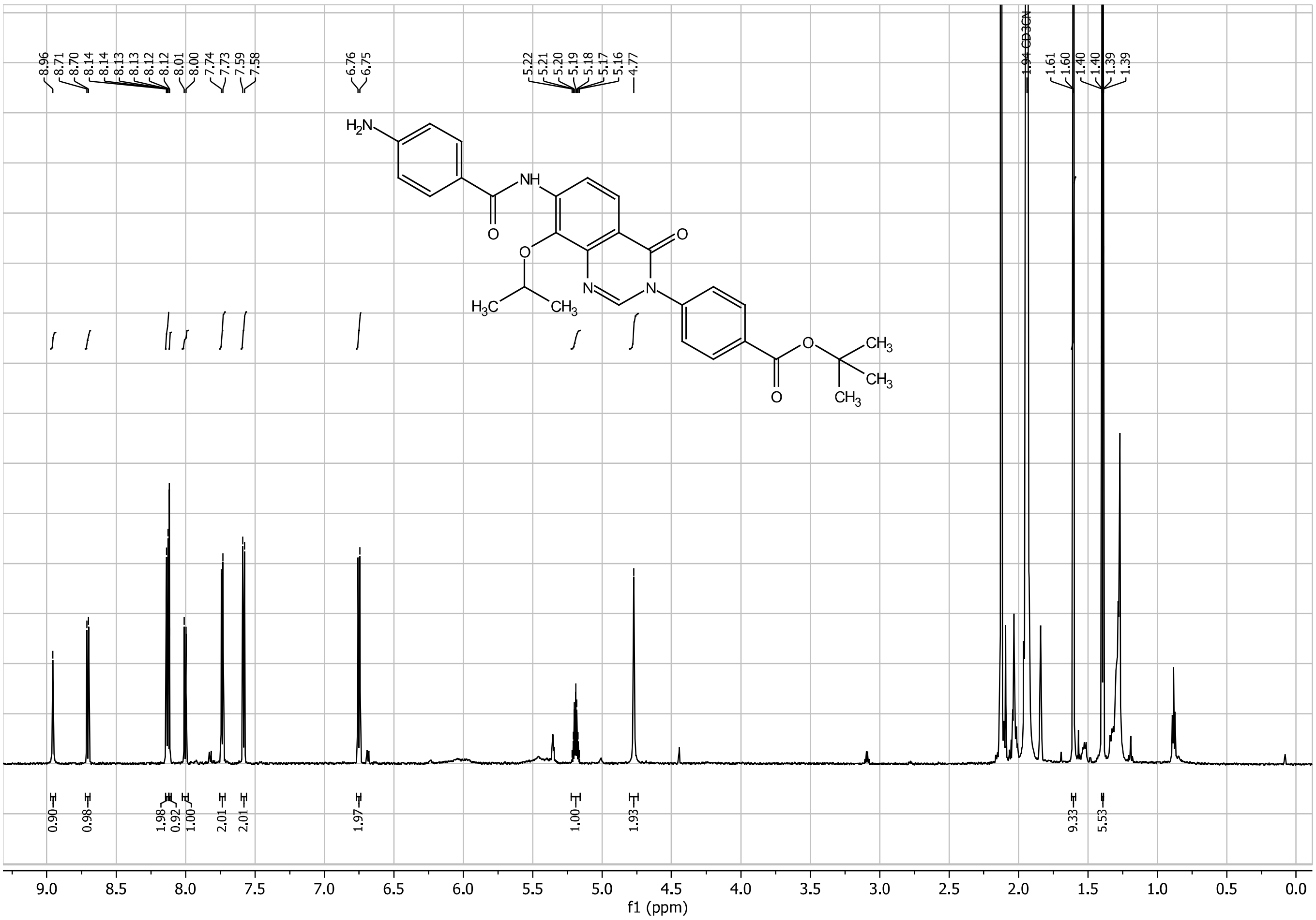

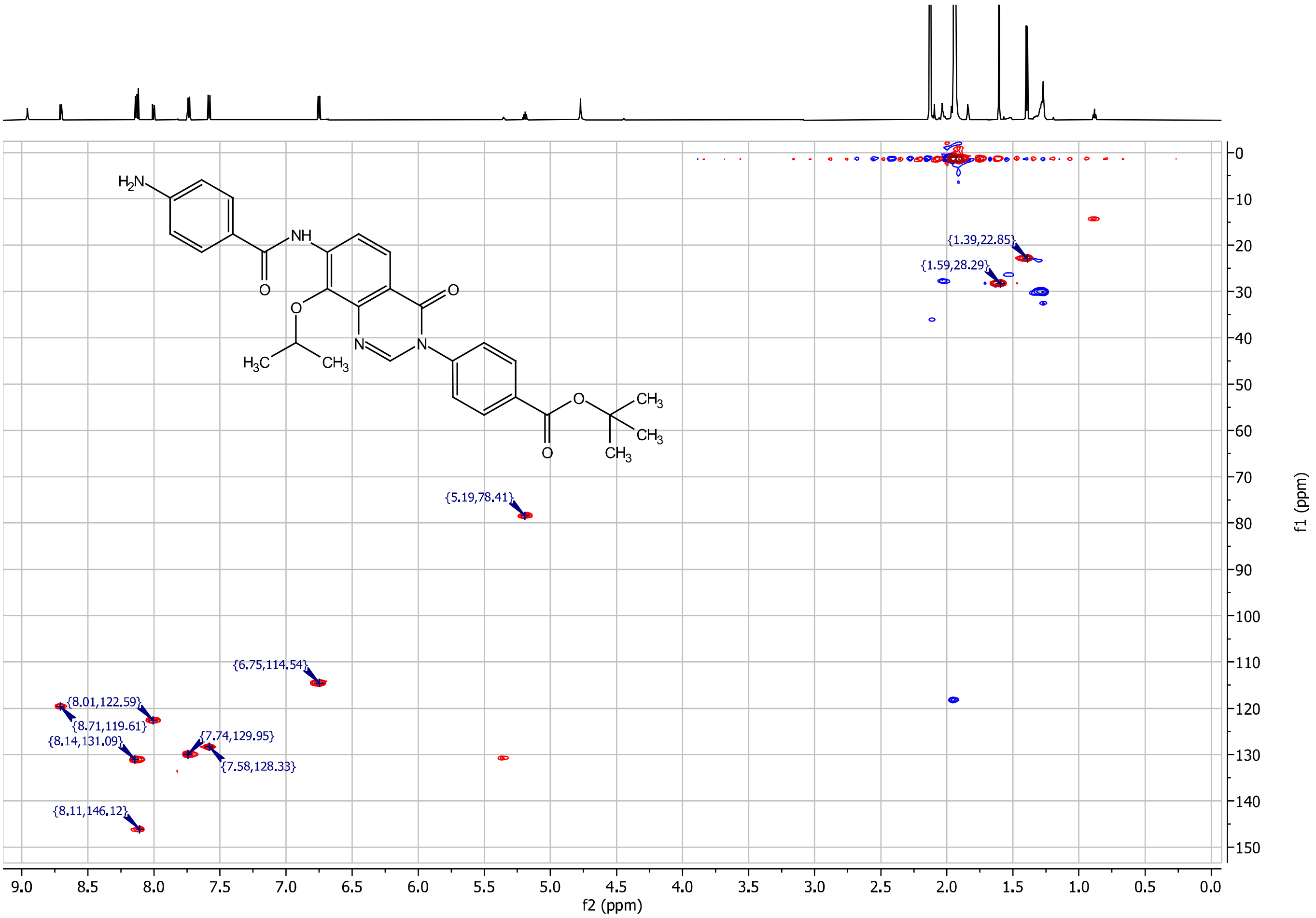

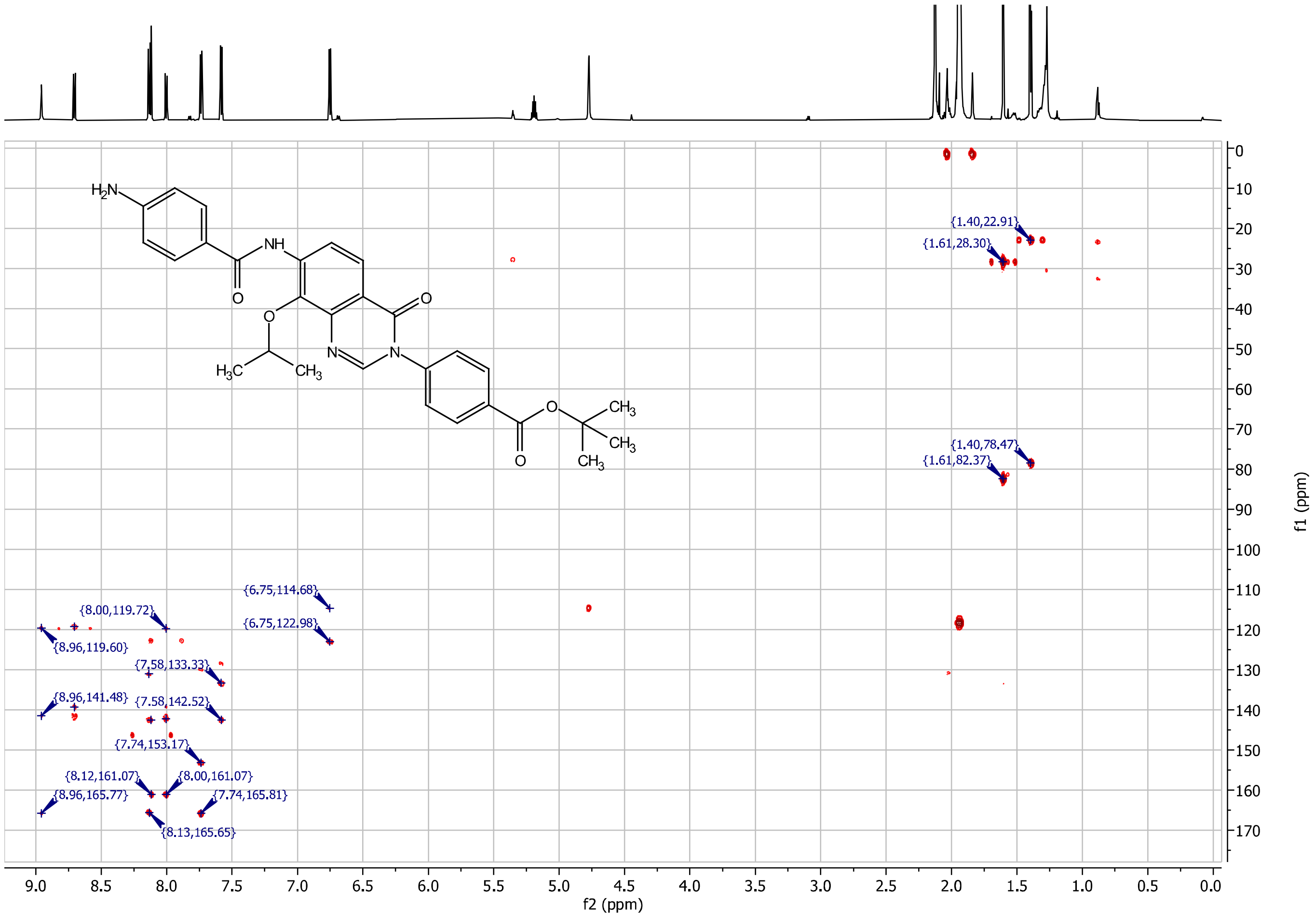
{1.40,22.91}
{1.61,28.30}
{1.40,78.47}
{1.61,82.37}
{6.75,114.68}
{6.75,122.98}
{8.00,119.72}
{8.96,119.60}
{7.58,133.33}
{8.96,141.48}
{7.58,142.52}
{7.74,153.17}
{8.12,161.07}
{8.00,161.07}
{8.96,165.77}
{7.74,165.81}
{8.13,165.65}
f2 (ppm)
f1 (ppm)

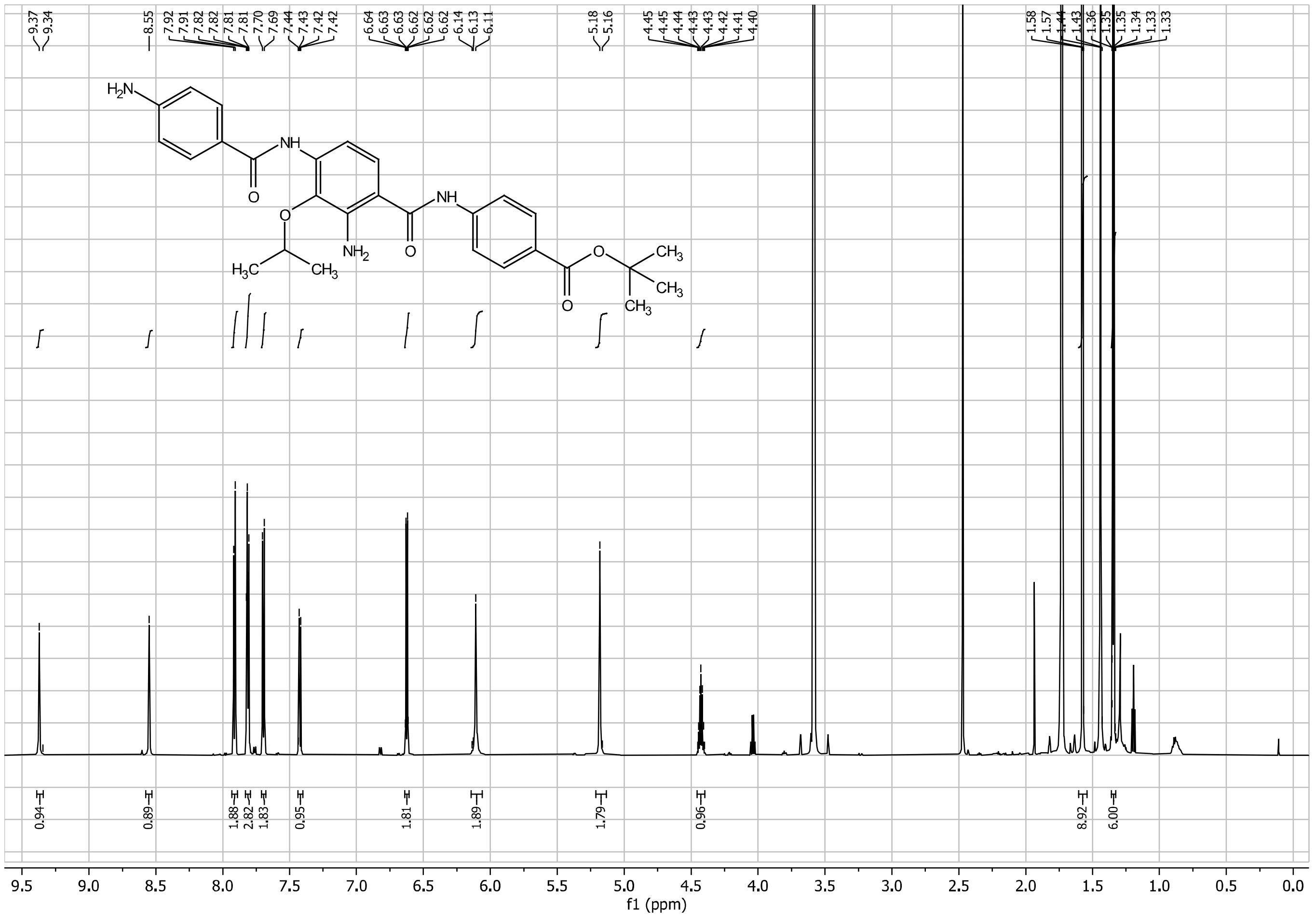
9.37
9.34
8.55
7.92
7.91
7.82
7.82
7.81
7.81
7.70
7.69
7.44
7.43
7.43
7.42
7.42
6.64
6.63
6.63
6.62
6.62
6.62
6.14
6.13
6.11
5.18
5.16
4.45
4.45
4.44
4.44
4.43
4.43
4.42
4.41
4.40
1.58
1.57
1.44
1.43
1.36
1.35
1.35
1.34
1.33
1.33
0.94
0.89
1.88
2.82
1.83
0.95
1.81
1.89
1.79
0.96
8.92
6.00
f1 (ppm)

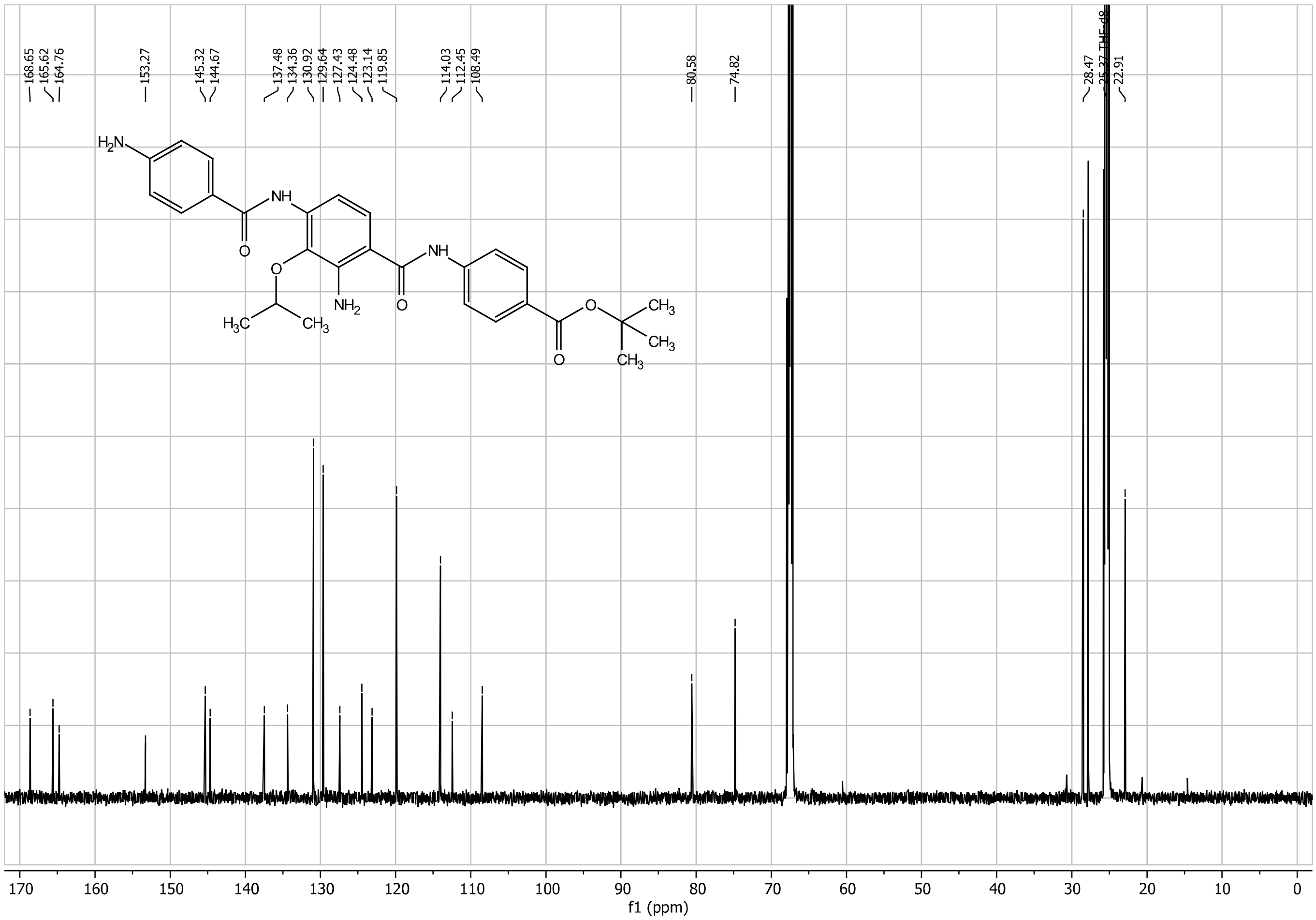

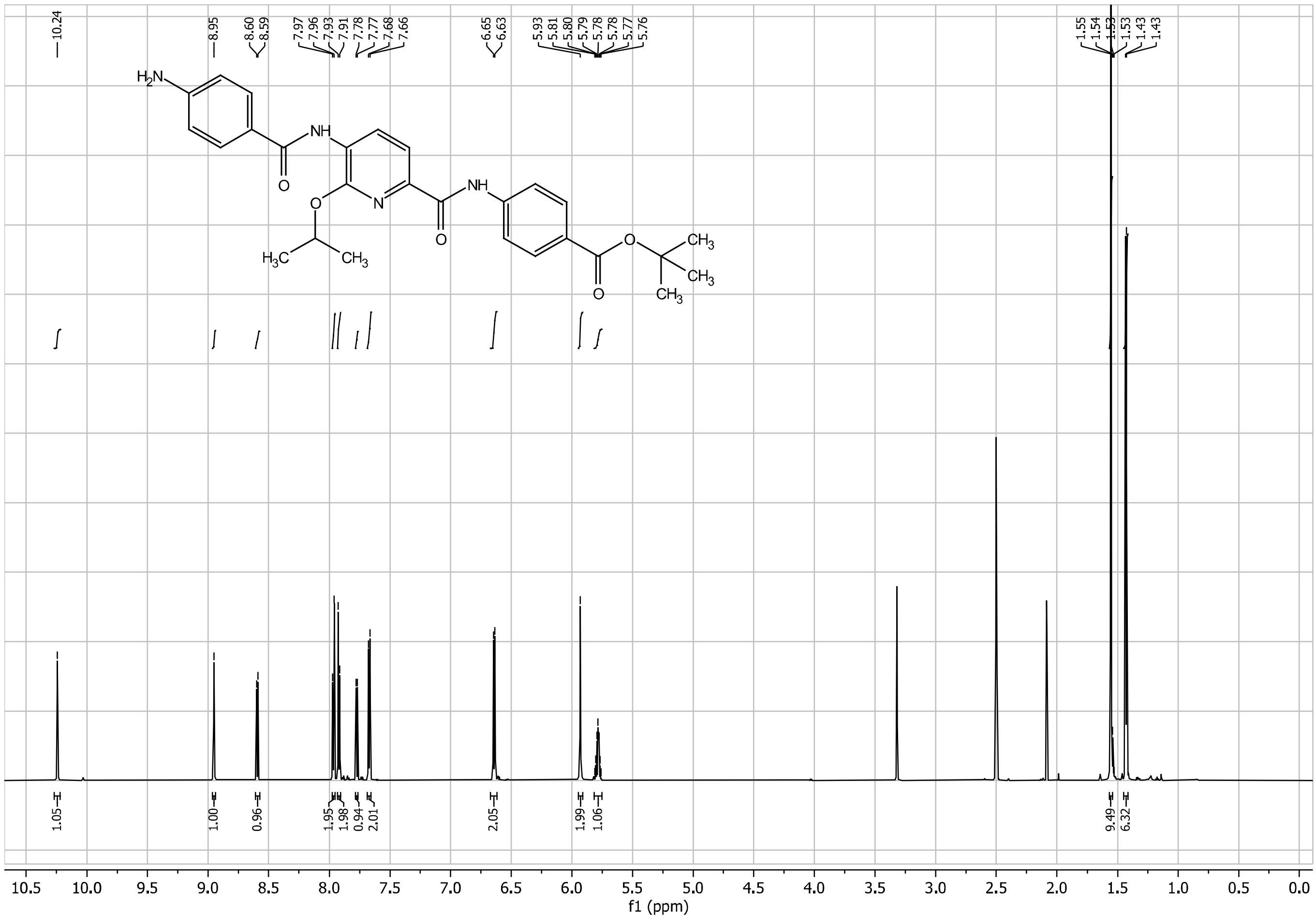

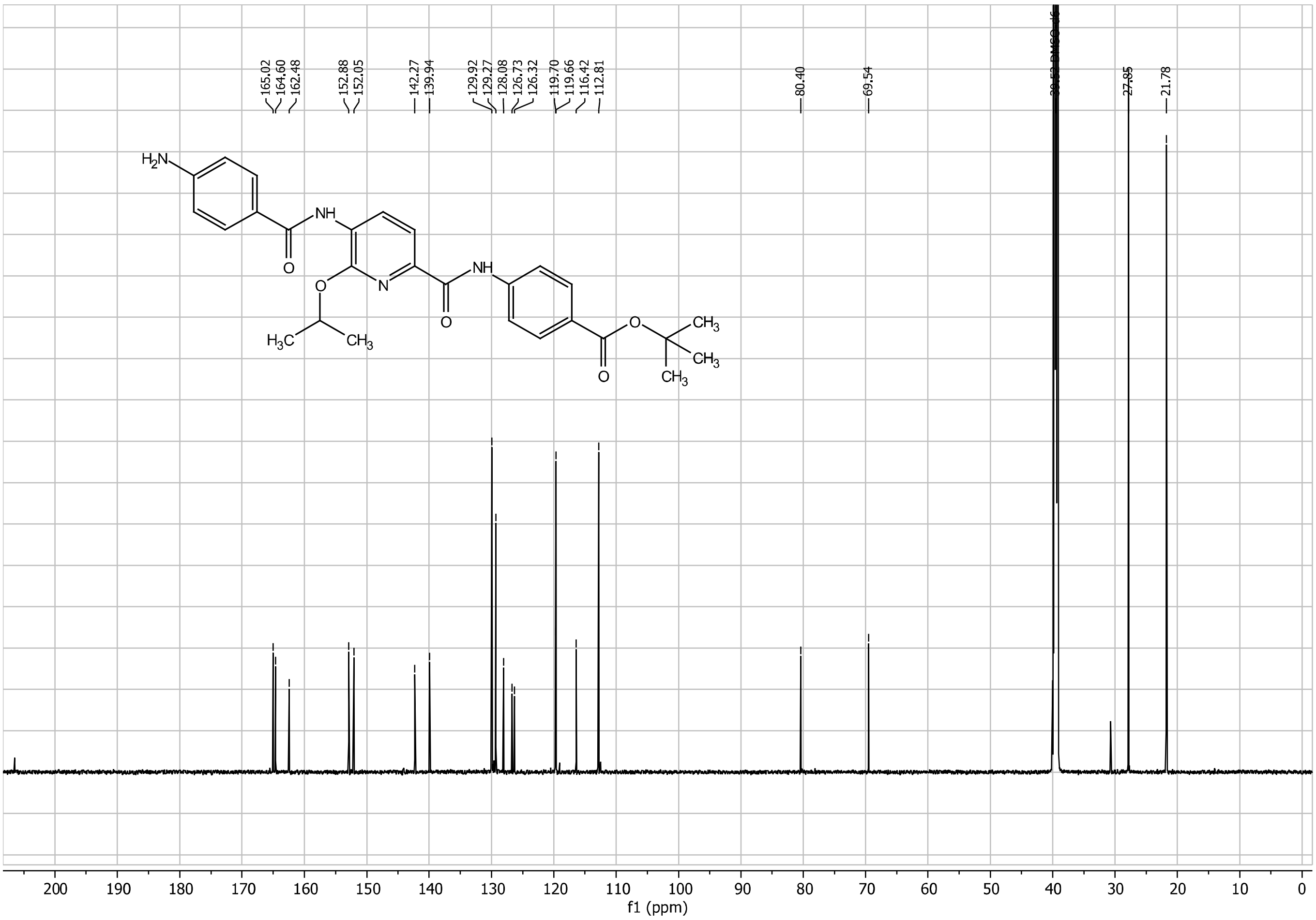
165.02
164.60
162.48
152.88
152.05
142.27
139.94
129.92
129.27
128.08
126.73
126.32
119.70
119.66
116.42
112.81
80.40
69.54
27.85
21.78
H2N
NH
O
O
N
NH
O
H3C
CH3
O
O
CH3
CH3
CH3
200 190 180 170 160 150 140 130 120 110 100 90 80 70 60 50 40 30 20 10 0
f1 (ppm)

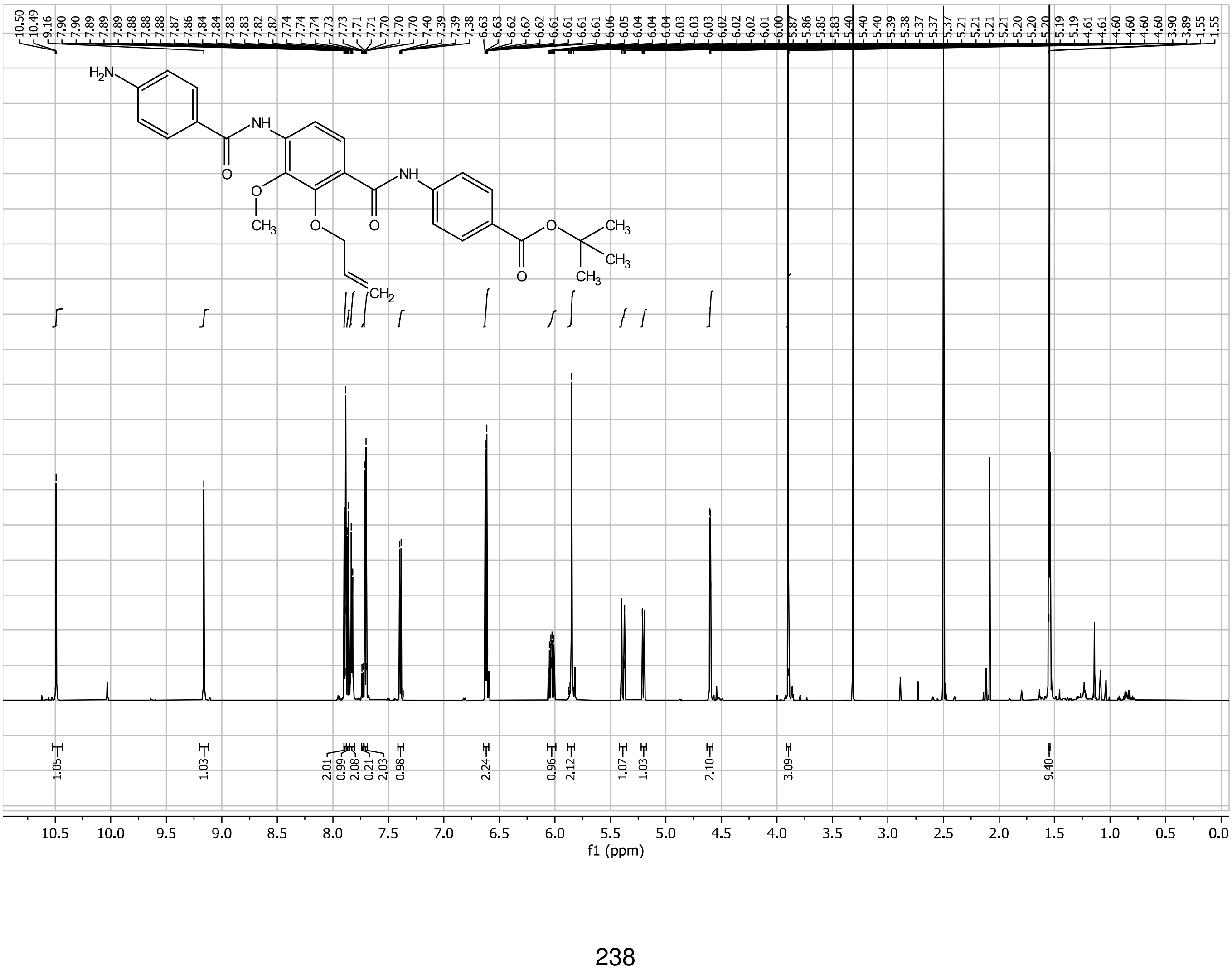
10.50
10.49
9.16
7.90
7.90
7.89
7.89
7.88
7.88
7.88
7.87
7.86
7.84
7.84
7.83
7.83
7.82
7.82
7.74
7.74
7.74
7.73
7.73
7.71
7.71
7.70
7.70
7.40
7.39
7.39
7.38
6.63
6.63
6.62
6.62
6.61
6.61
6.61
6.61
6.06
6.05
6.04
6.04
6.04
6.03
6.03
6.03
6.02
6.02
6.01
6.00
5.87
5.86
5.85
5.83
5.40
5.40
5.40
5.39
5.38
5.37
5.37
5.37
5.21
5.21
5.21
5.21
5.20
5.20
5.20
5.19
5.19
4.61
4.61
4.60
4.60
4.60
4.60
3.90
3.89
1.55
1.55
H2N
NH
O
O
CH3
O
CH2
O
NH
O
O
CH3
CH3
CH3
1.05
1.03
2.01
0.99
2.08
0.21
2.03
0.98
2.24
0.96
2.12
1.07
1.03
2.10
3.09
9.40
10.5 10.0 9.5 9.0 8.5 8.0 7.5 7.0 6.5 6.0 5.5 5.0 4.5 4.0 3.5 3.0 2.5 2.0 1.5 1.0 0.5 0.0
f1 (ppm)

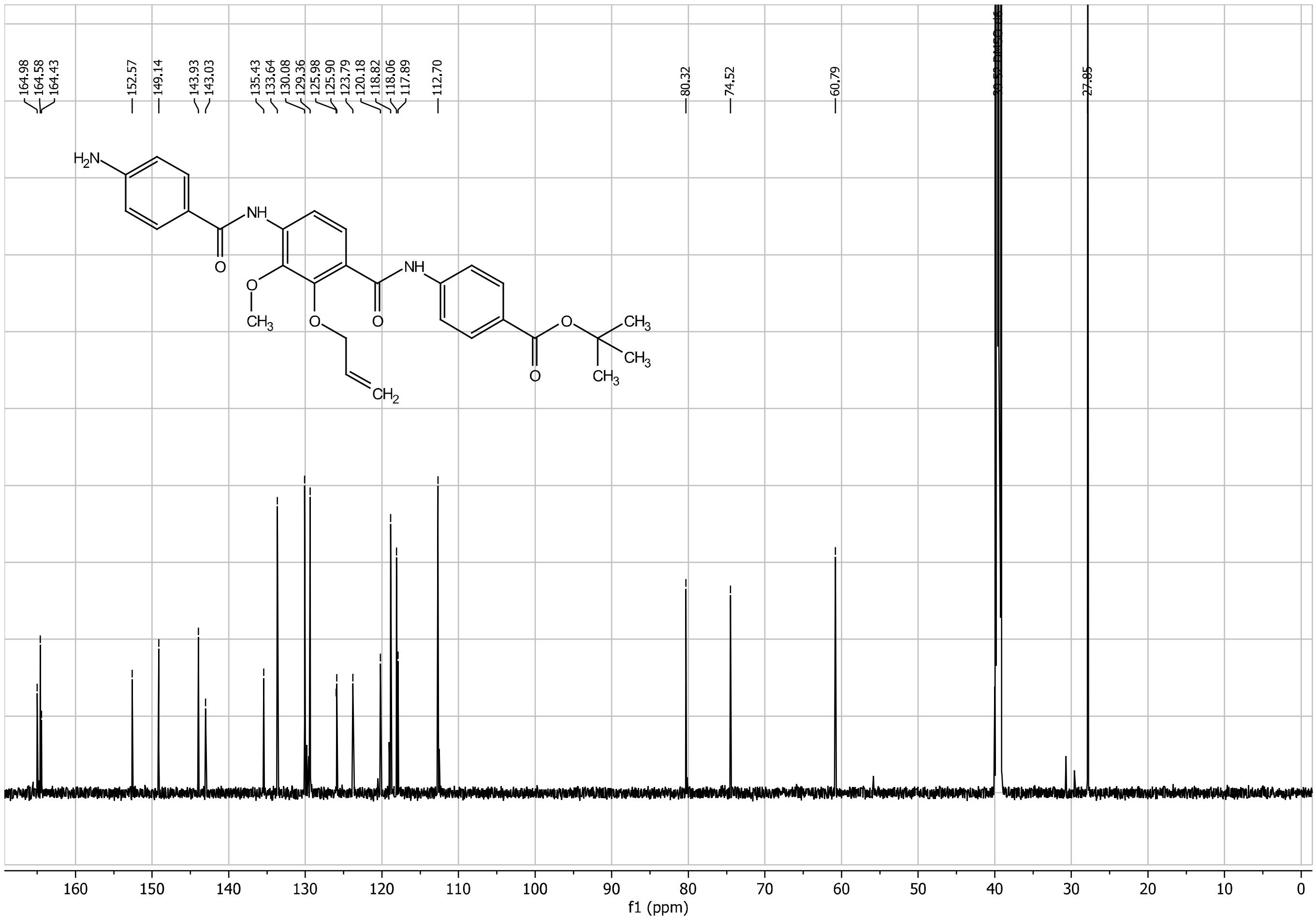
164.98
164.58
164.43
152.57
149.14
143.93
143.03
135.43
133.64
130.08
129.36
125.98
125.90
123.79
120.18
118.82
118.06
117.89
112.70
80.32
74.52
60.79
27.85
f1 (ppm)

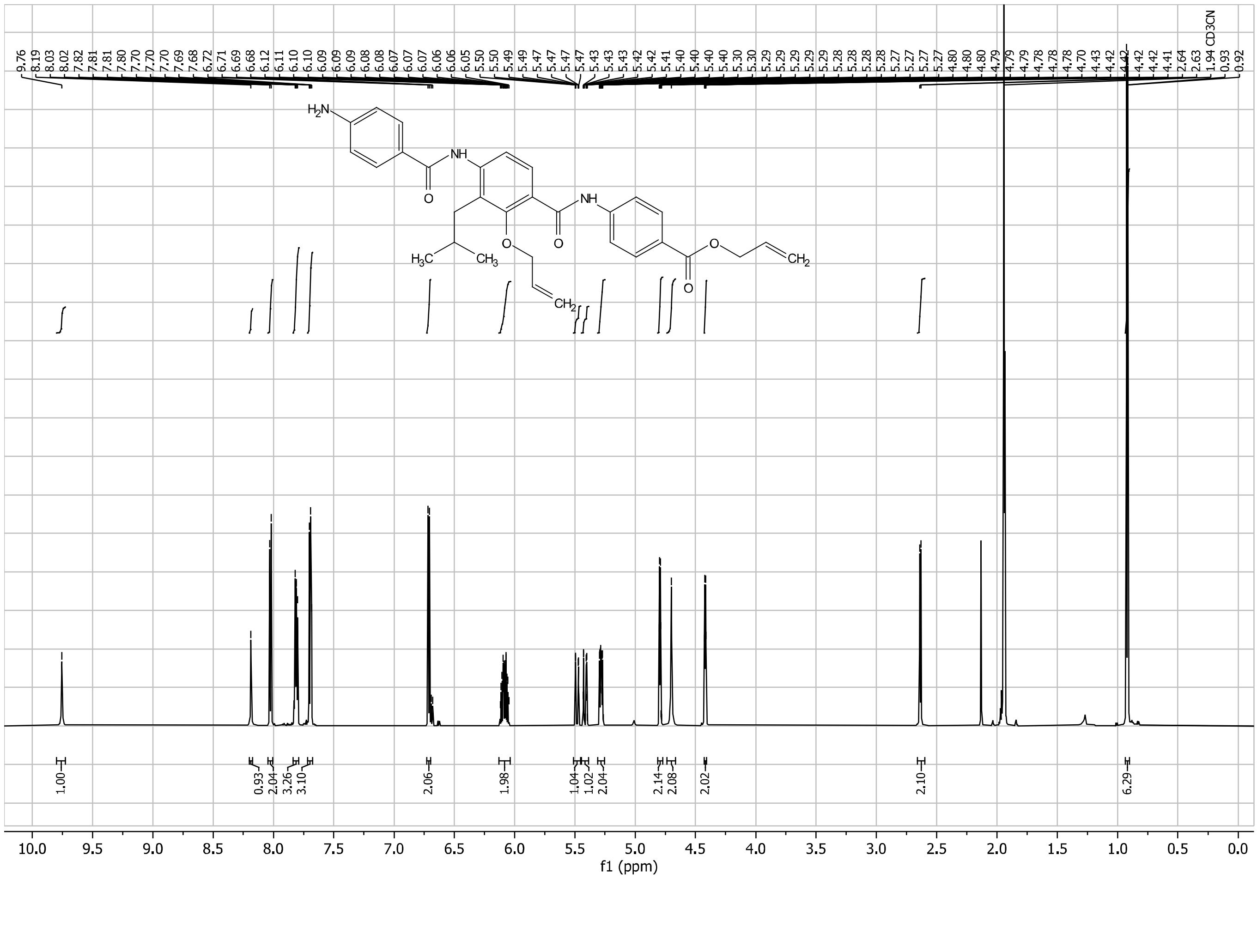
9.76
8.19
8.03
8.02
7.82
7.81
7.80
7.70
7.70
7.69
7.68
6.72
6.71
6.69
6.68
6.12
6.11
6.10
6.10
6.09
6.09
6.08
6.08
6.07
6.07
6.06
6.05
5.50
5.50
5.49
5.47
5.47
5.47
5.43
5.43
5.42
5.41
5.40
5.40
5.40
5.30
5.29
5.29
5.29
5.29
5.28
5.28
5.28
5.27
5.27
5.27
4.80
4.80
4.80
4.79
4.79
4.78
4.78
4.70
4.43
4.42
4.42
4.42
4.41
2.64
2.63
1.94 CD3CN
0.93
0.92
1.00
0.93
2.04
3.26
3.10
2.06
1.98
1.04
1.02
2.04
2.14
2.08
2.02
2.10
6.29
f1 (ppm)

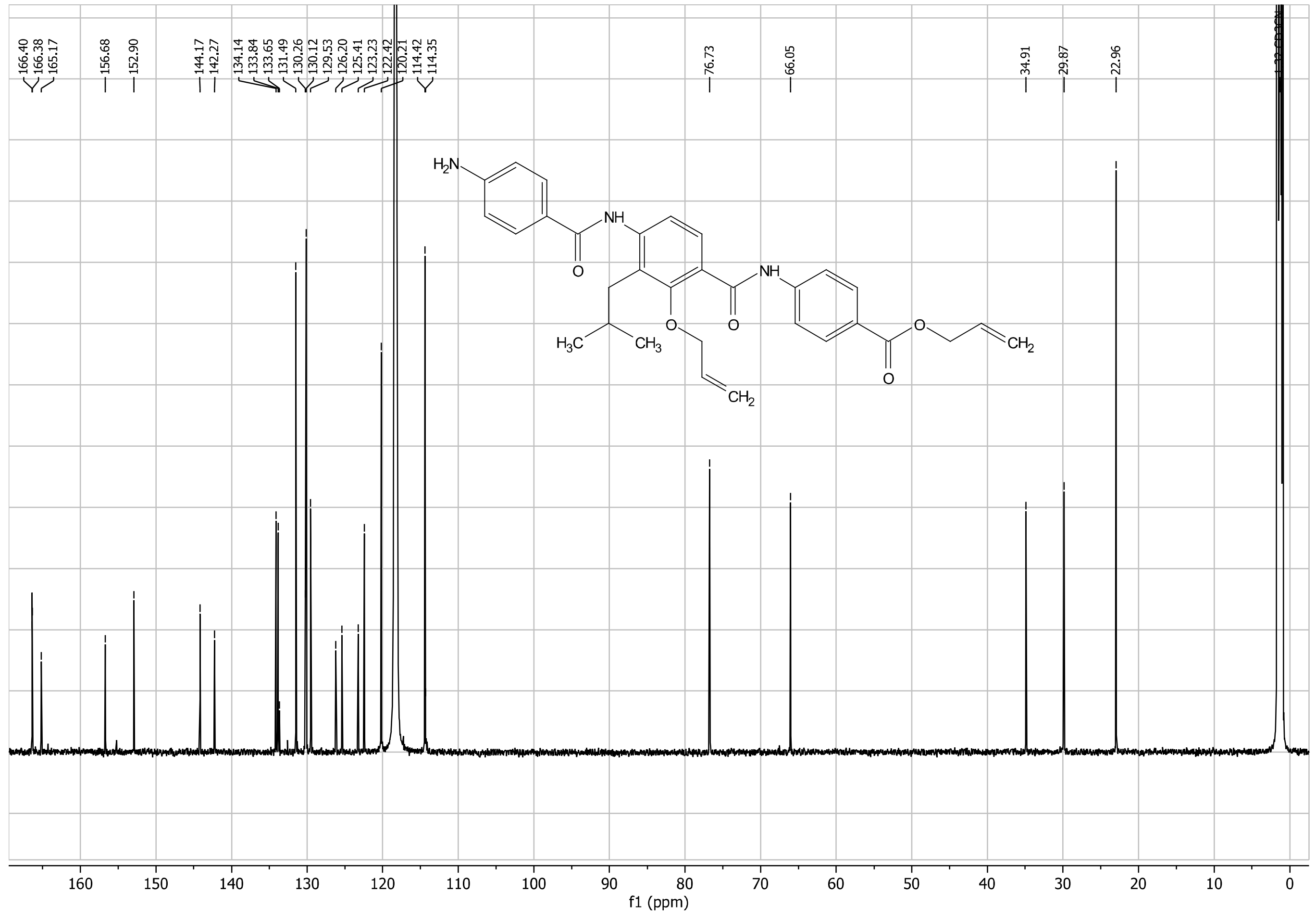

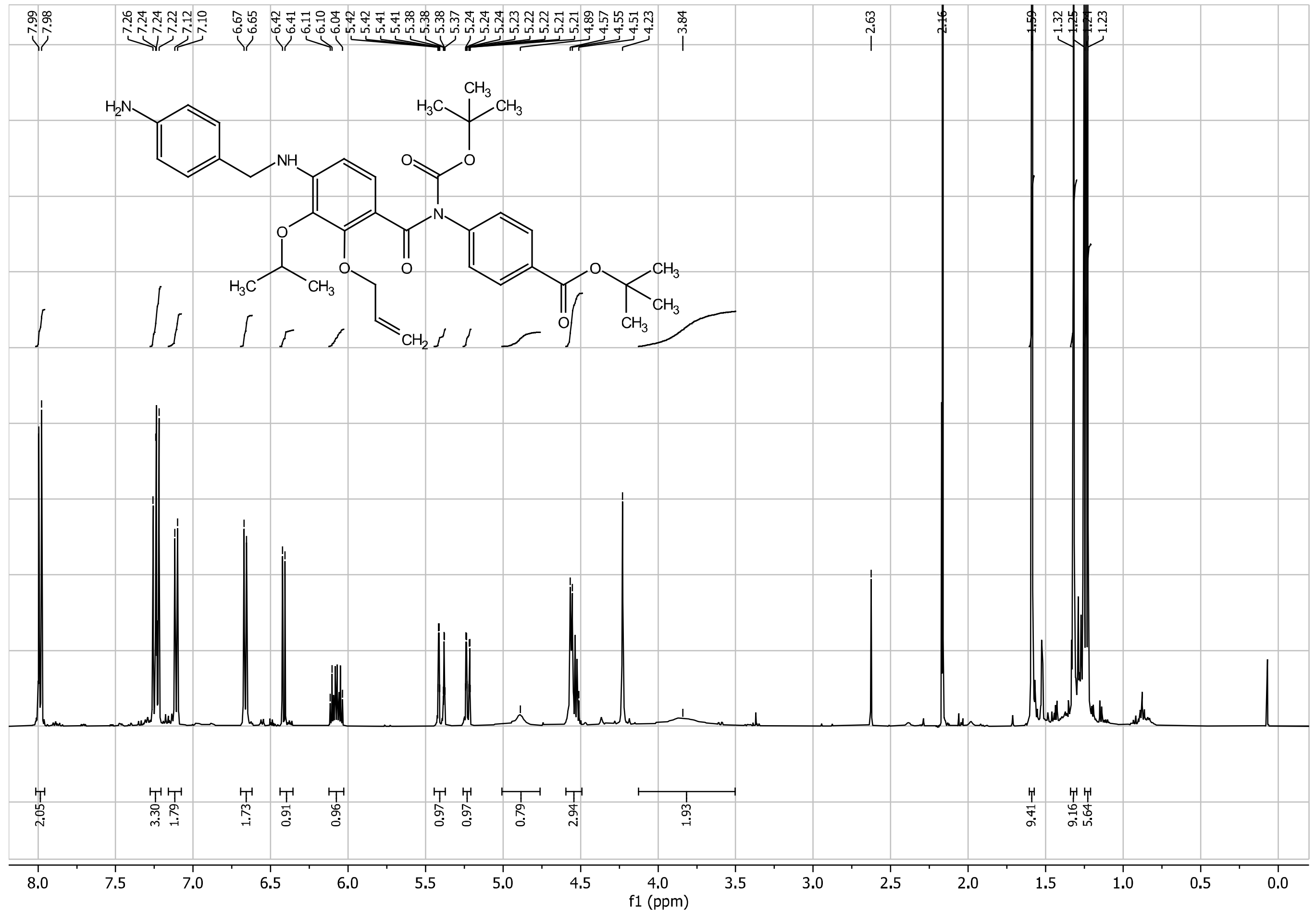

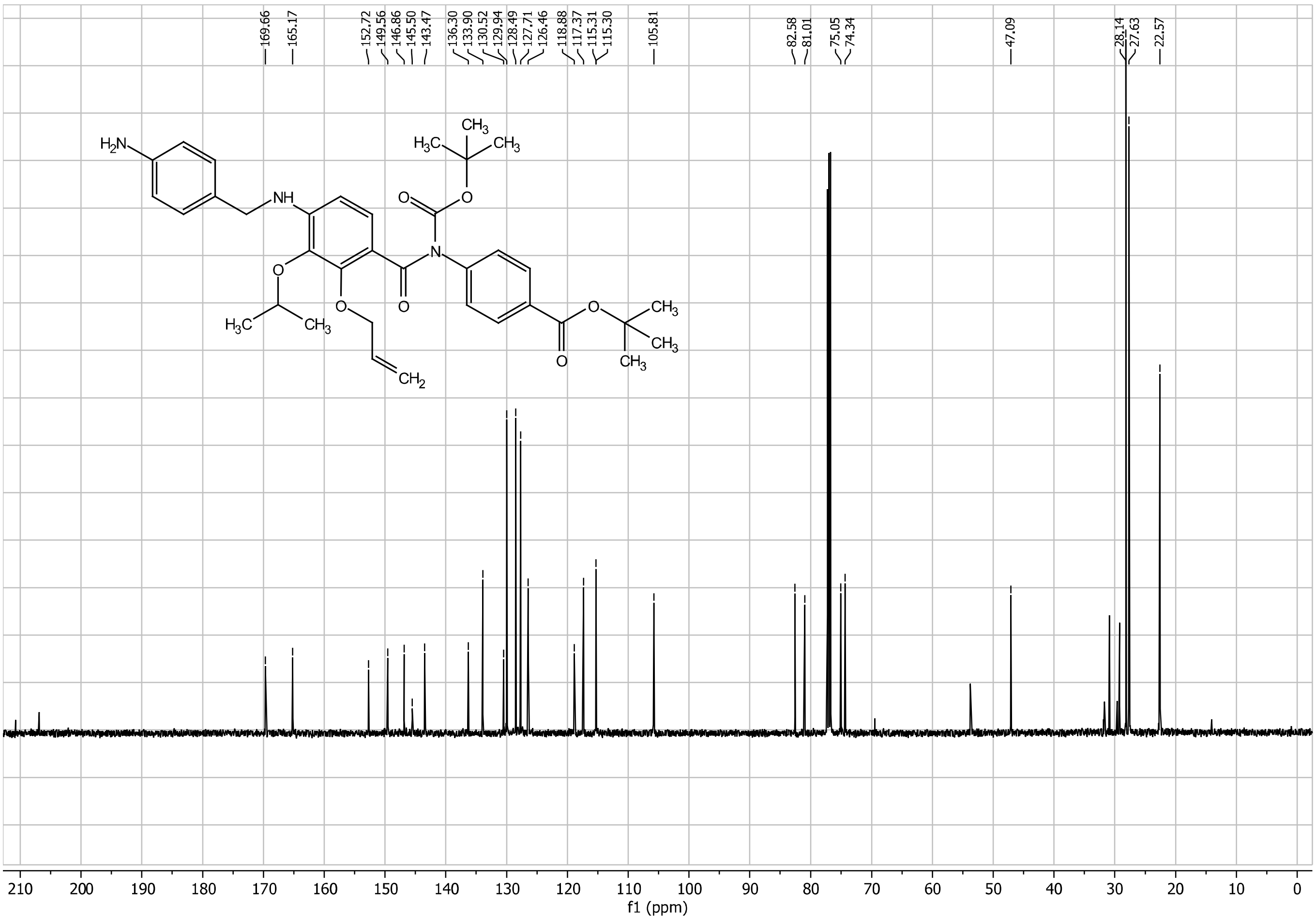

169.66
165.17
152.72
149.56
146.86
145.50
143.47
136.30
133.90
130.52
129.94
128.49
127.71
126.46
118.88
117.37
115.31
115.30
105.81
82.58
81.01
75.05
74.34
47.09
28.14
27.63
22.57
f1 (ppm)

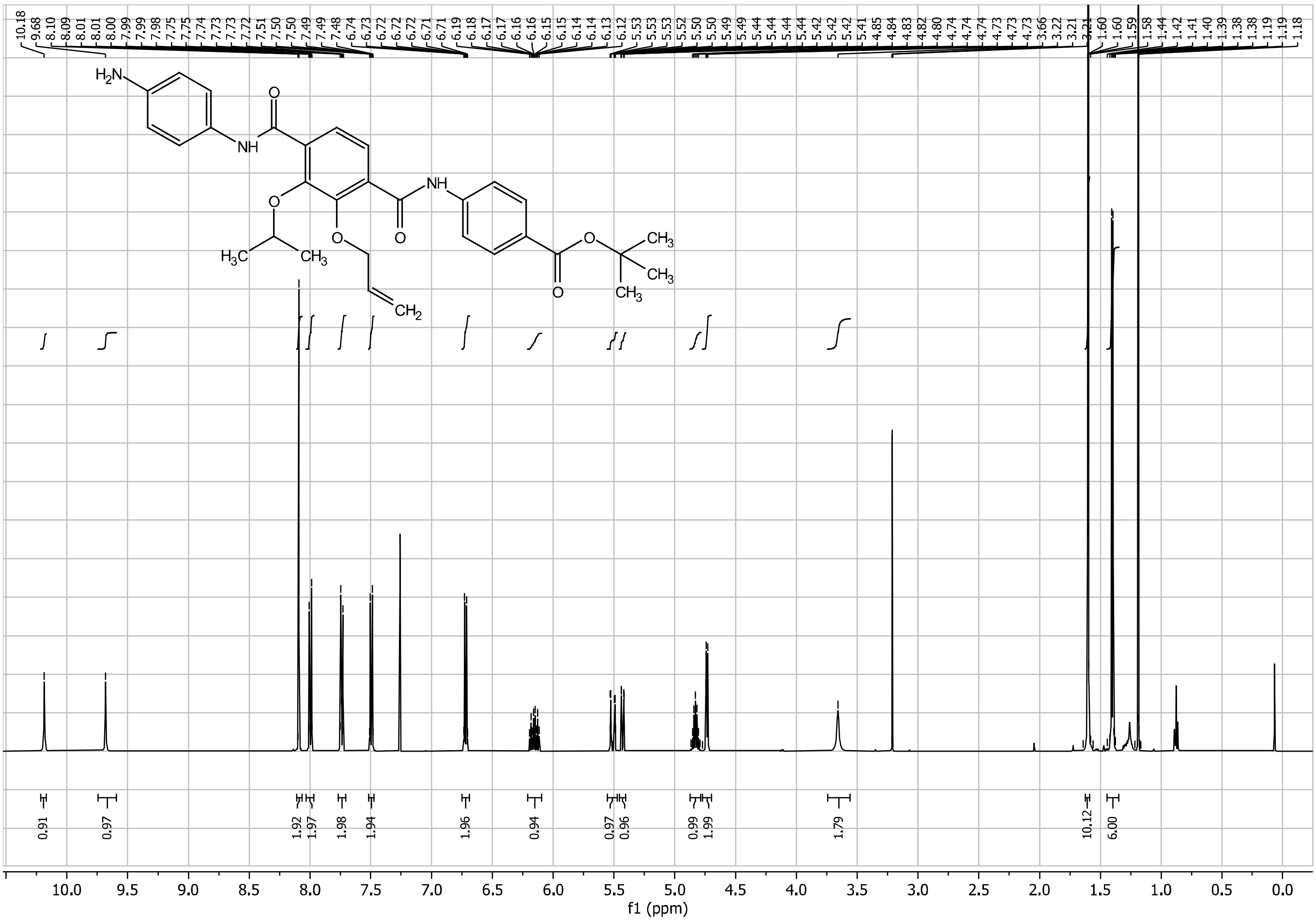

10.18
9.68
8.10
8.09
8.01
8.01
8.00
7.99
7.99
7.98
7.75
7.75
7.74
7.73
7.73
7.72
7.51
7.50
7.49
7.49
7.48
6.74
6.73
6.72
6.72
6.72
6.71
6.71
6.19
6.18
6.17
6.17
6.16
6.16
6.15
6.15
6.14
6.14
6.13
6.12
5.53
5.53
5.53
5.52
5.50
5.50
5.49
5.44
5.44
5.44
5.42
5.42
5.41
4.85
4.84
4.83
4.82
4.80
4.74
4.74
4.74
4.73
4.73
3.66
3.22
3.21
3.21
1.60
1.60
1.59
1.58
1.44
1.42
1.41
1.40
1.39
1.38
1.38
1.19
1.19
1.18
0.91
0.97
1.92
1.97
1.98
1.94
1.96
0.94
0.97
0.96
0.99
1.99
1.79
10.12
6.00
f1 (ppm)

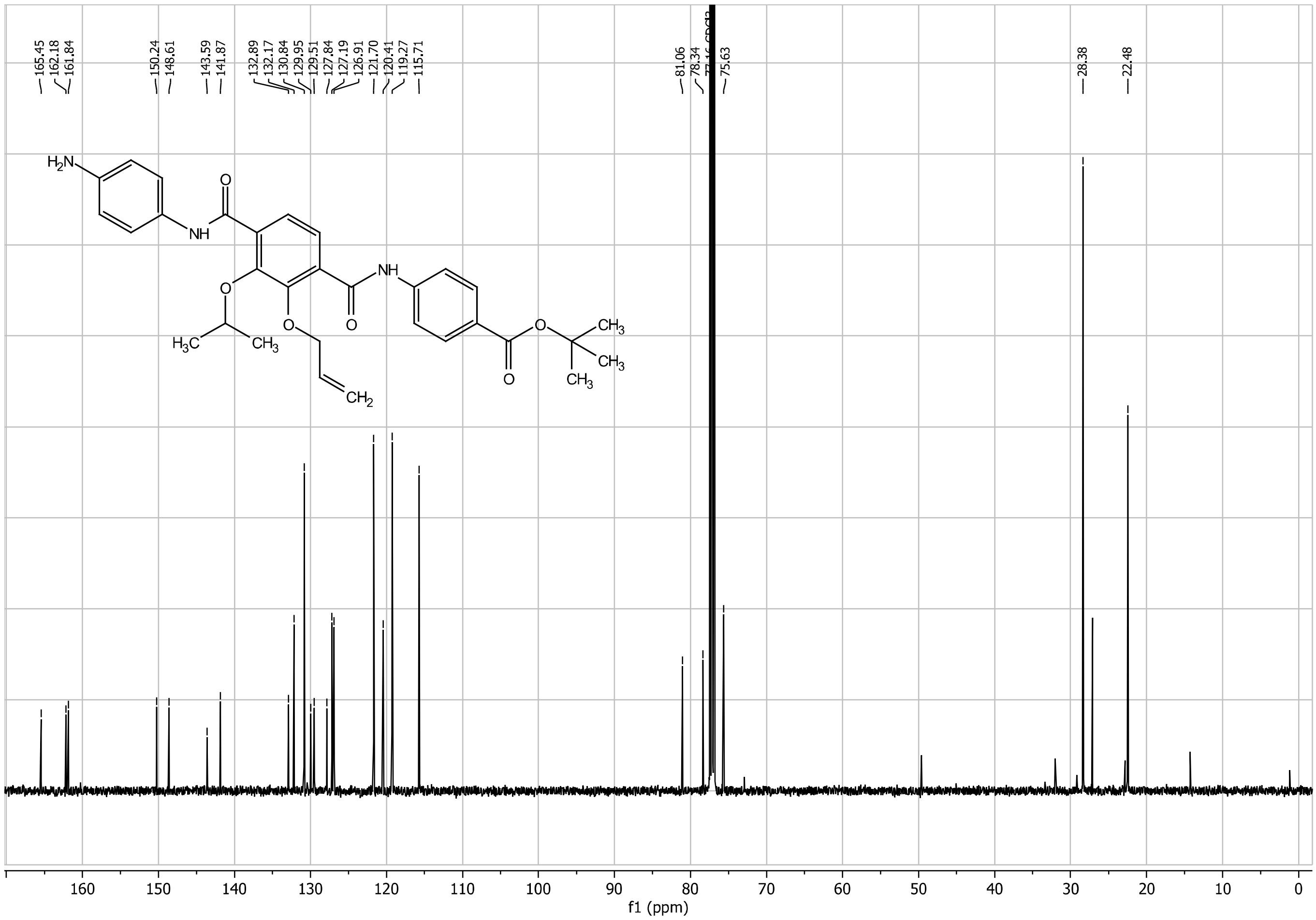
165.45
162.18
161.84
150.24
148.61
143.59
141.87
132.89
132.17
130.84
129.95
129.51
127.84
127.19
126.91
121.70
120.41
119.27
115.71
81.06
78.34
75.63
28.38
22.48
H2N
O
NH
NH
O
O
O
H3C
CH3
CH2
O
O
CH3
CH3
CH3
f1 (ppm)

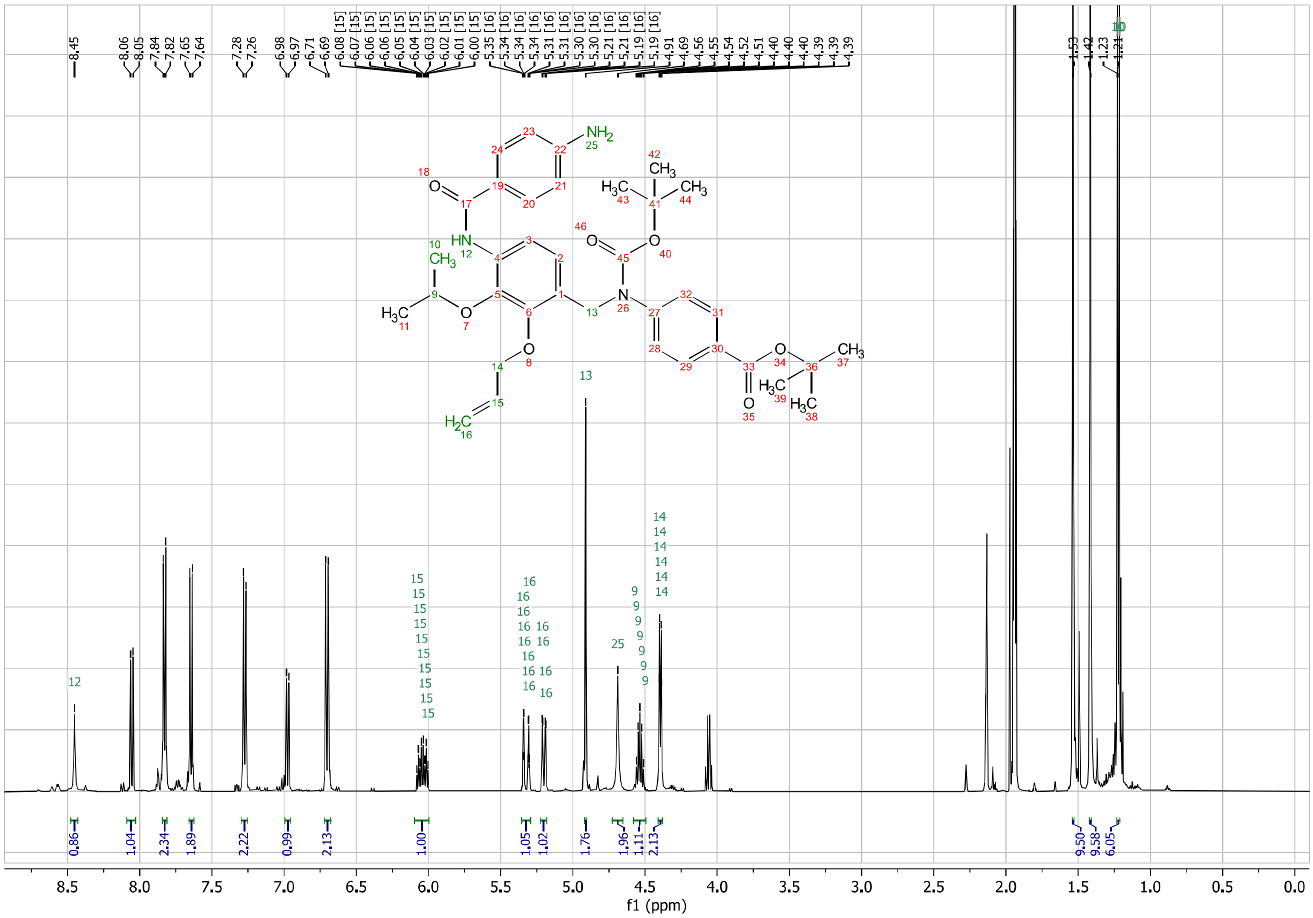
8.45
8.06
8.05
7.84
7.82
7.65
7.64
7.28
7.26
6.98
6.97
6.71
6.69
1.53
1.42
1.23
1.21
0.86
1.04
2.34
1.89
2.22
0.99
2.13
1.00
1.05
1.02
1.76
1.96
1.11
2.13
9.50
9.58
6.05
f1 (ppm)

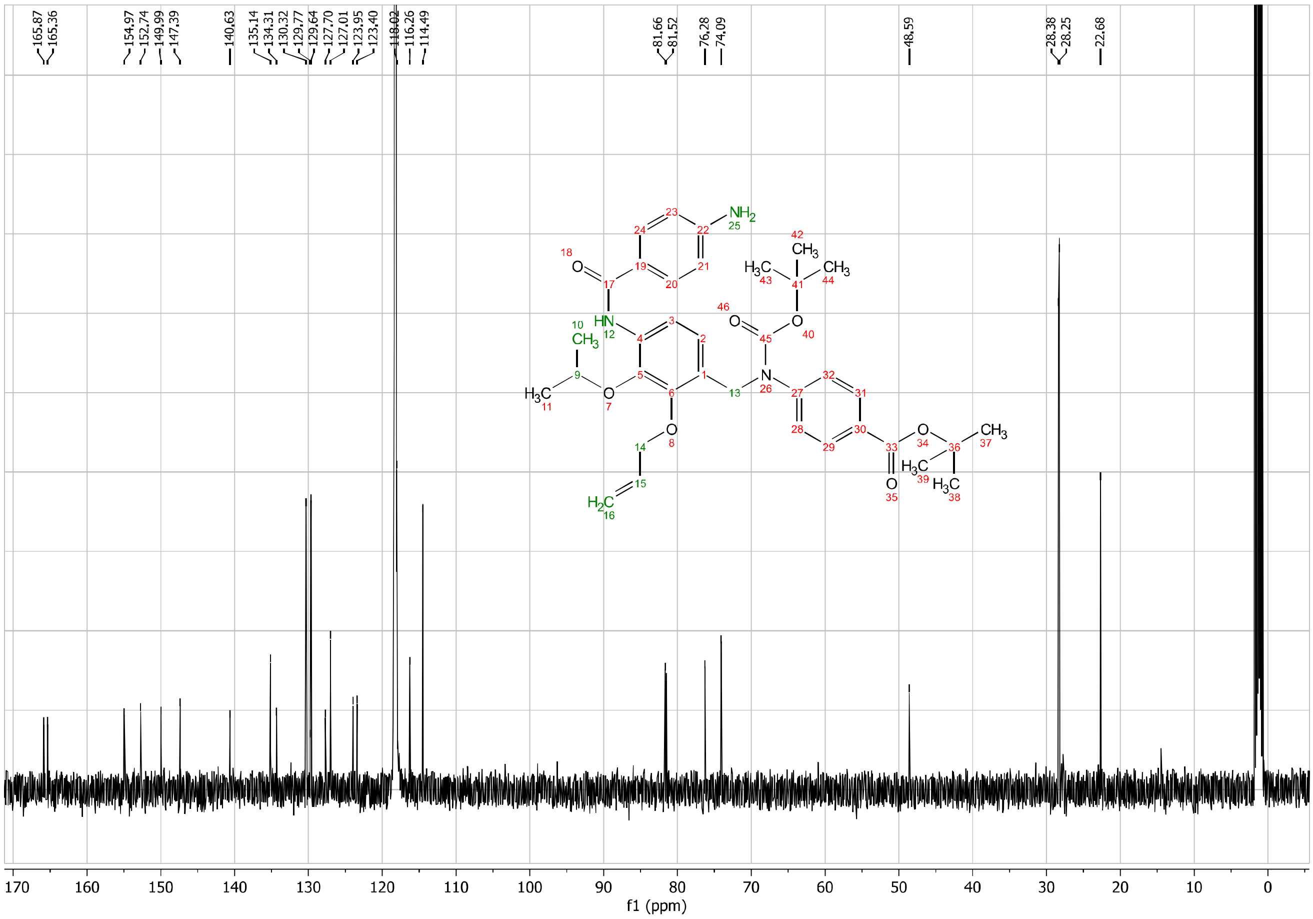
165.87
165.36
154.97
152.74
149.99
147.39
140.63
135.14
134.31
130.32
129.77
129.64
127.70
127.01
123.95
123.40
118.02
116.26
114.49
81.66
81.52
76.28
74.09
48.59
28.38
28.25
22.68
f1 (ppm)

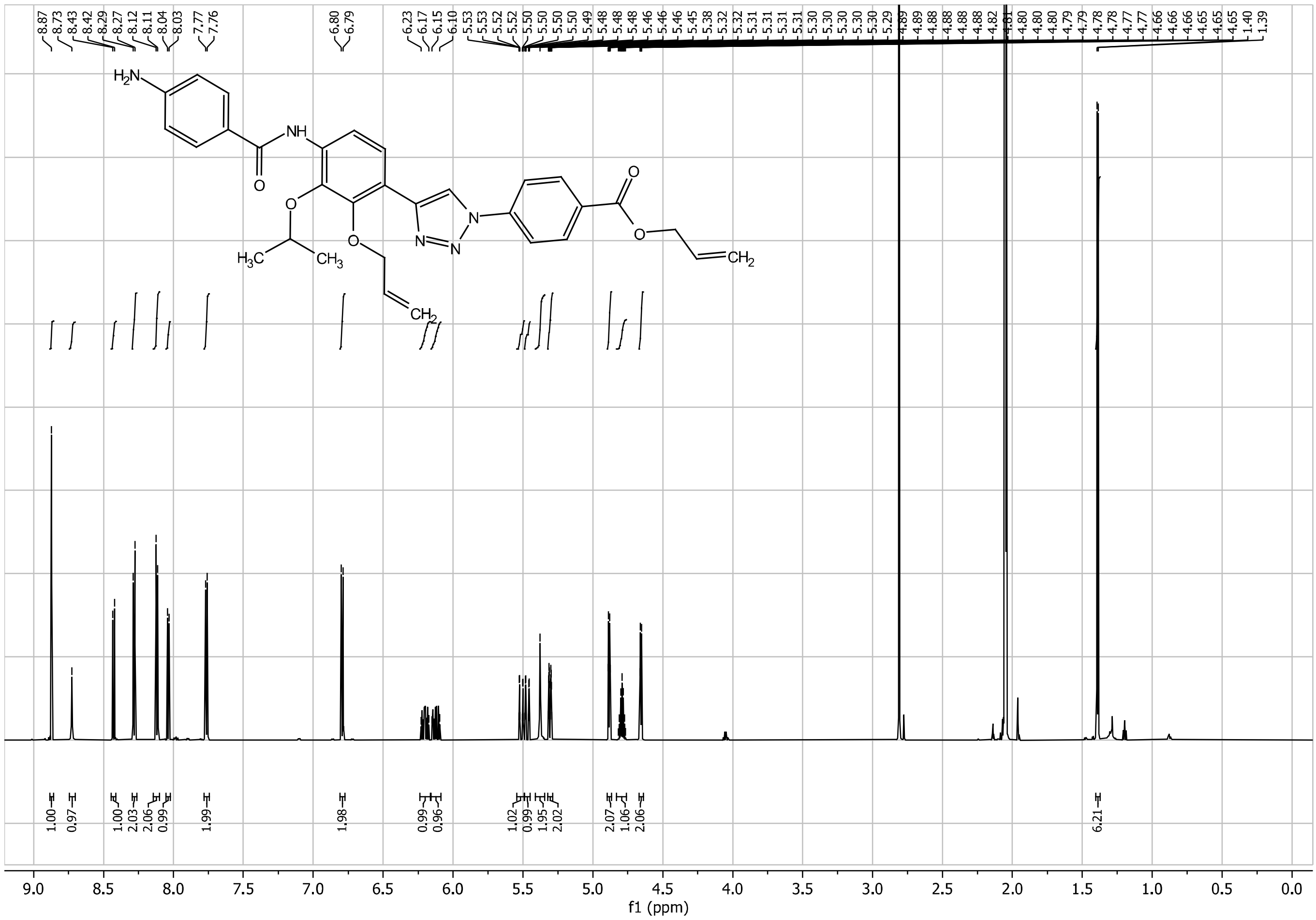
8.87 8.73 8.43 8.42 8.29 8.27 8.12 8.11 8.04 8.03 7.77 7.76
6.80 6.79
6.23 6.17 6.15 6.10
5.53 5.53 5.52 5.52 5.50 5.50 5.50 5.50 5.49 5.48 5.48 5.48 5.46 5.46 5.45 5.38 5.32 5.32 5.31 5.31 5.31 5.30 5.30 5.30 5.30 5.29
4.89 4.89 4.88 4.88 4.88 4.88 4.88 4.82 4.81 4.80 4.80 4.80 4.79 4.79 4.78 4.78 4.77 4.66 4.66 4.65 4.65 4.65
1.40 1.39
H2N
NH
O
O
H3C
CH3
O
N
N
N
O
O
CH2
CH2
1.00 0.97 1.00 2.03 2.06 0.99 1.99 1.98 0.99 0.96 1.02 0.99 1.95 2.02 2.07 1.06 2.06 6.21
f1 (ppm)

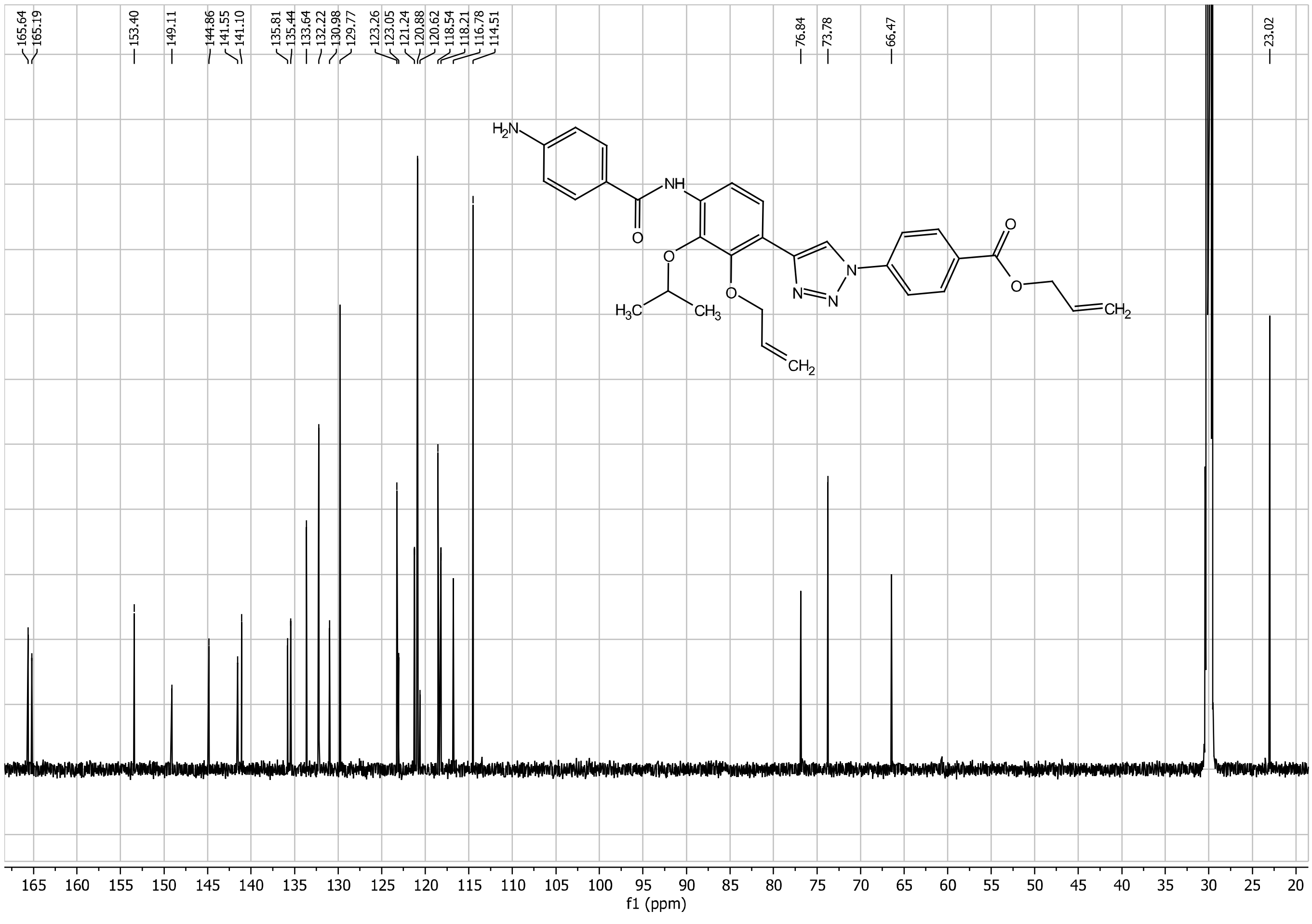
165.64 165.19 153.40 149.11 144.86 141.55 141.10 135.81 135.44 133.64 132.22 130.98 129.77 123.26 123.05 121.24 120.88 120.62 118.54 118.21 116.78 114.51
76.84 73.78 66.47 23.02
H2N
NH
O
O
H3C
CH3
O
N
N
N
O
O
CH2
CH2
f1 (ppm)

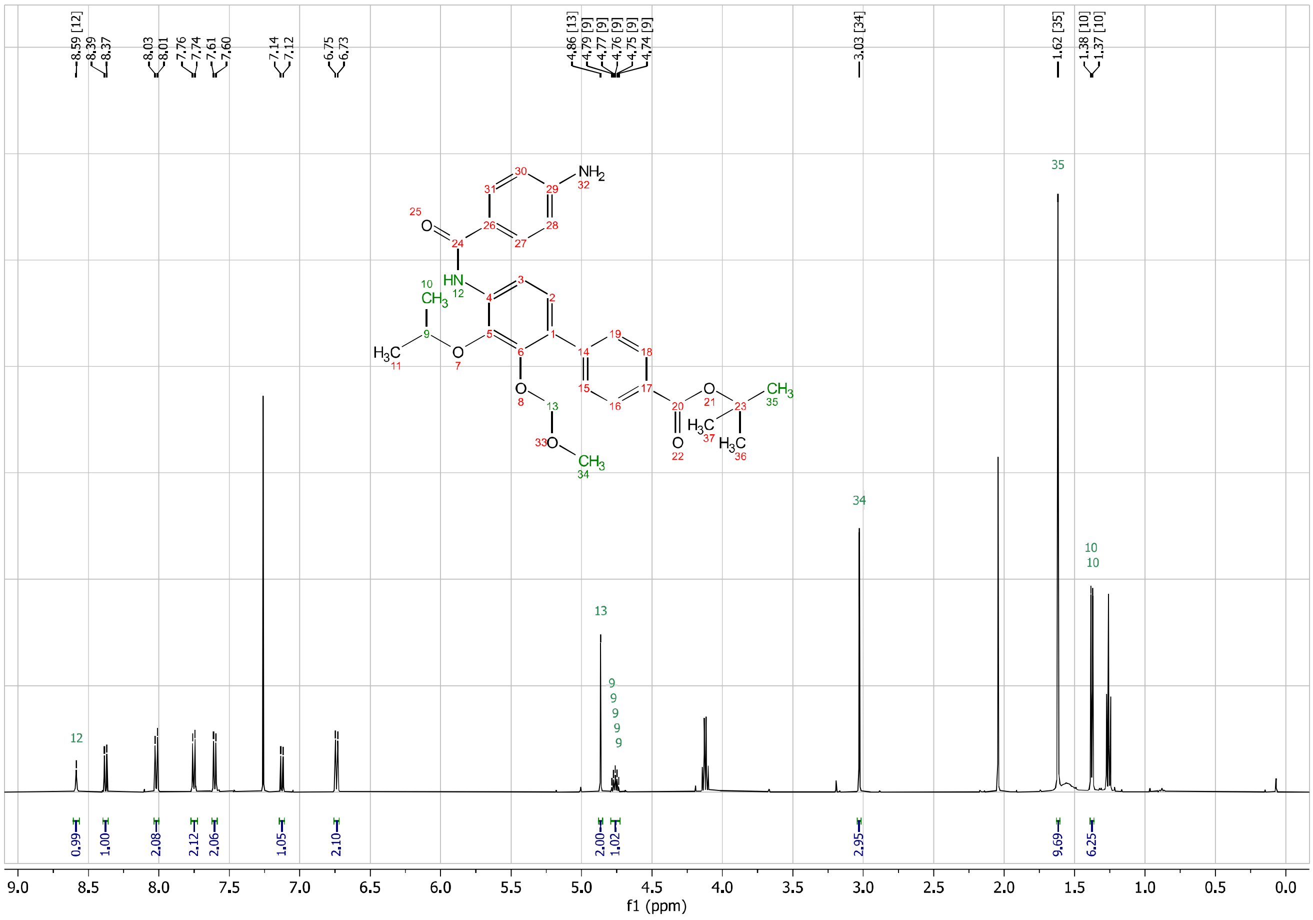
8.59 [12]
8.39
8.37
8.03
8.01
7.76
7.74
7.61
7.60
7.14
7.12
6.75
6.73
4.86 [13]
4.79 [9]
4.77 [9]
4.76 [9]
4.75 [9]
4.74 [9]
3.03 [34]
1.62 [35]
1.38 [10]
1.37 [10]
0.99
1.00
2.08
2.12
2.06
1.05
2.10
2.00
1.02
2.95
9.69
6.25
f1 (ppm)

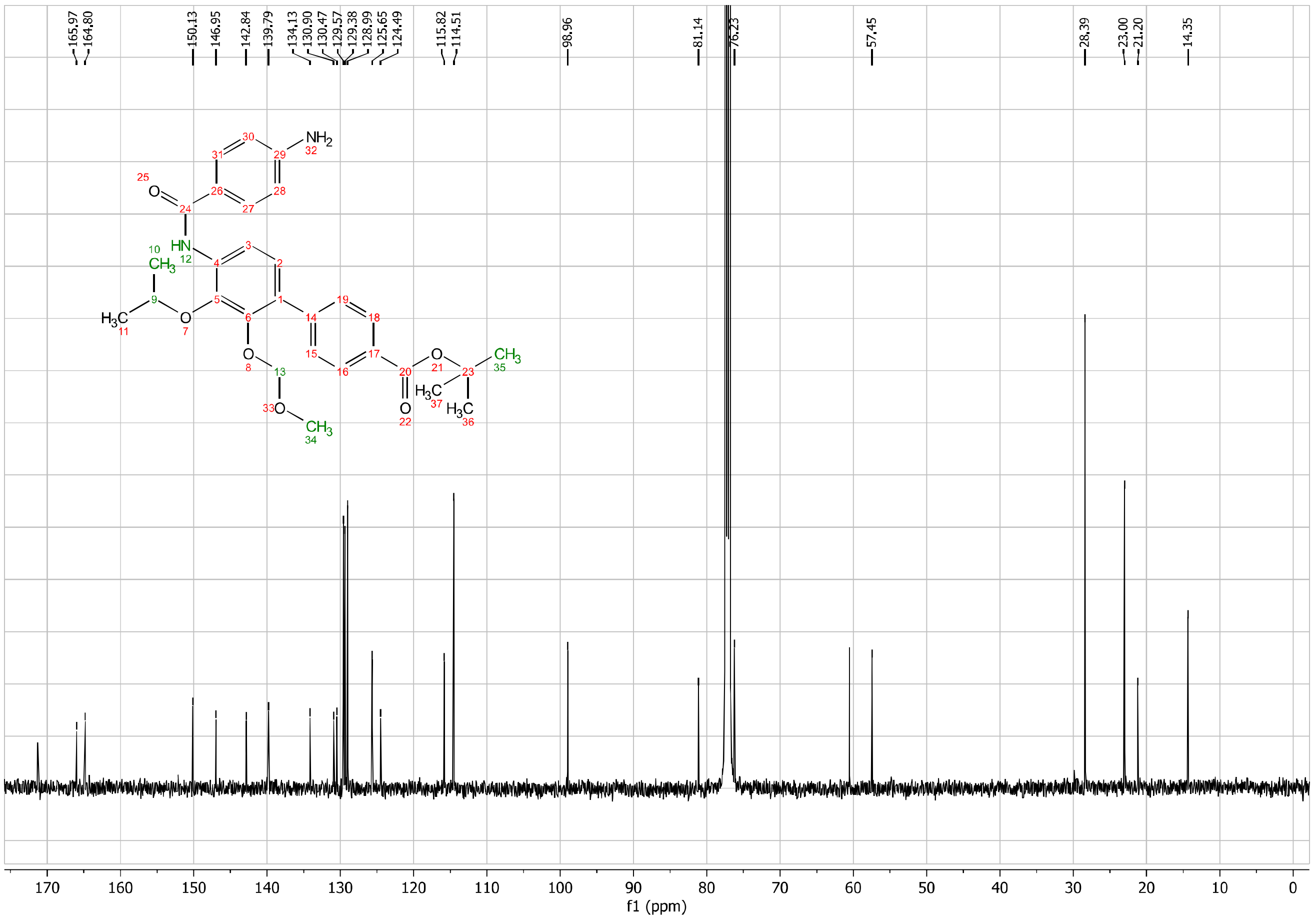
165.97
164.80
150.13
146.95
142.84
139.79
134.13
130.90
130.47
129.57
129.38
128.99
125.65
124.49
115.82
114.51
98.96
81.14
76.23
57.45
28.39
23.00
21.20
14.35
f1 (ppm)

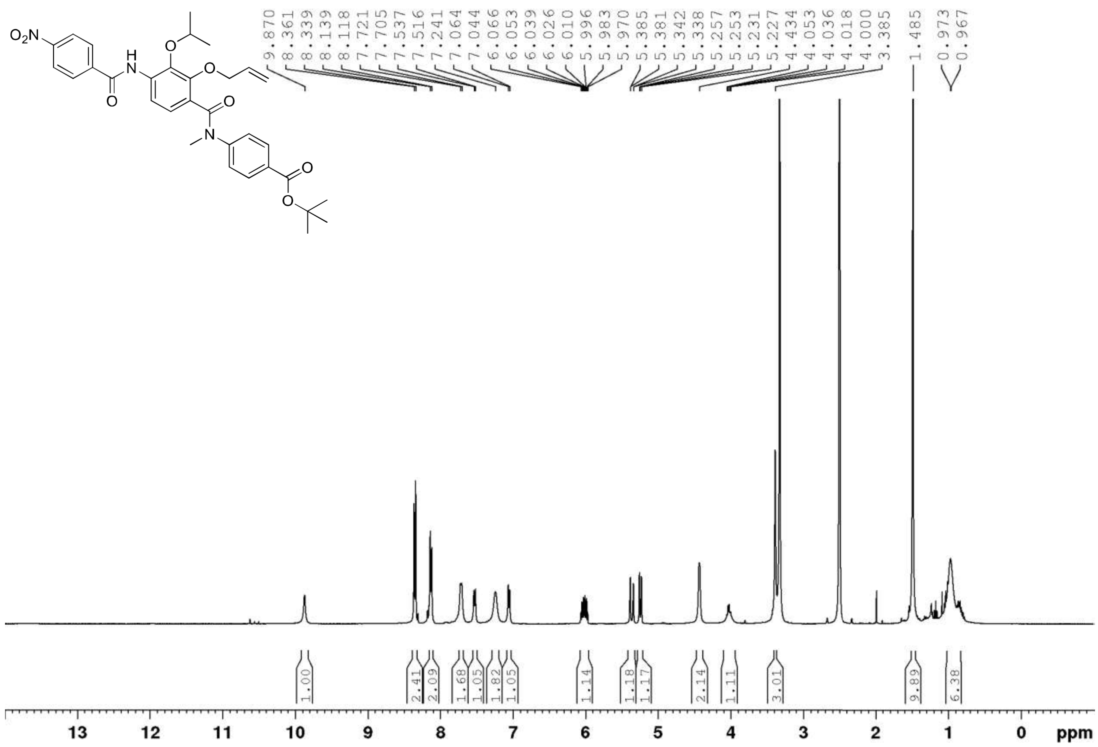
9.870
8.361
8.339
8.139
8.118
7.721
7.705
7.537
7.516
7.241
7.064
7.044
6.066
6.053
6.039
6.026
6.010
5.996
5.983
5.970
5.385
5.381
5.342
5.338
5.257
5.253
5.231
5.227
4.434
4.053
4.036
4.018
4.000
3.385
1.485
0.973
0.967
1.00
2.41
2.09
1.68
1.05
1.82
1.05
1.14
1.18
1.17
2.14
1.11
3.01
9.89
6.38
ppm

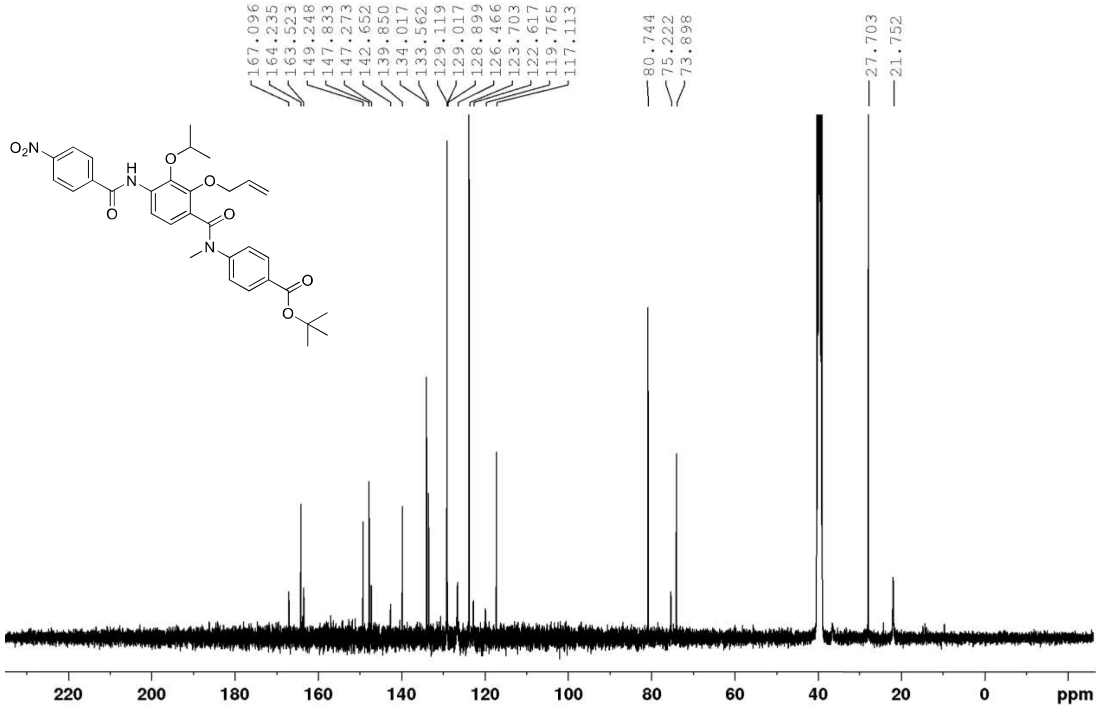
167.096
164.235
163.523
149.248
147.833
147.273
142.652
139.850
134.017
133.562
129.119
129.017
128.899
126.466
123.703
122.617
119.765
117.113
80.744
75.222
73.898
27.703
21.752
ppm

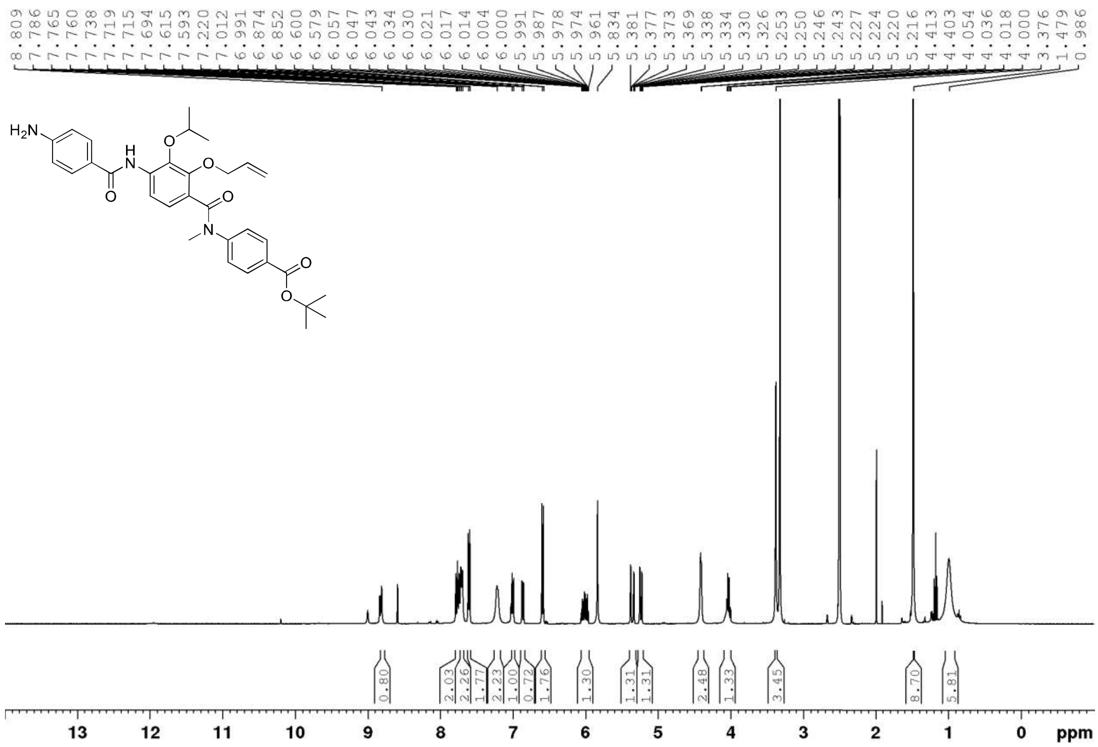
8.809 7.786 7.765 7.760 7.738 7.719 7.715 7.694 7.615 7.593 7.220 7.012 6.991 6.874 6.852 6.600 6.579 6.057 6.047 6.043 6.034 6.030 6.021 6.017 6.014 6.004 6.000 5.991 5.987 5.978 5.974 5.961 5.834 5.381 5.377 5.373 5.369 5.338 5.334 5.330 5.326 5.253 5.250 5.246 5.243 5.227 5.224 5.220 5.216 4.413 4.403 4.054 4.036 4.018 4.000 3.376 1.479 0.986
H₂N
H
N
O
0.80 2.03 2.26 1.77 2.23 1.00 0.72 1.76 1.30 1.31 1.31 2.48 1.33 3.45 8.70 5.81
13 12 11 10 9 8 7 6 5 4 3 2 1 0 ppm

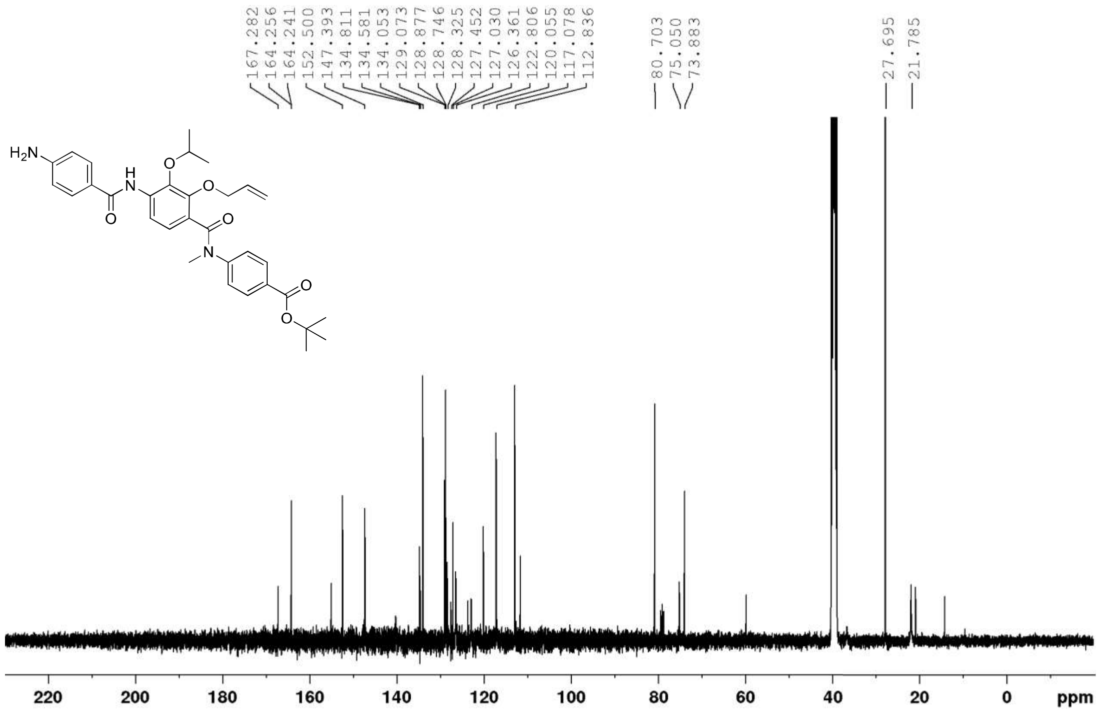
167.282 164.256 164.241 152.500 147.393 134.811 134.581 134.053 129.073 128.877 128.746 128.325 127.452 127.030 126.361 122.806 120.055 117.078 112.836
80.703 75.050 73.883
27.695 21.785
H₂N
H
N
O
220 200 180 160 140 120 100 80 60 40 20 0 ppm

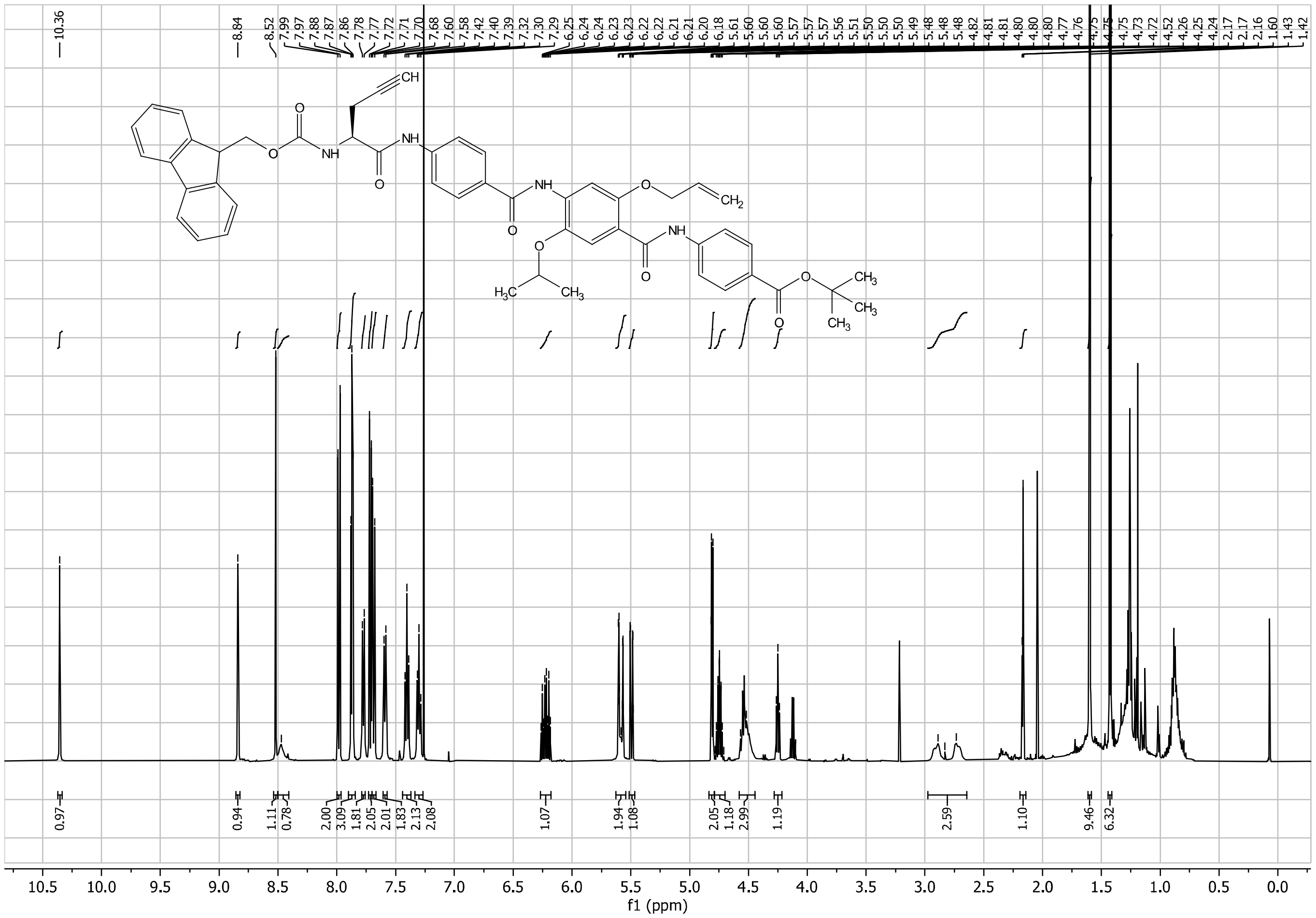
f1 (ppm)

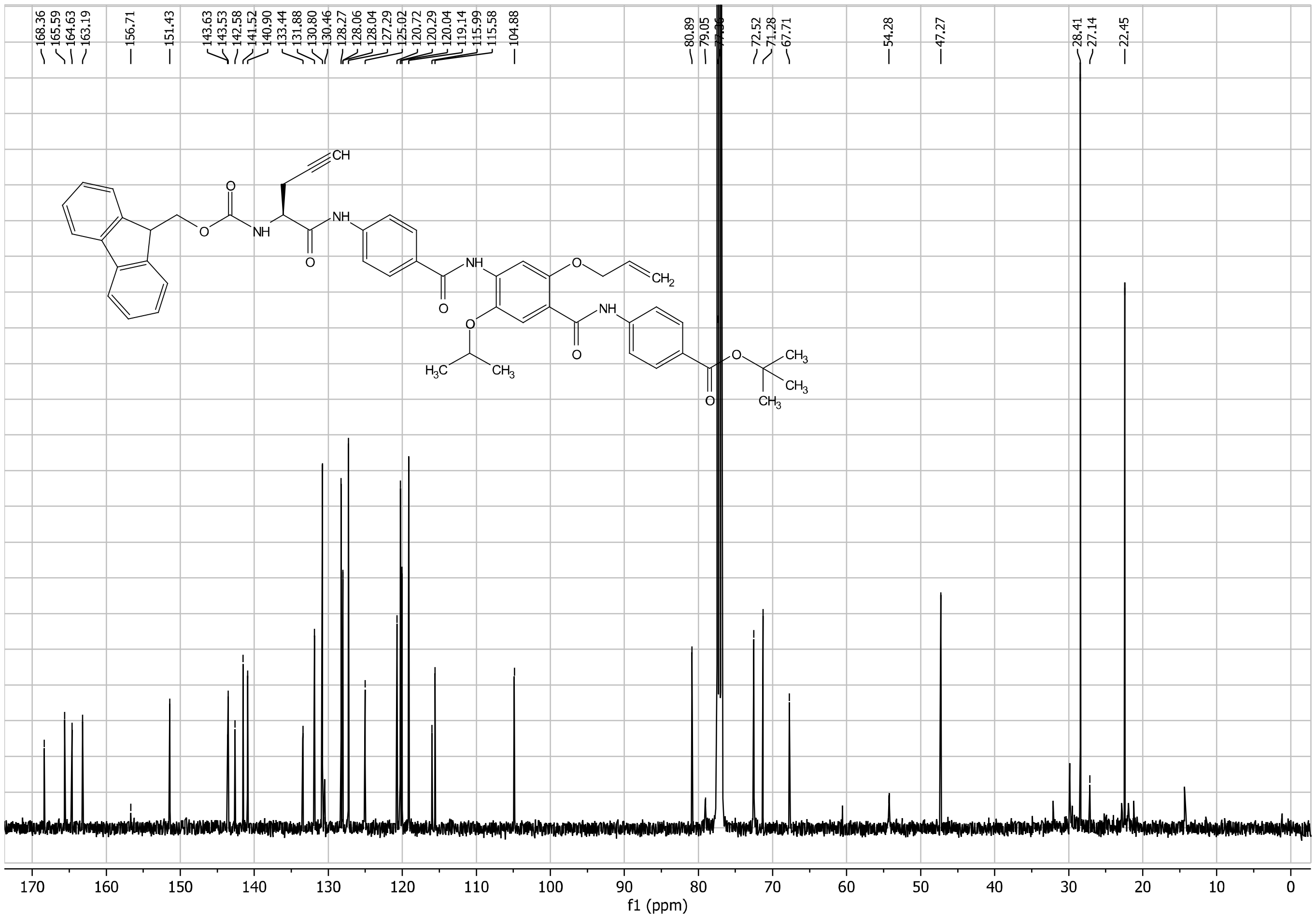
f1 (ppm)

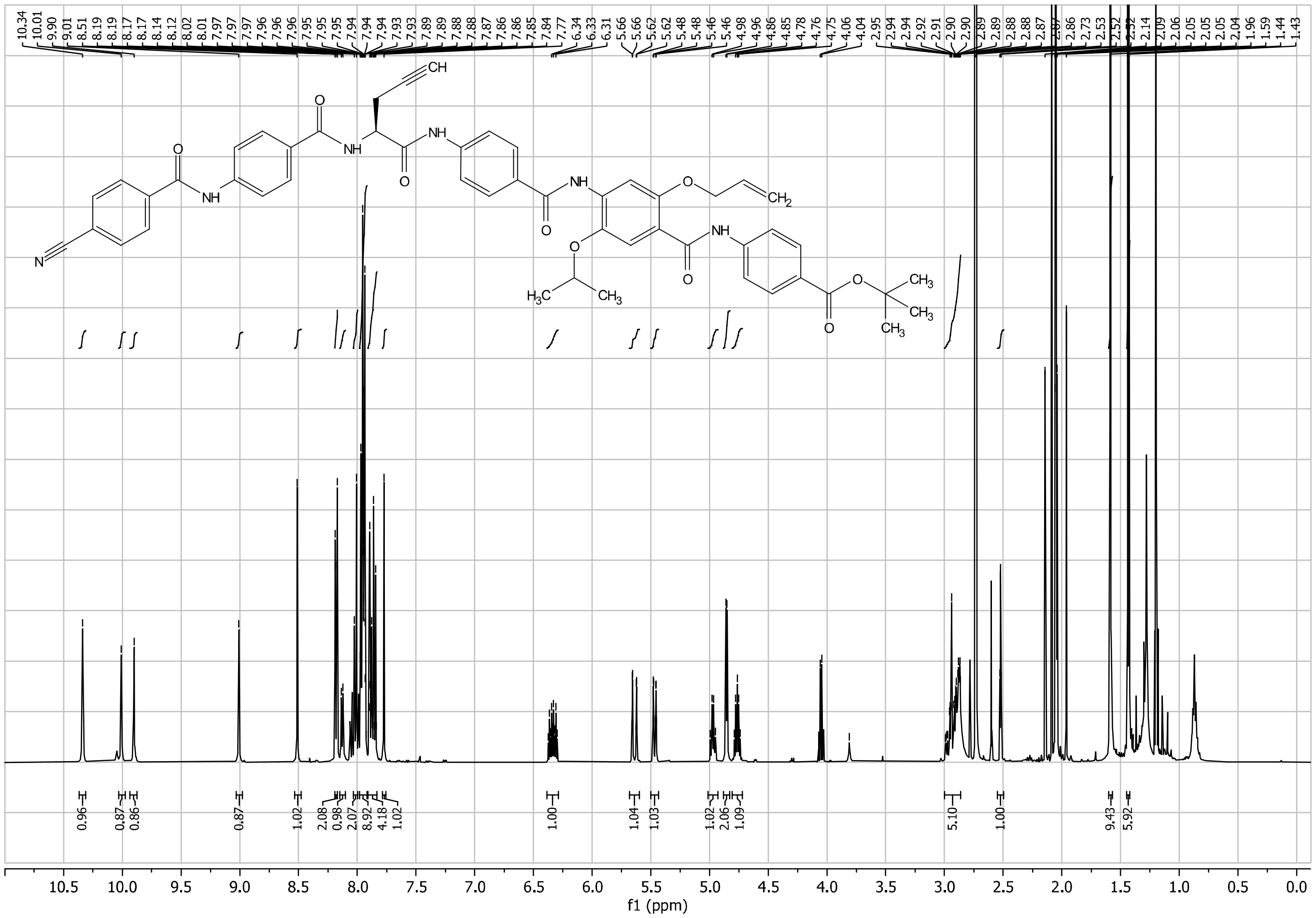

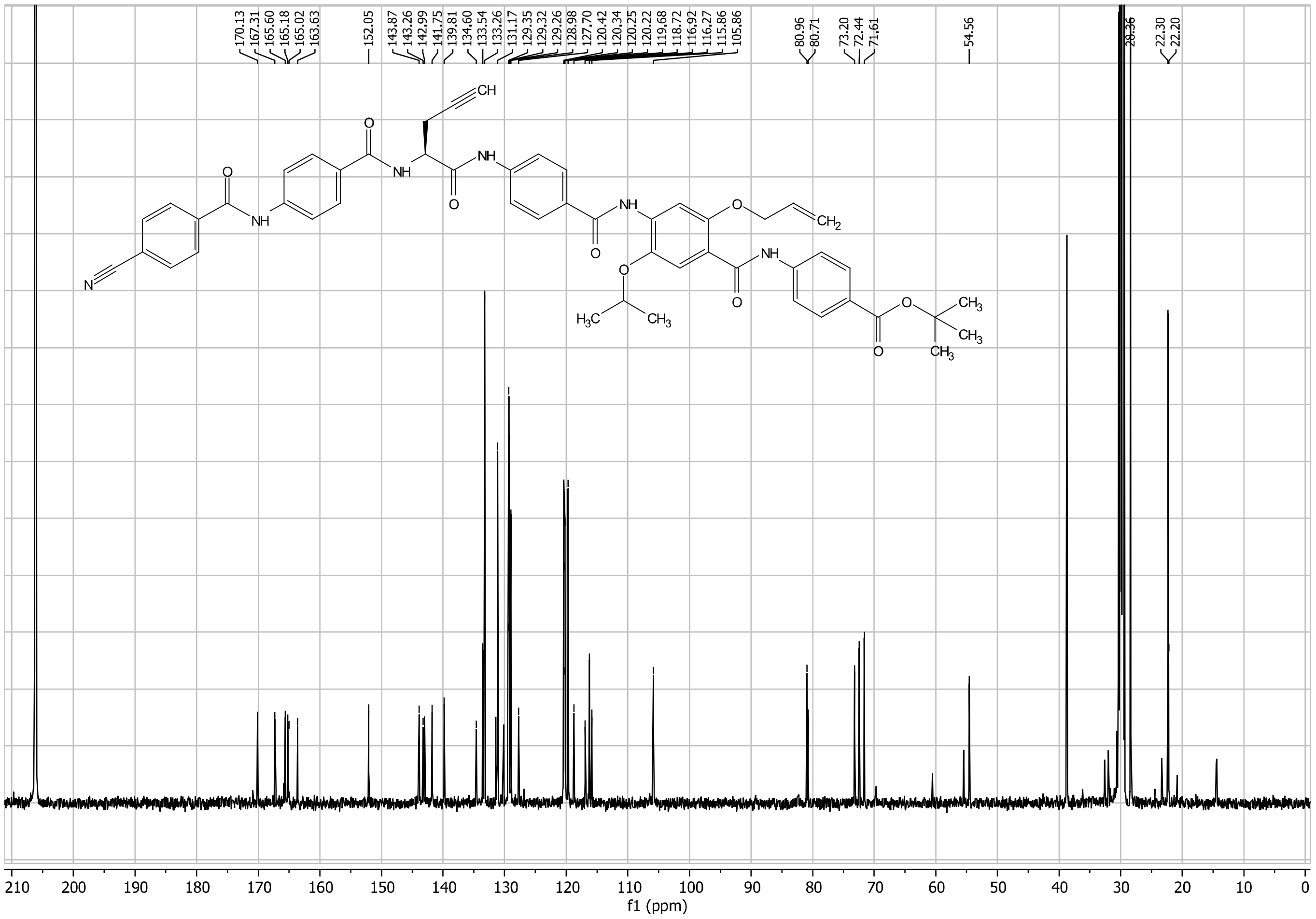

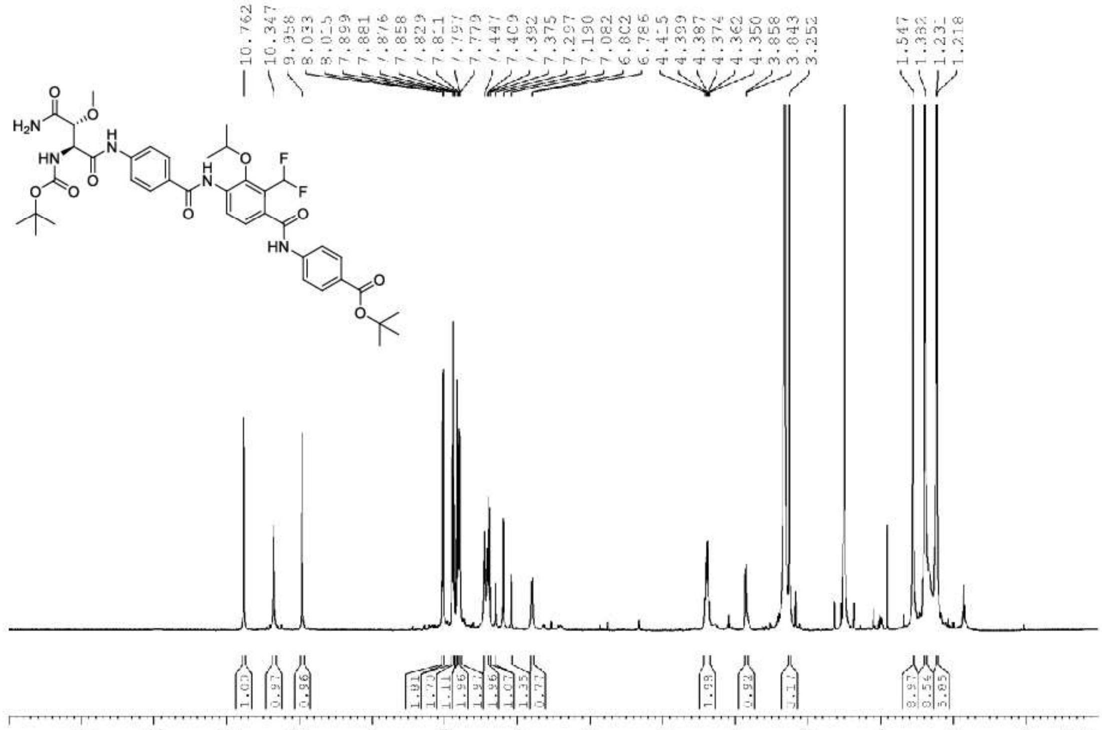
13 12 11 10 9 8 7 6 5 4 3 2 1 0 ppm

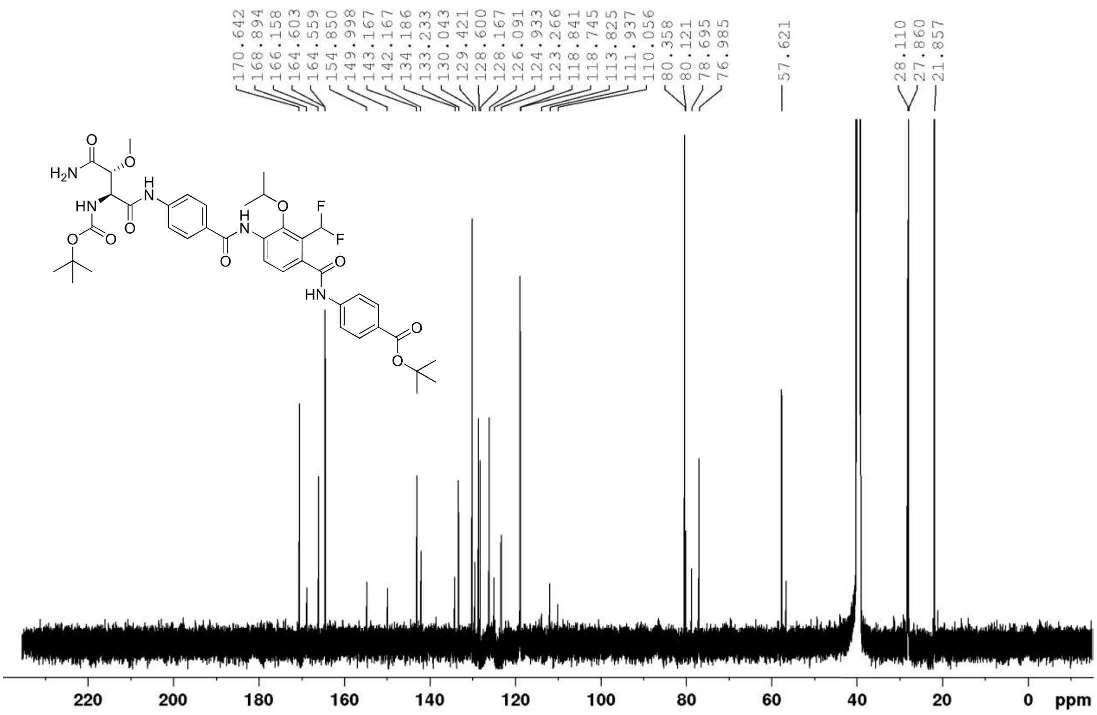
170.642
168.894
166.158
164.603
164.559
154.850
149.998
143.167
142.167
134.186
133.233
130.043
129.421
128.600
128.167
126.091
124.933
123.266
118.841
118.745
113.825
111.937
110.056
80.358
80.121
78.695
76.985
57.621
28.110
27.860
21.857
220 200 180 160 140 120 100 80 60 40 20 0 ppm

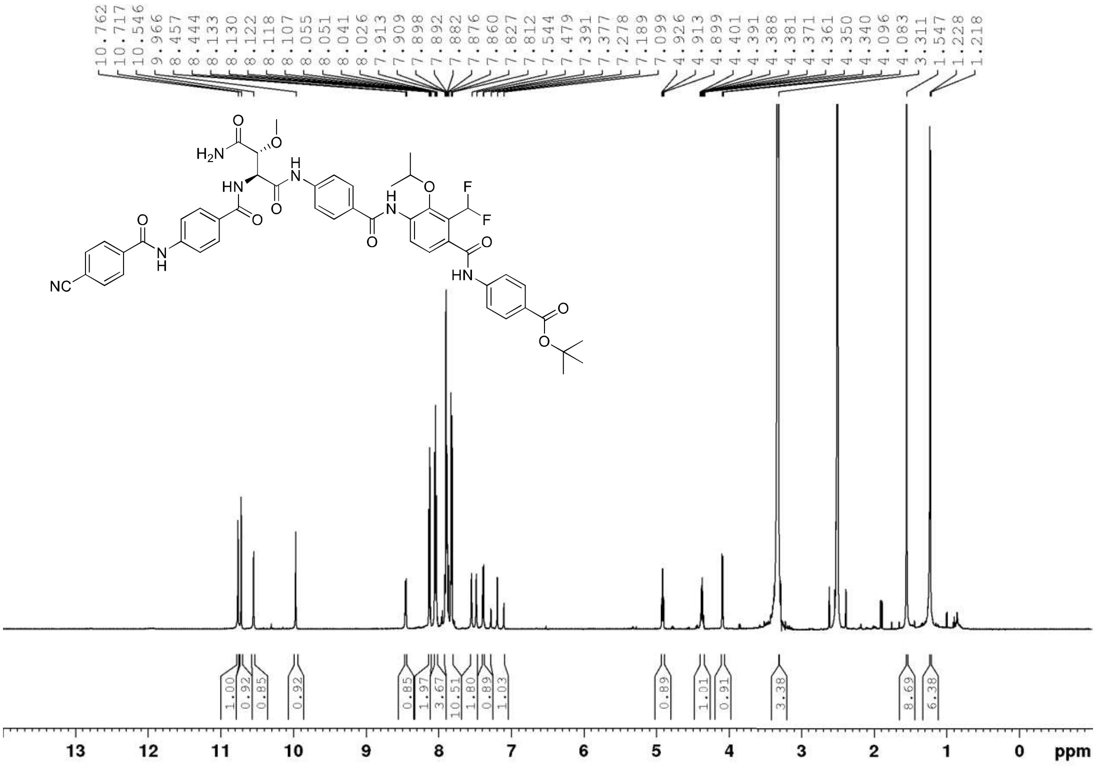
10.762
10.717
10.546
9.966
8.457
8.444
8.133
8.130
8.122
8.118
8.107
8.055
8.051
8.041
8.026
7.913
7.909
7.898
7.892
7.882
7.876
7.860
7.827
7.812
7.544
7.479
7.391
7.377
7.278
7.189
7.099
4.926
4.913
4.899
4.401
4.391
4.388
4.381
4.371
4.361
4.350
4.340
4.096
4.083
3.311
1.547
1.228
1.218
1.00 0.92 0.85 0.92 0.85 1.97 3.67 10.51 1.80 0.89 1.03 0.89 1.01 0.91 3.38 8.69 6.38
13 12 11 10 9 8 7 6 5 4 3 2 1 0 ppm

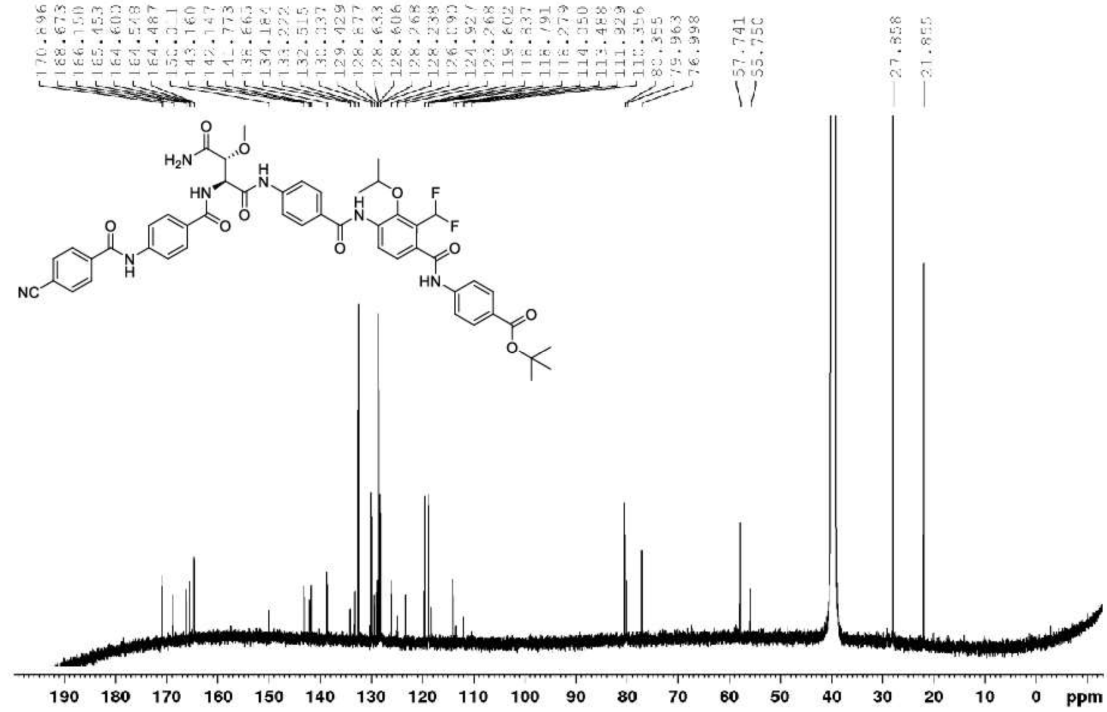
170.896
168.673
166.150
165.453
164.600
164.548
164.487
143.160
142.147
141.773
138.663
134.184
133.222
132.515
130.337
129.429
128.877
128.633
128.606
128.268
128.238
126.090
124.927
123.268
119.602
118.837
118.791
118.279
114.050
113.488
111.929
110.356
80.355
79.963
76.998
57.741
55.750
27.858
21.855
H2N
O
HN
NH
N
H
NC
F
ppm
190
180
170
160
150
140
130
120
110
100
90
80
70
60
50
40
30
20
10
0

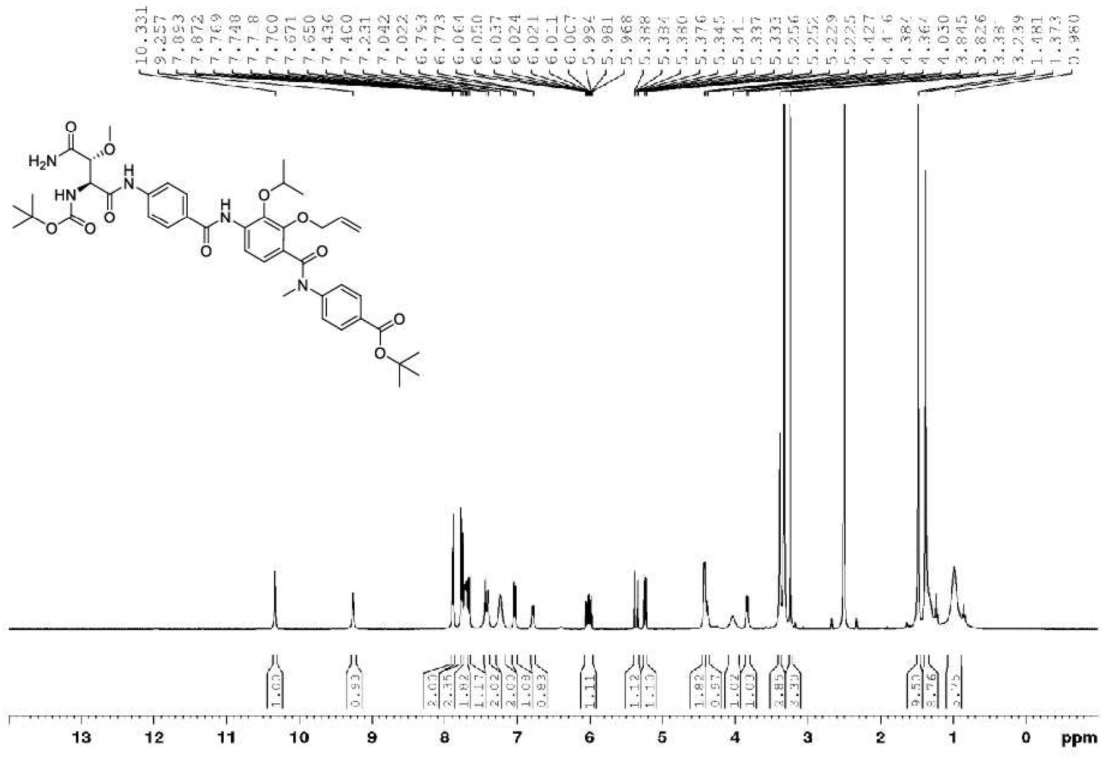
13 12 11 10 9 8 7 6 5 4 3 2 1 0 ppm

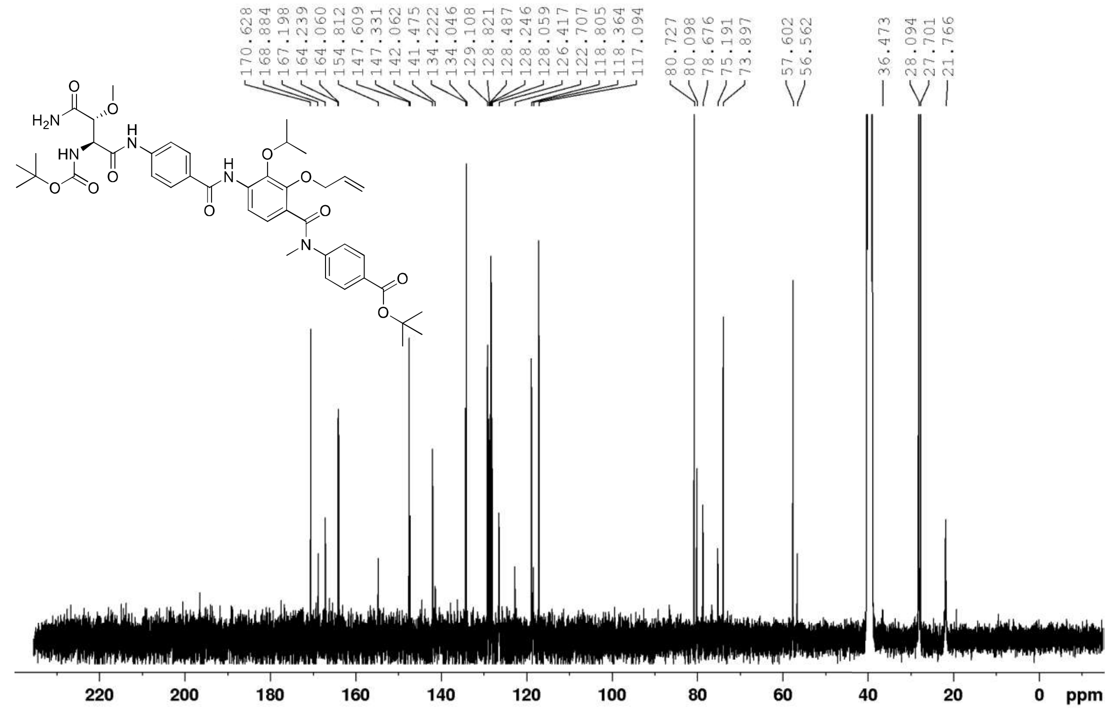

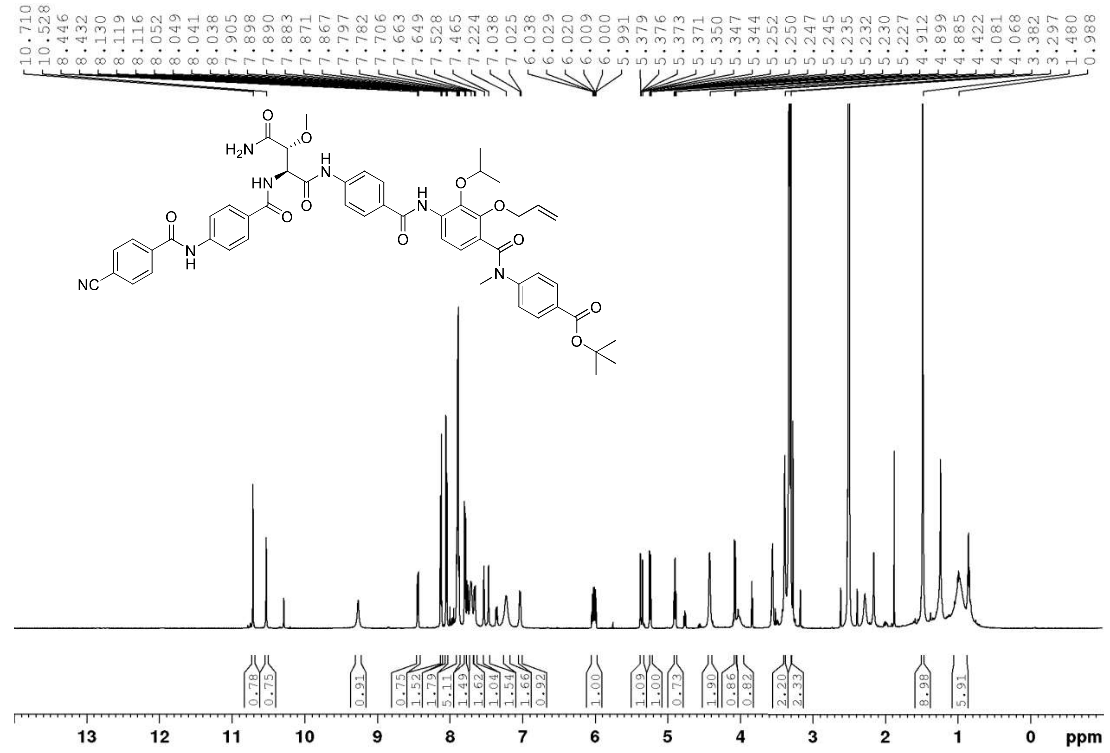

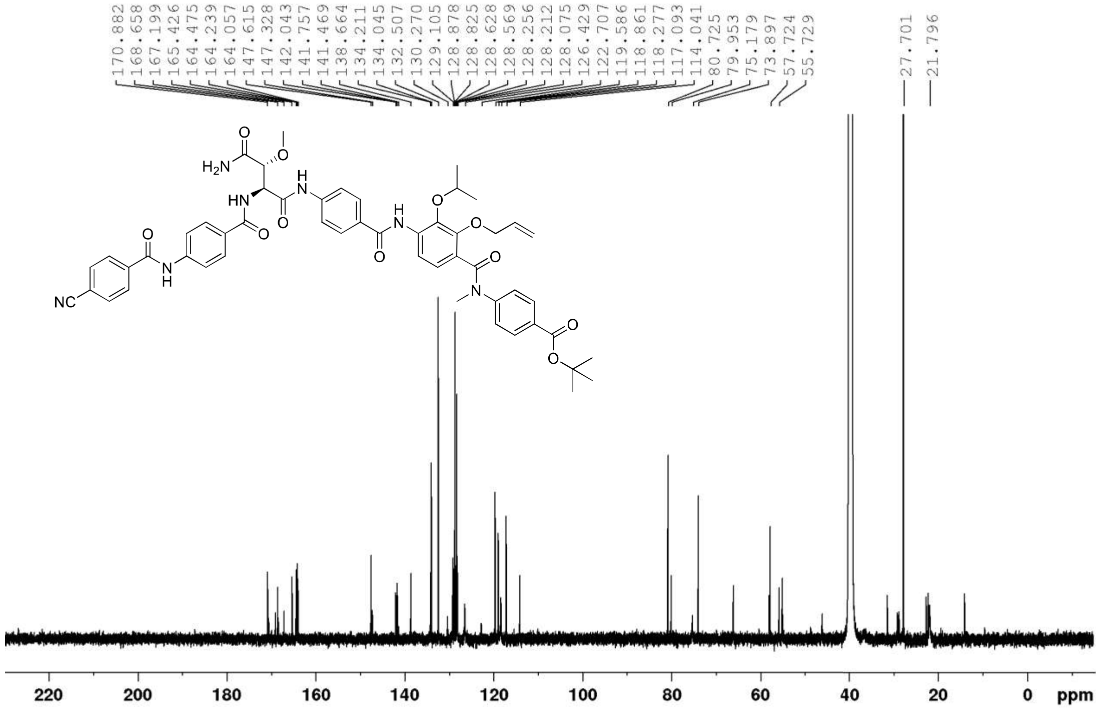
170.882
168.658
167.199
165.426
164.475
164.239
164.057
147.615
147.328
142.043
141.757
141.469
138.664
134.211
134.045
132.507
130.270
129.105
128.878
128.825
128.628
128.569
128.256
128.212
128.075
126.429
122.707
119.586
118.861
118.277
117.093
114.041
80.725
79.953
75.179
73.897
57.724
55.729
27.701
21.796
H2N
HN
NH
NC
220 200 180 160 140 120 100 80 60 40 20 0 ppm

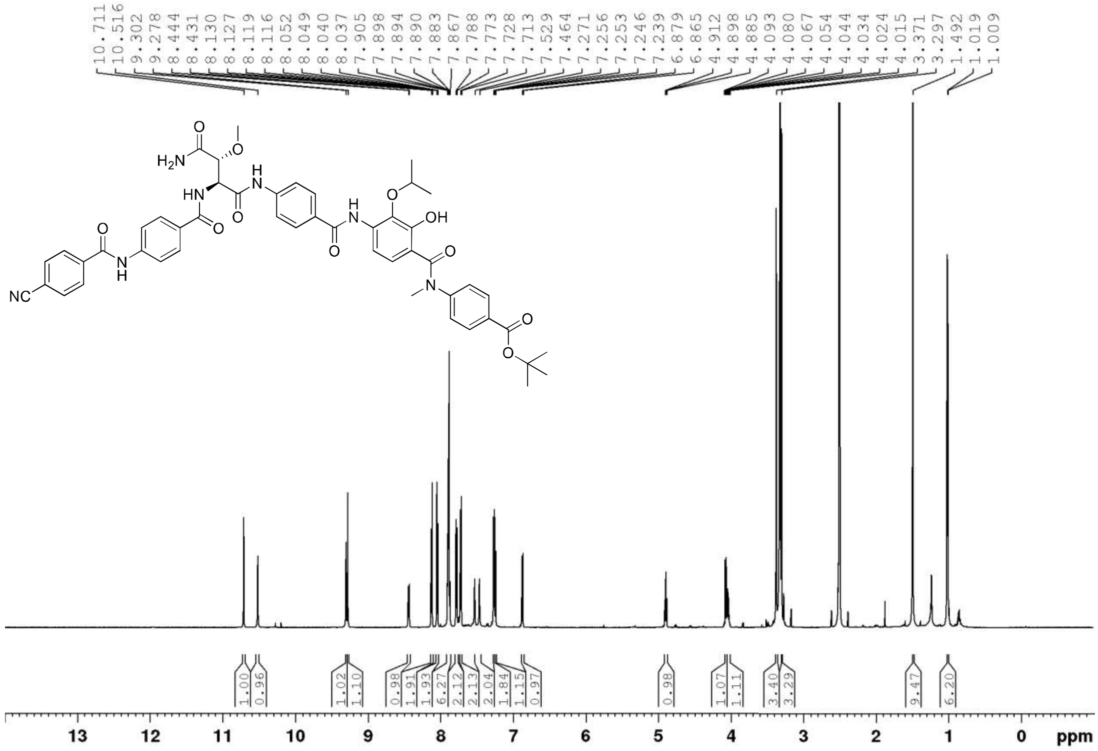
10.711
10.516
9.302
9.278
8.444
8.431
8.130
8.127
8.119
8.116
8.052
8.049
8.040
8.037
7.905
7.898
7.894
7.890
7.883
7.867
7.788
7.773
7.728
7.713
7.529
7.464
7.271
7.256
7.253
7.246
7.239
6.879
6.865
4.912
4.898
4.885
4.093
4.080
4.067
4.054
4.044
4.034
4.024
4.015
3.371
3.297
1.492
1.019
1.009
H2N
HN
NH
OH
NC
1.00
0.96
1.02
1.10
0.98
1.91
1.93
6.27
2.12
2.13
2.04
1.84
1.15
0.97
0.98
1.07
1.11
3.40
3.29
9.47
6.20
13 12 11 10 9 8 7 6 5 4 3 2 1 0 ppm

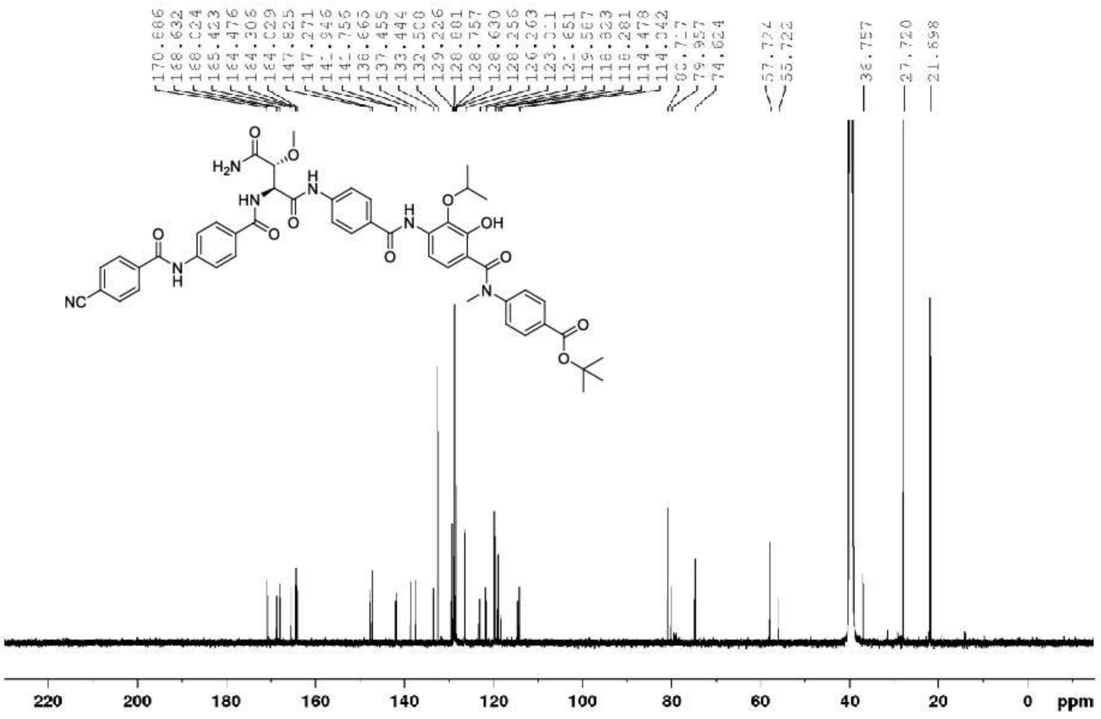
170.886
168.632
168.024
165.423
164.476
164.306
164.029
147.825
147.271
138.665
137.455
133.444
132.500
129.226
128.757
128.630
128.256
126.263
119.567
118.823
118.281
114.473
114.042
79.957
74.624
57.724
55.722
36.757
27.720
21.698
H2N
O
HN
NH
OH
NC
ppm
220
200
180
160
140
120
100
80
60
40
20
0

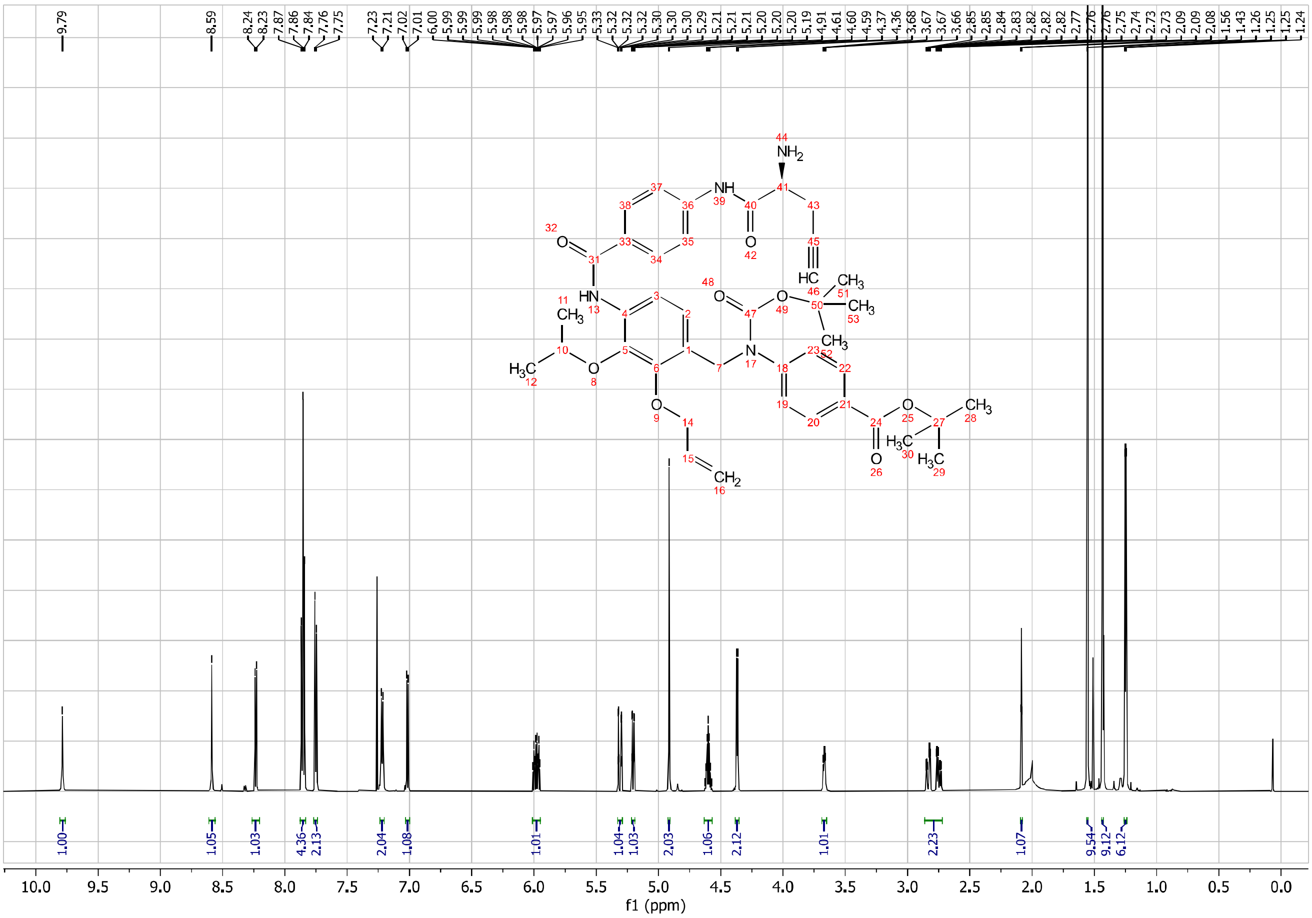
9.79 8.59 8.24 8.23 7.87 7.86 7.84 7.76 7.75 7.23 7.21 7.02 7.01 6.00 5.99 5.99 5.99 5.98 5.98 5.98 5.97 5.97 5.96 5.95 5.33 5.32 5.32 5.30 5.30 5.29 5.21 5.21 5.21 5.20 5.20 5.19 4.91 4.61 4.60 4.59 4.37 4.36 3.68 3.67 3.67 3.66 2.85 2.85 2.84 2.83 2.82 2.82 2.82 2.77 2.76 2.76 2.75 2.74 2.73 2.73 2.09 2.09 2.08 1.56 1.43 1.26 1.25 1.25 1.24
1.00 1.05 1.03 4.36 2.13 2.04 1.08 1.01 1.04 1.03 2.03 1.06 2.12 1.01 2.23 1.07 9.54 9.12 6.12
f1 (ppm)

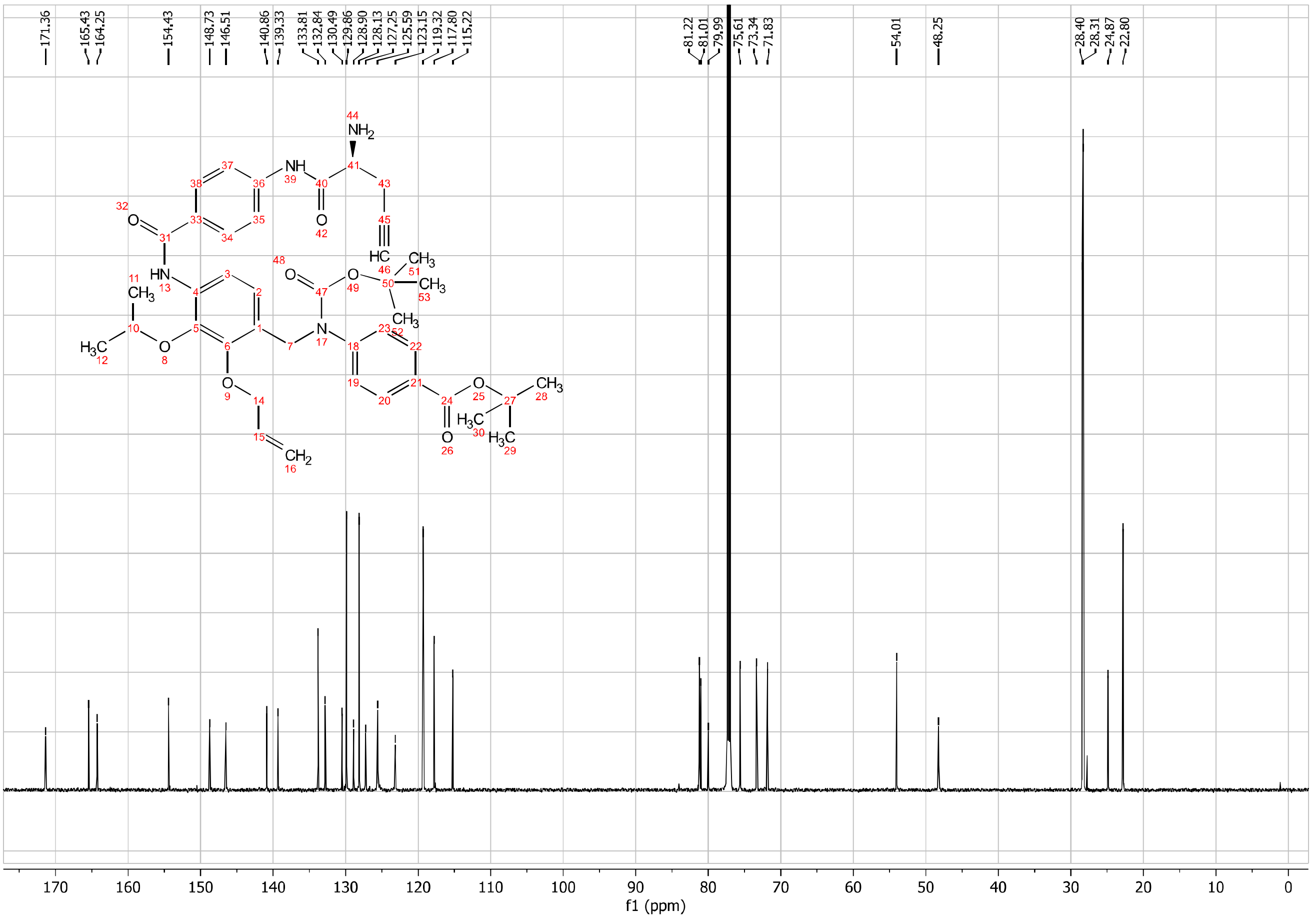
171.36 165.43 164.25 154.43 148.73 146.51 140.86 139.33 133.81 132.84 130.49 129.86 128.90 128.13 127.25 125.59 123.15 119.32 117.80 115.22 81.22 81.01 79.99 75.61 73.34 71.83 54.01 48.25 28.40 28.31 24.87 22.80
f1 (ppm)

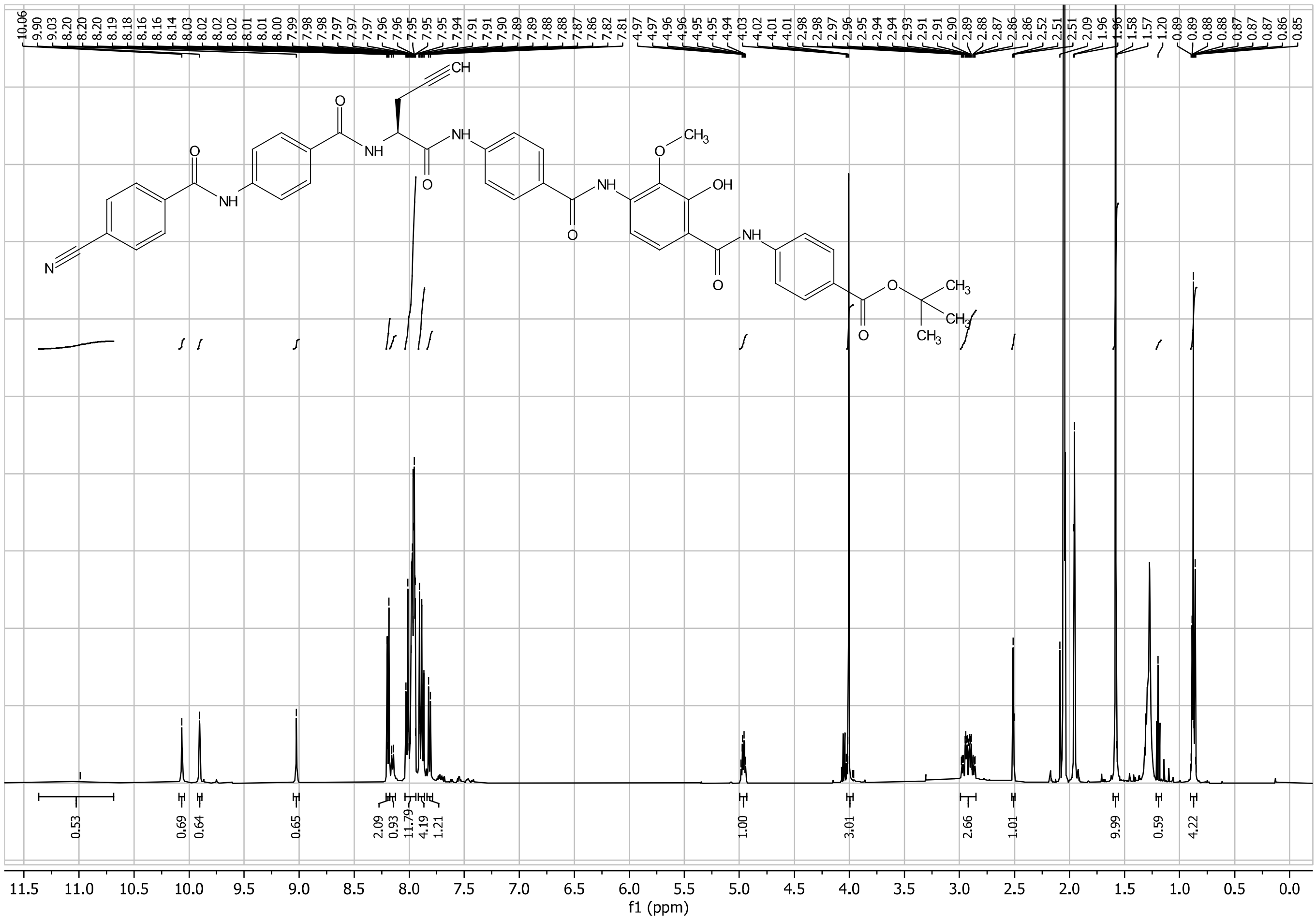
10.06 9.90 9.03 8.20 8.20 8.19 8.18 8.16 8.16 8.14 8.03 8.02 8.02 8.01 8.01 8.00 7.99 7.98 7.98 7.97 7.97 7.96 7.96 7.95 7.95 7.95 7.94 7.91 7.91 7.90 7.89 7.89 7.88 7.87 7.86 7.82 7.81 4.97 4.97 4.96 4.96 4.95 4.95 4.94 4.03 4.02 4.01 4.01 2.98 2.98 2.97 2.96 2.95 2.94 2.94 2.93 2.91 2.91 2.90 2.89 2.88 2.87 2.86 2.86 2.52 2.51 2.51 2.09 1.96 1.96 1.58 1.57 1.20 0.89 0.89 0.88 0.88 0.87 0.87 0.86 0.85
CH
O
NH
NH
O
NH
O
O
CH3
NH
OH
N
NH
O
O
O
CH3
CH3
O
CH3
0.53 0.69 0.64 0.65 2.09 0.93 11.79 4.19 1.21 1.00 3.01 2.66 1.01 9.99 0.59 4.22
11.5 11.0 10.5 10.0 9.5 9.0 8.5 8.0 7.5 7.0 6.5 6.0 5.5 5.0 4.5 4.0 3.5 3.0 2.5 2.0 1.5 1.0 0.5 0.0
f1 (ppm)

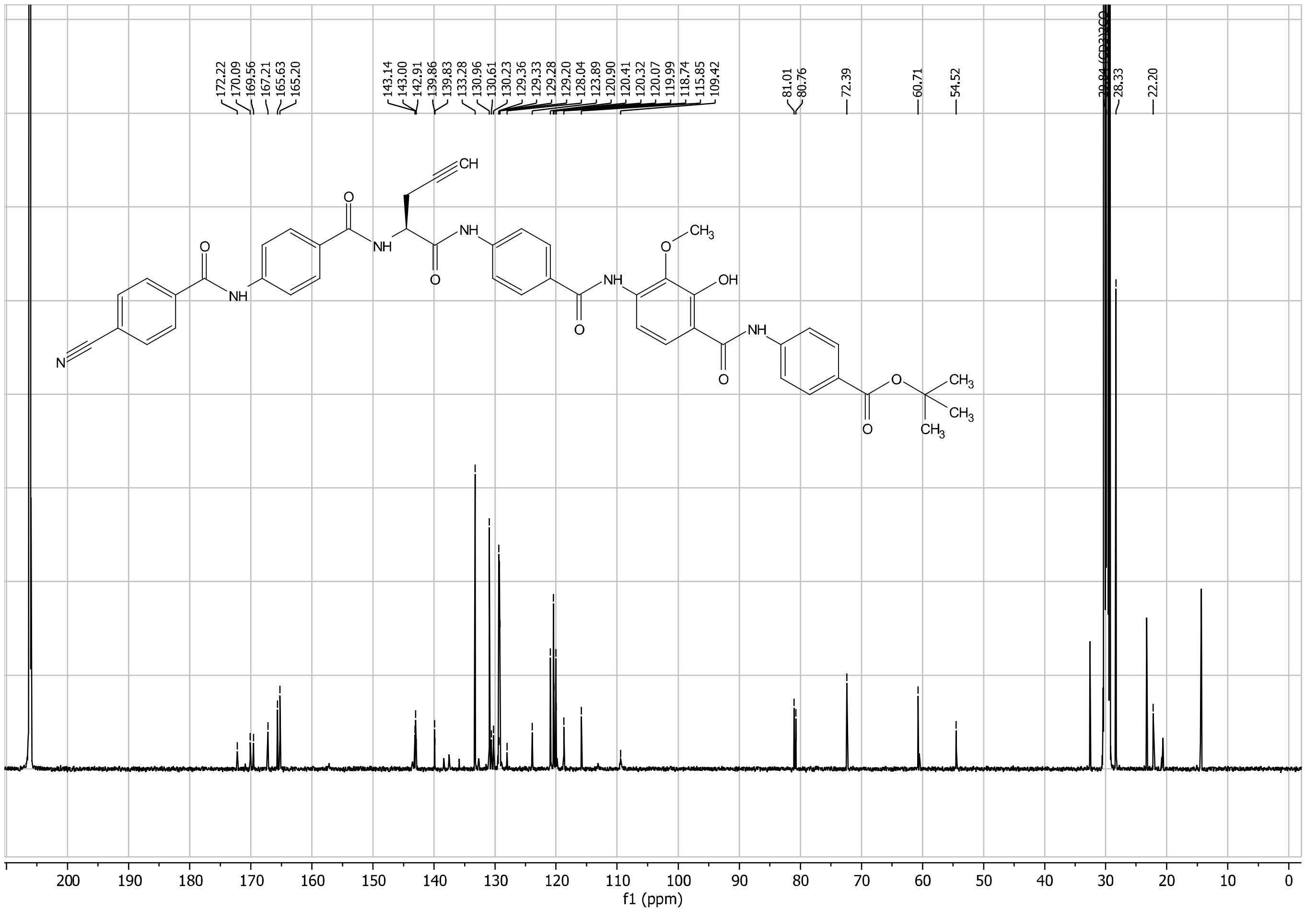
172.22 170.09 169.56 167.21 165.63 165.20 143.14 143.00 142.91 139.86 139.83 133.28 130.96 130.61 130.23 129.36 129.33 129.28 129.20 128.04 123.89 120.90 120.41 120.32 120.07 119.99 118.74 115.85 109.42 81.01 80.76 72.39 60.71 54.52 28.33 22.20
CH
O
NH
NH
O
NH
O
O
CH3
NH
OH
N
NH
O
O
O
CH3
CH3
O
CH3
200 190 180 170 160 150 140 130 120 110 100 90 80 70 60 50 40 30 20 10 0
f1 (ppm)

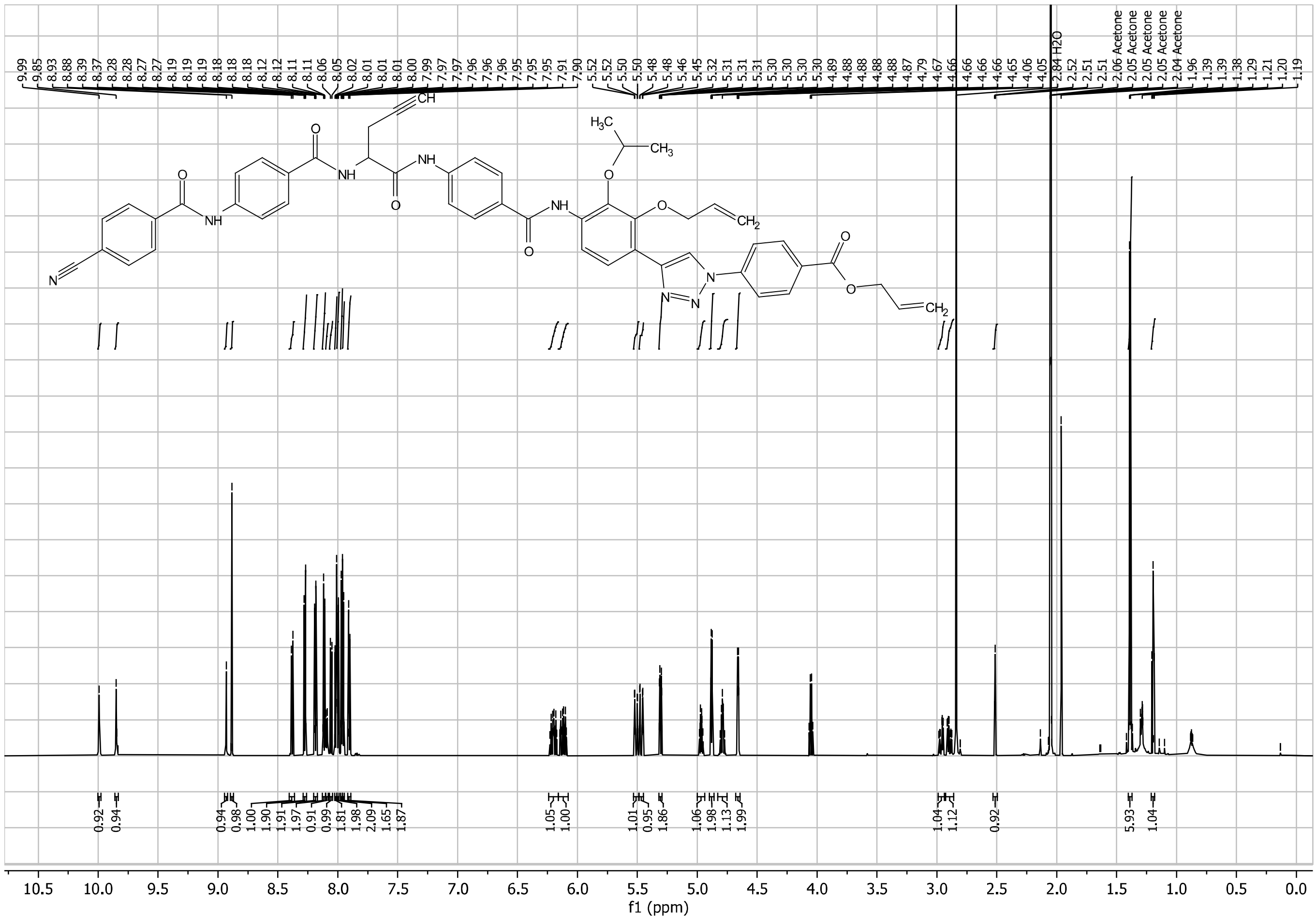
f1 (ppm)
2.84 H2O
2.06 Acetone
2.05 Acetone
2.05 Acetone
2.04 Acetone

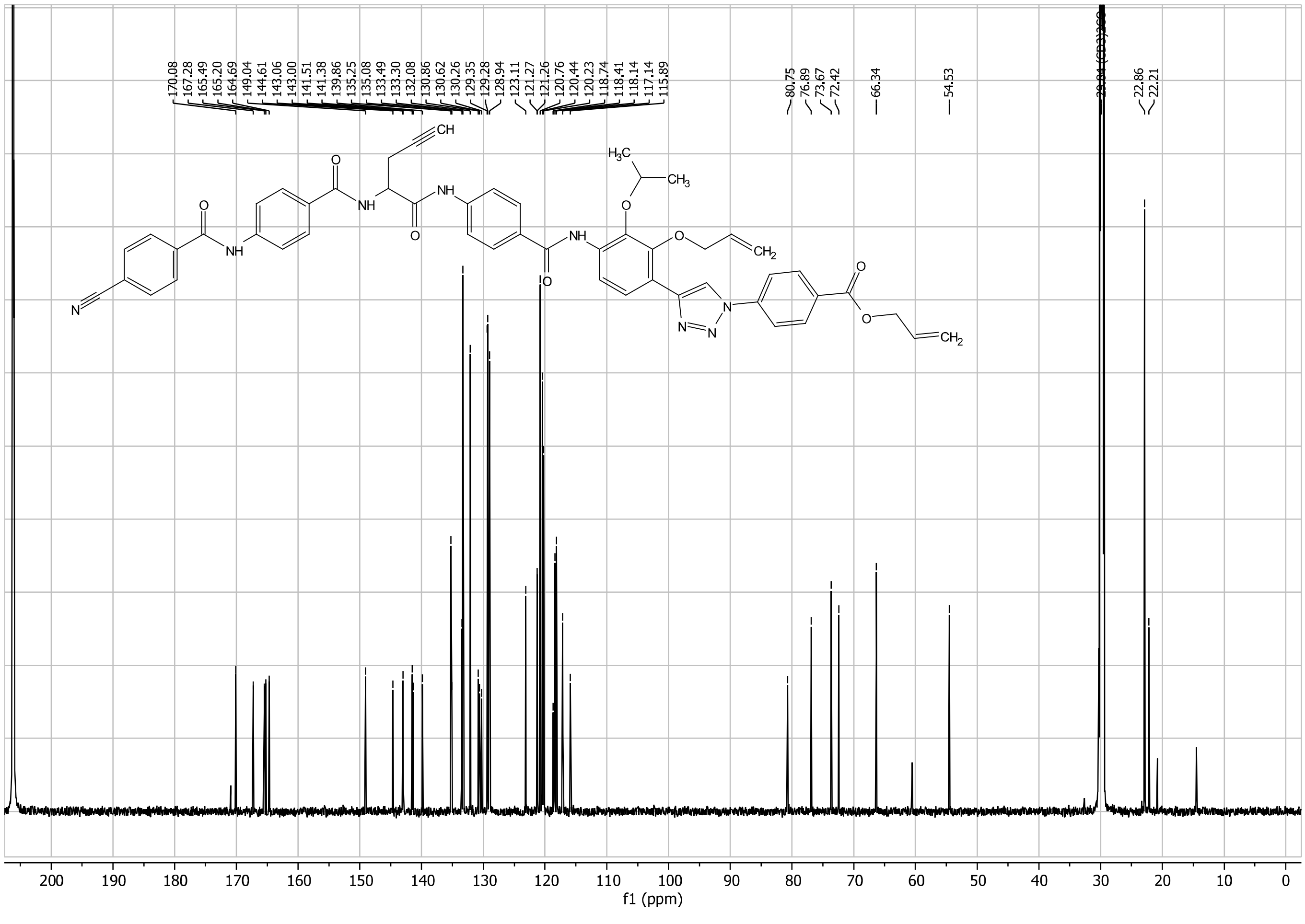
f1 (ppm)

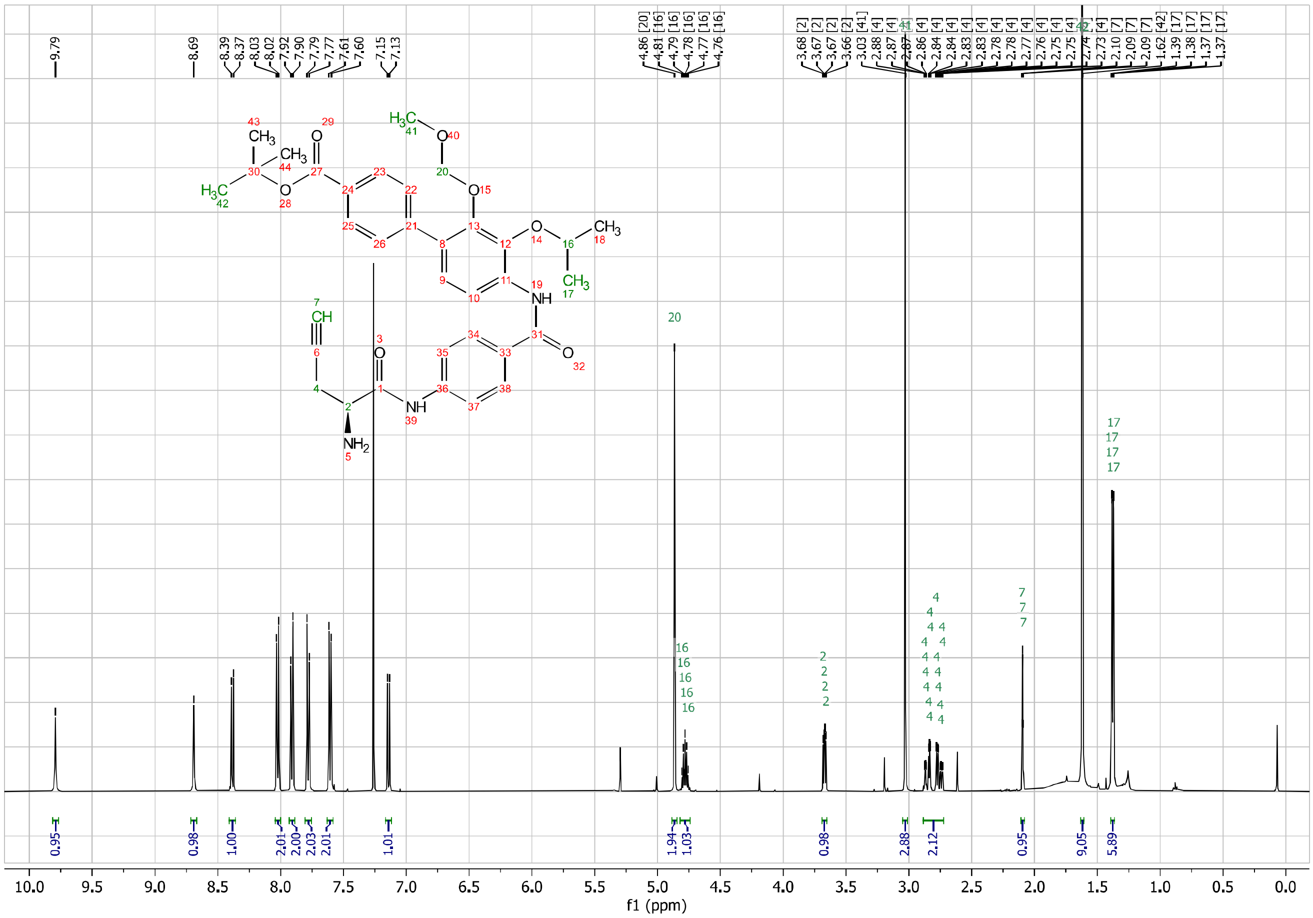
f1 (ppm)
9.79
8.69
8.39
8.37
8.03
8.02
7.92
7.90
7.79
7.77
7.61
7.60
7.15
7.13
0.95
0.98
1.00
2.01
2.00
2.03
2.01
1.01
1.94
1.03
0.98
2.88
2.12
0.95
9.05
5.89

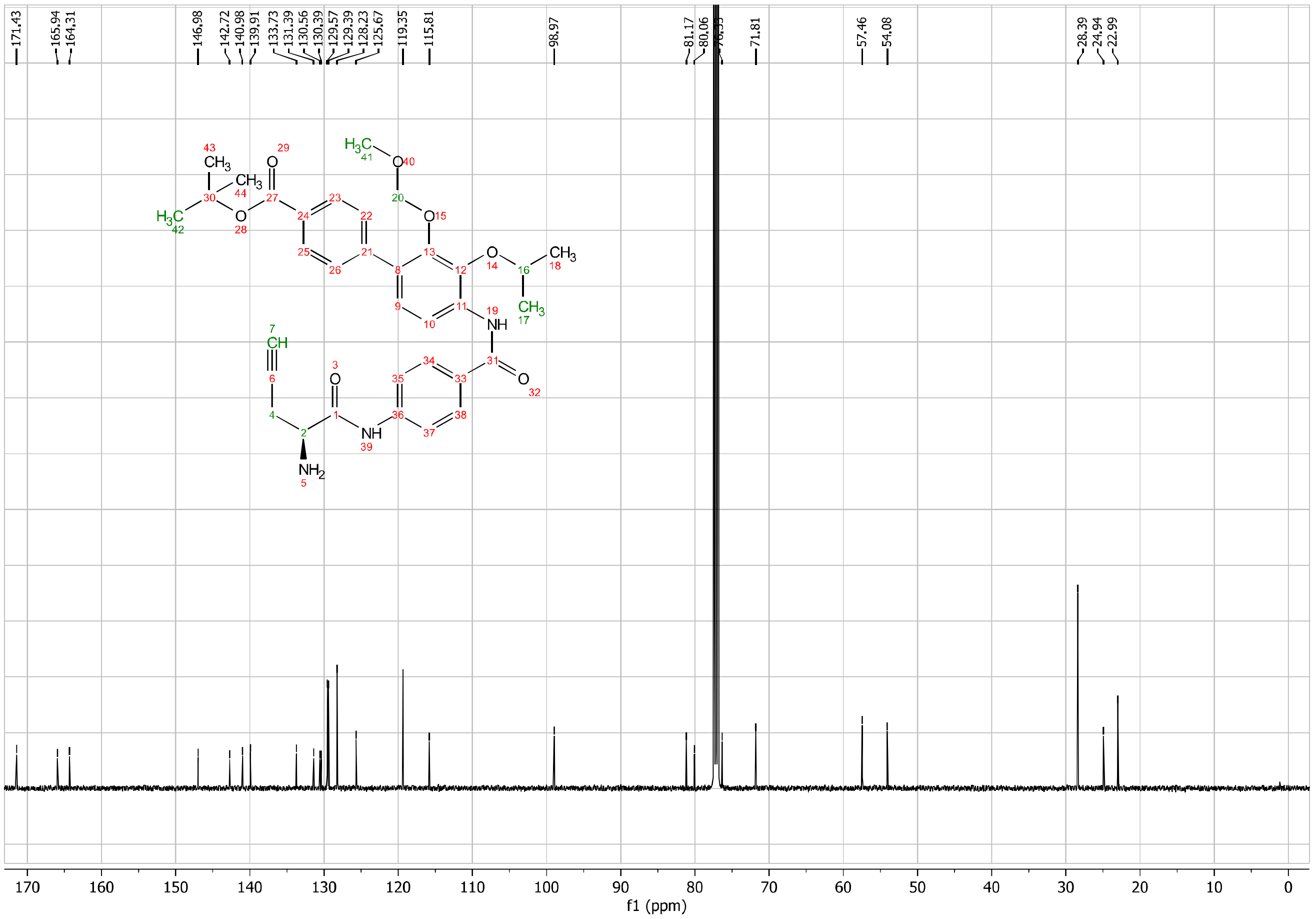
f1 (ppm)
171.43
165.94
164.31
146.98
142.72
140.98
139.91
133.73
131.39
130.56
130.39
129.57
129.39
128.23
125.67
119.35
115.81
98.97
81.17
80.06
71.81
57.46
54.08
28.39
24.94
22.99

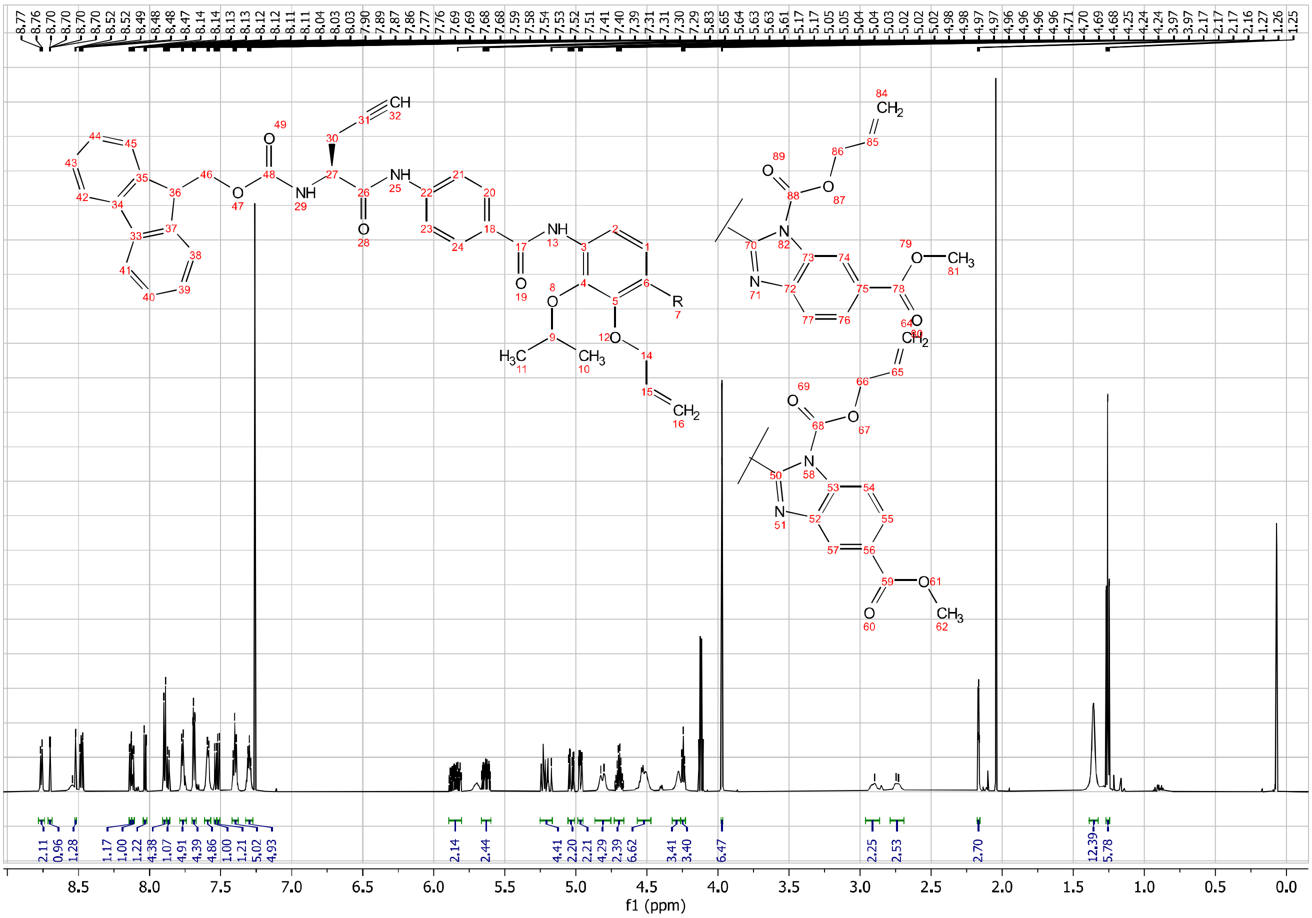
f1 (ppm)

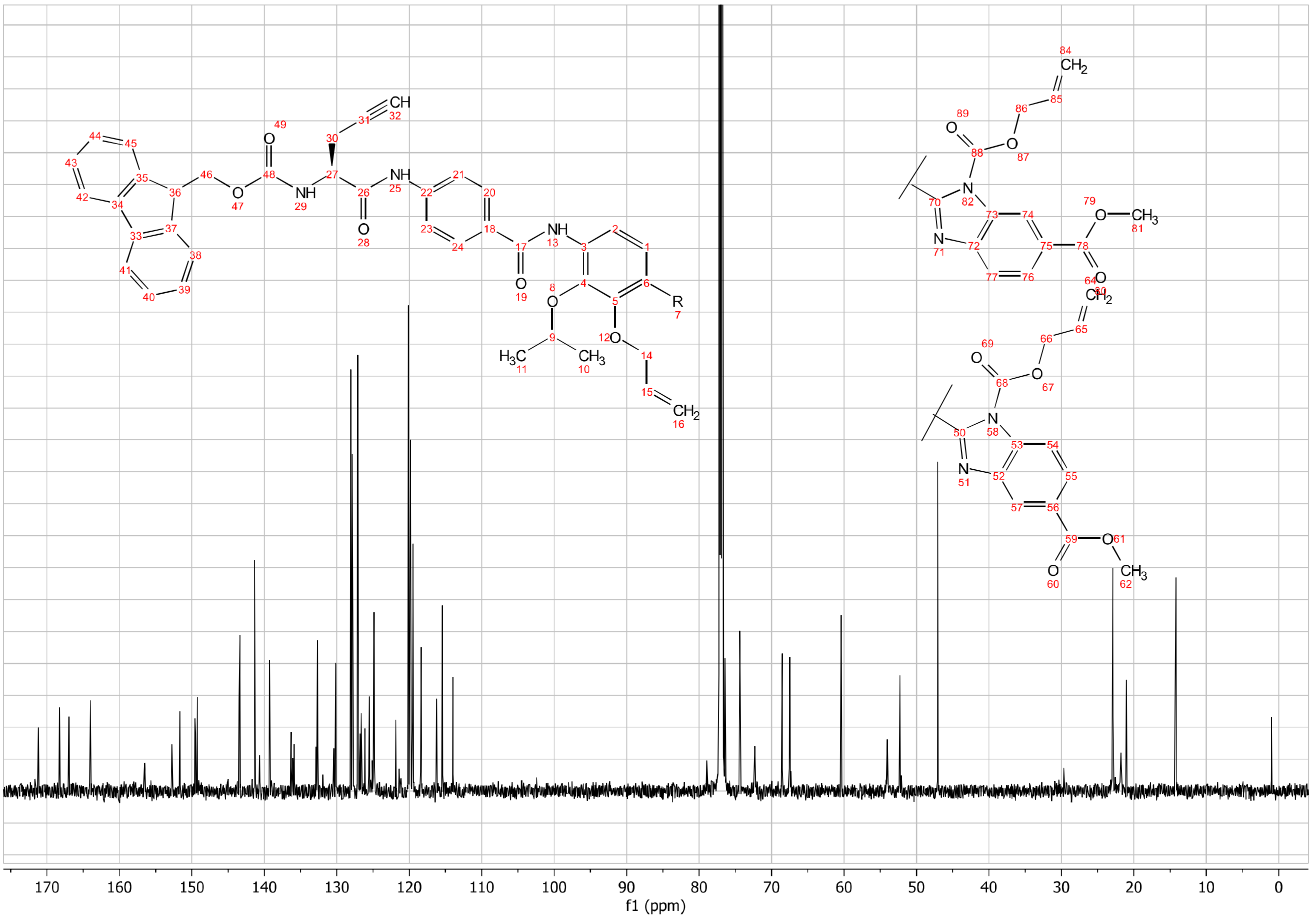
f1 (ppm)

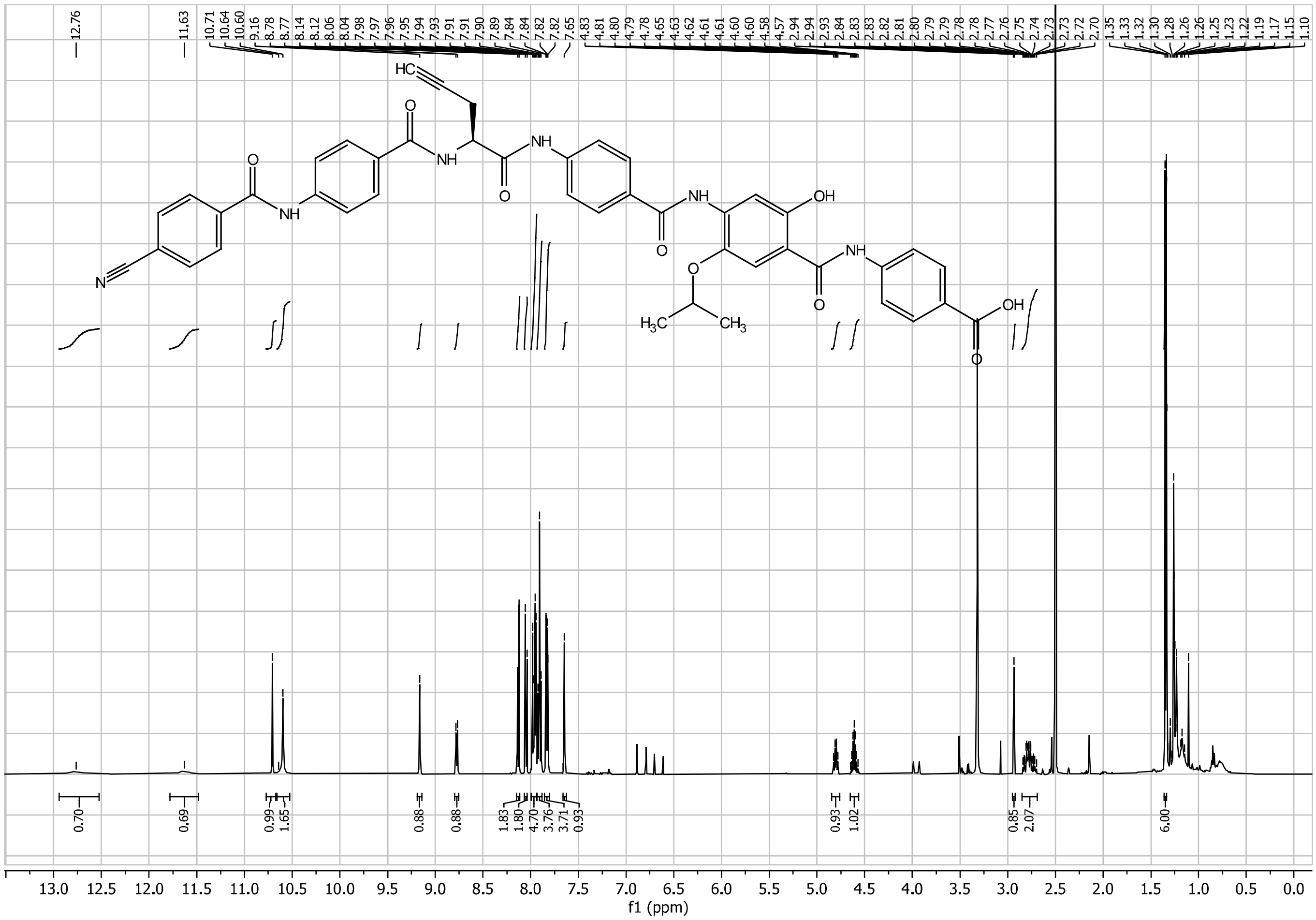

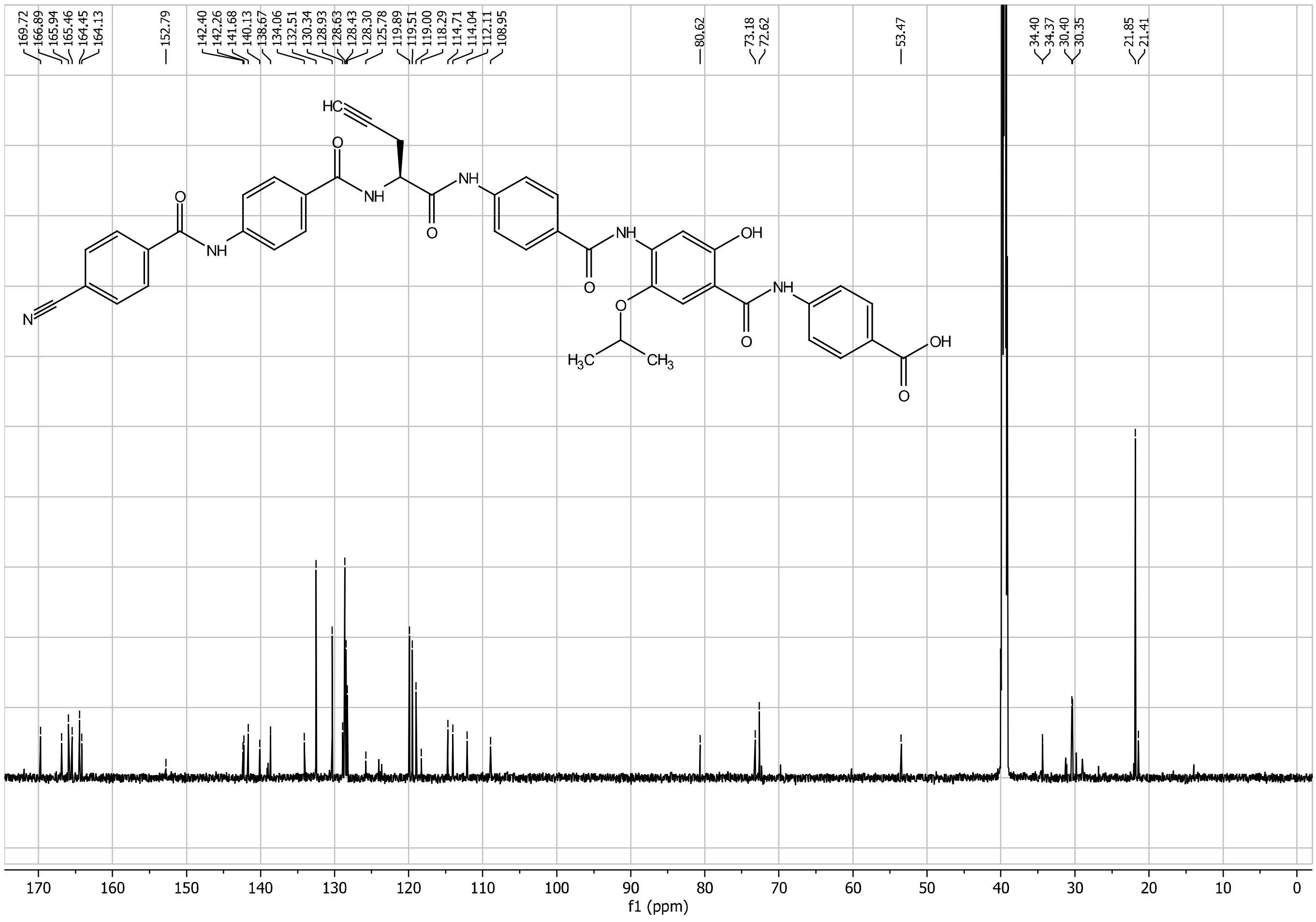

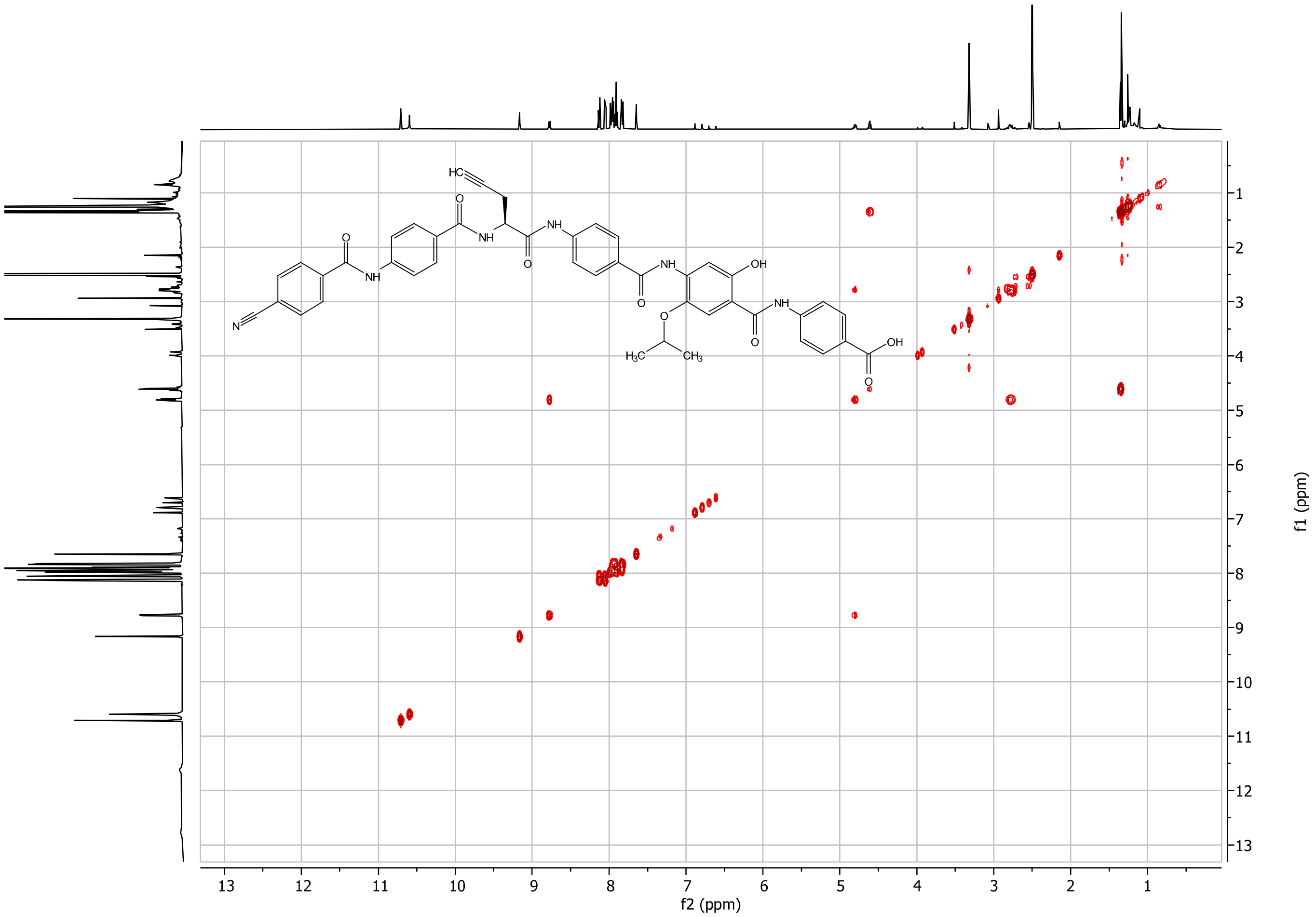
HC
NH
O
N
OH
H3C
CH3
f2 (ppm)
f1 (ppm)

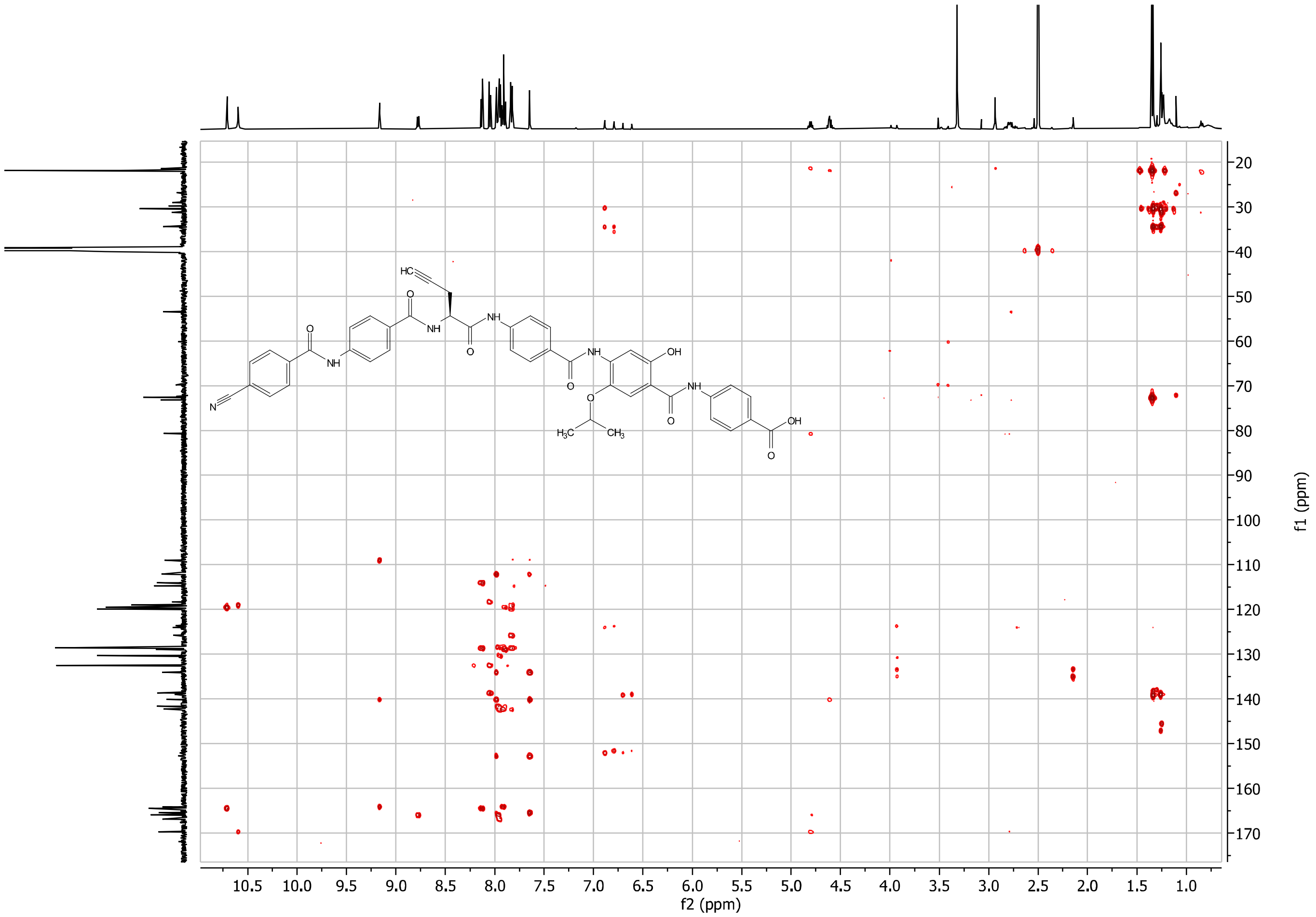
HC
NH
O
N
OH
H3C
CH3
f2 (ppm)
f1 (ppm)

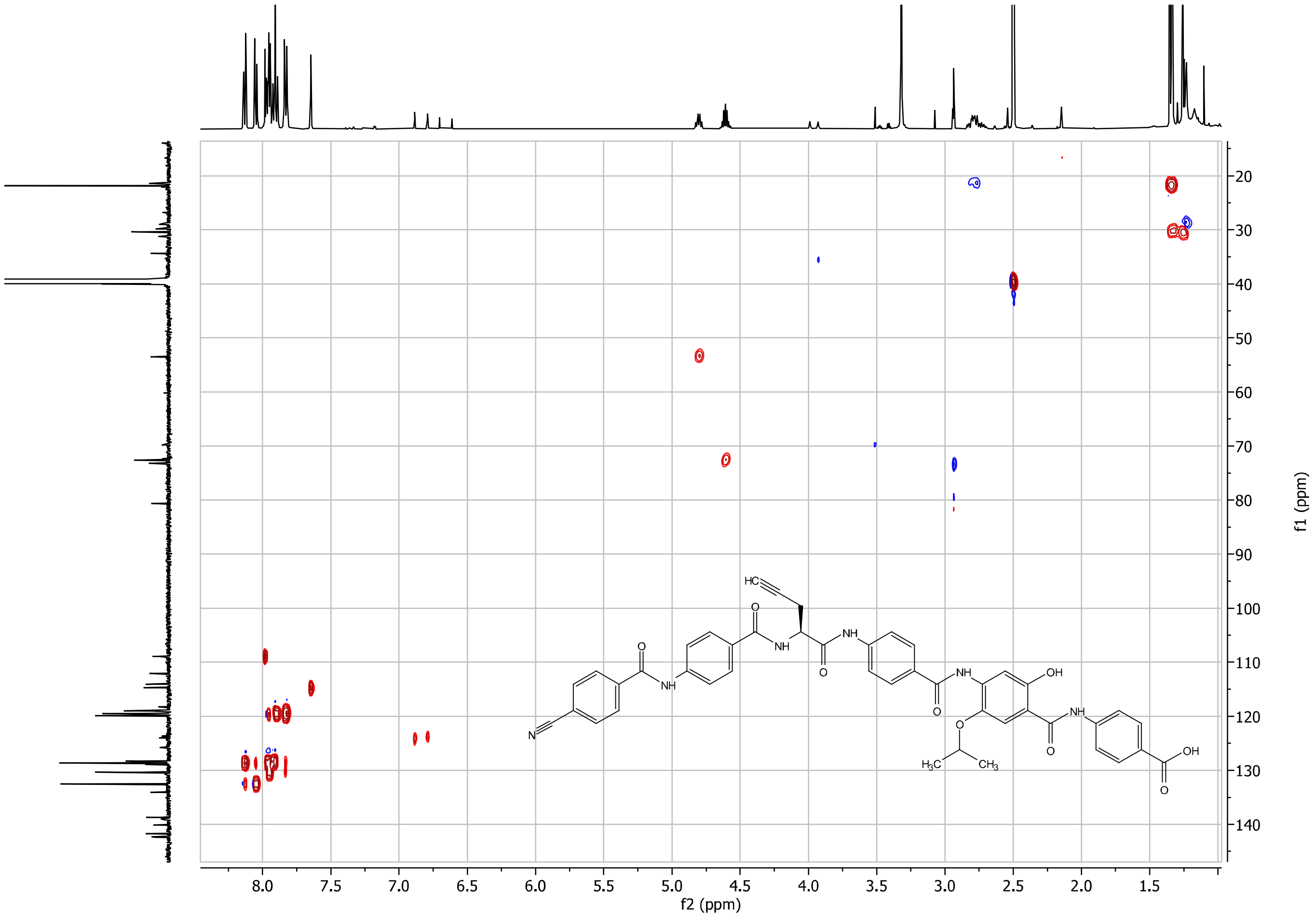

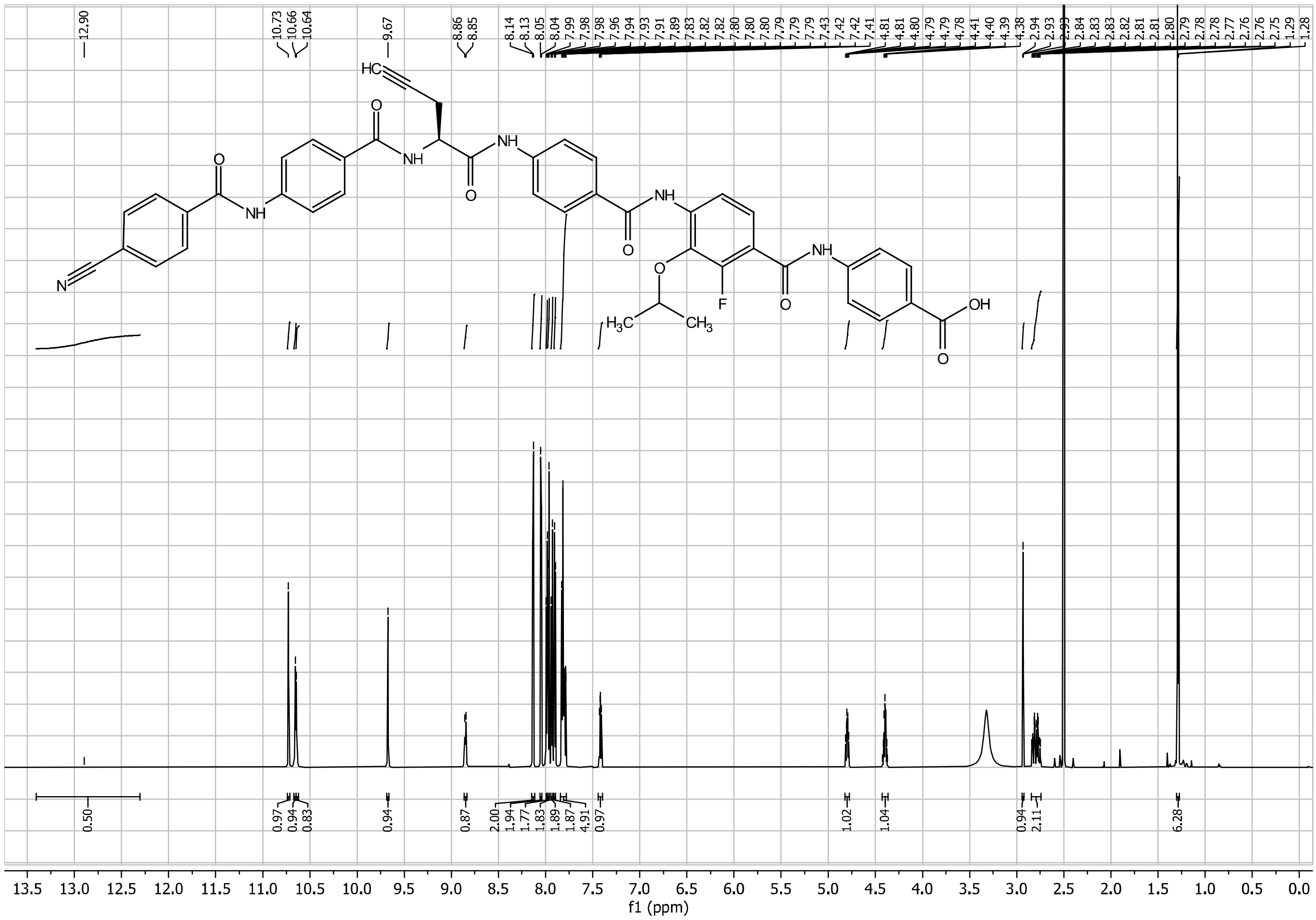

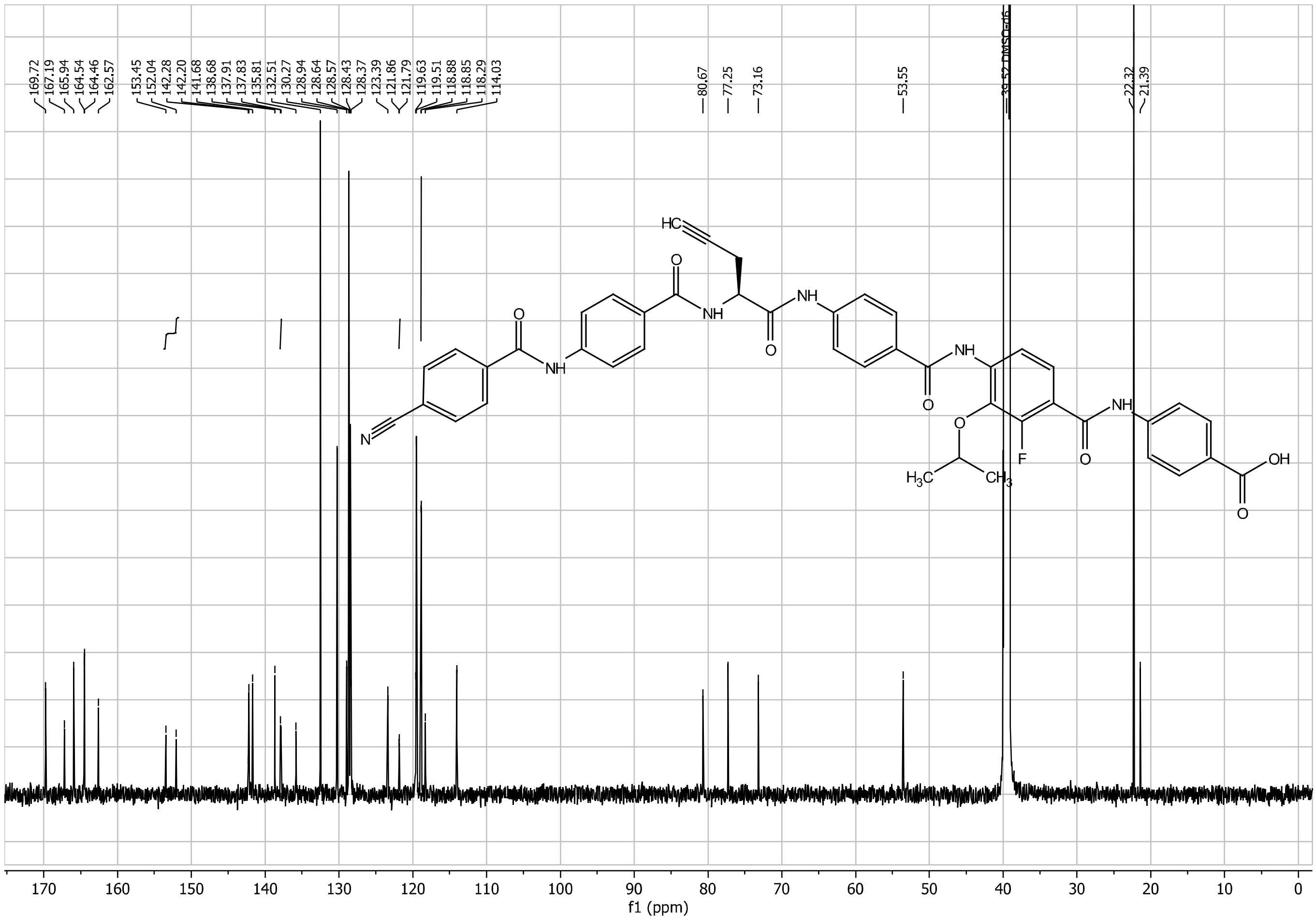
169.72
167.19
165.94
164.54
164.46
162.57
153.45
152.04
142.28
142.20
141.68
138.68
137.91
137.83
135.81
132.51
130.27
128.94
128.64
128.57
128.43
128.37
123.39
121.86
121.79
119.63
119.51
118.88
118.85
118.29
114.03
80.67
77.25
73.16
53.55
DMSO-d6
22.32
21.39
HC
O
NH
NH
O
O
NH
O
NH
NH
N
O
O
NH
H3C
CH3
F
O
OH
O
f1 (ppm)

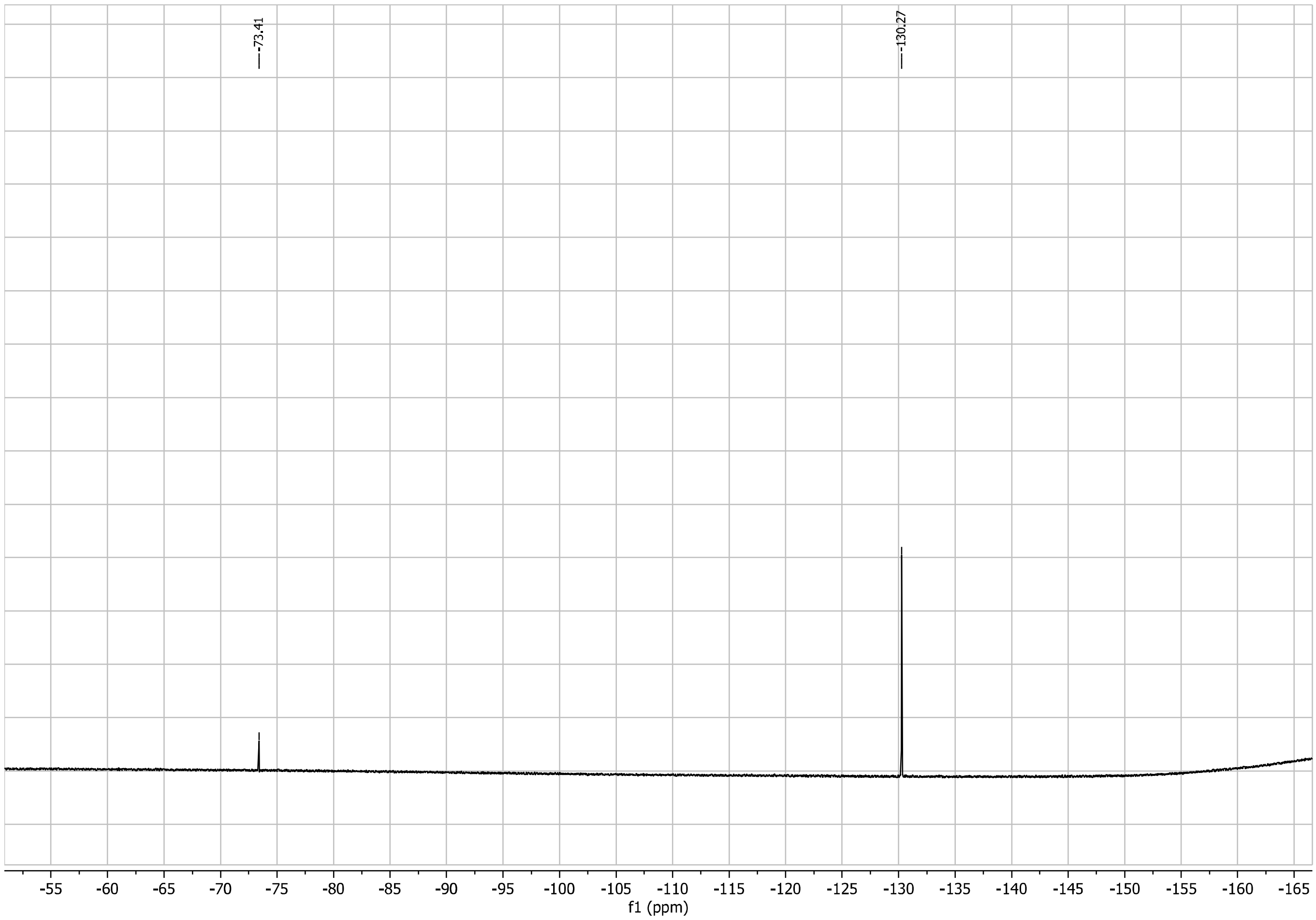
-73.41
-130.27
f1 (ppm)

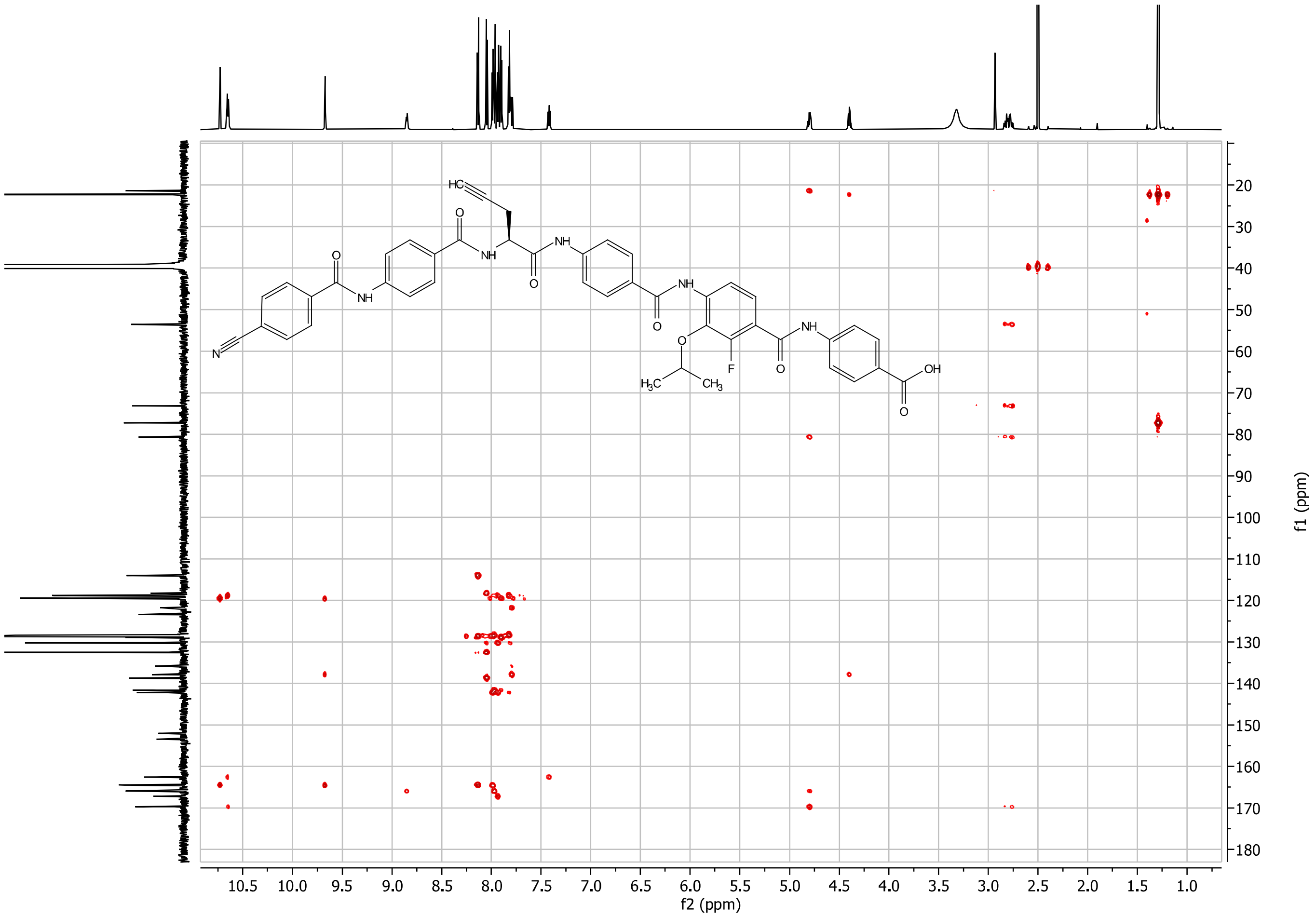
f2 (ppm)
f1 (ppm)
HC
NH
O
OH
N
F
H3C
CH3

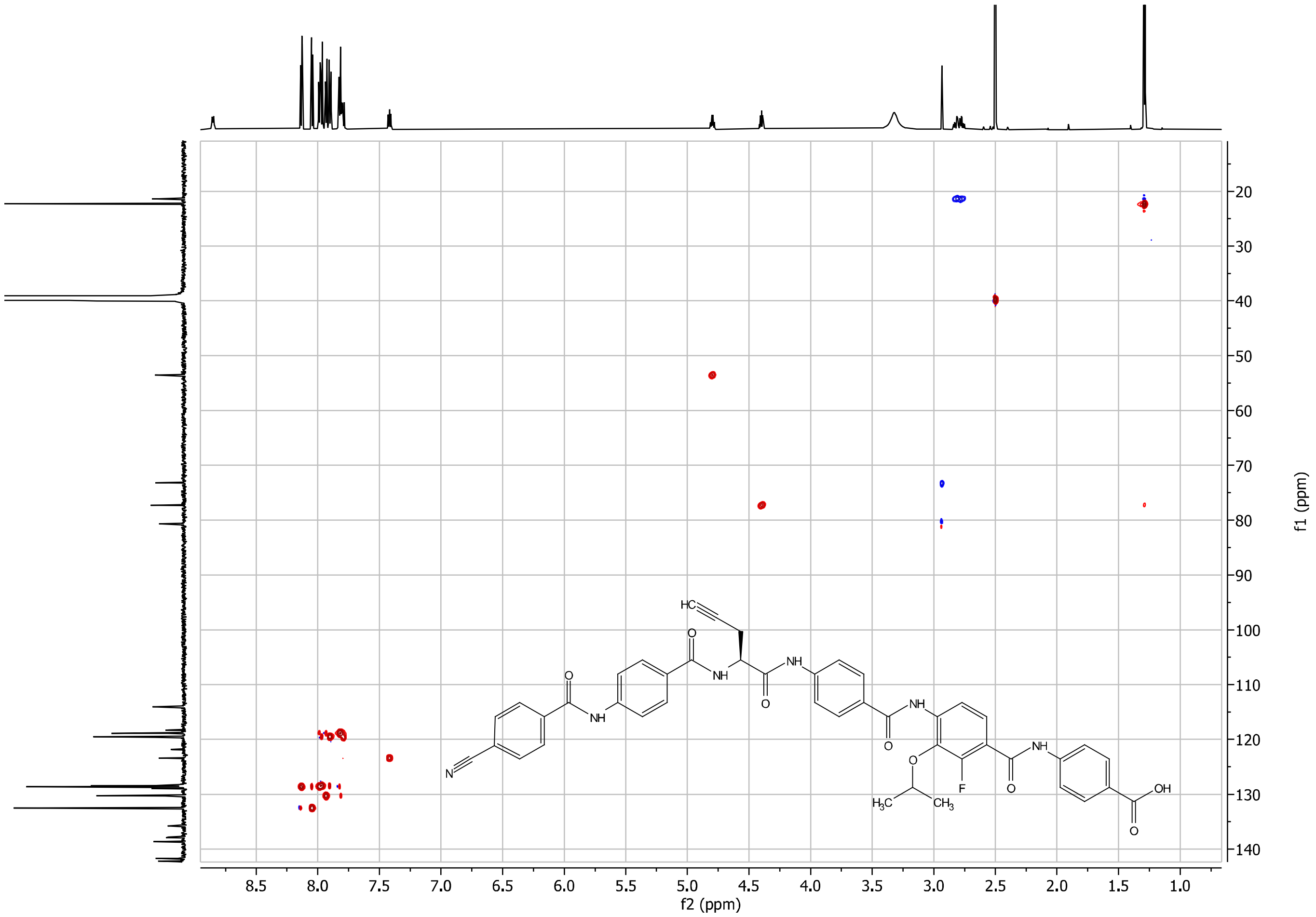
f2 (ppm)
f1 (ppm)
HC
NH
O
OH
N
F
H3C
CH3

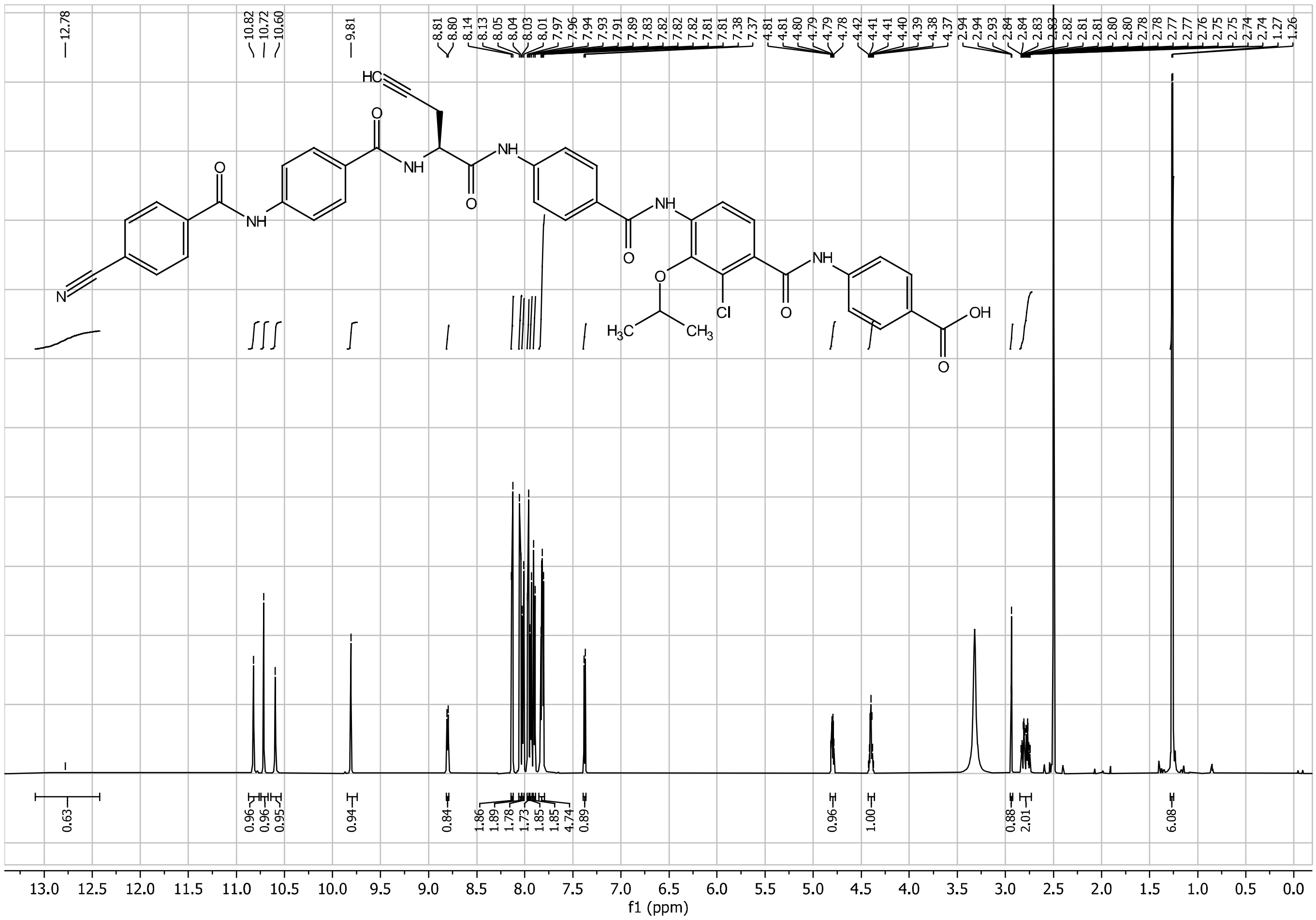

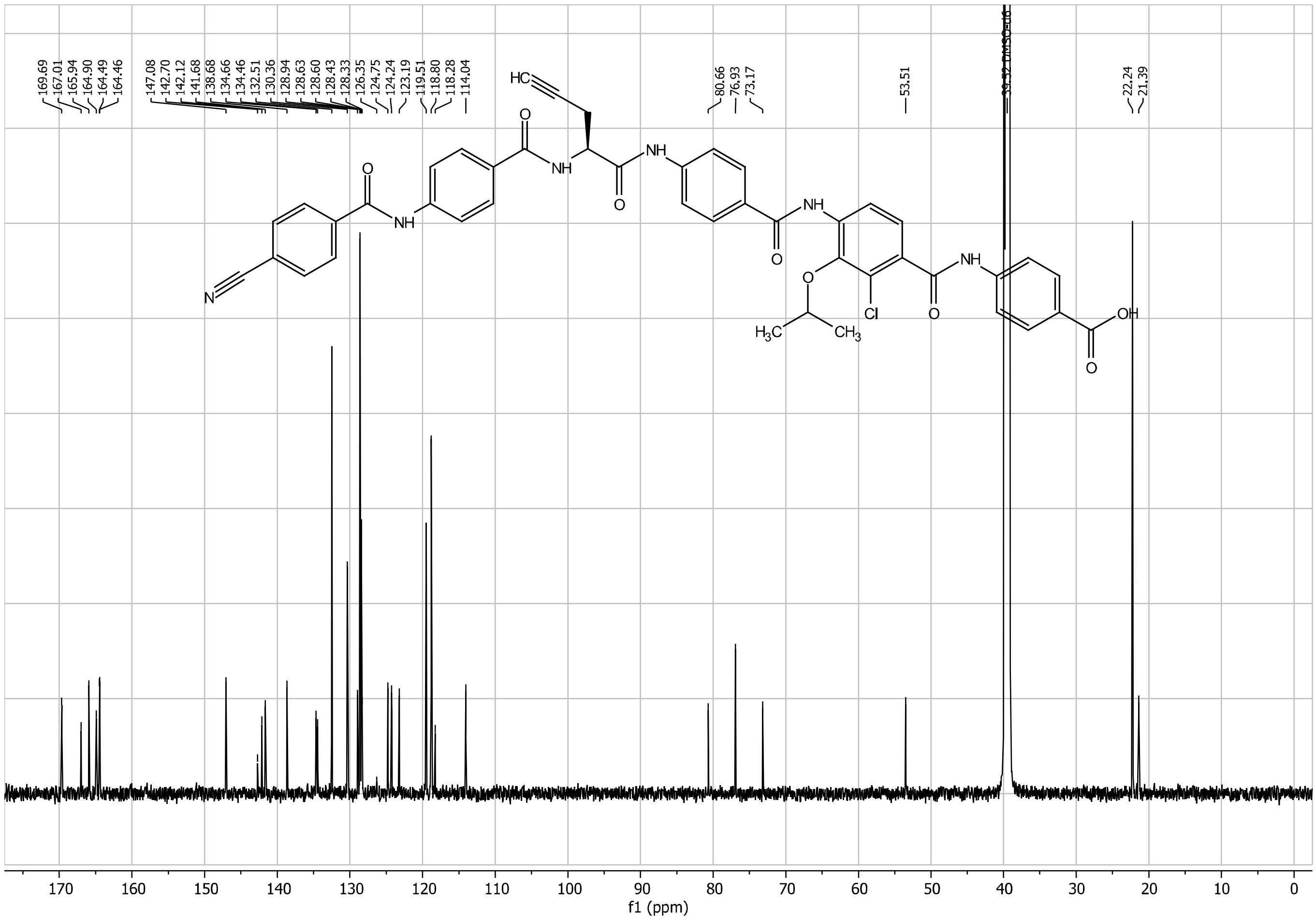

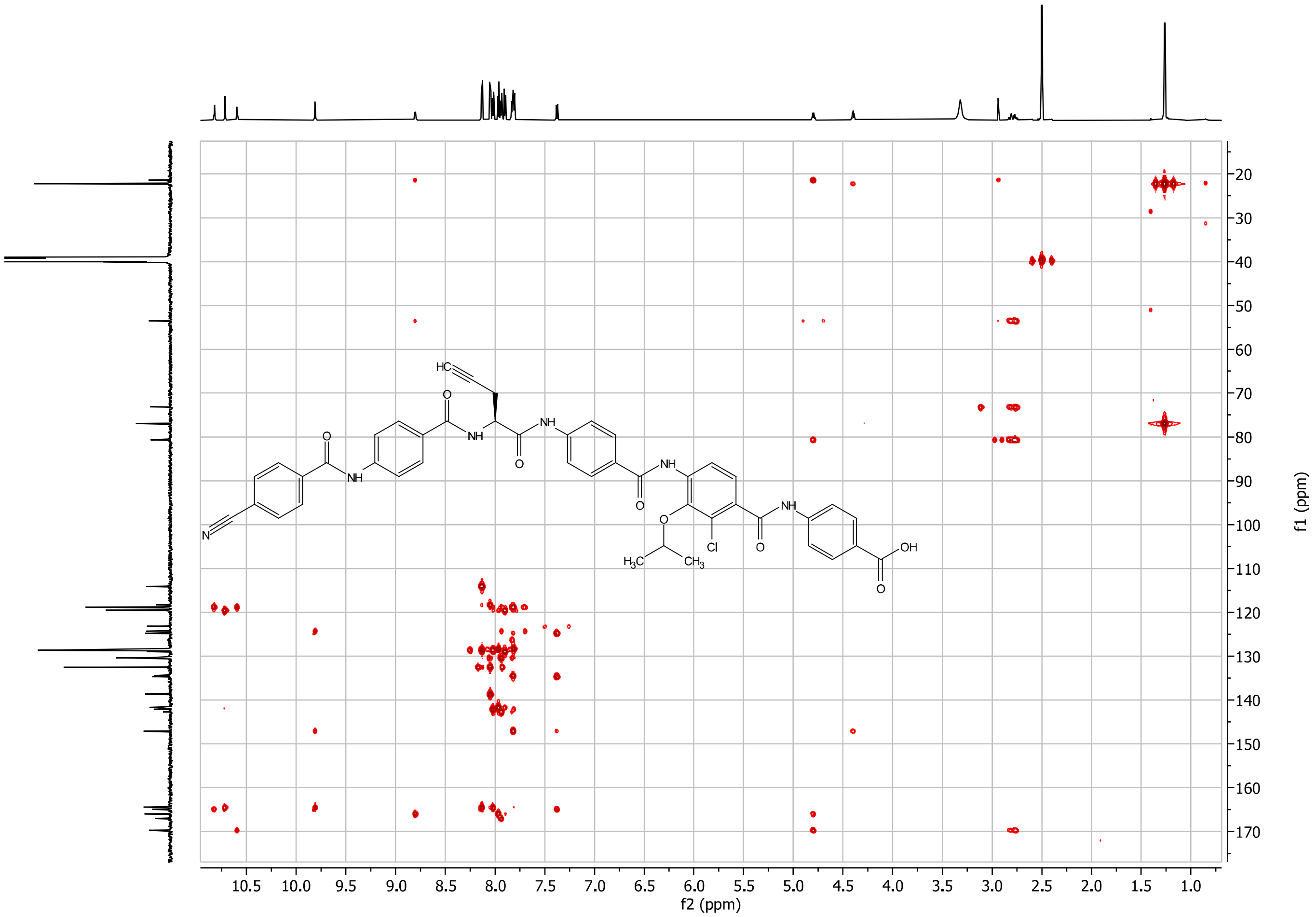
HC
O
NH
NH
O
NH
N
O
NH
O
O
NH
Cl
O
OH
O
H3C
CH3
f1 (ppm)
f2 (ppm)
10.5 10.0 9.5 9.0 8.5 8.0 7.5 7.0 6.5 6.0 5.5 5.0 4.5 4.0 3.5 3.0 2.5 2.0 1.5 1.0
20 30 40 50 60 70 80 90 100 110 120 130 140 150 160 170

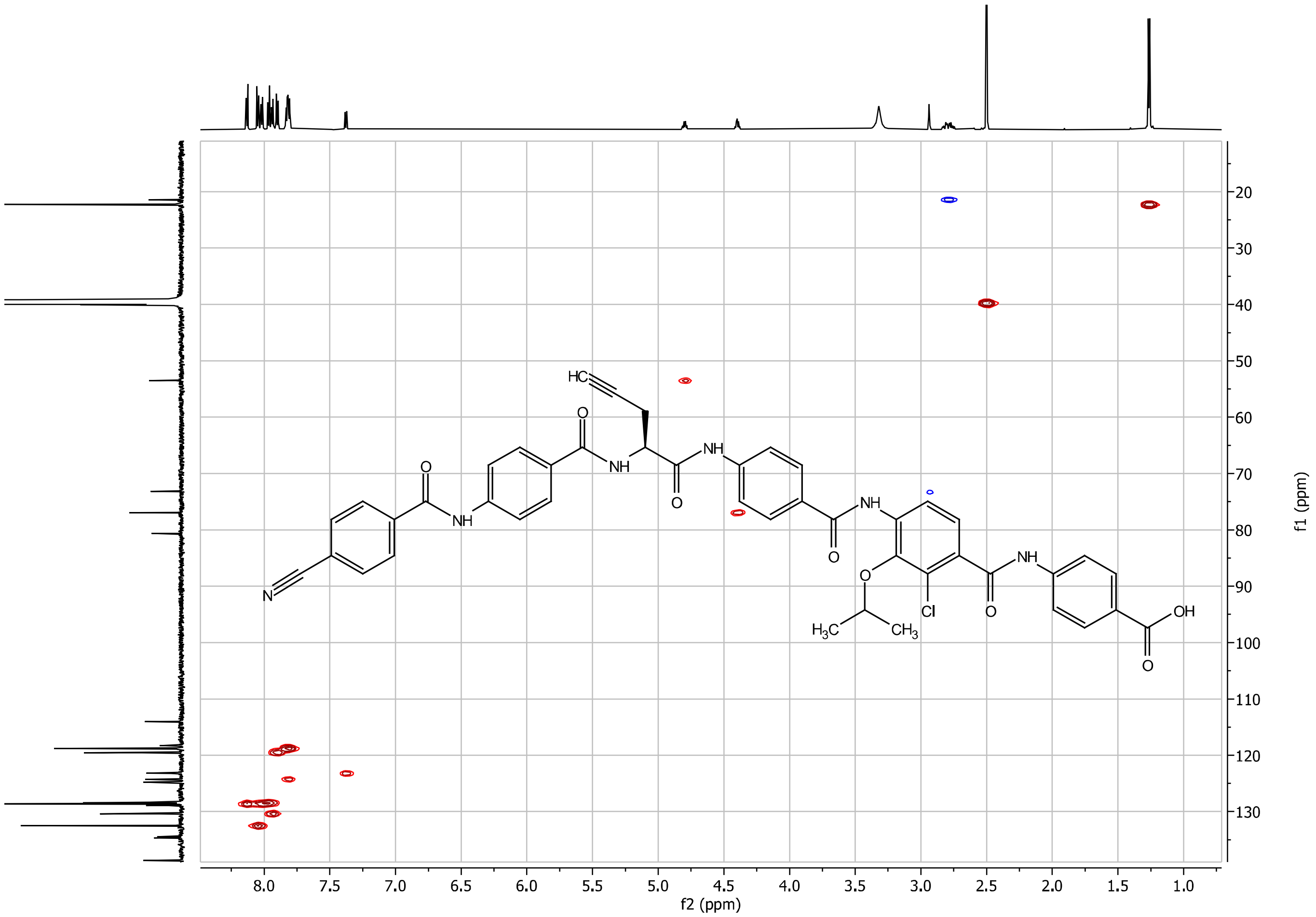
HC
O
O
NH
NH
O
NH
N
NH
O
O
NH
Cl
O
OH
O
H3C
CH3
f1 (ppm)
f2 (ppm)
8.0 7.5 7.0 6.5 6.0 5.5 5.0 4.5 4.0 3.5 3.0 2.5 2.0 1.5 1.0
20 30 40 50 60 70 80 90 100 110 120 130

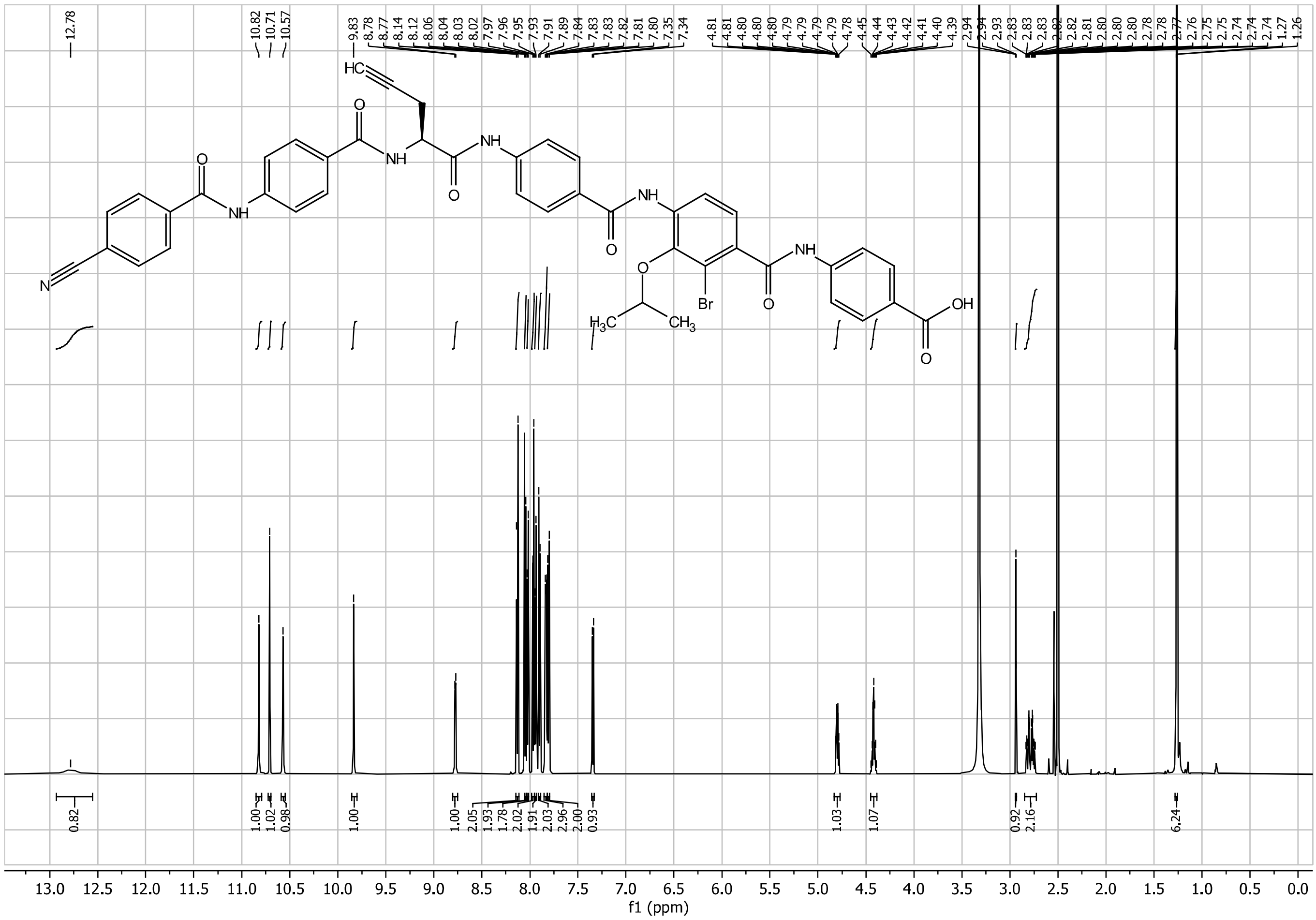

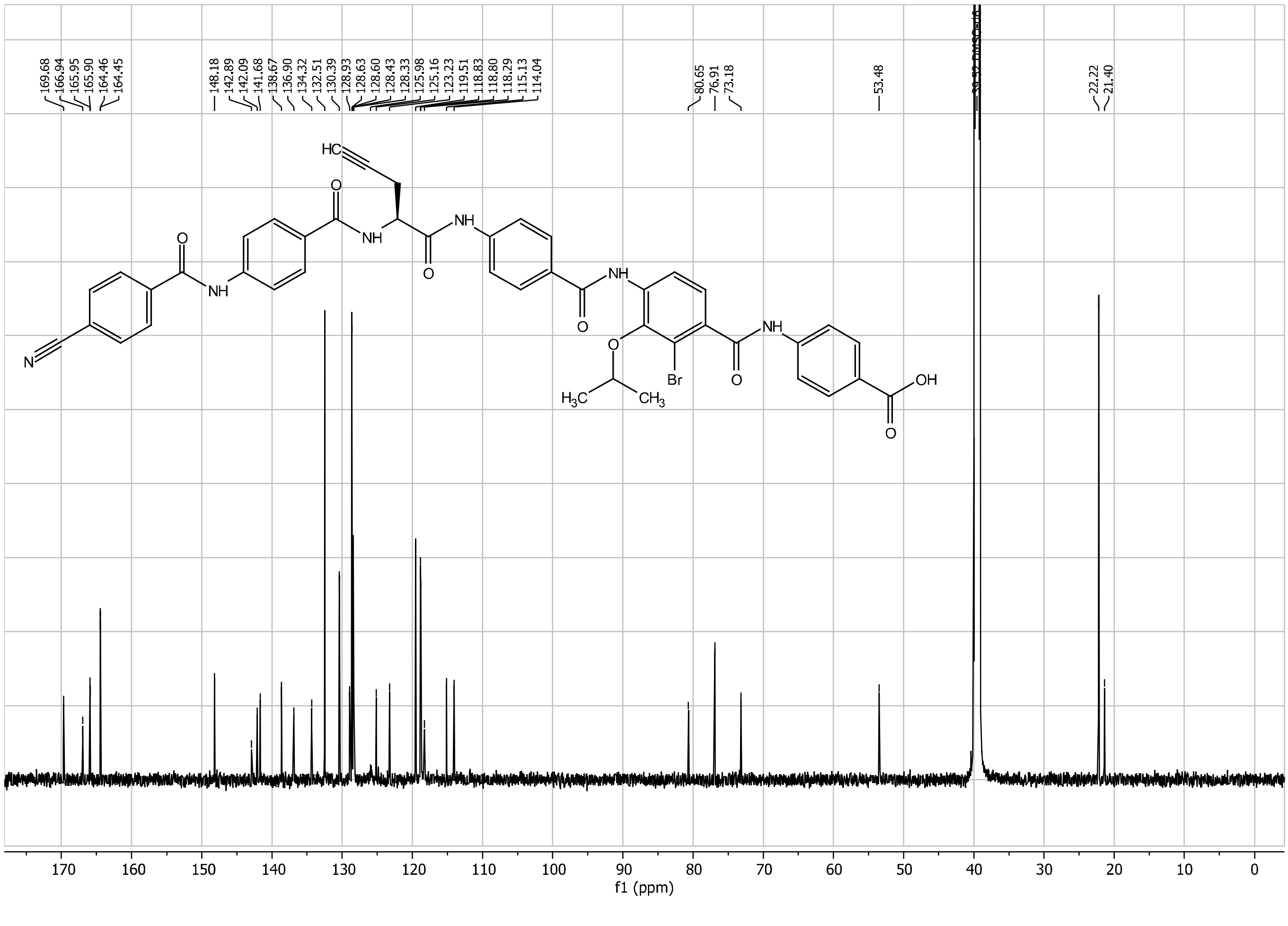

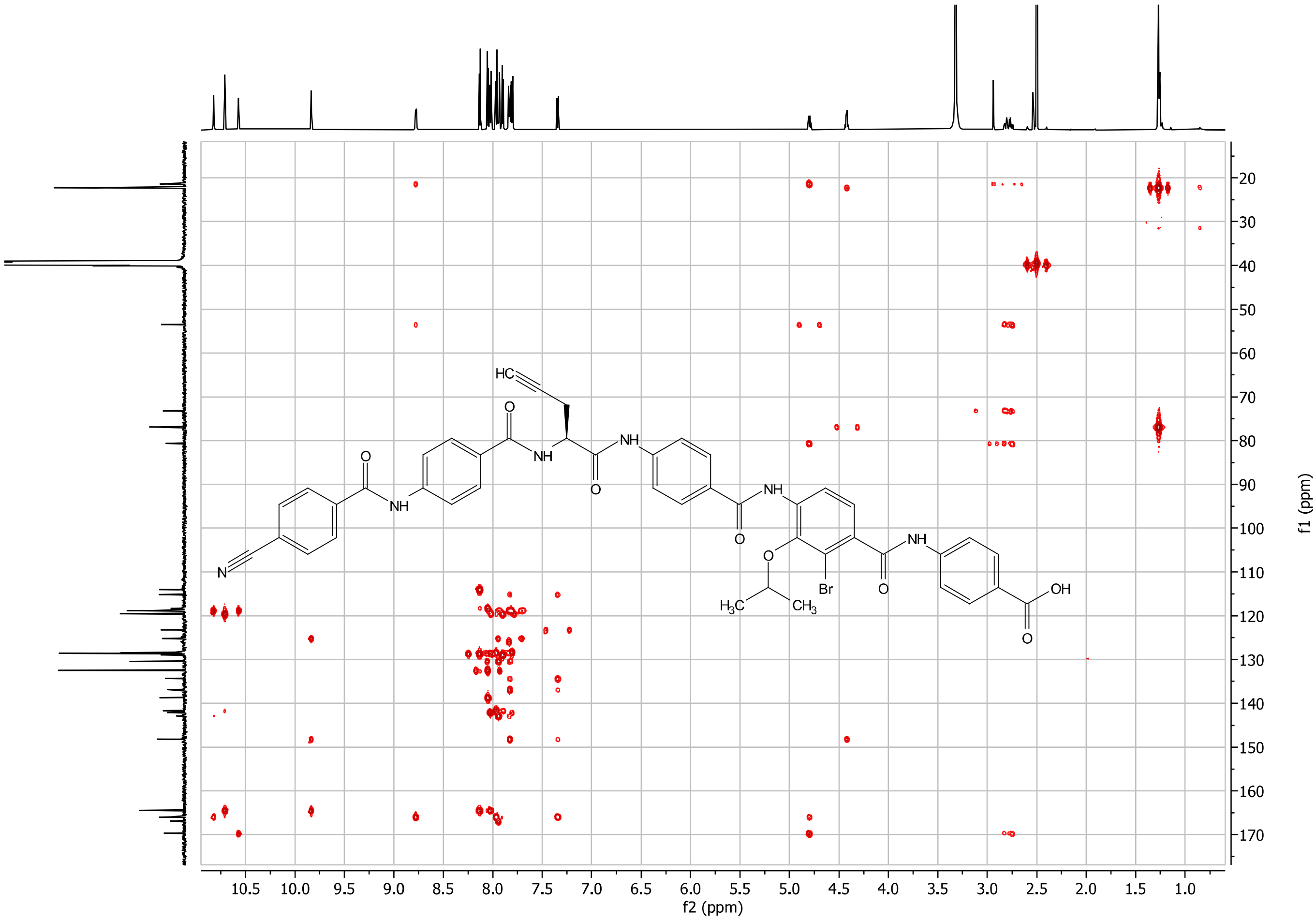
HC
O
NH
O
NH
O
NH
N
NH
O
O
Br
NH
O
OH
O
H3C
CH3
f2 (ppm)
f1 (ppm)

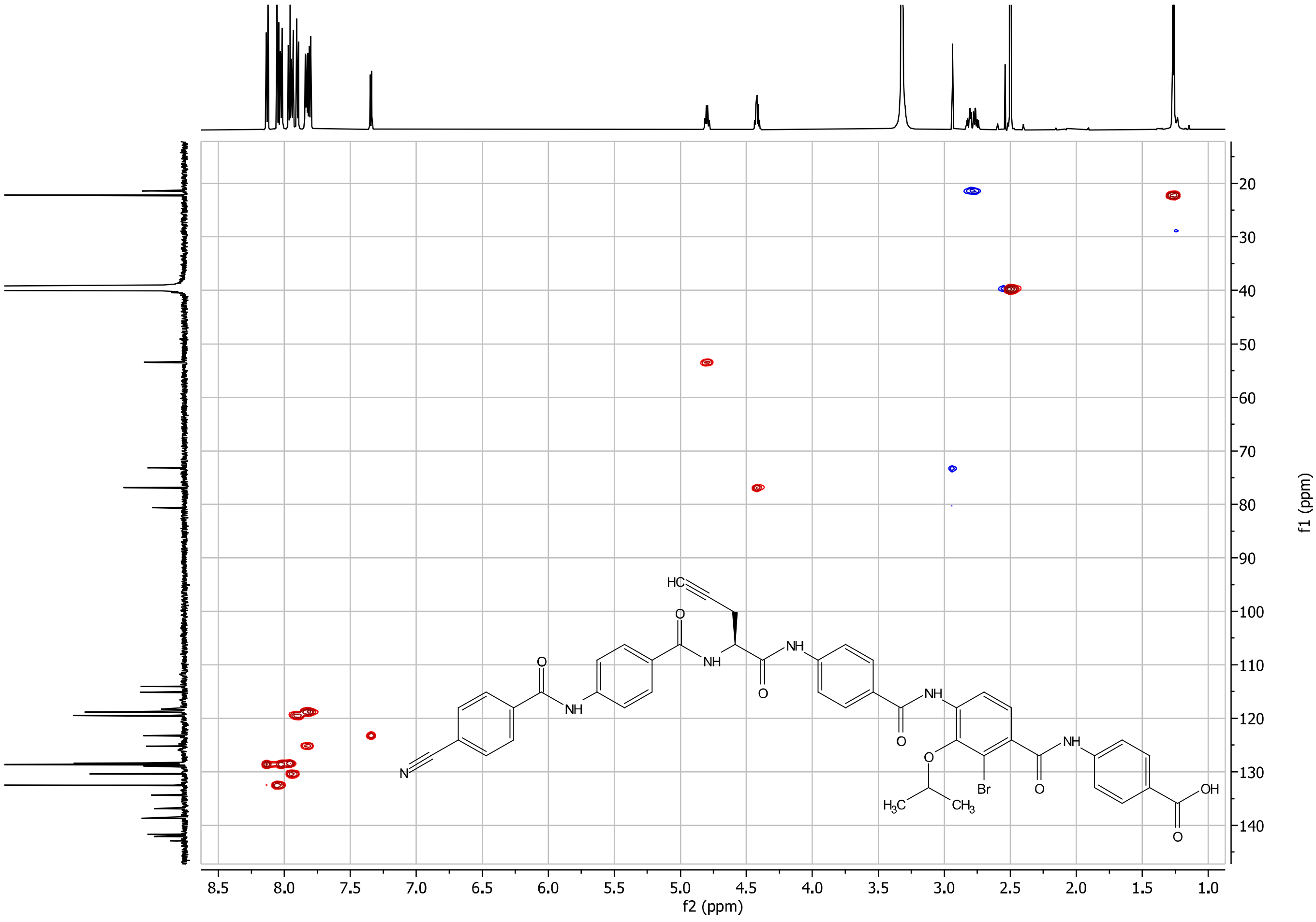
HC
O
NH
O
NH
O
NH
N
NH
O
O
Br
NH
O
OH
O
H3C
CH3
f2 (ppm)
f1 (ppm)

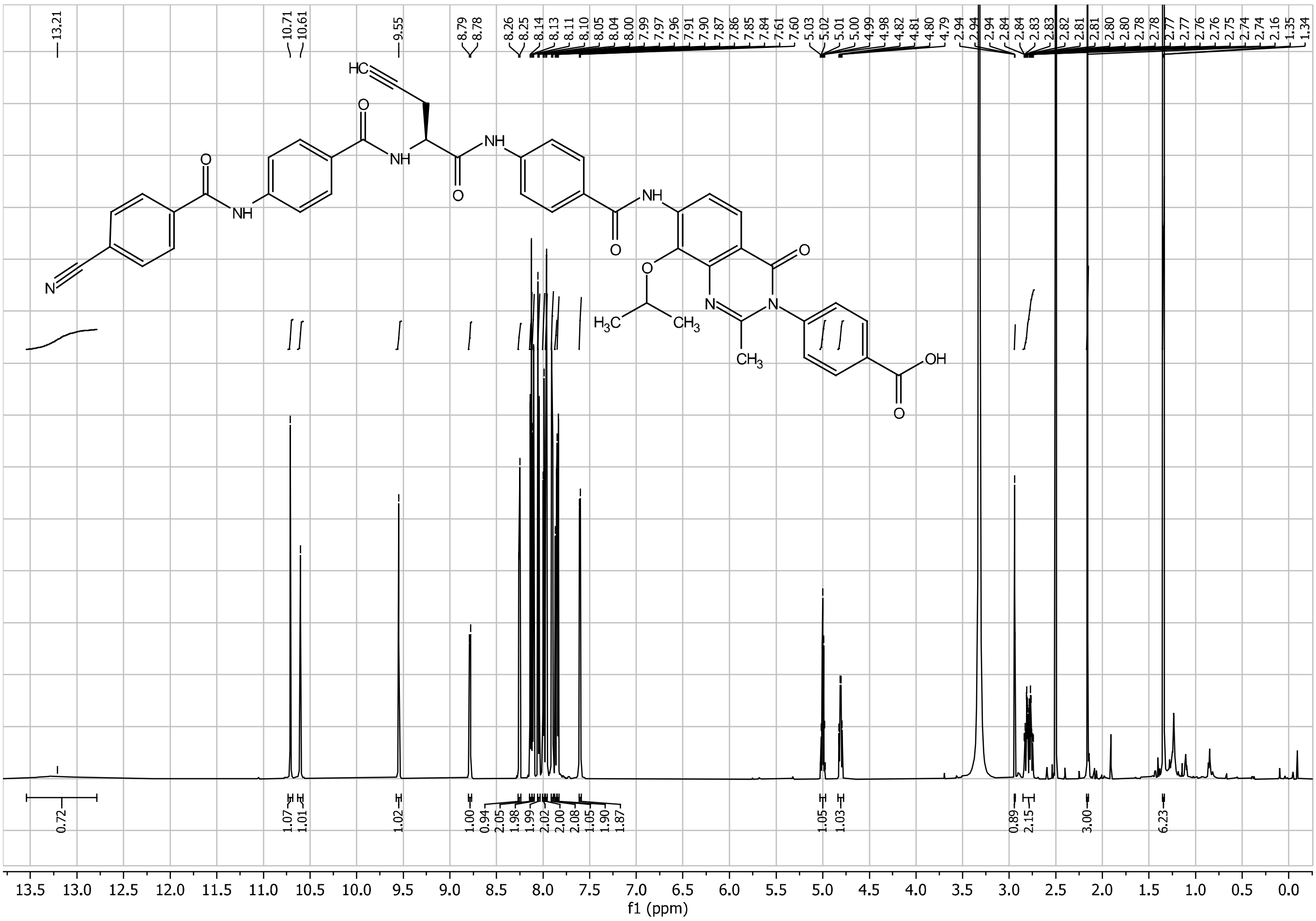
13.21
10.71 10.61
9.55
8.79 8.78
8.26 8.25 8.14 8.13 8.11 8.10 8.05 8.04 8.00 7.99 7.97 7.96 7.91 7.90 7.87 7.86 7.85 7.84 7.61 7.60
5.03 5.02 5.01 5.00 4.99 4.98 4.82 4.81 4.80 4.79
2.94 2.94 2.94 2.84 2.84 2.83 2.83 2.82 2.81 2.81 2.80 2.80 2.78 2.78 2.77 2.77 2.76 2.76 2.75 2.74 2.74 2.16 1.35 1.34
0.72
1.07 1.01
1.02
1.00 0.94 2.05 1.98 1.99 2.02 2.00 2.08 1.05 1.90 1.87
1.05 1.03
0.89 2.15
3.00
6.23
13.5 13.0 12.5 12.0 11.5 11.0 10.5 10.0 9.5 9.0 8.5 8.0 7.5 7.0 6.5 6.0 5.5 5.0 4.5 4.0 3.5 3.0 2.5 2.0 1.5 1.0 0.5 0.0
f1 (ppm)
HC
O
NH
NH
O
N
H3C
CH3
CH3
OH

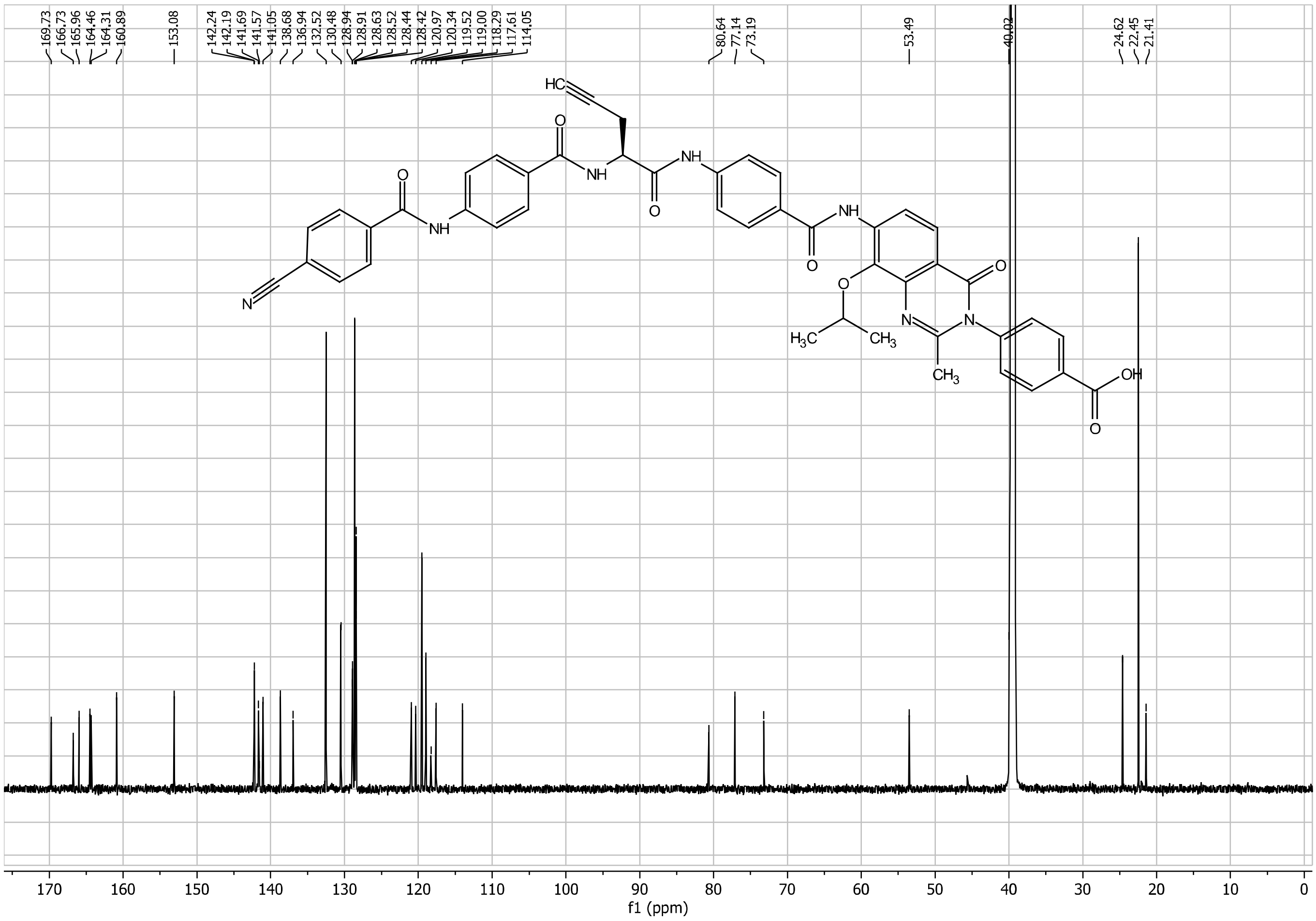
169.73 166.73 165.96 164.46 164.31 160.89
153.08
142.24 142.19 141.69 141.57 141.05 138.68 136.94 132.52 130.48 128.94 128.91 128.63 128.52 128.44 128.42 120.97 120.34 119.52 119.00 118.29 117.61 114.05
80.64 77.14 73.19
53.49
40.02
24.62 22.45 21.41
170 160 150 140 130 120 110 100 90 80 70 60 50 40 30 20 10 0
f1 (ppm)
HC
O
NH
NH
O
N
H3C
CH3
CH3
OH

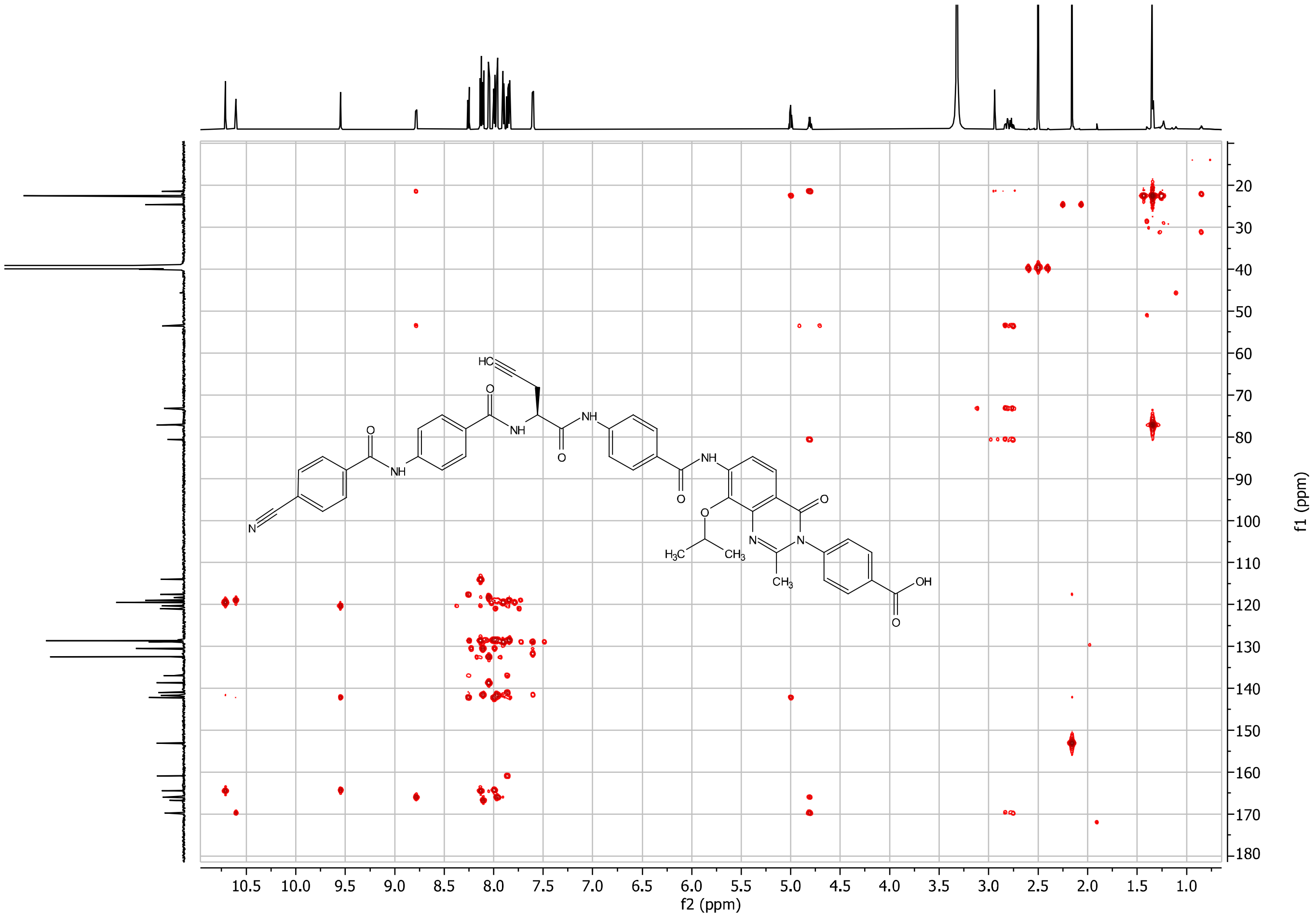
f1 (ppm)
f2 (ppm)

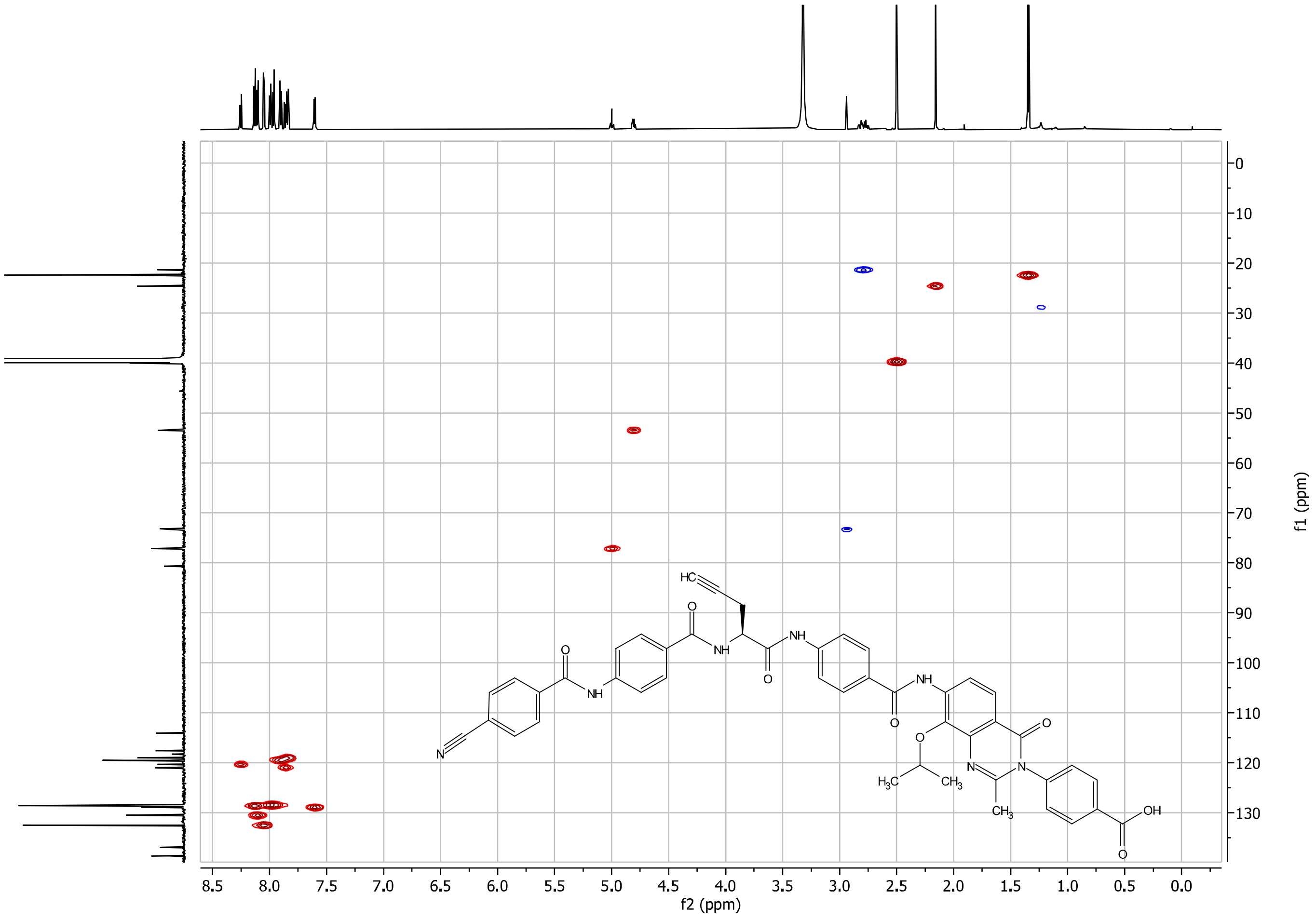
f1 (ppm)
f2 (ppm)

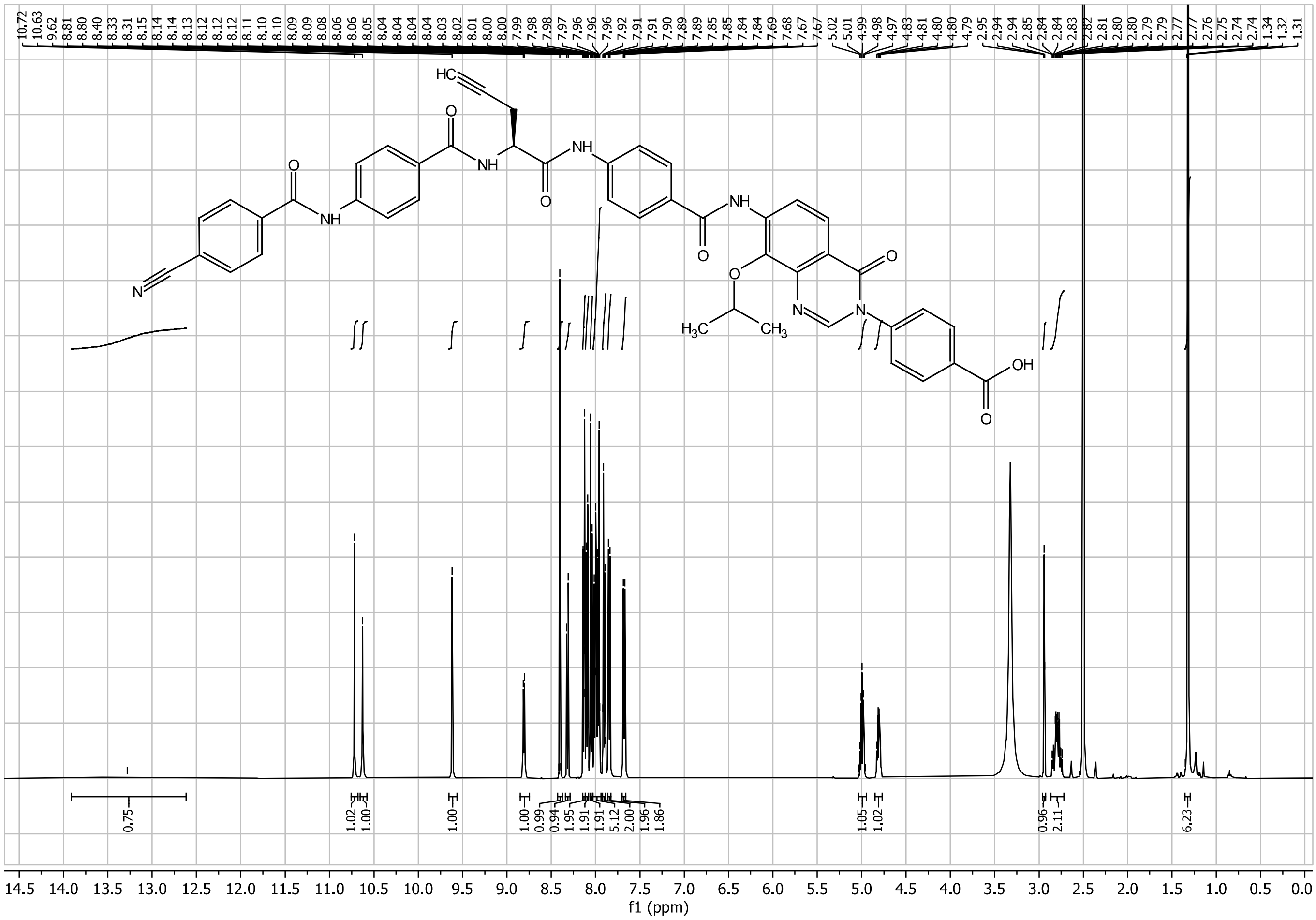
10.72
10.63
9.62
8.81
8.80
8.40
8.33
8.31
8.15
8.14
8.14
8.13
8.12
8.12
8.12
8.11
8.10
8.10
8.09
8.09
8.08
8.06
8.06
8.05
8.04
8.04
8.04
8.04
8.03
8.02
8.01
8.00
8.00
7.99
7.98
7.98
7.97
7.96
7.96
7.96
7.92
7.91
7.91
7.90
7.89
7.89
7.85
7.85
7.84
7.84
7.69
7.68
7.67
7.67
5.02
5.01
4.99
4.98
4.97
4.83
4.81
4.80
4.80
4.79
2.95
2.94
2.94
2.85
2.84
2.84
2.83
2.82
2.81
2.80
2.80
2.79
2.79
2.77
2.77
2.76
2.75
2.74
2.74
1.34
1.32
1.31
HC
O
NH
NH
O
O
NH
O
NH
O
NH
N
O
O
N
N
H3C
CH3
OH
O
0.75
1.02
1.00
1.00
1.00
0.99
0.94
1.95
1.91
1.91
5.12
2.00
1.96
1.86
1.05
1.02
0.96
2.11
6.23
14.5 14.0 13.5 13.0 12.5 12.0 11.5 11.0 10.5 10.0 9.5 9.0 8.5 8.0 7.5 7.0 6.5 6.0 5.5 5.0 4.5 4.0 3.5 3.0 2.5 2.0 1.5 1.0 0.5 0.0
f1 (ppm)

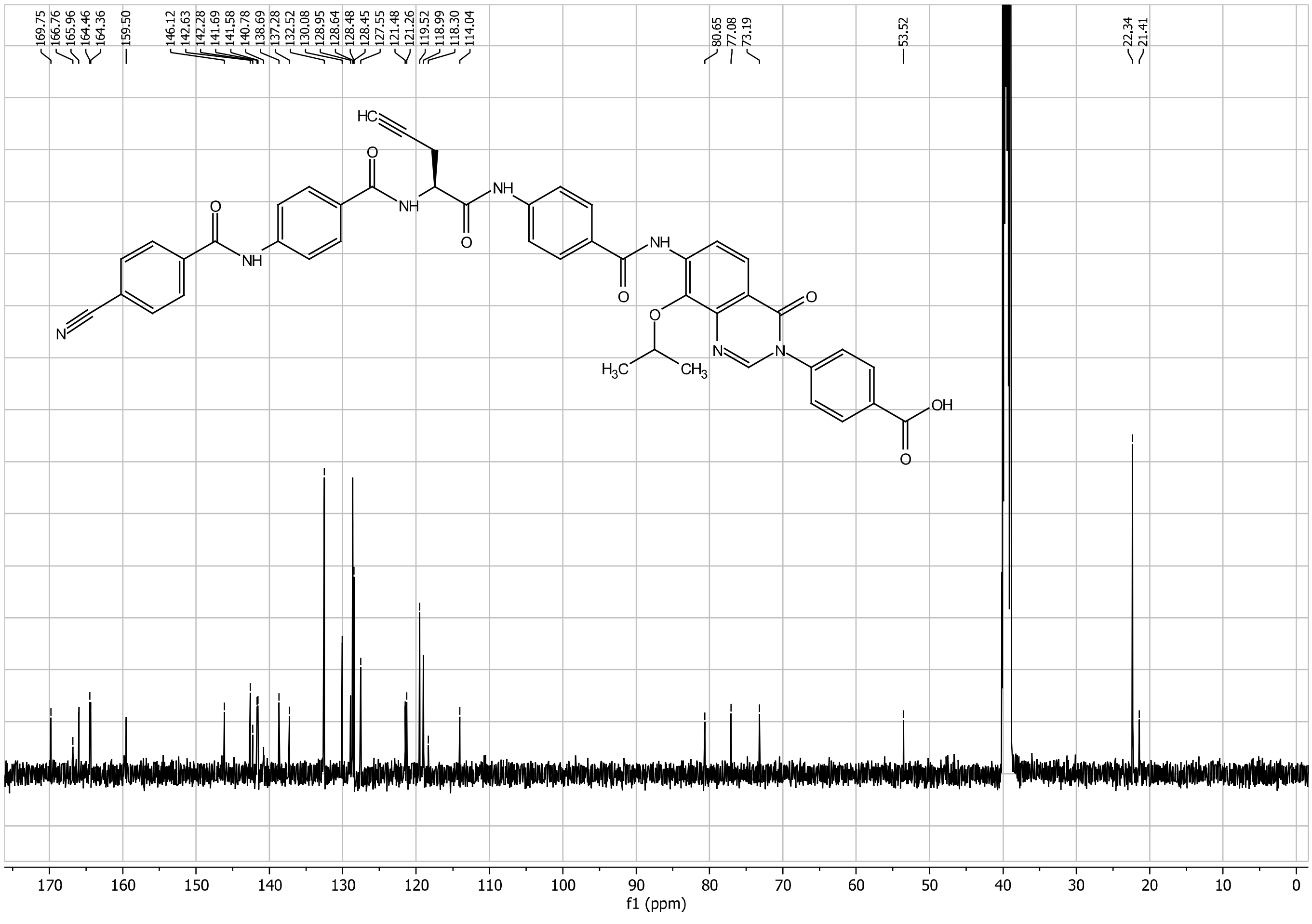
169.75
166.76
165.96
164.46
164.36
159.50
146.12
142.63
142.28
141.69
141.58
140.78
138.69
137.28
132.52
130.08
128.95
128.64
128.48
128.45
127.55
121.48
121.26
119.52
118.99
118.30
114.04
80.65
77.08
73.19
53.52
22.34
21.41
HC
O
NH
NH
O
O
NH
O
NH
O
NH
N
O
O
N
N
H3C
CH3
OH
O
170 160 150 140 130 120 110 100 90 80 70 60 50 40 30 20 10 0
f1 (ppm)

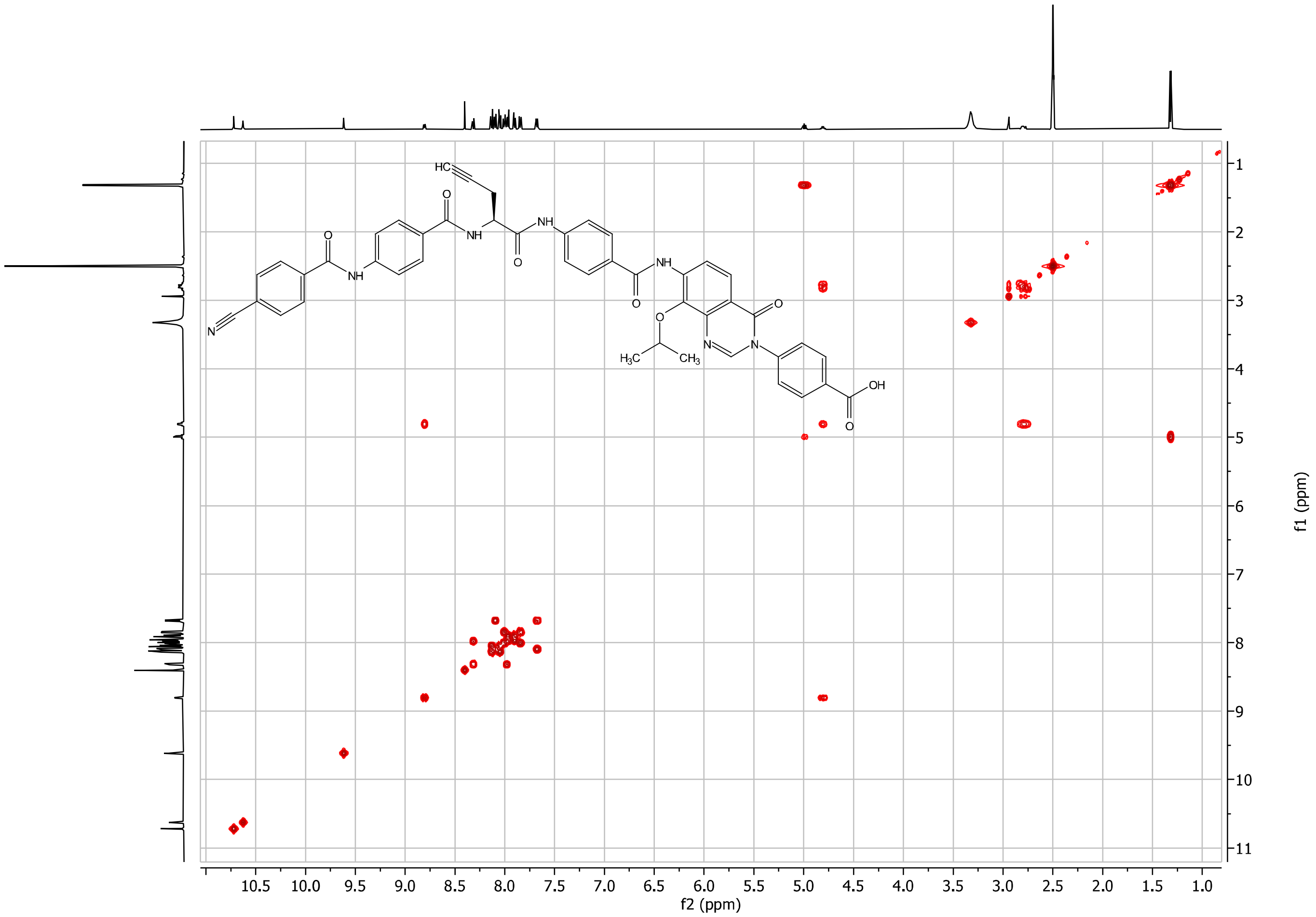

f1 (ppm)
f2 (ppm)
HC
NH
O
N
OH
H3C
CH3

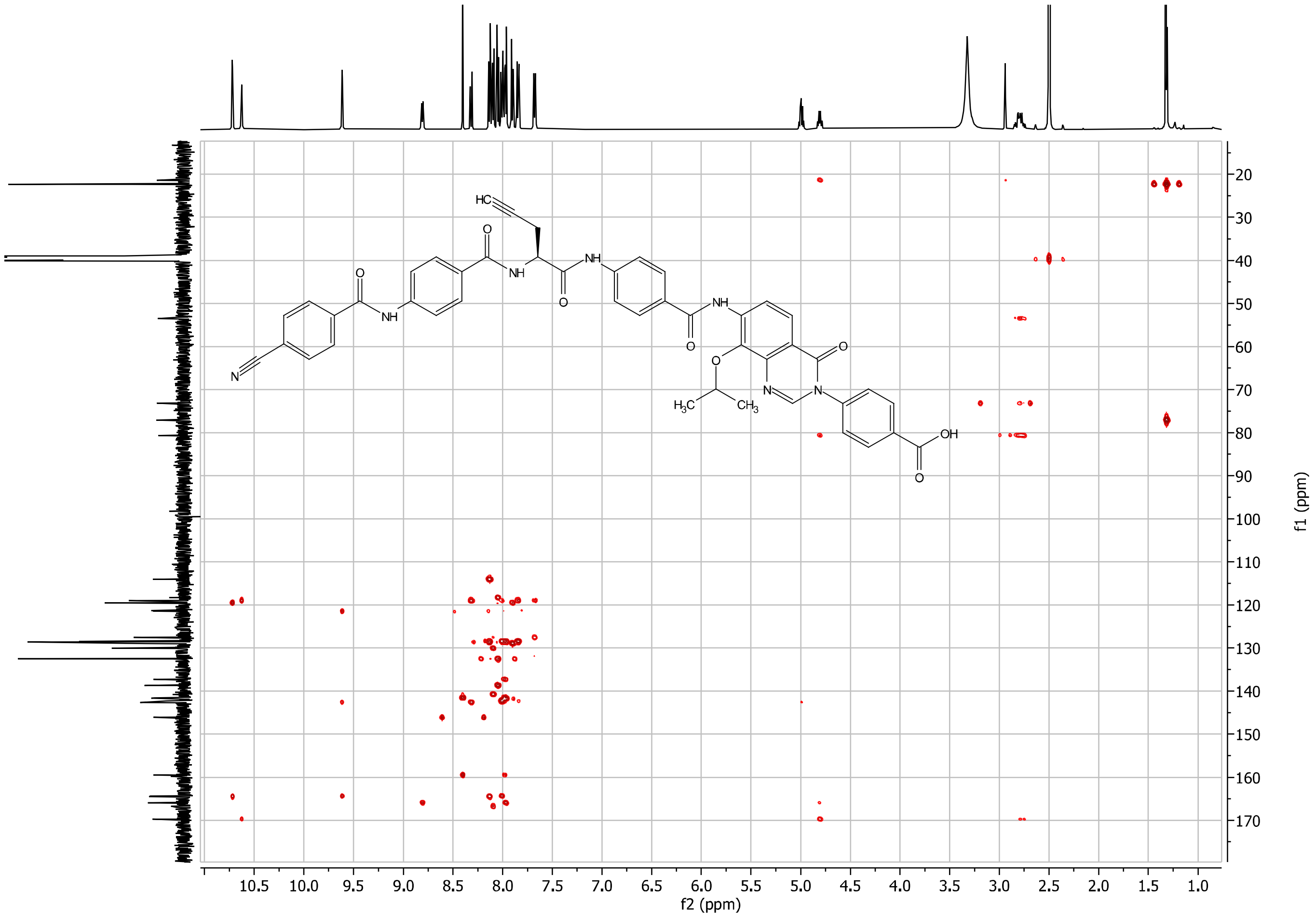

f1 (ppm)
f2 (ppm)
HC
NH
O
N
OH
H3C
CH3

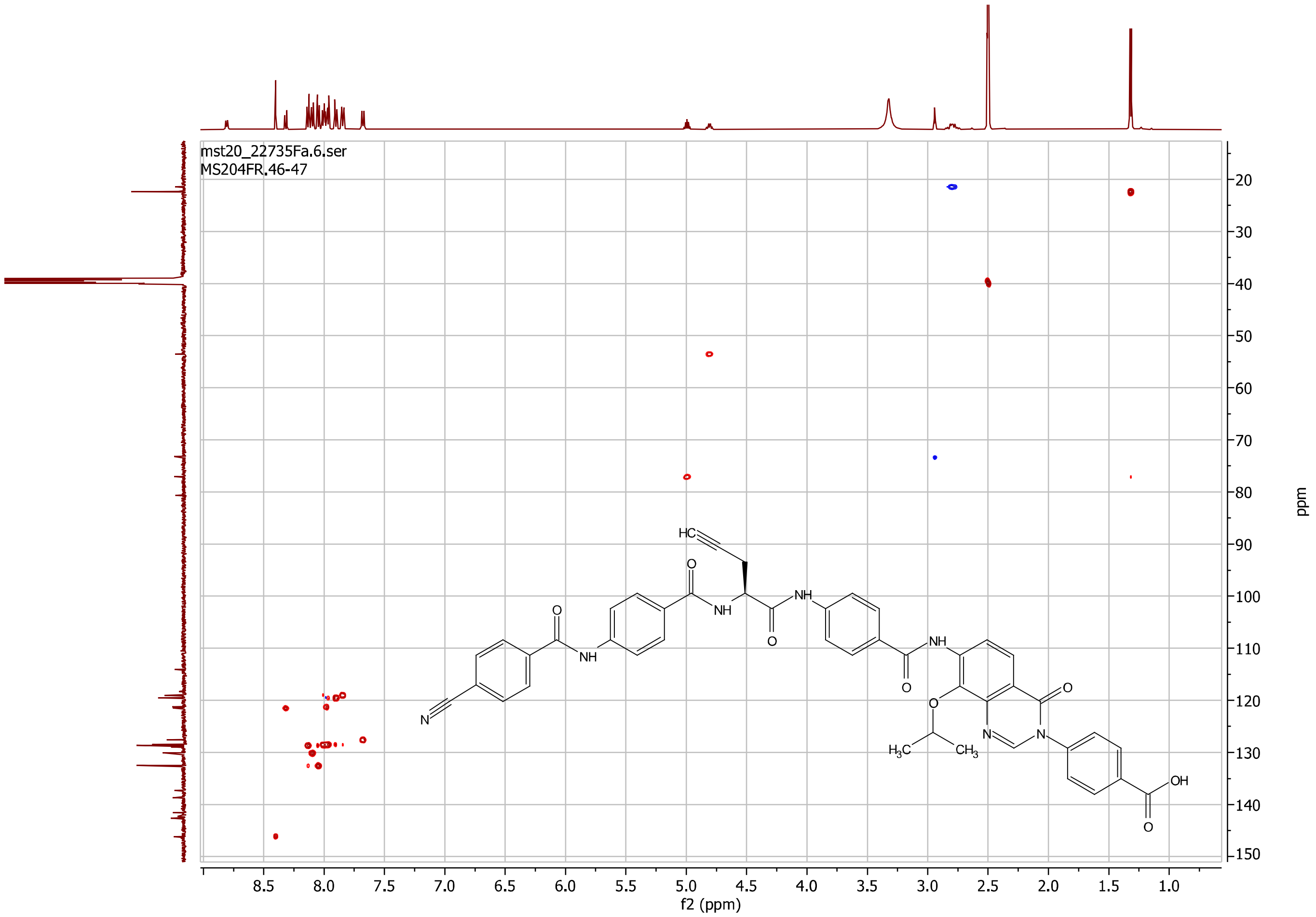
mst20_22735Fa.6.ser
MS204FR.46-47
f2 (ppm)
ppm

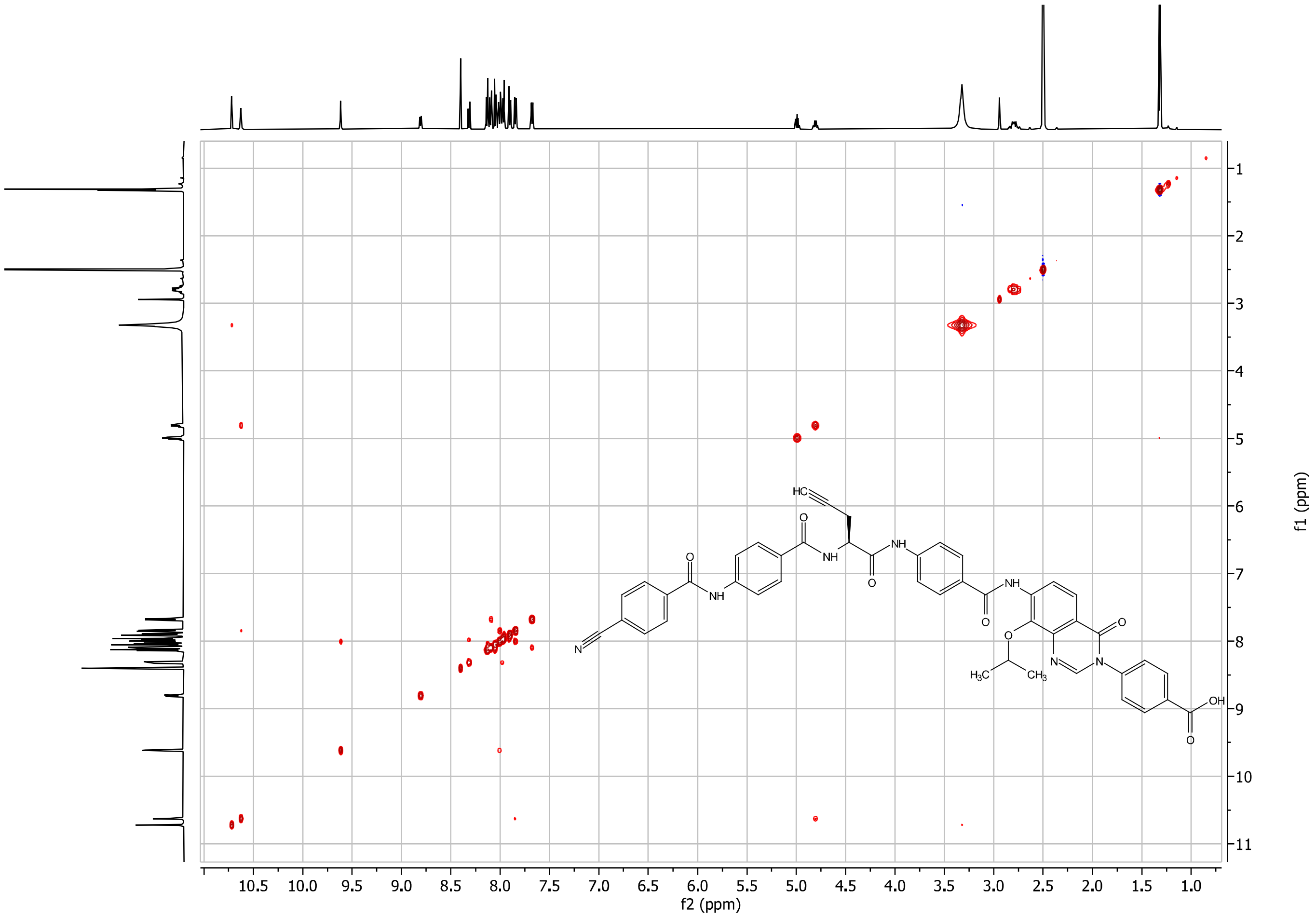
f2 (ppm)
f1 (ppm)

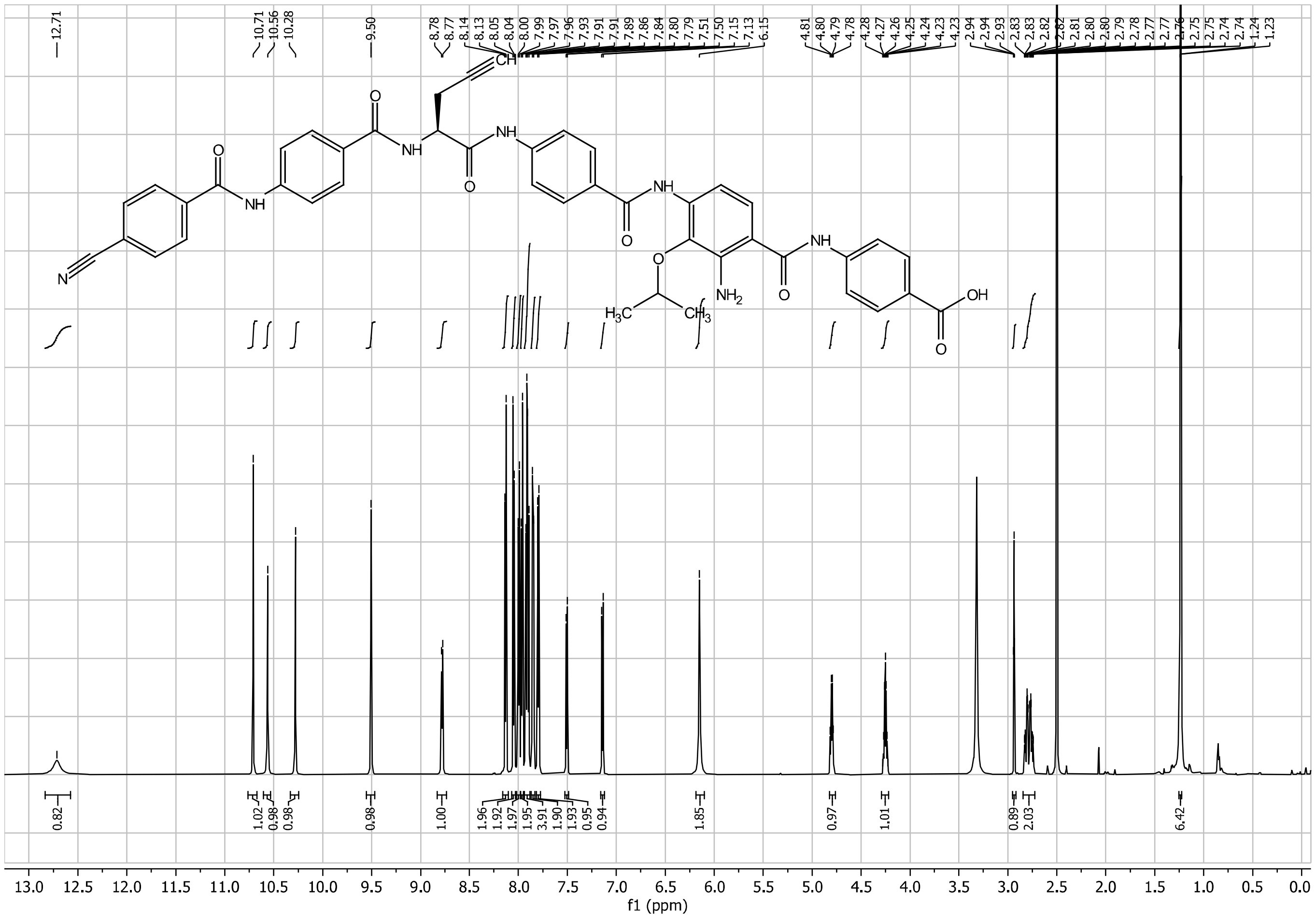
12.71
10.71 10.56 10.28
9.50
8.78 8.77 8.14 8.13 8.05 8.04 8.00 7.99 7.97 7.96 7.93 7.93 7.91 7.91 7.89 7.86 7.84 7.80 7.79 7.51 7.50 7.15 7.13 6.15
4.81 4.80 4.79 4.78 4.28 4.27 4.26 4.25 4.24 4.23 4.23
2.94 2.94 2.93 2.83 2.83 2.82 2.82 2.81 2.80 2.80 2.79 2.78 2.77 2.77 2.76 2.75 2.75 2.74 2.74 1.24 1.23
0.82 1.02 0.98 0.98 0.98 1.00 1.96 1.92 1.97 1.95 3.91 1.90 1.93 0.95 0.94 1.85 0.97 1.01 0.89 2.03 6.42
f1 (ppm)

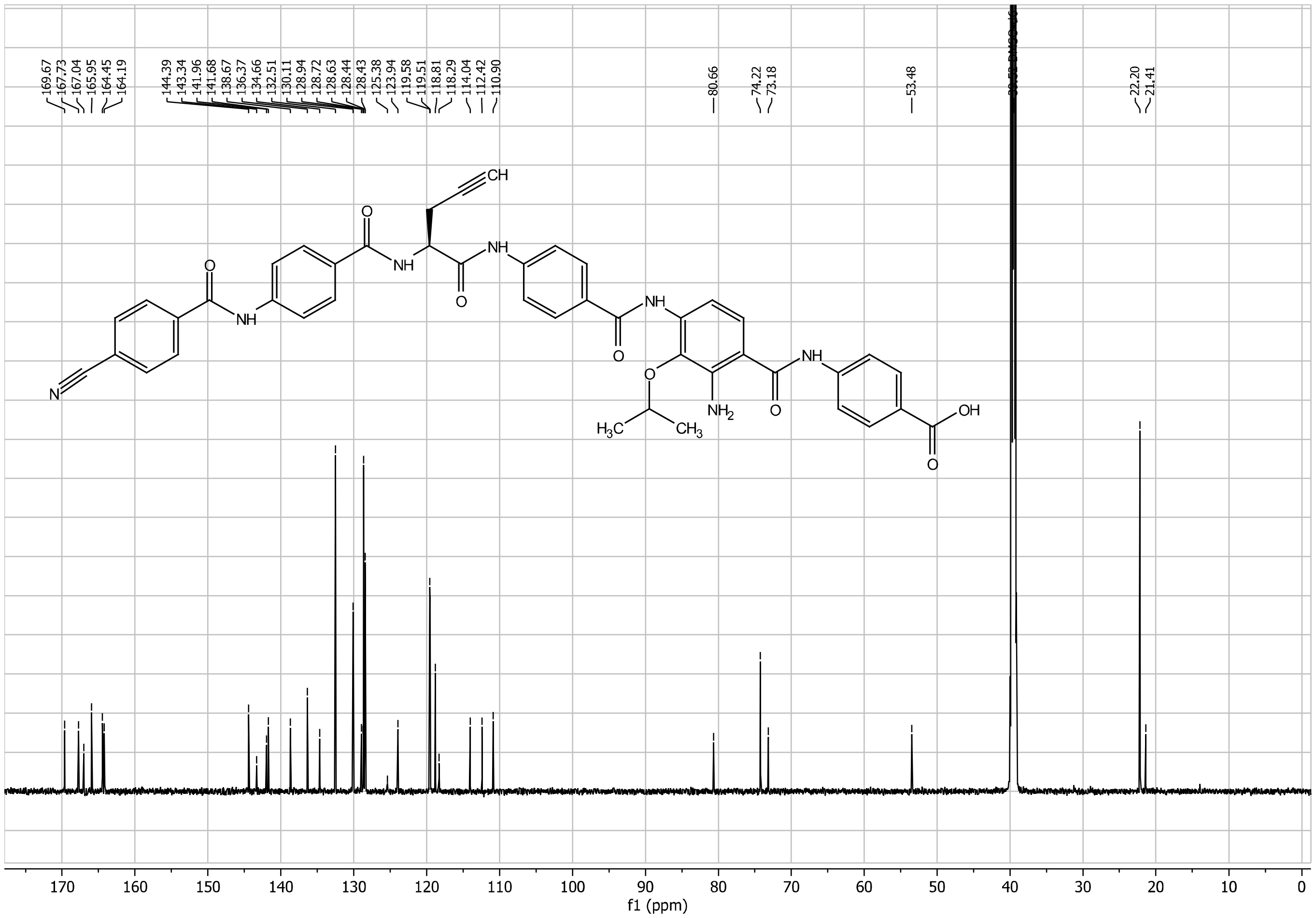
169.67 167.73 167.04 165.95 164.45 164.19
144.39 143.34 141.96 141.68 138.67 136.37 134.66 132.51 130.11 128.94 128.72 128.63 128.44 128.43 125.38 123.94 119.58 119.51 118.81 118.29 114.04 112.42 110.90
80.66
74.22 73.18
53.48
22.20 21.41
f1 (ppm)

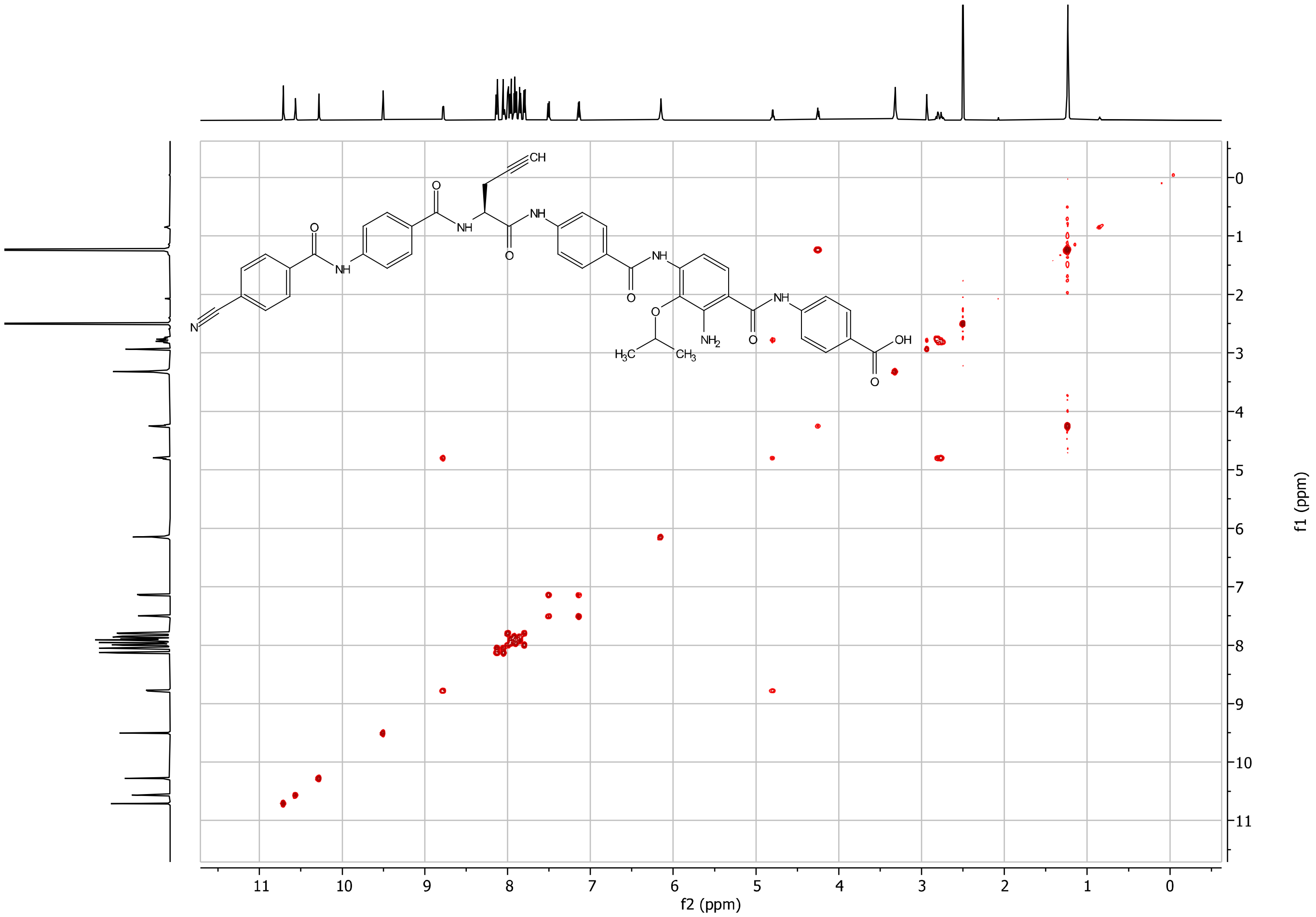
CH
O
NH
O
NH
O
NH
O
NH
O
N
O
NH
NH2
O
OH
O
H3C
CH3
f2 (ppm)
f1 (ppm)

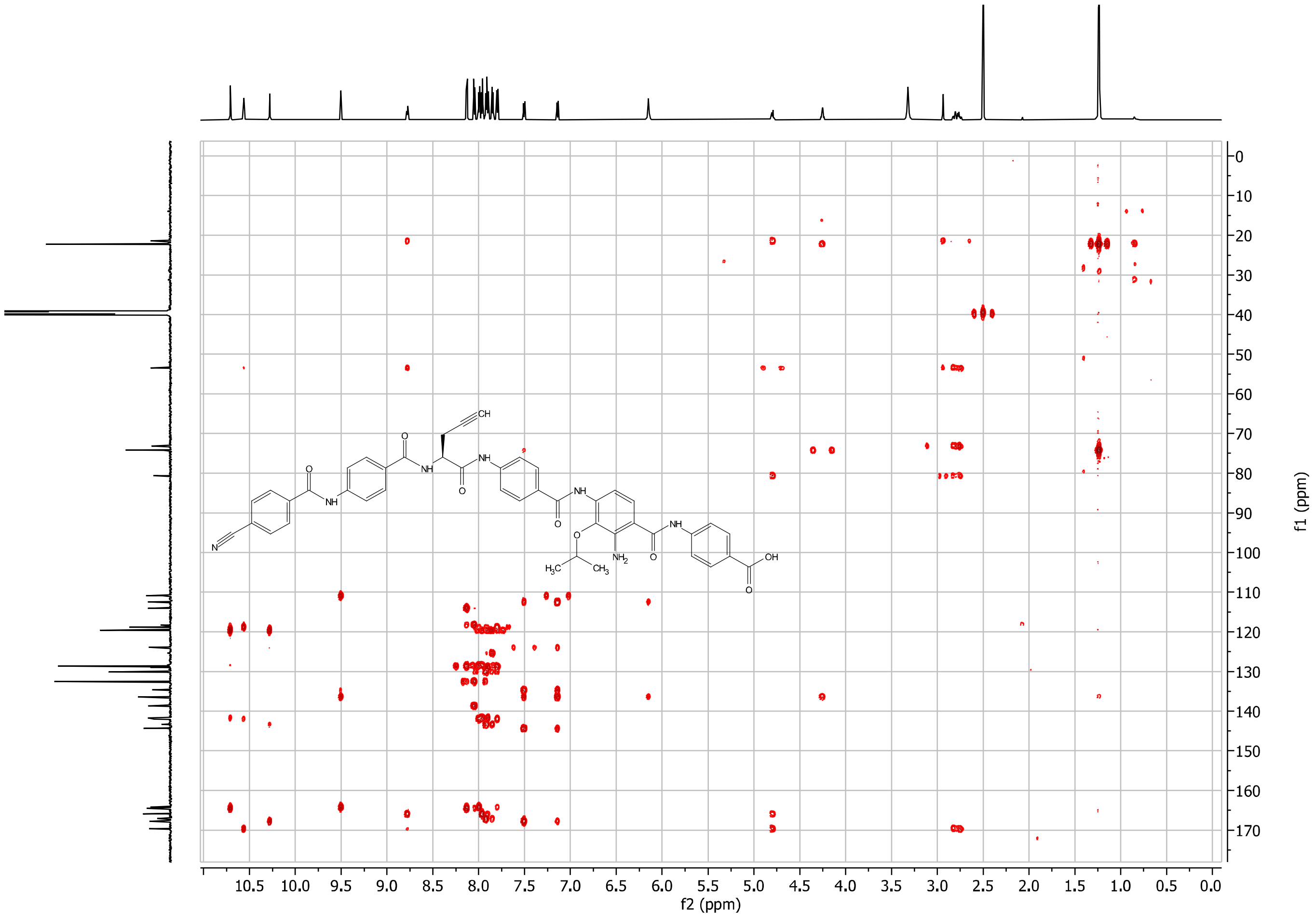
CH
O
NH
O
NH
O
NH
O
NH
O
N
O
NH
NH2
O
OH
O
H3C
CH3
f2 (ppm)
f1 (ppm)

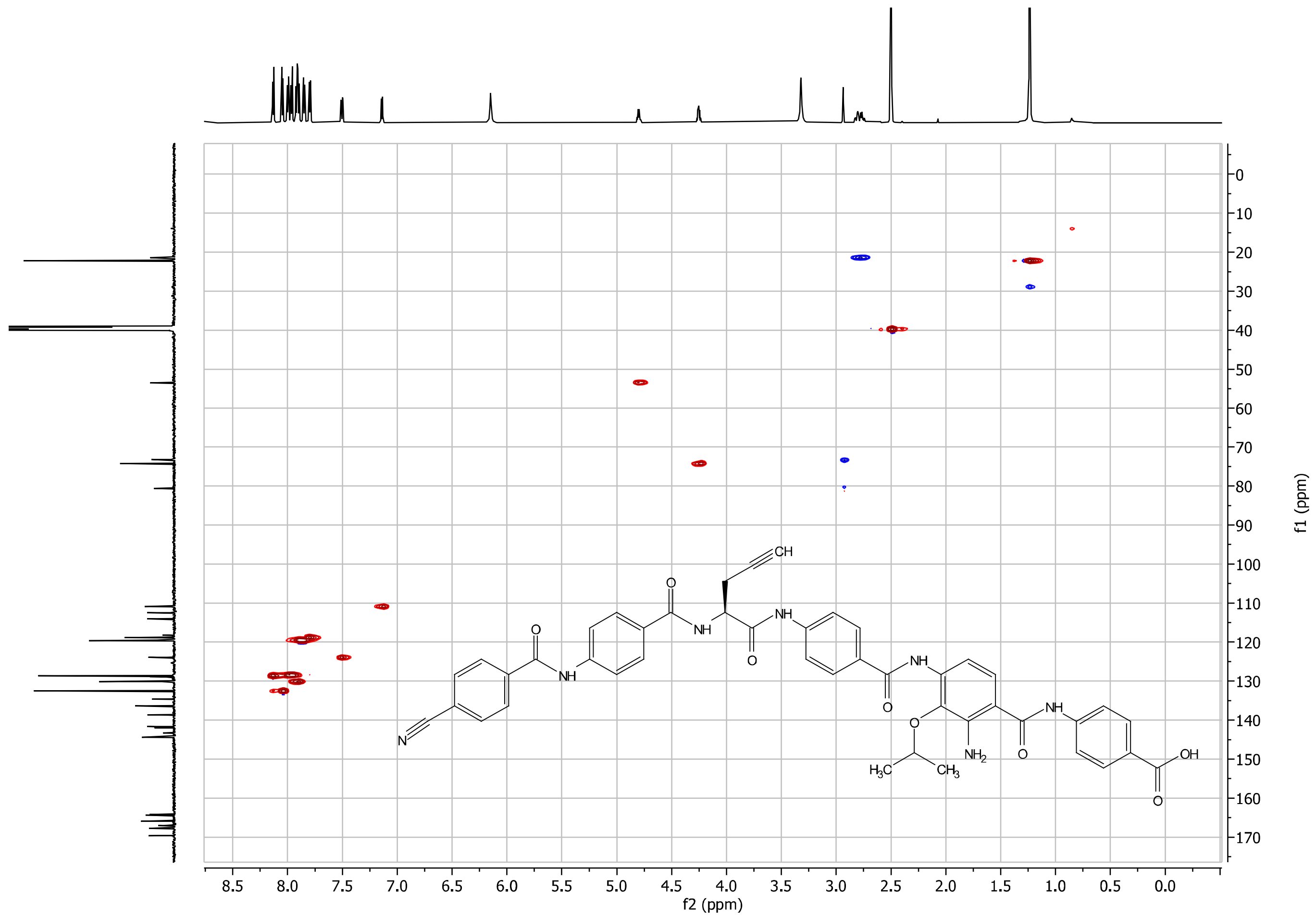
f2 (ppm)
f1 (ppm)
NH
NH
NH
NH
NH
NH₂
OH
CH
N
H₃C
CH₃

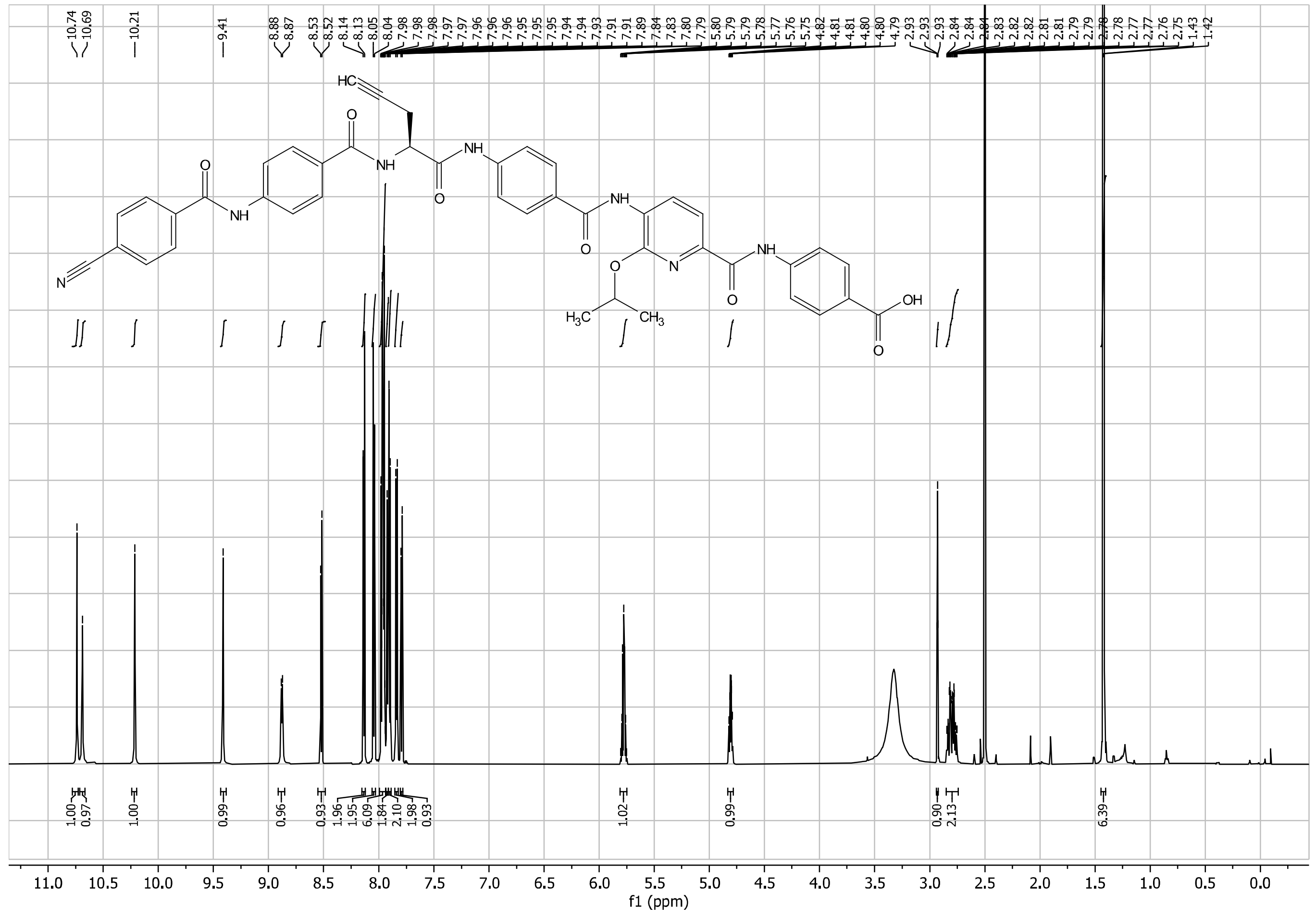
f1 (ppm)
HC
NH
NH
NH
NH
NH
N
OH
H₃C
CH₃

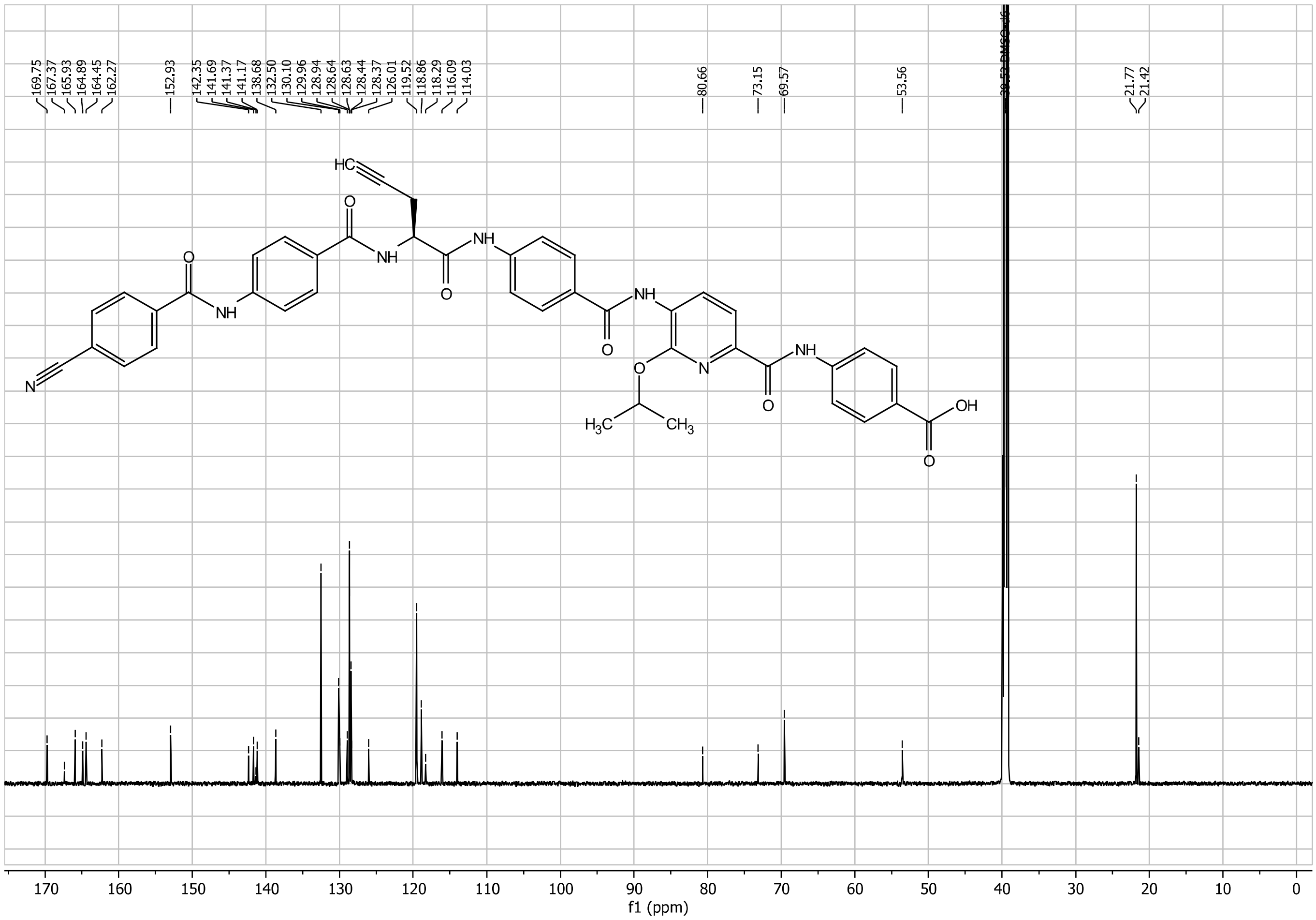
169.75
167.37
165.93
164.89
164.45
162.27
152.93
142.35
141.69
141.37
141.17
138.68
132.50
130.10
129.96
128.94
128.64
128.63
128.44
128.37
126.01
119.52
118.86
118.29
116.09
114.03
80.66
73.15
69.57
53.56
21.77
21.42
HC
O
NH
O
NH
NH
O
NH
O
N
O
N
NH
H3C
CH3
O
OH
O
f1 (ppm)

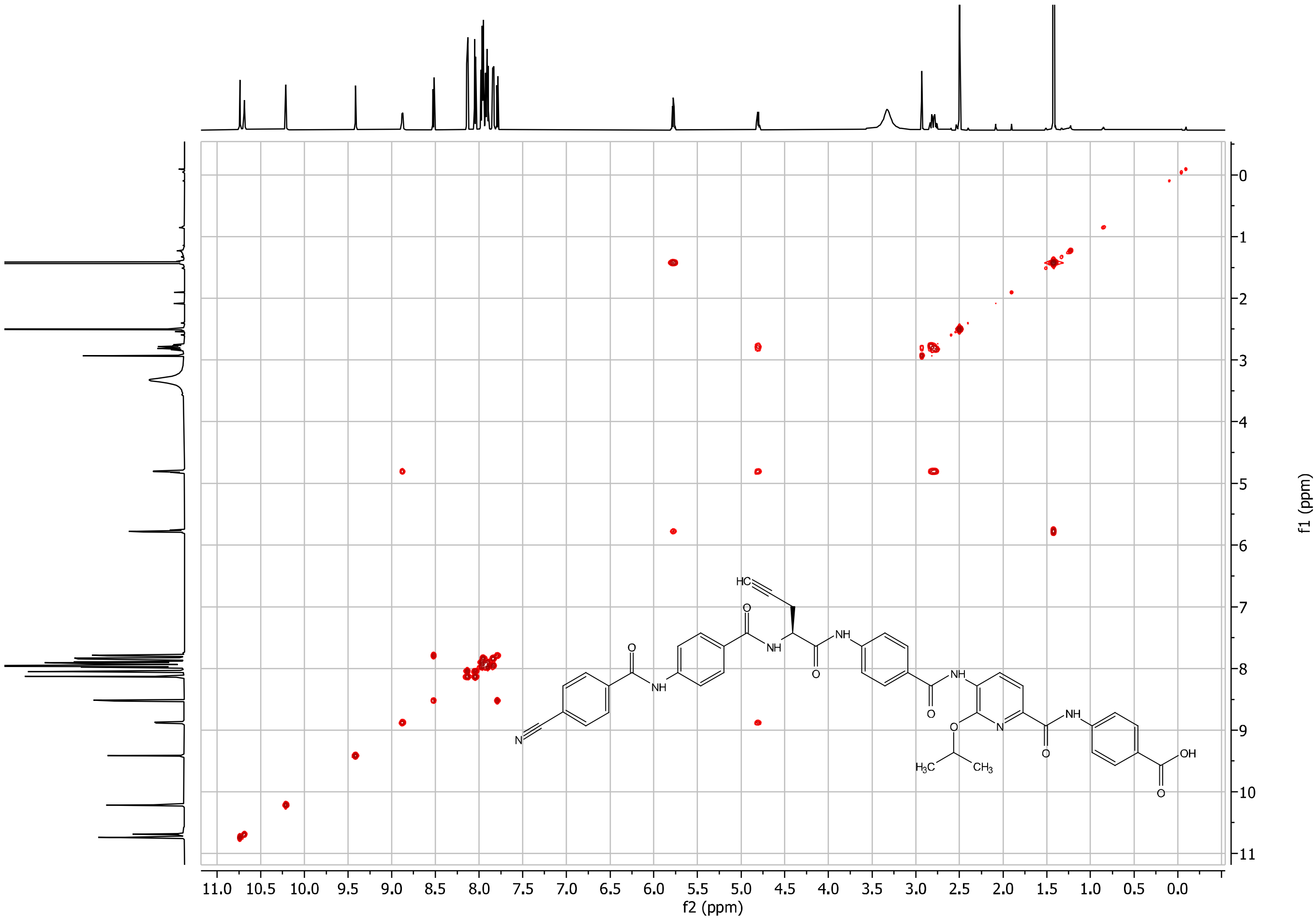
f2 (ppm)
f1 (ppm)

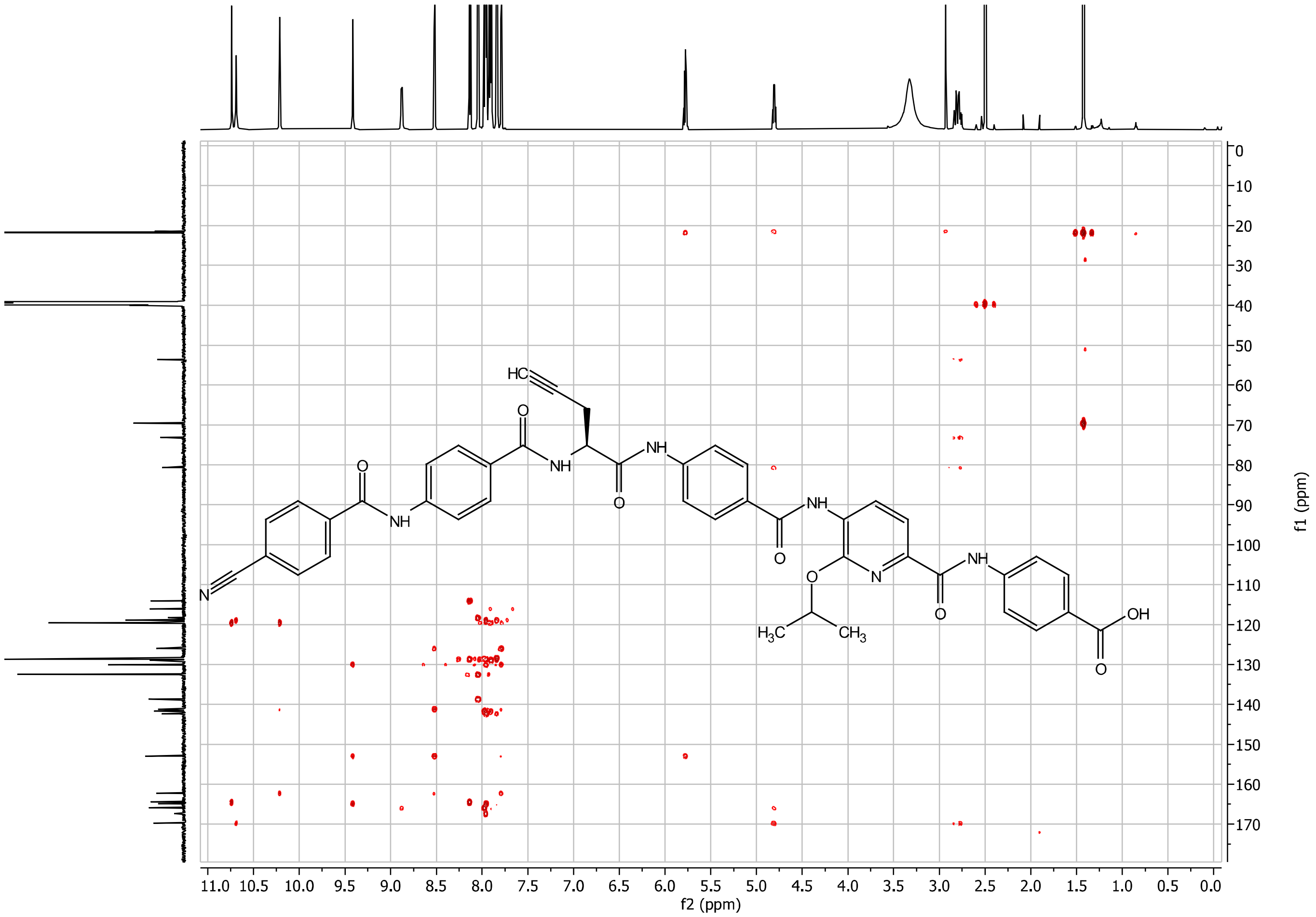
f1 (ppm)
f2 (ppm)
HC
O
NH
O
NH
NH
O
N
NH
O
O
N
NH
H3C
CH3
O
OH
O

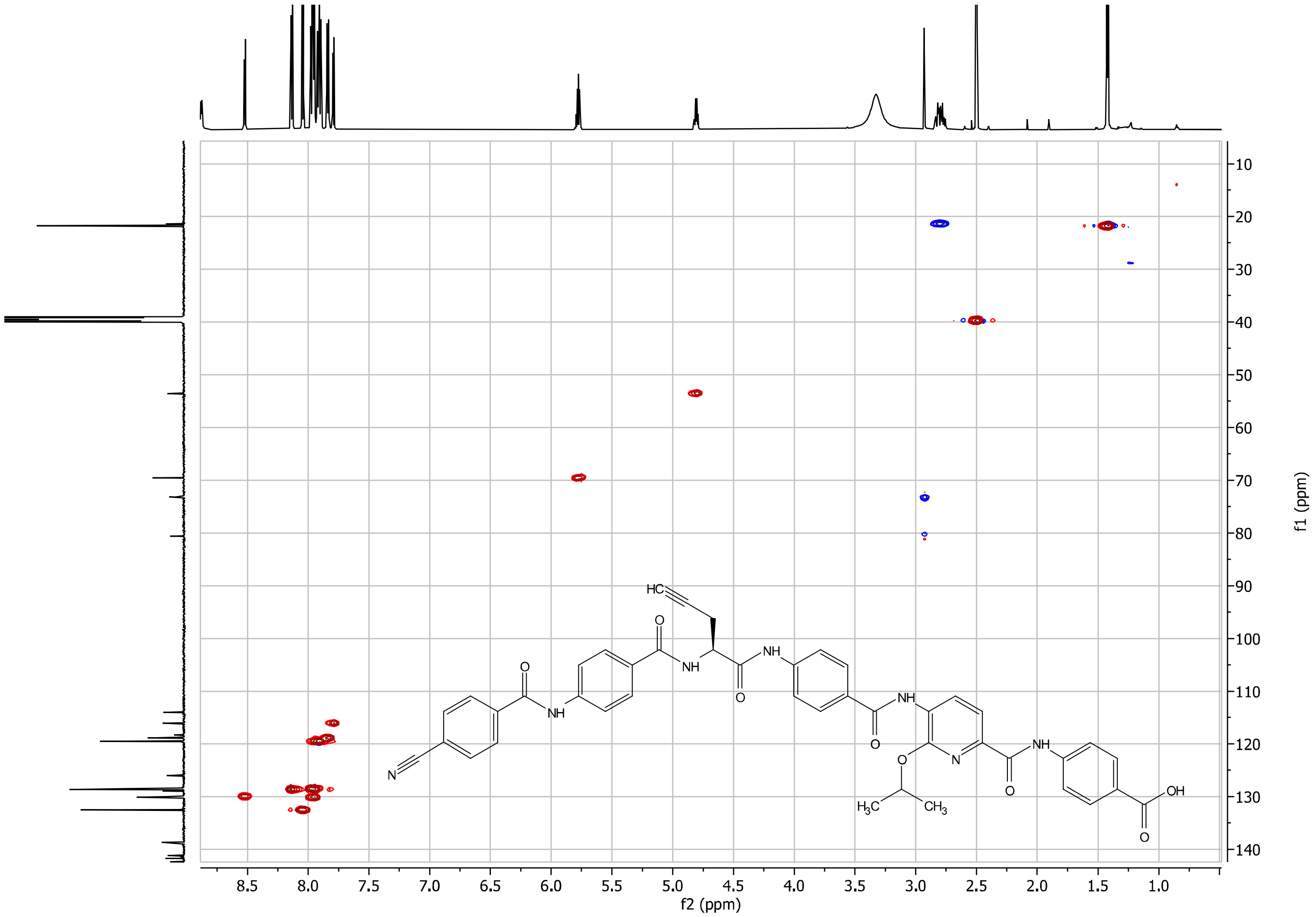
f1 (ppm)
f2 (ppm)
HC
O
NH
O
NH
NH
O
N
NH
O
O
N
NH
H3C
CH3
O
OH
O

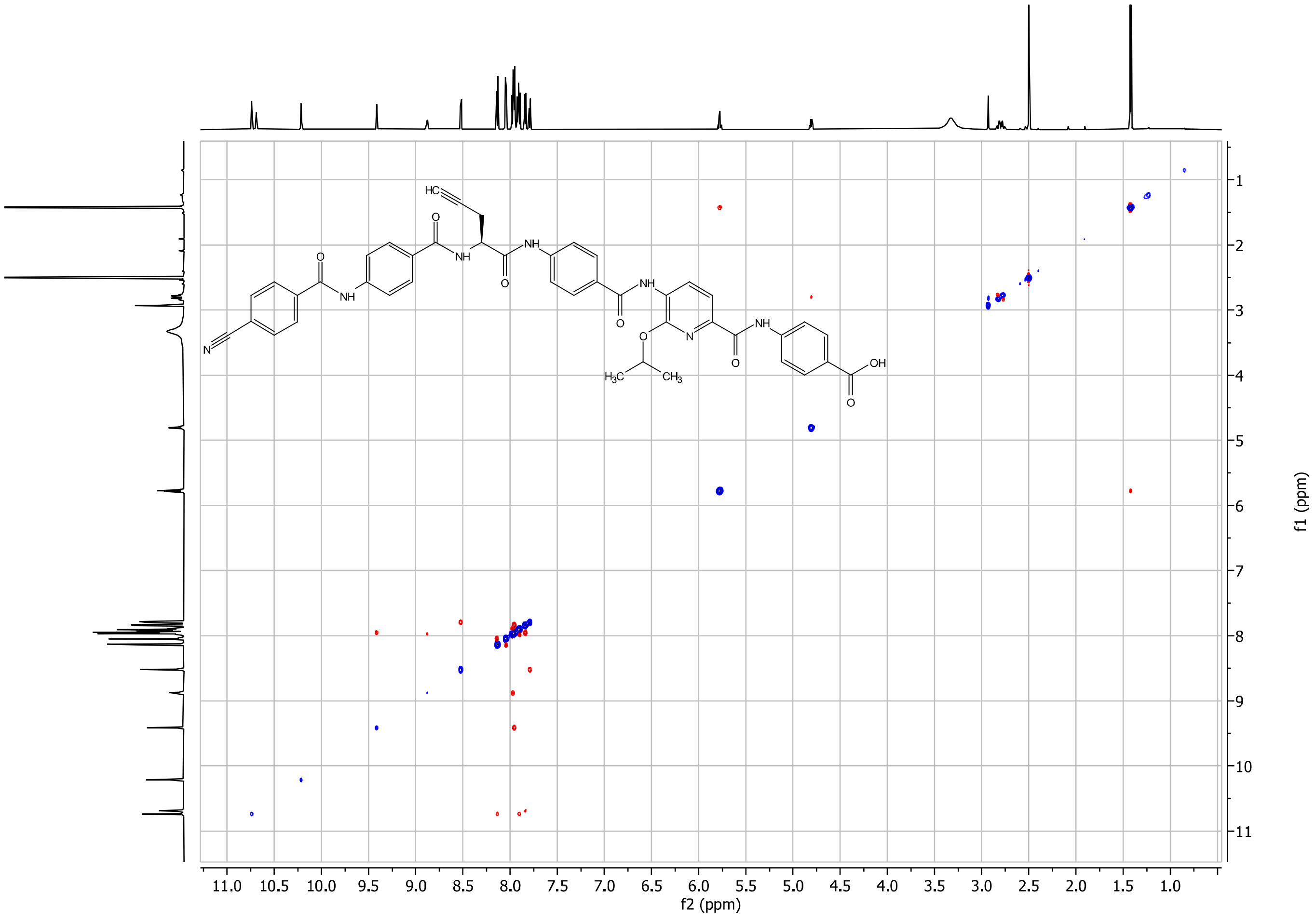
f2 (ppm)
f1 (ppm)

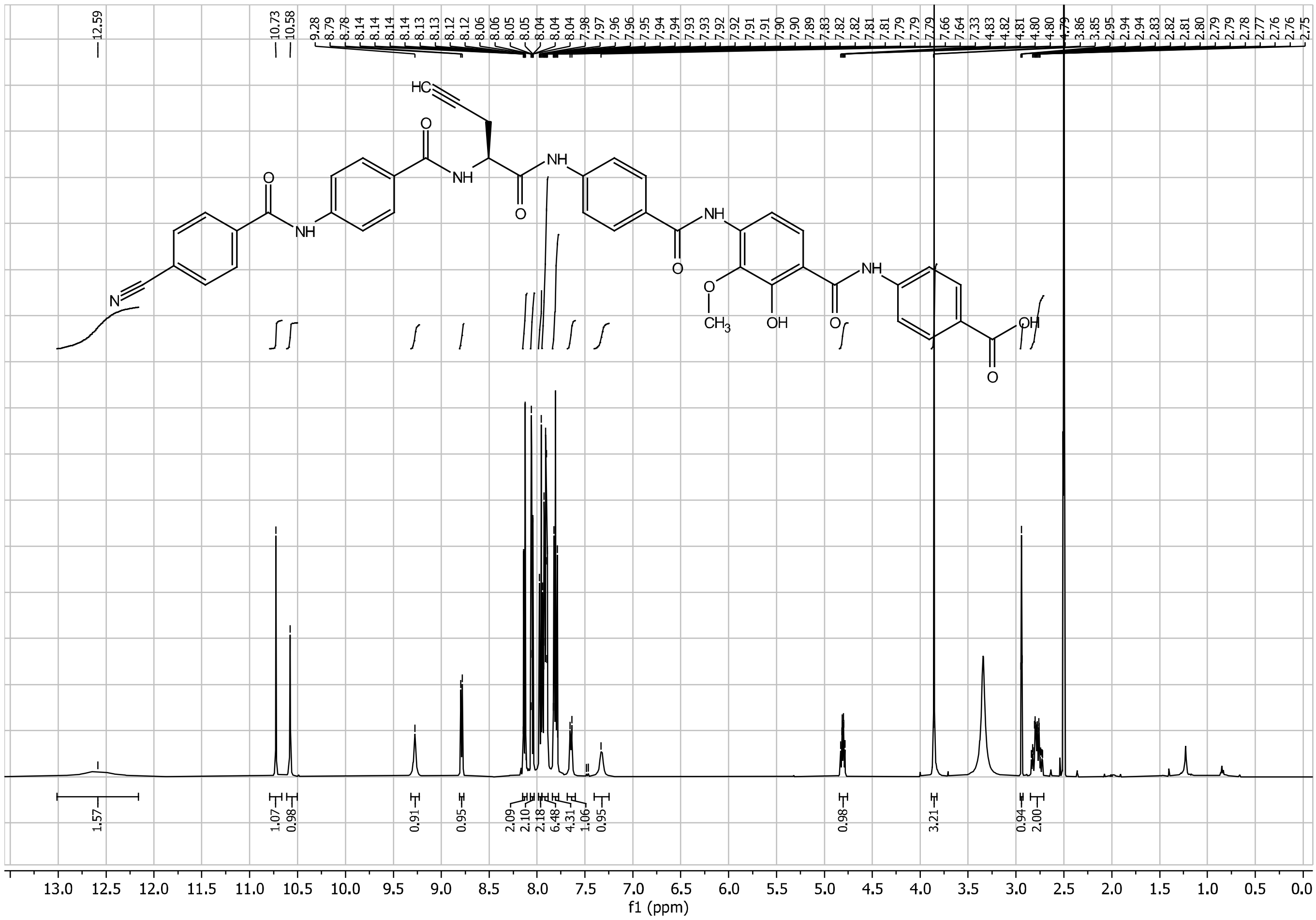
f1 (ppm)

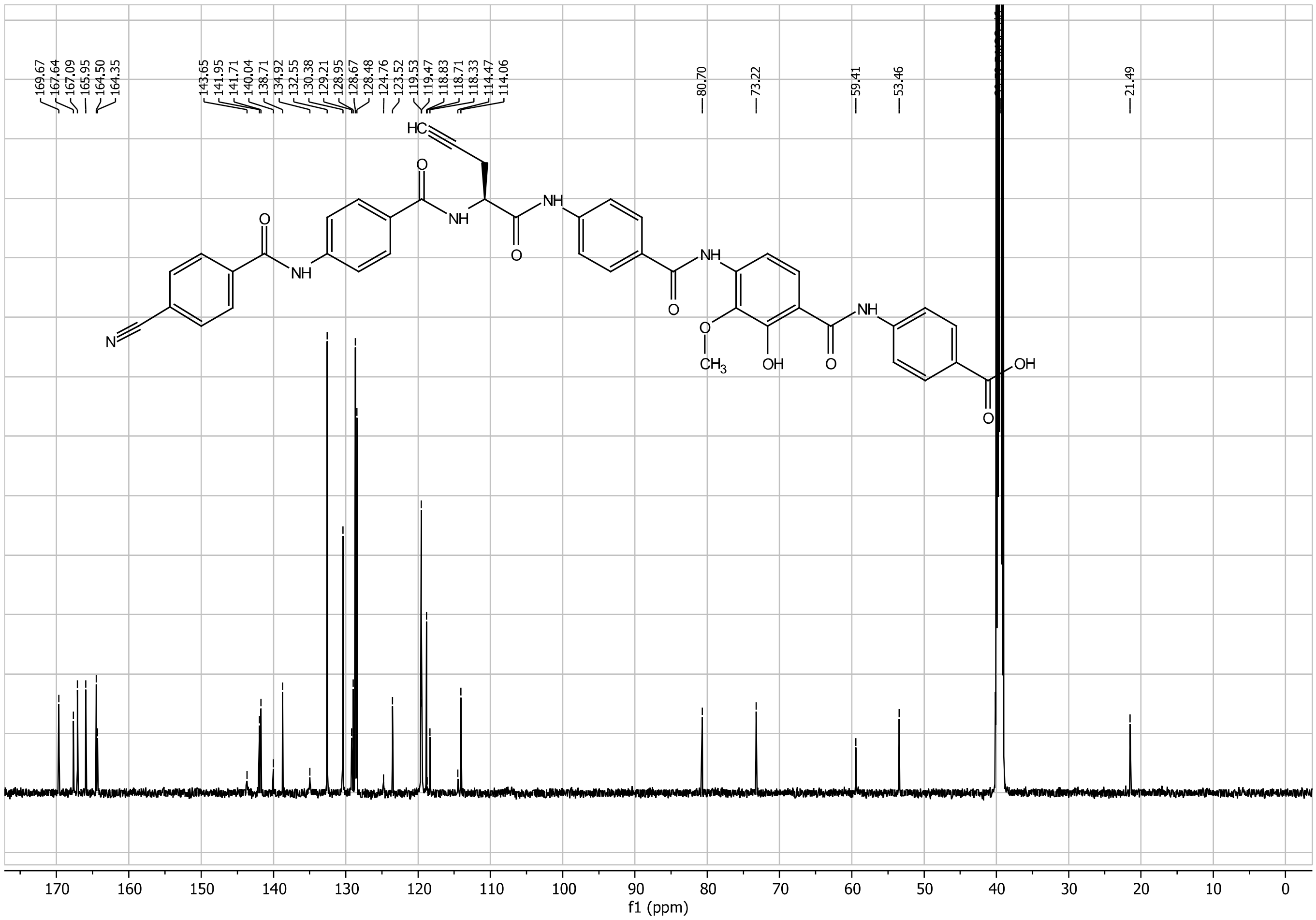
169.67
167.64
167.09
165.95
164.50
164.35
143.65
141.95
141.71
140.04
138.71
134.92
132.55
130.38
129.21
128.95
128.67
128.48
124.76
123.52
119.53
119.47
118.83
118.71
118.33
114.47
114.06
80.70
73.22
59.41
53.46
21.49
f1 (ppm)

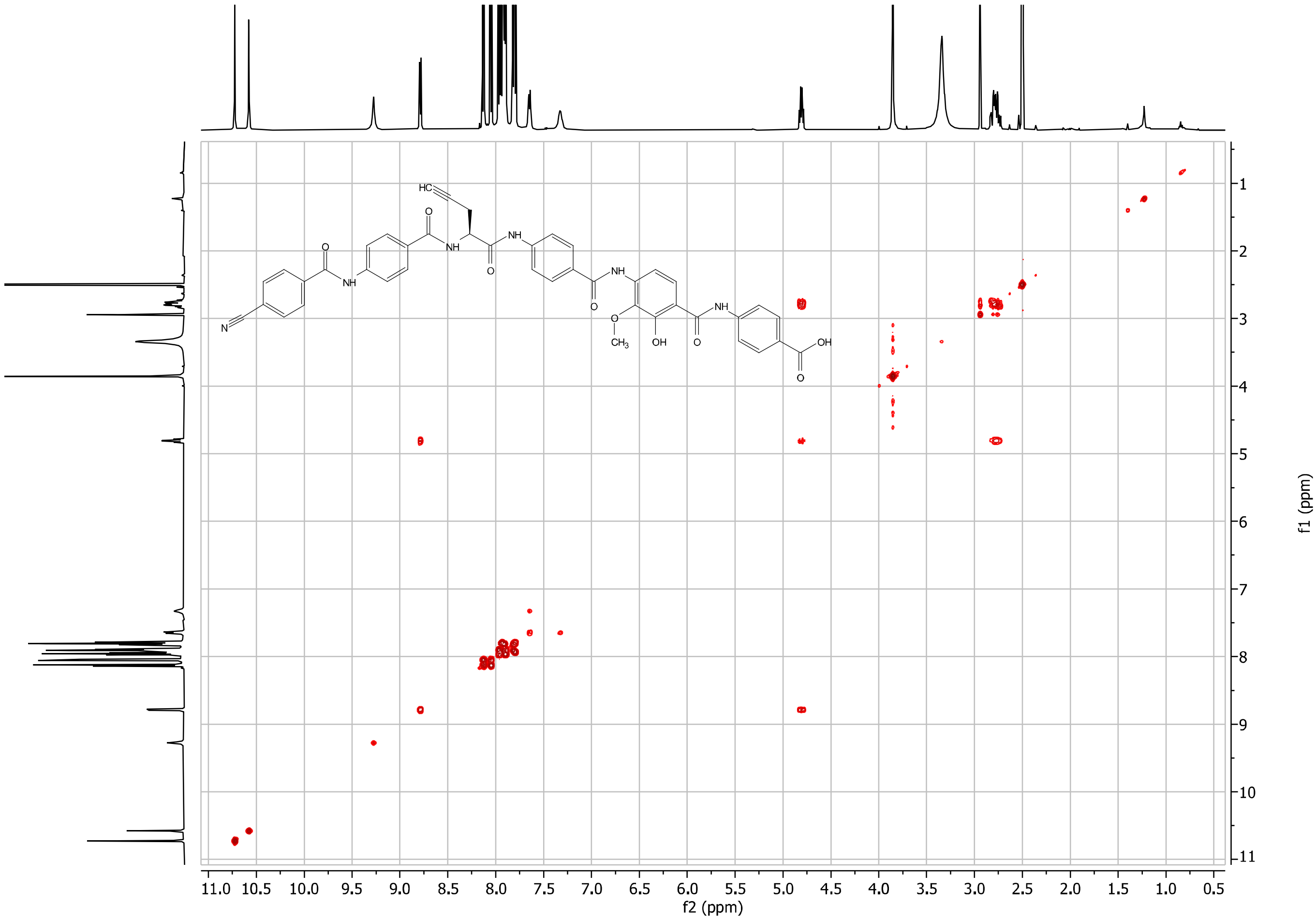
f2 (ppm)
f1 (ppm)

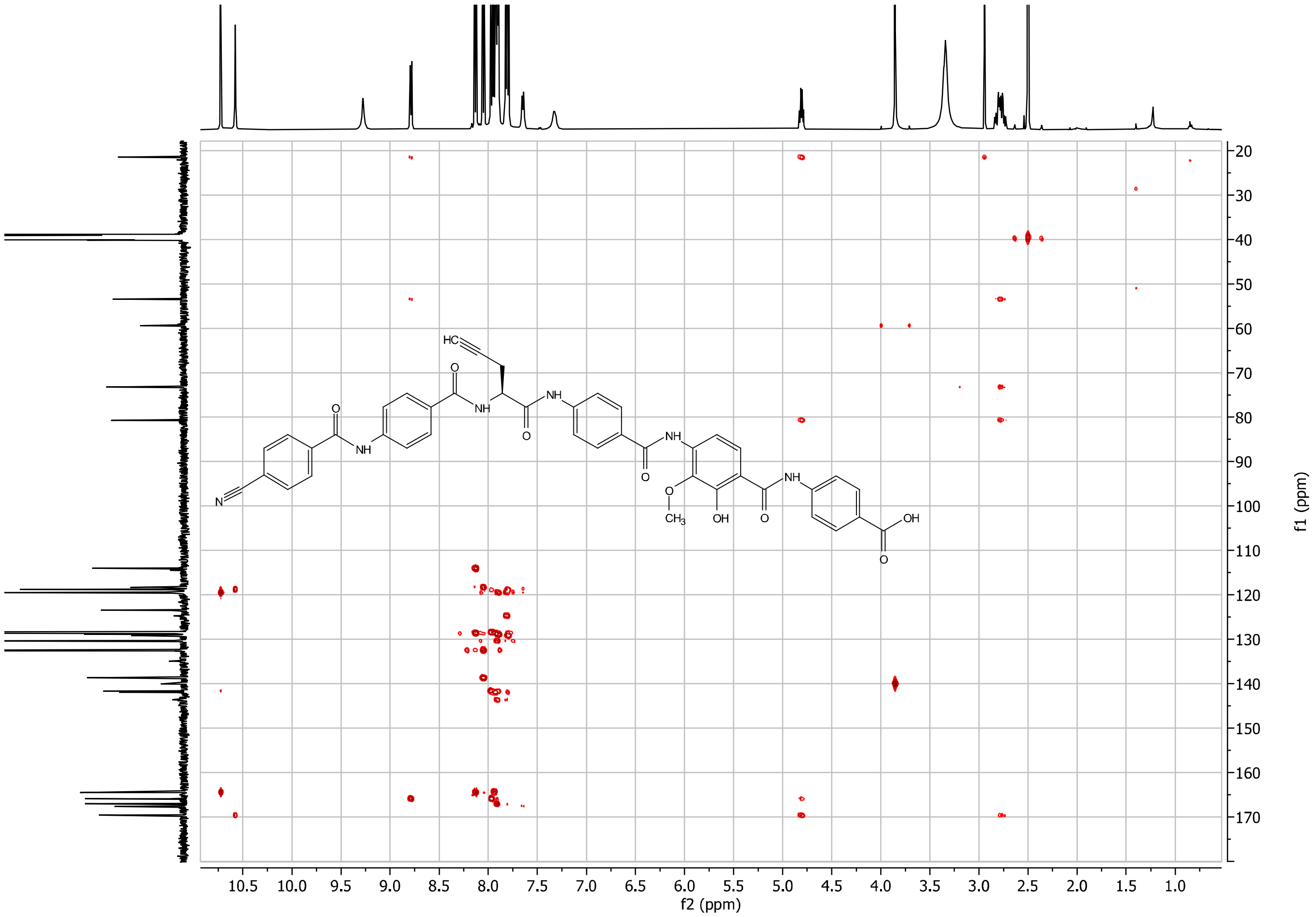
f2 (ppm)
f1 (ppm)
10.5 10.0 9.5 9.0 8.5 8.0 7.5 7.0 6.5 6.0 5.5 5.0 4.5 4.0 3.5 3.0 2.5 2.0 1.5 1.0
20 30 40 50 60 70 80 90 100 110 120 130 140 150 160 170
HC
O
NH
NH
O
NH
O
NH
O
O
NH
N
CH3
OH
O
OH
O

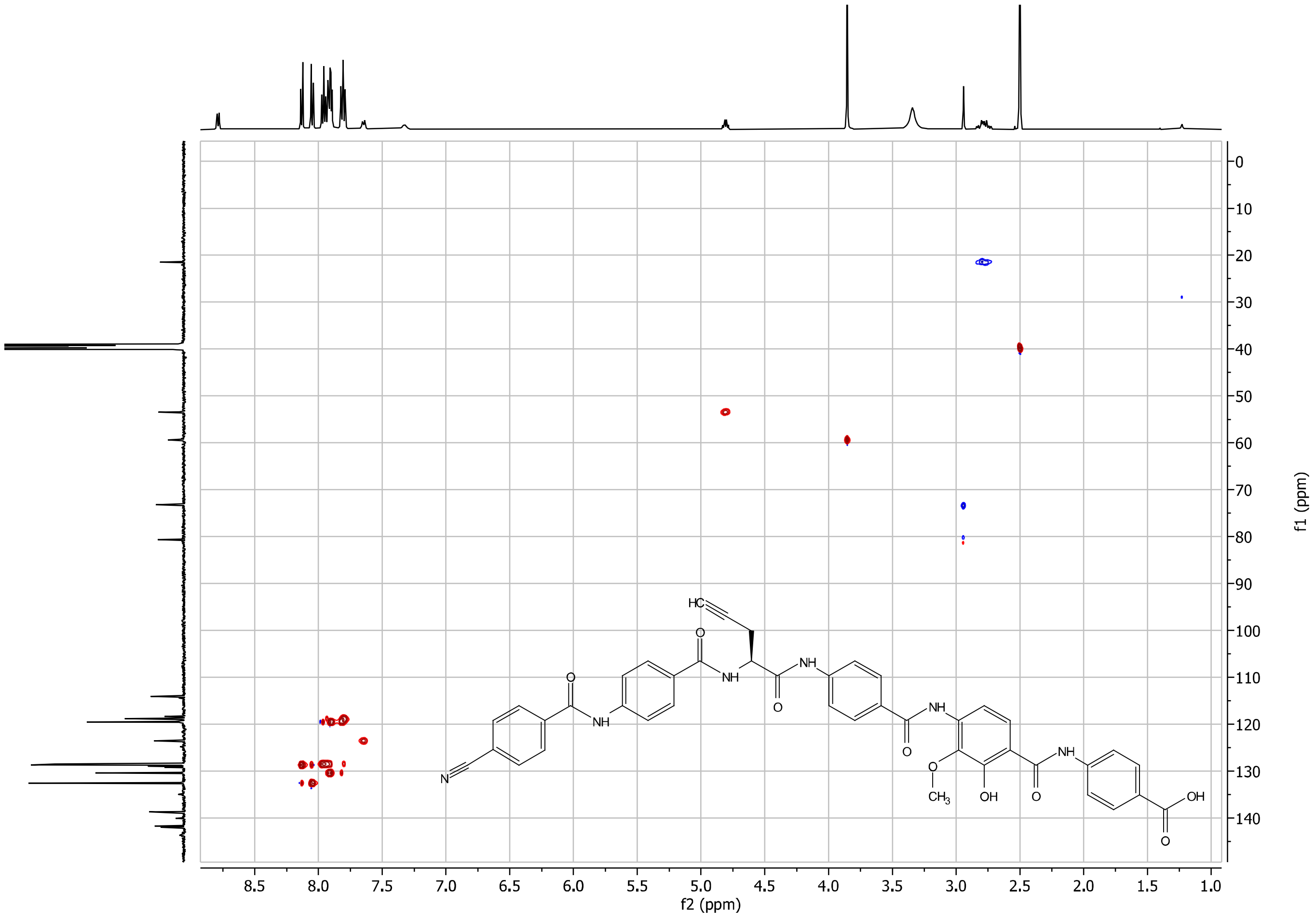
f2 (ppm)
f1 (ppm)
8.5 8.0 7.5 7.0 6.5 6.0 5.5 5.0 4.5 4.0 3.5 3.0 2.5 2.0 1.5 1.0
0 10 20 30 40 50 60 70 80 90 100 110 120 130 140
HC
O
NH
NH
O
NH
O
NH
O
O
NH
N
CH3
OH
O
OH
O

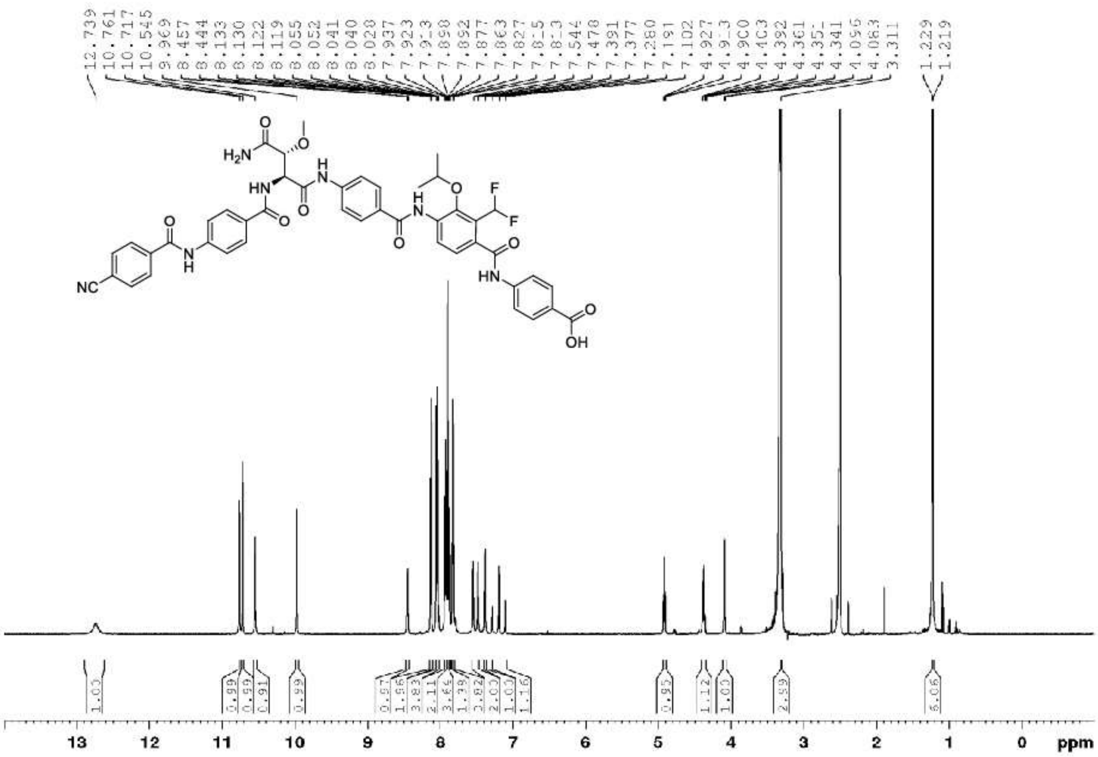

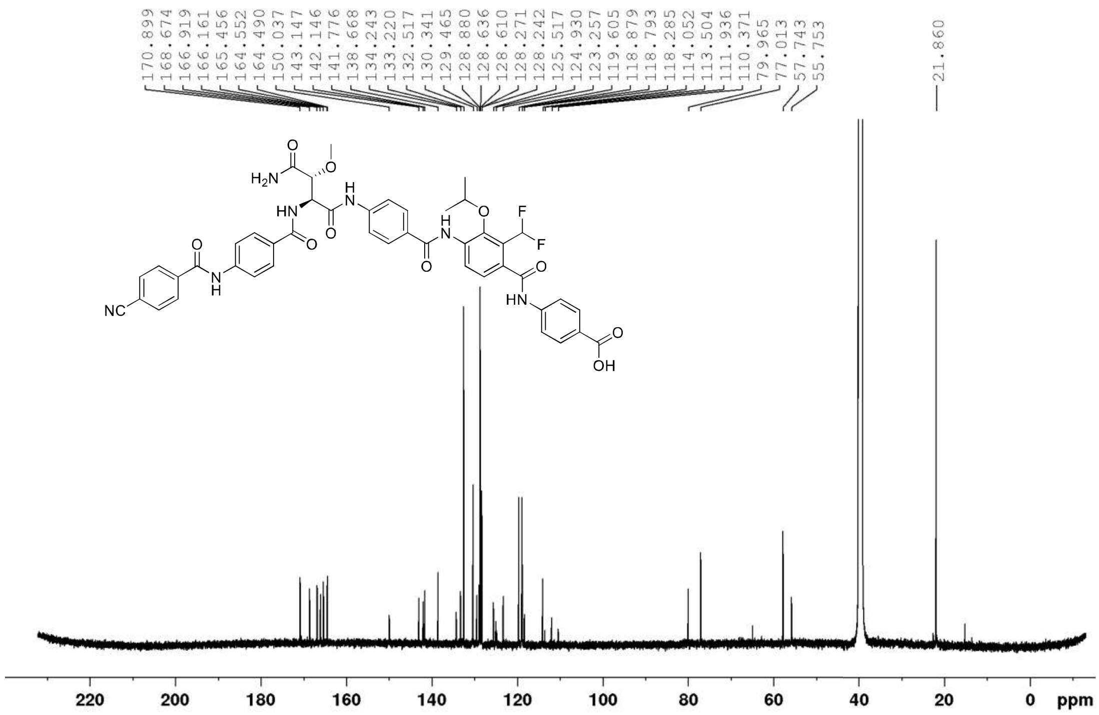
170.899
168.674
166.919
166.161
165.456
164.552
164.490
150.037
143.147
142.146
141.776
138.668
134.243
133.220
132.517
130.341
129.465
128.880
128.636
128.610
128.271
128.242
125.517
124.930
123.257
119.605
118.879
118.793
118.285
114.052
113.504
111.936
110.371
79.965
77.013
57.743
55.753
21.860
220
200
180
160
140
120
100
80
60
40
20
0
ppm

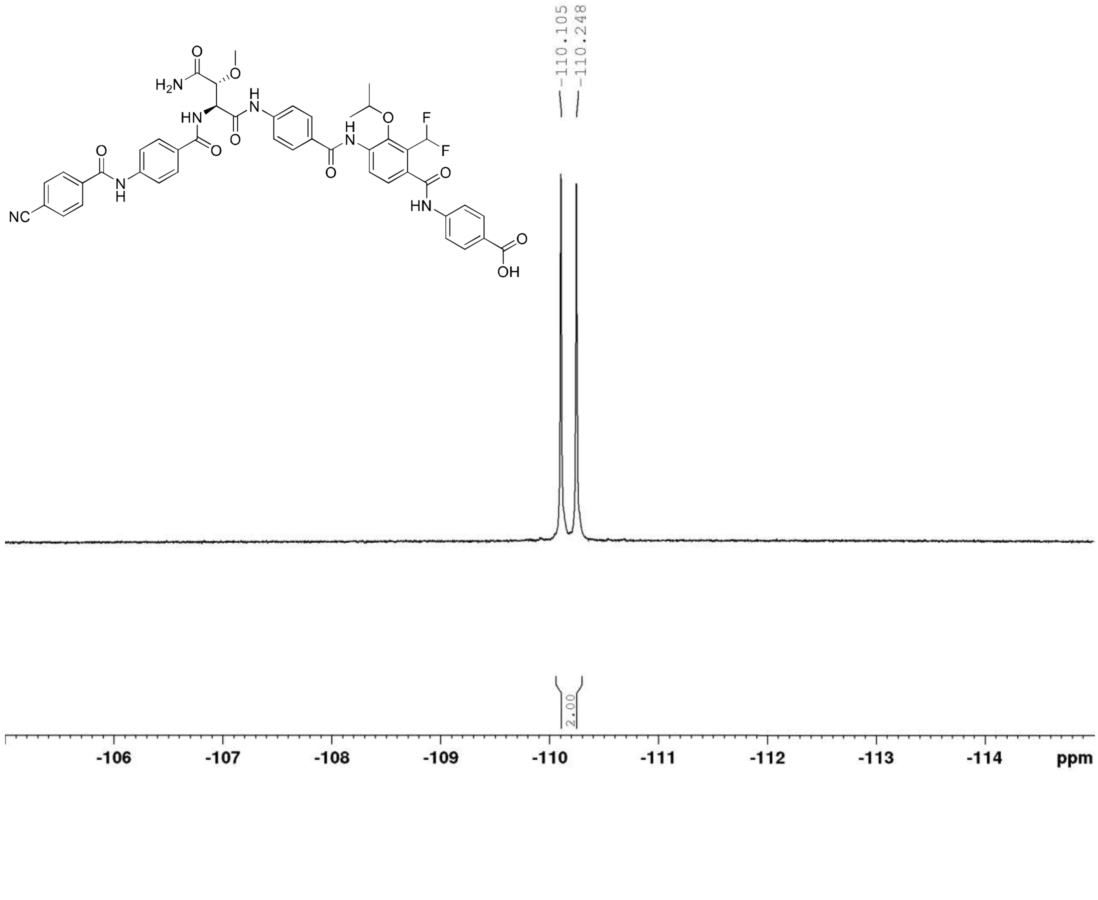
-110.105
-110.248
2.00
-106
-107
-108
-109
-110
-111
-112
-113
-114
ppm

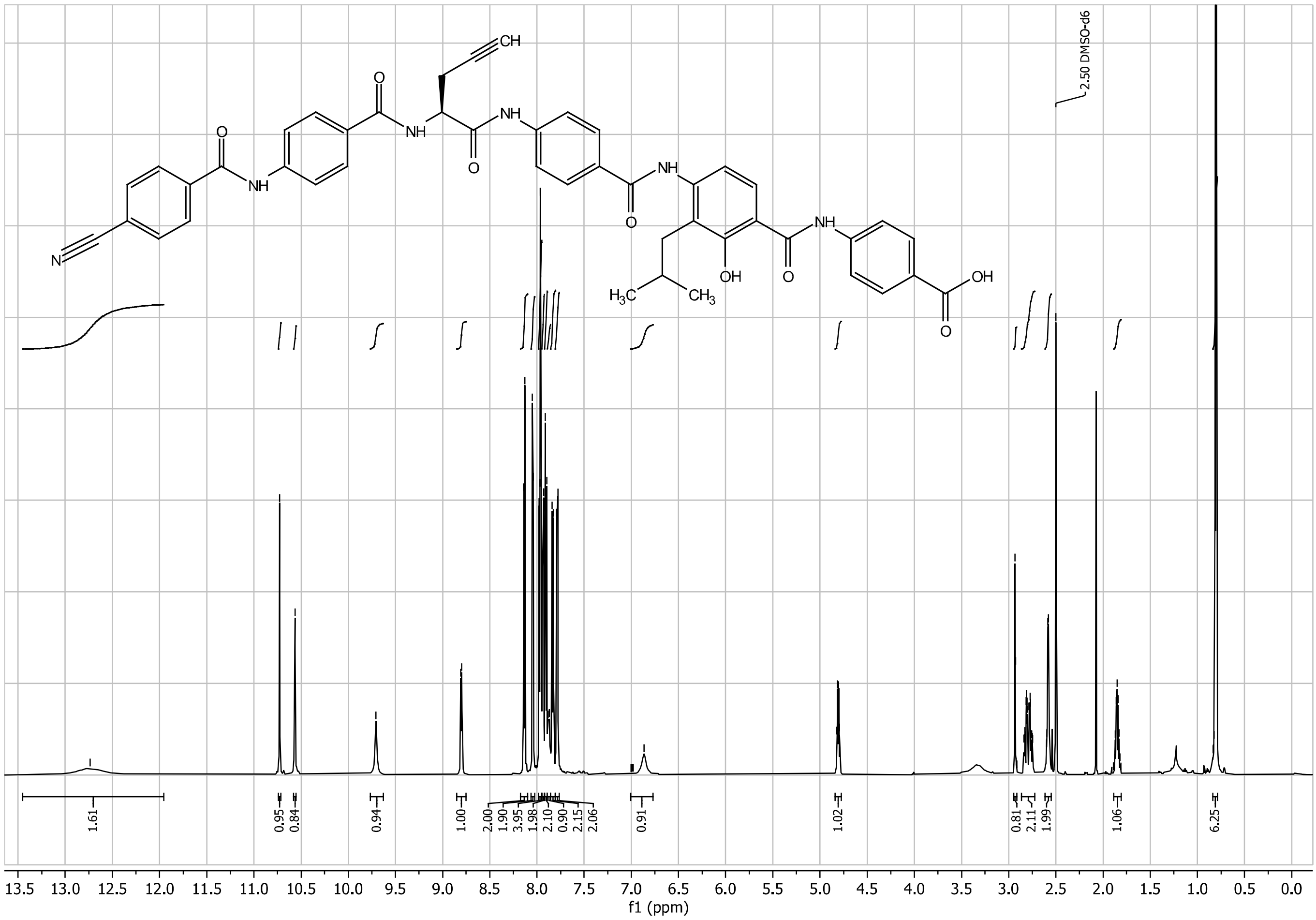
2.50 DMSO-d6
1.61
0.95
0.84
0.94
1.00
2.00
1.90
3.95
1.98
2.10
0.90
2.15
2.06
0.91
1.02
0.81
2.11
1.99
1.06
6.25
f1 (ppm)

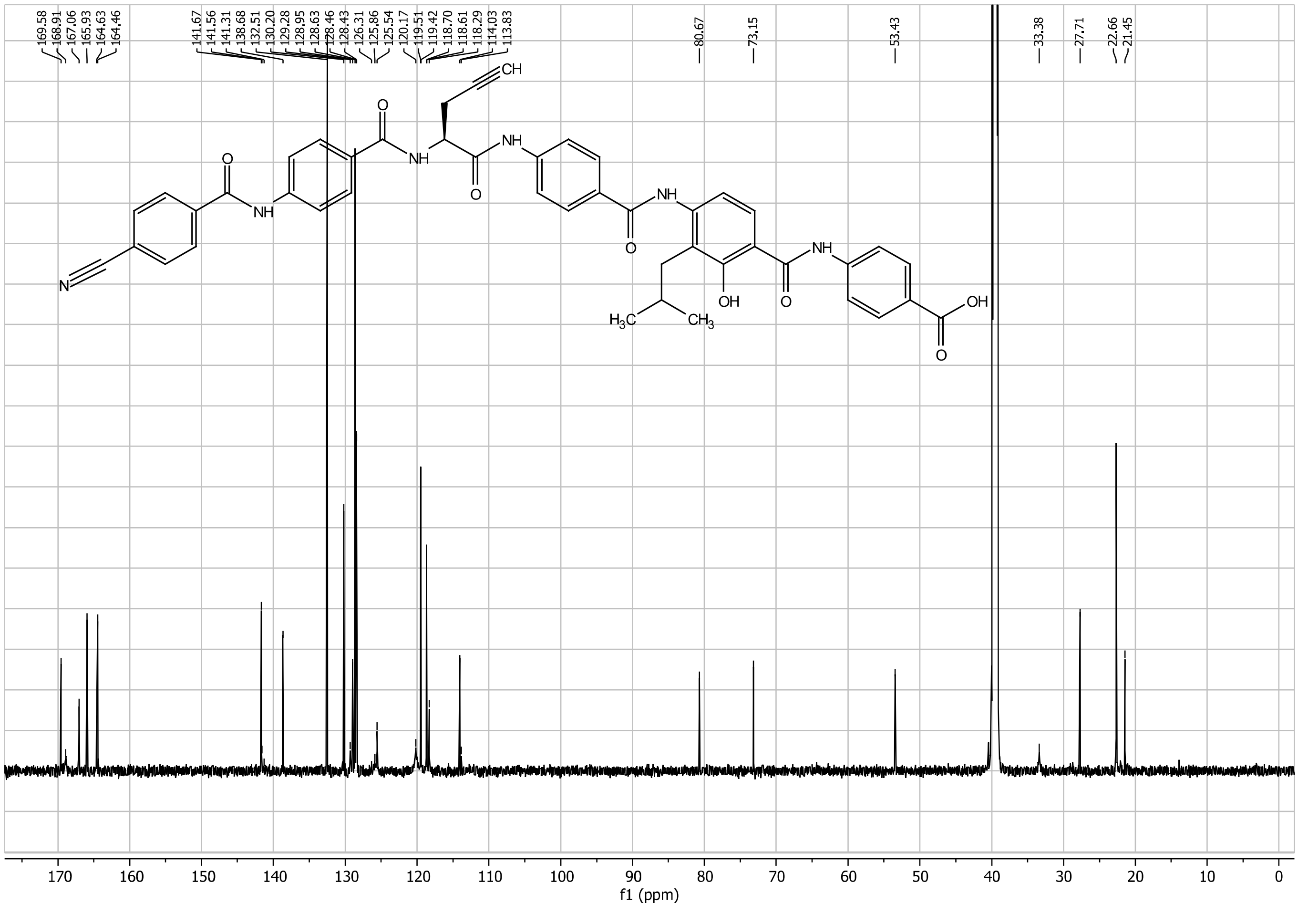
169.58
168.91
167.06
165.93
164.63
164.46
141.67
141.56
141.31
138.68
132.51
130.20
129.28
128.95
128.63
128.46
128.43
126.31
125.86
125.54
120.17
119.51
119.42
118.70
118.61
118.29
114.03
113.83
80.67
73.15
53.43
33.38
27.71
22.66
21.45
f1 (ppm)

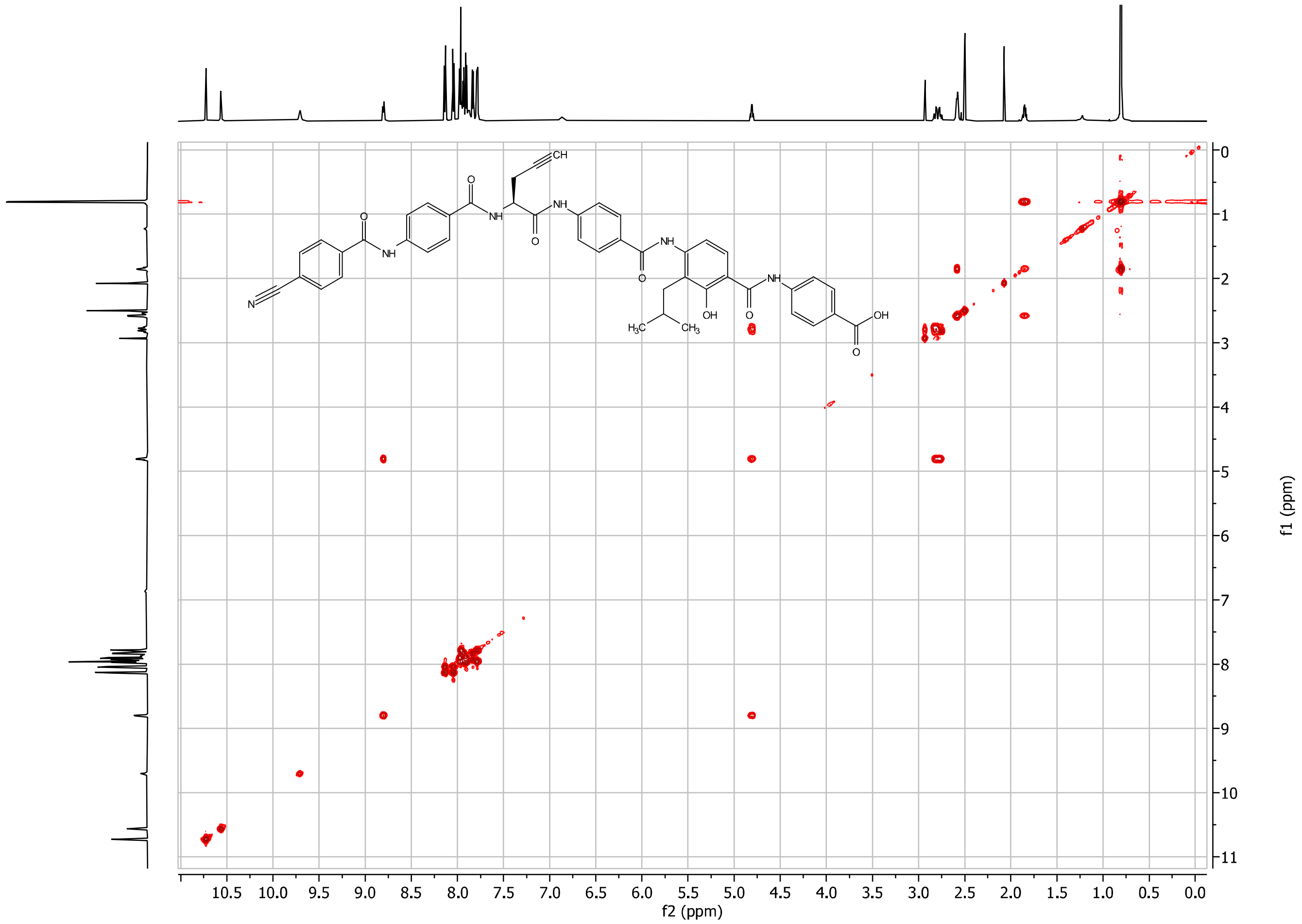
f2 (ppm)
f1 (ppm)

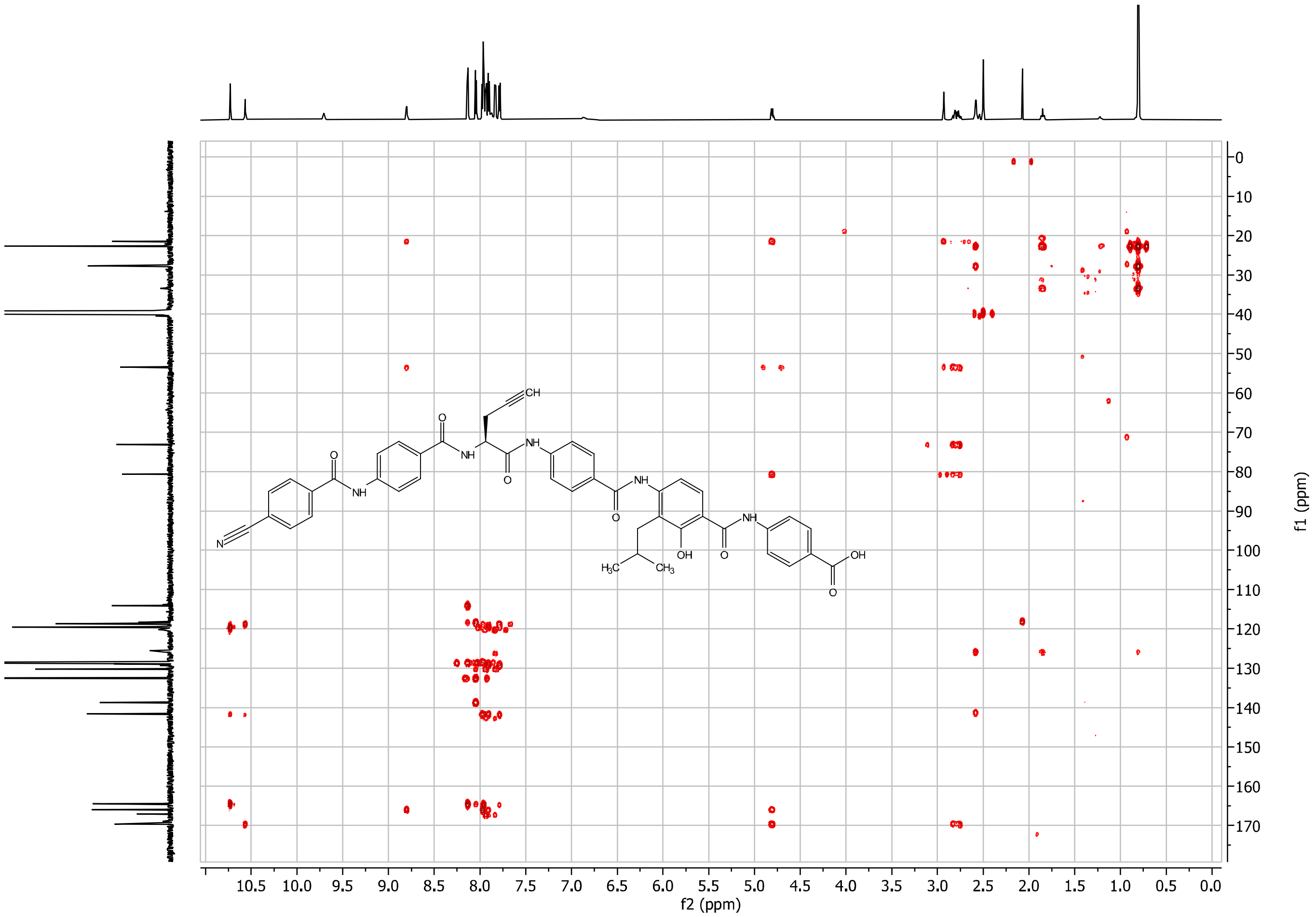
f2 (ppm)
f1 (ppm)

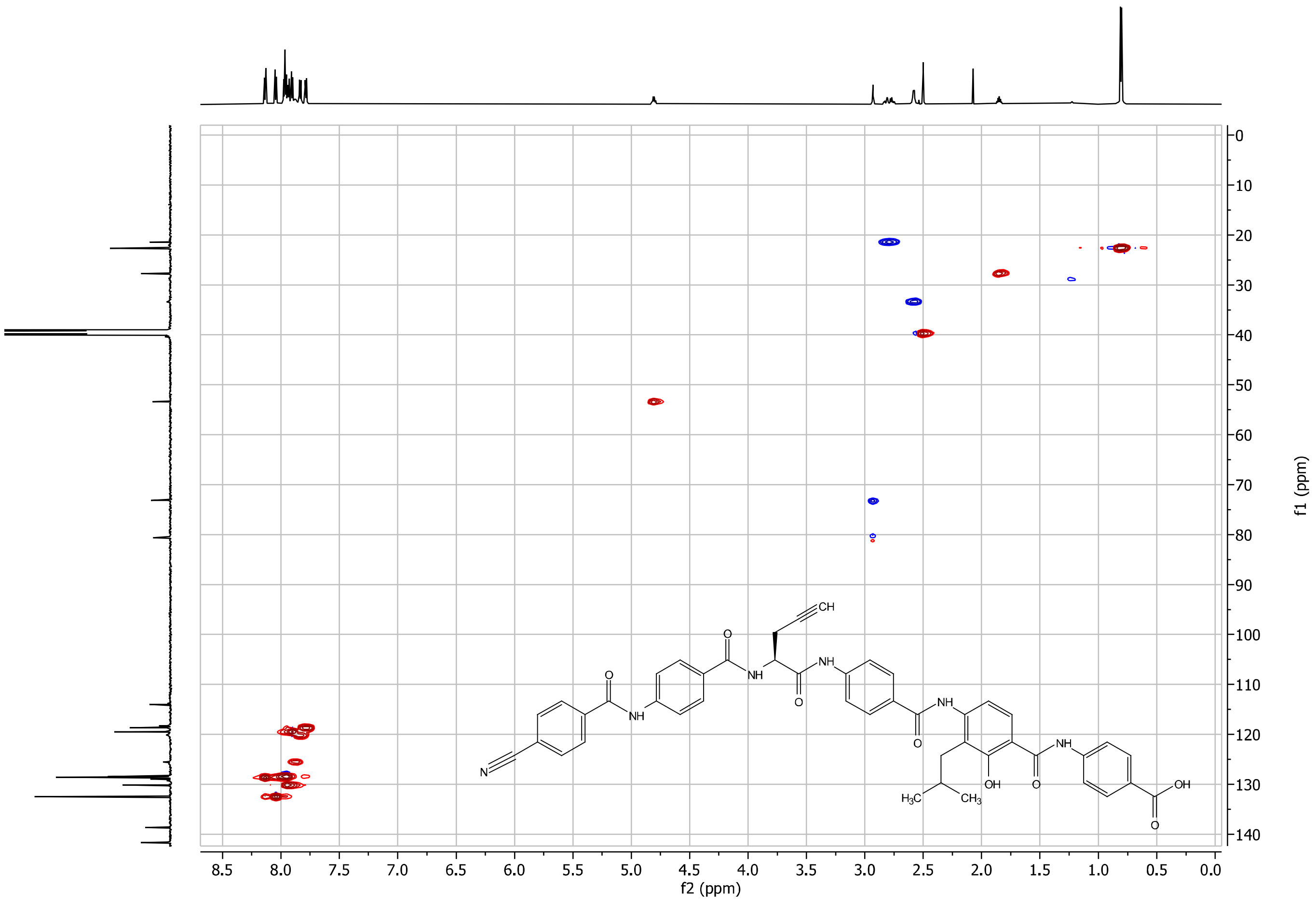
f2 (ppm)
f1 (ppm)

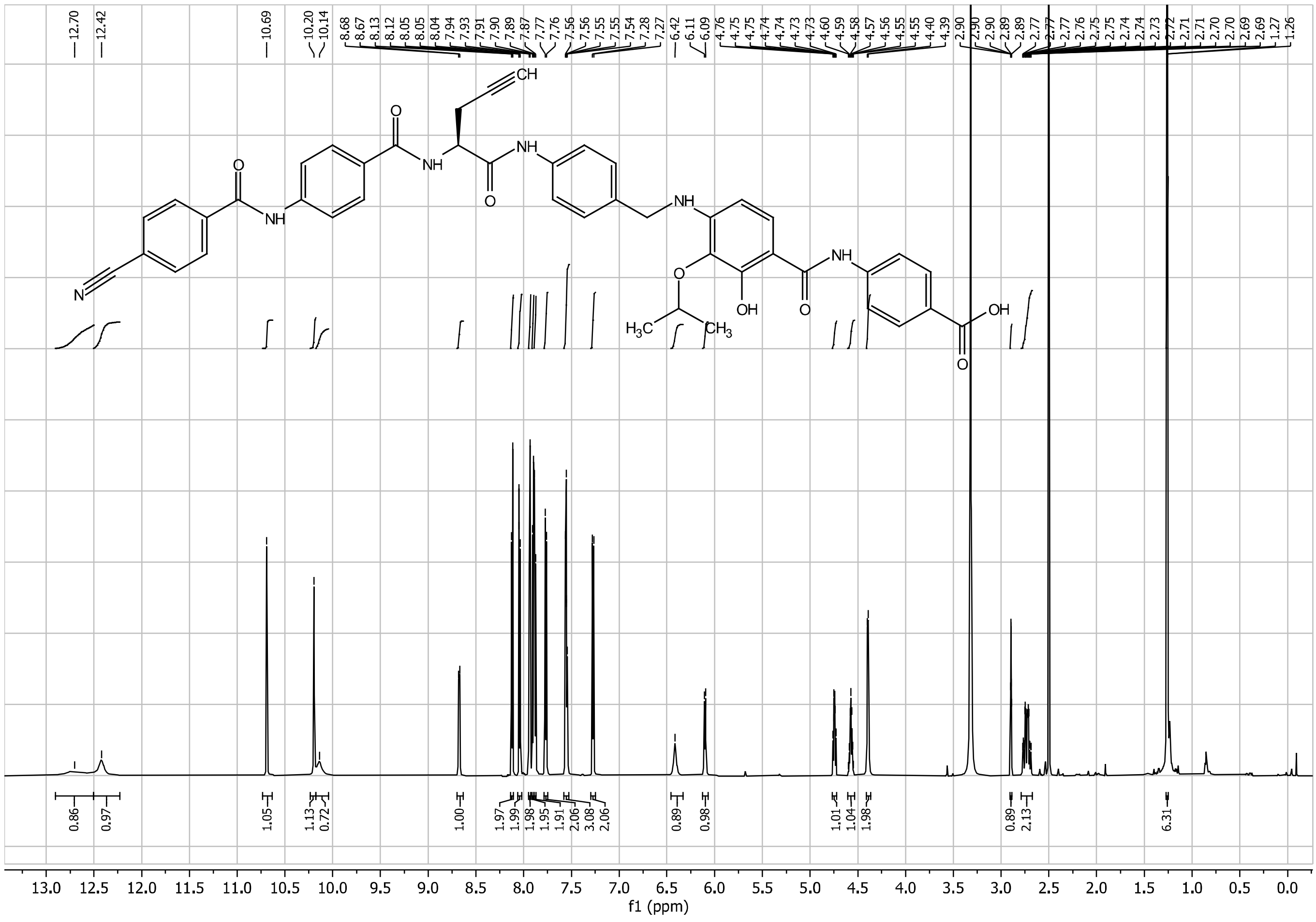
f1 (ppm)

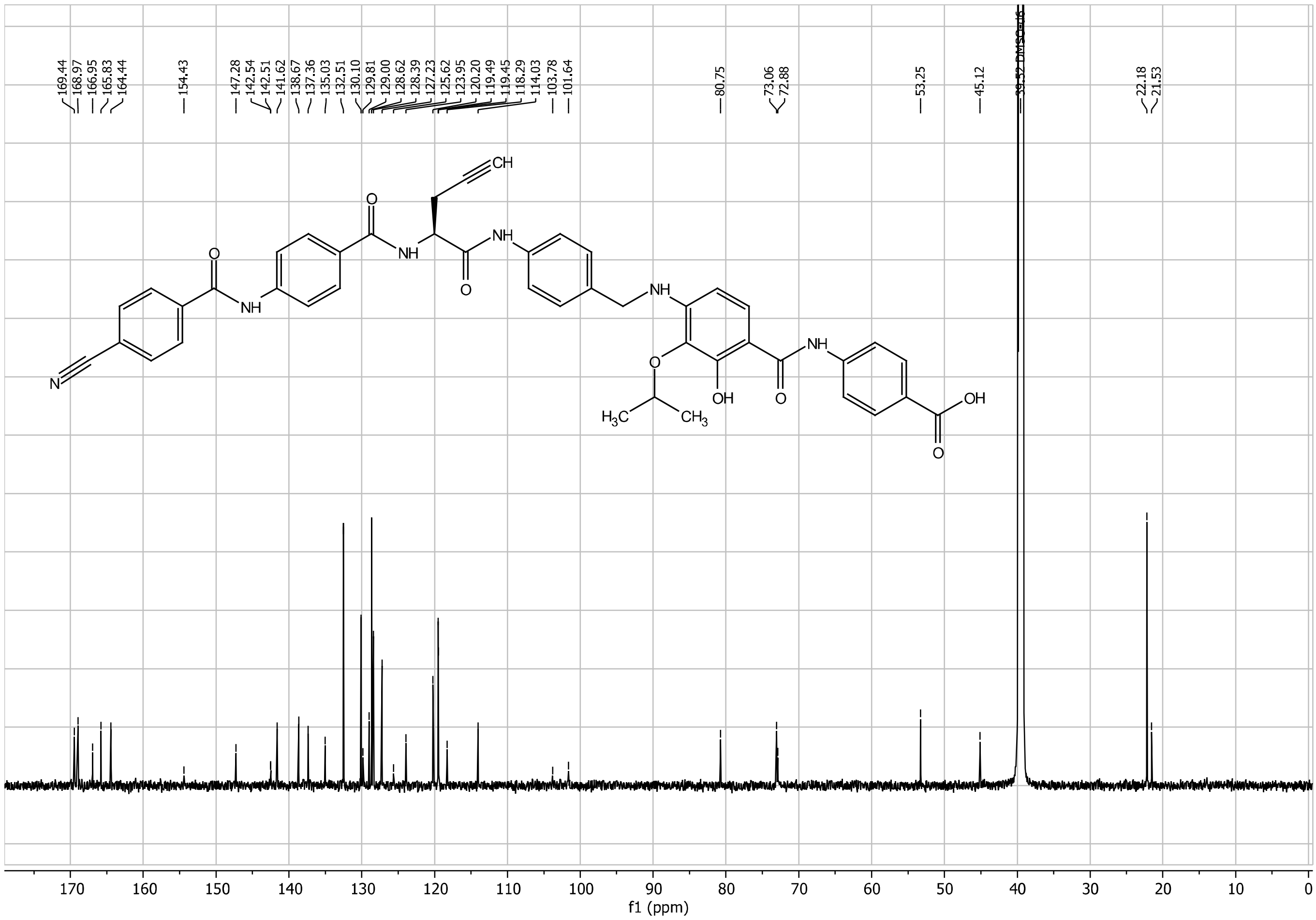
169.44
168.97
166.95
165.83
164.44
154.43
147.28
142.54
142.51
141.62
138.67
137.36
135.03
132.51
130.10
129.81
129.00
128.62
128.39
127.23
125.62
123.95
120.20
119.49
119.45
118.29
114.03
103.78
101.64
80.75
73.06
72.88
53.25
45.12
22.18
21.53
CH
O
NH
NH
O
NH
O
NH
N
NH
O
OH
O
H3C
CH3
NH
OH
O
f1 (ppm)

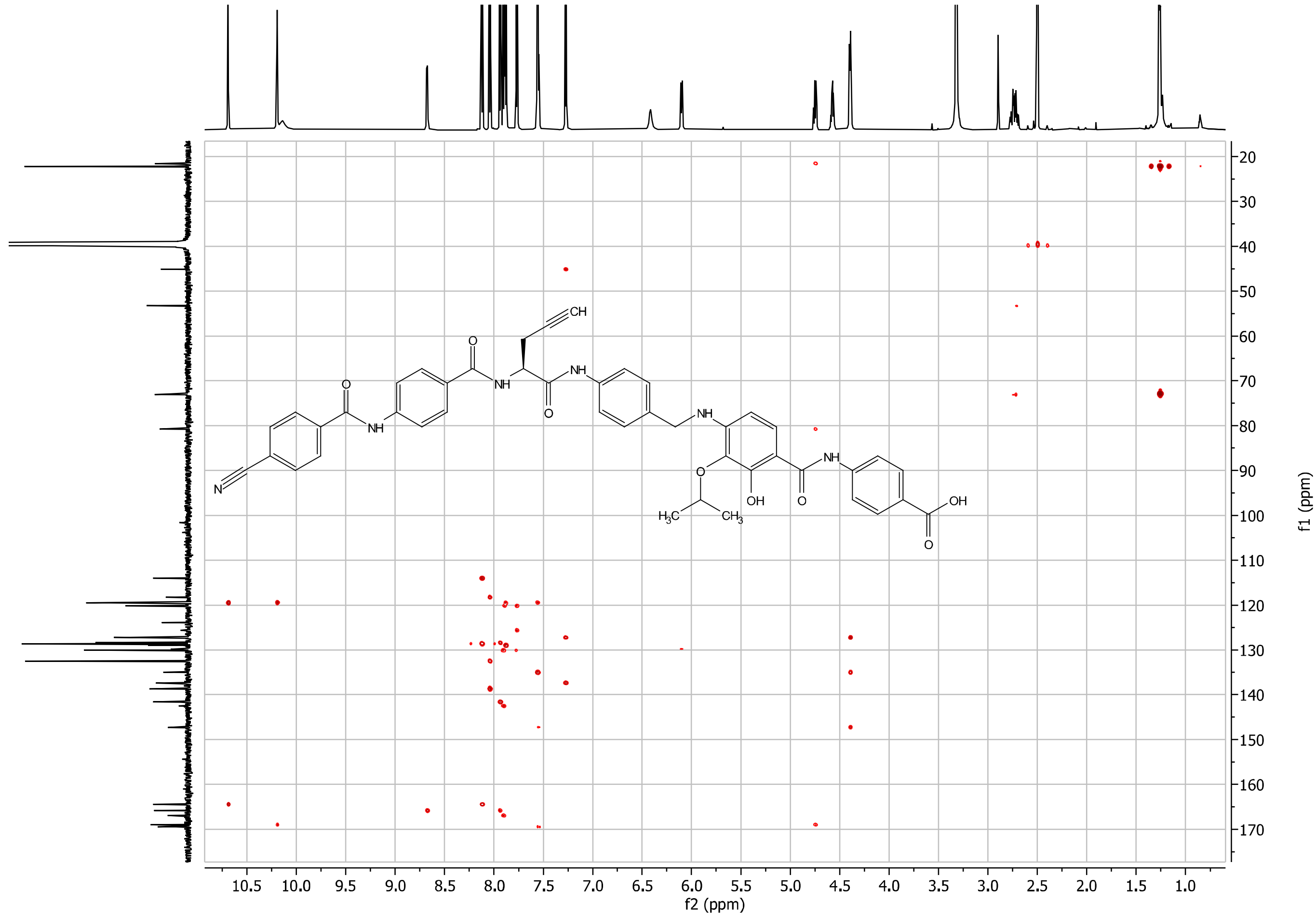
CH
O
NH
NH
O
NH
O
NH
N
NH
O
OH
O
H3C
CH3
NH
OH
O
f2 (ppm)
f1 (ppm)

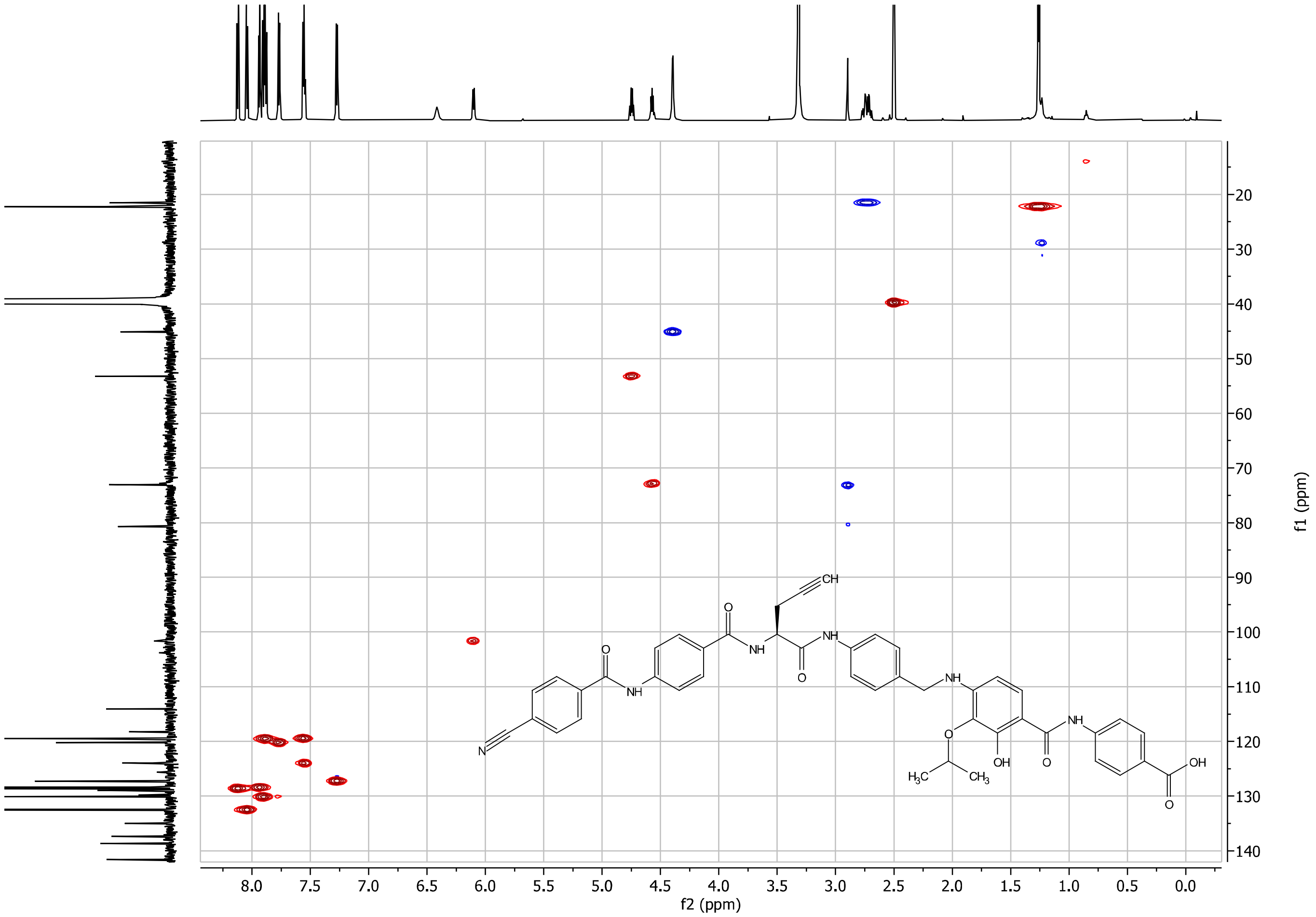
f2 (ppm)
f1 (ppm)
CH
O
NH
O
NH
O
NH
NH
N
O
OH
O
NH
H3C
CH3
OH
O

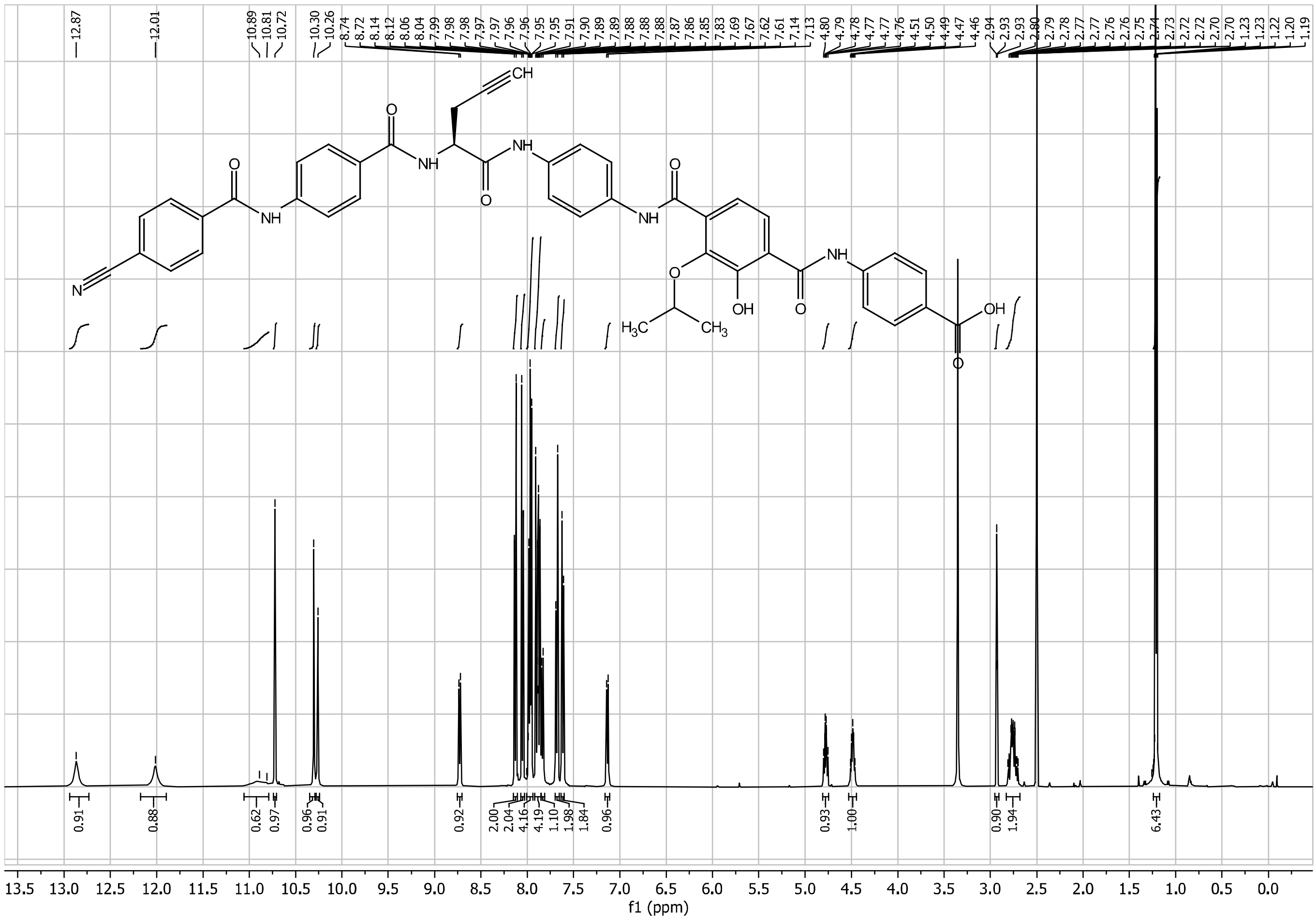
12.87
12.01
10.89
10.81
10.72
10.30
10.26
8.74
8.72
8.14
8.12
8.06
8.04
7.99
7.98
7.98
7.97
7.97
7.96
7.96
7.95
7.95
7.91
7.90
7.89
7.89
7.88
7.88
7.88
7.87
7.86
7.85
7.83
7.69
7.67
7.62
7.61
7.14
7.13
4.80
4.79
4.78
4.77
4.77
4.76
4.51
4.50
4.49
4.47
4.46
2.94
2.93
2.93
2.80
2.79
2.78
2.77
2.77
2.76
2.76
2.75
2.74
2.73
2.72
2.72
2.72
2.70
1.23
1.23
1.22
1.20
1.19
0.91
0.88
0.62
0.97
0.96
0.91
0.92
2.00
2.04
4.16
4.19
1.10
1.98
1.84
0.96
0.93
1.00
0.90
1.94
6.43
f1 (ppm)

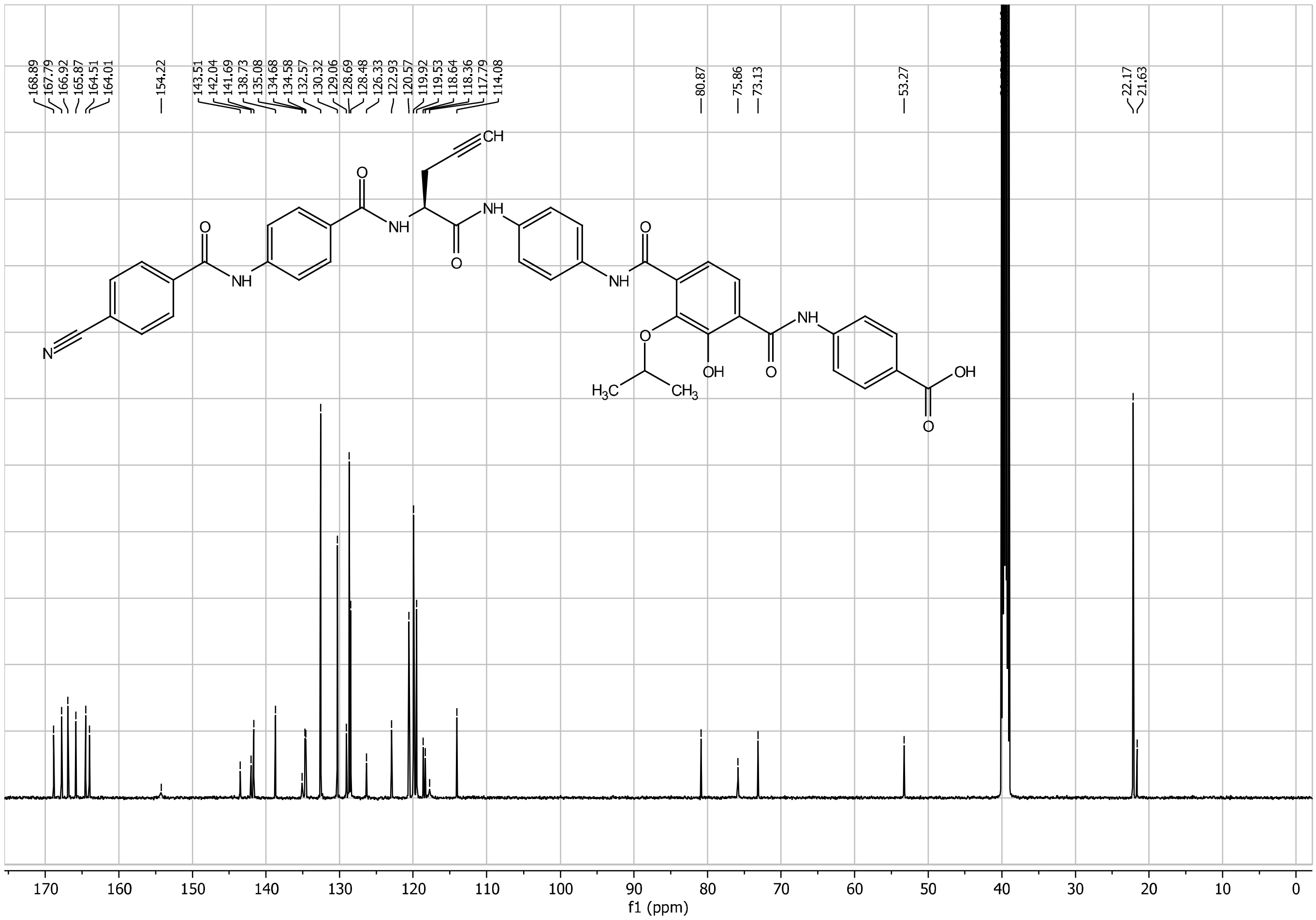
168.89
167.79
166.92
165.87
164.51
164.01
154.22
143.51
142.04
141.69
138.73
135.08
134.68
134.58
132.57
130.32
129.06
128.69
128.48
126.33
122.93
120.57
119.92
119.53
118.64
118.36
117.79
114.08
80.87
75.86
73.13
53.27
22.17
21.63
f1 (ppm)

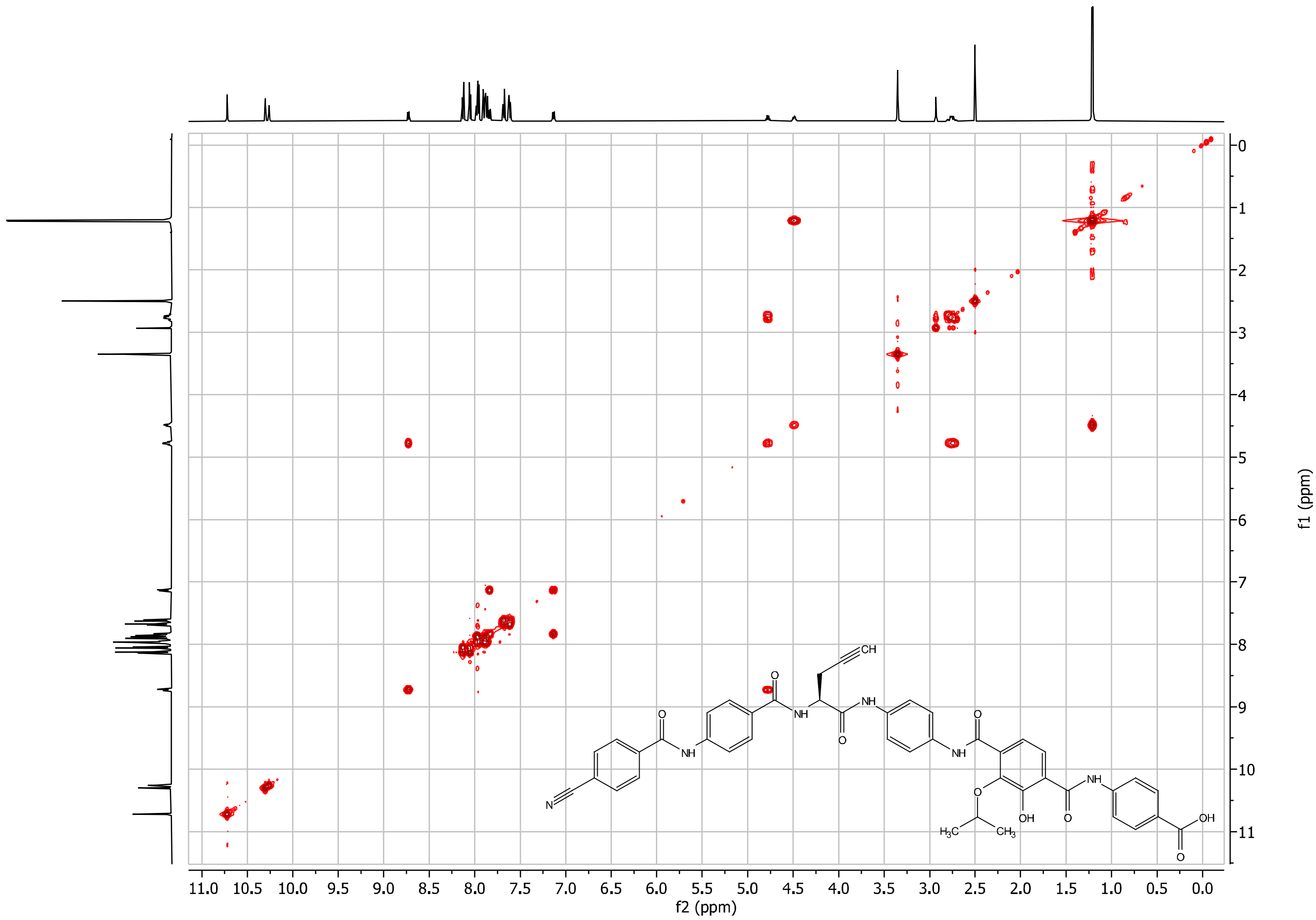
f2 (ppm)
f1 (ppm)

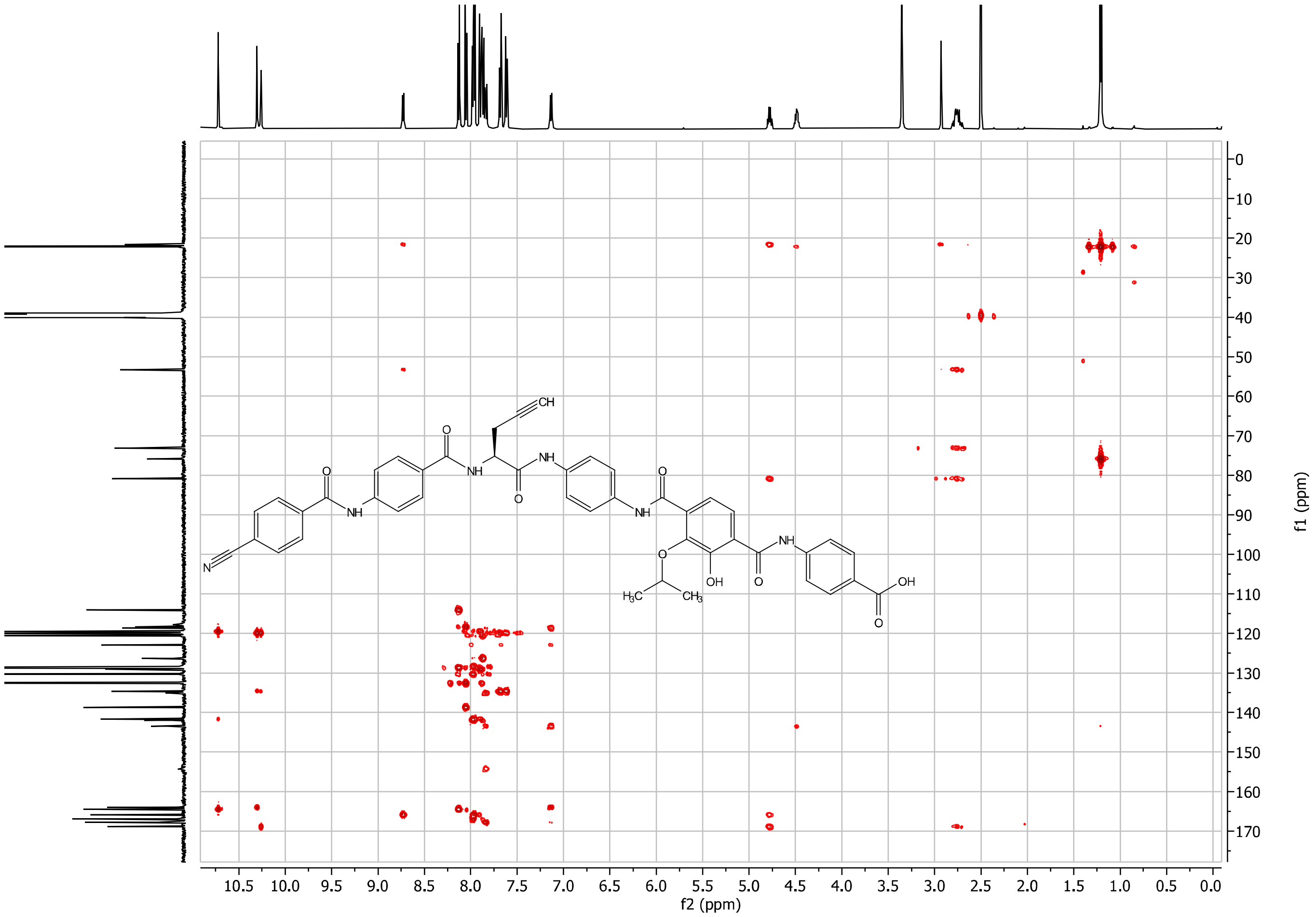

f1 (ppm)
f2 (ppm)
CH
O
NH
NH
O
NH
N
O
NH
O
OH
O
NH
H3C
CH3
OH
O

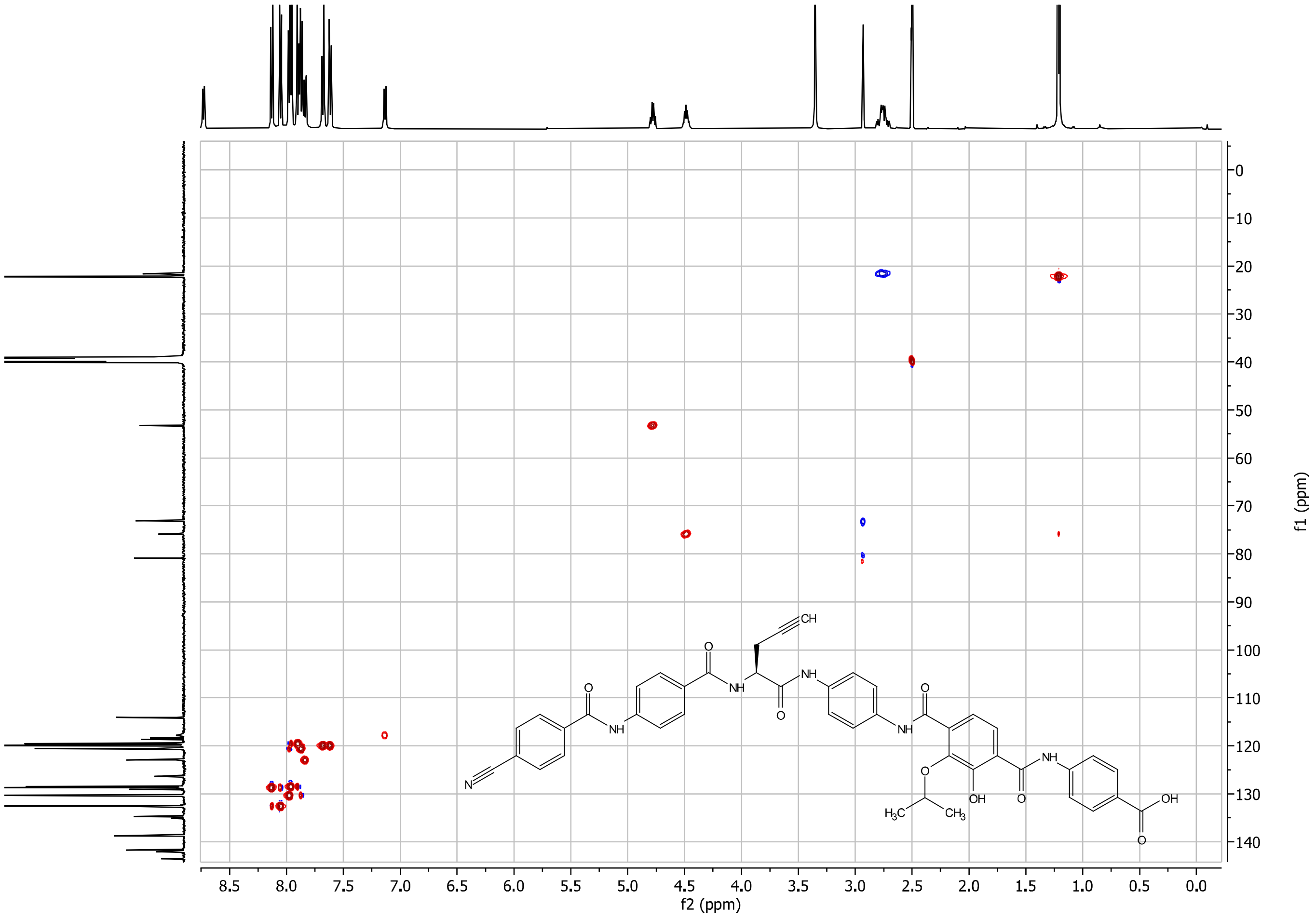

f1 (ppm)
f2 (ppm)
CH
O
NH
NH
O
NH
N
O
NH
O
OH
O
NH
H3C
CH3
OH
O

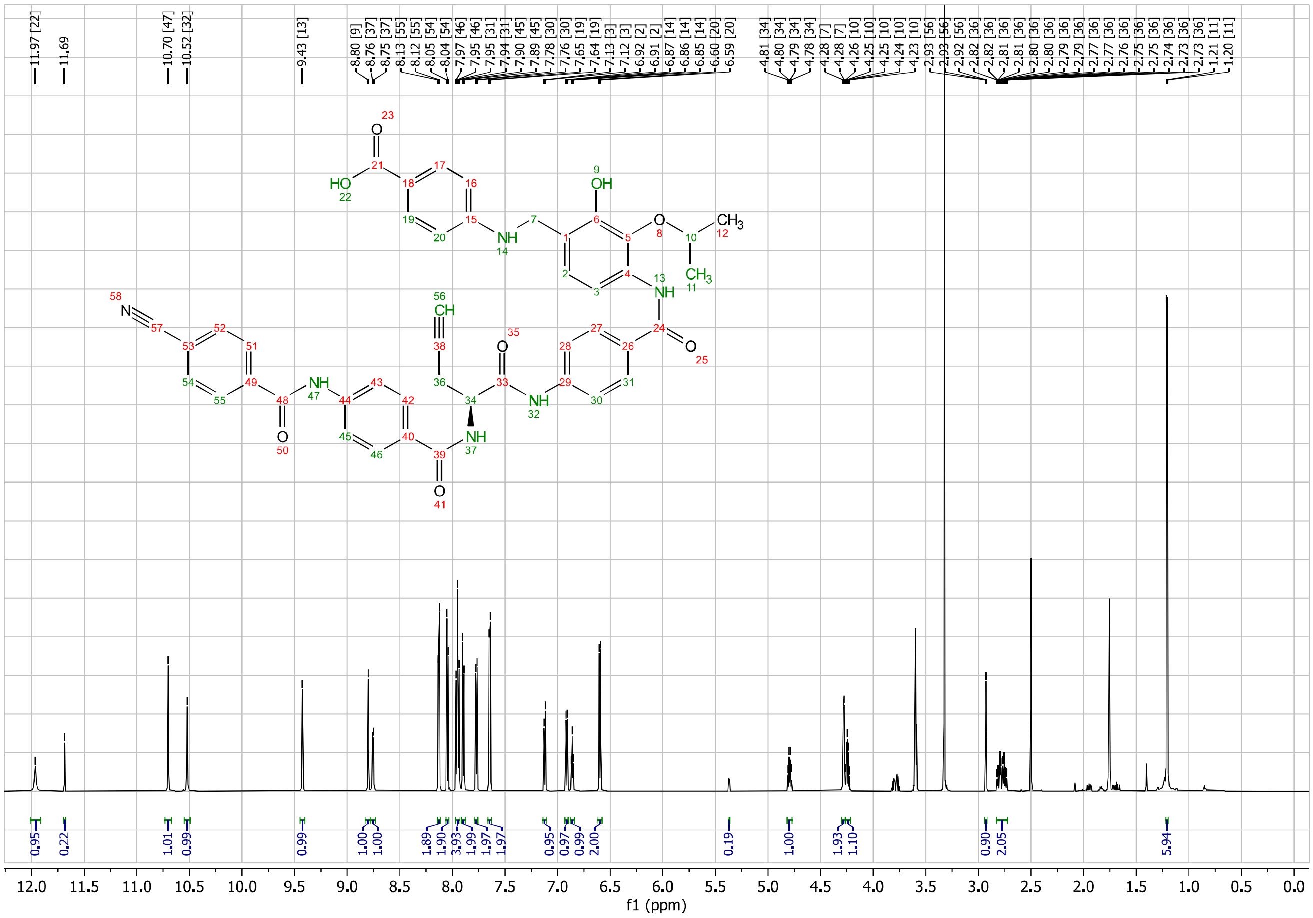
11.97 [22]
11.69
10.70 [47]
10.52 [32]
9.43 [13]
8.80 [9]
8.76 [37]
8.75 [37]
8.13 [55]
8.12 [55]
8.05 [54]
8.04 [54]
7.97 [46]
7.95 [46]
7.95 [31]
7.94 [31]
7.90 [45]
7.89 [45]
7.78 [30]
7.76 [30]
7.65 [19]
7.64 [19]
7.13 [3]
7.12 [3]
6.92 [2]
6.91 [2]
6.87 [14]
6.86 [14]
6.85 [14]
6.60 [20]
6.59 [20]
4.81 [34]
4.80 [34]
4.79 [34]
4.78 [34]
4.28 [7]
4.28 [7]
4.26 [10]
4.25 [10]
4.25 [10]
4.24 [10]
4.23 [10]
2.93 [56]
2.93 [56]
2.92 [56]
2.82 [36]
2.82 [36]
2.81 [36]
2.81 [36]
2.80 [36]
2.80 [36]
2.79 [36]
2.79 [36]
2.77 [36]
2.77 [36]
2.76 [36]
2.75 [36]
2.75 [36]
2.74 [36]
2.73 [36]
2.73 [36]
1.21 [11]
1.20 [11]
0.95
0.22
1.01
0.99
0.99
1.00
1.00
1.89
1.90
3.93
1.99
1.97
1.97
0.95
0.97
0.99
2.00
0.19
1.00
1.93
1.10
0.90
2.05
5.94
f1 (ppm)

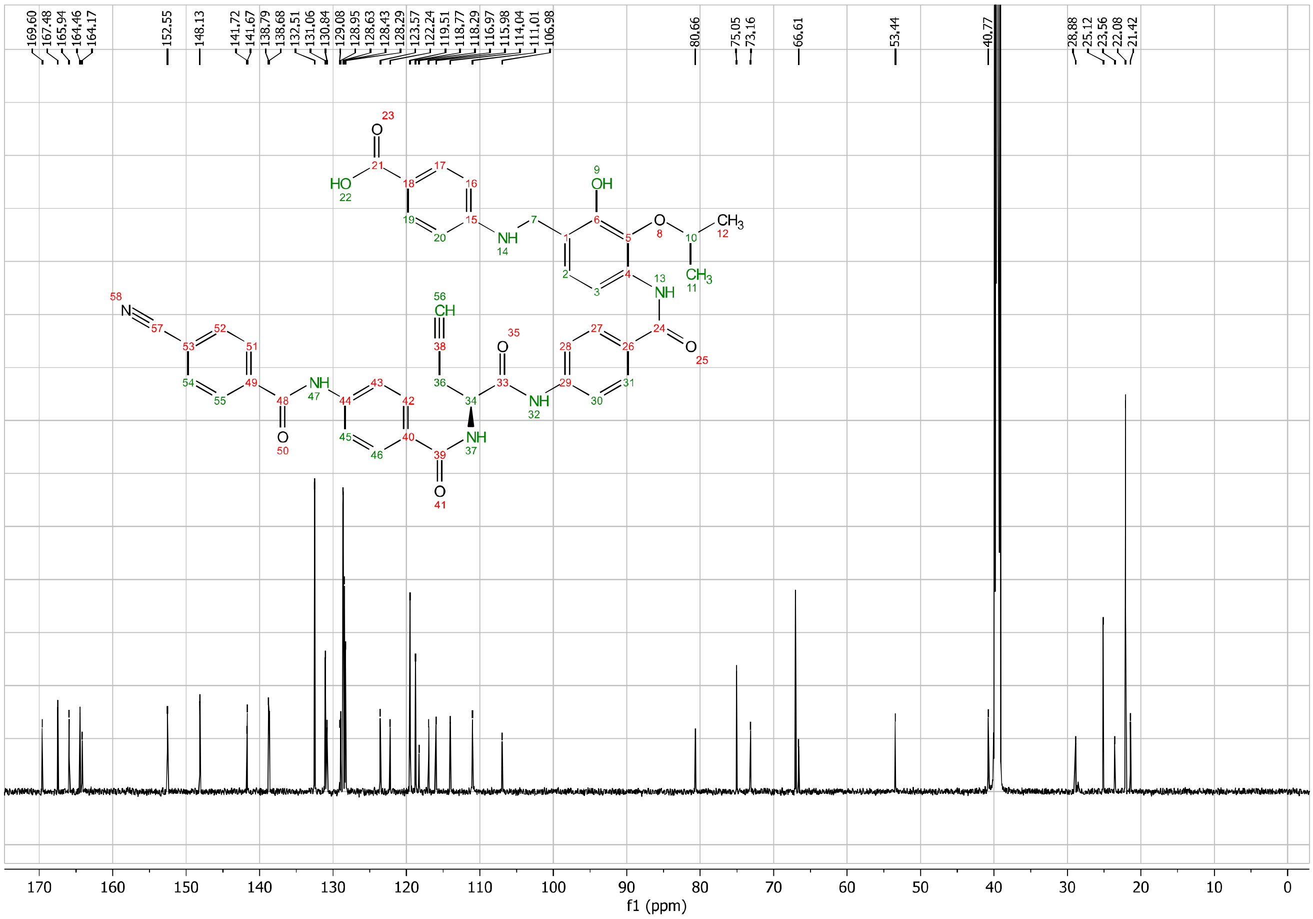
169.60
167.48
165.94
164.46
164.17
152.55
148.13
141.72
141.67
138.79
138.68
132.51
131.06
130.84
129.08
128.95
128.63
128.43
128.29
123.57
122.24
119.51
118.77
118.29
116.97
115.98
114.04
111.01
106.98
80.66
75.05
73.16
66.61
53.44
40.77
28.88
25.12
23.56
22.08
21.42
f1 (ppm)

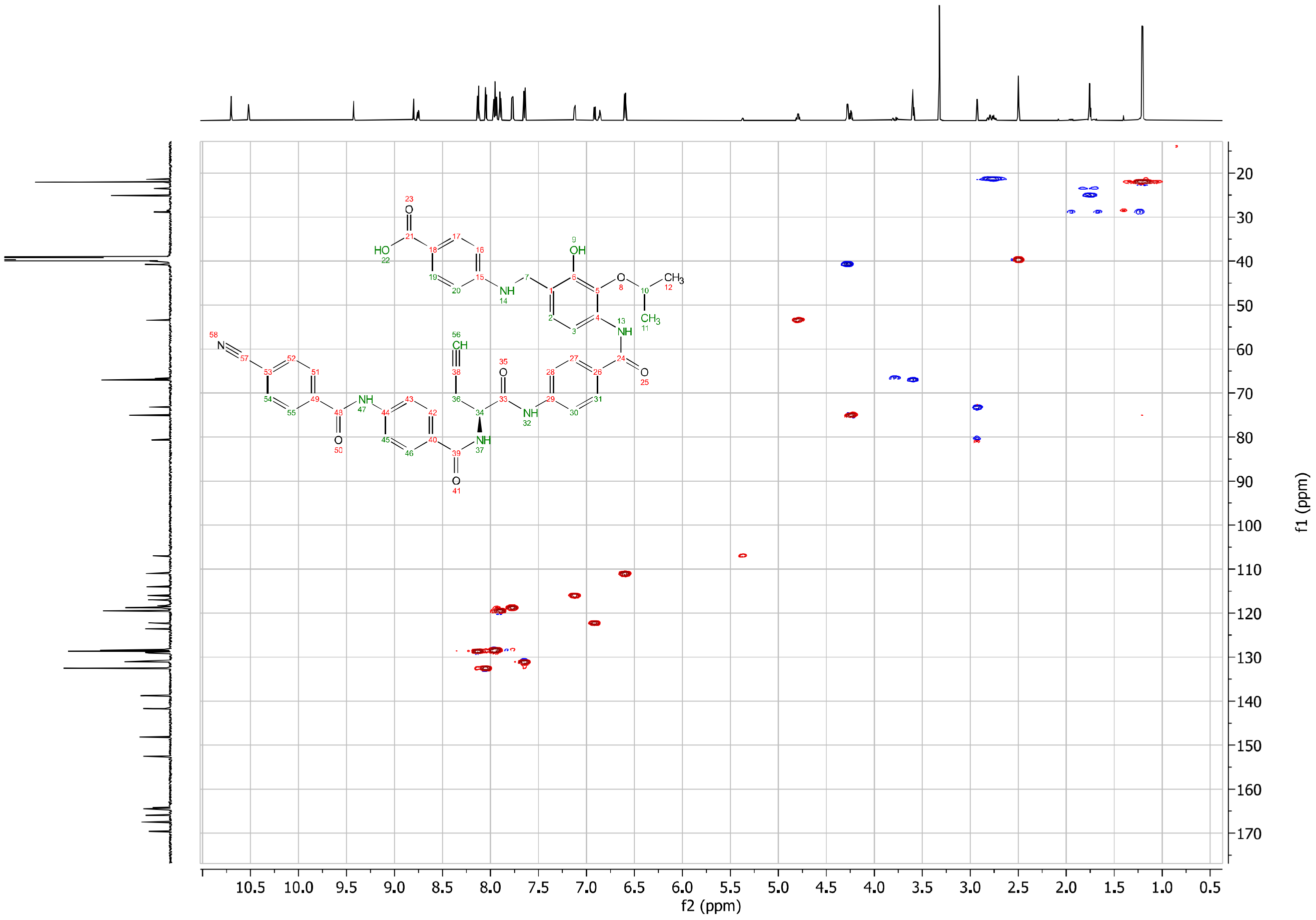
f2 (ppm)
f1 (ppm)

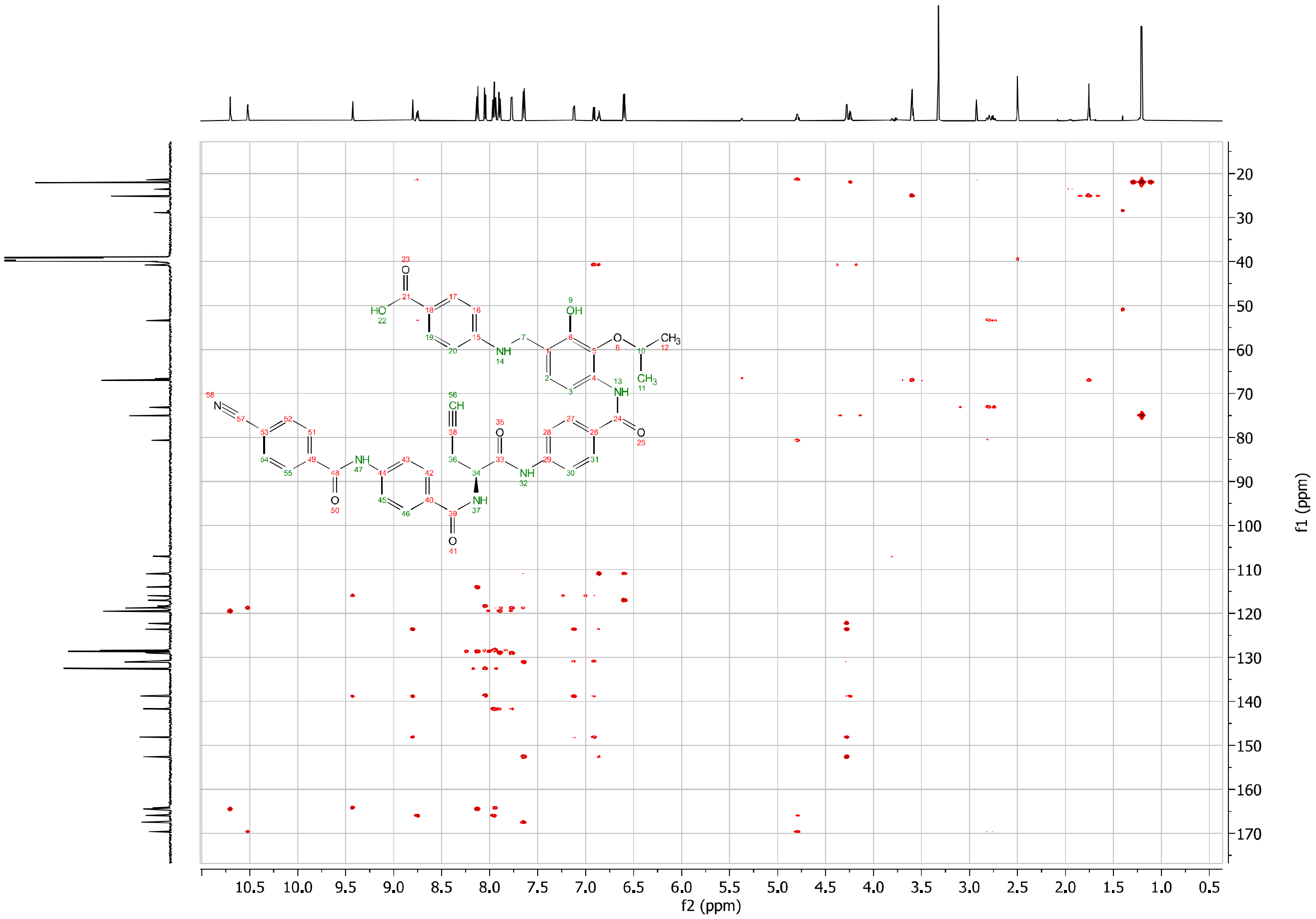
f2 (ppm)
f1 (ppm)

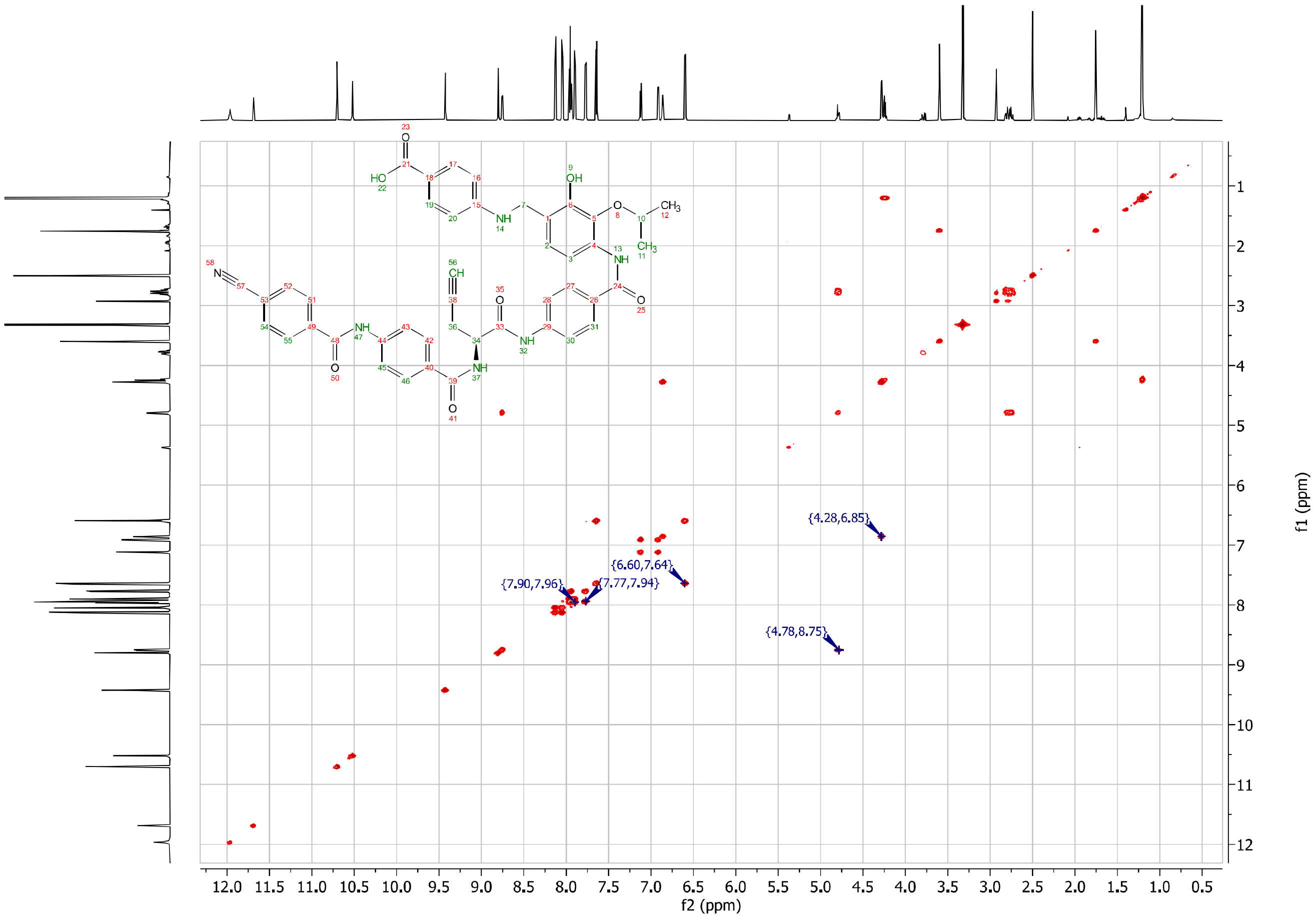
{4.28,6.85}
{6.60,7.64}
{7.90,7.96}
{7.77,7.94}
{4.78,8.75}
f2 (ppm)
f1 (ppm)

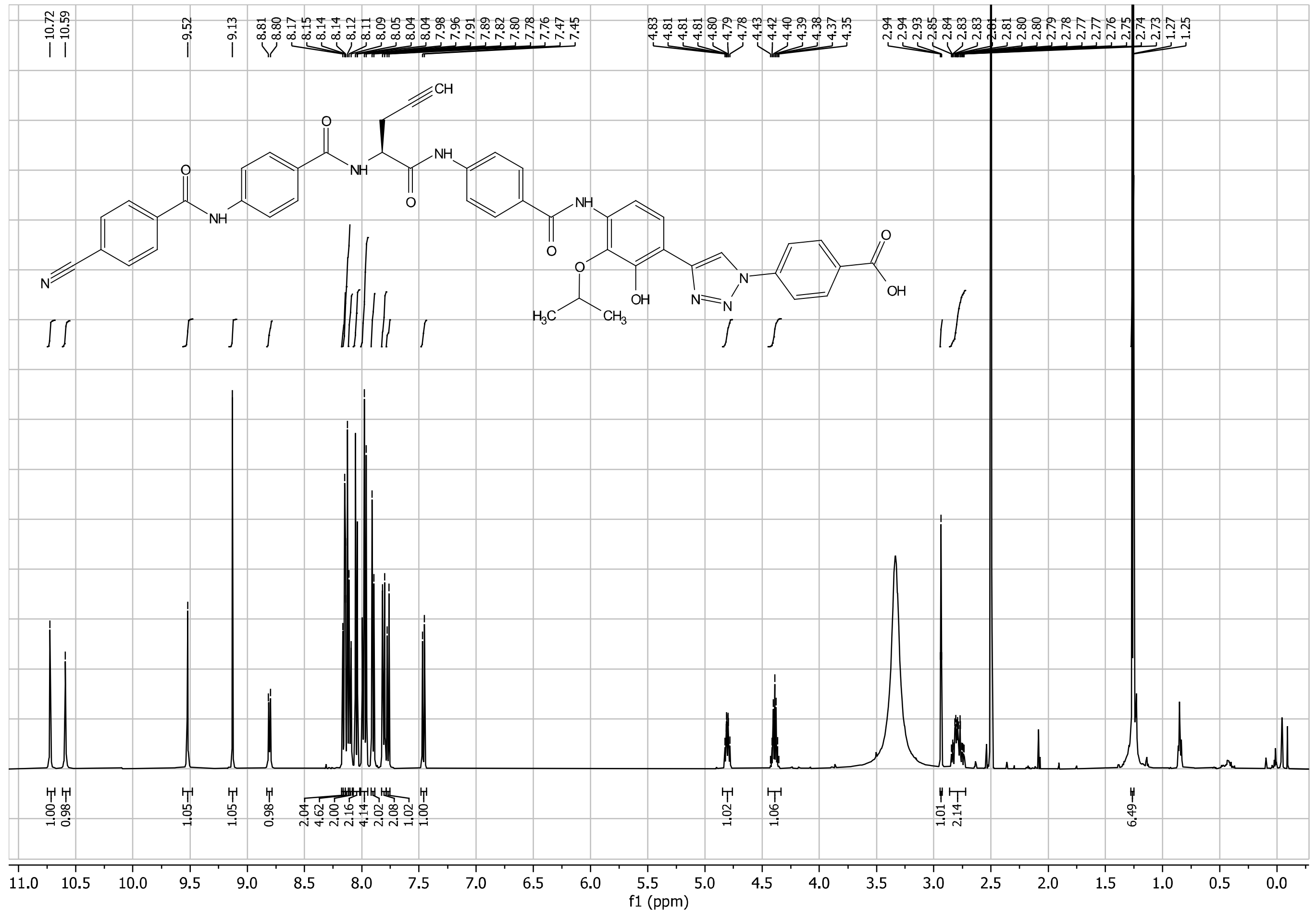

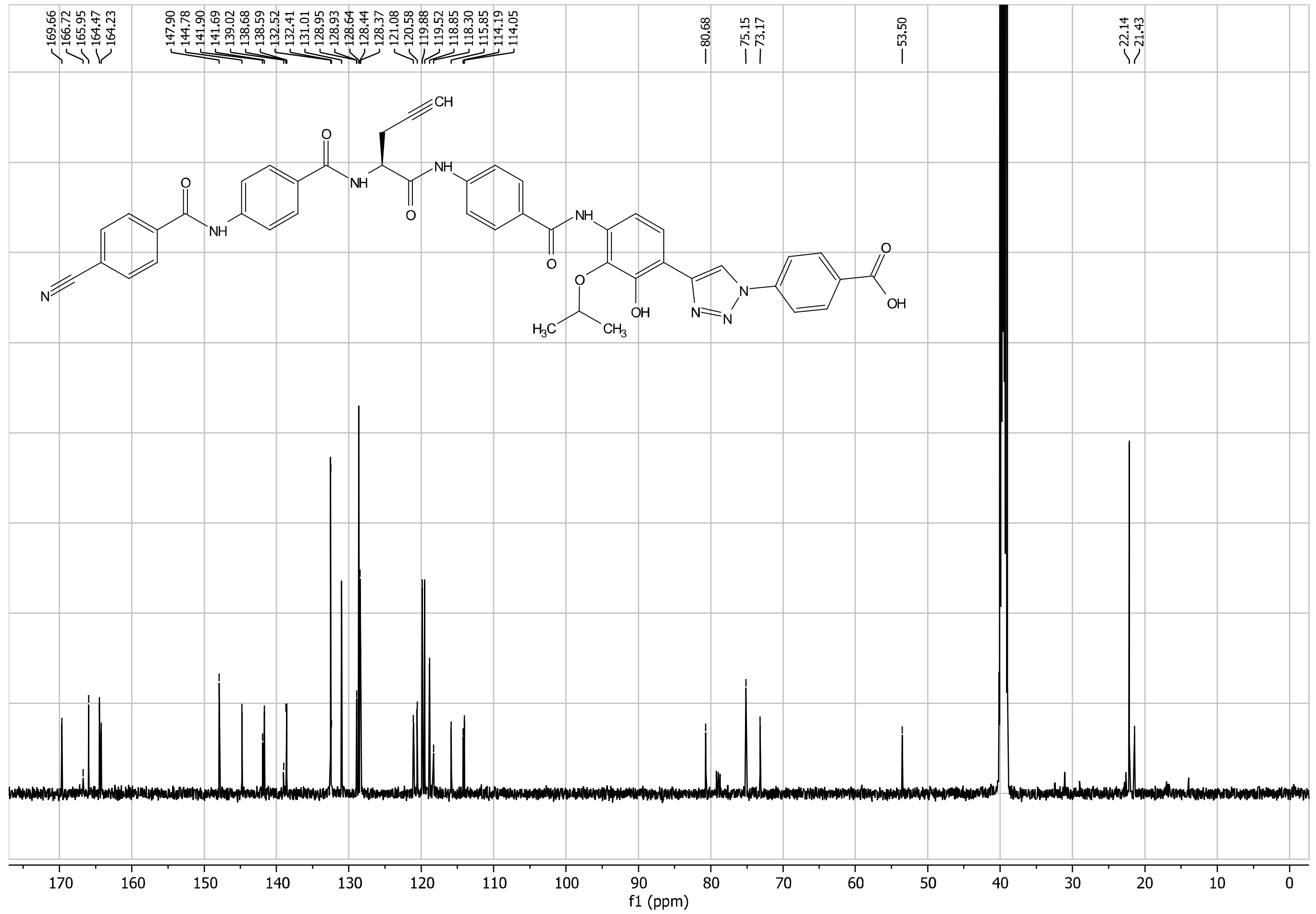

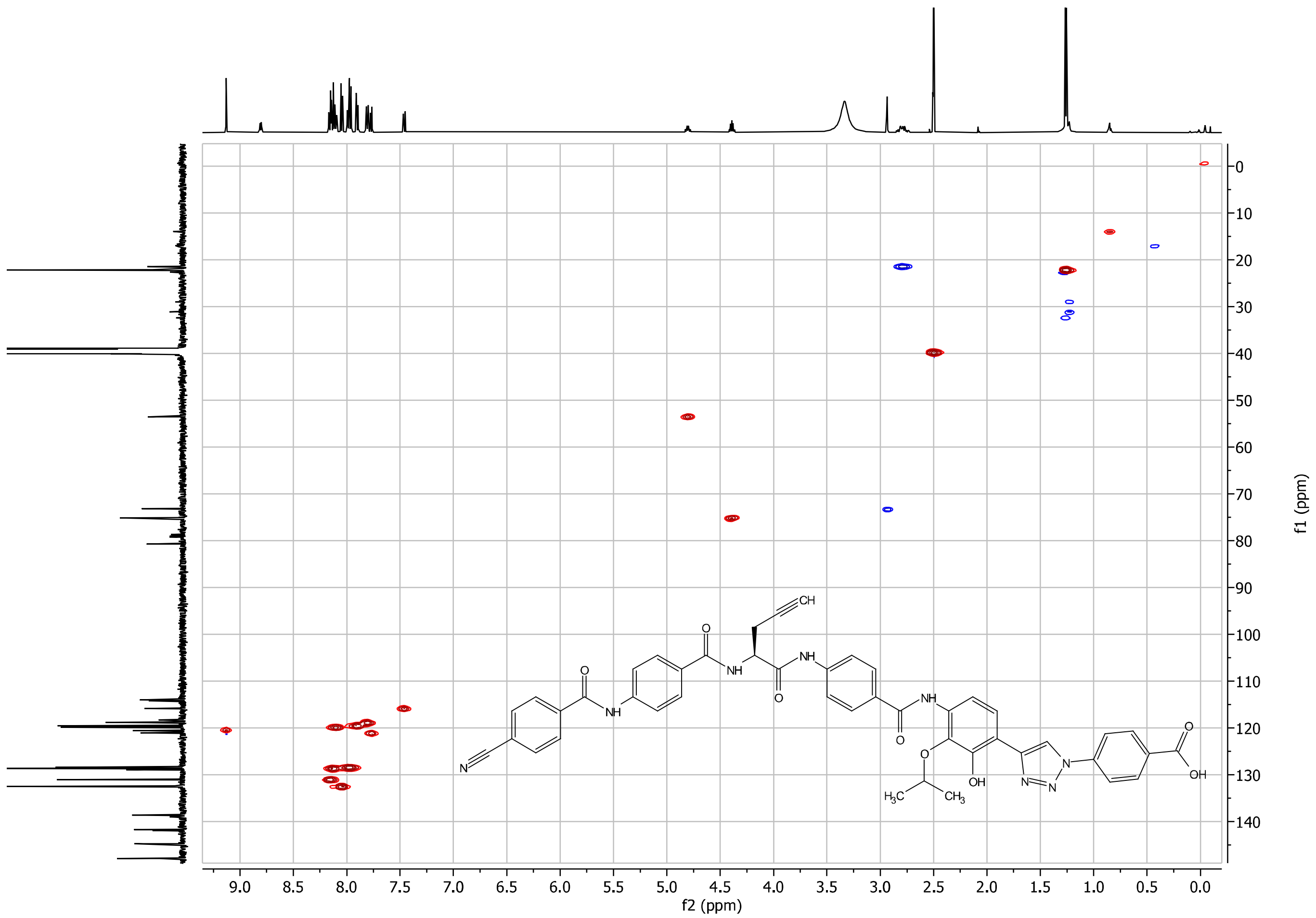
f1 (ppm)
f2 (ppm)
CH
O
NH
NH
O
NH
O
NH
O
N
O
OH
H3C
CH3
N
N
N
O
OH

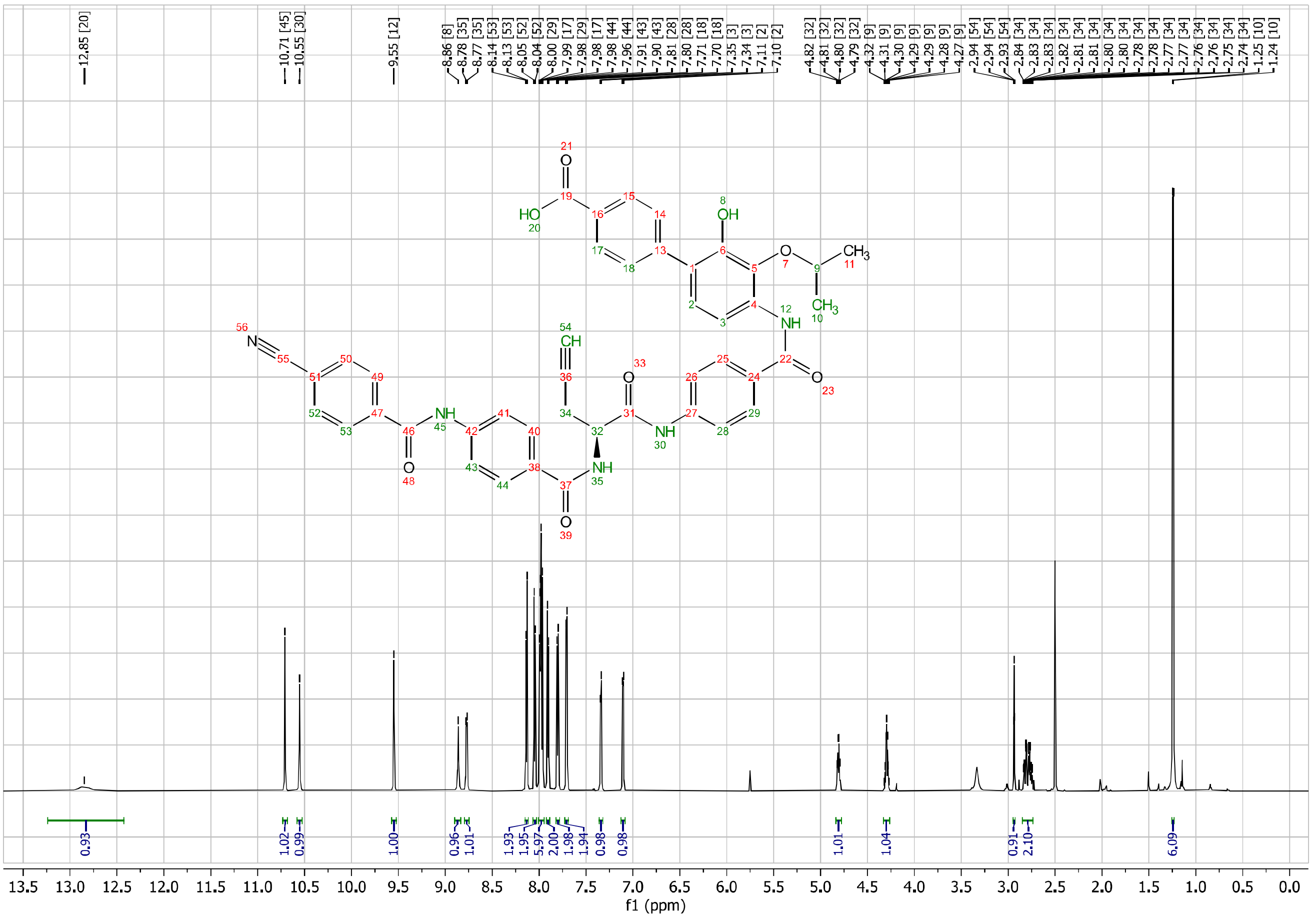
12.85 [20]
10.71 [45]
10.55 [30]
9.55 [12]
8.86 [8]
8.78 [35]
8.77 [35]
8.14 [53]
8.13 [53]
8.05 [52]
8.04 [52]
8.00 [29]
7.99 [17]
7.98 [29]
7.98 [17]
7.98 [44]
7.96 [44]
7.91 [43]
7.90 [43]
7.81 [28]
7.80 [28]
7.71 [18]
7.70 [18]
7.35 [3]
7.34 [3]
7.11 [2]
7.10 [2]
4.82 [32]
4.81 [32]
4.80 [32]
4.79 [32]
4.32 [9]
4.31 [9]
4.30 [9]
4.29 [9]
4.29 [9]
4.28 [9]
4.27 [9]
2.94 [54]
2.94 [54]
2.93 [54]
2.84 [34]
2.83 [34]
2.83 [34]
2.82 [34]
2.81 [34]
2.81 [34]
2.80 [34]
2.80 [34]
2.78 [34]
2.78 [34]
2.77 [34]
2.77 [34]
2.76 [34]
2.75 [34]
2.74 [34]
1.25 [10]
1.24 [10]
0.93
1.02
0.99
1.00
0.96
1.01
1.93
1.95
5.97
2.00
1.98
1.94
0.98
0.98
1.01
1.04
0.91
2.10
6.09
f1 (ppm)

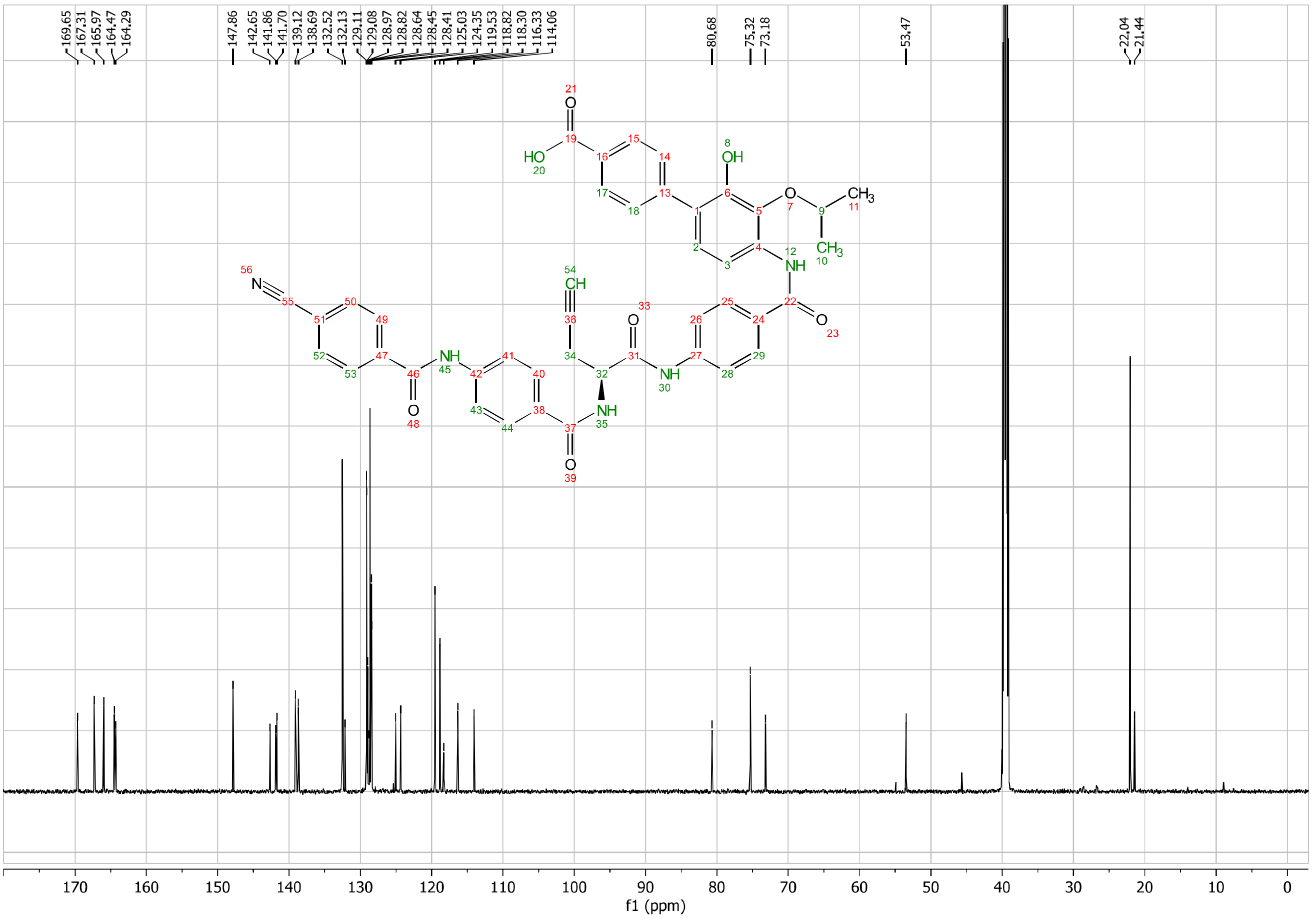
169.65
167.31
165.97
164.47
164.29
147.86
142.65
141.86
141.70
139.12
138.69
132.52
132.13
129.11
129.08
128.97
128.82
128.64
128.45
128.41
125.03
124.35
119.53
118.82
118.30
116.33
114.06
80.68
75.32
73.18
53.47
22.04
21.44
f1 (ppm)

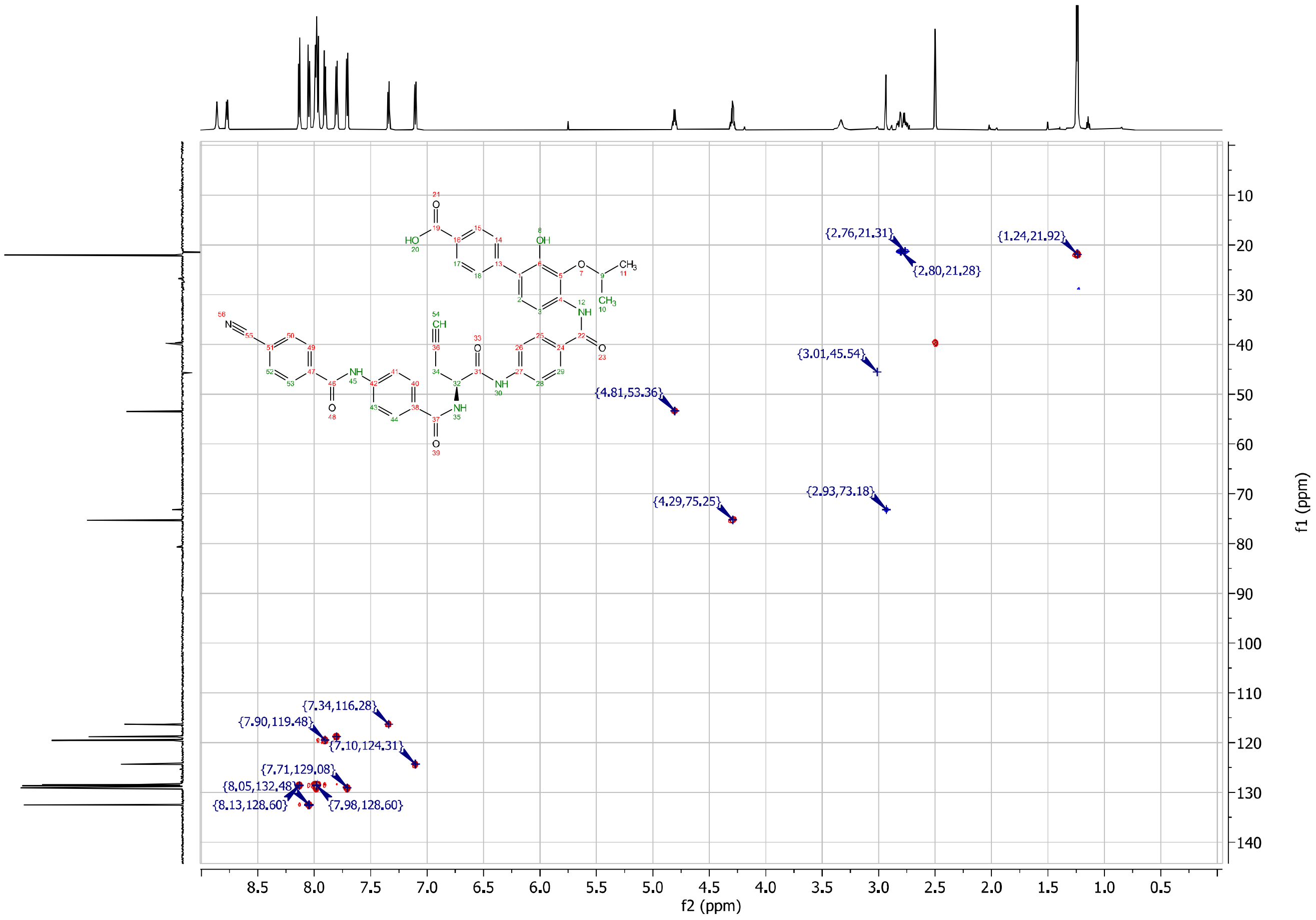
{2.76,21.31}
{1.24,21.92}
{2.80,21.28}
{3.01,45.54}
{4.81,53.36}
{4.29,75.25}
{2.93,73.18}
{7.34,116.28}
{7.90,119.48}
{7.10,124.31}
{7.71,129.08}
{8.05,132.48}
{8.13,128.60}
{7.98,128.60}
f2 (ppm)
f1 (ppm)

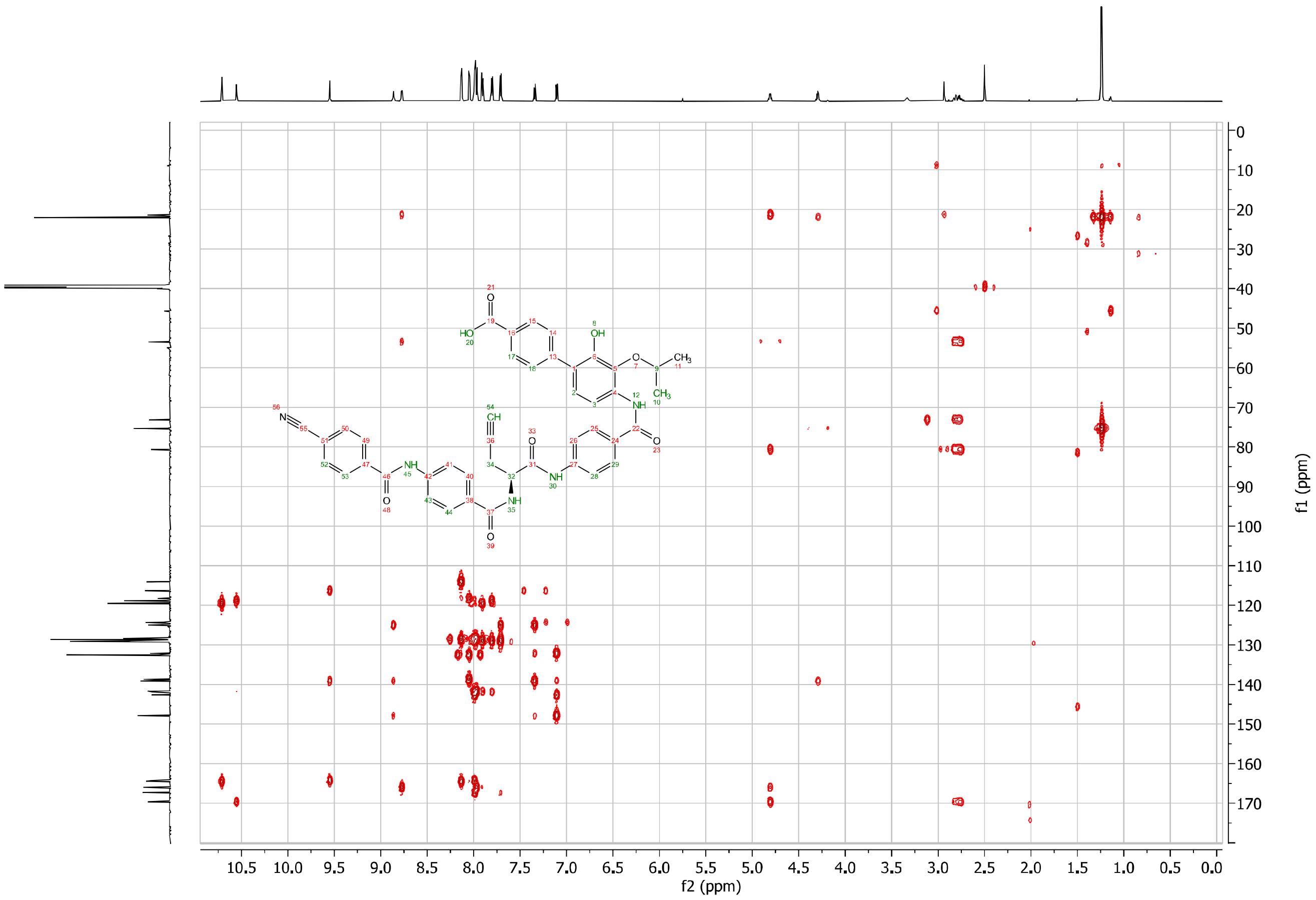
f2 (ppm)
f1 (ppm)

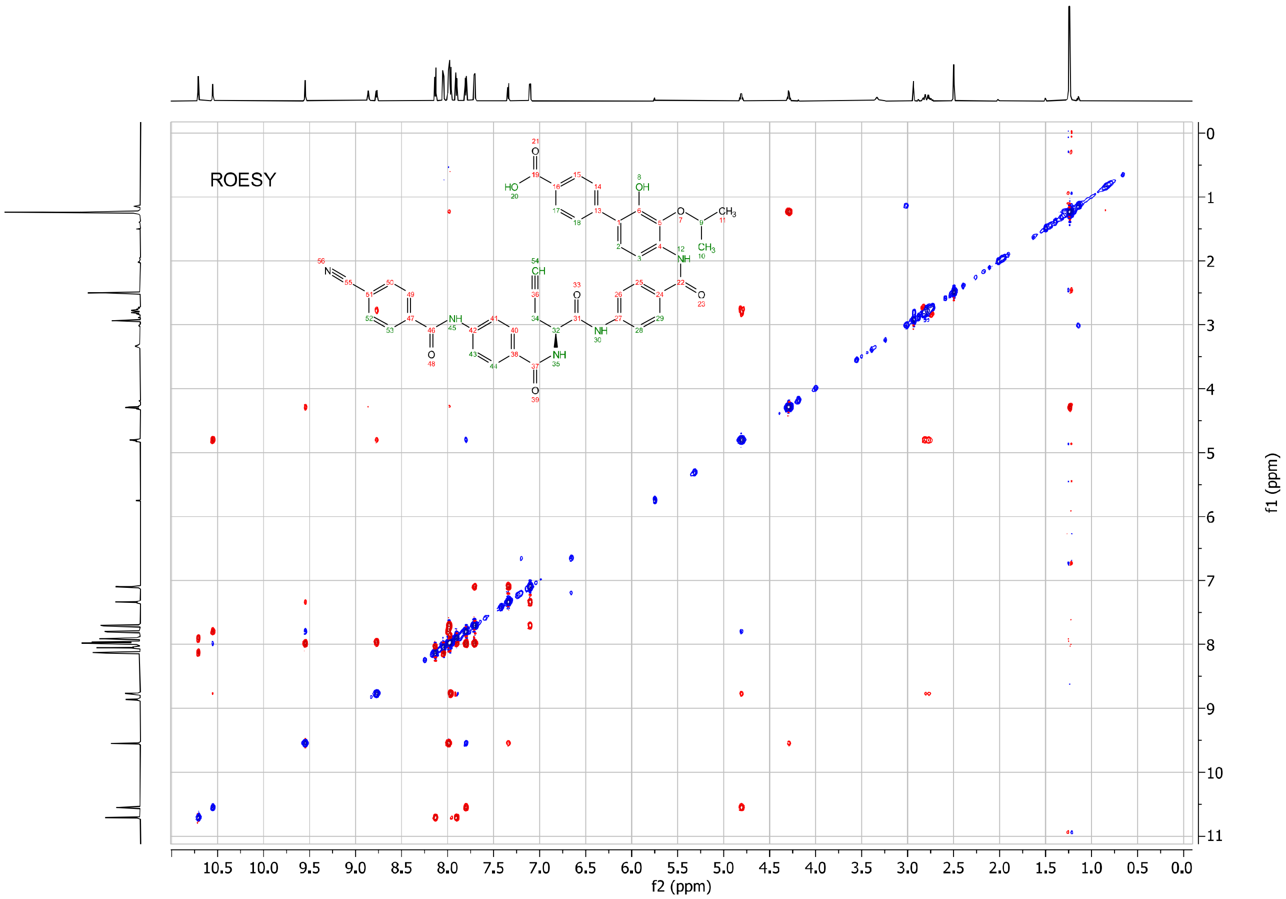

ROESY
f2 (ppm)
f1 (ppm)

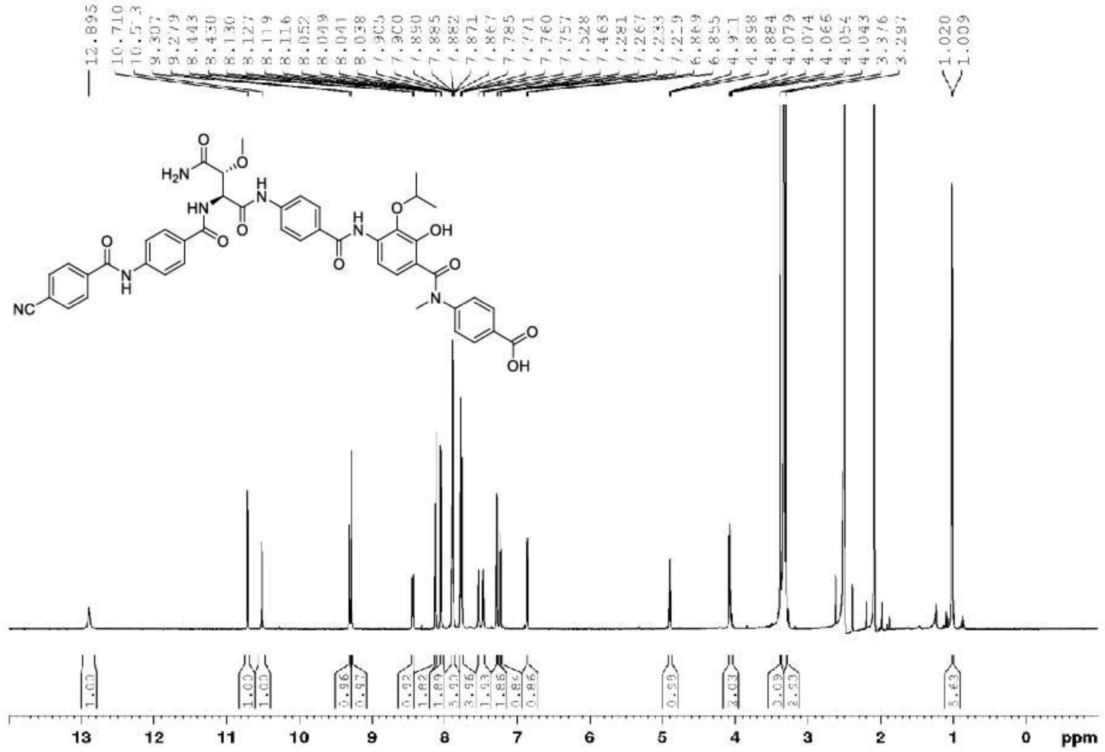

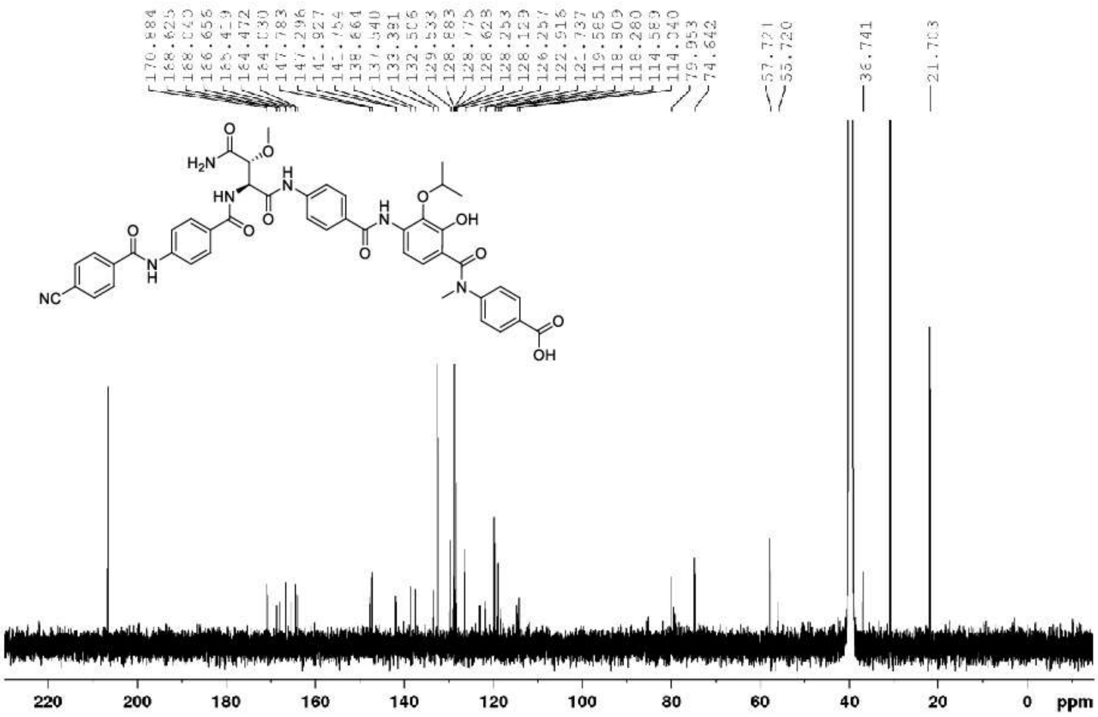
170.884
168.625
168.040
166.655
165.419
164.472
164.030
147.783
147.296
141.927
141.754
138.664
137.540
133.391
132.506
129.533
128.883
128.775
128.623
128.253
128.129
126.257
122.916
121.737
119.585
118.809
118.280
114.589
114.340
79.953
74.642
57.721
55.720
36.741
21.703
H2N
O
HN
O
NH
O
N
H
O
NC
NH
O
OH
O
N
O
OH
220
200
180
160
140
120
100
80
60
40
20
0
ppm

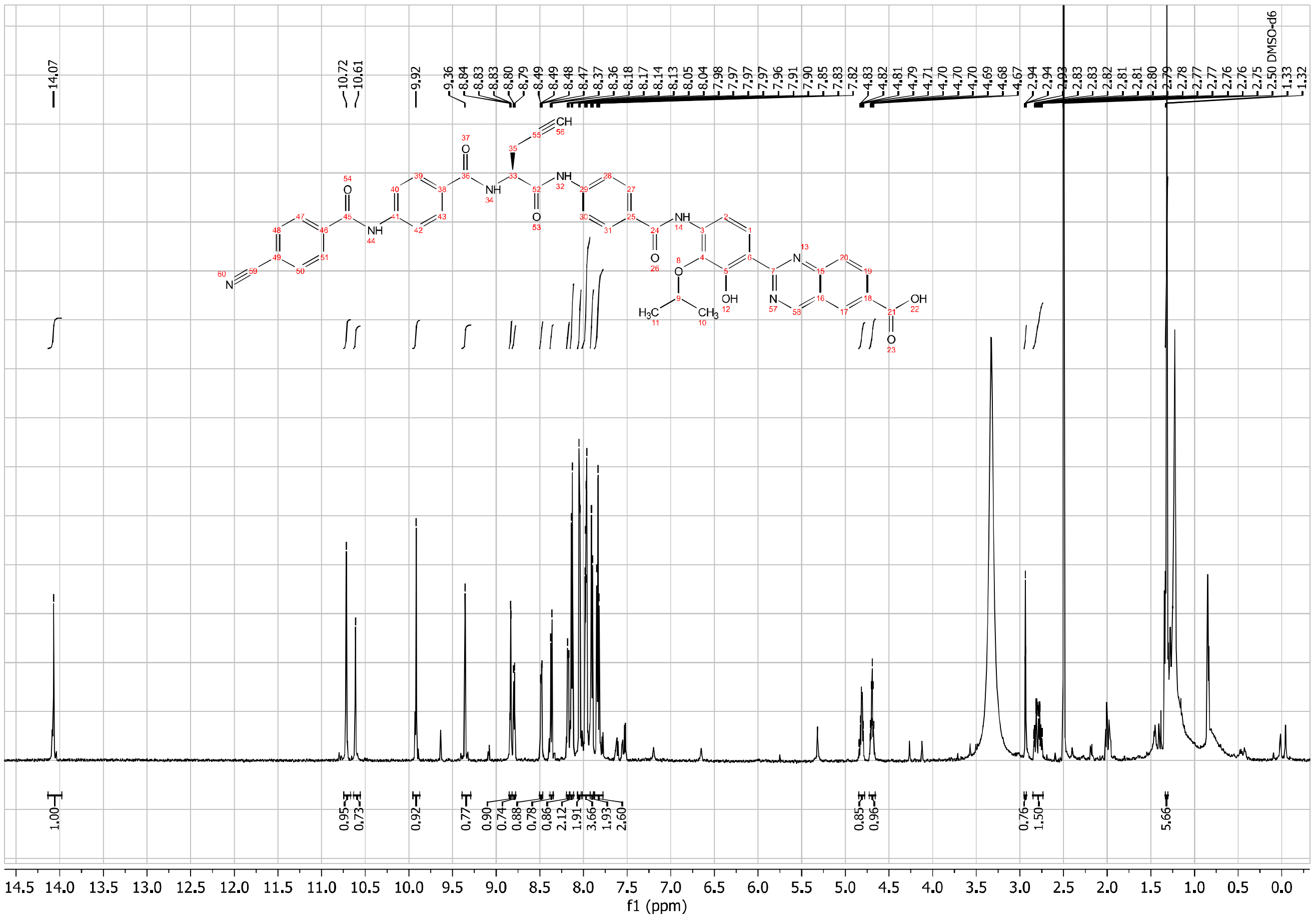

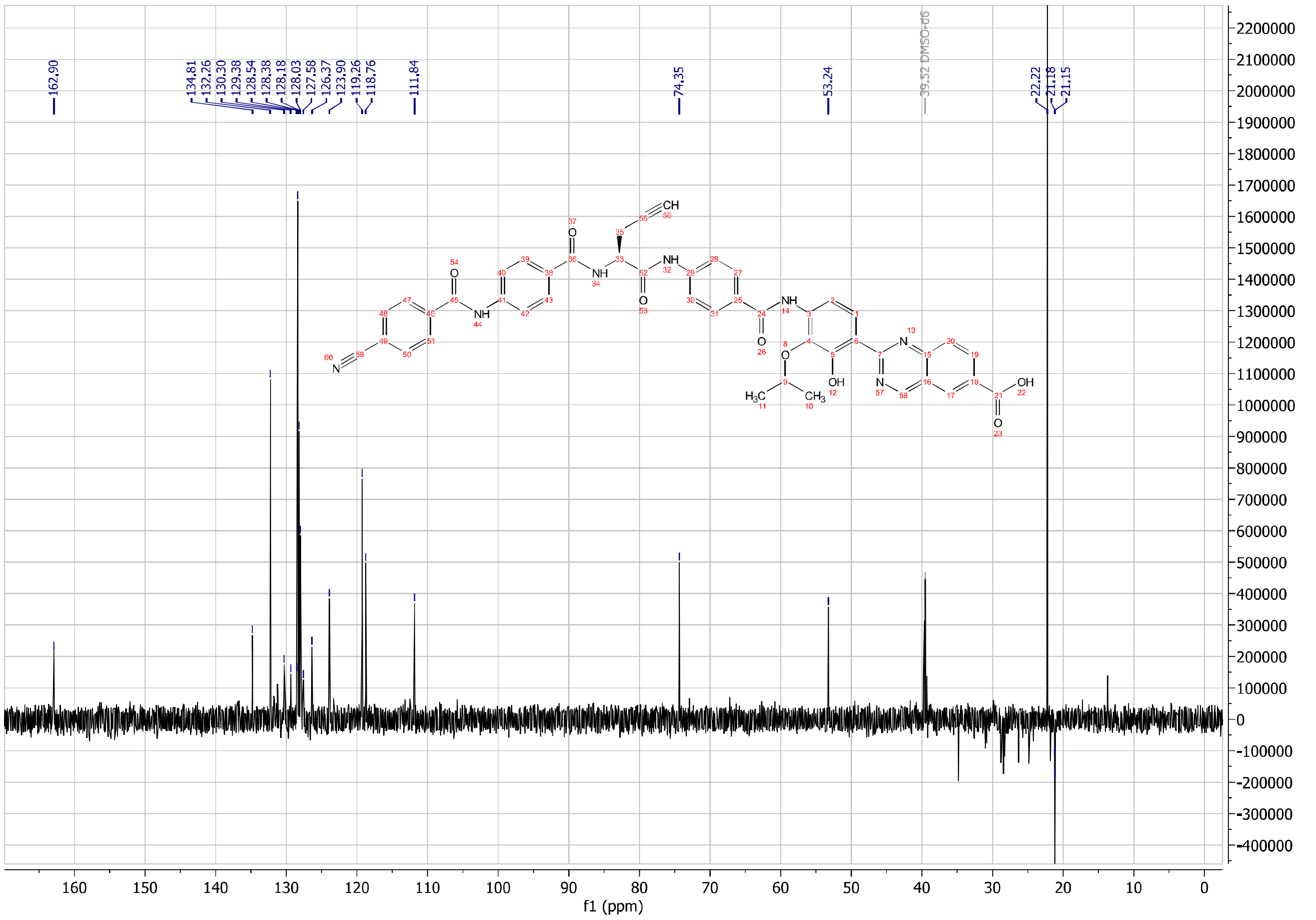

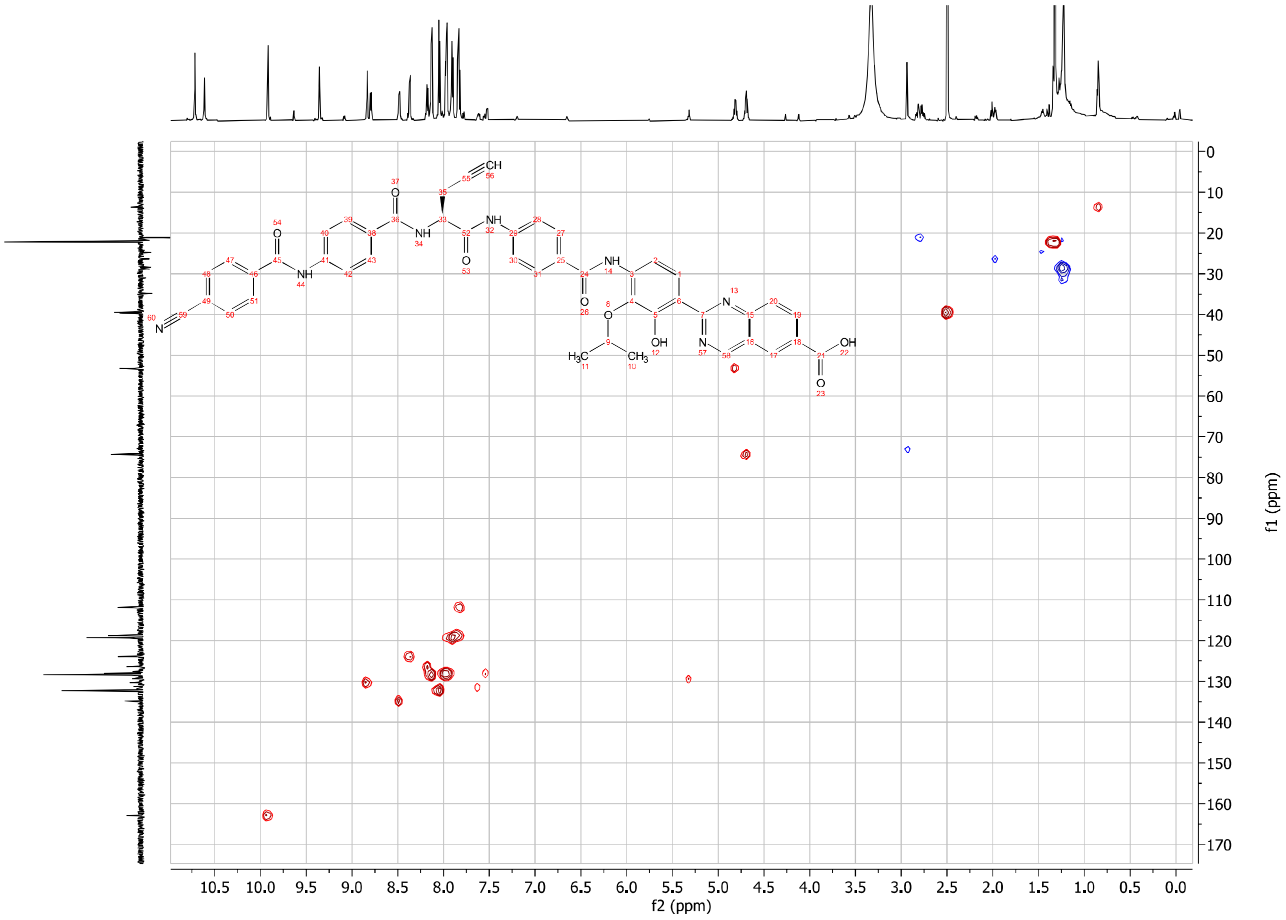
f1 (ppm)
f2 (ppm)
CH
NH
OH
N
O
H3C
CH3

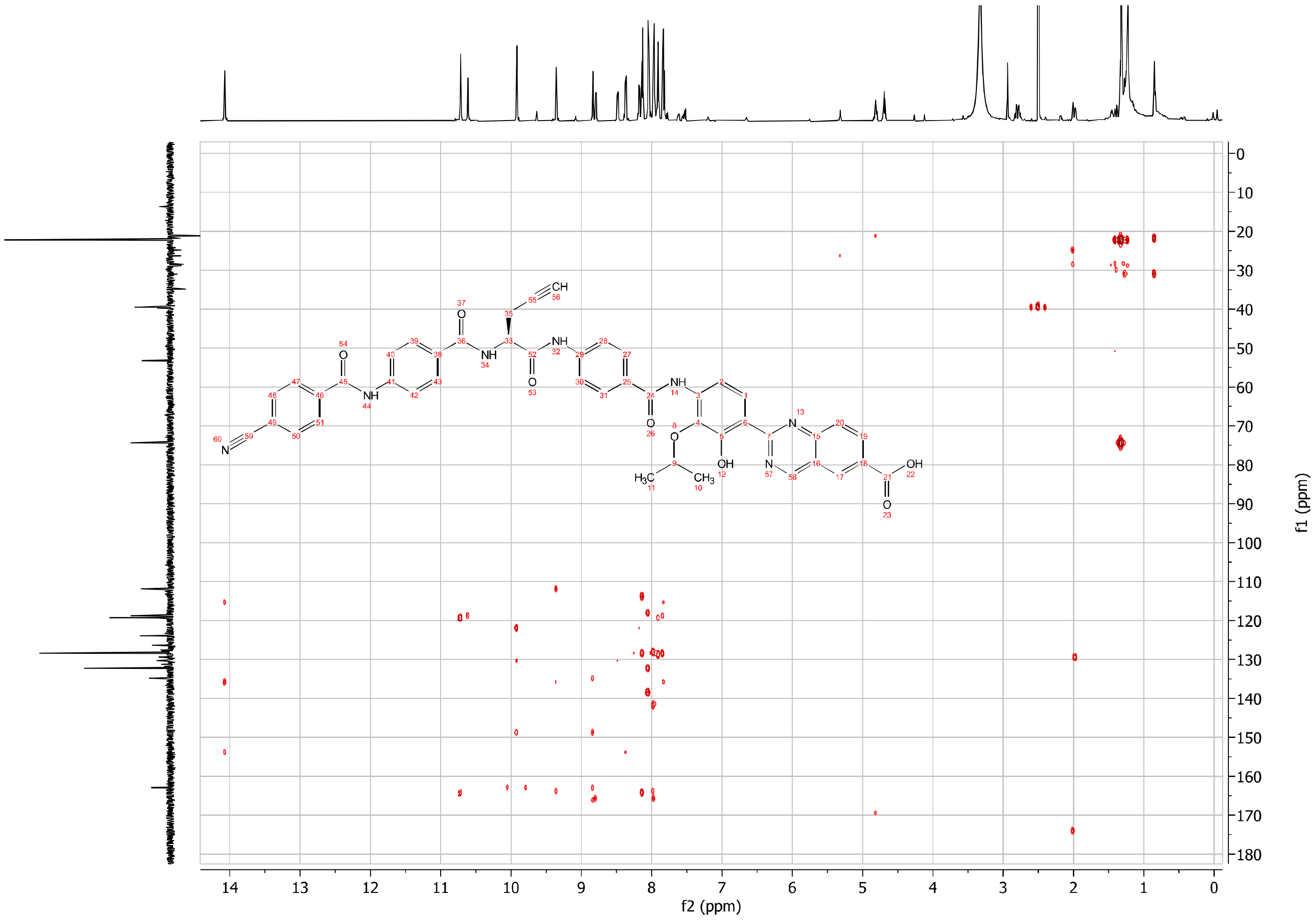
f1 (ppm)
f2 (ppm)
CH
NH
OH
N
O
H3C
CH3

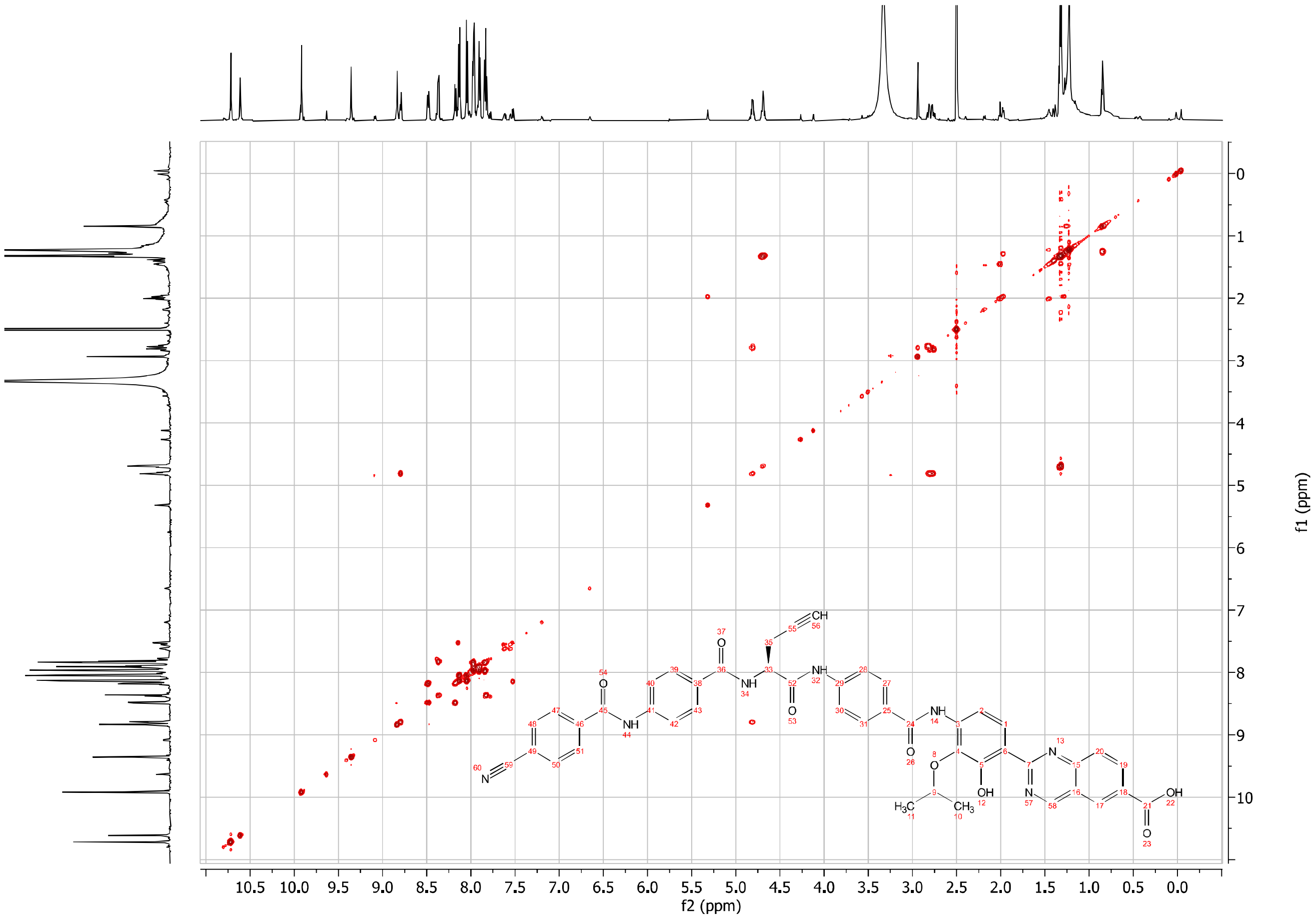
f2 (ppm)
f1 (ppm)

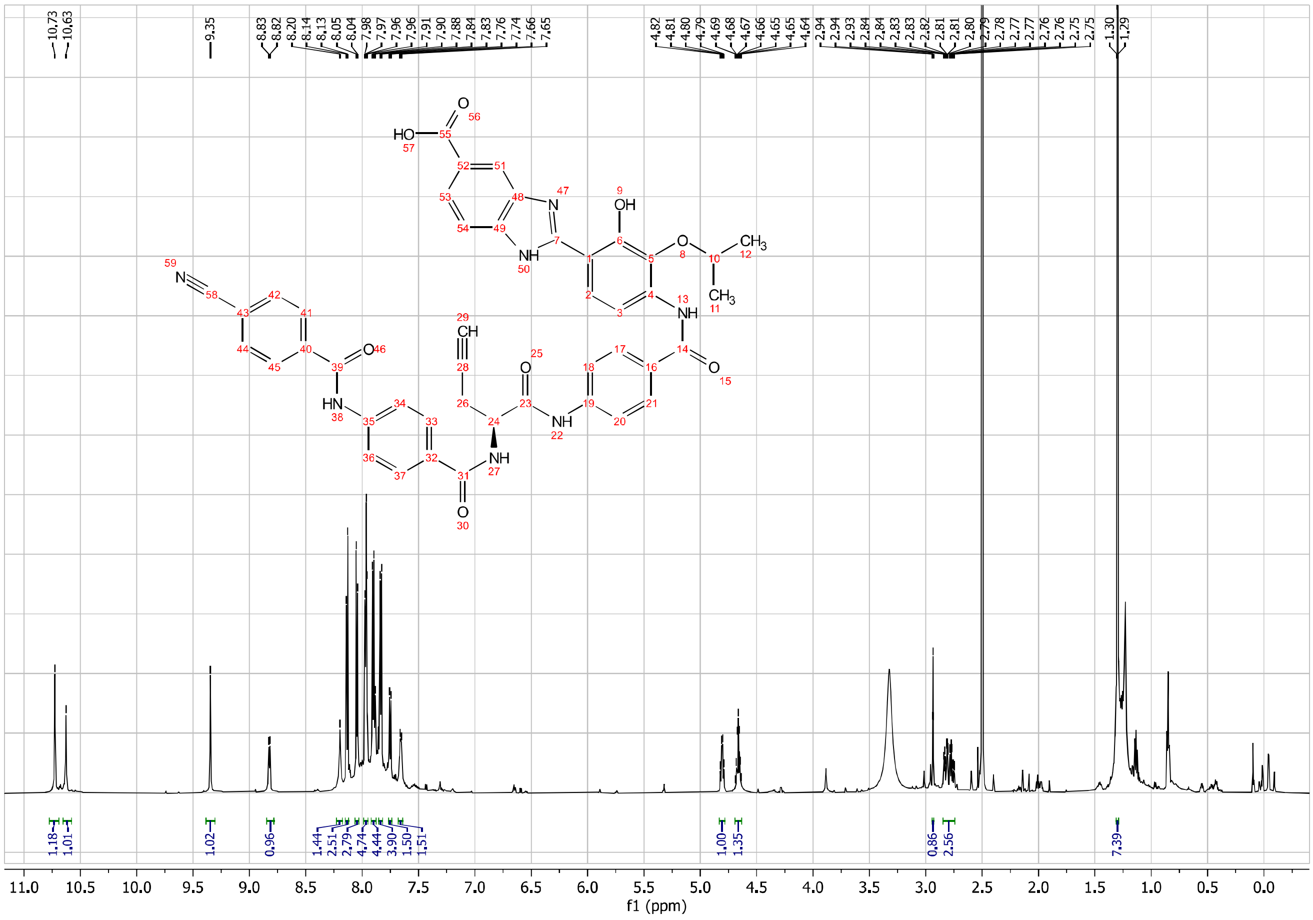

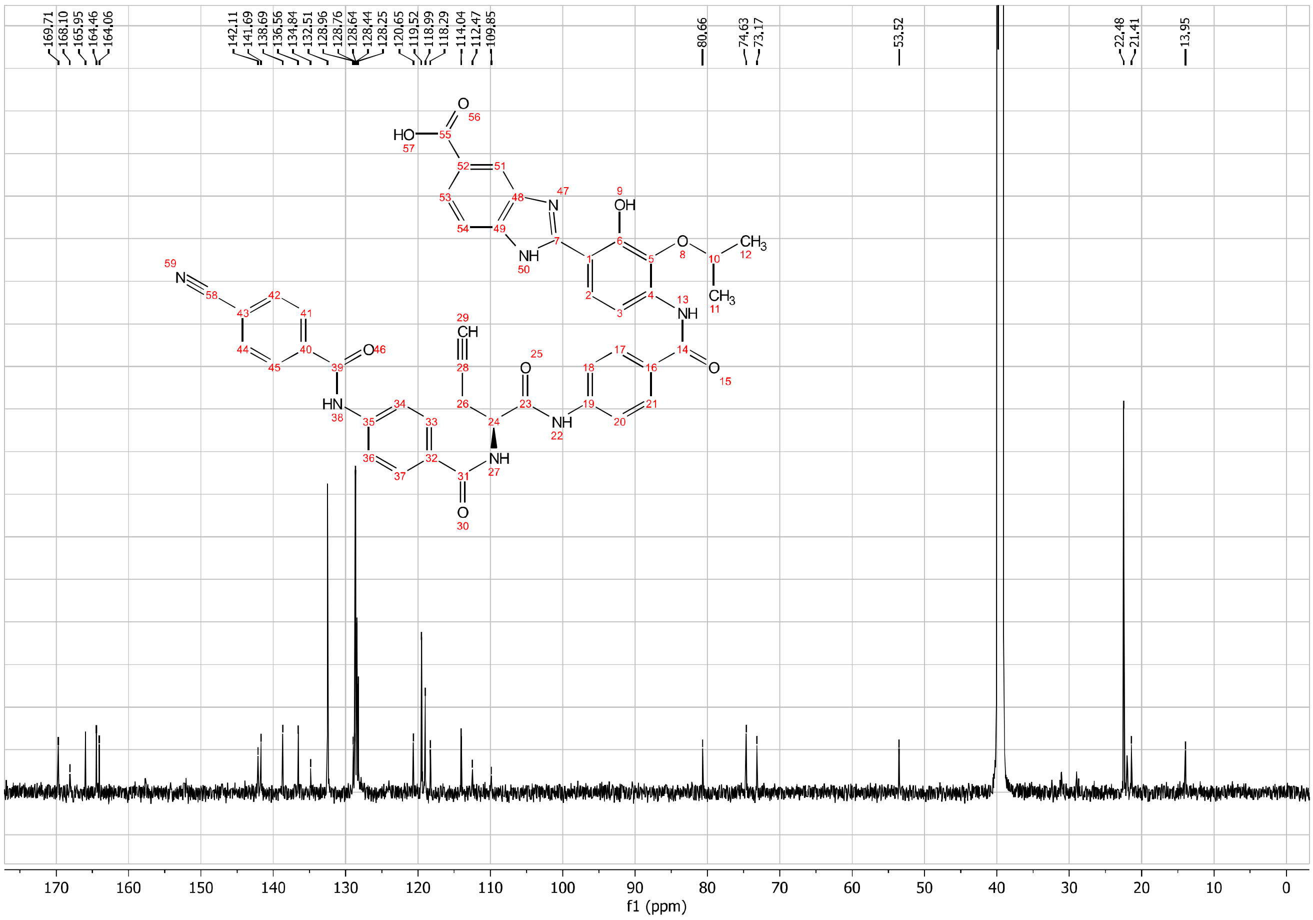

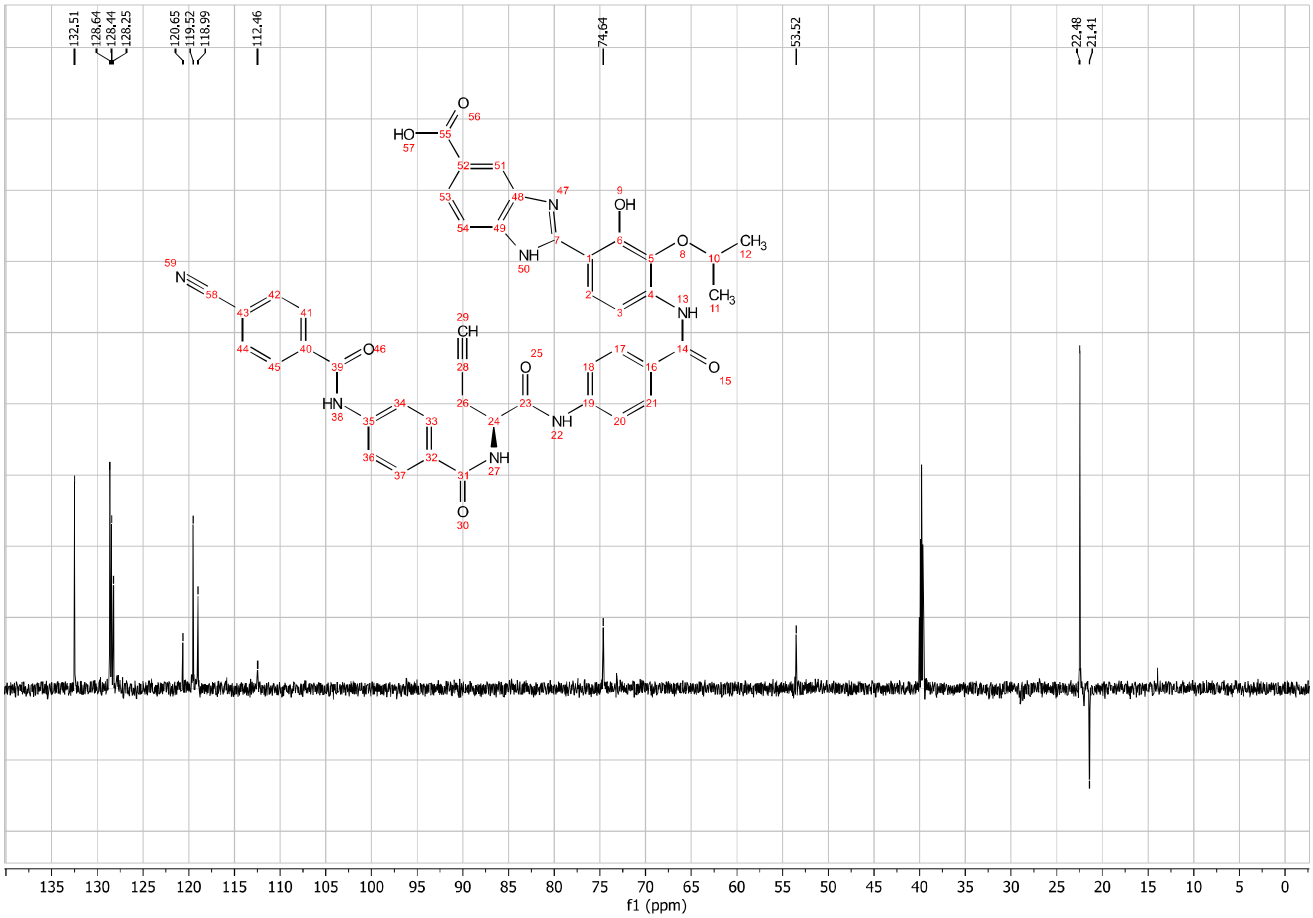

132.51
128.64
128.44
128.25
120.65
119.52
118.99
112.46
74.64
53.52
22.48
21.41
f1 (ppm)
